# Human and Microbial Proteins From Corpora Amylacea of Alzheimer’s Disease

**DOI:** 10.1038/s41598-018-28231-1

**Published:** 2018-06-29

**Authors:** Diana Pisa, Ruth Alonso, Ana Isabel Marina, Alberto Rábano, Luis Carrasco

**Affiliations:** 1 Centro de Biología Molecular “Severo Ochoa” (CSIC-UAM). c/Nicolás Cabrera, 1. Universidad Autónoma de Madrid. Cantoblanco., 28049 Madrid, Spain; 2 Department of Neuropathology and Tissue Bank, Unidad de Investigación Proyecto Alzheimer, Fundación CIEN, Instituto de Salud Carlos III, Madrid, Spain

## Abstract

Corpora amylacea (CA) are spherical bodies mainly composed of polyglucans and, to a lesser extent, proteins. They are abundant in brains from patients with neurodegenerative diseases, particularly Alzheimer’s disease. Although CA were discovered many years ago, their precise origin and function remain obscure. CA from the insular cortex of two Alzheimer’s patients were purified and the protein composition was assessed by proteomic analysis. A number of microbial proteins were identified and fungal DNA was detected by nested PCR.A wide variety of human proteins form part of CA. In addition, we unequivocally demonstrated several fungal and bacterial proteins in purified CA. In addition to a variety of human proteins, CA also contain fungal and bacterial polypeptides.In conclusion, this paper suggests that the function of CA is to scavenge cellular debris provoked by microbial infections.

## Introduction

Several different types of polyglucan bodies have been found in the central nervous system (CNS) of elderly people, as well as in patients with a variety of pathologies, including Alzheimer’s disease (AD), epilepsy, amyotrophic lateral sclerosis (ALS), multiple sclerosis (MS) and hippocampal sclerosis^1,2^. Among these polyglucan structures, Lafora bodies, Bielschowsky bodies and corpora amylacea (CA) have been identified based on their morphological characteristics^3–5^. Although CA were described early in the 18th century, their precise origin and potential function in normal or pathological conditions remains an enigma. The presence of CA in brain tissue was first described by Purkinje in 1837, and the Cajal school analyzed these structures, suggesting that they are formed by the microglia.

CA are amorphous rounded bodies approximately 10−50 μm in diameter. These glycoproteinaceous laminar structures accumulate in the brain during the course of normal aging and to a greater extent in the CNS of AD patients^6–8^. Of note, abundant CA are also found in some patients diagnosed with temporal epilepsy, where extensive deposits of CA have been observed in brain tissue^9,10^. Interestingly, CA have a perivascular distribution and are much more abundant in close proximity to blood vessels^10–12^. In addition to the CNS, CA are found in other organs and tissues, such as normal prostate, muscle, liver, lung, prostate tumours and other malignant tissues^13–18^. CA mostly contain polyglucans (over 85% are hexoses), with a minor component (4%) of proteins^19^. The rounded core is formed by different calcium salts, principally calcium phosphate and calcium oxalate depending on the bodies analyzed^20–22^. In general, a broad range of proteins can be found in CA and a number of them have been characterized using specific antibodies^23^. Accordingly, several blood proteins such as thrombospondin 1, some complement components, ubiquitin, heat-shock proteins and tau protein processed by caspase-3 have been detected in CA^24–28^. The protein composition of CA from prostate has been characterized in detail by proteomic analysis, showing that lactoferrin is the most abundant protein, together with myeloperoxidase, S100 calcium-binding proteins A8 and A9, which form the inflammatory protein calprotectin, and α-defensins, which form part of neutrophil granules^29^. Curiously, calprotectin is also present in CA from normal human brains^30^. The suggestion that CA are built up from breakdown products from neurons and oligodendroglial cells has been proposed^23^. Along this line, proteomic analysis of brain CA from patients with MS revealed the presence of cytoskeleton proteins and enzymes of the anaerobic glycolysis pathway . A number of microorganisms have also been suggested as the source of the chronic inflammation that triggers the formation of CA in prostate. Among these, several bacteria such as *Chlamydia trachomatis*, *Escherichia coli* and *Pseudomonas* spp., protozoa such as *Trichomonas vaginalis* and viruses known to contribute to different types of cancer, including human papillomavirus, have been considered^29,31^. Thus, the concept that CA represent the prostate response to a microbial infection has been proposed.

We have recently reported that CA from brain tissue of AD, ALS and patients with Parkinson’s disease contain fungal components, as revealed by immunoreactivity against antifungal antibodies^32^. However, the exact proteins present in CA were not identified. We have proposed that CA represent a response to the microbial infection present in these patients. Thus, this proposal reconciles the idea that CA are built up by cellular breakdown products, which originate from the microbial infection that exists in the CNS from patients with these neurodegenerative diseases. Indeed, there is strong evidence for the presence of fungal proteins and DNA in CNS from AD patients^33–35^, and also in patients diagnosed with ALS^36,37^. In the current study, we have characterized in detail the fungal and bacterial proteins that can be found in purified CA from two AD patients. In addition, the human proteins present in CA reveal a great variety of cellular polypeptides, consistent with the concept that CA represent breakdown products of neural cells.

## Results

### Purification of CA from brain tissue

**Figure 1:**
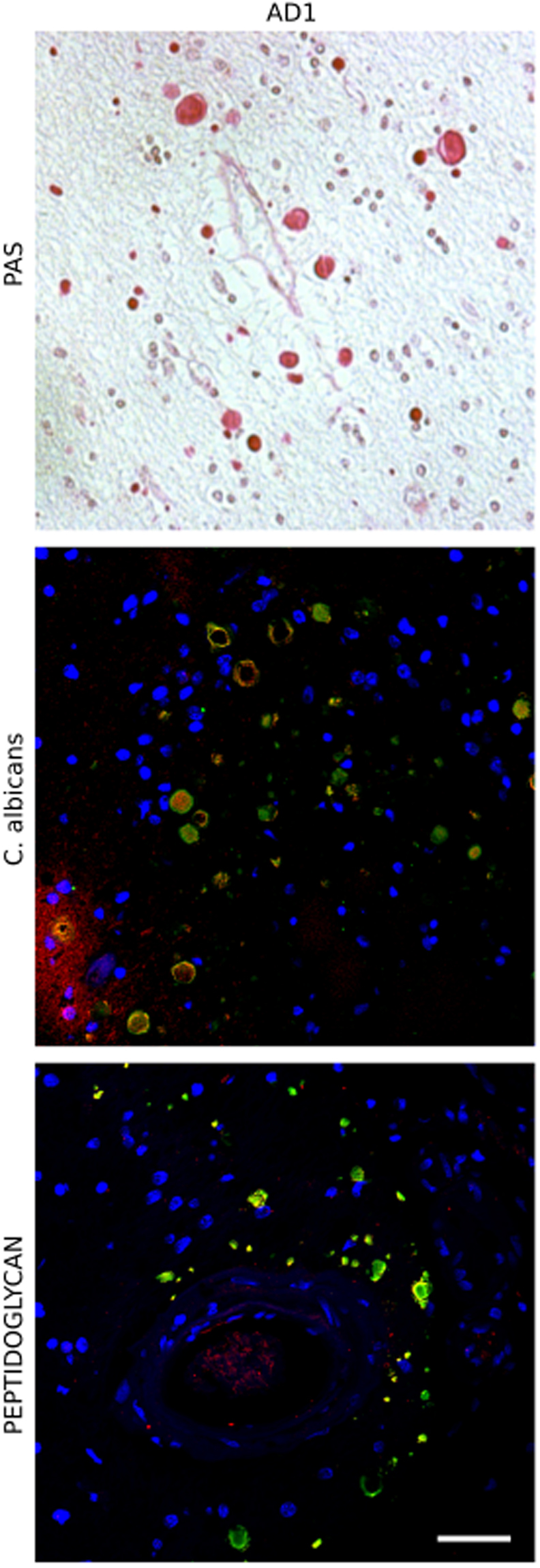
Histochemistry analysis of brain sections from patient AD1. Evidence of CA in the brain cortex containing microbial proteins. Insula cortex (INCO) sections from patient AD1 stained with Periodic acid–Schiff reagent (upper panel); immunostained with a rabbit polyclonal anti-*C. albicans* antibody (green) used at 1:100 dilution, and a mouse monoclonal anti-human α-tubulin antibody (red) used at 1:50 dilution (middle panel); immunostained with a mouse monoclonal anti-peptidoglycan antibody (green) used at 1:20 dilution, and a rabbit polyclonal anti-*C*. albicans antibody (red) used at 1:100 dilution (lower panel). DAPI staining of nuclei appears in blue. Scale bar: 50 μm.

Our initial goal was to develop a protocol to purify CA from brain tissue. We analyzed tissue from two patients (AD1 and AD2) diagnosed with AD; patient AD2 was also diagnosed with dementia with Lewy bodies. Prior results from our group have demonstrated the existence of fungal proteins in CA from brain sections of AD patients^32^. Accordingly, the presence of CA was first examined by periodic acid-Schiff (PAS) staining and by immunohistochemistry using antibodies against human α-tubulin, *C. albicans* and also bacterial peptidoglycan. Abundant CA were observed in insular cortex (INCO) tissue sections in patient AD1 by PAS staining and by immunohistochemistry (Fig. 1). For this reason, brain tissue from this INCO region was selected. Interestingly, CA immunoreacted with both antifungal and antibacterial antibodies, suggesting the presence of microbial components in CA. Similar results were found in patient AD2, although CA were less abundant in the sections examined from this patient (Supplementary Fig. 1A).

To obtain purified CA, we used the protocol described in Materials and Methods. Homogenized tissue was pelleted through a series of centrifugation steps on sucrose cushions to obtain a final pellet named P7. The proteins of the first homogenate and the P7 fraction were analyzed by SDS-PAGE under reducing conditions and visualized by Coomassie blue staining. Analysis showed evident differences in protein content between the first homogenate and the P7 pellet (Supplementary Fig. 1B). In addition, the P7 pellet was immunostained with an anti-*C. albicans* antibody to visualize the purified CA in this fraction. Notably, CA was evidenced in P7 from both patients after immunostaining with the fungal antibody (Supplementary Fig. 1C). These findings clearly indicate that P7 contains CA bodies, but we cannot rule out the possibility that other human proteins present in organelles also form part of this fraction. It is unlikely that soluble cellular proteins obtained during the homogenization procedure remain in P7, since the components of this fraction were sedimented through 25–45% sucrose and this step was repeated several times. However, it should be possible that soluble cellular proteins interact with CA during their formation. To test this possibility directly, we subjected fractions H and P7 to western blotting using an antibody against the translation initiation factor eIF4GI. This factor was clearly detected in fraction H, but not in P7 (Supplementary Fig. 2A, see also Supplementary info file). Furthermore, whereas the translation elongation factor eEF2 was detected only sparingly in fraction H, CA clearly accumulated this protein. Also, human α-tubulin was found both in fractions H and P7. Interestingly, 18S rRNA was detected by RT-PCR in the P7 fraction from both patients. By contrast, mtDNA was only found in the H and P2 fractions, but not in P7, reflecting that mitochondria do not co-purify with CA and that these organelles are not recruited by CA (Supplementary Fig. 2B, see also Supplementary info file).

As regards to CA from control subjects, their amount was very low (Supplementary Fig. 3). Only some CA can be revealed in the entorhinal cortex/hippocampus (ERH) using antibodies against human α-tubulin. Therefore, purification of these bodies by our present protocol was precluded. The best characterization of CA from control subjects can be accomplished by immunohistochemistry. However, immunolabeling with anti-*C. albicans* antibodies was not found (Supplementary Fig. 3). Besides, no signal was observed with anti-peptidoglycan antibodies. These findings are in good agreement with previous results^38^.

### Human proteins detected in purified CA

**Figure 2:**
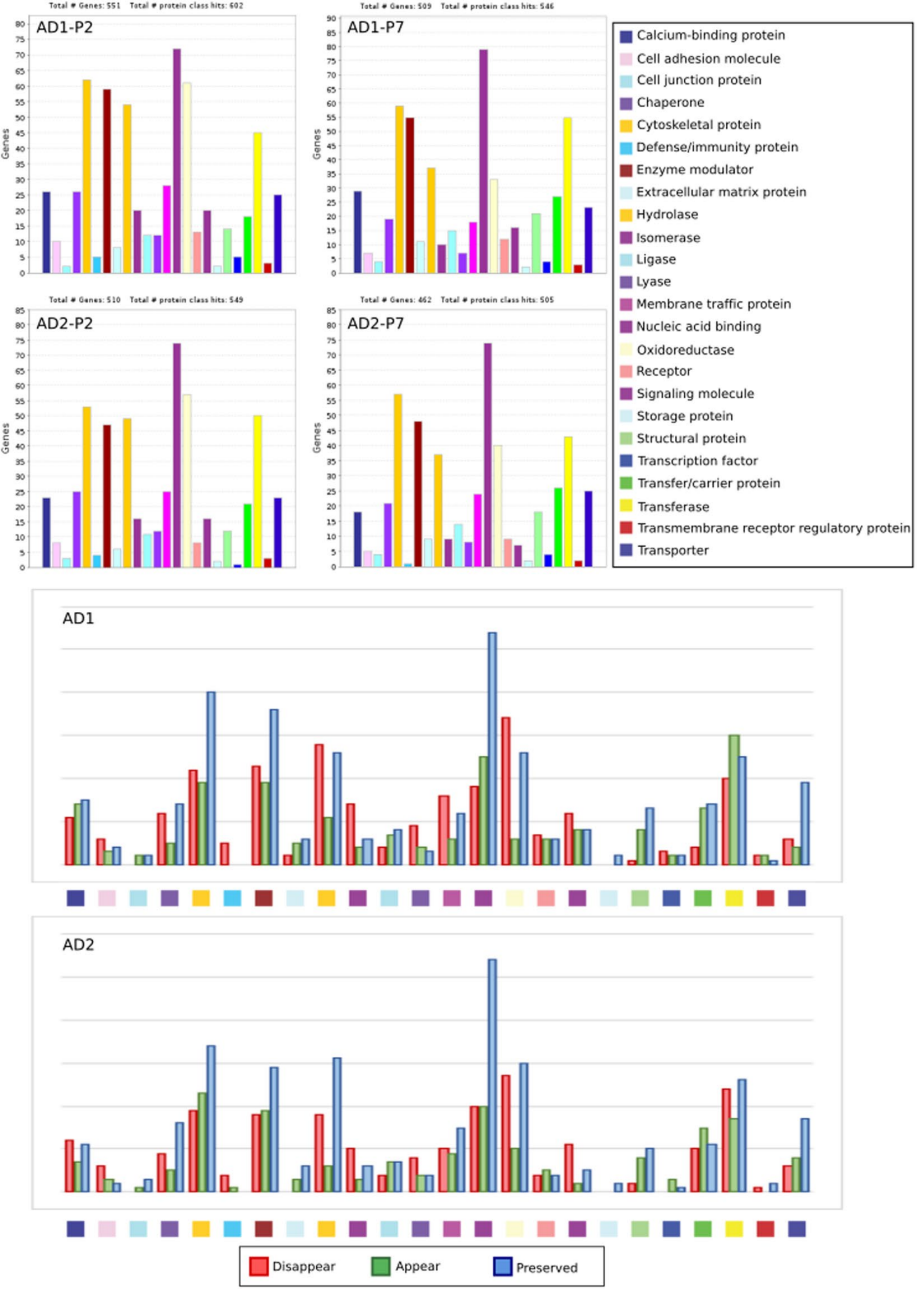
Proteomic analysis of P2 and P7 fractions from patients AD1 and AD2. Identification of the human proteins present in these fractions. Analysis of the human proteins in CA (fraction P7) by proteomics. Classification of the proteins found in P2 and P7 according to protein class function was carried out using Panther online software. Upper panels: P2 and P7 from AD1, and P2 and P7 from AD2. Lower panels: comparison of the proteins that appear in P2 and P7 from AD1 and AD2. Red: proteins absent in P7 and present in P2. Green: proteins present in P7 and absent in P2. Blue: proteins common to both P2 and P7.

After determining that P7 contains CA, we next sought to characterize this fraction using proteomic analysis. Initially, we sought to identify the human proteins that constitute CA. To do this, CA samples were digested with trypsin and analyzed by MS. Supplementary Table I and Table II list the human proteins that are found in P2 and P7 fractions from both patient AD1 and AD2, with at least two peptides. The human proteins detected were classified according to their function (Fig. 2). Most of these peptides belong to nucleic acid binding proteins, cytoskeleton and enzymatic proteins. Interestingly, a number of these proteins were detected in P2, but not in P7 and vice versa, while others were common to both fractions (Supplementary Tables III and IV). The major finding of this proteomic analysis was the great variety of human proteins detected in P7, presumably forming part of CA. Evidently, some of these proteins could be minor components of CA and/or could be present in a small fraction of these bodies due to their heterogeneity.

### Fungal proteins in CA

**Figure 3:**
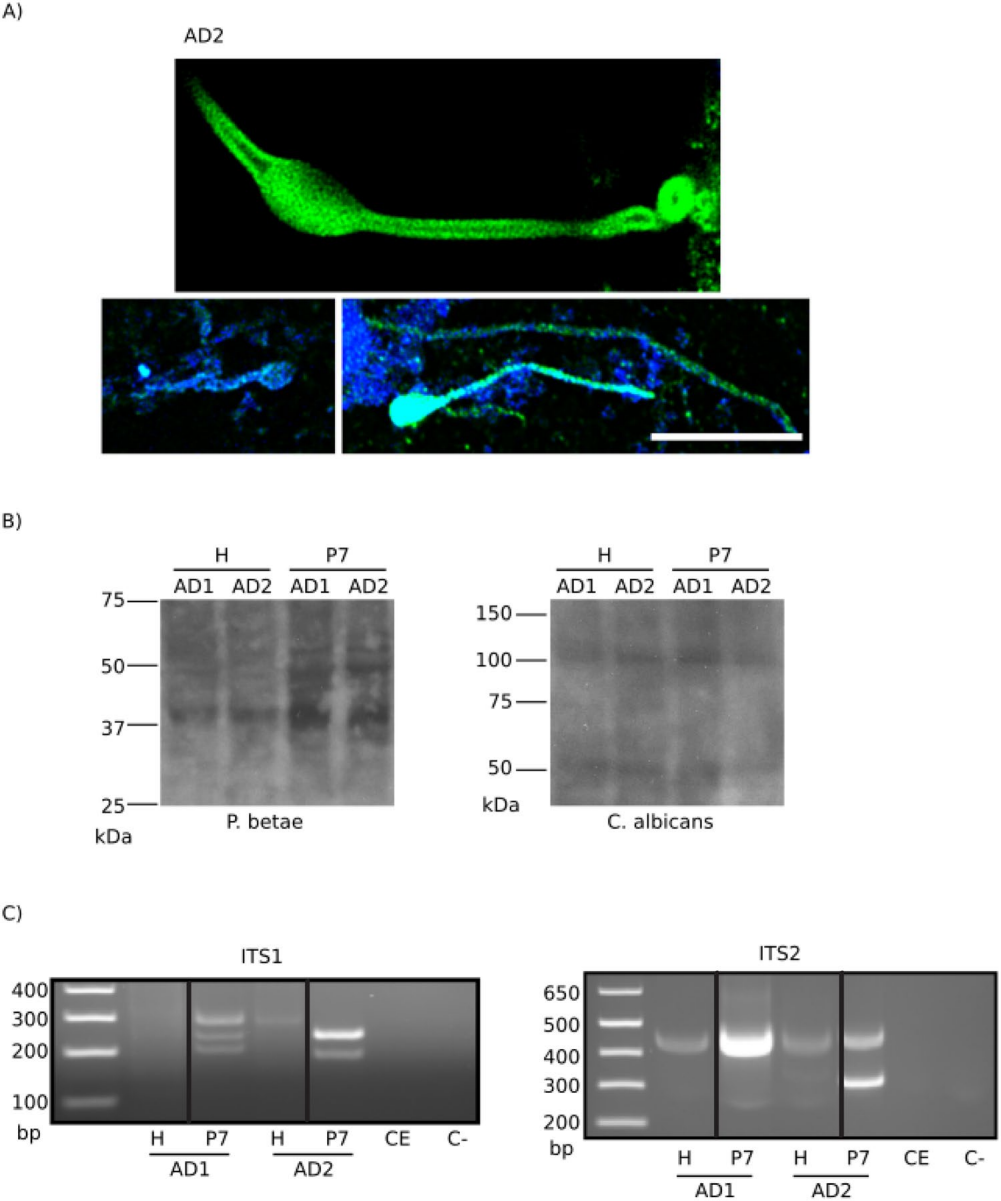
Fungal proteins and DNA in the homogenate and P7 fractions from patients AD1 and AD2. Identification of fungal components in CA. (**A**) Pellet (P2) of AD2 immunostained with a rabbit polyclonal anti-*C. albicans* antibody (green) used at 1:100 dilution. DAPI staining of nuclei appears in blue. Scale bar: 10 μm. (**B**) Western blot of proteins from homogenate (H) and P7 fractions from AD1 and AD2. Left panel: Rabbit polyclonal anti-*P. betae* antibody used at 1:500 dilution; right panel: rabbit polyclonal anti-*C. albicans* antibody used at 1:100 dilution. (**C**) Nested PCR analysis of DNA extracted from patients AD1 and AD2. Agarose gel electrophoresis of the DNA fragments amplified by nested PCR. Left panel: PCR analysis of the homogenate (H) and P7 fractions from patients AD1 and AD2 using primers ITS-5 and ITS-3 to amplify the ITS-1 region. Right panel: PCR analysis of the H and P7 fractions from patients AD1 and AD2 using primers ITS-3 and ITS-4 to amplify the ITS-2 region. (**C**) Control of PCR without DNA. CE: Control of DNA extraction without DNA. H: Homogenate. P7: fraction 7. See also Supplementary info file.

As stated above, fungal infection can be evidenced in AD brains by immunohistochemistry^34,39^, which is consistent with the detection of fungal proteins in CA from AD1 and AD2 (Fig. 1 and Supplementary Figure 1). We therefore investigated the presence of mycotic structures in the different fractions of CA. We found that the P2 fraction from both patients contained a significant proportion of yeast-like and hyphal structures that could be detected by immunostaining with a specific anti-*C. albicans* antibody (Fig. 3A). We also analyzed the fractions by western blotting using different antifungal antibodies. Figure 3B (see also Supplementary info file) shows a number of different protein bands that immunoreacted with the anti-*Phoma betae* and anti- *C. albicans* antibodies. To identify the fungal species present in the two patients, we performed nested PCR of the fungal ITS-1 and ITS-2 regions (Fig. 3C, see also Supplementary info file). Fungal DNA fragments were successfully amplified in fractions H and P7, which after sequencing, revealed a variety of fungal species present in both patients (Supplementary Table V). The fungal genera identified included *Cladosporium*, *Malassezia* or *Rhodotorula*. These fungal genera correspond with those previously described by our group^34,40^.

The presence of fungal proteins in CA as revealed by immunohistochemistry using different antibodies does not identify the precise proteins in these bodies^32^ (Fig. 1). Thus, to demonstrate that fungal proteins form part of CA and to identify them with precision, the peptides obtained after tryptic digestion of P2 and P7 were analyzed against the fungi database using the Proteome Discoverer 1.4 tool. Obviously, the vast majority of peptides obtained after tryptic digestion were of human origin and we only considered *bona fide* peptides belonging to fungi. Through this analysis, several fungal peptides from P2 and P7 could be unambiguously ascribed in the most part to cytoskeleton proteins (Table 1). It seems logical that the most abundant proteins in fungal cells, i.e., cytoskeletal proteins, appear in CA.

**Table 1:** Fungal peptides in P2 and P7 fractions from AD1 and AD2.

PEPTIDE	PROTEIN	SPECIES	AD1-P2	AD1-P7	AD2-P2	AD2-P7
GHYTEGAELIDSVLDVVR	Beta tubulin	Several species	—	YES	YES	YES
GHYTEGAELVEAVLDVVR	Beta tubulin	Several species	YES	—	—	—
MAATFIGNSTAQQELFK	Beta tubulin	Several species	—	YES	—	YES
MSATFIGNSTSIQELFK	Beta tubulin	Several species	—	—	YES	—
MSVTFIGNSTAIQELFK	Beta tubulin	Several species	—	—	YES	YES
MSVTFLGNSTAIQELFK	Beta tubulin	Several species	—	YES	—	—
MSVTLIGNSTAIQELFK	Beta tubulin	Rhizophlyctis rosea	YES	—	—	—
NSSYYVEWIPNNVK	Beta tubulin	Blastocladiella emersonii	—	YES	—	—
SLGGGTGAGMGTLLISK	Beta tubulin	Several species	YES	—	—	—
AICMLSNTTAIAEAWAR	Alpha tubulin	Lichtheimia sp	—	—	YES	—
ALCMLSNTTAIAEAWAR	Alpha tubulin	Several species	YES	YES	—	YES
AVCMLSNTTAIAEAWSR	Alpha tubulin	Several species	—	—	—	YES
AVCMLSNTTAISEAWSR	Alpha tubulin	Geotrichum candidum	—	YES	—	YES
IHFPLATYAPIISAEK	Alpha tubulin	Several species	—	YES	YES	—
IHFPLATYAPLISADK	Alpha tubulin	Several species	—	YES	—	—
IHFPLATYAPLLSAEK	Alpha tubulin	Several species	YES	—	—	YES
LIAQVVSSITASLR	Alpha tubulin	Several species	YES	—	YES	—
DSYVGDEAESK	Actin	Fusarium oxysporum	—	—	YES	—
GEEEVAALVIDNGSGMCK	Actin	Cryptococcus depauperatus	YES	YES	YES	YES
SYELPDGQNITIGNER	Actin	Nematocida sp	YES	YES	YES	—
TYELPDGQVITIGNER	Actin	Several species	—	—	—	YES
LILEVAQHLGESTVR	ATP synthase subunit beta	Ophiostoma piceae	YES	—	YES	—
VALTGLTIAEYFR	ATP synthase subunit beta	Several species	YES	—	YES	—
VSLVFGQMNEPPGAR	ATP synthase subunit beta	Several species	YES	—	—	—
IEIIANDQGNR	Heat shock protein	Several species	YES	—	YES	—
SLTNDWEEHLAVK	Heat shock protein	Several species	YES	—	—	—
FFTPEEISSMVLTK	Unplaced genomic scaffold	Several species	—	—	YES	—

### Bacterial proteins detected in purified CA

To complement our studies on human and fungal proteins in CA, we extended our analysis to the possibility that viral or bacterial proteins are also present in these samples. Indeed, we and others have recently reported that a variety of bacterial species are detected in brain tissue from AD patients^39,41^. Moreover, prokaryotic-like cells immunopositive for peptidoglycan could be detected in INCO tissue sections (Fig. 1). To test the existence of bacterial proteins, the peptides obtained in P7 from both AD1 and AD2 patients were tested against bacterial databases belonging to different phyla. Only those peptides that unequivocally belong to bacteria were considered. Interestingly, the high sensitivity of the proteomic analyses identified a few peptides that could be ascribed to bacteria. The bacterial peptides, the corresponding proteins and the phyla are listed in Table 2. Only three prokaryotic peptides were found in AD1 and one in AD2. Of note, the number of bacterial peptides detected was lower than those of fungal origin, possibly reflecting the lower burden of bacterial infection as compared with mycotic infection. Finally, the peptides obtained after tryptic digestion of P2 and P7 were also analyzed against the database of DNA animal viruses to identify potential proteins from these viruses. No peptides corresponding to herpesviruses or any other DNA virus were found in P2 or P7 from both AD patients, which is consistent with our recent report that HSV-1 proteins are not detected in brain tissue from AD patients^39^.

**Table 2:** Bacterial peptides in P2 and P7 fractions from AD1 and AD2.

PEPTIDE	PROTEIN	AD1-P7	AD2-P7	PHYLUM
LINEPTAAALAYGLSR	Chaperone protein DnaK	YES	—	proteobacteria
LLNEPTAAALAYGVEK	Chaperone protein HscA	—	YES	proteobacteria
FTQAGSEISALLGR	ATP synthase subunit beta	YES	—	proteobacteria tenericutes
NETDDQVTIDAAEATKK	Isocitrate dehydrogenase [NADP]	YES	—	firmicutes

### In-depth analysis of fungal proteins

Our finding that fungal proteins are present in a lower proportion than human proteins prompted us to analyze in more detail these proteins in the P7 fraction from both patients. To do this, proteins were fractionated by a high-pH, reversed-phase fractionation spin column. Nine fractions were obtained and were subsequently pooled into three fractions F1, F2 and F3, as described in Materials and Methods. After MS analyses of F1, F2 and F3, the number of fungal peptides detected increased considerably (Table 3). Thus, the number of peptides unambiguously identified as fungal were 49 in P7 from AD1 and 70 in P7 from AD2, from those 23 were common to both patients. These peptides corresponded to a number of fungal proteins mostly belonging to tubulins and actins, in agreement with the fact that these proteins are very abundant. These findings reinforce our previous observations that fungal infection exists in AD patients.

**Table 3:** Fungal proteins after column fractionation in P7 fractions.

PEPTIDE	PROTEIN	SPECIES	AD1-P7	Fract. AD1-P7	AD2-P7	Fract. AD2-P7
MSGTFIGDSTAIQELFK	Beta tubulin	Absidia spinosa	—	—	YES	F1
MSGTFIGNSTAIQELFK	Beta tubulin	Absidia spinosa	—	—	YES	F1
AILVDLEVATMDAVR	Beta tubulin	Agaricus bisporus var. Burnettii	—	—	YES	F3
AVLVDLEPGTMDTTR	Beta tubulin	Basidiobolus microsporus	YES	F1	YES	F3-F3
MAVTFVGNSTAIQELFK	Beta tubulin	Basidiobolus microsporus	YES	F1	—	—
NSSYYVEWIPNNVK	Beta tubulin	Blastocladiella emersonii	YES	F3	—	—
LGICDEPPTVVPGGDLAK	Alpha tubulin	Calocera sp.	YES	F1	—	—
EVEDQMLSVQTK	Beta tubulin	Claviceps purpurea	YES	F3	—	—
IAEQFTAMFR	Beta tubulin	Conidiobolus coronatus	YES	F1	—	—
MCSTFIGNSTAIQELFK	Beta tubulin	Conidiobolus coronatus	YES	F1	YES	F3
CVSMLSNTTAIAEAWAR	Alpha tubulin	Dactylellina haptotyla	YES	F1	YES	F1-F2
LEAEAAAAAAAAAK	Uncharacterized protein	Daedalea quercina	—	—	YES	F1
EVEEQMLAVQTK	Beta tubulin	Dentiscutata heterogama	YES	F1	YES	F3
MSATFIGNTTSIQEPFK	Beta tubulin	Geranomyces variabilis	—	—	YES	F2
YLVVNADEGEPQTCK	NADH dehydrogenase	Gonapodya prolifera	YES	F2	YES	F3
AICMLSNTTAIAEAWAR	Alpha tubulin	Lichtheimia sp	YES	F3	YES	F3
SLFHPEQLITGK	Alpha tubulin	Lichtheimia sp.	YES	F3	—	—
AVLVDLEPGTMDNVR	Beta tubulin	Microsporidia sp.	YES	F3	—	—
AVSIPELTQQMFDAK	Beta tubulin	Mycena chlorophos	—	—	YES	F3
ISVYYNEAGASK	Beta tubulin	Mycena chlorophos	YES	F2	—	—
EVDVQMLNVQNK	Beta tubulin	Nowakowskiella elegans	YES	F1	—	—
ALTVPELAQQMFDAK	Beta tubulin	Nowakowskiella hemisphaerospora	YES	F1	YES	F1
LILEVAQHLGESTVR	ATP synthase subunit beta	Ophiostoma piceae	—	—	YES	F2
KSIQFVDWCPTGFK	Alpha tubulin	Parasitella parasitica	—	—	YES	F1
IESLEEEIRK	Uncharacterized protein	Parasitella parasitica	—	—	YES	F1
EIAESFLGK	Heat shock protein 70	Penicillium sp.	—	—	YES	F3
EVDEQMLNVHNK	Beta tubulin	Phlyctochytrium californicum	—	—	YES	F2
AVCMLGNTTAIAEAWAR	Alpha tubulin	Phycomyces blakesleeanus	YES	F1	—	—
LVVIGDSGVGK	Uncharacterized protein	Piloderma croceum	YES	F2	—	—
MDQGFSTFFSETGAGK	Alpha tubulin	Pisolithus tinctorius	—	—	YES	F3
GDDGFSTFFSETGAGK	Alpha tubulin	Puccinia	YES	F1	YES	F1-F2
AILIDLEPGTMDSVR	Beta tubulin	Puccinia sorghi	—	—	YES	F3
ALCMLSNTTAIAEAWSR	Alpha tubulin	Rhizopus delemar	—	—	YES	F2
EVDEQMLQVQNK	Beta tubulin	Rhopalomyces elegans	—	—	YES	F2
VGEAMEEGEFSEAR	Alpha tubulin	Schizophyllum commune	YES	F1	—	—
HQGVMVGMSNK	Actin	Schizopora paradoxa	—	—	YES	F2
SMNLTRGINLLSIAEK	19 S proteasome regulatory	Schizosaccharomyces octosporus	—	—	YES	F2
AILVDLEPGTMDAVR	Beta tubulin	Several species	—	—	YES	F1
AVLIDLEPGTMDSVR	Beta tubulin	Several species	—	—	YES	F3
AVLVDLEPGTMDAIK	Beta tubulin	Several species	—	—	YES	F1
AVLVDLEPGTMDAVR	Beta tubulin	Several species	YES	F3	YES	F1
GGGTGAGMGTLLISK	Beta tubulin	Several species	YES	F3	—	—
GHYTEGAELIDSVLDVVR	Beta tubulin	Several species	—	—	YES	F1
GTGAGMGTLLISK	Beta tubulin	Several species	YES	F1	YES	F3
INVYYNEASGGKYVPR	Beta tubulin	Several species	YES	F1	—	—
ISVYYNEASGGKYVPR	Beta tubulin	Several species	—	—	YES	F1
LAVNTVPFPR	Beta tubulin	Several species	YES	F1	—	—
LGVNMVPFPR	Beta tubulin	Several species	—	—	YES	F1
MAATFIGNSTAQQELFK	Beta tubulin	Several species	YES	F2	YES	F2
MSVTFIGNSTAIQELFK	Beta tubulin	Several species	YES	F1-F1	YES	F2
MSVTFLGNSTAIQELFK	Beta tubulin	Several species	YES	F1	—	—
NSAYFVEWIPNNVK	Beta tubulin	Several species	—	—	YES	F1
SGPFGKLFRPDNFVFGQ	Beta tubulin	Several species	—	—	YES	F2
TAICDIPPR	Beta tubulin	Several species	—	—	YES	F3
TGAGMGTLLISK	Beta tubulin	Several species	YES	F3	—	—
VLVDLEPGTMDAVR	Beta tubulin	Several species	—	—	YES	F1
VNDQFTAMFR	Beta tubulin	Several species	—	—	YES	F2
ALCMLSNTTAIAEAWAR	Alpha tubulin	Several species	—	—	YES	F2
AVCALSNTTAIAEAWSR	Alpha tubulin	Several species	—	—	YES	F2
AVCMLSNTTAIAEAWKR	Alpha tubulin	Several species	—	—	YES	F1
AVCMLSNTTAIAEAWNR	Alpha tubulin	Several species	—	—	YES	F1
AVCMLSNTTAIAEAWSR	Alpha tubulin	Several species	—	—	YES	F2
AVCMLSNTTAISEAWAR	Alpha tubulin	Several species	YES	F1	YES	F2
CVSMLSNTTAIAEAWSR	Alpha tubulin	Several species	—	—	YES	F2
DVHASVATLK	Alpha tubulin	Several species	YES	F1	—	—
IIAQVVSSITASLR	Alpha tubulin	Several species	—	—	YES	F2
LIAQIVSSITASLR	Alpha tubulin	Several species	—	—	YES	F2
RTVQFVDWCPTGFK	Alpha tubulin	Several species	YES	F3	YES	F2
SLCMLSNTTAIATAWSR	Alpha tubulin	Several species	YES	F1	YES	F1
SVTMLSNTTAIAEAWSR	Alpha tubulin	Several species	—	—	YES	F2
TIQFVEWCPTGFK	Alpha tubulin	Several species	YES	F1	YES	F1
TVQFVDWCPTGFK	Alpha tubulin	Several species	YES	F1	—	—
TVQMVDWCPTGFK	Alpha tubulin	Several species	—	—	YES	F3
VGEGMEEGEFTEAR	Alpha tubulin	Several species	YES	F2	YES	F2
DLTDYLMR	Actin	Several species	—	—	YES	F3
EEEVAALVIDNGSGMCK	Actin	Several species	YES	F3	—	—
EEEVAALVVDNGSGMCK	Actin	Several species	YES	F2	—	—
NYELPDGQVITIGNER	Actin	Several species	YES	F3	YES	F1
VAPEEHPVLLTEAPLNPR	Actin	Several species	YES	F1	—	—
IEIIANDQGNR	Heat shock protein	Several species	—	—	YES	F1
AMSIMNSFVNDIFER	Histone H2B	Several species	—	—	YES	F3
HAVSEGTRAVTK	Histone H2B	Several species	YES	F2	—	—
QDLPNAMQAAEITDK	ADP-ribosylation factor	Several species	YES	F1	YES	F1
SVCTEAGMYAIR	26 s protease regulatory	Several species	—	—	YES	F2
TFTTQETITNAESAR	Glucose-6-phosphate isomerase	Several species	YES	F1	—	—
DAGTISGLNVLR	Several proteins	Several species	—	—	YES	F1
IVLIGDSGVGK	Several proteins	Several species	—	—	YES	F2
IVNEPTAAAIAYGLDK	Several proteins	Several species	YES	F2	YES	F2
VIVLGDSGVGK	Several proteins	Several species	—	—	YES	F2
DVNAALPPSR	Alpha tubulin	Spizellomyces punctatus	YES	F3	—	—
NPDDITNEEYAAFYK	Hsp82-like protein	Spizellomyces punctatus	—	—	YES	F2
NPGYFVEWIPNNVK	Beta tubulin	Spraguea lophii	YES	F1	YES	F1
NLTERGYSFTTTAER	Actin	Suillus bovinus	—	—	YES	F1
TIQFVDWCTTGFK	Alpha tubulin	Syncephalis depressa	YES	F1	YES	F1
CVPAAVLGSGAANGAR	Putative AMP-binding enzyme	Taphrina deformans	YES	F1	YES	F1
SSENAPAIVIDNGSGMCK	Actin	Trachipleistophora hominis	YES	F3	—	—

## Discussion

Knowledge on the precise protein composition of CA from the CNS may shed light on the origin of these bodies. In this regard, our current findings provide evidence for the complexity of the proteins that form part of CA, consistent with the notion that these proteins could be debris of dead cells, or could appear in the intercellular space, or are extravasated polypeptides^12,25^. Our results show that P7 contains CA and they are likely devoid of contamination by soluble cellular proteins and mitochondria. The finding of ribosomal components in P7 suggests that ribosomes are recruited to CA when they formed. Alternatively, it should be possible that ribosomes cosediment with CA, although this possibility is unlikely due to the purification protocol employed. However, not all cellular proteins are represented in the same proportion in CA and in the CNS tissue since some of them, such as eIF4GI, appeared in the tissue homogenate but not in CA. Comparison of proteins from P2 and P7 fractions indicated that whereas some of them are common to both fractions, each fraction contains several unique protein species. Perhaps the stability of the proteins, or their propensity to interact with polyglucans or with other proteins, dictate their fate to form part of CA. Aside from this complexity, another important concept in CA is their heterogeneity. For example, immunohistochemistry analyses revealed that not all CA stained equally with a given antibody. Some of them contained a given human protein while others in the same preparation contained less or were devoid of that protein. Heterogeneity can also exist between CA from different brain regions, and more important, between those found in AD and in elderly subjects. This heterogeneity also raises questions about the concept that CA simply agglutinate proteins in a random fashion. If so, CA could be envisaged as scavengers of cellular debris products that would be generated after cell injury. In agreement with this idea, myelin injury in patients with AD leads to the degradation of myelin basic protein, which appears in CA^42^.

Our proposal that CA contain fungal proteins based on the observation that they immunoreact with antifungal antibodies^32^ is now substantiated by proteomic analysis. Moreover, we can conclude that both fungal and bacterial proteins can be found in purified fractions of CA from AD patients. We recently reported that prokaryotic-like cells and bacterial DNA can be detected in brain tissue from AD^39^, expanding our previous results of fungal infection of the CNS in these patients^32,33^. Consistent with our findings, bacterial DNA has also been recently detected by next generation sequencing in Alzheimer’s brains^41^. Moreover, in support of the idea that polymicrobial infections are present in AD CNS is the observation that the amyloid peptide exhibits antifungal and antibacterial activity^43^. In this regard, the production of amyloid has been considered as part of the innate immune system and is synthesized in brain tissue as a response to microbial infections^44^. We believe that these findings are important to understand the origin of CA and their potential function. Accordingly, we posited that CA originate from fungal infection^32^. We now extend this hypothesis to consider both fungal and bacterial infections as the trigger for CA formation. In this regard, CA may represent a response to agglutinate cellular debris provoked by cell damage that in turn is the result of microbial infection. In addition, fungal and bacterial proteins will also be agglutinated through the adhesive properties of polyglucans. The local decrease in pH as a result of these infections will also induce the precipitation of calcium salts, which will also be trapped in CA. According to our hypothesis CA in AD patients originate because there is a microbial infection in the CNS, and their function would be to remove breakdown components from human and microbial cells. However, other possibilities to explain the origin of CA could be envisaged. For instance, the modification of the blood-brain barrier by the previous formation of CA could facilitate microbial colonization of the CNS. Then, glial activation could proceed, leading to microbial destruction and the consequent accumulation of fungal proteins in CA.

The existence of CA in other tissues of the human body as well as in the CNS of elderly people without neurodegenerative diseases could also be a consequence of microbial cells that may exist in much lower amounts than those found in AD patients. Indeed, bacteria have been detected in the CNS of control human subjects^39,41,45^. These microbial cells may have a tendency to locate in close proximity to blood vessels, where oxygen and nutrients are more abundant. This is in accord with the finding that CA are distributed in perivascular areas^10–12^. Remarkably, bacterial cells have also been found in human atherosclerosis and in aortic aneurysms^46–48^. In addition, bacteria can also be detected in internal tissues, that otherwise should be “sterile”^49^. The possibility that fungi coexist with these bacteria in some human organs or tissues remains to be explored. Therefore, the presence of CA in other tissues may be a consequence of low burdens of microbial cells that, with time, may increase in number and provoke clinical symptoms. Indeed, in the case of CA from prostate, the suggestion that they are a consequence of bacterial or viral infections has been proposed^31,50^.

## Materials and Methods

### Description of subjects

The study comprised two women: one aged 92, diagnosed with AD (hereafter described as AD1), and the other aged 76, diagnosed with AD and dementia with Lewy bodies (hereafter described as AD2). The two cases were diagnosed according to current neuropathological consensus guidelines^51^. Samples were supplied by Banco de Tejidos, Fundación CIEN (Centro de Investigación de Enfermedades Neurológicas, Madrid. Spain).Brain samples from two control subjects were also tested. These two controls did not suffer any neurodegenerative disease. Control 1 was a woman aged 48, while Control 2 was a man aged 78. The brain donors were anonymous to the investigators who participated in the study. All ethico-legal documents of the brain bank, including written informed consent, were approved by an ethics committee external to the bank. The study was approved by the ethics committee of Universidad Autónoma de Madrid. The transfer of samples was carried out according to national regulations concerning research on human biological samples. In addition to the informed consents, all experiments were performed in accordance with relevant guidelines and regulations.

Frozen brain samples and paraffin-fixed sections from insular cortex (INCO) were used for DNA purification and immunohistochemistry analyses, respectively. Brain and sample processing were carried out as described previously in detail^32^. Briefly, rapid neuropathological autopsy was performed upon call by the donor’s proxies (mean post-mortem interval, 4.5 h). Immediately after brain extraction, two symmetrical brain halves were obtained in fresh through a mid-sagital cut. The right half was frozen in **−**50 °C isopentane after serial cutting of the brain hemisphere, the cerebellar hemisphere and the brainstem. The left half was fixed in 4% phosphate-buffered formaldehyde during 3 weeks, and thereafter it was cut and sampled. A total 25 tissue blocks were obtained and embedded in paraffin. Samples from the frozen tissue were obtained with sterile instruments in a laminar flow hood, taking all measures to avoid contamination.

### CA purification

INCO tissue (350 mg) was suspended in 1 ml phosphate buffered saline with calcium (PBS-Ca). The tissue was homogenized gently using a Miccra D-1 homogenizer (Miccra, Müllheim; Germany). A 30 μl aliquot of this homogenate was removed for later analysis. The remaining of the homogenate was centrifuged through a series of sucrose solutions at 20,000 × *g*. Accordingly, the pellet from the first centrifugation step (5 min) was resuspended in 300 μl PBS-Ca (P1) and loaded onto 1.5 ml of 25% sucrose in PBS-Ca. After centrifugation (10 min), the pellet was again resuspended in 300 μl PBS-Ca (P2). This pellet (P2) was treated with 1% sodium deoxycholate and the resulting suspension was loaded onto 1.5 ml 25% sucrose in PBS-Ca and centrifuged as before. This pellet was resuspended in 300 μl PBS-Ca (P3) and treated with 1,000 IU RNAse T1 (Thermoscientific, MA, USA) and 15 IU DNAse 1 (TAKARA Clontech, CA, USA) at 37 °C for 5 min. The resulting suspension was loaded onto 1.5 ml 25% sucrose in PBS-Ca and centrifuged as before. The pellet was again resuspended in 300 μl PBS-Ca (P4). This pellet (P4) was treated with 1.5% sodium deoxycholate and the resulting suspension was loaded onto 1.5 ml 35% sucrose in PBS-Ca and centrifuged as before. After this centrifugation step, this pellet was resuspended in 300 μl PBS-Ca (P5) and treated with 1,000 IU RNAse T1 and 15 IU DNAse 1 at 37 °C for 5 min. The resulting suspension was layered onto 1.5 ml of 35% sucrose in PBS-Ca and centrifuged and resuspended in 300 μl PBS-Ca to obtain pellet P6. Finally, the P6 pellet obtained was treated with 1,000 IU RNAse T1 and 15 IU DNAse 1 at 37 °C for 5 min and layered onto 1.5 ml 45% sucrose in PBS-Ca and centrifuged. It was then resuspended in 300 μl of PBS-Ca to obtain pellet P7.

### Immunohistochemistry

Tissue sections from the CNS (5 μm) were fixed in 10% buffered formalin for 24 h and embedded in paraffin following standard protocols. Methods for immunohistochemical analysis have been previously described in detail^34^.

### Western blotting

CA proteins were separated by SDS-PAGE (4–12% polyacrylamide), transferred to nitrocellulose membranes by wet immunotransfer and processed for western blotting as described^38^ after blocking the membranes with 1% bovine serum albumin. Rabbit polyclonal antibodies against eIF4GI^52^, *Candida albicans* and *Phoma betae*^34^ have been described previously. Mouse monoclonal antibodies against peptidoglycan and human α-tubulin were purchased from Sigma and Thermo Scientific, respectively. The goat polyclonal antibody against elongation factor eEF2 was purchased from Santa Cruz Biotechnology. Stripping of the nitrocellulose membrane was accomplished in some instances to test different antibodies.

### Mass Spectrometry Analysis

The methodology for mass spectrometry (MS) analysis has been described in detail elsewhere^36^. Briefly, proteins from INCO samples were separated by PAGE under reducing conditions using a 12.5% separating gel and a 5% stacking gel. Protein staining was carried out with GelCode Blue Stain Reagent (Thermo Scientific). Proteins were digested *in situ* with sequencing grade trypsin (Promega, Madison, WI). Digestion was stopped by the addition of 1% trifluroacetic acid. The desalted protein digest was dried, resuspended in 10 µl of 0.1% formic acid and analyzed by RP-LC-MS/MS in an Easy-nLC II system coupled to a LTQ-Orbitrap Velos Pro hybrid mass spectrometer (Thermo Scientific). ESI ionization was performed using a stainless steel nano-bore emitter (Proxeon, ID 30 μm). The Orbitrap mass resolution was set at 30,000.

To perform a more in-depth analysis of the peptides obtained after trypsin digestión, whole supernatants were dried down, reconstituted in 0.1% TFA and then loaded onto a high-pH, reversed-phase fractionation spin column (Pierce). A step gradient of increasing acetonitrile concentrations in a volatile high-pH solution was applied to elute bound peptides into nine different fractions. After collecting together alternate fractions into three groups (F1 = 1 + 4 + 7, F2 = 2 + 5 + 8, F3 = 3 + 6 + 9), samples were dried and stored for MS analysis. To do this, each pool of fractions was resuspended in 10 μl of 0.1% formic acid and analyzed as described above.

Processing of the MS data was carried out as previously described^36^. Database searching was performed against uniprot-homo fasta and uniprot-fungi fasta.

### Nested PCR assay

DNA was extracted from frozen tissue using the QIAmp Genomic DNA Isolation Kit (Qiagen, Hilden, Germany) as previously described^33^. To amplify the intergenic sequences 1 and 2 (ITS-1 and ITS-2) from fungal DNA, we performed nested PCR using the oligonucleotide primers and conditions described^36^. In addition, human mitochondrial DNA (mtDNA, D-loop region) was amplifed following the protocol described by Ghatak *et al*.^53^. RT-PCR analysis of the human 18S rRNA was performed using the primers 18SF 5′GTAACCCGTTGAACCCCATT 3′and 18SR 5′ CCATCCAATCGGTAGTAGCG 3′.

## Electronic supplementary material

Supplementary information
