# Supplementary material for: Human and Microbial Proteins From Corpora Amylacea of Alzheimer’s Disease
Source: Sci Rep. 2018 Jun 29;8:9880. doi: 10.1038/s41598-018-28231-1 (PMC6026157; doi:10.1038/s41598-018-28231-1)
Supplement: Supplementary file 1 — Supplementary information [file 41598_2018_28231_MOESM1_ESM.pdf]

HUMAN AND MICROBIAL PROTEINS FROM CORPORA AMYLACEA OF ALZHEIMER'S DISEASE

Diana Pisa[1], Ruth Alonso[1], Ana Isabel Marina[1], Alberto Rábano[2] and Luis Carrasco[1*]

[1]Centro de Biología Molecular "Severo Ochoa" (CSIC-UAM). c/Nicolás Cabrera, 1. Universidad Autónoma de Madrid. Cantoblanco. 28049 Madrid. Spain.

[2]Department of Neuropathology and Tissue Bank, Unidad de Investigación Proyecto Alzheimer, Fundación CIEN, Instituto de Salud Carlos III, Madrid. Spain.

[*]Corresponding author

Email address: lcarrasco@cbm.csic.es. Telephone number: +34 91 497 84 50

Supplementary Figure 1. Characterization of CA from AD1 and AD2.

A: Identification of CA in brain tissue before purification. Immunohistochemistry analysis of brain tissue from AD2. INCO sections of tissue were stained with PAS (left panel); incubated with a rabbit polyclonal anti-*C. albicans* antibody (green) used at 1:100 dilution, and a mouse monoclonal anti-human α-tubulin antibody (red) used at 1:50 dilution (middle panel); incubated with a mouse monoclonal antibody anti-peptidoglycan antibody (green) used at 1:20 dilution, and a rabbit polyclonal anti-*C. albicans* antibody (red) used at 1:100 dilution (right panel). DAPI staining of nuclei appears in blue. Scale bar: 10 μm. B: Comparison of proteins present in different fractions obtained during the purification protocol. Coomassie blue staining of the 4–12% SDS-PAGE gel containing the homogenate (H) and P7 fractions from AD1 and AD2. C: P7 fraction from AD1 and AD2 immunostained with a rabbit polyclonal anti-*C. albicans* antibody (green) used at 1:100 dilution. Three different fields of view are shown. Scale bar: 10 μm.

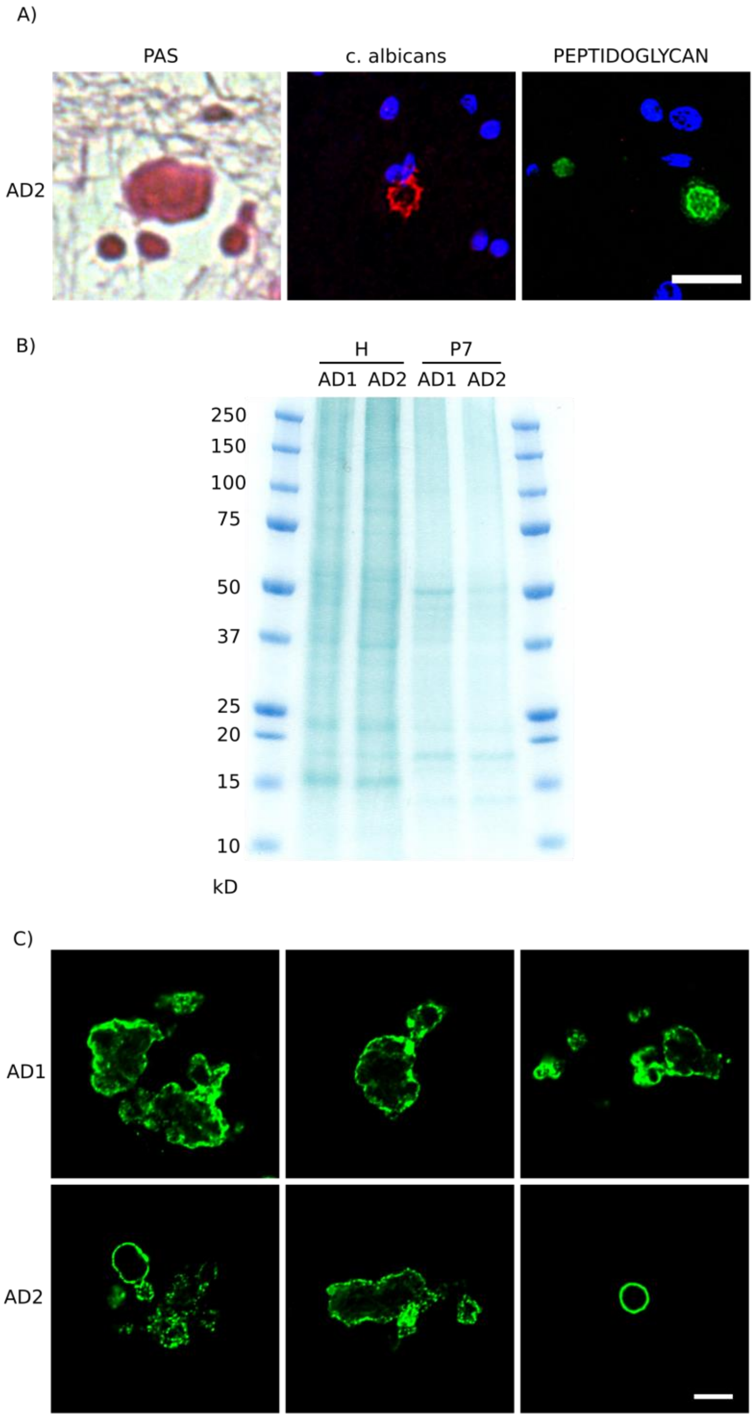

A)
PAS
c. albicans
PEPTIDOGLYCAN
AD2
B)
H
P7
AD1
AD2
AD1
AD2
250
150
100
75
50
37
25
20
15
10
kD
C)
AD1
AD2

Supplementary Figure 2. Characterization of proteins and nucleic acids in the homogenate and P7 fractions from patients AD1 and AD2.

A: Identification of human proteins by using specific antibodies. Western blot of proteins from homogenate (H) and P7 fractions from AD1 and AD2. Left panels: upper panel, goat polyclonal anti-eEF2 antibody used at 1:1,000 dilution as primary antibody and anti-goat peroxidase (POD) antibody used at 1:5,000 as secondary antibody, lower panel, control with anti-goat POD antibody used at 1:5,000; middle panels: upper panel, rabbit polyclonal eIF4GI antibody used at 1:50 dilution as primary antibody and anti-rabbit POD antibody used at 1:5,000 as secondary antibody, lower panel, control with anti-rabbit POD antibody used at 1:5,000; right panels: upper panel, mouse monoclonal anti-human α-tubulin antibody used at 1:50 dilution as primary antibody and anti-mouse POD antibody used at 1:5,000 as secondary antibody, lower panel, control with anti-mouse POD antibody used at 1:5000. B: Identification of mitochondrial DNA and 18S rRNA by PCR. Left panel: PCR analysis of mitochondrial DNA extracted from patients AD1 and AD2. The primers used to amplify mitochondrial DNA D-loop region were human mitochondrial (HMt)-F 5´CACCATTAGCACCCAAAGCT 3´ and HMt-R 5´ CTGTTAAAAGTGCATACCGCCA 3´. Right panel: RT-PCR analysis of human 18S rRNA from DNA extracted from the homogenate, P2 and P7 fractions from patients AD1 and AD2. C+: Positive control of DNA from HeLa cells; C-: Control of PCR without DNA. See also Supplementary info file.

A)

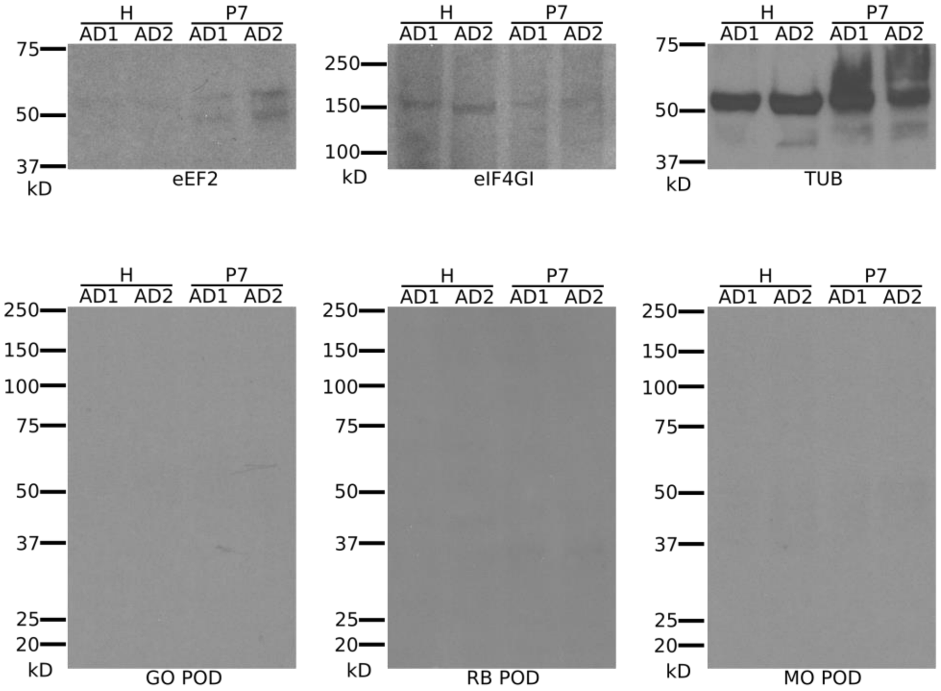
H
P7
AD1
AD2
75
50
37
kD
eEF2
250
150
100
eIF4GI
TUB
20
25
GO POD
RB POD
MO POD

B)

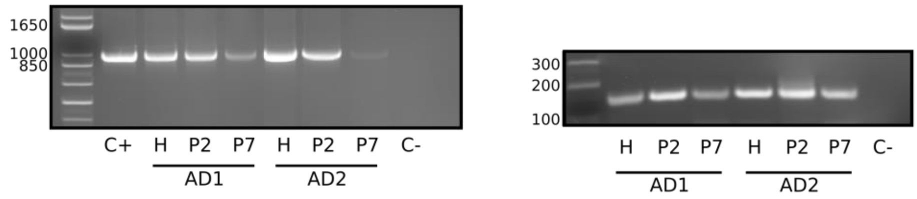
1650
1000
850
C+
H
P2
P7
C-
AD1
AD2
300
200
100

Supplementary Figure 3. Immunohistochemistry of CA in brain tissue from control subjects.

Identification of CA immunolabeled with anti-tubulin and anti-*C. albicans* antibodies. Entorhinal cortex/hippocampus (ERH) sections from control 1 and control 2 were immunostained with a rabbit polyclonal anti-*C. albicans* antibody (green) used at 1:100 dilution, and a mouse monoclonal anti-human α-tubulin antibody (red) used at 1:50 dilution. DAPI staining of nuclei appears in blue. Some CA are indicated (arrows). No immunolabeling is observed for fungal antigens (green) in CA. Scale bar: 20 μm.

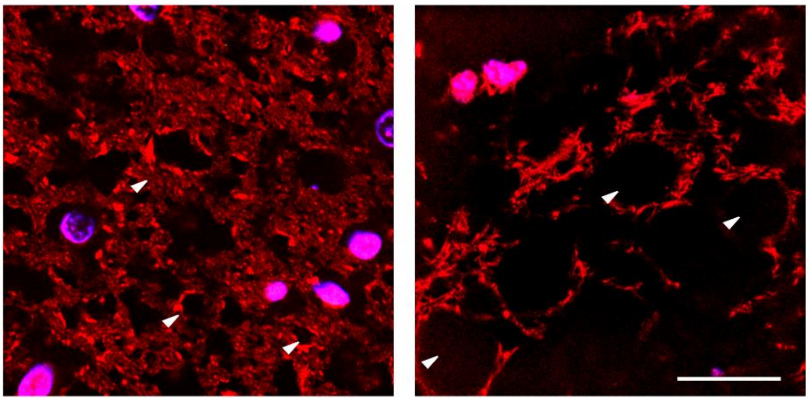

Supplementary Table I. Human peptides identified in P2 and P7 fractions in AD1.

Description AD1 P2	Peptides
Spectrin alpha chain, non-erythrocytic 1 OS=Homo sapiens GN=SPTAN1 PE=1 SV=3 - [SPTN1_HUMAN]	93
Isoform 7 of Plectin OS=Homo sapiens GN=PLEC - [PLEC_HUMAN]	81
Spectrin beta chain, non-erythrocytic 1 OS=Homo sapiens GN=SPTBN1 PE=1 SV=2 - [SPTB2_HUMAN]	50
Vesicle-fusing ATPase OS=Homo sapiens GN=NSF PE=1 SV=1 - [I3L0N3_HUMAN]	35
Isoform 4 of Dynamin-1 OS=Homo sapiens GN=DNM1 - [DYN1_HUMAN]	34
Isoform 2 of Annexin A6 OS=Homo sapiens GN=ANXA6 - [ANXA6_HUMAN]	33
Glial fibrillary acidic protein OS=Homo sapiens GN=GFAP PE=1 SV=1 - [GFAP_HUMAN]	33
Heat shock protein HSP 90-alpha OS=Homo sapiens GN=HSP90AA1 PE=1 SV=5 - [HS90A_HUMAN]	33
Isoform 3 of Dynamin-1 OS=Homo sapiens GN=DNM1 - [DYN1_HUMAN]	32
Vimentin OS=Homo sapiens GN=VIM PE=1 SV=4 - [VIME_HUMAN]	31
Isoform 4 of Hexokinase-1 OS=Homo sapiens GN=HK1 - [HXK1_HUMAN]	30
Neurofilament medium polypeptide OS=Homo sapiens GN=NEFM PE=1 SV=1 - [E7ESP9_HUMAN]	30
Heat shock protein HSP 90-beta OS=Homo sapiens GN=HSP90AB1 PE=1 SV=4 - [HS90B_HUMAN]	29
Isoform 2 of Alpha-actinin-1 OS=Homo sapiens GN=ACTN1 - [ACTN1_HUMAN]	28
Ankyrin-2 OS=Homo sapiens GN=ANK2 PE=1 SV=4 - [ANK2_HUMAN]	28
Isoform CNPI of 2',3'-cyclic-nucleotide 3'-phosphodiesterase OS=Homo sapiens GN=CNP - [CN37_HUMAN]	28
Neurofilament light polypeptide OS=Homo sapiens GN=NEFL PE=1 SV=3 - [NFL_HUMAN]	28
Isoform 2 of Contactin-1 OS=Homo sapiens GN=CNTN1 - [CNTN1_HUMAN]	25
Syntaxin-binding protein 1 OS=Homo sapiens GN=STXBP1 PE=1 SV=1 - [STXB1_HUMAN]	25
Isoform 2 of Syntaxin-binding protein 1 OS=Homo sapiens GN=STXBP1 - [STXB1_HUMAN]	25
Glutamate dehydrogenase 1, mitochondrial OS=Homo sapiens GN=GLUD1 PE=1 SV=2 - [DHE3_HUMAN]	23
Heat shock 70 kDa protein 12A OS=Homo sapiens GN=HSPA12A PE=1 SV=2 - [HS12A_HUMAN]	23
Heat shock cognate 71 kDa protein OS=Homo sapiens GN=HSPA8 PE=1 SV=1 - [HSP7C_HUMAN]	23
Alpha-internexin OS=Homo sapiens GN=INA PE=1 SV=2 - [AINX_HUMAN]	22
Calcium-binding mitochondrial carrier protein Aralar1 OS=Homo sapiens GN=SLC25A12 PE=1 SV=2 - [CMC1_HUMAN]	22
Isoform M1 of Pyruvate kinase PKM OS=Homo sapiens GN=PKM - [KPYM_HUMAN]	22
Isoform 2 of Tubulin alpha-1A chain OS=Homo sapiens GN=TUBA1A - [TBA1A_HUMAN]	22
ATP synthase subunit alpha, mitochondrial OS=Homo sapiens GN=ATP5A1 PE=1 SV=1 - [ATPA_HUMAN]	21
Microtubule-associated protein 1B OS=Homo sapiens GN=MAP1B PE=1 SV=2 - [MAP1B_HUMAN]	21
Tubulin beta-2A chain OS=Homo sapiens GN=TUBB2A PE=1 SV=1 - [TBB2A_HUMAN]	21
Tubulin beta-2B chain OS=Homo sapiens GN=TUBB2B PE=1 SV=1 - [TBB2B_HUMAN]	21

Tubulin beta-4B chain OS=Homo sapiens GN=TUBB4B PE=1 SV=1 - [TBB4B_HUMAN]	21
Tubulin beta chain OS=Homo sapiens GN=TUBB PE=1 SV=2 - [TBB5_HUMAN]	21
Aconitate hydratase, mitochondrial OS=Homo sapiens GN=ACO2 PE=1 SV=2 - [ACON_HUMAN]	20
60 kDa heat shock protein, mitochondrial OS=Homo sapiens GN=HSPD1 PE=1 SV=2 - [CH60_HUMAN]	20
NADH-ubiquinone oxidoreductase 75 kDa subunit, mitochondrial OS=Homo sapiens GN=NDUFS1 PE=1 SV=3 - [NDUS1_HUMAN]	20
Tubulin beta-4A chain OS=Homo sapiens GN=TUBB4A PE=1 SV=2 - [TBB4A_HUMAN]	20
Isoform 4 of Band 4.1-like protein 3 OS=Homo sapiens GN=EPB41L3 - [E41L3_HUMAN]	20
Amine oxidase [flavin-containing] B OS=Homo sapiens GN=MAOB PE=1 SV=3 - [AOFB_HUMAN]	19
Isoform B of AP-2 complex subunit alpha-1 OS=Homo sapiens GN=AP2A1 - [AP2A1_HUMAN]	19
Isoform C of Prelamin-A/C OS=Homo sapiens GN=LMNA - [LMNA_HUMAN]	19
Tubulin beta-3 chain OS=Homo sapiens GN=TUBB3 PE=1 SV=2 - [TBB3_HUMAN]	19
Isoform 2 of V-type proton ATPase catalytic subunit A OS=Homo sapiens GN=ATP6V1A - [VATA_HUMAN]	19
AP-2 complex subunit beta OS=Homo sapiens GN=AP2B1 PE=1 SV=1 - [A0A087X253_HUMAN]	19
Alpha-actinin-4 OS=Homo sapiens GN=ACTN4 PE=1 SV=2 - [ACTN4_HUMAN]	18
Alpha-enolase OS=Homo sapiens GN=ENO1 PE=1 SV=2 - [ENOA_HUMAN]	18
Stress-70 protein, mitochondrial OS=Homo sapiens GN=HSPA9 PE=1 SV=2 - [GRP75_HUMAN]	18
Isocitrate dehydrogenase [NADP], mitochondrial OS=Homo sapiens GN=IDH2 PE=1 SV=2 - [IDHP_HUMAN]	18
Isoform 2 of Tubulin alpha-4A chain OS=Homo sapiens GN=TUBA4A - [TBA4A_HUMAN]	18
Serum albumin OS=Homo sapiens GN=ALB PE=1 SV=2 - [ALBU_HUMAN]	17
Isoform 2 of Dynamin-3 OS=Homo sapiens GN=DNM3 - [DYN3_HUMAN]	17
Guanine nucleotide-binding protein G(o) subunit alpha OS=Homo sapiens GN=GNAO1 PE=1 SV=4 - [GNAO_HUMAN]	17
Heat shock-related 70 kDa protein 2 OS=Homo sapiens GN=HSPA2 PE=1 SV=1 - [HSP72_HUMAN]	17
Isoform 2 of Syntaxin-1B OS=Homo sapiens GN=STX1B - [STX1B_HUMAN]	17
Synaptotagmin I, isoform CRA_b OS=Homo sapiens GN=SYT1 PE=1 SV=1 - [J3KQA0_HUMAN]	17
Isoform 2 of Ubiquitin-like modifier-activating enzyme 1 OS=Homo sapiens GN=UBA1 - [UBA1_HUMAN]	17
14-3-3 protein epsilon OS=Homo sapiens GN=YWHAE PE=1 SV=1 - [1433E_HUMAN]	16
ATP synthase subunit beta, mitochondrial OS=Homo sapiens GN=ATP5B PE=1 SV=3 - [ATPB_HUMAN]	16
Isoform 2 of Clathrin heavy chain 1 OS=Homo sapiens GN=CLTC - [CLH1_HUMAN]	16
Isoform 2 of Dihydropyrimidinase-related protein 2 OS=Homo sapiens GN=DPYSL2 - [DPYL2_HUMAN]	16
EH domain-containing protein 3 OS=Homo sapiens GN=EHD3 PE=1 SV=2 - [EHD3_HUMAN]	16
ATP-dependent 6-phosphofructokinase, platelet type OS=Homo sapiens GN=PFKP PE=1 SV=2 - [PFKAP_HUMAN]	16
Tubulin alpha-8 chain OS=Homo sapiens GN=TUBA8 PE=1 SV=1 - [TBA8_HUMAN]	16
V-type proton ATPase subunit B, brain isoform OS=Homo sapiens GN=ATP6V1B2 PE=1 SV=3 - [VATB2_HUMAN]	16
Aspartate aminotransferase, mitochondrial OS=Homo sapiens GN=GOT2 PE=1 SV=3 - [AATM_HUMAN]	15
Actin, aortic smooth muscle OS=Homo sapiens GN=ACTA2 PE=1 SV=1 - [ACTA_HUMAN]	15
Actin, cytoplasmic 1 OS=Homo sapiens GN=ACTB PE=1 SV=1 - [ACTB_HUMAN]	15
Annexin A5 OS=Homo sapiens GN=ANXA5 PE=1 SV=2 - [ANXA5_HUMAN]	15
AP-2 complex subunit alpha-2 OS=Homo sapiens GN=AP2A2 PE=1 SV=2 - [AP2A2_HUMAN]	15
Trifunctional enzyme subunit alpha, mitochondrial OS=Homo sapiens GN=HADHA PE=1 SV=2 - [ECHA_HUMAN]	15
Keratin, type II cytoskeletal 1 OS=Homo sapiens GN=KRT1 PE=1 SV=6 - [K2C1_HUMAN]	15
Isoform 3 of Microtubule-associated protein 2 OS=Homo sapiens GN=MAP2 - [MTAP2_HUMAN]	15
Isoform 10 of Neurofascin OS=Homo sapiens GN=NFASC - [NFASC_HUMAN]	15
Protein disulfide-isomerase A3 OS=Homo sapiens GN=PDIA3 PE=1 SV=4 - [PDIA3_HUMAN]	15
T-complex protein 1 subunit theta OS=Homo sapiens GN=CCT8 PE=1 SV=4 - [TCPQ_HUMAN]	15
MICOS complex subunit MIC60 OS=Homo sapiens GN=IMMT PE=1 SV=2 - [B9A067_HUMAN]	15
Aldehyde dehydrogenase, mitochondrial OS=Homo sapiens GN=ALDH2 PE=1 SV=2 - [ALDH2_HUMAN]	14
Rab GDP dissociation inhibitor alpha OS=Homo sapiens GN=GDI1 PE=1 SV=2 - [GDIA_HUMAN]	14
Heat shock 70 kDa protein 1A/1B OS=Homo sapiens GN=HSPA1A PE=1 SV=5 - [HSP71_HUMAN]	14
Neural cell adhesion molecule 1 OS=Homo sapiens GN=NCAM1 PE=1 SV=3 - [NCAM1_HUMAN]	14
NADH dehydrogenase [ubiquinone] 1 alpha subcomplex subunit 9, mitochondrial OS=Homo sapiens GN=NDUFA9 PE=1 SV=2 - [NDUA9_HUMAN]	14
Glycogen phosphorylase, brain form OS=Homo sapiens GN=PYGB PE=1 SV=5 - [PYGB_HUMAN]	14
Septin-7 OS=Homo sapiens GN=SEPT7 PE=1 SV=2 - [SEPT7_HUMAN]	14
Isoform Short of Ubiquitin carboxyl-terminal hydrolase 5 OS=Homo sapiens GN=USP5 - [UBP5_HUMAN]	14

Sodium/potassium-transporting ATPase subunit alpha-3 OS=Homo sapiens GN=ATP1A3 PE=1 SV=1 - [M0R116_HUMAN]	14
Band 4.1-like protein 1 OS=Homo sapiens GN=EPB41L1 PE=4 SV=1 - [A0A0C4DH22_HUMAN]	14
Serine/threonine-protein phosphatase 2A 65 kDa regulatory subunit A alpha isoform OS=Homo sapiens GN=PPP2R1A PE=1 SV=4 - [2AAA_HUMAN]	13
Annexin A2 OS=Homo sapiens GN=ANXA2 PE=1 SV=2 - [ANXA2_HUMAN]	13
Isoform 3 of Sodium/potassium-transporting ATPase subunit alpha-1 OS=Homo sapiens GN=ATP1A1 - [AT1A1_HUMAN]	13
Calnexin OS=Homo sapiens GN=CANX PE=1 SV=2 - [CALX_HUMAN]	13
Isoform 3 of Catenin alpha-2 OS=Homo sapiens GN=CTNNA2 - [CTNA2_HUMAN]	13
Elongation factor Tu, mitochondrial OS=Homo sapiens GN=TUFM PE=1 SV=2 - [EFTU_HUMAN]	13
Gamma-enolase OS=Homo sapiens GN=ENO2 PE=1 SV=3 - [ENOG_HUMAN]	13
Endoplasmin OS=Homo sapiens GN=HSP90B1 PE=1 SV=1 - [ENPL_HUMAN]	13
Glycerol-3-phosphate dehydrogenase, mitochondrial OS=Homo sapiens GN=GPD2 PE=1 SV=3 - [GPDM_HUMAN]	13
78 kDa glucose-regulated protein OS=Homo sapiens GN=HSPA5 PE=1 SV=2 - [GRP78_HUMAN]	13
Calcium/calmodulin-dependent protein kinase type II subunit alpha OS=Homo sapiens GN=CAMK2A PE=1 SV=2 - [KCC2A_HUMAN]	13
Malate dehydrogenase, mitochondrial OS=Homo sapiens GN=MDH2 PE=1 SV=3 - [MDHM_HUMAN]	13
Cytochrome b-c1 complex subunit 2, mitochondrial OS=Homo sapiens GN=UQCRC2 PE=1 SV=3 - [QCR2_HUMAN]	13
Tubulin beta-6 chain OS=Homo sapiens GN=TUBB6 PE=1 SV=1 - [TBB6_HUMAN]	13
T-complex protein 1 subunit beta OS=Homo sapiens GN=CCT2 PE=1 SV=4 - [TCPB_HUMAN]	13
Visinin-like protein 1 OS=Homo sapiens GN=VSNL1 PE=1 SV=2 - [VISL1_HUMAN]	13
Isoform 2 of Trifunctional enzyme subunit beta, mitochondrial OS=Homo sapiens GN=HADHB - [ECHB_HUMAN]	13
Sodium/potassium-transporting ATPase subunit alpha-2 OS=Homo sapiens GN=ATP1A2 PE=1 SV=1 - [B1AKY9_HUMAN]	13
14-3-3 protein eta OS=Homo sapiens GN=YWHAH PE=1 SV=4 - [1433F_HUMAN]	12
14-3-3 protein zeta/delta OS=Homo sapiens GN=YWHAZ PE=1 SV=1 - [1433Z_HUMAN]	12
Alpha-actinin-2 OS=Homo sapiens GN=ACTN2 PE=1 SV=1 - [ACTN2_HUMAN]	12
Fructose-bisphosphate aldolase A OS=Homo sapiens GN=ALDOA PE=1 SV=2 - [ALDOA_HUMAN]	12
Glucose-6-phosphate isomerase OS=Homo sapiens GN=GPI PE=1 SV=4 - [G6PI_HUMAN]	12
4-aminobutyrate aminotransferase, mitochondrial OS=Homo sapiens GN=ABAT PE=1 SV=3 - [GABT_HUMAN]	12
Isoform 2 of Gelsolin OS=Homo sapiens GN=GSN - [GELS_HUMAN]	12
Importin subunit beta-1 OS=Homo sapiens GN=KPNB1 PE=1 SV=2 - [IMB1_HUMAN]	12
Keratin, type I cytoskeletal 10 OS=Homo sapiens GN=KRT10 PE=1 SV=6 - [K1C10_HUMAN]	12
L-lactate dehydrogenase B chain OS=Homo sapiens GN=LDHB PE=1 SV=2 - [LDHB_HUMAN]	12
Lamin-B2 OS=Homo sapiens GN=LMNB2 PE=1 SV=3 - [LMNB2_HUMAN]	12
Isoform 2 of Pyruvate dehydrogenase E1 component subunit beta, mitochondrial OS=Homo sapiens GN=PDHB - [ODPB_HUMAN]	12
Dynamin-like 120 kDa protein, mitochondrial OS=Homo sapiens GN=OPA1 PE=1 SV=3 - [OPA1_HUMAN]	12
Isoform A2 of Heterogeneous nuclear ribonucleoproteins A2/B1 OS=Homo sapiens GN=HNRNPA2B1 - [ROA2_HUMAN]	12
Synaptosomal-associated protein 25 OS=Homo sapiens GN=SNAP25 PE=1 SV=1 - [SNP25_HUMAN]	12
Syntaxin-1A OS=Homo sapiens GN=STX1A PE=1 SV=1 - [STX1A_HUMAN]	12
Isoform 2 of Succinyl-CoA ligase [ADP-forming] subunit beta, mitochondrial OS=Homo sapiens GN=SUCLA2 - [SUCB1_HUMAN]	12
Mitochondrial import receptor subunit TOM70 OS=Homo sapiens GN=TOMM70A PE=1 SV=1 - [TOM70_HUMAN]	12
Isoform 2 of Triosephosphate isomerase OS=Homo sapiens GN=TPI1 - [TPIS_HUMAN]	12
Citrate synthase, mitochondrial OS=Homo sapiens GN=CS PE=1 SV=1 - [B4DJV2_HUMAN]	12
Fructose-bisphosphate aldolase OS=Homo sapiens GN=ALDOC PE=1 SV=1 - [A8MVZ9_HUMAN]	12
14-3-3 protein gamma OS=Homo sapiens GN=YWHAG PE=1 SV=2 - [1433G_HUMAN]	11
Isoform 4 of AP-1 complex subunit beta-1 OS=Homo sapiens GN=AP1B1 - [AP1B1_HUMAN]	11
Isoform 3 of Disks large homolog 4 OS=Homo sapiens GN=DLG4 - [DLG4_HUMAN]	11
Cytoplasmic dynein 1 heavy chain 1 OS=Homo sapiens GN=DYNC1H1 PE=1 SV=5 - [DYHC1_HUMAN]	11
Isoform 3 of Calcium/calmodulin-dependent protein kinase type II subunit beta OS=Homo sapiens GN=CAMK2B - [KCC2B_HUMAN]	11
Creatine kinase B-type OS=Homo sapiens GN=CKB PE=1 SV=1 - [KCRB_HUMAN]	11
L-lactate dehydrogenase A chain OS=Homo sapiens GN=LDHA PE=1 SV=2 - [LDHA_HUMAN]	11
Microtubule-associated protein 1A OS=Homo sapiens GN=MAP1A PE=1 SV=6 - [MAP1A_HUMAN]	11
Isoform 3 of Pyruvate dehydrogenase E1 component subunit alpha, somatic form, mitochondrial OS=Homo sapiens GN=PDHA1 - [ODPA_HUMAN]	11
2-oxoglutarate dehydrogenase-like, mitochondrial OS=Homo sapiens GN=OGDHL PE=1 SV=3 - [OGDHL_HUMAN]	11
Protein disulfide-isomerase OS=Homo sapiens GN=P4HB PE=1 SV=3 - [PDIA1_HUMAN]	11
Cytochrome b-c1 complex subunit 1, mitochondrial OS=Homo sapiens GN=UQCRC1 PE=1 SV=3 - [QCR1_HUMAN]	11

Protein RUFY3 OS=Homo sapiens GN=RUFY3 PE=1 SV=1 - [RUFY3_HUMAN]	11
Beta-soluble NSF attachment protein OS=Homo sapiens GN=NAPB PE=1 SV=2 - [SNAB_HUMAN]	11
Isoform 2 of Synaptosomal-associated protein 25 OS=Homo sapiens GN=SNAP25 - [SNP25_HUMAN]	11
V-type proton ATPase subunit E 1 OS=Homo sapiens GN=ATP6V1E1 PE=1 SV=1 - [VATE1_HUMAN]	11
Isoform 2 of Phosphoglycerate kinase 1 OS=Homo sapiens GN=PGK1 - [PGK1_HUMAN]	11
Catenin beta-1 OS=Homo sapiens GN=CTNNB1 PE=1 SV=1 - [B4DGU4_HUMAN]	11
Calcium/calmodulin-dependent protein kinase (CaM kinase) II gamma, isoform CRA_n OS=Homo sapiens GN=CAMK2G PE=1 SV=1 - [Q5SWX3_HUMAN]	11
Isoform 2 of Methylmalonate-semialdehyde dehydrogenase [acylating], mitochondrial OS=Homo sapiens GN=ALDH6A1 - [MMSA_HUMAN]	11
Neural cell adhesion molecule 1 OS=Homo sapiens GN=NCAM1 PE=1 SV=2 - [H7BYX6_HUMAN]	11
Isoform Short of 14-3-3 protein beta/alpha OS=Homo sapiens GN=YWHAB - [1433B_HUMAN]	10
Isoform 2 of 4F2 cell-surface antigen heavy chain OS=Homo sapiens GN=SLC3A2 - [4F2_HUMAN]	10
Isoform 2 of Alpha-aminoadipic semialdehyde dehydrogenase OS=Homo sapiens GN=ALDH7A1 - [AL7A1_HUMAN]	10
ATP synthase F(0) complex subunit B1, mitochondrial OS=Homo sapiens GN=ATP5F1 PE=1 SV=2 - [AT5F1_HUMAN]	10
Isoform 2 of Atlastin-1 OS=Homo sapiens GN=ATL1 - [ATLA1_HUMAN]	10
Isoform 4 of Voltage-dependent calcium channel subunit alpha-2/delta-1 OS=Homo sapiens GN=CACNA2D1 - [CA2D1_HUMAN]	10
Carbonyl reductase [NADPH] 1 OS=Homo sapiens GN=CBR1 PE=1 SV=3 - [CBR1_HUMAN]	10
Cytosolic non-specific dipeptidase OS=Homo sapiens GN=CNDP2 PE=1 SV=2 - [CNDP2_HUMAN]	10
Copine-6 OS=Homo sapiens GN=CPNE6 PE=1 SV=3 - [CPNE6_HUMAN]	10
Dihydropyrimidinase-related protein 3 OS=Homo sapiens GN=DPYSL3 PE=1 SV=1 - [DPYL3_HUMAN]	10
Guanine nucleotide-binding protein G(i) subunit alpha-2 OS=Homo sapiens GN=GNAI2 PE=1 SV=3 - [GNAI2_HUMAN]	10
Hemoglobin subunit beta OS=Homo sapiens GN=HBB PE=1 SV=2 - [HBB_HUMAN]	10
Immunoglobulin superfamily member 8 OS=Homo sapiens GN=IGSF8 PE=1 SV=1 - [IGSF8_HUMAN]	10
Isoform Delta 12 of Calcium/calmodulin-dependent protein kinase type II subunit delta OS=Homo sapiens GN=CAMK2D - [KCC2D_HUMAN]	10
Malate dehydrogenase, cytoplasmic OS=Homo sapiens GN=MDH1 PE=1 SV=4 - [MDHC_HUMAN]	10
Isoform 2 of Neurochondrin OS=Homo sapiens GN=NCDN - [NCDN_HUMAN]	10
NADH dehydrogenase [ubiquinone] iron-sulfur protein 3, mitochondrial OS=Homo sapiens GN=NDUFS3 PE=1 SV=1 - [NDUS3_HUMAN]	10
Isoform 2 of Neurofilament heavy polypeptide OS=Homo sapiens GN=NEFH - [NFH_HUMAN]	10
T-complex protein 1 subunit delta OS=Homo sapiens GN=CCT4 PE=1 SV=4 - [TCPD_HUMAN]	10
Tenascin-R OS=Homo sapiens GN=TNR PE=1 SV=3 - [TENR_HUMAN]	10
Acetyl-CoA acetyltransferase, mitochondrial OS=Homo sapiens GN=ACAT1 PE=1 SV=1 - [THIL_HUMAN]	10
Voltage-dependent anion-selective channel protein 1 OS=Homo sapiens GN=VDAC1 PE=1 SV=2 - [VDAC1_HUMAN]	10
Dipeptidyl aminopeptidase-like protein 6 OS=Homo sapiens GN=DPP6 PE=1 SV=1 - [E9PF59_HUMAN]	10
Alpha-crystallin B chain (Fragment) OS=Homo sapiens GN=CRYAB PE=1 SV=1 - [E9PR44_HUMAN]	10
Prohibitin-2 OS=Homo sapiens GN=PHB2 PE=1 SV=1 - [F5GY37_HUMAN]	10
Myelin-oligodendrocyte glycoprotein OS=Homo sapiens GN=MOG PE=1 SV=1 - [C9JTE0_HUMAN]	10
Myelin basic protein OS=Homo sapiens GN=MBP PE=1 SV=1 - [J3QL64_HUMAN]	10
Peroxiredoxin-1 (Fragment) OS=Homo sapiens GN=PRDX1 PE=1 SV=1 - [A0A0A0MSI0_HUMAN]	10
14-3-3 protein theta OS=Homo sapiens GN=YWHAQ PE=1 SV=1 - [1433T_HUMAN]	9
Isoform 2 of Alpha-adducin OS=Homo sapiens GN=ADD1 - [ADDA_HUMAN]	9
ADP/ATP translocase 1 OS=Homo sapiens GN=SLC25A4 PE=1 SV=4 - [ADT1_HUMAN]	9
Cytosolic 10-formyltetrahydrofolate dehydrogenase OS=Homo sapiens GN=ALDH1L1 PE=1 SV=2 - [AL1L1_HUMAN]	9
ATP synthase subunit gamma, mitochondrial OS=Homo sapiens GN=ATP5C1 PE=1 SV=1 - [ATPG_HUMAN]	9
Brain acid soluble protein 1 OS=Homo sapiens GN=BASP1 PE=1 SV=2 - [BASP1_HUMAN]	9
C-1-tetrahydrofolate synthase, cytoplasmic OS=Homo sapiens GN=MTHFD1 PE=1 SV=3 - [C1TC_HUMAN]	9
Cullin-associated NEDD8-dissociated protein 1 OS=Homo sapiens GN=CAND1 PE=1 SV=2 - [CAND1_HUMAN]	9
Cathepsin D OS=Homo sapiens GN=CTSD PE=1 SV=1 - [CATD_HUMAN]	9
Isoform 1 of Serine/threonine-protein kinase DCLK1 OS=Homo sapiens GN=DCLK1 - [DCLK1_HUMAN]	9
Desmin OS=Homo sapiens GN=DES PE=1 SV=3 - [DESM_HUMAN]	9
Glutaminase kidney isoform, mitochondrial OS=Homo sapiens GN=GLS PE=1 SV=1 - [GLSK_HUMAN]	9
Guanine nucleotide-binding protein G(i) subunit alpha-1 OS=Homo sapiens GN=GNAI1 PE=1 SV=2 - [GNAI1_HUMAN]	9
Isoform 3 of Guanine nucleotide-binding protein G(s) subunit alpha isoforms short OS=Homo sapiens GN=GNAS - [GNAS2_HUMAN]	9
Guanine deaminase OS=Homo sapiens GN=GDA PE=1 SV=1 - [GUAD_HUMAN]	9
Heat shock protein beta-1 OS=Homo sapiens GN=HSPB1 PE=1 SV=2 - [HSPB1_HUMAN]	9

Isocitrate dehydrogenase [NAD] subunit beta, mitochondrial OS=Homo sapiens GN=IDH3B PE=1 SV=2 - [IDH3B_HUMAN]	9
Eukaryotic initiation factor 4A-II OS=Homo sapiens GN=EIF4A2 PE=1 SV=2 - [IF4A2_HUMAN]	9
Keratin, type I cytoskeletal 9 OS=Homo sapiens GN=KRT9 PE=1 SV=3 - [K1C9_HUMAN]	9
Keratin, type II cytoskeletal 2 epidermal OS=Homo sapiens GN=KRT2 PE=1 SV=2 - [K22E_HUMAN]	9
Isoform 3 of Adenylate kinase isoenzyme 5 OS=Homo sapiens GN=AK5 - [KAD5_HUMAN]	9
Isoform 6 of Myelin basic protein OS=Homo sapiens GN=MBP - [MBP_HUMAN]	9
Dual specificity mitogen-activated protein kinase kinase 1 OS=Homo sapiens GN=MAP2K1 PE=1 SV=2 - [MP2K1_HUMAN]	9
Isoform 3 of Nucleoside diphosphate kinase B OS=Homo sapiens GN=NME2 - [NDKB_HUMAN]	9
Isoform 2 of NADH dehydrogenase [ubiquinone] iron-sulfur protein 2, mitochondrial OS=Homo sapiens GN=NDUFS2 - [NDUS2_HUMAN]	9
Isoform 2 of NADH dehydrogenase [ubiquinone] flavoprotein 1, mitochondrial OS=Homo sapiens GN=NDUFV1 - [NDUV1_HUMAN]	9
Isoform 2 of 2-oxoglutarate dehydrogenase, mitochondrial OS=Homo sapiens GN=OGDH - [ODO1_HUMAN]	9
Phosphatidylethanolamine-binding protein 1 OS=Homo sapiens GN=PEBP1 PE=1 SV=3 - [PEBP1_HUMAN]	9
Prohibitin OS=Homo sapiens GN=PHB PE=1 SV=1 - [PHB_HUMAN]	9
Phosphatidylinositol 5-phosphate 4-kinase type-2 beta OS=Homo sapiens GN=PIP4K2B PE=1 SV=1 - [PI42B_HUMAN]	9
Succinyl-CoA:3-ketoacid coenzyme A transferase 1, mitochondrial OS=Homo sapiens GN=OXCT1 PE=1 SV=1 - [SCOT1_HUMAN]	9
Isoform 2 of Spectrin beta chain, non-erythrocytic 2 OS=Homo sapiens GN=SPTBN2 - [SPTN2_HUMAN]	9
Isoform IB of Synapsin-1 OS=Homo sapiens GN=SYN1 - [SYN1_HUMAN]	9
T-complex protein 1 subunit zeta OS=Homo sapiens GN=CCT6A PE=1 SV=3 - [TCPZ_HUMAN]	9
Ubiquitin carboxyl-terminal hydrolase isozyme L1 OS=Homo sapiens GN=UCHL1 PE=1 SV=2 - [UCHL1_HUMAN]	9
V-type proton ATPase subunit C 1 OS=Homo sapiens GN=ATP6V1C1 PE=1 SV=4 - [VATC1_HUMAN]	9
Vacuolar protein sorting-associated protein 35 OS=Homo sapiens GN=VPS35 PE=1 SV=2 - [VPS35_HUMAN]	9
T-complex protein 1 subunit gamma OS=Homo sapiens GN=CCT3 PE=1 SV=1 - [B4DUR8_HUMAN]	9
Isoform 2 of Phosphatidylinositol 5-phosphate 4-kinase type-2 alpha OS=Homo sapiens GN=PIP4K2A - [PI42A_HUMAN]	9
Septin 11, isoform CRA_b OS=Homo sapiens GN=SEPT11 PE=1 SV=1 - [D6RGI3_HUMAN]	9
Puromycin-sensitive aminopeptidase OS=Homo sapiens GN=NPEPPS PE=1 SV=1 - [E9PLK3_HUMAN]	9
Cytoplasmic FMR1-interacting protein 2 OS=Homo sapiens GN=CYFIP2 PE=1 SV=1 - [E7EVJ5_HUMAN]	9
Dihydropyrimidinase-related protein 1 OS=Homo sapiens GN=CRMP1 PE=1 SV=1 - [E9PD68_HUMAN]	9
Endophilin-B2 OS=Homo sapiens GN=SH3GLB2 PE=1 SV=1 - [B7ZC39_HUMAN]	9
Unconventional myosin-Va OS=Homo sapiens GN=MYO5A PE=1 SV=1 - [A0A087WY00_HUMAN]	9
Synapsin-2 OS=Homo sapiens GN=SYN2 PE=1 SV=1 - [A0A087X2E3_HUMAN]	9
Aspartate aminotransferase, cytoplasmic OS=Homo sapiens GN=GOT1 PE=1 SV=3 - [AATC_HUMAN]	8
Alpha-actinin-3 OS=Homo sapiens GN=ACTN3 PE=1 SV=2 - [ACTN3_HUMAN]	8
Annexin A1 OS=Homo sapiens GN=ANXA1 PE=1 SV=2 - [ANXA1_HUMAN]	8
Isoform E of Plasma membrane calcium-transporting ATPase 1 OS=Homo sapiens GN=ATP2B1 - [AT2B1_HUMAN]	8
ATP synthase subunit d, mitochondrial OS=Homo sapiens GN=ATP5H PE=1 SV=3 - [ATP5H_HUMAN]	8
F-actin-capping protein subunit beta OS=Homo sapiens GN=CAPZB PE=1 SV=4 - [CAPZB_HUMAN]	8
Cytochrome c oxidase subunit 4 isoform 1, mitochondrial OS=Homo sapiens GN=COX4I1 PE=1 SV=1 - [COX41_HUMAN]	8
Ferritin heavy chain OS=Homo sapiens GN=FTH1 PE=1 SV=2 - [FRIH_HUMAN]	8
Glyceraldehyde-3-phosphate dehydrogenase OS=Homo sapiens GN=GAPDH PE=1 SV=3 - [G3P_HUMAN]	8
Isoform 6 of Rap1 GTPase-GDP dissociation stimulator 1 OS=Homo sapiens GN=RAP1GDS1 - [GDS1_HUMAN]	8
Glutamine synthetase OS=Homo sapiens GN=GLUL PE=1 SV=4 - [GLNA_HUMAN]	8
Histone H4 OS=Homo sapiens GN=HIST1H4A PE=1 SV=2 - [H4_HUMAN]	8
Isocitrate dehydrogenase [NAD] subunit alpha, mitochondrial OS=Homo sapiens GN=IDH3A PE=1 SV=1 - [IDH3A_HUMAN]	8
Isoform 2 of NADH-cytochrome b5 reductase 3 OS=Homo sapiens GN=CYB5R3 - [NB5R3_HUMAN]	8
Nucleoside diphosphate kinase A OS=Homo sapiens GN=NME1 PE=1 SV=1 - [NDKA_HUMAN]	8
Isoform 5 of Neuroplastin OS=Homo sapiens GN=NPTN - [NPTN_HUMAN]	8
Peptidyl-prolyl cis-trans isomerase A OS=Homo sapiens GN=PPIA PE=1 SV=2 - [PPIA_HUMAN]	8
Ras-related C3 botulinum toxin substrate 1 OS=Homo sapiens GN=RAC1 PE=1 SV=1 - [RAC1_HUMAN]	8
Radixin OS=Homo sapiens GN=RDX PE=1 SV=1 - [RADI_HUMAN]	8
Ras-related protein Rab-11B OS=Homo sapiens GN=RAB11B PE=1 SV=4 - [RB11B_HUMAN]	8
Isoform 2 of Heterogeneous nuclear ribonucleoprotein A1 OS=Homo sapiens GN=HNRNPA1 - [ROA1_HUMAN]	8
Sorting and assembly machinery component 50 homolog OS=Homo sapiens GN=SAMM50 PE=1 SV=3 - [SAM50_HUMAN]	8
Septin-5 OS=Homo sapiens GN=SEPT5 PE=1 SV=1 - [SEPT5_HUMAN]	8

Sideroflexin-1 OS=Homo sapiens GN=SFXN1 PE=1 SV=4 - [SFXN1_HUMAN]	8
Tyrosine-protein phosphatase non-receptor type substrate 1 OS=Homo sapiens GN=SIRPA PE=1 SV=2 - [SHPS1_HUMAN]	8
Gamma-soluble NSF attachment protein OS=Homo sapiens GN=NAPG PE=1 SV=1 - [SNAG_HUMAN]	8
Succinate-semialdehyde dehydrogenase, mitochondrial OS=Homo sapiens GN=ALDH5A1 PE=1 SV=2 - [SSDH_HUMAN]	8
Phenylalanine--tRNA ligase beta subunit OS=Homo sapiens GN=FARSB PE=1 SV=3 - [SYFB_HUMAN]	8
Isoform Tau-A of Microtubule-associated protein tau OS=Homo sapiens GN=MAPT - [TAU_HUMAN]	8
Isoform Tau-D of Microtubule-associated protein tau OS=Homo sapiens GN=MAPT - [TAU_HUMAN]	8
T-complex protein 1 subunit epsilon OS=Homo sapiens GN=CCT5 PE=1 SV=1 - [TCPE_HUMAN]	8
Isoform 2 of V-type proton ATPase subunit H OS=Homo sapiens GN=ATP6V1H - [VATH_HUMAN]	8
Voltage-dependent anion-selective channel protein 3 OS=Homo sapiens GN=VDAC3 PE=1 SV=1 - [VDAC3_HUMAN]	8
Isoform 2 of V-type proton ATPase 116 kDa subunit a isoform 1 OS=Homo sapiens GN=ATP6V0A1 - [VPP1_HUMAN]	8
VCAN protein OS=Homo sapiens GN=VCAN PE=1 SV=1 - [Q86W61_HUMAN]	8
Neutral alpha-glucosidase AB OS=Homo sapiens GN=GANAB PE=1 SV=1 - [F5H6X6_HUMAN]	8
Tropomyosin alpha-1 chain OS=Homo sapiens GN=TPM1 PE=1 SV=2 - [F5H7S3_HUMAN]	8
Ubiquitin thioesterase OTUB1 OS=Homo sapiens GN=OTUB1 PE=1 SV=1 - [F5GYN4_HUMAN]	8
Lon protease homolog, mitochondrial OS=Homo sapiens GN=LONP1 PE=1 SV=1 - [K7EJE8_HUMAN]	8
Alpha-soluble NSF attachment protein OS=Homo sapiens GN=NAPA PE=1 SV=1 - [M0R0Y2_HUMAN]	8
Mitochondrial 2-oxoglutarate/malate carrier protein (Fragment) OS=Homo sapiens GN=SLC25A11 PE=1 SV=1 - [I3L1P8_HUMAN]	8
Sideroflexin-3 OS=Homo sapiens GN=SFXN3 PE=1 SV=1 - [A0A0A0MS41_HUMAN]	8
Glutathione S-transferase Mu 3 OS=Homo sapiens GN=GSTM3 PE=1 SV=1 - [A0A0A0MTN3_HUMAN]	8
NAD-dependent protein deacetylase sirtuin-2 OS=Homo sapiens GN=SIRT2 PE=1 SV=1 - [A0A0A0MRF5_HUMAN]	8
Voltage-dependent anion-selective channel protein 2 (Fragment) OS=Homo sapiens GN=VDAC2 PE=1 SV=1 - [A0A0A0MR02_HUMAN]	8
Septin-8 OS=Homo sapiens GN=SEPT8 PE=1 SV=1 - [A0A087X142_HUMAN]	8
ADP/ATP translocase 2 OS=Homo sapiens GN=SLC25A5 PE=1 SV=7 - [ADT2_HUMAN]	7
ADP/ATP translocase 3 OS=Homo sapiens GN=SLC25A6 PE=1 SV=4 - [ADT3_HUMAN]	7
Isoform 2 of Annexin A7 OS=Homo sapiens GN=ANXA7 - [ANXA7_HUMAN]	7
Amine oxidase [flavin-containing] A OS=Homo sapiens GN=MAOA PE=1 SV=1 - [AOFA_HUMAN]	7
Apolipoprotein E OS=Homo sapiens GN=APOE PE=1 SV=1 - [APOE_HUMAN]	7
Isoform ZK of Plasma membrane calcium-transporting ATPase 4 OS=Homo sapiens GN=ATP2B4 - [AT2B4_HUMAN]	7
ATP synthase subunit O, mitochondrial OS=Homo sapiens GN=ATP5O PE=1 SV=1 - [ATPO_HUMAN]	7
Carbonic anhydrase 4 OS=Homo sapiens GN=CA4 PE=1 SV=2 - [CAH4_HUMAN]	7
Copine-5 OS=Homo sapiens GN=CPNE5 PE=1 SV=2 - [CPNE5_HUMAN]	7
Cytochrome c1, heme protein, mitochondrial OS=Homo sapiens GN=CYC1 PE=1 SV=3 - [CY1_HUMAN]	7
N(G),N(G)-dimethylarginine dimethylaminohydrolase 1 OS=Homo sapiens GN=DDAH1 PE=1 SV=3 - [DDAH1_HUMAN]	7
Very-long-chain 3-oxoacyl-CoA reductase OS=Homo sapiens GN=HSD17B12 PE=1 SV=2 - [DHB12_HUMAN]	7
Succinate dehydrogenase [ubiquinone] iron-sulfur subunit, mitochondrial OS=Homo sapiens GN=SDHB PE=1 SV=3 - [SDHB_HUMAN]	7
Isoform 3 of Dynamin-1-like protein OS=Homo sapiens GN=DNM1L - [DNM1L_HUMAN]	7
Elongation factor 1-alpha 1 OS=Homo sapiens GN=EEF1A1 PE=1 SV=1 - [EF1A1_HUMAN]	7
Elongation factor 1-alpha 2 OS=Homo sapiens GN=EEF1A2 PE=1 SV=1 - [EF1A2_HUMAN]	7
Ferritin light chain OS=Homo sapiens GN=FTL PE=1 SV=2 - [FRIL_HUMAN]	7
Ganglioside-induced differentiation-associated protein 1 OS=Homo sapiens GN=GDAP1 PE=1 SV=3 - [GDAP1_HUMAN]	7
Rab GDP dissociation inhibitor beta OS=Homo sapiens GN=GDI2 PE=1 SV=2 - [GDIB_HUMAN]	7
Mitochondrial glutamate carrier 1 OS=Homo sapiens GN=SLC25A22 PE=1 SV=1 - [GHC1_HUMAN]	7
Guanine nucleotide-binding protein G(q) subunit alpha OS=Homo sapiens GN=GNAQ PE=1 SV=4 - [GNAQ_HUMAN]	7
Isoform 3 of Neuronal membrane glycoprotein M6-a OS=Homo sapiens GN=GPM6A - [GPM6A_HUMAN]	7
Hemoglobin subunit delta OS=Homo sapiens GN=HBD PE=1 SV=2 - [HBD_HUMAN]	7
Hypoxanthine-guanine phosphoribosyltransferase OS=Homo sapiens GN=HPRT1 PE=1 SV=2 - [HPRT_HUMAN]	7
Epoxide hydrolase 1 OS=Homo sapiens GN=EPHX1 PE=1 SV=1 - [HYEP_HUMAN]	7
Kelch repeat and BTB domain-containing protein 11 OS=Homo sapiens GN=KBTBD11 PE=1 SV=1 - [KBTBB_HUMAN]	7
Isoform 2 of Calcium/calmodulin-dependent protein kinase type 1D OS=Homo sapiens GN=CAMK1D - [KCC1D_HUMAN]	7
UMP-CMP kinase OS=Homo sapiens GN=CMPK1 PE=1 SV=3 - [KCY_HUMAN]	7
Isoform 3 of Neural cell adhesion molecule L1 OS=Homo sapiens GN=L1CAM - [L1CAM_HUMAN]	7
Mitogen-activated protein kinase 1 OS=Homo sapiens GN=MAPK1 PE=1 SV=3 - [MK01_HUMAN]	7

Nck-associated protein 1 OS=Homo sapiens GN=NCKAP1 PE=1 SV=1 - [NCKP1_HUMAN]	7
NADH dehydrogenase [ubiquinone] 1 beta subcomplex subunit 10 OS=Homo sapiens GN=NDUFB10 PE=1 SV=3 - [NDUBA_HUMAN]	7
Protein NipSnap homolog 1 OS=Homo sapiens GN=NIPSNAP1 PE=1 SV=1 - [NIPS1_HUMAN]	7
Neuroplastin OS=Homo sapiens GN=NPTN PE=1 SV=2 - [NPTN_HUMAN]	7
Isoform 3 of Neuronal cell adhesion molecule OS=Homo sapiens GN=NRCAM - [NRCAM_HUMAN]	7
Brevican core protein OS=Homo sapiens GN=BCAN PE=1 SV=2 - [PGCB_HUMAN]	7
Phosphoglucomutase-1 OS=Homo sapiens GN=PGM1 PE=1 SV=3 - [PGM1_HUMAN]	7
Protein-L-isoaspartate(D-aspartate) O-methyltransferase OS=Homo sapiens GN=PCMT1 PE=1 SV=4 - [PIMT_HUMAN]	7
Isoform 2 of Serine/threonine-protein phosphatase 2B catalytic subunit alpha isoform OS=Homo sapiens GN=PPP3CA - [PP2BA_HUMAN]	7
Peptidyl-prolyl cis-trans isomerase B OS=Homo sapiens GN=PPIB PE=1 SV=2 - [PPIB_HUMAN]	7
Peroxiredoxin-2 OS=Homo sapiens GN=PRDX2 PE=1 SV=5 - [PRDX2_HUMAN]	7
Ras-related protein Rab-2A OS=Homo sapiens GN=RAB2A PE=1 SV=1 - [RAB2A_HUMAN]	7
Ras-related protein Rab-5C OS=Homo sapiens GN=RAB5C PE=1 SV=2 - [RAB5C_HUMAN]	7
Isoform 3 of Ras-related protein Rap-1b OS=Homo sapiens GN=RAP1B - [RAP1B_HUMAN]	7
60S ribosomal protein L6 OS=Homo sapiens GN=RPL6 PE=1 SV=3 - [RL6_HUMAN]	7
Reticulon-4 OS=Homo sapiens GN=RTN4 PE=1 SV=2 - [RTN4_HUMAN]	7
D-3-phosphoglycerate dehydrogenase OS=Homo sapiens GN=PHGDH PE=1 SV=4 - [SERA_HUMAN]	7
Phosphoserine aminotransferase OS=Homo sapiens GN=PSAT1 PE=1 SV=2 - [SERC_HUMAN]	7
Endophilin-A1 OS=Homo sapiens GN=SH3GL2 PE=1 SV=1 - [SH3G2_HUMAN]	7
Transgelin-3 OS=Homo sapiens GN=TAGLN3 PE=1 SV=2 - [TAGL3_HUMAN]	7
Isoform 3 of T-complex protein 1 subunit eta OS=Homo sapiens GN=CCT7 - [TCPH_HUMAN]	7
Transketolase OS=Homo sapiens GN=TKT PE=1 SV=3 - [TKT_HUMAN]	7
Isoform 2 of X-ray repair cross-complementing protein 6 OS=Homo sapiens GN=XRCC6 - [XRCC6_HUMAN]	7
Clathrin coat assembly protein AP180 OS=Homo sapiens GN=SNAP91 PE=1 SV=1 - [E9PDG8_HUMAN]	7
Isoform 2 of Flotillin-1 OS=Homo sapiens GN=FLOT1 - [FLOT1_HUMAN]	7
Isoform 2 of Annexin A11 OS=Homo sapiens GN=ANXA11 - [ANX11_HUMAN]	7
Septin-2 OS=Homo sapiens GN=SEPT2 PE=1 SV=1 - [B5MCX3_HUMAN]	7
cGMP-dependent 3',5'-cyclic phosphodiesterase OS=Homo sapiens GN=PDE2A PE=1 SV=2 - [E9PEF1_HUMAN]	7
V-type proton ATPase subunit B, kidney isoform OS=Homo sapiens GN=ATP6V1B1 PE=1 SV=1 - [C9JL73_HUMAN]	7
Isoform 3 of Receptor-type tyrosine-protein phosphatase zeta OS=Homo sapiens GN=PTPRZ1 - [PTPRZ_HUMAN]	7
Isoform 2 of Thioredoxin-dependent peroxide reductase, mitochondrial OS=Homo sapiens GN=PRDX3 - [PRDX3_HUMAN]	7
V-type proton ATPase subunit d 1 OS=Homo sapiens GN=ATP6V0D1 PE=1 SV=1 - [R4GN72_HUMAN]	7
Acetyltransferase component of pyruvate dehydrogenase complex (Fragment) OS=Homo sapiens GN=DLAT PE=1 SV=1 - [H0YDD4_HUMAN]	7
Band 4.1-like protein 2 OS=Homo sapiens GN=EPB41L2 PE=1 SV=1 - [I6L9B1_HUMAN]	7
Malic enzyme (Fragment) OS=Homo sapiens GN=ME3 PE=1 SV=4 - [E9PMB9_HUMAN]	7
Mitochondrial carrier homolog 2 (Fragment) OS=Homo sapiens GN=MTCH2 PE=1 SV=4 - [E9PIE4_HUMAN]	7
cAMP-dependent protein kinase catalytic subunit beta OS=Homo sapiens GN=PRKACB PE=1 SV=1 - [A0A087WVC4_HUMAN]	7
Isoform 2 of Acyl-coenzyme A thioesterase 9, mitochondrial OS=Homo sapiens GN=ACOT9 - [ACOT9_HUMAN]	6
Beta-centractin OS=Homo sapiens GN=ACTR1B PE=1 SV=1 - [ACTY_HUMAN]	6
Alpha-centractin OS=Homo sapiens GN=ACTR1A PE=1 SV=1 - [ACTZ_HUMAN]	6
Isoform 2 of Beta-adducin OS=Homo sapiens GN=ADD2 - [ADDB_HUMAN]	6
Isoform 3 of Delta-1-pyrroline-5-carboxylate dehydrogenase, mitochondrial OS=Homo sapiens GN=ALDH4A1 - [AL4A1_HUMAN]	6
Adipocyte plasma membrane-associated protein OS=Homo sapiens GN=APMAP PE=1 SV=2 - [APMAP_HUMAN]	6
Calreticulin OS=Homo sapiens GN=CALR PE=1 SV=1 - [CALR_HUMAN]	6
Isoform 2 of Adenylyl cyclase-associated protein 1 OS=Homo sapiens GN=CAP1 - [CAP1_HUMAN]	6
Cyclin-dependent-like kinase 5 OS=Homo sapiens GN=CDK5 PE=1 SV=3 - [CDK5_HUMAN]	6
Isoform 4 of Clusterin OS=Homo sapiens GN=CLU - [CLUS_HUMAN]	6
Dihydropteridine reductase OS=Homo sapiens GN=QDPR PE=1 SV=2 - [DHPR_HUMAN]	6
Elongation factor 1-gamma OS=Homo sapiens GN=EEF1G PE=1 SV=3 - [EF1G_HUMAN]	6
Isoform Cytoplasmic of Fumarate hydratase, mitochondrial OS=Homo sapiens GN=FH - [FUMH_HUMAN]	6
Guanine nucleotide-binding protein G(I)/G(S)/G(T) subunit beta-2 OS=Homo sapiens GN=GNB2 PE=1 SV=3 - [GBB2_HUMAN]	6
Guanine nucleotide-binding protein G(z) subunit alpha OS=Homo sapiens GN=GNAZ PE=2 SV=3 - [GNAZ_HUMAN]	6
Glutathione S-transferase Mu 2 OS=Homo sapiens GN=GSTM2 PE=1 SV=2 - [GSTM2_HUMAN]	6

Histone H1.2 OS=Homo sapiens GN=HIST1H1C PE=1 SV=2 - [H12_HUMAN]	6
Isoform 3 of Heterogeneous nuclear ribonucleoprotein K OS=Homo sapiens GN=HNRNPK - [HNRPK_HUMAN]	6
Neuron-specific calcium-binding protein hippocalcin OS=Homo sapiens GN=HPCA PE=1 SV=2 - [HPCA_HUMAN]	6
Hippocalcin-like protein 4 OS=Homo sapiens GN=HPCAL4 PE=1 SV=3 - [HPCL4_HUMAN]	6
Hyaluronan and proteoglycan link protein 2 OS=Homo sapiens GN=HAPLN2 PE=1 SV=1 - [HPLN2_HUMAN]	6
Isoform 2 of Hydroxysteroid dehydrogenase-like protein 2 OS=Homo sapiens GN=HSDL2 - [HSDL2_HUMAN]	6
Eukaryotic initiation factor 4A-I OS=Homo sapiens GN=EIF4A1 PE=1 SV=1 - [IF4A1_HUMAN]	6
Isoform 5 of Interleukin enhancer-binding factor 3 OS=Homo sapiens GN=ILF3 - [ILF3_HUMAN]	6
Adenylate kinase 4, mitochondrial OS=Homo sapiens GN=AK4 PE=1 SV=1 - [KAD4_HUMAN]	6
Isoform 2 of cAMP-dependent protein kinase catalytic subunit alpha OS=Homo sapiens GN=PRKACA - [KAPCA_HUMAN]	6
Creatine kinase U-type, mitochondrial OS=Homo sapiens GN=CKMT1A PE=1 SV=1 - [KCRU_HUMAN]	6
Isoform 2 of Kinesin-like protein KIF2A OS=Homo sapiens GN=KIF2A - [KIF2A_HUMAN]	6
Galectin-1 OS=Homo sapiens GN=LGALS1 PE=1 SV=2 - [LEG1_HUMAN]	6
Leucine-rich PPR motif-containing protein, mitochondrial OS=Homo sapiens GN=LRPPRC PE=1 SV=3 - [LPPRC_HUMAN]	6
Isoform 2 of Myelin-associated glycoprotein OS=Homo sapiens GN=MAG - [MAG_HUMAN]	6
Microtubule-associated protein RP/EB family member 3 OS=Homo sapiens GN=MAPRE3 PE=1 SV=1 - [MARE3_HUMAN]	6
Moesin OS=Homo sapiens GN=MSN PE=1 SV=3 - [MOES_HUMAN]	6
Dihydrolipoyllysine-residue succinyltransferase component of 2-oxoglutarate dehydrogenase complex, mitochondrial OS=Homo sapiens GN=DLST PE=1 SV=4 - [ODO2_HUMAN]	6
Pyridoxal kinase OS=Homo sapiens GN=PDXK PE=1 SV=1 - [PDXK_HUMAN]	6
Peripherin OS=Homo sapiens GN=PRPH PE=1 SV=2 - [PERI_HUMAN]	6
Isoform 3 of Phosphatidylinositol 5-phosphate 4-kinase type-2 gamma OS=Homo sapiens GN=PIP4K2C - [PI42C_HUMAN]	6
POTE ankyrin domain family member E OS=Homo sapiens GN=POTEE PE=1 SV=3 - [POTEE_HUMAN]	6
Isoform Cytoplasmic+peroxisomal of Peroxiredoxin-5, mitochondrial OS=Homo sapiens GN=PRDX5 - [PRDX5_HUMAN]	6
Peroxiredoxin-6 OS=Homo sapiens GN=PRDX6 PE=1 SV=3 - [PRDX6_HUMAN]	6
Isoform 2 of Bifunctional purine biosynthesis protein PURH OS=Homo sapiens GN=ATIC - [PUR9_HUMAN]	6
Cytochrome b-c1 complex subunit 7 OS=Homo sapiens GN=UQCRB PE=1 SV=2 - [QCR7_HUMAN]	6
Isoform 3 of Quinone oxidoreductase OS=Homo sapiens GN=CRYZ - [QOR_HUMAN]	6
Ras-related protein Rab-3A OS=Homo sapiens GN=RAB3A PE=1 SV=1 - [RAB3A_HUMAN]	6
Ras-related protein Rab-5B OS=Homo sapiens GN=RAB5B PE=1 SV=1 - [RAB5B_HUMAN]	6
Ras-related protein Rab-6A OS=Homo sapiens GN=RAB6A PE=1 SV=3 - [RAB6A_HUMAN]	6
Ras-related protein Rab-6B OS=Homo sapiens GN=RAB6B PE=1 SV=1 - [RAB6B_HUMAN]	6
Rho GTPase-activating protein 1 OS=Homo sapiens GN=ARHGAP1 PE=1 SV=1 - [RHG01_HUMAN]	6
Transforming protein RhoA OS=Homo sapiens GN=RHOA PE=1 SV=1 - [RHOA_HUMAN]	6
Isoform 2 of Heterogeneous nuclear ribonucleoprotein A3 OS=Homo sapiens GN=HNRNPA3 - [ROA3_HUMAN]	6
Signal-regulatory protein beta-1 isoform 3 OS=Homo sapiens GN=SIRPB1 PE=1 SV=1 - [SIRBL_HUMAN]	6
Alanine--tRNA ligase, cytoplasmic OS=Homo sapiens GN=AARS PE=1 SV=2 - [SYAC_HUMAN]	6
Isoform 2-4 of Alpha-synuclein OS=Homo sapiens GN=SNCA - [SYUA_HUMAN]	6
Transgelin OS=Homo sapiens GN=TAGLN PE=1 SV=4 - [TAGL_HUMAN]	6
Transitional endoplasmic reticulum ATPase OS=Homo sapiens GN=VCP PE=1 SV=4 - [TERA_HUMAN]	6
Ubiquitin-conjugating enzyme E2 N OS=Homo sapiens GN=UBE2N PE=1 SV=1 - [UBE2N_HUMAN]	6
V-type proton ATPase subunit D OS=Homo sapiens GN=ATP6V1D PE=1 SV=1 - [VATD_HUMAN]	6
Isoform 1 of Vinculin OS=Homo sapiens GN=VCL - [VINC_HUMAN]	6
Isoform 3 of AP-3 complex subunit beta-2 OS=Homo sapiens GN=AP3B2 - [AP3B2_HUMAN]	6
Isoform 2 of Protein kinase C gamma type OS=Homo sapiens GN=PRKCG - [KPCG_HUMAN]	6
Isoform 2 of Dihydrolipoyl dehydrogenase, mitochondrial OS=Homo sapiens GN=DLD - [DLDH_HUMAN]	6
Tropomodulin 2 (Neuronal), isoform CRA_a OS=Homo sapiens GN=TMOD2 PE=1 SV=1 - [G5EA42_HUMAN]	6
Isoform 4 of Heterogeneous nuclear ribonucleoprotein R OS=Homo sapiens GN=HNRNPR - [HNRPR_HUMAN]	6
Adenylyl cyclase-associated protein OS=Homo sapiens GN=CAP2 PE=1 SV=1 - [E9PDI2_HUMAN]	6
Isoform 2 of Heat shock protein 75 kDa, mitochondrial OS=Homo sapiens GN=TRAP1 - [TRAP1_HUMAN]	6
Myosin light polypeptide 6 OS=Homo sapiens GN=MYL6 PE=1 SV=1 - [F8W1R7_HUMAN]	6
Isoform 2 of Succinate dehydrogenase [ubiquinone] flavoprotein subunit, mitochondrial OS=Homo sapiens GN=SDHA - [SDHA_HUMAN]	6
Neuronal-specific septin-3 OS=Homo sapiens GN=SEPT3 PE=1 SV=1 - [B1AHR1_HUMAN]	6
Ras-related protein Rab-7a (Fragment) OS=Homo sapiens GN=RAB7A PE=1 SV=1 - [C9J592_HUMAN]	6

Protein disulfide-isomerase A3 (Fragment) OS=Homo sapiens GN=PDIA3 PE=1 SV=1 - [H7BZJ3_HUMAN]	6
Epididymis luminal protein 189 OS=Homo sapiens GN=DKFZp686J1372 PE=1 SV=1 - [Q5HYB6_HUMAN]	6
Ezrin OS=Homo sapiens GN=EZR PE=1 SV=3 - [E7EQR4_HUMAN]	6
Carbonic anhydrase 1 (Fragment) OS=Homo sapiens GN=CA1 PE=1 SV=4 - [E5RH81_HUMAN]	6
NADH dehydrogenase [ubiquinone] 1 alpha subcomplex subunit 10, mitochondrial OS=Homo sapiens GN=NDUFA10 PE=1 SV=1 - [A0A087WXC5_HUMAN]	6
AFG3-like protein 2 OS=Homo sapiens GN=AFG3L2 PE=1 SV=2 - [AFG32_HUMAN]	5
Isoform 3 of Apoptosis-inducing factor 1, mitochondrial OS=Homo sapiens GN=AIFM1 - [AIFM1_HUMAN]	5
Isoform 2 of Amphiphysin OS=Homo sapiens GN=AMPH - [AMPH_HUMAN]	5
Apolipoprotein D OS=Homo sapiens GN=APOD PE=1 SV=1 - [APOD_HUMAN]	5
Isoform 2 of Sodium/potassium-transporting ATPase subunit beta-1 OS=Homo sapiens GN=ATP1B1 - [AT1B1_HUMAN]	5
Sodium/potassium-transporting ATPase subunit beta-2 OS=Homo sapiens GN=ATP1B2 PE=1 SV=3 - [AT1B2_HUMAN]	5
Isoform ZA of Plasma membrane calcium-transporting ATPase 2 OS=Homo sapiens GN=ATP2B2 - [AT2B2_HUMAN]	5
Isoform 3 of Brain-specific angiogenesis inhibitor 1-associated protein 2 OS=Homo sapiens GN=BAIAP2 - [BAIP2_HUMAN]	5
Isoform BIN1-10-13 of Myc box-dependent-interacting protein 1 OS=Homo sapiens GN=BIN1 - [BIN1_HUMAN]	5
Cell adhesion molecule 3 OS=Homo sapiens GN=CADM3 PE=1 SV=1 - [CADM3_HUMAN]	5
Carbonic anhydrase 2 OS=Homo sapiens GN=CA2 PE=1 SV=2 - [CAH2_HUMAN]	5
Calmodulin OS=Homo sapiens GN=CALM1 PE=1 SV=2 - [CALM_HUMAN]	5
Cell division control protein 42 homolog OS=Homo sapiens GN=CDC42 PE=1 SV=2 - [CDC42_HUMAN]	5
MICOS complex subunit MIC19 OS=Homo sapiens GN=CHCHD3 PE=1 SV=1 - [MIC19_HUMAN]	5
Gap junction alpha-1 protein OS=Homo sapiens GN=GJA1 PE=1 SV=2 - [CXA1_HUMAN]	5
Dynactin subunit 2 OS=Homo sapiens GN=DCTN2 PE=1 SV=4 - [DCTN2_HUMAN]	5
Spliceosome RNA helicase DDX39B OS=Homo sapiens GN=DDX39B PE=1 SV=1 - [DX39B_HUMAN]	5
Enoyl-CoA hydratase, mitochondrial OS=Homo sapiens GN=ECHS1 PE=1 SV=4 - [ECHM_HUMAN]	5
Elongation factor 2 OS=Homo sapiens GN=EEF2 PE=1 SV=4 - [EF2_HUMAN]	5
Endonuclease domain-containing 1 protein OS=Homo sapiens GN=ENDOD1 PE=1 SV=2 - [ENDD1_HUMAN]	5
Electron transfer flavoprotein subunit beta OS=Homo sapiens GN=ETFB PE=1 SV=3 - [ETFB_HUMAN]	5
F-box only protein 2 OS=Homo sapiens GN=FBXO2 PE=1 SV=2 - [FBX2_HUMAN]	5
Guanine nucleotide-binding protein subunit beta-4 OS=Homo sapiens GN=GNB4 PE=1 SV=3 - [GBB4_HUMAN]	5
Guanine nucleotide-binding protein subunit alpha-11 OS=Homo sapiens GN=GNA11 PE=1 SV=2 - [GNA11_HUMAN]	5
Guanine nucleotide-binding protein subunit alpha-13 OS=Homo sapiens GN=GNA13 PE=1 SV=2 - [GNA13_HUMAN]	5
Guanine nucleotide-binding protein G(k) subunit alpha OS=Homo sapiens GN=GNAI3 PE=1 SV=3 - [GNAI3_HUMAN]	5
Guanine nucleotide-binding protein-like 1 OS=Homo sapiens GN=GNL1 PE=1 SV=2 - [GNL1_HUMAN]	5
Histone H2B type 1-K OS=Homo sapiens GN=HIST1H2BK PE=1 SV=3 - [H2B1K_HUMAN]	5
Hemoglobin subunit alpha OS=Homo sapiens GN=HBA1 PE=1 SV=2 - [HBA_HUMAN]	5
Hippocalcin-like protein 1 OS=Homo sapiens GN=HPCAL1 PE=1 SV=3 - [HPCL1_HUMAN]	5
Intercellular adhesion molecule 5 OS=Homo sapiens GN=ICAM5 PE=1 SV=3 - [ICAM5_HUMAN]	5
ATP-dependent 6-phosphofructokinase, muscle type OS=Homo sapiens GN=PFKM PE=1 SV=2 - [PFKAM_HUMAN]	5
ATP-dependent 6-phosphofructokinase, liver type OS=Homo sapiens GN=PFKL PE=1 SV=6 - [PFKAL_HUMAN]	5
Adenylate kinase isoenzyme 1 OS=Homo sapiens GN=AK1 PE=1 SV=3 - [KAD1_HUMAN]	5
cAMP-dependent protein kinase type II-beta regulatory subunit OS=Homo sapiens GN=PRKAR2B PE=1 SV=3 - [KAP3_HUMAN]	5
Isoform 6 of Kinesin-like protein KIF21A OS=Homo sapiens GN=KIF21A - [KI21A_HUMAN]	5
Limbic system-associated membrane protein OS=Homo sapiens GN=LSAMP PE=1 SV=2 - [LSAMP_HUMAN]	5
Metallo-beta-lactamase domain-containing protein 2 OS=Homo sapiens GN=MBLAC2 PE=1 SV=3 - [MBLC2_HUMAN]	5
Isoform 2 of Mitogen-activated protein kinase 3 OS=Homo sapiens GN=MAPK3 - [MK03_HUMAN]	5
Dual specificity mitogen-activated protein kinase kinase 2 OS=Homo sapiens GN=MAP2K2 PE=1 SV=1 - [MP2K2_HUMAN]	5
Isoform 2 of Protein NDRG4 OS=Homo sapiens GN=NDRG4 - [NDRG4_HUMAN]	5
Protein NipSnap homolog 3A OS=Homo sapiens GN=NIPSNAP3A PE=1 SV=2 - [NPS3A_HUMAN]	5
Protein-arginine deiminase type-2 OS=Homo sapiens GN=PADI2 PE=2 SV=2 - [PADI2_HUMAN]	5
Prenylcysteine oxidase 1 OS=Homo sapiens GN=PCYOX1 PE=1 SV=3 - [PCYOX_HUMAN]	5
Programmed cell death 6-interacting protein OS=Homo sapiens GN=PDCD6IP PE=1 SV=1 - [PDC6I_HUMAN]	5
Programmed cell death protein 6 OS=Homo sapiens GN=PDCD6 PE=1 SV=1 - [PDCD6_HUMAN]	5
Phosphoglycerate mutase 1 OS=Homo sapiens GN=PGAM1 PE=1 SV=2 - [PGAM1_HUMAN]	5
Isoform 4 of Phosphatidylinositol 4-phosphate 5-kinase type-1 gamma OS=Homo sapiens GN=PIP5K1C - [PI51C_HUMAN]	5

Palmitoyl-protein thioesterase 1 OS=Homo sapiens GN=PPT1 PE=1 SV=1 - [PPT1_HUMAN]	5
Pyruvate carboxylase, mitochondrial OS=Homo sapiens GN=PC PE=1 SV=2 - [PYC_HUMAN]	5
Ras-related protein Rab-10 OS=Homo sapiens GN=RAB10 PE=1 SV=1 - [RAB10_HUMAN]	5
Ras-related C3 botulinum toxin substrate 3 OS=Homo sapiens GN=RAC3 PE=1 SV=1 - [RAC3_HUMAN]	5
60S ribosomal protein L12 OS=Homo sapiens GN=RPL12 PE=1 SV=1 - [RL12_HUMAN]	5
60S ribosomal protein L4 OS=Homo sapiens GN=RPL4 PE=1 SV=5 - [RL4_HUMAN]	5
Isoform 2 of Rap1 GTPase-activating protein 1 OS=Homo sapiens GN=RAP1GAP - [RPGP1_HUMAN]	5
40S ribosomal protein S3 OS=Homo sapiens GN=RPS3 PE=1 SV=2 - [RS3_HUMAN]	5
Isoform RTN1-B of Reticulon-1 OS=Homo sapiens GN=RTN1 - [RTN1_HUMAN]	5
Vesicle-trafficking protein SEC22b OS=Homo sapiens GN=SEC22B PE=1 SV=4 - [SC22B_HUMAN]	5
Secernin-1 OS=Homo sapiens GN=SCRN1 PE=1 SV=2 - [SCRN1_HUMAN]	5
Isoform I of Septin-6 OS=Homo sapiens GN=SEPT6 - [SEPT6_HUMAN]	5
Isoform 3 of Septin-9 OS=Homo sapiens GN=SEPT9 - [SEPT9_HUMAN]	5
Sorting nexin-1 OS=Homo sapiens GN=SNX1 PE=1 SV=3 - [SNX1_HUMAN]	5
Erythrocyte band 7 integral membrane protein OS=Homo sapiens GN=STOM PE=1 SV=3 - [STOM_HUMAN]	5
Isoform 2 of Tubulin alpha chain-like 3 OS=Homo sapiens GN=TUBAL3 - [TBAL3_HUMAN]	5
T-complex protein 1 subunit alpha OS=Homo sapiens GN=TCP1 PE=1 SV=1 - [TCPA_HUMAN]	5
Tropomyosin alpha-4 chain OS=Homo sapiens GN=TPM4 PE=1 SV=3 - [TPM4_HUMAN]	5
Isoform 2 of Tripeptidyl-peptidase 1 OS=Homo sapiens GN=TPP1 - [TPP1_HUMAN]	5
Tubulin polymerization-promoting protein OS=Homo sapiens GN=TPPP PE=1 SV=1 - [TPPP_HUMAN]	5
Tricarboxylate transport protein, mitochondrial OS=Homo sapiens GN=SLC25A1 PE=1 SV=2 - [TXTP_HUMAN]	5
Vesicle-associated membrane protein-associated protein A OS=Homo sapiens GN=VAPA PE=1 SV=3 - [VAPA_HUMAN]	5
Vesicle-associated membrane protein-associated protein B/C OS=Homo sapiens GN=VAPB PE=1 SV=3 - [VAPB_HUMAN]	5
Synaptic vesicle membrane protein VAT-1 homolog OS=Homo sapiens GN=VAT1 PE=1 SV=2 - [VAT1_HUMAN]	5
Isoform 4 of Dematin OS=Homo sapiens GN=DMTN - [DEMA_HUMAN]	5
60S ribosomal protein L7 OS=Homo sapiens GN=RPL7 PE=1 SV=1 - [A8MUD9_HUMAN]	5
Isoform 4 of Superoxide dismutase [Mn], mitochondrial OS=Homo sapiens GN=SOD2 - [SODM_HUMAN]	5
ANXA4 protein OS=Homo sapiens GN=ANXA4 PE=1 SV=1 - [Q6P452_HUMAN]	5
Protein phosphatase 3 (Formerly 2B), catalytic subunit, beta isoform (Calcineurin A beta), isoform CRA_a OS=Homo sapiens GN=PPP3CB PE=1 SV=1 - [Q5F2F8_HUMAN]	5
PH and SEC7 domain-containing protein 3 OS=Homo sapiens GN=PSD3 PE=1 SV=1 - [B4DKF8_HUMAN]	5
Heterogeneous nuclear ribonucleoproteins C1/C2 OS=Homo sapiens GN=HNRNPC PE=1 SV=1 - [G3V576_HUMAN]	5
Copine-8 OS=Homo sapiens GN=CPNE8 PE=1 SV=2 - [E7ENV7_HUMAN]	5
Protein SOGA3 OS=Homo sapiens GN=SOGA3 PE=1 SV=1 - [E9PJP2_HUMAN]	5
Protein kinase C and casein kinase substrate in neurons protein 1 OS=Homo sapiens GN=PACSIN1 PE=1 SV=1 - [F6U236_HUMAN]	5
CaM kinase-like vesicle-associated protein OS=Homo sapiens GN=CAMKV PE=1 SV=1 - [C9J9E2_HUMAN]	5
ATP synthase subunit g, mitochondrial OS=Homo sapiens GN=ATP5L PE=1 SV=1 - [E9PN17_HUMAN]	5
Isoform 3 of Excitatory amino acid transporter 2 OS=Homo sapiens GN=SLC1A2 - [EAA2_HUMAN]	5
Sorcin OS=Homo sapiens GN=SRI PE=1 SV=1 - [C9J0K6_HUMAN]	5
Peroxisomal multifunctional enzyme type 2 OS=Homo sapiens GN=HSD17B4 PE=1 SV=3 - [E7ER27_HUMAN]	5
Ras/Rap GTPase-activating protein SynGAP (Fragment) OS=Homo sapiens GN=SYNGAP1 PE=1 SV=2 - [B7ZCA0_HUMAN]	5
Protein deglycase DJ-1 OS=Homo sapiens GN=PARK7 PE=1 SV=1 - [K7ELW0_HUMAN]	5
Microtubule-associated protein RP/EB family member 2 (Fragment) OS=Homo sapiens GN=MAPRE2 PE=1 SV=1 - [K7EL66_HUMAN]	5
Isoform 2 of Guanine nucleotide-binding protein G(I)/G(S)/G(T) subunit beta-1 OS=Homo sapiens GN=GNB1 - [GBB1_HUMAN]	5
Interleukin enhancer-binding factor 2 OS=Homo sapiens GN=ILF2 PE=1 SV=1 - [B4DY09_HUMAN]	5
Tubulointerstitial nephritis antigen-like OS=Homo sapiens GN=TINAGL1 PE=1 SV=1 - [F6SDV2_HUMAN]	5
Electron transfer flavoprotein subunit alpha, mitochondrial (Fragment) OS=Homo sapiens GN=ETFA PE=1 SV=4 - [H0YL12_HUMAN]	5
Gamma-adducin OS=Homo sapiens GN=ADD3 PE=1 SV=1 - [A0A087WX08_HUMAN]	5
Isoform Delta-3 of Serine/threonine-protein phosphatase 2A 56 kDa regulatory subunit delta isoform OS=Homo sapiens GN=PPP2R5D - [2A5D_HUMAN]	4
Serine/threonine-protein phosphatase 2A 55 kDa regulatory subunit B alpha isoform OS=Homo sapiens GN=PPP2R2A PE=1 SV=1 - [2ABA_HUMAN]	4
Isoform 2 of Very long-chain specific acyl-CoA dehydrogenase, mitochondrial OS=Homo sapiens GN=ACADVL - [ACADV_HUMAN]	4
Isoform 2 of Acyl-coenzyme A thioesterase 13 OS=Homo sapiens GN=ACOT13 - [ACO13_HUMAN]	4
Isoform 3 of Arf-GAP with GTPase, ANK repeat and PH domain-containing protein 3 OS=Homo sapiens GN=AGAP3 - [AGAP3_HUMAN]	4
Alcohol dehydrogenase [NADP(+)] OS=Homo sapiens GN=AKR1A1 PE=1 SV=3 - [AK1A1_HUMAN]	4

4-trimethylaminobutyraldehyde dehydrogenase OS=Homo sapiens GN=ALDH9A1 PE=1 SV=3 - [AL9A1_HUMAN]	4
Isoform 2 of AP-2 complex subunit mu OS=Homo sapiens GN=AP2M1 - [AP2M1_HUMAN]	4
ADP-ribosylation factor 4 OS=Homo sapiens GN=ARF4 PE=1 SV=3 - [ARF4_HUMAN]	4
Actin-related protein 2/3 complex subunit 2 OS=Homo sapiens GN=ARPC2 PE=1 SV=1 - [ARPC2_HUMAN]	4
Isoform ZG of Plasma membrane calcium-transporting ATPase 3 OS=Homo sapiens GN=ATP2B3 - [AT2B3_HUMAN]	4
Calpain-1 catalytic subunit OS=Homo sapiens GN=CAPN1 PE=1 SV=1 - [CAN1_HUMAN]	4
Isoform 2 of Macrophage-capping protein OS=Homo sapiens GN=CAPG - [CAPG_HUMAN]	4
Isoform 2 of Calcium-dependent secretion activator 1 OS=Homo sapiens GN=CADPS - [CAPS1_HUMAN]	4
Isoform 3 of Cell cycle control protein 50A OS=Homo sapiens GN=TMEM30A - [CC50A_HUMAN]	4
10 kDa heat shock protein, mitochondrial OS=Homo sapiens GN=HSPE1 PE=1 SV=2 - [CH10_HUMAN]	4
Cytochrome c oxidase subunit 2 OS=Homo sapiens GN=MT-CO2 PE=1 SV=1 - [COX2_HUMAN]	4
Cytochrome c oxidase subunit 5B, mitochondrial OS=Homo sapiens GN=COX5B PE=1 SV=2 - [COX5B_HUMAN]	4
Isoform 5 of Dynactin subunit 1 OS=Homo sapiens GN=DCTN1 - [DCTN1_HUMAN]	4
ATP-dependent RNA helicase A OS=Homo sapiens GN=DHX9 PE=1 SV=4 - [DHX9_HUMAN]	4
Isoform 8 of Disks large homolog 1 OS=Homo sapiens GN=DLG1 - [DLG1_HUMAN]	4
DnaJ homolog subfamily A member 1 OS=Homo sapiens GN=DNAJA1 PE=1 SV=2 - [DNJA1_HUMAN]	4
Ectonucleotide pyrophosphatase/phosphodiesterase family member 6 OS=Homo sapiens GN=ENPP6 PE=1 SV=2 - [ENPP6_HUMAN]	4
Endoplasmic reticulum resident protein 29 OS=Homo sapiens GN=ERP29 PE=1 SV=4 - [ERP29_HUMAN]	4
Protein FAM49B OS=Homo sapiens GN=FAM49B PE=1 SV=1 - [FA49B_HUMAN]	4
Isoform Gamma-A of Fibrinogen gamma chain OS=Homo sapiens GN=FGG - [FIBG_HUMAN]	4
Fascin OS=Homo sapiens GN=FSCN1 PE=1 SV=3 - [FSCN1_HUMAN]	4
Isoform 2 of Histone H1.0 OS=Homo sapiens GN=H1F0 - [H10_HUMAN]	4
Histone H2A type 1-H OS=Homo sapiens GN=HIST1H2AH PE=1 SV=3 - [H2A1H_HUMAN]	4
Hydroxyacyl-coenzyme A dehydrogenase, mitochondrial OS=Homo sapiens GN=HADH PE=1 SV=3 - [HCDH_HUMAN]	4
Heme-binding protein 1 OS=Homo sapiens GN=HEBP1 PE=1 SV=1 - [HEBP1_HUMAN]	4
Heterogeneous nuclear ribonucleoprotein H2 OS=Homo sapiens GN=HNRNPH2 PE=1 SV=1 - [HNRH2_HUMAN]	4
Isoform 2 of Heterogeneous nuclear ribonucleoprotein L OS=Homo sapiens GN=HNRNPL - [HNRPL_HUMAN]	4
IQ motif and SEC7 domain-containing protein 1 OS=Homo sapiens GN=IQSEC1 PE=1 SV=1 - [IQEC1_HUMAN]	4
Protein KIAA1045 OS=Homo sapiens GN=KIAA1045 PE=1 SV=2 - [K1045_HUMAN]	4
Keratin, type I cytoskeletal 14 OS=Homo sapiens GN=KRT14 PE=1 SV=4 - [K1C14_HUMAN]	4
Keratin, type II cytoskeletal 5 OS=Homo sapiens GN=KRT5 PE=1 SV=3 - [K2C5_HUMAN]	4
Guanylate kinase OS=Homo sapiens GN=GUK1 PE=1 SV=2 - [KGUA_HUMAN]	4
LETM1 and EF-hand domain-containing protein 1, mitochondrial OS=Homo sapiens GN=LETM1 PE=1 SV=1 - [LETM1_HUMAN]	4
NAD-dependent malic enzyme, mitochondrial OS=Homo sapiens GN=ME2 PE=1 SV=1 - [MAOM_HUMAN]	4
Isoform 2 of Methylcrotonoyl-CoA carboxylase beta chain, mitochondrial OS=Homo sapiens GN=MCCC2 - [MCCB_HUMAN]	4
Myelin proteolipid protein OS=Homo sapiens GN=PLP1 PE=1 SV=2 - [MYPR_HUMAN]	4
NADH-cytochrome b5 reductase 1 OS=Homo sapiens GN=CYB5R1 PE=1 SV=1 - [NB5R1_HUMAN]	4
Neurocalcin-delta OS=Homo sapiens GN=NCALD PE=1 SV=2 - [NCALD_HUMAN]	4
Isoform 2 of Neuronal calcium sensor 1 OS=Homo sapiens GN=NCS1 - [NCS1_HUMAN]	4
Protein NDRG1 OS=Homo sapiens GN=NDRG1 PE=1 SV=1 - [NDRG1_HUMAN]	4
NADH dehydrogenase [ubiquinone] 1 alpha subcomplex subunit 5 OS=Homo sapiens GN=NDUFA5 PE=1 SV=3 - [NDUA5_HUMAN]	4
NADH dehydrogenase [ubiquinone] 1 alpha subcomplex subunit 7 OS=Homo sapiens GN=NDUFA7 PE=1 SV=3 - [NDUA7_HUMAN]	4
NADH dehydrogenase [ubiquinone] 1 alpha subcomplex subunit 8 OS=Homo sapiens GN=NDUFA8 PE=1 SV=3 - [NDUA8_HUMAN]	4
NADH dehydrogenase [ubiquinone] 1 alpha subcomplex subunit 12 OS=Homo sapiens GN=NDUFA12 PE=1 SV=1 - [NDUAC_HUMAN]	4
NADH dehydrogenase [ubiquinone] iron-sulfur protein 7, mitochondrial OS=Homo sapiens GN=NDUFS7 PE=1 SV=3 - [NDUS7_HUMAN]	4
NADH dehydrogenase [ubiquinone] flavoprotein 2, mitochondrial OS=Homo sapiens GN=NDUFV2 PE=1 SV=2 - [NDUV2_HUMAN]	4
Na(+)/H(+) exchange regulatory cofactor NHE-RF1 OS=Homo sapiens GN=SLC9A3R1 PE=1 SV=4 - [NHRF1_HUMAN]	4
Nucleosome assembly protein 1-like 4 OS=Homo sapiens GN=NAP1L4 PE=1 SV=1 - [NP1L4_HUMAN]	4
Lipoamide acyltransferase component of branched-chain alpha-keto acid dehydrogenase complex, mitochondrial OS=Homo sapiens GN=DBT PE=1 SV=3 - [ODB2_HUMAN]	4
Proliferation-associated protein 2G4 OS=Homo sapiens GN=PA2G4 PE=1 SV=3 - [PA2G4_HUMAN]	4
Phosphofurin acidic cluster sorting protein 1 OS=Homo sapiens GN=PACS1 PE=1 SV=2 - [PACS1_HUMAN]	4
Isoform 6 of Poly(rC)-binding protein 2 OS=Homo sapiens GN=PCBP2 - [PCBP2_HUMAN]	4
[Pyruvate dehydrogenase (acetyl-transferring)] kinase isozyme 3, mitochondrial OS=Homo sapiens GN=PDK3 PE=1 SV=1 - [PDK3_HUMAN]	4

Astrocytic phosphoprotein PEA-15 OS=Homo sapiens GN=PEA15 PE=1 SV=2 - [PEA15_HUMAN]	4
Membrane-associated progesterone receptor component 1 OS=Homo sapiens GN=PGRMC1 PE=1 SV=3 - [PGRC1_HUMAN]	4
Peptidyl-prolyl cis-trans isomerase NIMA-interacting 1 OS=Homo sapiens GN=PIN1 PE=1 SV=1 - [PIN1_HUMAN]	4
Serine/threonine-protein phosphatase PP1-beta catalytic subunit OS=Homo sapiens GN=PPP1CB PE=1 SV=3 - [PP1B_HUMAN]	4
Serine/threonine-protein phosphatase 2A catalytic subunit beta isoform OS=Homo sapiens GN=PPP2CB PE=1 SV=1 - [PP2AB_HUMAN]	4
Prolargin OS=Homo sapiens GN=PRELP PE=1 SV=1 - [PRELP_HUMAN]	4
Isoform 2 of Major prion protein OS=Homo sapiens GN=PRNP - [PRIO_HUMAN]	4
Profilin-1 OS=Homo sapiens GN=PFN1 PE=1 SV=2 - [PROF1_HUMAN]	4
26S proteasome non-ATPase regulatory subunit 2 OS=Homo sapiens GN=PSMD2 PE=1 SV=3 - [PSMD2_HUMAN]	4
Transcriptional activator protein Pur-alpha OS=Homo sapiens GN=PURA PE=1 SV=2 - [PURA_HUMAN]	4
Ras-related protein Rab-1A OS=Homo sapiens GN=RAB1A PE=1 SV=3 - [RAB1A_HUMAN]	4
Ras-related protein Rab-1B OS=Homo sapiens GN=RAB1B PE=1 SV=1 - [RAB1B_HUMAN]	4
Ras-related protein Ral-A OS=Homo sapiens GN=RALA PE=1 SV=1 - [RALA_HUMAN]	4
Ras-related protein Rap-2a OS=Homo sapiens GN=RAP2A PE=1 SV=1 - [RAP2A_HUMAN]	4
Isoform 2B of GTPase KRas OS=Homo sapiens GN=KRAS - [RASK_HUMAN]	4
Ribonuclease inhibitor OS=Homo sapiens GN=RNH1 PE=1 SV=2 - [RINI_HUMAN]	4
Dolichyl-diphosphooligosaccharide--protein glycosyltransferase subunit 1 OS=Homo sapiens GN=RPN1 PE=1 SV=1 - [RPN1_HUMAN]	4
40S ribosomal protein S18 OS=Homo sapiens GN=RPS18 PE=1 SV=3 - [RS18_HUMAN]	4
40S ribosomal protein S4, X isoform OS=Homo sapiens GN=RPS4X PE=1 SV=2 - [RS4X_HUMAN]	4
40S ribosomal protein S7 OS=Homo sapiens GN=RPS7 PE=1 SV=1 - [RS7_HUMAN]	4
tRNA-splicing ligase RtcB homolog OS=Homo sapiens GN=RTCB PE=1 SV=1 - [RTCB_HUMAN]	4
Isoform RTN1-C of Reticulon-1 OS=Homo sapiens GN=RTN1 - [RTN1_HUMAN]	4
Isoform 3 of Reticulon-3 OS=Homo sapiens GN=RTN3 - [RTN3_HUMAN]	4
Isoform 3 of Reticulon-4 OS=Homo sapiens GN=RTN4 - [RTN4_HUMAN]	4
Isoform 3 of Selenium-binding protein 1 OS=Homo sapiens GN=SELENBP1 - [SBP1_HUMAN]	4
Protein SCAI OS=Homo sapiens GN=SCAI PE=1 SV=2 - [SCAI_HUMAN]	4
Saccharopine dehydrogenase-like oxidoreductase OS=Homo sapiens GN=SCCPDH PE=1 SV=1 - [SCPDL_HUMAN]	4
Isoform 3 of NCK-interacting protein with SH3 domain OS=Homo sapiens GN=NCKIPSD - [SPN90_HUMAN]	4
Succinyl-CoA ligase [ADP/GDP-forming] subunit alpha, mitochondrial OS=Homo sapiens GN=SUCLG1 PE=1 SV=4 - [SUCA_HUMAN]	4
Phenylalanine--tRNA ligase alpha subunit OS=Homo sapiens GN=FARSA PE=1 SV=3 - [SYFA_HUMAN]	4
Synaptotagmin-7 OS=Homo sapiens GN=SYT7 PE=1 SV=3 - [SYT7_HUMAN]	4
Tropomyosin beta chain OS=Homo sapiens GN=TPM2 PE=1 SV=1 - [TPM2_HUMAN]	4
Isoform 2 of 2,4-dienoyl-CoA reductase, mitochondrial OS=Homo sapiens GN=DECR1 - [DECR_HUMAN]	4
Isoform 4 of Prostaglandin E synthase 3 OS=Homo sapiens GN=PTGES3 - [TEBP_HUMAN]	4
Matrin-3 OS=Homo sapiens GN=MATR3 PE=1 SV=1 - [B3KM87_HUMAN]	4
Isoform 2 of ADP-ribosylation factor 3 OS=Homo sapiens GN=ARF3 - [ARF3_HUMAN]	4
Isoform 3 of Protein disulfide-isomerase A6 OS=Homo sapiens GN=PDIA6 - [PDIA6_HUMAN]	4
High mobility group protein B1 OS=Homo sapiens GN=HMGB1 PE=1 SV=1 - [Q5T7C4_HUMAN]	4
Calcium/calmodulin-dependent 3',5'-cyclic nucleotide phosphodiesterase 1B OS=Homo sapiens GN=PDE1B PE=1 SV=1 - [B4DK72_HUMAN]	4
Isoform 2 of Stomatin-like protein 2, mitochondrial OS=Homo sapiens GN=STOML2 - [STML2_HUMAN]	4
Cofilin 1 (Non-muscle), isoform CRA_a OS=Homo sapiens GN=CFL1 PE=1 SV=1 - [G3V1A4_HUMAN]	4
Erlin-2 (Fragment) OS=Homo sapiens GN=ERLIN2 PE=1 SV=1 - [E5RHW4_HUMAN]	4
Casein kinase II subunit alpha OS=Homo sapiens GN=CSNK2A1 PE=1 SV=1 - [E7EU96_HUMAN]	4
NADH dehydrogenase [ubiquinone] iron-sulfur protein 8, mitochondrial (Fragment) OS=Homo sapiens GN=NDUFS8 PE=1 SV=1 - [E9PN51_HUMAN]	4
40S ribosomal protein S8 OS=Homo sapiens GN=RPS8 PE=1 SV=1 - [Q5JR95_HUMAN]	4
Isoform 5 of MAGUK p55 subfamily member 2 OS=Homo sapiens GN=MPP2 - [MPP2_HUMAN]	4
Single-stranded DNA-binding protein, mitochondrial (Fragment) OS=Homo sapiens GN=SSBP1 PE=1 SV=1 - [C9K0U8_HUMAN]	4
26S protease regulatory subunit 6A (Fragment) OS=Homo sapiens GN=PSMC3 PE=1 SV=2 - [E9PKD5_HUMAN]	4
Filamin-A OS=Homo sapiens GN=FLNA PE=1 SV=1 - [Q5HY54_HUMAN]	4
Glutathione S-transferase Mu 1 OS=Homo sapiens GN=GSTM1 PE=1 SV=1 - [B9ZVX7_HUMAN]	4
Acid ceramidase OS=Homo sapiens GN=ASAH1 PE=1 SV=1 - [E7EMM4_HUMAN]	4
Heterogeneous nuclear ribonucleoprotein D0 (Fragment) OS=Homo sapiens GN=HNRNPD PE=1 SV=1 - [D6RAF8_HUMAN]	4
Polyubiquitin-C (Fragment) OS=Homo sapiens GN=UBC PE=1 SV=1 - [F5H265_HUMAN]	4

Serine/threonine-protein phosphatase OS=Homo sapiens GN=PPP1CC PE=1 SV=1 - [F8VYE8_HUMAN]	4
Phosphate carrier protein, mitochondrial OS=Homo sapiens GN=SLC25A3 PE=1 SV=1 - [F8VVM2_HUMAN]	4
60S acidic ribosomal protein P0 (Fragment) OS=Homo sapiens GN=RPLP0 PE=1 SV=1 - [F8VPE8_HUMAN]	4
CLIP-associating protein 2 OS=Homo sapiens GN=CLASP2 PE=1 SV=2 - [E3W994_HUMAN]	4
Cytochrome c (Fragment) OS=Homo sapiens GN=CYCS PE=1 SV=1 - [C9JFR7_HUMAN]	4
Nucleosome assembly protein 1-like 1 (Fragment) OS=Homo sapiens GN=NAP1L1 PE=1 SV=1 - [H0YHC3_HUMAN]	4
Fatty acid-binding protein, heart (Fragment) OS=Homo sapiens GN=FABP3 PE=1 SV=1 - [S4R371_HUMAN]	4
Apolipoprotein O (Fragment) OS=Homo sapiens GN=APOO PE=1 SV=1 - [H7C1U8_HUMAN]	4
Mitogen-activated protein kinase 10 (Fragment) OS=Homo sapiens GN=MAPK10 PE=1 SV=1 - [H0Y9H3_HUMAN]	4
Ganglioside-induced differentiation-associated protein 1-like 1 (Fragment) OS=Homo sapiens GN=GDAP1L1 PE=1 SV=1 - [H0Y6A7_HUMAN]	4
Aflatoxin B1 aldehyde reductase member 2 (Fragment) OS=Homo sapiens GN=AKR7A2 PE=1 SV=1 - [H3BLU7_HUMAN]	4
Pyruvate dehydrogenase protein X component, mitochondrial (Fragment) OS=Homo sapiens GN=PDHX PE=1 SV=1 - [H0YD97_HUMAN]	4
Hydroxyacylglutathione hydrolase, mitochondrial (Fragment) OS=Homo sapiens GN=HAGH PE=1 SV=1 - [H3BPK3_HUMAN]	4
NADH dehydrogenase [ubiquinone] 1 alpha subcomplex subunit 6 OS=Homo sapiens GN=NDUFA6 PE=4 SV=1 - [A0A0C4DGS0_HUMAN]	4
Heterogeneous nuclear ribonucleoprotein Q OS=Homo sapiens GN=SYNCRIP PE=1 SV=1 - [B7Z645_HUMAN]	4
ARP2 actin-related protein 2 homolog (Yeast), isoform CRA_d OS=Homo sapiens GN=ACTR2 PE=1 SV=2 - [F5H6T1_HUMAN]	4
40S ribosomal protein SA (Fragment) OS=Homo sapiens GN=RPSA PE=1 SV=4 - [C9J9K3_HUMAN]	4
Protein NDRG2 OS=Homo sapiens GN=NDRG2 PE=1 SV=1 - [A0A0A0MS87_HUMAN]	4
Rho GDP-dissociation inhibitor 1 (Fragment) OS=Homo sapiens GN=ARHGDIA PE=1 SV=4 - [J3KTF8_HUMAN]	4
Regulator of G-protein-signaling 7 OS=Homo sapiens GN=RGS7 PE=1 SV=1 - [A0A0A0MSE0_HUMAN]	4
Thy-1 membrane glycoprotein (Fragment) OS=Homo sapiens GN=THY1 PE=1 SV=4 - [E9PIM6_HUMAN]	4
Opalin OS=Homo sapiens GN=OPALIN PE=1 SV=1 - [A0A0A0MTN4_HUMAN]	4
Protein LOC102724023 OS=Homo sapiens GN=LOC102724023 PE=4 SV=1 - [A0A096LNH5_HUMAN]	4
Myristoylated alanine-rich C-kinase substrate OS=Homo sapiens GN=MARCKS PE=1 SV=1 - [A0A087WZH7_HUMAN]	4
Ras-related protein Rab-14 (Fragment) OS=Homo sapiens GN=RAB14 PE=1 SV=1 - [X6RFL8_HUMAN]	4
Hsc70-interacting protein OS=Homo sapiens GN=ST13 PE=1 SV=1 - [A0A087X1H6_HUMAN]	4
ATPase, H+ transporting, lysosomal accessory protein 1, isoform CRA_c OS=Homo sapiens GN=ATP6AP1 PE=4 SV=1 - [A0A0C4DGX8_HUMAN]	4
3-ketoacyl-CoA thiolase, mitochondrial OS=Homo sapiens GN=ACAA2 PE=1 SV=1 - [A0A0B4J2A4_HUMAN]	4
ARF GTPase-activating protein GIT1 OS=Homo sapiens GN=GIT1 PE=4 SV=1 - [A0A0C4DGN6_HUMAN]	4
Isoform 7 of Long-chain-fatty-acid--CoA ligase 6 OS=Homo sapiens GN=ACSL6 - [ACSL6_HUMAN]	3
Retinal dehydrogenase 1 OS=Homo sapiens GN=ALDH1A1 PE=1 SV=2 - [AL1A1_HUMAN]	3
Isoform 2 of Cytosol aminopeptidase OS=Homo sapiens GN=LAP3 - [AMPL_HUMAN]	3
Isoform 10 of Ankyrin repeat and sterile alpha motif domain-containing protein 1B OS=Homo sapiens GN=ANKS1B - [ANS1B_HUMAN]	3
Actin-related protein 2/3 complex subunit 3 OS=Homo sapiens GN=ARPC3 PE=1 SV=3 - [ARPC3_HUMAN]	3
Isoform 7 of Alpha-tubulin N-acetyltransferase 1 OS=Homo sapiens GN=ATAT1 - [ATAT_HUMAN]	3
Isoform 3 of ATPase family AAA domain-containing protein 3A OS=Homo sapiens GN=ATAD3A - [ATD3A_HUMAN]	3
ATP synthase subunit delta, mitochondrial OS=Homo sapiens GN=ATP5D PE=1 SV=2 - [ATPD_HUMAN]	3
Isoform 4 of Putative tyrosine-protein phosphatase auxilin OS=Homo sapiens GN=DNAJC6 - [AUXI_HUMAN]	3
Protein bassoon OS=Homo sapiens GN=BSN PE=2 SV=4 - [BSN_HUMAN]	3
Isoform 2 of Carnitine O-acetyltransferase OS=Homo sapiens GN=CRAT - [CACP_HUMAN]	3
Catalase OS=Homo sapiens GN=CAT PE=1 SV=3 - [CATA_HUMAN]	3
F-actin-capping protein subunit alpha-1 OS=Homo sapiens GN=CAPZA1 PE=1 SV=3 - [CAZA1_HUMAN]	3
F-actin-capping protein subunit alpha-2 OS=Homo sapiens GN=CAPZA2 PE=1 SV=3 - [CAZA2_HUMAN]	3
Isoform 2 of CD166 antigen OS=Homo sapiens GN=ALCAM - [CD166_HUMAN]	3
CDGSH iron-sulfur domain-containing protein 1 OS=Homo sapiens GN=CISD1 PE=1 SV=1 - [CISD1_HUMAN]	3
Chloride intracellular channel protein 4 OS=Homo sapiens GN=CLIC4 PE=1 SV=4 - [CLIC4_HUMAN]	3
Contactin-associated protein 1 OS=Homo sapiens GN=CNTNAP1 PE=1 SV=1 - [CNTP1_HUMAN]	3
Isoform 3 of Collagen alpha-3(VI) chain OS=Homo sapiens GN=COL6A3 - [CO6A3_HUMAN]	3
Copine-3 OS=Homo sapiens GN=CPNE3 PE=1 SV=1 - [CPNE3_HUMAN]	3
Isoform 3 of Choline transporter-like protein 2 OS=Homo sapiens GN=SLC44A2 - [CTL2_HUMAN]	3
Cytochrome c oxidase subunit 6B1 OS=Homo sapiens GN=COX6B1 PE=1 SV=2 - [CX6B1_HUMAN]	3
Isoform 3 of Calcyclin-binding protein OS=Homo sapiens GN=CACYBP - [CYBP_HUMAN]	3
Drebrin-like protein OS=Homo sapiens GN=DBNL PE=1 SV=1 - [DBNL_HUMAN]	3

Isoform 2 of ATP-dependent RNA helicase DDX3X OS=Homo sapiens GN=DDX3X - [DDX3X_HUMAN]	3
Isoform 3 of Disks large homolog 2 OS=Homo sapiens GN=DLG2 - [DLG2_HUMAN]	3
Isoform 2 of DnaJ homolog subfamily C member 5 OS=Homo sapiens GN=DNAJC5 - [DNJC5_HUMAN]	3
D-dopachrome decarboxylase OS=Homo sapiens GN=DDT PE=1 SV=3 - [DOPD_HUMAN]	3
Dihydropyrimidinase-related protein 4 OS=Homo sapiens GN=DPYSL4 PE=1 SV=2 - [DPYL4_HUMAN]	3
Dual specificity protein phosphatase 3 OS=Homo sapiens GN=DUSP3 PE=1 SV=1 - [DUS3_HUMAN]	3
Delta(3,5)-Delta(2,4)-dienoyl-CoA isomerase, mitochondrial OS=Homo sapiens GN=ECH1 PE=1 SV=2 - [ECH1_HUMAN]	3
Isoform 2 of Redox-regulatory protein FAM213A OS=Homo sapiens GN=FAM213A - [F213A_HUMAN]	3
Isoform 2 of 6-phosphofructo-2-kinase/fructose-2,6-bisphosphatase 2 OS=Homo sapiens GN=PFKFB2 - [F262_HUMAN]	3
Fatty acid synthase OS=Homo sapiens GN=FASN PE=1 SV=3 - [FAS_HUMAN]	3
Fibrinogen beta chain OS=Homo sapiens GN=FGB PE=1 SV=2 - [FIBB_HUMAN]	3
Peptidyl-prolyl cis-trans isomerase FKBP8 OS=Homo sapiens GN=FKBP8 PE=1 SV=2 - [FKBP8_HUMAN]	3
Isoform 2 of Growth factor receptor-bound protein 2 OS=Homo sapiens GN=GRB2 - [GRB2_HUMAN]	3
Glyoxylate reductase/hydroxypyruvate reductase OS=Homo sapiens GN=GRHPR PE=1 SV=1 - [GRHPR_HUMAN]	3
Histone H1x OS=Homo sapiens GN=H1FX PE=1 SV=1 - [H1X_HUMAN]	3
Isoform 2 of Histone H2A.V OS=Homo sapiens GN=H2AFV - [H2AV_HUMAN]	3
Isoform 1 of Core histone macro-H2A.1 OS=Homo sapiens GN=H2AFY - [H2AY_HUMAN]	3
Putative histone H2B type 2-D OS=Homo sapiens GN=HIST2H2BD PE=5 SV=3 - [H2B2D_HUMAN]	3
Isoform 2 of 3-hydroxyacyl-CoA dehydrogenase type-2 OS=Homo sapiens GN=HSD17B10 - [HCD2_HUMAN]	3
Hepatoma-derived growth factor-related protein 3 OS=Homo sapiens GN=HDGFRP3 PE=1 SV=1 - [HDGR3_HUMAN]	3
Hepatocyte cell adhesion molecule OS=Homo sapiens GN=HEPACAM PE=1 SV=1 - [HECAM_HUMAN]	3
Isoform 3 of Heterogeneous nuclear ribonucleoprotein H3 OS=Homo sapiens GN=HNRNPH3 - [HNRH3_HUMAN]	3
Isoform 2 of Heterogeneous nuclear ribonucleoprotein M OS=Homo sapiens GN=HNRNPM - [HNRPM_HUMAN]	3
Eukaryotic translation initiation factor 5 OS=Homo sapiens GN=EIF5 PE=1 SV=2 - [IF5_HUMAN]	3
Ig gamma-1 chain C region OS=Homo sapiens GN=IGHG1 PE=1 SV=1 - [IGHG1_HUMAN]	3
Isoform 2 of Importin-5 OS=Homo sapiens GN=IPO5 - [IPO5_HUMAN]	3
GTP:AMP phosphotransferase AK3, mitochondrial OS=Homo sapiens GN=AK3 PE=1 SV=4 - [KAD3_HUMAN]	3
BTB/POZ domain-containing protein KCTD16 OS=Homo sapiens GN=KCTD16 PE=2 SV=1 - [KCD16_HUMAN]	3
Kinesin heavy chain isoform 5C OS=Homo sapiens GN=KIF5C PE=1 SV=1 - [KIF5C_HUMAN]	3
Protein kinase C alpha type OS=Homo sapiens GN=PRKCA PE=1 SV=4 - [KPCA_HUMAN]	3
LanC-like protein 2 OS=Homo sapiens GN=LANCL2 PE=1 SV=1 - [LANC2_HUMAN]	3
Isoform 2 of Lactoylglutathione lyase OS=Homo sapiens GN=GLO1 - [LGUL_HUMAN]	3
Isoform 2 of Tyrosine-protein kinase Lyn OS=Homo sapiens GN=LYN - [LYN_HUMAN]	3
Microsomal glutathione S-transferase 3 OS=Homo sapiens GN=MGST3 PE=1 SV=1 - [MGST3_HUMAN]	3
Dual specificity mitogen-activated protein kinase kinase 4 OS=Homo sapiens GN=MAP2K4 PE=1 SV=1 - [MP2K4_HUMAN]	3
Isoform 2 of NAD kinase 2, mitochondrial OS=Homo sapiens GN=NADK2 - [NAKD2_HUMAN]	3
Neurocan core protein OS=Homo sapiens GN=NCAN PE=1 SV=3 - [NCAN_HUMAN]	3
Neutral cholesterol ester hydrolase 1 OS=Homo sapiens GN=NCEH1 PE=1 SV=3 - [NCEH1_HUMAN]	3
NADH dehydrogenase [ubiquinone] 1 alpha subcomplex subunit 13 OS=Homo sapiens GN=NDUFA13 PE=1 SV=3 - [NDUAD_HUMAN]	3
NADH dehydrogenase [ubiquinone] iron-sulfur protein 6, mitochondrial OS=Homo sapiens GN=NDUFS6 PE=1 SV=1 - [NDUS6_HUMAN]	3
Isoform Cytoplasmic of Cysteine desulfurase, mitochondrial OS=Homo sapiens GN=NFS1 - [NFS1_HUMAN]	3
Nucleolin OS=Homo sapiens GN=NCL PE=1 SV=3 - [NUCL_HUMAN]	3
Ornithine aminotransferase, mitochondrial OS=Homo sapiens GN=OAT PE=1 SV=1 - [OAT_HUMAN]	3
Isoform 2 of UDP-N-acetylglucosamine--peptide N-acetylglucosaminyltransferase 110 kDa subunit OS=Homo sapiens GN=OGT - [OGT1_HUMAN]	3
Obg-like ATPase 1 OS=Homo sapiens GN=OLA1 PE=1 SV=2 - [OLA1_HUMAN]	3
Oligodendrocyte-myelin glycoprotein OS=Homo sapiens GN=OMG PE=1 SV=2 - [OMGP_HUMAN]	3
Isoform 3 of Opioid-binding protein/cell adhesion molecule OS=Homo sapiens GN=OPCML - [OPCM_HUMAN]	3
Isoform 7 of Oxidation resistance protein 1 OS=Homo sapiens GN=OXR1 - [OXR1_HUMAN]	3
Poly [ADP-ribose] polymerase 1 OS=Homo sapiens GN=PARP1 PE=1 SV=4 - [PARP1_HUMAN]	3
Isoform 2 of Pre-B-cell leukemia transcription factor-interacting protein 1 OS=Homo sapiens GN=PBXIP1 - [PBIP1_HUMAN]	3
Poly(rC)-binding protein 1 OS=Homo sapiens GN=PCBP1 PE=1 SV=2 - [PCBP1_HUMAN]	3
Isoform 3 of Propionyl-CoA carboxylase alpha chain, mitochondrial OS=Homo sapiens GN=PCCA - [PCCA_HUMAN]	3
Protein disulfide-isomerase A4 OS=Homo sapiens GN=PDIA4 PE=1 SV=2 - [PDIA4_HUMAN]	3

Peflin OS=Homo sapiens GN=PEF1 PE=1 SV=1 - [PEF1_HUMAN]	3
Prostaglandin E synthase 2 OS=Homo sapiens GN=PTGES2 PE=1 SV=1 - [PGES2_HUMAN]	3
Membrane-associated progesterone receptor component 2 OS=Homo sapiens GN=PGRMC2 PE=1 SV=1 - [PGRC2_HUMAN]	3
Phytanoyl-CoA hydroxylase-interacting protein OS=Homo sapiens GN=PHYHIP PE=1 SV=1 - [PHYIP_HUMAN]	3
Phosphomevalonate kinase OS=Homo sapiens GN=PMVK PE=1 SV=3 - [PMVK_HUMAN]	3
Low molecular weight phosphotyrosine protein phosphatase OS=Homo sapiens GN=ACP1 PE=1 SV=3 - [PPAC_HUMAN]	3
Ribose-phosphate pyrophosphokinase 1 OS=Homo sapiens GN=PRPS1 PE=1 SV=2 - [PRPS1_HUMAN]	3
Isoform 2 of 26S protease regulatory subunit 6B OS=Homo sapiens GN=PSMC4 - [PRS6B_HUMAN]	3
26S proteasome non-ATPase regulatory subunit 11 OS=Homo sapiens GN=PSMD11 PE=1 SV=3 - [PSD11_HUMAN]	3
Isoform 3 of PC4 and SFRS1-interacting protein OS=Homo sapiens GN=PSIP1 - [PSIP1_HUMAN]	3
26S proteasome non-ATPase regulatory subunit 3 OS=Homo sapiens GN=PSMD3 PE=1 SV=2 - [PSMD3_HUMAN]	3
Isoform 2 of Paraspeckle component 1 OS=Homo sapiens GN=PSPC1 - [PSPC1_HUMAN]	3
Prostaglandin-H2 D-isomerase OS=Homo sapiens GN=PTGDS PE=1 SV=1 - [PTGDS_HUMAN]	3
Ras-related protein Rab-18 OS=Homo sapiens GN=RAB18 PE=1 SV=1 - [RAB18_HUMAN]	3
Ras-related protein Ral-B OS=Homo sapiens GN=RALB PE=1 SV=1 - [RALB_HUMAN]	3
GTP-binding nuclear protein Ran OS=Homo sapiens GN=RAN PE=1 SV=3 - [RAN_HUMAN]	3
Ras-related protein Rap-2b OS=Homo sapiens GN=RAP2B PE=1 SV=1 - [RAP2B_HUMAN]	3
Ras-related protein M-Ras OS=Homo sapiens GN=MRAS PE=1 SV=2 - [RASM_HUMAN]	3
Retinol dehydrogenase 11 OS=Homo sapiens GN=RDH11 PE=1 SV=2 - [RDH11_HUMAN]	3
Rho-related GTP-binding protein RhoG OS=Homo sapiens GN=RHOG PE=1 SV=1 - [RHOG_HUMAN]	3
60S ribosomal protein L7a OS=Homo sapiens GN=RPL7A PE=1 SV=2 - [RL7A_HUMAN]	3
Ras-related protein R-Ras OS=Homo sapiens GN=RRAS PE=1 SV=1 - [RRAS_HUMAN]	3
40S ribosomal protein S19 OS=Homo sapiens GN=RPS19 PE=1 SV=2 - [RS19_HUMAN]	3
RuvB-like 2 OS=Homo sapiens GN=RUVBL2 PE=1 SV=3 - [RUVB2_HUMAN]	3
Isoform 3 of Electrogenic sodium bicarbonate cotransporter 1 OS=Homo sapiens GN=SLC4A4 - [S4A4_HUMAN]	3
Serum amyloid P-component OS=Homo sapiens GN=APCS PE=1 SV=2 - [SAMP_HUMAN]	3
Prosaposin OS=Homo sapiens GN=PSAP PE=1 SV=2 - [SAP_HUMAN]	3
GTP-binding protein SAR1a OS=Homo sapiens GN=SAR1A PE=1 SV=1 - [SAR1A_HUMAN]	3
Neutral amino acid transporter A OS=Homo sapiens GN=SLC1A4 PE=1 SV=1 - [SATT_HUMAN]	3
Isoform 5 of Septin-4 OS=Homo sapiens GN=SEPT4 - [SEPT4_HUMAN]	3
Isoform Short of Splicing factor, proline- and glutamine-rich OS=Homo sapiens GN=SFPQ - [SFPQ_HUMAN]	3
SH3 domain-binding glutamic acid-rich-like protein OS=Homo sapiens GN=SH3BGRL PE=1 SV=1 - [SH3L1_HUMAN]	3
Synaptogyrin-3 OS=Homo sapiens GN=SYNGR3 PE=1 SV=2 - [SNG3_HUMAN]	3
Isoform 2 of Sorting nexin-3 OS=Homo sapiens GN=SNX3 - [SNX3_HUMAN]	3
Stathmin OS=Homo sapiens GN=STMN1 PE=1 SV=3 - [STMN1_HUMAN]	3
Isoform 2 of Syntaxin-7 OS=Homo sapiens GN=STX7 - [STX7_HUMAN]	3
Isoleucine--tRNA ligase, mitochondrial OS=Homo sapiens GN=IARS2 PE=1 SV=2 - [SYIM_HUMAN]	3
Synaptophysin OS=Homo sapiens GN=SYP PE=1 SV=3 - [SYPH_HUMAN]	3
Beta-synuclein OS=Homo sapiens GN=SNCB PE=1 SV=1 - [SYUB_HUMAN]	3
Transmembrane protein 65 OS=Homo sapiens GN=TMEM65 PE=1 SV=2 - [TMM65_HUMAN]	3
Protein disulfide-isomerase TMX3 OS=Homo sapiens GN=TMX3 PE=1 SV=2 - [TMX3_HUMAN]	3
Thioredoxin-related transmembrane protein 4 OS=Homo sapiens GN=TMX4 PE=1 SV=1 - [TMX4_HUMAN]	3
Isoform 3 of Transportin-1 OS=Homo sapiens GN=TNPO1 - [TNPO1_HUMAN]	3
Isoform 2 of Transportin-2 OS=Homo sapiens GN=TNPO2 - [TNPO2_HUMAN]	3
Isoform 2 of Tumor protein D52 OS=Homo sapiens GN=TPD52 - [TPD52_HUMAN]	3
Serotransferrin OS=Homo sapiens GN=TF PE=1 SV=3 - [TRFE_HUMAN]	3
Cytochrome b-c1 complex subunit Rieske, mitochondrial OS=Homo sapiens GN=UQCRFS1 PE=1 SV=2 - [UCRI_HUMAN]	3
General vesicular transport factor p115 OS=Homo sapiens GN=USO1 PE=1 SV=2 - [USO1_HUMAN]	3
Isoform 2 of WD repeat-containing protein 1 OS=Homo sapiens GN=WDR1 - [WDR1_HUMAN]	3
Exportin-1 OS=Homo sapiens GN=XPO1 PE=1 SV=1 - [XPO1_HUMAN]	3
Isoform 4 of Exportin-2 OS=Homo sapiens GN=CSE1L - [XPO2_HUMAN]	3
X-ray repair cross-complementing protein 5 OS=Homo sapiens GN=XRCC5 PE=1 SV=3 - [XRCC5_HUMAN]	3
Isoform 2 of Mycophenolic acid acyl-glucuronide esterase, mitochondrial OS=Homo sapiens GN=ABHD10 - [ABHDA_HUMAN]	3

Isoform 2 of S-adenosylmethionine synthase isoform type-2 OS=Homo sapiens GN=MAT2A - [METK2_HUMAN]	3
Isoform 3 of ATP-citrate synthase OS=Homo sapiens GN=ACLY - [ACLY_HUMAN]	3
HOMER1F OS=Homo sapiens GN=HOMER1 PE=1 SV=1 - [Q86YM6_HUMAN]	3
40S ribosomal protein S9 OS=Homo sapiens GN=RPS9 PE=1 SV=1 - [B5MCT8_HUMAN]	3
Transcription elongation factor B (SIII), polypeptide 2 (18kDa, elongin B), isoform CRA_b OS=Homo sapiens GN=TCEB2 PE=1 SV=1 - [B8ZZU8_HUMAN]	3
Acylglycerol kinase, mitochondrial OS=Homo sapiens GN=AGK PE=1 SV=1 - [E9PG39_HUMAN]	3
Ribosomal protein S6 kinase alpha-2 OS=Homo sapiens GN=RPS6KA2 PE=1 SV=1 - [B7Z3B5_HUMAN]	3
Isoform 2 of 26S protease regulatory subunit 4 OS=Homo sapiens GN=PSMC1 - [PRS4_HUMAN]	3
COP9 signalosome complex subunit 1 OS=Homo sapiens GN=GPS1 PE=1 SV=2 - [C9JFE4_HUMAN]	3
MAGUK p55 subfamily member 6 OS=Homo sapiens GN=MPP6 PE=1 SV=1 - [B8ZZG1_HUMAN]	3
Isoform 2 of 6-phosphogluconate dehydrogenase, decarboxylating OS=Homo sapiens GN=PGD - [6PGD_HUMAN]	3
Catenin delta-2 OS=Homo sapiens GN=CTNND2 PE=1 SV=1 - [B4DRK2_HUMAN]	3
26S proteasome non-ATPase regulatory subunit 13 (Fragment) OS=Homo sapiens GN=PSMD13 PE=1 SV=1 - [E9PL38_HUMAN]	3
DnaJ homolog subfamily B member 6 (Fragment) OS=Homo sapiens GN=DNAJB6 PE=1 SV=1 - [C9J2C4_HUMAN]	3
Isocitrate dehydrogenase [NAD] subunit, mitochondrial (Fragment) OS=Homo sapiens GN=IDH3G PE=3 SV=1 - [E9PF84_HUMAN]	3
Proteasome subunit alpha type OS=Homo sapiens GN=PSMA6 PE=1 SV=1 - [G3V3U4_HUMAN]	3
40S ribosomal protein S3a (Fragment) OS=Homo sapiens GN=RPS3A PE=1 SV=1 - [D6RG13_HUMAN]	3
Nascent polypeptide-associated complex subunit alpha OS=Homo sapiens GN=NACA PE=1 SV=1 - [F8VZJ2_HUMAN]	3
Ribosomal protein L15 (Fragment) OS=Homo sapiens GN=RPL15 PE=1 SV=1 - [E7EQV9_HUMAN]	3
Calcineurin subunit B type 1 OS=Homo sapiens GN=PPP3R1 PE=1 SV=1 - [F6U1T9_HUMAN]	3
Proline synthase co-transcribed bacterial homolog protein (Fragment) OS=Homo sapiens GN=PROSC PE=1 SV=1 - [E5RFX7_HUMAN]	3
Propionyl-CoA carboxylase beta chain, mitochondrial OS=Homo sapiens GN=PCCB PE=1 SV=1 - [E9PEC3_HUMAN]	3
Glutamate receptor 2 OS=Homo sapiens GN=GRIA2 PE=1 SV=1 - [F8W7L6_HUMAN]	3
Fibroblast growth factor (Fragment) OS=Homo sapiens GN=FGF12 PE=1 SV=1 - [C9JUK8_HUMAN]	3
Cysteine and glycine-rich protein 1 OS=Homo sapiens GN=CSRP1 PE=1 SV=1 - [E9PP21_HUMAN]	3
40S ribosomal protein S16 OS=Homo sapiens GN=RPS16 PE=1 SV=1 - [M0R3H0_HUMAN]	3
Heterochromatin protein 1-binding protein 3 (Fragment) OS=Homo sapiens GN=HP1BP3 PE=1 SV=1 - [B0QZK4_HUMAN]	3
40S ribosomal protein S2 (Fragment) OS=Homo sapiens GN=RPS2 PE=1 SV=1 - [H0YEN5_HUMAN]	3
Serine-threonine kinase receptor-associated protein (Fragment) OS=Homo sapiens GN=STRAP PE=1 SV=1 - [H0YH33_HUMAN]	3
Protein ARPC4-TTLL3 (Fragment) OS=Homo sapiens GN=ARPC4-TTLL3 PE=1 SV=1 - [H7C0A3_HUMAN]	3
Epidermal growth factor receptor substrate 15-like 1 OS=Homo sapiens GN=EPS15L1 PE=1 SV=1 - [M0R2S2_HUMAN]	3
Alpha-actinin-4 (Fragment) OS=Homo sapiens GN=ACTN4 PE=1 SV=1 - [K7EJH8_HUMAN]	3
D-beta-hydroxybutyrate dehydrogenase, mitochondrial (Fragment) OS=Homo sapiens GN=BDH1 PE=1 SV=1 - [H7C2W1_HUMAN]	3
60S ribosomal protein L18a (Fragment) OS=Homo sapiens GN=RPL18A PE=1 SV=1 - [M0R3D6_HUMAN]	3
Isoform 5 of Cell adhesion molecule 2 OS=Homo sapiens GN=CADM2 - [CADM2_HUMAN]	3
Basal cell adhesion molecule OS=Homo sapiens GN=BCAM PE=1 SV=1 - [A0A087WXM8_HUMAN]	3
Mitochondrial carrier homolog 1 OS=Homo sapiens GN=MTCH1 PE=1 SV=1 - [F6WFU6_HUMAN]	3
Cytoplasmic dynein 1 intermediate chain 1 OS=Homo sapiens GN=DYNC1I1 PE=1 SV=1 - [A0A0A0MTG2_HUMAN]	3
Phosphoinositide phospholipase C OS=Homo sapiens GN=PLCB1 PE=1 SV=1 - [A0A087WT80_HUMAN]	3
Phosphatidylinositol-binding clathrin assembly protein (Fragment) OS=Homo sapiens GN=PICALM PE=1 SV=4 - [E9PI56_HUMAN]	3
Neural cell adhesion molecule 2 OS=Homo sapiens GN=NCAM2 PE=1 SV=2 - [H9KV31_HUMAN]	3
Neuronal growth regulator 1 OS=Homo sapiens GN=NEGR1 PE=1 SV=1 - [F6X2W2_HUMAN]	3
Polyadenylate-binding protein OS=Homo sapiens GN=PABPC1 PE=1 SV=1 - [A0A087WTT1_HUMAN]	3
TAR DNA-binding protein 43 (Fragment) OS=Homo sapiens GN=TARDBP PE=1 SV=1 - [A0A087WXQ5_HUMAN]	3
Basigin (Fragment) OS=Homo sapiens GN=BSG PE=1 SV=1 - [A0A087X2B5_HUMAN]	3
GRIP1-associated protein 1 OS=Homo sapiens GN=GRIPAP1 PE=1 SV=1 - [A0A087WT45_HUMAN]	3
Methyl-CpG-binding protein 2 OS=Homo sapiens GN=MECP2 PE=1 SV=1 - [A0A087X1U4_HUMAN]	3
Glutathione S-transferase P (Fragment) OS=Homo sapiens GN=GSTP1 PE=1 SV=1 - [A0A087X2E9_HUMAN]	3
Nicotinamide phosphoribosyltransferase OS=Homo sapiens GN=NAMPT PE=4 SV=1 - [A0A0C4DFS8_HUMAN]	3
HLA class I histocompatibility antigen, A-11 alpha chain OS=Homo sapiens GN=HLA-A PE=1 SV=1 - [1A11_HUMAN]	2
HLA class I histocompatibility antigen, A-68 alpha chain OS=Homo sapiens GN=HLA-A PE=1 SV=4 - [1A68_HUMAN]	2
HLA class I histocompatibility antigen, B-58 alpha chain OS=Homo sapiens GN=HLA-B PE=1 SV=1 - [1B58_HUMAN]	2
3-hydroxyisobutyrate dehydrogenase, mitochondrial OS=Homo sapiens GN=HIBADH PE=1 SV=2 - [3HIDH_HUMAN]	2

Isoform 6 of Acyl-CoA-binding protein OS=Homo sapiens GN=DBI - [ACBP_HUMAN]	2
Disintegrin and metalloproteinase domain-containing protein 10 OS=Homo sapiens GN=ADAM10 PE=1 SV=1 - [ADA10_HUMAN]	2
AP-1 complex subunit gamma-1 OS=Homo sapiens GN=AP1G1 PE=1 SV=5 - [AP1G1_HUMAN]	2
Apolipoprotein A-I OS=Homo sapiens GN=APOA1 PE=1 SV=1 - [APOA1_HUMAN]	2
ADP-ribosylation factor-like protein 2 OS=Homo sapiens GN=ARL2 PE=1 SV=4 - [ARL2_HUMAN]	2
Isoform 2 of Actin-related protein 3B OS=Homo sapiens GN=ACTR3B - [ARP3B_HUMAN]	2
Caytaxin OS=Homo sapiens GN=ATCAY PE=1 SV=2 - [ATCAY_HUMAN]	2
Isoform 2 of Ubiquitin-like-conjugating enzyme ATG3 OS=Homo sapiens GN=ATG3 - [ATG3_HUMAN]	2
ATP synthase subunit e, mitochondrial OS=Homo sapiens GN=ATP5I PE=1 SV=2 - [ATP5I_HUMAN]	2
Isoform 4 of ATP synthase subunit f, mitochondrial OS=Homo sapiens GN=ATP5J2 - [ATPK_HUMAN]	2
Isoform 2 of Methylglutaconyl-CoA hydratase, mitochondrial OS=Homo sapiens GN=AUH - [AUHM_HUMAN]	2
Gamma-butyrobetaine dioxygenase OS=Homo sapiens GN=BBOX1 PE=1 SV=1 - [BODG_HUMAN]	2
Complement component 1 Q subcomponent-binding protein, mitochondrial OS=Homo sapiens GN=C1QBP PE=1 SV=1 - [C1QBP_HUMAN]	2
Cell adhesion molecule 4 OS=Homo sapiens GN=CADM4 PE=1 SV=1 - [CADM4_HUMAN]	2
Cell cycle exit and neuronal differentiation protein 1 OS=Homo sapiens GN=CEND1 PE=2 SV=1 - [CEND_HUMAN]	2
MICOS complex subunit MIC25 OS=Homo sapiens GN=CHCHD6 PE=1 SV=1 - [MIC25_HUMAN]	2
Charged multivesicular body protein 4b OS=Homo sapiens GN=CHMP4B PE=1 SV=1 - [CHM4B_HUMAN]	2
CDGSH iron-sulfur domain-containing protein 2 OS=Homo sapiens GN=CISD2 PE=1 SV=1 - [CISD2_HUMAN]	2
Cytoskeleton-associated protein 4 OS=Homo sapiens GN=CKAP4 PE=1 SV=2 - [CKAP4_HUMAN]	2
Isoform Non-brain of Clathrin light chain A OS=Homo sapiens GN=CLTA - [CLCA_HUMAN]	2
Claudin-11 OS=Homo sapiens GN=CLDN11 PE=1 SV=2 - [CLD11_HUMAN]	2
Isoform 2 of CAP-Gly domain-containing linker protein 2 OS=Homo sapiens GN=CLIP2 - [CLIP2_HUMAN]	2
Contactin-2 OS=Homo sapiens GN=CNTN2 PE=1 SV=1 - [CNTN2_HUMAN]	2
Bifunctional coenzyme A synthase OS=Homo sapiens GN=COASY PE=1 SV=4 - [COASY_HUMAN]	2
Copine-4 OS=Homo sapiens GN=CPNE4 PE=2 SV=1 - [CPNE4_HUMAN]	2
Isoform 2 of Lambda-crystallin homolog OS=Homo sapiens GN=CRYL1 - [CRYL1_HUMAN]	2
Casein kinase II subunit beta OS=Homo sapiens GN=CSNK2B PE=1 SV=1 - [CSK2B_HUMAN]	2
Isoform 2 of COP9 signalosome complex subunit 8 OS=Homo sapiens GN=COPS8 - [CSN8_HUMAN]	2
Estradiol 17-beta-dehydrogenase 8 OS=Homo sapiens GN=HSD17B8 PE=1 SV=2 - [DHB8_HUMAN]	2
Isoform 3 of DnaJ homolog subfamily C member 11 OS=Homo sapiens GN=DNAJC11 - [DJC11_HUMAN]	2
Isoform 3 of Disks large-associated protein 1 OS=Homo sapiens GN=DLGAP1 - [DLGP1_HUMAN]	2
Isoform 2 of DnaJ homolog subfamily A member 3, mitochondrial OS=Homo sapiens GN=DNAJA3 - [DNJA3_HUMAN]	2
Dihydropyrimidinase-related protein 5 OS=Homo sapiens GN=DPYSL5 PE=1 SV=1 - [DPYL5_HUMAN]	2
Dynein light chain 1, cytoplasmic OS=Homo sapiens GN=DYNLL1 PE=1 SV=1 - [DYL1_HUMAN]	2
Eukaryotic translation initiation factor 3 subunit F OS=Homo sapiens GN=EIF3F PE=1 SV=1 - [EIF3F_HUMAN]	2
ER membrane protein complex subunit 2 OS=Homo sapiens GN=EMC2 PE=1 SV=1 - [EMC2_HUMAN]	2
Isoform 3 of Epsin-1 OS=Homo sapiens GN=EPN1 - [EPN1_HUMAN]	2
ERO1-like protein alpha OS=Homo sapiens GN=ERO1L PE=1 SV=2 - [ERO1A_HUMAN]	2
Exocyst complex component 4 OS=Homo sapiens GN=EXOC4 PE=1 SV=1 - [EXOC4_HUMAN]	2
Protein FAM49A OS=Homo sapiens GN=FAM49A PE=1 SV=1 - [FA49A_HUMAN]	2
Protein FAM98B OS=Homo sapiens GN=FAM98B PE=1 SV=1 - [FA98B_HUMAN]	2
Acylpyruvase FAHD1, mitochondrial OS=Homo sapiens GN=FAHD1 PE=1 SV=2 - [FAHD1_HUMAN]	2
Mitochondrial fission 1 protein OS=Homo sapiens GN=FIS1 PE=1 SV=2 - [FIS1_HUMAN]	2
Peptidyl-prolyl cis-trans isomerase FKBP2 OS=Homo sapiens GN=FKBP2 PE=1 SV=2 - [FKBP2_HUMAN]	2
Peptidyl-prolyl cis-trans isomerase FKBP3 OS=Homo sapiens GN=FKBP3 PE=1 SV=1 - [FKBP3_HUMAN]	2
Peptidyl-prolyl cis-trans isomerase FKBP4 OS=Homo sapiens GN=FKBP4 PE=1 SV=3 - [FKBP4_HUMAN]	2
Fructosamine-3-kinase OS=Homo sapiens GN=FN3K PE=1 SV=1 - [FN3K_HUMAN]	2
DOMON domain-containing protein FRRS1L OS=Homo sapiens GN=FRRS1L PE=2 SV=2 - [FRS1L_HUMAN]	2
Isoform 2 of Growth arrest-specific protein 7 OS=Homo sapiens GN=GAS7 - [GAS7_HUMAN]	2
Glia maturation factor beta OS=Homo sapiens GN=GMFB PE=1 SV=2 - [GMFB_HUMAN]	2
Guanine nucleotide-binding protein subunit alpha-12 OS=Homo sapiens GN=GNA12 PE=1 SV=4 - [GNA12_HUMAN]	2
Histone H2A type 2-B OS=Homo sapiens GN=HIST2H2AB PE=1 SV=3 - [H2A2B_HUMAN]	2
Isoform 2 of Haloacid dehalogenase-like hydrolase domain-containing protein 2 OS=Homo sapiens GN=HDHD2 - [HDHD2_HUMAN]	2

Histidine triad nucleotide-binding protein 2, mitochondrial OS=Homo sapiens GN=HINT2 PE=1 SV=1 - [HINT2_HUMAN]	2
Histidine triad nucleotide-binding protein 3 OS=Homo sapiens GN=HINT3 PE=1 SV=1 - [HINT3_HUMAN]	2
Isoform Short of Heterogeneous nuclear ribonucleoprotein U OS=Homo sapiens GN=HNRNPU - [HNRPU_HUMAN]	2
Isoform Beta of Heat shock protein 105 kDa OS=Homo sapiens GN=HSPH1 - [HS105_HUMAN]	2
Immunoglobulin superfamily member 21 OS=Homo sapiens GN=IGSF21 PE=2 SV=1 - [IGS21_HUMAN]	2
Importin-7 OS=Homo sapiens GN=IPO7 PE=1 SV=1 - [IPO7_HUMAN]	2
Isoform 3 of Integrin alpha-V OS=Homo sapiens GN=ITGAV - [ITAV_HUMAN]	2
Isoform 2 of Keratin, type II cytoskeletal 73 OS=Homo sapiens GN=KRT73 - [K2C73_HUMAN]	2
BTB/POZ domain-containing protein KCTD12 OS=Homo sapiens GN=KCTD12 PE=1 SV=1 - [KCD12_HUMAN]	2
Laminin subunit beta-2 OS=Homo sapiens GN=LAMB2 PE=1 SV=2 - [LAMB2_HUMAN]	2
Lysosome-associated membrane glycoprotein 2 OS=Homo sapiens GN=LAMP2 PE=1 SV=2 - [LAMP2_HUMAN]	2
Phospholysine phosphohistidine inorganic pyrophosphate phosphatase OS=Homo sapiens GN=LHPP PE=1 SV=2 - [LHPP_HUMAN]	2
Leucine-rich repeat-containing protein 47 OS=Homo sapiens GN=LRRC47 PE=1 SV=1 - [LRC47_HUMAN]	2
Trans-2-enoyl-CoA reductase, mitochondrial OS=Homo sapiens GN=MECR PE=1 SV=2 - [MECR_HUMAN]	2
Isoform 2 of Lactadherin OS=Homo sapiens GN=MFGE8 - [MFGM_HUMAN]	2
Mitochondrial-processing peptidase subunit alpha OS=Homo sapiens GN=PMPCA PE=1 SV=2 - [MPPA_HUMAN]	2
Isoform 1A of Myotubularin-related protein 1 OS=Homo sapiens GN=MTMR1 - [MTMR1_HUMAN]	2
Cell surface glycoprotein MUC18 OS=Homo sapiens GN=MCAM PE=1 SV=2 - [MUC18_HUMAN]	2
NADPH--cytochrome P450 reductase OS=Homo sapiens GN=POR PE=1 SV=2 - [NCPR_HUMAN]	2
NADH dehydrogenase [ubiquinone] 1 beta subcomplex subunit 3 OS=Homo sapiens GN=NDUFB3 PE=1 SV=3 - [NDUB3_HUMAN]	2
Isoform 2 of NADH dehydrogenase [ubiquinone] 1 beta subcomplex subunit 4 OS=Homo sapiens GN=NDUFB4 - [NDUB4_HUMAN]	2
NADH dehydrogenase [ubiquinone] 1 beta subcomplex subunit 6 OS=Homo sapiens GN=NDUFB6 PE=1 SV=3 - [NDUB6_HUMAN]	2
NADH dehydrogenase [ubiquinone] iron-sulfur protein 4, mitochondrial OS=Homo sapiens GN=NDUFS4 PE=1 SV=1 - [NDUS4_HUMAN]	2
NADH dehydrogenase [ubiquinone] iron-sulfur protein 5 OS=Homo sapiens GN=NDUFS5 PE=1 SV=3 - [NDUS5_HUMAN]	2
Isoform 2 of NAD(P)H-hydrate epimerase OS=Homo sapiens GN=APOA1BP - [NNRE_HUMAN]	2
Isoform 3 of Nucleophosmin OS=Homo sapiens GN=NPM1 - [NPM_HUMAN]	2
Protein NipSnap homolog 3B OS=Homo sapiens GN=NIPSNAP3B PE=2 SV=1 - [NPS3B_HUMAN]	2
Neuronal pentraxin-1 OS=Homo sapiens GN=NPTX1 PE=2 SV=2 - [NPTX1_HUMAN]	2
Isoform 3 of Neurotrimin OS=Homo sapiens GN=NTM - [NTRI_HUMAN]	2
Isoform 3 of Optineurin OS=Homo sapiens GN=OPTN - [OPTN_HUMAN]	2
Isoform 2 of Serine/threonine-protein phosphatase PGAM5, mitochondrial OS=Homo sapiens GN=PGAM5 - [PGAM5_HUMAN]	2
Biglycan OS=Homo sapiens GN=BGN PE=1 SV=2 - [PGS1_HUMAN]	2
Isoform 2 of Phytanoyl-CoA hydroxylase-interacting protein-like OS=Homo sapiens GN=PHYHIPL - [PHIPL_HUMAN]	2
1-phosphatidylinositol 4,5-bisphosphate phosphodiesterase delta-1 OS=Homo sapiens GN=PLCD1 PE=1 SV=2 - [PLCD1_HUMAN]	2
1-phosphatidylinositol 4,5-bisphosphate phosphodiesterase delta-3 OS=Homo sapiens GN=PLCD3 PE=1 SV=3 - [PLCD3_HUMAN]	2
Pyridoxal phosphate phosphatase OS=Homo sapiens GN=PDXP PE=1 SV=2 - [PLPP_HUMAN]	2
PRA1 family protein 2 OS=Homo sapiens GN=PRAF2 PE=1 SV=1 - [PRAF2_HUMAN]	2
Isoform 2 of Proteasome subunit alpha type-3 OS=Homo sapiens GN=PSMA3 - [PSA3_HUMAN]	2
Proteasome subunit beta type-1 OS=Homo sapiens GN=PSMB1 PE=1 SV=2 - [PSB1_HUMAN]	2
Proteasome subunit beta type-4 OS=Homo sapiens GN=PSMB4 PE=1 SV=4 - [PSB4_HUMAN]	2
Ras-related protein Rab-21 OS=Homo sapiens GN=RAB21 PE=1 SV=3 - [RAB21_HUMAN]	2
GTPase NRas OS=Homo sapiens GN=NRAS PE=1 SV=1 - [RASN_HUMAN]	2
Rab3 GTPase-activating protein non-catalytic subunit OS=Homo sapiens GN=RAB3GAP2 PE=1 SV=1 - [RBGPR_HUMAN]	2
Receptor expression-enhancing protein 5 OS=Homo sapiens GN=REEP5 PE=1 SV=3 - [REEP5_HUMAN]	2
Rho-related GTP-binding protein RhoB OS=Homo sapiens GN=RHOB PE=1 SV=1 - [RHOB_HUMAN]	2
60S ribosomal protein L10a OS=Homo sapiens GN=RPL10A PE=1 SV=2 - [RL10A_HUMAN]	2
Isoform 2 of 60S ribosomal protein L11 OS=Homo sapiens GN=RPL11 - [RL11_HUMAN]	2
Isoform 2 of 60S ribosomal protein L13 OS=Homo sapiens GN=RPL13 - [RL13_HUMAN]	2
Heterogeneous nuclear ribonucleoprotein A0 OS=Homo sapiens GN=HNRNPA0 PE=1 SV=1 - [ROA0_HUMAN]	2
40S ribosomal protein S20 OS=Homo sapiens GN=RPS20 PE=1 SV=1 - [RS20_HUMAN]	2
Isoform 2 of 40S ribosomal protein S24 OS=Homo sapiens GN=RPS24 - [RS24_HUMAN]	2
Isoform 2 of RuvB-like 1 OS=Homo sapiens GN=RUVBL1 - [RUVB1_HUMAN]	2
Isoform 2 of Adenosylhomocysteinase OS=Homo sapiens GN=AHCY - [SAHH_HUMAN]	2

Isoform 6 of Calcium-binding mitochondrial carrier protein SCaMC-2 OS=Homo sapiens GN=SLC25A25 - [SCMC2_HUMAN]	2
Isoform 3 of Septin-10 OS=Homo sapiens GN=SEPT10 - [SEP10_HUMAN]	2
Small glutamine-rich tetratricopeptide repeat-containing protein alpha OS=Homo sapiens GN=SGTA PE=1 SV=1 - [SGTA_HUMAN]	2
Superoxide dismutase [Cu-Zn] OS=Homo sapiens GN=SOD1 PE=1 SV=2 - [SODC_HUMAN]	2
Isoform 2 of SLIT-ROBO Rho GTPase-activating protein 3 OS=Homo sapiens GN=SRGAP3 - [SRGP3_HUMAN]	2
Syntaxin-binding protein 3 OS=Homo sapiens GN=STXBP3 PE=1 SV=2 - [STXB3_HUMAN]	2
Translationally-controlled tumor protein OS=Homo sapiens GN=TPT1 PE=1 SV=1 - [TCTP_HUMAN]	2
Talin-2 OS=Homo sapiens GN=TLN2 PE=1 SV=4 - [TLN2_HUMAN]	2
Talin-1 OS=Homo sapiens GN=TLN1 PE=1 SV=3 - [TLN1_HUMAN]	2
Mitochondrial import receptor subunit TOM40 homolog OS=Homo sapiens GN=TOMM40 PE=1 SV=1 - [TOM40_HUMAN]	2
Isoform 6 of Tumor protein D54 OS=Homo sapiens GN=TPD52L2 - [TPD54_HUMAN]	2
Tripartite motif-containing protein 2 OS=Homo sapiens GN=TRIM2 PE=1 SV=1 - [TRIM2_HUMAN]	2
Isoform 4 of Tripartite motif-containing protein 3 OS=Homo sapiens GN=TRIM3 - [TRIM3_HUMAN]	2
Protein TSSC1 OS=Homo sapiens GN=TSSC1 PE=1 SV=2 - [TSSC1_HUMAN]	2
Isoform 4 of Protein tweety homolog 1 OS=Homo sapiens GN=TTYH1 - [TTYH1_HUMAN]	2
Ubiquitin carboxyl-terminal hydrolase 12 OS=Homo sapiens GN=USP12 PE=1 SV=2 - [UBP12_HUMAN]	2
Isoform 2 of UTP--glucose-1-phosphate uridylyltransferase OS=Homo sapiens GN=UGP2 - [UGPA_HUMAN]	2
V-type proton ATPase subunit G 2 OS=Homo sapiens GN=ATP6V1G2 PE=1 SV=1 - [VATG2_HUMAN]	2
Vacuolar protein sorting-associated protein 26B OS=Homo sapiens GN=VPS26B PE=1 SV=2 - [VP26B_HUMAN]	2
Isoform 2 of WD repeat-containing protein 47 OS=Homo sapiens GN=WDR47 - [WDR47_HUMAN]	2
Isoform 2 of 26S protease regulatory subunit 7 OS=Homo sapiens GN=PSMC2 - [PRS7_HUMAN]	2
Synaptogyrin-1 OS=Homo sapiens GN=SYNGR1 PE=1 SV=1 - [B5MCD7_HUMAN]	2
Isoform 3 of Uncharacterized protein KIAA0513 OS=Homo sapiens GN=KIAA0513 - [K0513_HUMAN]	2
Isoform 2 of Aldo-keto reductase family 1 member C3 OS=Homo sapiens GN=AKR1C3 - [AK1C3_HUMAN]	2
Profilin-2 OS=Homo sapiens GN=PFN2 PE=1 SV=1 - [C9J712_HUMAN]	2
Emerin OS=Homo sapiens GN=EMD PE=1 SV=1 - [Q5HY57_HUMAN]	2
Isoform 2 of Cytoplasmic dynein 1 light intermediate chain 2 OS=Homo sapiens GN=DYNC1LI2 - [DC1L2_HUMAN]	2
Histidine triad nucleotide-binding protein 1 OS=Homo sapiens GN=HINT1 PE=1 SV=1 - [D6RC06_HUMAN]	2
Actin-related protein 3 OS=Homo sapiens GN=ACTR3 PE=1 SV=1 - [B4DXW1_HUMAN]	2
Isoform 2 of EH domain-containing protein 2 OS=Homo sapiens GN=EHD2 - [EHD2_HUMAN]	2
Isoform 2 of F-box/LRR-repeat protein 16 OS=Homo sapiens GN=FBXL16 - [FXL16_HUMAN]	2
Isoform 3 of NADH dehydrogenase [ubiquinone] 1 beta subcomplex subunit 8, mitochondrial OS=Homo sapiens GN=NDUFB8 - [NDUB8_HUMAN]	2
Histidine--tRNA ligase, cytoplasmic OS=Homo sapiens GN=HARS PE=1 SV=1 - [B3KWE1_HUMAN]	2
DnaJ homolog subfamily B member 1 (Fragment) OS=Homo sapiens GN=DNAJB1 PE=1 SV=1 - [M0QYT3_HUMAN]	2
60S ribosomal protein L21 OS=Homo sapiens GN=RPL21 PE=1 SV=1 - [G3V1B3_HUMAN]	2
Glycogenin 1, isoform CRA_e OS=Homo sapiens GN=GYG1 PE=1 SV=1 - [G5E9W8_HUMAN]	2
60S ribosomal protein L23a OS=Homo sapiens GN=RPL23A PE=1 SV=1 - [K7EMA7_HUMAN]	2
Vesicle-associated membrane protein 2 OS=Homo sapiens GN=VAMP2 PE=4 SV=2 - [J3QRU4_HUMAN]	2
LanC-like protein 1 (Fragment) OS=Homo sapiens GN=LANCL1 PE=1 SV=1 - [E9PHS0_HUMAN]	2
Golgi-associated plant pathogenesis-related protein 1 OS=Homo sapiens GN=GLIPR2 PE=1 SV=1 - [Q5VZR0_HUMAN]	2
Cold shock domain-containing protein E1 OS=Homo sapiens GN=CSDE1 PE=1 SV=1 - [E9PLT0_HUMAN]	2
NADH dehydrogenase [ubiquinone] 1 beta subcomplex subunit 9 OS=Homo sapiens GN=NDUFB9 PE=1 SV=1 - [E9PH64_HUMAN]	2
Ras GTPase-activating protein-binding protein 2 (Fragment) OS=Homo sapiens GN=G3BP2 PE=1 SV=1 - [D6RAC7_HUMAN]	2
Inorganic pyrophosphatase OS=Homo sapiens GN=PPA1 PE=1 SV=1 - [Q5SQT6_HUMAN]	2
Calcium-binding protein 39-like OS=Homo sapiens GN=CAB39L PE=1 SV=1 - [B7ZBJ4_HUMAN]	2
Cyclin-dependent kinase 14 OS=Homo sapiens GN=CDK14 PE=1 SV=1 - [E7EUK8_HUMAN]	2
Pleckstrin homology domain-containing family B member 1 OS=Homo sapiens GN=PLEKHB1 PE=1 SV=1 - [F5GYU9_HUMAN]	2
Monoacylglycerol lipase ABHD12 (Fragment) OS=Homo sapiens GN=ABHD12 PE=1 SV=1 - [Q5T712_HUMAN]	2
Destrin OS=Homo sapiens GN=DSTN PE=1 SV=1 - [F6RFD5_HUMAN]	2
Epsin-3 (Fragment) OS=Homo sapiens GN=EPN3 PE=1 SV=1 - [D6RBR6_HUMAN]	2
Protein RPS10-NUDT3 (Fragment) OS=Homo sapiens GN=RPS10-NUDT3 PE=3 SV=1 - [S4R435_HUMAN]	2
Voltage-dependent L-type calcium channel subunit beta-3 (Fragment) OS=Homo sapiens GN=CACNB3 PE=4 SV=1 - [F8VU10_HUMAN]	2
LIM and calponin homology domains-containing protein 1 OS=Homo sapiens GN=LIMCH1 PE=1 SV=1 - [D6RD46_HUMAN]	2

Kinesin-like protein (Fragment) OS=Homo sapiens GN=KIF1A PE=1 SV=1 - [F8W8V9_HUMAN]	2
Disintegrin and metalloproteinase domain-containing protein 22 OS=Homo sapiens GN=ADAM22 PE=1 SV=1 - [E7EPF1_HUMAN]	2
OCIA domain-containing protein 1 (Fragment) OS=Homo sapiens GN=OCIAD1 PE=1 SV=1 - [D6RG39_HUMAN]	2
Transaldolase OS=Homo sapiens GN=TALDO1 PE=1 SV=1 - [F2Z393_HUMAN]	2
Isoform 3 of Stress-induced-phosphoprotein 1 OS=Homo sapiens GN=STIP1 - [STIP1_HUMAN]	2
Tetraspanin OS=Homo sapiens GN=CD9 PE=1 SV=1 - [A6NNI4_HUMAN]	2
Uncharacterized protein (Fragment) OS=Homo sapiens PE=1 SV=1 - [F8W031_HUMAN]	2
AP-3 complex subunit mu-2 (Fragment) OS=Homo sapiens GN=AP3M2 PE=1 SV=1 - [E5RJ52_HUMAN]	2
Guanine nucleotide-binding protein subunit beta-2-like 1 (Fragment) OS=Homo sapiens GN=GNB2L1 PE=1 SV=1 - [D6RFX4_HUMAN]	2
Glutathione S-transferase omega-1 (Fragment) OS=Homo sapiens GN=GSTO1 PE=1 SV=1 - [Q5TA02_HUMAN]	2
Sorting nexin-4 (Fragment) OS=Homo sapiens GN=SNX4 PE=1 SV=1 - [F8W9T3_HUMAN]	2
Serine/threonine-protein kinase OSR1 OS=Homo sapiens GN=OXSR1 PE=1 SV=1 - [C9JIG9_HUMAN]	2
Drebrin (Fragment) OS=Homo sapiens GN=DBN1 PE=1 SV=1 - [D6R9W4_HUMAN]	2
Protein transport protein Sec23A OS=Homo sapiens GN=SEC23A PE=1 SV=1 - [F5H365_HUMAN]	2
Transmembrane protein 33 (Fragment) OS=Homo sapiens GN=TMEM33 PE=1 SV=1 - [D6RAA6_HUMAN]	2
60S ribosomal protein L24 OS=Homo sapiens GN=RPL24 PE=1 SV=1 - [C9JXB8_HUMAN]	2
Isoform 2 of 60S ribosomal protein L18 OS=Homo sapiens GN=RPL18 - [RL18_HUMAN]	2
Ubiquitin carboxyl-terminal hydrolase OS=Homo sapiens GN=USP14 PE=1 SV=2 - [A6NJA2_HUMAN]	2
CD59 glycoprotein OS=Homo sapiens GN=CD59 PE=1 SV=1 - [E9PNW4_HUMAN]	2
Glutamate receptor 3 OS=Homo sapiens GN=GRIA3 PE=1 SV=1 - [A0A087WYJ6_HUMAN]	2
Heat shock 70 kDa protein 4L (Fragment) OS=Homo sapiens GN=HSPA4L PE=1 SV=1 - [D6RJ96_HUMAN]	2
WD repeat-containing protein 37 (Fragment) OS=Homo sapiens GN=WDR37 PE=1 SV=1 - [C9JGR9_HUMAN]	2
AP-1 complex subunit mu-1 OS=Homo sapiens GN=AP1M1 PE=1 SV=1 - [E7ENJ6_HUMAN]	2
TRAF2 and NCK-interacting protein kinase (Fragment) OS=Homo sapiens GN=TNIK PE=1 SV=1 - [C9J338_HUMAN]	2
Sickle tail protein homolog (Fragment) OS=Homo sapiens GN=KIAA1217 PE=1 SV=1 - [Q5T5P0_HUMAN]	2
Isoform 2 of Heme oxygenase 2 OS=Homo sapiens GN=HMOX2 - [HMOX2_HUMAN]	2
Ran GTPase-activating protein 1 (Fragment) OS=Homo sapiens GN=RANGAP1 PE=1 SV=1 - [H0Y4Q3_HUMAN]	2
Cytochrome c oxidase subunit 5A, mitochondrial OS=Homo sapiens GN=COX5A PE=1 SV=1 - [H3BRM5_HUMAN]	2
Ketimine reductase mu-crystallin (Fragment) OS=Homo sapiens GN=CRYM PE=1 SV=1 - [I3L325_HUMAN]	2
UBX domain-containing protein 6 (Fragment) OS=Homo sapiens GN=UBXN6 PE=1 SV=1 - [K7EP32_HUMAN]	2
cAMP-dependent protein kinase type II-alpha regulatory subunit (Fragment) OS=Homo sapiens GN=PRKAR2A PE=1 SV=1 - [H7C330_HUMAN]	2
Protein lin-7 homolog A (Fragment) OS=Homo sapiens GN=LIN7A PE=1 SV=1 - [H0YI92_HUMAN]	2
Protein rogdi homolog OS=Homo sapiens GN=ROGDI PE=1 SV=1 - [K7ERP1_HUMAN]	2
Isoform 2 of cAMP-dependent protein kinase type I-alpha regulatory subunit OS=Homo sapiens GN=PRKAR1A - [KAP0_HUMAN]	2
Grancalcin (Fragment) OS=Homo sapiens GN=GCA PE=1 SV=1 - [H7C2Z6_HUMAN]	2
Alanyl-tRNA-editing protein Aarsd1 (Fragment) OS=Homo sapiens GN=AARSD1 PE=1 SV=1 - [K7EQ85_HUMAN]	2
Serum paraoxonase/arylesterase 2 OS=Homo sapiens GN=PON2 PE=1 SV=1 - [J3QT77_HUMAN]	2
Inactive hydroxysteroid dehydrogenase-like protein 1 (Fragment) OS=Homo sapiens GN=HSDL1 PE=1 SV=1 - [H3BQI7_HUMAN]	2
Histone H3 OS=Homo sapiens GN=H3F3B PE=1 SV=1 - [K7EMV3_HUMAN]	2
Nucleoside diphosphate kinase OS=Homo sapiens GN=NME3 PE=1 SV=1 - [H3BPR2_HUMAN]	2
Haptoglobin (Fragment) OS=Homo sapiens GN=HP PE=1 SV=1 - [H3BS21_HUMAN]	2
Eukaryotic translation initiation factor 3 subunit E (Fragment) OS=Homo sapiens GN=EIF3E PE=1 SV=1 - [H0YAW4_HUMAN]	2
Gamma-aminobutyric acid receptor-associated protein-like 2 OS=Homo sapiens GN=GABARAPL2 PE=1 SV=1 - [H3BSM5_HUMAN]	2
Ras-related protein Rab-4B (Fragment) OS=Homo sapiens GN=RAB4B PE=1 SV=1 - [M0R1E0_HUMAN]	2
Neuronal membrane glycoprotein M6-b (Fragment) OS=Homo sapiens GN=GPM6B PE=1 SV=1 - [H7C5H4_HUMAN]	2
Collagen alpha-1(XVIII) chain (Fragment) OS=Homo sapiens GN=COL18A1 PE=1 SV=1 - [H7BXV5_HUMAN]	2
26S protease regulatory subunit 8 (Fragment) OS=Homo sapiens GN=PSMC5 PE=1 SV=1 - [J3QRW1_HUMAN]	2
UPF0556 protein C19orf10 (Fragment) OS=Homo sapiens GN=C19orf10 PE=1 SV=1 - [M0QXF7_HUMAN]	2
Protein NipSnap homolog 2 (Fragment) OS=Homo sapiens GN=GBAS PE=1 SV=1 - [H7C333_HUMAN]	2
Isoform 2 of Ran-specific GTPase-activating protein OS=Homo sapiens GN=RANBP1 - [RANG_HUMAN]	2
Ubiquitin-conjugating enzyme E2 variant 1 OS=Homo sapiens GN=UBE2V1 PE=4 SV=2 - [G3V2F7_HUMAN]	2
60S ribosomal protein L13a OS=Homo sapiens GN=RPL13a PE=1 SV=1 - [Q8J015_HUMAN]	2
Non-POU domain-containing octamer-binding protein (Fragment) OS=Homo sapiens GN=NONO PE=1 SV=4 - [C9JYS8_HUMAN]	2

Protein-tyrosine-phosphatase OS=Homo sapiens GN=PTPRS PE=1 SV=2 - [G8JL96_HUMAN]	2
Proteasome subunit beta type-2 OS=Homo sapiens GN=PSMB2 PE=1 SV=1 - [A0A087WVV1_HUMAN]	2
Proteasome subunit alpha type (Fragment) OS=Homo sapiens GN=PSMA4 PE=1 SV=4 - [H0YMZ1_HUMAN]	2
ATPase ASNA1 OS=Homo sapiens GN=ASNA1 PE=1 SV=1 - [A0A087WXS7_HUMAN]	2
Tubulin-folding cofactor B (Fragment) OS=Homo sapiens GN=TBCB PE=1 SV=4 - [K7EP07_HUMAN]	2
Uncharacterized protein (Fragment) OS=Homo sapiens PE=1 SV=3 - [I3L4J1_HUMAN]	2
Amino acid transporter OS=Homo sapiens GN=SLC1A3 PE=1 SV=1 - [A0A087X0U3_HUMAN]	2
60S ribosomal protein L9 (Fragment) OS=Homo sapiens GN=RPL9 PE=1 SV=4 - [D6RAN4_HUMAN]	2
L-xylulose reductase (Fragment) OS=Homo sapiens GN=DCXR PE=1 SV=4 - [J3KS22_HUMAN]	2
Kynurenine--oxoglutarate transaminase 3 (Fragment) OS=Homo sapiens GN=CCBL2 PE=1 SV=1 - [A0A0A0MRN7_HUMAN]	2
Glutathione peroxidase OS=Homo sapiens GN=GPX1 PE=1 SV=1 - [A0A087WUQ6_HUMAN]	2
Collagen alpha-1(VI) chain OS=Homo sapiens GN=COL6A1 PE=1 SV=1 - [A0A087X0S5_HUMAN]	2
Dynactin subunit 3 (Fragment) OS=Homo sapiens GN=DCTN3 PE=1 SV=1 - [X6RCK5_HUMAN]	2
Calcium-binding protein 39 OS=Homo sapiens GN=CAB39 PE=1 SV=1 - [A0A087X0K1_HUMAN]	2
Fibrinogen alpha chain OS=Homo sapiens GN=FGA PE=1 SV=1 - [A0A087WUA0_HUMAN]	2
Enolase-phosphatase E1 OS=Homo sapiens GN=ENOPH1 PE=4 SV=1 - [A0A0C4DGY8_HUMAN]	2

**Description AD1 P7**	**Peptides**
Isoform 7 of Plectin OS=Homo sapiens GN=PLEC - [PLEC_HUMAN]	107
Isoform 2 of Spectrin alpha chain, non-erythrocytic 1 OS=Homo sapiens GN=SPTAN1 - [SPTN1_HUMAN]	70
Cytoplasmic dynein 1 heavy chain 1 OS=Homo sapiens GN=DYNC1H1 PE=1 SV=5 - [DYHC1_HUMAN]	52
Spectrin beta chain, non-erythrocytic 1 OS=Homo sapiens GN=SPTBN1 PE=1 SV=2 - [SPTB2_HUMAN]	46
Isoform 3 of Dynamin-1 OS=Homo sapiens GN=DNM1 - [DYN1_HUMAN]	35
Isoform 4 of Dynamin-1 OS=Homo sapiens GN=DNM1 - [DYN1_HUMAN]	35
Glial fibrillary acidic protein OS=Homo sapiens GN=GFAP PE=1 SV=1 - [GFAP_HUMAN]	34
Vimentin OS=Homo sapiens GN=VIM PE=1 SV=4 - [VIME_HUMAN]	32
Vesicle-fusing ATPase OS=Homo sapiens GN=NSF PE=1 SV=1 - [I3L0N3_HUMAN]	29
Neurofilament medium polypeptide OS=Homo sapiens GN=NEFM PE=1 SV=1 - [E7EMV2_HUMAN]	27
Alpha-internexin OS=Homo sapiens GN=INA PE=1 SV=2 - [AINX_HUMAN]	26
Heat shock 70 kDa protein 12A OS=Homo sapiens GN=HSPA12A PE=1 SV=2 - [HS12A_HUMAN]	26
Keratin, type II cytoskeletal 2 epidermal OS=Homo sapiens GN=KRT2 PE=1 SV=2 - [K22E_HUMAN]	26
Isoform 2 of Dynamin-3 OS=Homo sapiens GN=DNM3 - [DYN3_HUMAN]	24
Syntaxin-binding protein 1 OS=Homo sapiens GN=STXBP1 PE=1 SV=1 - [STXB1_HUMAN]	24
Isoform 2 of Syntaxin-binding protein 1 OS=Homo sapiens GN=STXBP1 - [STXB1_HUMAN]	24
Isoform CNPI of 2',3'-cyclic-nucleotide 3'-phosphodiesterase OS=Homo sapiens GN=CNP - [CN37_HUMAN]	23
Keratin, type II cytoskeletal 6A OS=Homo sapiens GN=KRT6A PE=1 SV=3 - [K2C6A_HUMAN]	23
ATP-dependent 6-phosphofructokinase, platelet type OS=Homo sapiens GN=PFKP PE=1 SV=2 - [PFKAP_HUMAN]	23
Neurofilament light polypeptide OS=Homo sapiens GN=NEFL PE=1 SV=3 - [NFL_HUMAN]	23
Alanine--tRNA ligase, cytoplasmic OS=Homo sapiens GN=AARS PE=1 SV=2 - [SYAC_HUMAN]	23
Keratin, type II cytoskeletal 1 OS=Homo sapiens GN=KRT1 PE=1 SV=6 - [K2C1_HUMAN]	22
MICOS complex subunit MIC60 OS=Homo sapiens GN=IMMT PE=1 SV=2 - [B9A067_HUMAN]	22
Spectrin beta chain, non-erythrocytic 2 OS=Homo sapiens GN=SPTBN2 PE=1 SV=3 - [SPTN2_HUMAN]	21
Isoform B of AP-2 complex subunit alpha-1 OS=Homo sapiens GN=AP2A1 - [AP2A1_HUMAN]	20
Calcium-binding mitochondrial carrier protein Aralar1 OS=Homo sapiens GN=SLC25A12 PE=1 SV=2 - [CMC1_HUMAN]	20
Isoform 4 of Hexokinase-1 OS=Homo sapiens GN=HK1 - [HXK1_HUMAN]	20
Isoform 3 of Microtubule-associated protein 2 OS=Homo sapiens GN=MAP2 - [MTAP2_HUMAN]	20
Isoform 2 of Unconventional myosin-Va OS=Homo sapiens GN=MYO5A - [MYO5A_HUMAN]	20
Tubulin beta-2A chain OS=Homo sapiens GN=TUBB2A PE=1 SV=1 - [TBB2A_HUMAN]	20
Tubulin beta-2B chain OS=Homo sapiens GN=TUBB2B PE=1 SV=1 - [TBB2B_HUMAN]	20
Tubulin beta-4A chain OS=Homo sapiens GN=TUBB4A PE=1 SV=2 - [TBB4A_HUMAN]	20
Tubulin beta-4B chain OS=Homo sapiens GN=TUBB4B PE=1 SV=1 - [TBB4B_HUMAN]	20
Isoform 4 of Band 4.1-like protein 3 OS=Homo sapiens GN=EPB41L3 - [E41L3_HUMAN]	20
Isoform 2 of Ubiquitin-like modifier-activating enzyme 1 OS=Homo sapiens GN=UBA1 - [UBA1_HUMAN]	20
AP-2 complex subunit alpha-2 OS=Homo sapiens GN=AP2A2 PE=1 SV=2 - [AP2A2_HUMAN]	19

ATP synthase subunit alpha, mitochondrial OS=Homo sapiens GN=ATP5A1 PE=1 SV=1 - [ATPA_HUMAN]	19
Keratin, type I cytoskeletal 17 OS=Homo sapiens GN=KRT17 PE=1 SV=2 - [K1C17_HUMAN]	19
Keratin, type II cytoskeletal 5 OS=Homo sapiens GN=KRT5 PE=1 SV=3 - [K2C5_HUMAN]	19
Keratin, type II cytoskeletal 6B OS=Homo sapiens GN=KRT6B PE=1 SV=5 - [K2C6B_HUMAN]	19
Tubulin beta-3 chain OS=Homo sapiens GN=TUBB3 PE=1 SV=2 - [TBB3_HUMAN]	19
Ankyrin-2 OS=Homo sapiens GN=ANK2 PE=1 SV=1 - [I6L894_HUMAN]	19
C-1-tetrahydrofolate synthase, cytoplasmic OS=Homo sapiens GN=MTHFD1 PE=1 SV=3 - [C1TC_HUMAN]	18
Elongation factor Tu, mitochondrial OS=Homo sapiens GN=TUFM PE=1 SV=2 - [EFTU_HUMAN]	18
Keratin, type I cytoskeletal 10 OS=Homo sapiens GN=KRT10 PE=1 SV=6 - [K1C10_HUMAN]	18
Heat shock cognate 71 kDa protein OS=Homo sapiens GN=HSPA8 PE=1 SV=1 - [HSP7C_HUMAN]	17
Keratin, type I cytoskeletal 14 OS=Homo sapiens GN=KRT14 PE=1 SV=4 - [K1C14_HUMAN]	17
Leucine-rich PPR motif-containing protein, mitochondrial OS=Homo sapiens GN=LRPPRC PE=1 SV=3 - [LPPRC_HUMAN]	17
Microtubule-associated protein 1B OS=Homo sapiens GN=MAP1B PE=1 SV=2 - [MAP1B_HUMAN]	17
NADH-ubiquinone oxidoreductase 75 kDa subunit, mitochondrial OS=Homo sapiens GN=NDUFS1 PE=1 SV=3 - [NDUS1_HUMAN]	17
Dynamin-1-like protein OS=Homo sapiens GN=DNM1L PE=1 SV=1 - [G8JLD5_HUMAN]	17
Isoform 2 of Contactin-1 OS=Homo sapiens GN=CNTN1 - [CNTN1_HUMAN]	16
Glutaminase kidney isoform, mitochondrial OS=Homo sapiens GN=GLS PE=1 SV=1 - [GLSK_HUMAN]	16
Isoform C of Prelamin-A/C OS=Homo sapiens GN=LMNA - [LMNA_HUMAN]	16
Pyruvate carboxylase, mitochondrial OS=Homo sapiens GN=PC PE=1 SV=2 - [PYC_HUMAN]	16
Isoform 2 of Dihydropyrimidinase-related protein 2 OS=Homo sapiens GN=DPYSL2 - [DPYL2_HUMAN]	15
Guanine nucleotide-binding protein G(o) subunit alpha OS=Homo sapiens GN=GNAO1 PE=1 SV=4 - [GNAO_HUMAN]	15
Isoform 2 of Neurofilament heavy polypeptide OS=Homo sapiens GN=NEFH - [NFH_HUMAN]	15
Isoform 2 of 2-oxoglutarate dehydrogenase, mitochondrial OS=Homo sapiens GN=OGDH - [ODO1_HUMAN]	15
Isoform 1 of UDP-N-acetylglucosamine--peptide N-acetylglucosaminyltransferase 110 kDa subunit OS=Homo sapiens GN=OGT - [OGT1_HUMAN]	15
Isoform 2 of Tubulin alpha-1A chain OS=Homo sapiens GN=TUBA1A - [TBA1A_HUMAN]	15
Talin-2 OS=Homo sapiens GN=TLN2 PE=1 SV=4 - [TLN2_HUMAN]	15
Isoform Short of Ubiquitin carboxyl-terminal hydrolase 5 OS=Homo sapiens GN=USP5 - [UBP5_HUMAN]	15
Catenin beta-1 OS=Homo sapiens GN=CTNNB1 PE=1 SV=1 - [B4DGU4_HUMAN]	15
Isoform 2 of Tubulin alpha-4A chain OS=Homo sapiens GN=TUBA4A - [TBA4A_HUMAN]	15
Isoform 2 of Atlastin-1 OS=Homo sapiens GN=ATL1 - [ATLA1_HUMAN]	14
Elongation factor 2 OS=Homo sapiens GN=EEF2 PE=1 SV=4 - [EF2_HUMAN]	14
Isocitrate dehydrogenase [NADP], mitochondrial OS=Homo sapiens GN=IDH2 PE=1 SV=2 - [IDHP_HUMAN]	14
Keratin, type I cytoskeletal 16 OS=Homo sapiens GN=KRT16 PE=1 SV=4 - [K1C16_HUMAN]	14
Protein kinase C gamma type OS=Homo sapiens GN=PRKCG PE=1 SV=3 - [KPCG_HUMAN]	14
Heterogeneous nuclear ribonucleoproteins A2/B1 OS=Homo sapiens GN=HNRNPA2B1 PE=1 SV=2 - [ROA2_HUMAN]	14
Protein RUFY3 OS=Homo sapiens GN=RUFY3 PE=1 SV=1 - [RUFY3_HUMAN]	14
Isoform 2 of V-type proton ATPase catalytic subunit A OS=Homo sapiens GN=ATP6V1A - [VATA_HUMAN]	14
Actin, aortic smooth muscle OS=Homo sapiens GN=ACTA2 PE=1 SV=1 - [ACTA_HUMAN]	13
Amine oxidase [flavin-containing] B OS=Homo sapiens GN=MAOB PE=1 SV=3 - [AOFB_HUMAN]	13
ATP synthase subunit beta, mitochondrial OS=Homo sapiens GN=ATP5B PE=1 SV=3 - [ATPB_HUMAN]	13
ATP-dependent RNA helicase DDX1 OS=Homo sapiens GN=DDX1 PE=1 SV=2 - [DDX1_HUMAN]	13
Fibrinogen beta chain OS=Homo sapiens GN=FGB PE=1 SV=2 - [FIBB_HUMAN]	13
Heat shock-related 70 kDa protein 2 OS=Homo sapiens GN=HSPA2 PE=1 SV=1 - [HSP72_HUMAN]	13
ATP-dependent 6-phosphofructokinase, liver type OS=Homo sapiens GN=PFKL PE=1 SV=6 - [PFKAL_HUMAN]	13
Lamin-B2 OS=Homo sapiens GN=LMNB2 PE=1 SV=3 - [LMNB2_HUMAN]	13
Isoform 2 of Tubulin alpha-8 chain OS=Homo sapiens GN=TUBA8 - [TBA8_HUMAN]	13
Isoform 2 of T-complex protein 1 subunit beta OS=Homo sapiens GN=CCT2 - [TCPB_HUMAN]	13
T-complex protein 1 subunit gamma OS=Homo sapiens GN=CCT3 PE=1 SV=1 - [B4DUR8_HUMAN]	13
Calcium/calmodulin-dependent protein kinase (CaM kinase) II gamma, isoform CRA_n OS=Homo sapiens GN=CAMK2G PE=1 SV=1 - [Q5SWX3_HUMAN]	13
Synaptotagmin I, isoform CRA_b OS=Homo sapiens GN=SYT1 PE=1 SV=1 - [J3KQA0_HUMAN]	13
Isoform 2 of Methylmalonate-semialdehyde dehydrogenase [acylating], mitochondrial OS=Homo sapiens GN=ALDH6A1 - [MMSA_HUMAN]	13
Ras/Rap GTPase-activating protein SynGAP (Fragment) OS=Homo sapiens GN=SYNGAP1 PE=1 SV=2 - [B7ZCA0_HUMAN]	13
Serine/threonine-protein phosphatase 2A 65 kDa regulatory subunit A alpha isoform OS=Homo sapiens GN=PPP2R1A PE=1 SV=4 - [2AAA_HUMAN]	12

Actin, cytoplasmic 1 OS=Homo sapiens GN=ACTB PE=1 SV=1 - [ACTB_HUMAN]	12
Isoform 3 of Ankyrin-3 OS=Homo sapiens GN=ANK3 - [ANK3_HUMAN]	12
Isoform 2 of Annexin A6 OS=Homo sapiens GN=ANXA6 - [ANXA6_HUMAN]	12
Isoform 4 of Putative tyrosine-protein phosphatase auxilin OS=Homo sapiens GN=DNAJC6 - [AUXI_HUMAN]	12
Isoform 2 of Calcium-dependent secretion activator 1 OS=Homo sapiens GN=CADPS - [CAPS1_HUMAN]	12
Isoform 3 of Dynamin-2 OS=Homo sapiens GN=DNM2 - [DYN2_HUMAN]	12
EH domain-containing protein 3 OS=Homo sapiens GN=EHD3 PE=1 SV=2 - [EHD3_HUMAN]	12
Guanine nucleotide-binding protein G(z) subunit alpha OS=Homo sapiens GN=GNAZ PE=2 SV=3 - [GNAZ_HUMAN]	12
Creatine kinase B-type OS=Homo sapiens GN=CKB PE=1 SV=1 - [KCRB_HUMAN]	12
Microtubule-associated protein 1A OS=Homo sapiens GN=MAP1A PE=1 SV=6 - [MAP1A_HUMAN]	12
Isoform 2 of Nidogen-1 OS=Homo sapiens GN=NID1 - [NID1_HUMAN]	12
2-oxoglutarate dehydrogenase-like, mitochondrial OS=Homo sapiens GN=OGDHL PE=1 SV=3 - [OGDHL_HUMAN]	12
Protein disulfide-isomerase A3 OS=Homo sapiens GN=PDIA3 PE=1 SV=4 - [PDIA3_HUMAN]	12
Isoform 2 of Heterogeneous nuclear ribonucleoprotein A1 OS=Homo sapiens GN=HNRNPA1 - [ROA1_HUMAN]	12
Sorting and assembly machinery component 50 homolog OS=Homo sapiens GN=SAMM50 PE=1 SV=3 - [SAM50_HUMAN]	12
Cytoplasmic FMR1-interacting protein 2 OS=Homo sapiens GN=CYFIP2 PE=1 SV=1 - [E7EVJ5_HUMAN]	12
Sodium/potassium-transporting ATPase subunit alpha-3 OS=Homo sapiens GN=ATP1A3 PE=1 SV=1 - [M0R116_HUMAN]	12
Isoform 2 of Alpha-adducin OS=Homo sapiens GN=ADD1 - [ADDA_HUMAN]	11
Isoform 3 of Rho guanine nucleotide exchange factor 7 OS=Homo sapiens GN=ARHGEF7 - [ARHG7_HUMAN]	11
Isoform 3 of Brain-specific angiogenesis inhibitor 1-associated protein 2 OS=Homo sapiens GN=BAIAP2 - [BAIP2_HUMAN]	11
Isoform 2 of Catenin alpha-2 OS=Homo sapiens GN=CTNNA2 - [CTNA2_HUMAN]	11
Dihydropyrimidinase-related protein 1 OS=Homo sapiens GN=CRMP1 PE=1 SV=1 - [DPYL1_HUMAN]	11
Guanine nucleotide-binding protein G(i) subunit alpha-2 OS=Homo sapiens GN=GNAI2 PE=1 SV=3 - [GNAI2_HUMAN]	11
Stress-70 protein, mitochondrial OS=Homo sapiens GN=HSPA9 PE=1 SV=2 - [GRP75_HUMAN]	11
Glutathione S-transferase Mu 3 OS=Homo sapiens GN=GSTM3 PE=1 SV=3 - [GSTM3_HUMAN]	11
Heat shock protein HSP 90-alpha OS=Homo sapiens GN=HSP90AA1 PE=1 SV=5 - [HS90A_HUMAN]	11
Heat shock protein HSP 90-beta OS=Homo sapiens GN=HSP90AB1 PE=1 SV=4 - [HS90B_HUMAN]	11
Heat shock protein beta-1 OS=Homo sapiens GN=HSPB1 PE=1 SV=2 - [HSPB1_HUMAN]	11
Keratin, type I cytoskeletal 9 OS=Homo sapiens GN=KRT9 PE=1 SV=3 - [K1C9_HUMAN]	11
Isoform 3 of Calcium/calmodulin-dependent protein kinase type II subunit beta OS=Homo sapiens GN=CAMK2B - [KCC2B_HUMAN]	11
Laminin subunit beta-2 OS=Homo sapiens GN=LAMB2 PE=1 SV=2 - [LAMB2_HUMAN]	11
Dual specificity mitogen-activated protein kinase kinase 1 OS=Homo sapiens GN=MAP2K1 PE=1 SV=2 - [MP2K1_HUMAN]	11
Isoform 2 of Neurochondrin OS=Homo sapiens GN=NCDN - [NCDN_HUMAN]	11
Isoform 2 of 26S protease regulatory subunit 8 OS=Homo sapiens GN=PSMC5 - [PRS8_HUMAN]	11
tRNA-splicing ligase RtcB homolog OS=Homo sapiens GN=RTCB PE=1 SV=1 - [RTCB_HUMAN]	11
Isoform 2 of Succinyl-CoA ligase [ADP-forming] subunit beta, mitochondrial OS=Homo sapiens GN=SUCLA2 - [SUCB1_HUMAN]	11
T-complex protein 1 subunit delta OS=Homo sapiens GN=CCT4 PE=1 SV=4 - [TCPD_HUMAN]	11
Sodium/potassium-transporting ATPase subunit alpha-2 OS=Homo sapiens GN=ATP1A2 PE=1 SV=1 - [B1AKY9_HUMAN]	11
Mitochondrial 2-oxoglutarate/malate carrier protein (Fragment) OS=Homo sapiens GN=SLC25A11 PE=1 SV=1 - [I3L1P8_HUMAN]	11
Collagen alpha-3(VI) chain OS=Homo sapiens GN=COL6A3 PE=1 SV=2 - [E7ENL6_HUMAN]	11
14-3-3 protein eta OS=Homo sapiens GN=YWHAH PE=1 SV=4 - [1433F_HUMAN]	10
14-3-3 protein theta OS=Homo sapiens GN=YWHAQ PE=1 SV=1 - [1433T_HUMAN]	10
14-3-3 protein zeta/delta OS=Homo sapiens GN=YWHAZ PE=1 SV=1 - [1433Z_HUMAN]	10
ADP/ATP translocase 1 OS=Homo sapiens GN=SLC25A4 PE=1 SV=4 - [ADT1_HUMAN]	10
ADP/ATP translocase 2 OS=Homo sapiens GN=SLC25A5 PE=1 SV=7 - [ADT2_HUMAN]	10
ADP/ATP translocase 3 OS=Homo sapiens GN=SLC25A6 PE=1 SV=4 - [ADT3_HUMAN]	10
Carbonyl reductase [NADPH] 1 OS=Homo sapiens GN=CBR1 PE=1 SV=3 - [CBR1_HUMAN]	10
Elongation factor 1-alpha 1 OS=Homo sapiens GN=EEF1A1 PE=1 SV=1 - [EF1A1_HUMAN]	10
Fatty acid synthase OS=Homo sapiens GN=FASN PE=1 SV=3 - [FAS_HUMAN]	10
Isoform 2 of Heterogeneous nuclear ribonucleoprotein M OS=Homo sapiens GN=HNRNPM - [HNRPM_HUMAN]	10
Protein KIAA1045 OS=Homo sapiens GN=KIAA1045 PE=1 SV=2 - [K1045_HUMAN]	10
Calcium/calmodulin-dependent protein kinase type II subunit alpha OS=Homo sapiens GN=CAMK2A PE=1 SV=2 - [KCC2A_HUMAN]	10
Isoform Delta 12 of Calcium/calmodulin-dependent protein kinase type II subunit delta OS=Homo sapiens GN=CAMK2D - [KCC2D_HUMAN]	10

Phytanoyl-CoA hydroxylase-interacting protein OS=Homo sapiens GN=PHYHIP PE=1 SV=1 - [PHYIP_HUMAN]	10
26S protease regulatory subunit 10B OS=Homo sapiens GN=PSMC6 PE=1 SV=1 - [PRS10_HUMAN]	10
D-3-phosphoglycerate dehydrogenase OS=Homo sapiens GN=PHGDH PE=1 SV=4 - [SERA_HUMAN]	10
Beta-soluble NSF attachment protein OS=Homo sapiens GN=NAPB PE=1 SV=2 - [SNAB_HUMAN]	10
Isoform 3 of NCK-interacting protein with SH3 domain OS=Homo sapiens GN=NCKIPSD - [SPN90_HUMAN]	10
Visinin-like protein 1 OS=Homo sapiens GN=VSNL1 PE=1 SV=2 - [VISL1_HUMAN]	10
PH and SEC7 domain-containing protein 3 OS=Homo sapiens GN=PSD3 PE=1 SV=1 - [B4DKF8_HUMAN]	10
Casein kinase II subunit alpha OS=Homo sapiens GN=CSNK2A1 PE=1 SV=1 - [E7EU96_HUMAN]	10
Versican core protein OS=Homo sapiens GN=VCAN PE=1 SV=2 - [E9PF17_HUMAN]	10
Protein transport protein Sec23A OS=Homo sapiens GN=SEC23A PE=1 SV=1 - [F5H365_HUMAN]	10
Synapsin-2 OS=Homo sapiens GN=SYN2 PE=1 SV=1 - [A0A087X2E3_HUMAN]	10
14-3-3 protein epsilon OS=Homo sapiens GN=YWHAE PE=1 SV=1 - [1433E_HUMAN]	9
14-3-3 protein gamma OS=Homo sapiens GN=YWHAG PE=1 SV=2 - [1433G_HUMAN]	9
Aconitate hydratase, mitochondrial OS=Homo sapiens GN=ACO2 PE=1 SV=2 - [ACON_HUMAN]	9
Alpha-actinin-2 OS=Homo sapiens GN=ACTN2 PE=1 SV=1 - [ACTN2_HUMAN]	9
Isoform 3 of Ankyrin repeat and sterile alpha motif domain-containing protein 1B OS=Homo sapiens GN=ANKS1B - [ANS1B_HUMAN]	9
Annexin A2 OS=Homo sapiens GN=ANXA2 PE=1 SV=2 - [ANXA2_HUMAN]	9
Isoform 2 of Annexin A7 OS=Homo sapiens GN=ANXA7 - [ANXA7_HUMAN]	9
Isoform 3 of Sodium/potassium-transporting ATPase subunit alpha-1 OS=Homo sapiens GN=ATP1A1 - [AT1A1_HUMAN]	9
Isoform 4 of Clusterin OS=Homo sapiens GN=CLU - [CLUS_HUMAN]	9
Contactin-associated protein 1 OS=Homo sapiens GN=CNTNAP1 PE=1 SV=1 - [CNTP1_HUMAN]	9
Copine-6 OS=Homo sapiens GN=CPNE6 PE=1 SV=3 - [CPNE6_HUMAN]	9
Isoform 3 of Disks large homolog 4 OS=Homo sapiens GN=DLG4 - [DLG4_HUMAN]	9
Trifunctional enzyme subunit alpha, mitochondrial OS=Homo sapiens GN=HADHA PE=1 SV=2 - [ECHA_HUMAN]	9
Isoform 2 of Gelsolin OS=Homo sapiens GN=GSN - [GELS_HUMAN]	9
Isoform 2 of Heat shock 70 kDa protein 1A/1B OS=Homo sapiens GN=HSPA1A - [HSP71_HUMAN]	9
ATP-dependent 6-phosphofructokinase, muscle type OS=Homo sapiens GN=PFKM PE=1 SV=2 - [PFKAM_HUMAN]	9
Adenylate kinase 4, mitochondrial OS=Homo sapiens GN=AK4 PE=1 SV=1 - [KAD4_HUMAN]	9
Isoform 2 of Liprin-alpha-3 OS=Homo sapiens GN=PPFIA3 - [LIPA3_HUMAN]	9
Mitogen-activated protein kinase 3 OS=Homo sapiens GN=MAPK3 PE=1 SV=4 - [MK03_HUMAN]	9
Dual specificity mitogen-activated protein kinase kinase 2 OS=Homo sapiens GN=MAP2K2 PE=1 SV=1 - [MP2K2_HUMAN]	9
NADH dehydrogenase [ubiquinone] iron-sulfur protein 3, mitochondrial OS=Homo sapiens GN=NDUFS3 PE=1 SV=1 - [NDUS3_HUMAN]	9
Pyridoxal kinase OS=Homo sapiens GN=PDXK PE=1 SV=1 - [PDXK_HUMAN]	9
Glycogen phosphorylase, brain form OS=Homo sapiens GN=PYGB PE=1 SV=5 - [PYGB_HUMAN]	9
Isoform 2 of Rap1 GTPase-activating protein 1 OS=Homo sapiens GN=RAP1GAP - [RPGP1_HUMAN]	9
Alpha-soluble NSF attachment protein OS=Homo sapiens GN=NAPA PE=1 SV=3 - [SNAA_HUMAN]	9
Isoform 4 of SRC kinase signaling inhibitor 1 OS=Homo sapiens GN=SRCIN1 - [SRCN1_HUMAN]	9
Phenylalanine--tRNA ligase alpha subunit OS=Homo sapiens GN=FARSA PE=1 SV=3 - [SYFA_HUMAN]	9
Phenylalanine--tRNA ligase beta subunit OS=Homo sapiens GN=FARSB PE=1 SV=3 - [SYFB_HUMAN]	9
Isoform IB of Synapsin-1 OS=Homo sapiens GN=SYN1 - [SYN1_HUMAN]	9
T-complex protein 1 subunit theta OS=Homo sapiens GN=CCT8 PE=1 SV=4 - [TCPQ_HUMAN]	9
V-type proton ATPase subunit B, brain isoform OS=Homo sapiens GN=ATP6V1B2 PE=1 SV=3 - [VATB2_HUMAN]	9
Protein kinase C and casein kinase substrate in neurons protein 1 OS=Homo sapiens GN=PACSIN1 PE=1 SV=1 - [F6U236_HUMAN]	9
Glucose-6-phosphate isomerase OS=Homo sapiens GN=GPI PE=1 SV=2 - [K7EQ48_HUMAN]	9
Endophilin-B2 OS=Homo sapiens GN=SH3GLB2 PE=1 SV=1 - [B7ZC39_HUMAN]	9
EH domain-containing protein 1 (Fragment) OS=Homo sapiens GN=EHD1 PE=1 SV=1 - [C9JC03_HUMAN]	9
Ubiquitin thioesterase OTUB1 OS=Homo sapiens GN=OTUB1 PE=1 SV=1 - [F5GYN4_HUMAN]	9
Tubulointerstitial nephritis antigen-like OS=Homo sapiens GN=TINAGL1 PE=1 SV=1 - [F6SDV2_HUMAN]	9
Isoform Short of 14-3-3 protein beta/alpha OS=Homo sapiens GN=YWHAB - [1433B_HUMAN]	8
Isoform 2 of Long-chain-fatty-acid--CoA ligase 6 OS=Homo sapiens GN=ACSL6 - [ACSL6_HUMAN]	8
Cyclin-dependent-like kinase 5 OS=Homo sapiens GN=CDK5 PE=1 SV=3 - [CDK5_HUMAN]	8
Collagen alpha-2(VI) chain OS=Homo sapiens GN=COL6A2 PE=1 SV=4 - [CO6A2_HUMAN]	8
Bifunctional coenzyme A synthase OS=Homo sapiens GN=COASY PE=1 SV=4 - [COASY_HUMAN]	8

Isoform 1 of Serine/threonine-protein kinase DCLK1 OS=Homo sapiens GN=DCLK1 - [DCLK1_HUMAN]	8
Isoform 3 of Band 4.1-like protein 2 OS=Homo sapiens GN=EPB41L2 - [E41L2_HUMAN]	8
Elongation factor 1-alpha 2 OS=Homo sapiens GN=EEF1A2 PE=1 SV=1 - [EF1A2_HUMAN]	8
Guanine nucleotide-binding protein subunit alpha-13 OS=Homo sapiens GN=GNA13 PE=1 SV=2 - [GNA13_HUMAN]	8
Guanine nucleotide-binding protein G(q) subunit alpha OS=Homo sapiens GN=GNAQ PE=1 SV=4 - [GNAQ_HUMAN]	8
Isoform 3 of Guanine nucleotide-binding protein G(s) subunit alpha isoforms short OS=Homo sapiens GN=GNAS - [GNAS2_HUMAN]	8
Glutamate receptor 2 OS=Homo sapiens GN=GRIA2 PE=1 SV=3 - [GRIA2_HUMAN]	8
Histone H4 OS=Homo sapiens GN=HIST1H4A PE=1 SV=2 - [H4_HUMAN]	8
Isoform 3 of Heterogeneous nuclear ribonucleoprotein K OS=Homo sapiens GN=HNRNPK - [HNRPK_HUMAN]	8
Keratin, type II cytoskeletal 79 OS=Homo sapiens GN=KRT79 PE=1 SV=2 - [K2C79_HUMAN]	8
Isoform 2 of Kinesin-like protein KIF2A OS=Homo sapiens GN=KIF2A - [KIF2A_HUMAN]	8
Microtubule-associated protein RP/EB family member 3 OS=Homo sapiens GN=MAPRE3 PE=1 SV=1 - [MARE3_HUMAN]	8
Isoform 2 of Mitogen-activated protein kinase 1 OS=Homo sapiens GN=MAPK1 - [MK01_HUMAN]	8
Nicotinamide phosphoribosyltransferase OS=Homo sapiens GN=NAMPT PE=1 SV=1 - [NAMPT_HUMAN]	8
Isoform 2 of Pyruvate dehydrogenase E1 component subunit beta, mitochondrial OS=Homo sapiens GN=PDHB - [ODPB_HUMAN]	8
Isoform 2 of Optineurin OS=Homo sapiens GN=OPTN - [OPTN_HUMAN]	8
Protein-arginine deiminase type-2 OS=Homo sapiens GN=PADI2 PE=2 SV=2 - [PADI2_HUMAN]	8
Basement membrane-specific heparan sulfate proteoglycan core protein OS=Homo sapiens GN=HSPG2 PE=1 SV=4 - [PGBM_HUMAN]	8
Serine/threonine-protein phosphatase PP1-alpha catalytic subunit OS=Homo sapiens GN=PPP1CA PE=1 SV=1 - [PP1A_HUMAN]	8
26S proteasome non-ATPase regulatory subunit 11 OS=Homo sapiens GN=PSMD11 PE=1 SV=3 - [PSD11_HUMAN]	8
Cytochrome b-c1 complex subunit 2, mitochondrial OS=Homo sapiens GN=UQCRC2 PE=1 SV=3 - [QCR2_HUMAN]	8
Ubiquitin carboxyl-terminal hydrolase isozyme L1 OS=Homo sapiens GN=UCHL1 PE=1 SV=2 - [UCHL1_HUMAN]	8
Voltage-dependent anion-selective channel protein 3 OS=Homo sapiens GN=VDAC3 PE=1 SV=1 - [VDAC3_HUMAN]	8
Adenylyl cyclase-associated protein OS=Homo sapiens GN=CAP2 PE=1 SV=1 - [E9PDI2_HUMAN]	8
ATP synthase F(0) complex subunit B1, mitochondrial OS=Homo sapiens GN=ATP5F1 PE=1 SV=1 - [Q5QNZ2_HUMAN]	8
Alpha-crystallin B chain (Fragment) OS=Homo sapiens GN=CRYAB PE=1 SV=1 - [E9PR44_HUMAN]	8
Prohibitin-2 OS=Homo sapiens GN=PHB2 PE=1 SV=1 - [F5GY37_HUMAN]	8
Serine/threonine-protein phosphatase OS=Homo sapiens GN=PPP1CC PE=1 SV=1 - [F8VYE8_HUMAN]	8
Myelin basic protein OS=Homo sapiens GN=MBP PE=1 SV=1 - [J3QL64_HUMAN]	8
MAGUK p55 subfamily member 2 OS=Homo sapiens GN=MPP2 PE=1 SV=1 - [B4DZ84_HUMAN]	8
Neural cell adhesion molecule 1 OS=Homo sapiens GN=NCAM1 PE=1 SV=2 - [H7BYX6_HUMAN]	8
NADH dehydrogenase [ubiquinone] 1 alpha subcomplex subunit 10, mitochondrial OS=Homo sapiens GN=NDUFA10 PE=1 SV=1 - [A0A087WXC5_HUMAN]	8
ARF GTPase-activating protein GIT1 OS=Homo sapiens GN=GIT1 PE=4 SV=1 - [A0A0C4DGN6_HUMAN]	8
Isoform 2 of Alpha-actinin-1 OS=Homo sapiens GN=ACTN1 - [ACTN1_HUMAN]	7
Isoform 2 of AP-2 complex subunit mu OS=Homo sapiens GN=AP2M1 - [AP2M1_HUMAN]	7
Apolipoprotein E OS=Homo sapiens GN=APOE PE=1 SV=1 - [APOE_HUMAN]	7
ATP synthase subunit d, mitochondrial OS=Homo sapiens GN=ATP5H PE=1 SV=3 - [ATP5H_HUMAN]	7
ATP synthase subunit gamma, mitochondrial OS=Homo sapiens GN=ATP5C1 PE=1 SV=1 - [ATPG_HUMAN]	7
ATP synthase subunit O, mitochondrial OS=Homo sapiens GN=ATP5O PE=1 SV=1 - [ATPO_HUMAN]	7
Protein bassoon OS=Homo sapiens GN=BSN PE=2 SV=4 - [BSN_HUMAN]	7
Cell division control protein 42 homolog OS=Homo sapiens GN=CDC42 PE=1 SV=2 - [CDC42_HUMAN]	7
60 kDa heat shock protein, mitochondrial OS=Homo sapiens GN=HSPD1 PE=1 SV=2 - [CH60_HUMAN]	7
MICOS complex subunit MIC19 OS=Homo sapiens GN=CHCHD3 PE=1 SV=1 - [MIC19_HUMAN]	7
Isoform 2 of ATP-dependent RNA helicase DDX3X OS=Homo sapiens GN=DDX3X - [DDX3X_HUMAN]	7
Desmin OS=Homo sapiens GN=DES PE=1 SV=3 - [DESM_HUMAN]	7
Very-long-chain 3-oxoacyl-CoA reductase OS=Homo sapiens GN=HSD17B12 PE=1 SV=2 - [DHB12_HUMAN]	7
Tyrosine-protein kinase Fyn OS=Homo sapiens GN=FYN PE=1 SV=3 - [FYN_HUMAN]	7
Glyceraldehyde-3-phosphate dehydrogenase OS=Homo sapiens GN=GAPDH PE=1 SV=3 - [G3P_HUMAN]	7
Guanine nucleotide-binding protein G(I)/G(S)/G(T) subunit beta-2 OS=Homo sapiens GN=GNB2 PE=1 SV=3 - [GBB2_HUMAN]	7
Mitochondrial glutamate carrier 1 OS=Homo sapiens GN=SLC25A22 PE=1 SV=1 - [GHC1_HUMAN]	7
Guanine nucleotide-binding protein subunit alpha-11 OS=Homo sapiens GN=GNA11 PE=1 SV=2 - [GNA11_HUMAN]	7
Hemoglobin subunit beta OS=Homo sapiens GN=HBB PE=1 SV=2 - [HBB_HUMAN]	7
Homer protein homolog 1 OS=Homo sapiens GN=HOMER1 PE=1 SV=2 - [HOME1_HUMAN]	7

Kelch repeat and BTB domain-containing protein 11 OS=Homo sapiens GN=KBTBD11 PE=1 SV=1 - [KBTBB_HUMAN]	7
Laminin subunit gamma-1 OS=Homo sapiens GN=LAMC1 PE=1 SV=3 - [LAMC1_HUMAN]	7
Methyl-CpG-binding protein 2 OS=Homo sapiens GN=MECP2 PE=1 SV=1 - [MECP2_HUMAN]	7
Nck-associated protein 1 OS=Homo sapiens GN=NCKAP1 PE=1 SV=1 - [NCKP1_HUMAN]	7
Isoform 2 of NADH dehydrogenase [ubiquinone] flavoprotein 1, mitochondrial OS=Homo sapiens GN=NDUFV1 - [NDUV1_HUMAN]	7
Pantothenate kinase 4 OS=Homo sapiens GN=PANK4 PE=1 SV=1 - [PANK4_HUMAN]	7
Isoform 6 of Poly(rC)-binding protein 2 OS=Homo sapiens GN=PCBP2 - [PCBP2_HUMAN]	7
Phosphoglycerate mutase 1 OS=Homo sapiens GN=PGAM1 PE=1 SV=2 - [PGAM1_HUMAN]	7
Prohibitin OS=Homo sapiens GN=PHB PE=1 SV=1 - [PHB_HUMAN]	7
Phosphatidylinositol 5-phosphate 4-kinase type-2 gamma OS=Homo sapiens GN=PIP4K2C PE=1 SV=3 - [PI42C_HUMAN]	7
Isoform 4 of Phosphatidylinositol 4-phosphate 5-kinase type-1 gamma OS=Homo sapiens GN=PIP5K1C - [PI51C_HUMAN]	7
Serine/threonine-protein phosphatase PP1-beta catalytic subunit OS=Homo sapiens GN=PPP1CB PE=1 SV=3 - [PP1B_HUMAN]	7
Prolargin OS=Homo sapiens GN=PRELP PE=1 SV=1 - [PRELP_HUMAN]	7
Ras-related protein Rab-10 OS=Homo sapiens GN=RAB10 PE=1 SV=1 - [RAB10_HUMAN]	7
Ras-related protein Rab-3A OS=Homo sapiens GN=RAB3A PE=1 SV=1 - [RAB3A_HUMAN]	7
Isoform 2 of Heterogeneous nuclear ribonucleoprotein A3 OS=Homo sapiens GN=HNRNPA3 - [ROA3_HUMAN]	7
40S ribosomal protein S3 OS=Homo sapiens GN=RPS3 PE=1 SV=2 - [RS3_HUMAN]	7
Isoform 2 of Solute carrier family 12 member 5 OS=Homo sapiens GN=SLC12A5 - [S12A5_HUMAN]	7
Succinyl-CoA:3-ketoacid coenzyme A transferase 1, mitochondrial OS=Homo sapiens GN=OXCT1 PE=1 SV=1 - [SCOT1_HUMAN]	7
Endophilin-A1 OS=Homo sapiens GN=SH3GL2 PE=1 SV=1 - [SH3G2_HUMAN]	7
Succinyl-CoA ligase [ADP/GDP-forming] subunit alpha, mitochondrial OS=Homo sapiens GN=SUCLG1 PE=1 SV=4 - [SUCA_HUMAN]	7
Isoleucine--tRNA ligase, mitochondrial OS=Homo sapiens GN=IARS2 PE=1 SV=2 - [SYIM_HUMAN]	7
Isoform 3 of T-complex protein 1 subunit eta OS=Homo sapiens GN=CCT7 - [TCPH_HUMAN]	7
T-complex protein 1 subunit zeta OS=Homo sapiens GN=CCT6A PE=1 SV=3 - [TCPZ_HUMAN]	7
Voltage-dependent anion-selective channel protein 1 OS=Homo sapiens GN=VDAC1 PE=1 SV=2 - [VDAC1_HUMAN]	7
Isoform 2 of WD repeat-containing protein 47 OS=Homo sapiens GN=WDR47 - [WDR47_HUMAN]	7
Exportin-1 OS=Homo sapiens GN=XPO1 PE=1 SV=1 - [XPO1_HUMAN]	7
Tyrosine-protein kinase Yes OS=Homo sapiens GN=YES1 PE=1 SV=3 - [YES_HUMAN]	7
Isoform 2 of Phosphatidylinositol 5-phosphate 4-kinase type-2 alpha OS=Homo sapiens GN=PIP4K2A - [PI42A_HUMAN]	7
Hypoxia up-regulated protein 1 OS=Homo sapiens GN=HYOU1 PE=1 SV=1 - [A0A087X054_HUMAN]	7
Isoform 2 of Flotillin-1 OS=Homo sapiens GN=FLOT1 - [FLOT1_HUMAN]	7
Isoform 2 of Probable ATP-dependent RNA helicase DDX5 OS=Homo sapiens GN=DDX5 - [DDX5_HUMAN]	7
Core histone macro-H2A.1 OS=Homo sapiens GN=H2AFY PE=1 SV=1 - [B4DJC3_HUMAN]	7
Doublecortin and CaM kinase-like 2, isoform CRA_c OS=Homo sapiens GN=DCLK2 PE=1 SV=1 - [G5E9L9_HUMAN]	7
40S ribosomal protein S3a (Fragment) OS=Homo sapiens GN=RPS3A PE=1 SV=1 - [D6RG13_HUMAN]	7
Myosin light polypeptide 6 OS=Homo sapiens GN=MYL6 PE=1 SV=1 - [F8W1R7_HUMAN]	7
Septin-11 (Fragment) OS=Homo sapiens GN=SEPT11 PE=1 SV=1 - [D6RDU5_HUMAN]	7
Mitogen-activated protein kinase 10 (Fragment) OS=Homo sapiens GN=MAPK10 PE=1 SV=1 - [H0Y9H3_HUMAN]	7
Pyruvate kinase (Fragment) OS=Homo sapiens GN=PKM PE=1 SV=1 - [H3BTN5_HUMAN]	7
Sideroflexin-3 OS=Homo sapiens GN=SFXN3 PE=1 SV=1 - [A0A0A0MS41_HUMAN]	7
Voltage-dependent anion-selective channel protein 2 (Fragment) OS=Homo sapiens GN=VDAC2 PE=1 SV=1 - [A0A0A0MR02_HUMAN]	7
GRIP1-associated protein 1 OS=Homo sapiens GN=GRIPAP1 PE=1 SV=1 - [A0A087WT45_HUMAN]	7
Isoform 2 of Beta-adducin OS=Homo sapiens GN=ADD2 - [ADDB_HUMAN]	6
Fructose-bisphosphate aldolase A OS=Homo sapiens GN=ALDOA PE=1 SV=2 - [ALDOA_HUMAN]	6
Annexin A5 OS=Homo sapiens GN=ANXA5 PE=1 SV=2 - [ANXA5_HUMAN]	6
D-beta-hydroxybutyrate dehydrogenase, mitochondrial OS=Homo sapiens GN=BDH1 PE=1 SV=3 - [BDH_HUMAN]	6
CAP-Gly domain-containing linker protein 2 OS=Homo sapiens GN=CLIP2 PE=1 SV=1 - [CLIP2_HUMAN]	6
Copine-5 OS=Homo sapiens GN=CPNE5 PE=1 SV=2 - [CPNE5_HUMAN]	6
COP9 signalosome complex subunit 4 OS=Homo sapiens GN=COPS4 PE=1 SV=1 - [CSN4_HUMAN]	6
Isoform 3 of Disks large homolog 2 OS=Homo sapiens GN=DLG2 - [DLG2_HUMAN]	6
Protein FAM49A OS=Homo sapiens GN=FAM49A PE=1 SV=1 - [FA49A_HUMAN]	6
FERM, RhoGEF and pleckstrin domain-containing protein 1 OS=Homo sapiens GN=FARP1 PE=1 SV=1 - [FARP1_HUMAN]	6
Guanine nucleotide-binding protein G(i) subunit alpha-1 OS=Homo sapiens GN=GNAI1 PE=1 SV=2 - [GNAI1_HUMAN]	6

Isoform 2 of Glycerol-3-phosphate dehydrogenase, mitochondrial OS=Homo sapiens GN=GPD2 - [GPDM_HUMAN]	6
Neuronal membrane glycoprotein M6-a OS=Homo sapiens GN=GPM6A PE=1 SV=2 - [GPM6A_HUMAN]	6
Heterogeneous nuclear ribonucleoprotein H2 OS=Homo sapiens GN=HNRNPH2 PE=1 SV=1 - [HNRH2_HUMAN]	6
Neuron-specific calcium-binding protein hippocalcin OS=Homo sapiens GN=HPCA PE=1 SV=2 - [HPCA_HUMAN]	6
Isocitrate dehydrogenase [NAD] subunit alpha, mitochondrial OS=Homo sapiens GN=IDH3A PE=1 SV=1 - [IDH3A_HUMAN]	6
Eukaryotic initiation factor 4A-II OS=Homo sapiens GN=EIF4A2 PE=1 SV=2 - [IF4A2_HUMAN]	6
IQ motif and SEC7 domain-containing protein 1 OS=Homo sapiens GN=IQSEC1 PE=1 SV=1 - [IQEC1_HUMAN]	6
Kinesin heavy chain isoform 5C OS=Homo sapiens GN=KIF5C PE=1 SV=1 - [KIF5C_HUMAN]	6
Protein kinase C alpha type OS=Homo sapiens GN=PRKCA PE=1 SV=4 - [KPCA_HUMAN]	6
Isoform 6 of Liprin-alpha-2 OS=Homo sapiens GN=PPFIA2 - [LIPA2_HUMAN]	6
Matrin-3 OS=Homo sapiens GN=MATR3 PE=1 SV=2 - [MATR3_HUMAN]	6
Isoform B of Phosphate carrier protein, mitochondrial OS=Homo sapiens GN=SLC25A3 - [MPCP_HUMAN]	6
Myosin-10 OS=Homo sapiens GN=MYH10 PE=1 SV=3 - [MYH10_HUMAN]	6
Isoform 4 of Myosin-11 OS=Homo sapiens GN=MYH11 - [MYH11_HUMAN]	6
NADH-cytochrome b5 reductase 1 OS=Homo sapiens GN=CYB5R1 PE=1 SV=1 - [NB5R1_HUMAN]	6
Isoform 2 of Nebulette OS=Homo sapiens GN=NEBL - [NEBL_HUMAN]	6
Dihydrolipoyllysine-residue succinyltransferase component of 2-oxoglutarate dehydrogenase complex, mitochondrial OS=Homo sapiens GN=DLST PE=1 SV=4 - [ODO2_HUMAN]	6
Dihydrolipoyllysine-residue acetyltransferase component of pyruvate dehydrogenase complex, mitochondrial OS=Homo sapiens GN=DLAT PE=1 SV=3 - [ODP2_HUMAN]	6
Dynamin-like 120 kDa protein, mitochondrial OS=Homo sapiens GN=OPA1 PE=1 SV=3 - [OPA1_HUMAN]	6
Serine/threonine-protein kinase OSR1 OS=Homo sapiens GN=OXSR1 PE=1 SV=1 - [OXSR1_HUMAN]	6
Poly(rC)-binding protein 1 OS=Homo sapiens GN=PCBP1 PE=1 SV=2 - [PCBP1_HUMAN]	6
[Pyruvate dehydrogenase (acetyl-transferring)] kinase isozyme 3, mitochondrial OS=Homo sapiens GN=PDK3 PE=1 SV=1 - [PDK3_HUMAN]	6
Putative 3-phosphoinositide-dependent protein kinase 2 OS=Homo sapiens GN=PDPK2P PE=5 SV=1 - [PDPK2_HUMAN]	6
Brevican core protein OS=Homo sapiens GN=BCAN PE=1 SV=2 - [PGCB_HUMAN]	6
Biglycan OS=Homo sapiens GN=BGN PE=1 SV=2 - [PGS1_HUMAN]	6
Peroxiredoxin-2 OS=Homo sapiens GN=PRDX2 PE=1 SV=5 - [PRDX2_HUMAN]	6
Isoform Cytoplasmic+peroxisomal of Peroxiredoxin-5, mitochondrial OS=Homo sapiens GN=PRDX5 - [PRDX5_HUMAN]	6
26S proteasome non-ATPase regulatory subunit 13 OS=Homo sapiens GN=PSMD13 PE=1 SV=2 - [PSD13_HUMAN]	6
Ras-related protein Rab-5C OS=Homo sapiens GN=RAB5C PE=1 SV=2 - [RAB5C_HUMAN]	6
Ras-related protein Rap-1A OS=Homo sapiens GN=RAP1A PE=1 SV=1 - [RAP1A_HUMAN]	6
RNA 3'-terminal phosphate cyclase OS=Homo sapiens GN=RTCA PE=1 SV=1 - [RTCA_HUMAN]	6
Isoform RTN1-C of Reticulon-1 OS=Homo sapiens GN=RTN1 - [RTN1_HUMAN]	6
Sideroflexin-1 OS=Homo sapiens GN=SFXN1 PE=1 SV=4 - [SFXN1_HUMAN]	6
Sorting nexin-3 OS=Homo sapiens GN=SNX3 PE=1 SV=3 - [SNX3_HUMAN]	6
Signal transducer and activator of transcription 1-alpha/beta OS=Homo sapiens GN=STAT1 PE=1 SV=2 - [STAT1_HUMAN]	6
Arginine--tRNA ligase, cytoplasmic OS=Homo sapiens GN=RARS PE=1 SV=2 - [SYRC_HUMAN]	6
Synaptotagmin-7 OS=Homo sapiens GN=SYT7 PE=1 SV=3 - [SYT7_HUMAN]	6
Transgelin OS=Homo sapiens GN=TAGLN PE=1 SV=4 - [TAGL_HUMAN]	6
Tenascin-R OS=Homo sapiens GN=TNR PE=1 SV=3 - [TENR_HUMAN]	6
Isoform 2 of UBX domain-containing protein 6 OS=Homo sapiens GN=UBXN6 - [UBXN6_HUMAN]	6
WD repeat-containing protein 1 OS=Homo sapiens GN=WDR1 PE=1 SV=4 - [WDR1_HUMAN]	6
Isoform 2 of T-complex protein 1 subunit epsilon OS=Homo sapiens GN=CCT5 - [TCPE_HUMAN]	6
Isoform 2 of Aspartate--tRNA ligase, cytoplasmic OS=Homo sapiens GN=DARS - [SYDC_HUMAN]	6
Capping protein (Actin filament) muscle Z-line, beta, isoform CRA_a OS=Homo sapiens GN=CAPZB PE=1 SV=1 - [B1AK87_HUMAN]	6
Erlin-2 (Fragment) OS=Homo sapiens GN=ERLIN2 PE=1 SV=1 - [E5RHW4_HUMAN]	6
Isoform 4 of Heterogeneous nuclear ribonucleoprotein R OS=Homo sapiens GN=HNRNPR - [HNRPR_HUMAN]	6
Acylglycerol kinase, mitochondrial OS=Homo sapiens GN=AGK PE=1 SV=1 - [E9PC15_HUMAN]	6
Heterogeneous nuclear ribonucleoprotein H OS=Homo sapiens GN=HNRNPH1 PE=1 SV=1 - [E9PCY7_HUMAN]	6
Catenin delta-2 OS=Homo sapiens GN=CTNND2 PE=1 SV=1 - [E7EPC8_HUMAN]	6
Myelin-oligodendrocyte glycoprotein OS=Homo sapiens GN=MOG PE=1 SV=1 - [C9JTE0_HUMAN]	6
Ubiquitin carboxyl-terminal hydrolase OS=Homo sapiens GN=USP14 PE=1 SV=2 - [A6NJA2_HUMAN]	6
Keratin, type I cytoskeletal 13 OS=Homo sapiens GN=KRT13 PE=1 SV=1 - [K7ERE3_HUMAN]	6
Isoform 2 of Guanine nucleotide-binding protein G(I)/G(S)/G(T) subunit beta-1 OS=Homo sapiens GN=GNB1 - [GBB1_HUMAN]	6

Interleukin enhancer-binding factor 2 OS=Homo sapiens GN=ILF2 PE=1 SV=1 - [B4DY09_HUMAN]	6
Peroxiredoxin-1 (Fragment) OS=Homo sapiens GN=PRDX1 PE=1 SV=1 - [A0A0A0MSI0_HUMAN]	6
cAMP-dependent protein kinase catalytic subunit beta OS=Homo sapiens GN=PRKACB PE=1 SV=1 - [A0A087WVC4_HUMAN]	6
Clathrin coat assembly protein AP180 (Fragment) OS=Homo sapiens GN=SNAP91 PE=1 SV=4 - [E5RGY9_HUMAN]	6
Myelin expression factor 2 OS=Homo sapiens GN=MYEF2 PE=1 SV=1 - [A0A087WUT0_HUMAN]	6
Septin-8 OS=Homo sapiens GN=SEPT8 PE=1 SV=1 - [A0A087X142_HUMAN]	6
Septin-7 OS=Homo sapiens GN=SEPT7 PE=1 SV=3 - [E7ES33_HUMAN]	6
Collagen alpha-1(VI) chain OS=Homo sapiens GN=COL6A1 PE=1 SV=1 - [A0A087X0S5_HUMAN]	6
Band 4.1-like protein 1 OS=Homo sapiens GN=EPB41L1 PE=4 SV=1 - [A0A0C4DH22_HUMAN]	6
Isoform 3 of Acyl-coenzyme A thioesterase 9, mitochondrial OS=Homo sapiens GN=ACOT9 - [ACOT9_HUMAN]	5
Isoform 2 of Arf-GAP with GTPase, ANK repeat and PH domain-containing protein 2 OS=Homo sapiens GN=AGAP2 - [AGAP2_HUMAN]	5
Alcohol dehydrogenase [NADP(+)] OS=Homo sapiens GN=AKR1A1 PE=1 SV=3 - [AK1A1_HUMAN]	5
Isoform 2 of Amphiphysin OS=Homo sapiens GN=AMPH - [AMPH_HUMAN]	5
Amine oxidase [flavin-containing] A OS=Homo sapiens GN=MAOA PE=1 SV=1 - [AOFA_HUMAN]	5
ADP-ribosylation factor 3 OS=Homo sapiens GN=ARF3 PE=1 SV=2 - [ARF3_HUMAN]	5
Actin-related protein 2/3 complex subunit 3 OS=Homo sapiens GN=ARPC3 PE=1 SV=3 - [ARPC3_HUMAN]	5
Isoform 2 of Sodium/potassium-transporting ATPase subunit beta-1 OS=Homo sapiens GN=ATP1B1 - [AT1B1_HUMAN]	5
Isoform 2 of Late secretory pathway protein AVL9 homolog OS=Homo sapiens GN=AVL9 - [AVL9_HUMAN]	5
Isoform IIC1 of Myc box-dependent-interacting protein 1 OS=Homo sapiens GN=BIN1 - [BIN1_HUMAN]	5
Isoform 3 of CaM kinase-like vesicle-associated protein OS=Homo sapiens GN=CAMKV - [CAMKV_HUMAN]	5
Isoform 2 of Adenylyl cyclase-associated protein 1 OS=Homo sapiens GN=CAP1 - [CAP1_HUMAN]	5
Collagen alpha-2(IV) chain OS=Homo sapiens GN=COL4A2 PE=1 SV=4 - [CO4A2_HUMAN]	5
Cytochrome c oxidase subunit 2 OS=Homo sapiens GN=MT-CO2 PE=1 SV=1 - [COX2_HUMAN]	5
Cytochrome c oxidase subunit 4 isoform 1, mitochondrial OS=Homo sapiens GN=COX4I1 PE=1 SV=1 - [COX41_HUMAN]	5
Casein kinase II subunit alpha' OS=Homo sapiens GN=CSNK2A2 PE=1 SV=1 - [CSK22_HUMAN]	5
Caskin-1 OS=Homo sapiens GN=CASKIN1 PE=1 SV=1 - [CSKI1_HUMAN]	5
Isoform 2 of Epimerase family protein SDR39U1 OS=Homo sapiens GN=SDR39U1 - [D39U1_HUMAN]	5
Isoform 2 of N(G),N(G)-dimethylarginine dimethylaminohydrolase 1 OS=Homo sapiens GN=DDAH1 - [DDAH1_HUMAN]	5
Isoform 2 of Probable ATP-dependent RNA helicase DDX17 OS=Homo sapiens GN=DDX17 - [DDX17_HUMAN]	5
GTP-binding protein Di-Ras2 OS=Homo sapiens GN=DIRAS2 PE=1 SV=1 - [DIRA2_HUMAN]	5
Disks large homolog 3 OS=Homo sapiens GN=DLG3 PE=1 SV=2 - [DLG3_HUMAN]	5
Dihydropyrimidinase-related protein 4 OS=Homo sapiens GN=DPYSL4 PE=1 SV=2 - [DPYL4_HUMAN]	5
Elongation factor 1-gamma OS=Homo sapiens GN=EEF1G PE=1 SV=3 - [EF1G_HUMAN]	5
Alpha-enolase OS=Homo sapiens GN=ENO1 PE=1 SV=2 - [ENOA_HUMAN]	5
F-box only protein 2 OS=Homo sapiens GN=FBXO2 PE=1 SV=2 - [FBX2_HUMAN]	5
Isoform 2 of Fibrinogen alpha chain OS=Homo sapiens GN=FGA - [FIBA_HUMAN]	5
Ferritin heavy chain OS=Homo sapiens GN=FTH1 PE=1 SV=2 - [FRIH_HUMAN]	5
Histone H1.2 OS=Homo sapiens GN=HIST1H1C PE=1 SV=2 - [H12_HUMAN]	5
Huntingtin-interacting protein 1-related protein OS=Homo sapiens GN=HIP1R PE=1 SV=2 - [HIP1R_HUMAN]	5
Isoform 3 of Heterogeneous nuclear ribonucleoprotein H3 OS=Homo sapiens GN=HNRNPH3 - [HNRH3_HUMAN]	5
Heterogeneous nuclear ribonucleoprotein U OS=Homo sapiens GN=HNRNPU PE=1 SV=6 - [HNRPU_HUMAN]	5
Hippocalcin-like protein 1 OS=Homo sapiens GN=HPCAL1 PE=1 SV=3 - [HPCL1_HUMAN]	5
Hyaluronan and proteoglycan link protein 1 OS=Homo sapiens GN=HAPLN1 PE=2 SV=2 - [HPLN1_HUMAN]	5
Isocitrate dehydrogenase [NAD] subunit beta, mitochondrial OS=Homo sapiens GN=IDH3B PE=1 SV=2 - [IDH3B_HUMAN]	5
Immunoglobulin superfamily member 8 OS=Homo sapiens GN=IGSF8 PE=1 SV=1 - [IGSF8_HUMAN]	5
Importin subunit beta-1 OS=Homo sapiens GN=KPNB1 PE=1 SV=2 - [IMB1_HUMAN]	5
Adenylate kinase isoenzyme 1 OS=Homo sapiens GN=AK1 PE=1 SV=3 - [KAD1_HUMAN]	5
cAMP-dependent protein kinase type II-beta regulatory subunit OS=Homo sapiens GN=PRKAR2B PE=1 SV=3 - [KAP3_HUMAN]	5
Isoform 2 of cAMP-dependent protein kinase catalytic subunit alpha OS=Homo sapiens GN=PRKACA - [KAPCA_HUMAN]	5
Isoform 2 of Calcium/calmodulin-dependent protein kinase type 1D OS=Homo sapiens GN=CAMK1D - [KCC1D_HUMAN]	5
UMP-CMP kinase OS=Homo sapiens GN=CMPK1 PE=1 SV=3 - [KCY_HUMAN]	5
Guanylate kinase OS=Homo sapiens GN=GUK1 PE=1 SV=2 - [KGUA_HUMAN]	5
Protein kinase C beta type OS=Homo sapiens GN=PRKCB PE=1 SV=4 - [KPCB_HUMAN]	5

Laminin subunit alpha-5 OS=Homo sapiens GN=LAMA5 PE=1 SV=8 - [LAMA5_HUMAN]	5
L-lactate dehydrogenase B chain OS=Homo sapiens GN=LDHB PE=1 SV=2 - [LDHB_HUMAN]	5
Protein lin-7 homolog A OS=Homo sapiens GN=LIN7A PE=1 SV=2 - [LIN7A_HUMAN]	5
Leucine-rich repeat-containing protein 47 OS=Homo sapiens GN=LRRC47 PE=1 SV=1 - [LRC47_HUMAN]	5
Isoform 2 of Malate dehydrogenase, cytoplasmic OS=Homo sapiens GN=MDH1 - [MDHC_HUMAN]	5
Isoform 2 of Mitofusin-2 OS=Homo sapiens GN=MFN2 - [MFN2_HUMAN]	5
Isoform 5 of Misshapen-like kinase 1 OS=Homo sapiens GN=MINK1 - [MINK1_HUMAN]	5
Dual specificity mitogen-activated protein kinase kinase 4 OS=Homo sapiens GN=MAP2K4 PE=1 SV=1 - [MP2K4_HUMAN]	5
Myelin proteolipid protein OS=Homo sapiens GN=PLP1 PE=1 SV=2 - [MYPR_HUMAN]	5
Neurocan core protein OS=Homo sapiens GN=NCAN PE=1 SV=3 - [NCAN_HUMAN]	5
NADH dehydrogenase [ubiquinone] 1 alpha subcomplex subunit 9, mitochondrial OS=Homo sapiens GN=NDUFA9 PE=1 SV=2 - [NDUA9_HUMAN]	5
Isoform 2 of NADH dehydrogenase [ubiquinone] iron-sulfur protein 2, mitochondrial OS=Homo sapiens GN=NDUFS2 - [NDUS2_HUMAN]	5
Protein NipSnap homolog 1 OS=Homo sapiens GN=NIPSNAP1 PE=1 SV=1 - [NIPS1_HUMAN]	5
Lipoamide acyltransferase component of branched-chain alpha-keto acid dehydrogenase complex, mitochondrial OS=Homo sapiens GN=DBT PE=1 SV=3 - [ODB2_HUMAN]	5
Isoform 3 of Pyruvate dehydrogenase E1 component subunit alpha, somatic form, mitochondrial OS=Homo sapiens GN=PDHA1 - [ODPA_HUMAN]	5
Platelet-activating factor acetylhydrolase IB subunit gamma OS=Homo sapiens GN=PAFAH1B3 PE=1 SV=1 - [PA1B3_HUMAN]	5
Poly [ADP-ribose] polymerase 1 OS=Homo sapiens GN=PARP1 PE=1 SV=4 - [PARP1_HUMAN]	5
Phosphatidylinositol 5-phosphate 4-kinase type-2 beta OS=Homo sapiens GN=PIP4K2B PE=1 SV=1 - [PI42B_HUMAN]	5
Serine/threonine-protein phosphatase 2A catalytic subunit beta isoform OS=Homo sapiens GN=PPP2CB PE=1 SV=1 - [PP2AB_HUMAN]	5
Peptidyl-prolyl cis-trans isomerase A OS=Homo sapiens GN=PPIA PE=1 SV=2 - [PPIA_HUMAN]	5
Peptidyl-prolyl cis-trans isomerase B OS=Homo sapiens GN=PPIB PE=1 SV=2 - [PPIB_HUMAN]	5
26S proteasome non-ATPase regulatory subunit 12 OS=Homo sapiens GN=PSMD12 PE=1 SV=3 - [PSD12_HUMAN]	5
Ras-related protein Rab-3C OS=Homo sapiens GN=RAB3C PE=2 SV=1 - [RAB3C_HUMAN]	5
Isoform 2 of Ras-related protein Rab-5B OS=Homo sapiens GN=RAB5B - [RAB5B_HUMAN]	5
Ras-related C3 botulinum toxin substrate 1 OS=Homo sapiens GN=RAC1 PE=1 SV=1 - [RAC1_HUMAN]	5
60S ribosomal protein L4 OS=Homo sapiens GN=RPL4 PE=1 SV=5 - [RL4_HUMAN]	5
Isoform RTN1-B of Reticulon-1 OS=Homo sapiens GN=RTN1 - [RTN1_HUMAN]	5
Protein S100-A9 OS=Homo sapiens GN=S100A9 PE=1 SV=1 - [S10A9_HUMAN]	5
GTP-binding protein SAR1a OS=Homo sapiens GN=SAR1A PE=1 SV=1 - [SAR1A_HUMAN]	5
Isoform 4 of Septin-9 OS=Homo sapiens GN=SEPT9 - [SEPT9_HUMAN]	5
Synaptosomal-associated protein 25 OS=Homo sapiens GN=SNAP25 PE=1 SV=1 - [SNP25_HUMAN]	5
Isoform 2 of Sorting nexin-12 OS=Homo sapiens GN=SNX12 - [SNX12_HUMAN]	5
Isoform 2 of Suppressor of G2 allele of SKP1 homolog OS=Homo sapiens GN=SUGT1 - [SUGT1_HUMAN]	5
Isoform Tau-D of Microtubule-associated protein tau OS=Homo sapiens GN=MAPT - [TAU_HUMAN]	5
Isoform 2 of Triosephosphate isomerase OS=Homo sapiens GN=TPI1 - [TPIS_HUMAN]	5
Isoform 2 of Tripeptidyl-peptidase 1 OS=Homo sapiens GN=TPP1 - [TPP1_HUMAN]	5
Tubulin polymerization-promoting protein OS=Homo sapiens GN=TPPP PE=1 SV=1 - [TPPP_HUMAN]	5
Isoform 4 of Tripartite motif-containing protein 3 OS=Homo sapiens GN=TRIM3 - [TRIM3_HUMAN]	5
Ubiquitin-like modifier-activating enzyme 6 OS=Homo sapiens GN=UBA6 PE=1 SV=1 - [UBA6_HUMAN]	5
Ubiquitin-conjugating enzyme E2 N OS=Homo sapiens GN=UBE2N PE=1 SV=1 - [UBE2N_HUMAN]	5
V-type proton ATPase subunit C 1 OS=Homo sapiens GN=ATP6V1C1 PE=1 SV=4 - [VATC1_HUMAN]	5
Vacuolar protein sorting-associated protein 51 homolog OS=Homo sapiens GN=VPS51 PE=1 SV=2 - [VPS51_HUMAN]	5
Isoform 4 of Vacuolar protein sorting-associated protein 53 homolog OS=Homo sapiens GN=VPS53 - [VPS53_HUMAN]	5
WD repeat-containing protein 37 OS=Homo sapiens GN=WDR37 PE=1 SV=2 - [WDR37_HUMAN]	5
Isoform 4 of Exportin-2 OS=Homo sapiens GN=CSE1L - [XPO2_HUMAN]	5
Septin-5 (Fragment) OS=Homo sapiens GN=SEPT5 PE=1 SV=1 - [C9JM82_HUMAN]	5
Isoform 2 of Leucine--tRNA ligase, cytoplasmic OS=Homo sapiens GN=LARS - [SYLC_HUMAN]	5
Isoform 3 of Integrin-linked protein kinase OS=Homo sapiens GN=ILK - [ILK_HUMAN]	5
Isoform 4 of Dematin OS=Homo sapiens GN=DMTN - [DEMA_HUMAN]	5
Isoform 2 of Ras-related protein Rab-5A OS=Homo sapiens GN=RAB5A - [RAB5A_HUMAN]	5
60S ribosomal protein L7 OS=Homo sapiens GN=RPL7 PE=1 SV=1 - [A8MUD9_HUMAN]	5
Isocitrate dehydrogenase [NAD] subunit, mitochondrial OS=Homo sapiens GN=IDH3G PE=3 SV=1 - [G5E9Q7_HUMAN]	5
Isoform 2 of Gamma-enolase OS=Homo sapiens GN=ENO2 - [ENOG_HUMAN]	5

ATPase, H+ transporting, lysosomal 50/57kDa, V1 subunit H, isoform CRA_c OS=Homo sapiens GN=ATP6V1H PE=1 SV=1 - [G3V126_HUMAN]	5
40S ribosomal protein S8 OS=Homo sapiens GN=RPS8 PE=1 SV=1 - [Q5JR95_HUMAN]	5
ATP synthase subunit g, mitochondrial OS=Homo sapiens GN=ATP5L PE=1 SV=1 - [E9PN17_HUMAN]	5
Maestro heat-like repeat-containing protein family member 1 OS=Homo sapiens GN=MROH1 PE=1 SV=1 - [E9PHY8_HUMAN]	5
Isoform 3 of Lon protease homolog, mitochondrial OS=Homo sapiens GN=LONP1 - [LONM_HUMAN]	5
Kinesin-like protein (Fragment) OS=Homo sapiens GN=KIF1A PE=1 SV=1 - [F8W8V9_HUMAN]	5
Pleckstrin homology domain-containing family B member 1 (Fragment) OS=Homo sapiens GN=PLEKHB1 PE=1 SV=1 - [F5GZH3_HUMAN]	5
Neuronal-specific septin-3 OS=Homo sapiens GN=SEPT3 PE=1 SV=1 - [B1AHR1_HUMAN]	5
Heat shock 70 kDa protein 4L (Fragment) OS=Homo sapiens GN=HSPA4L PE=1 SV=1 - [D6RJ96_HUMAN]	5
Isoform 2 of Heme oxygenase 2 OS=Homo sapiens GN=HMOX2 - [HMOX2_HUMAN]	5
Alpha-centractin OS=Homo sapiens GN=ACTR1A PE=1 SV=1 - [R4GMT0_HUMAN]	5
Isoform 2 of Alpha-1-syntrophin OS=Homo sapiens GN=SNTA1 - [SNTA1_HUMAN]	5
NAD-dependent protein deacetylase sirtuin-2 OS=Homo sapiens GN=SIRT2 PE=1 SV=1 - [A0A0A0MRF5_HUMAN]	5
Electron transfer flavoprotein subunit alpha, mitochondrial (Fragment) OS=Homo sapiens GN=ETFA PE=1 SV=4 - [H0YL12_HUMAN]	5
Nidogen-2 OS=Homo sapiens GN=NID2 PE=1 SV=1 - [A0A087WZP6_HUMAN]	5
Gamma-adducin OS=Homo sapiens GN=ADD3 PE=1 SV=1 - [A0A087WX08_HUMAN]	5
Serine/threonine-protein phosphatase 2A 55 kDa regulatory subunit B alpha isoform OS=Homo sapiens GN=PPP2R2A PE=1 SV=1 - [2ABA_HUMAN]	4
Isoform 4 of Active breakpoint cluster region-related protein OS=Homo sapiens GN=ABR - [ABR_HUMAN]	4
Isoform 2 of ATP-citrate synthase OS=Homo sapiens GN=ACLY - [ACLY_HUMAN]	4
Isoform 3 of Arf-GAP with GTPase, ANK repeat and PH domain-containing protein 3 OS=Homo sapiens GN=AGAP3 - [AGAP3_HUMAN]	4
Apolipoprotein L2 OS=Homo sapiens GN=APOL2 PE=1 SV=1 - [APOL2_HUMAN]	4
ADP-ribosylation factor 5 OS=Homo sapiens GN=ARF5 PE=1 SV=2 - [ARF5_HUMAN]	4
ADP-ribosylation factor-like protein 2 OS=Homo sapiens GN=ARL2 PE=1 SV=4 - [ARL2_HUMAN]	4
ADP-ribosylation factor-like protein 8B OS=Homo sapiens GN=ARL8B PE=1 SV=1 - [ARL8B_HUMAN]	4
Isoform 3 of ATPase family AAA domain-containing protein 3A OS=Homo sapiens GN=ATAD3A - [ATD3A_HUMAN]	4
Isoform 4 of Large proline-rich protein BAG6 OS=Homo sapiens GN=BAG6 - [BAG6_HUMAN]	4
Flavin reductase (NADPH) OS=Homo sapiens GN=BLVRB PE=1 SV=3 - [BLVRB_HUMAN]	4
Cathepsin D OS=Homo sapiens GN=CTSD PE=1 SV=1 - [CATD_HUMAN]	4
CDGSH iron-sulfur domain-containing protein 1 OS=Homo sapiens GN=CISD1 PE=1 SV=1 - [CISD1_HUMAN]	4
Isoform 2 of Cytoskeleton-associated protein 5 OS=Homo sapiens GN=CKAP5 - [CKAP5_HUMAN]	4
Gap junction alpha-1 protein OS=Homo sapiens GN=GJA1 PE=1 SV=2 - [CXA1_HUMAN]	4
Isoform 3 of Calcyclin-binding protein OS=Homo sapiens GN=CACYBP - [CYBP_HUMAN]	4
Dynactin subunit 2 OS=Homo sapiens GN=DCTN2 PE=1 SV=4 - [DCTN2_HUMAN]	4
Isoform 2 of Destrin OS=Homo sapiens GN=DSTN - [DEST_HUMAN]	4
Isoform 8 of Disks large homolog 1 OS=Homo sapiens GN=DLG1 - [DLG1_HUMAN]	4
Dihydropyrimidinase-related protein 3 OS=Homo sapiens GN=DPYSL3 PE=1 SV=1 - [DPYL3_HUMAN]	4
Isoform 5 of Double-stranded RNA-specific adenosine deaminase OS=Homo sapiens GN=ADAR - [DSRAD_HUMAN]	4
Emerin OS=Homo sapiens GN=EMD PE=1 SV=1 - [EMD_HUMAN]	4
Isoform 2 of 6-phosphofructo-2-kinase/fructose-2,6-bisphosphatase 2 OS=Homo sapiens GN=PFKFB2 - [F262_HUMAN]	4
Ferritin light chain OS=Homo sapiens GN=FTL PE=1 SV=2 - [FRIL_HUMAN]	4
Isoform 2 of Growth arrest-specific protein 7 OS=Homo sapiens GN=GAS7 - [GAS7_HUMAN]	4
Isoform 2 of Ganglioside-induced differentiation-associated protein 1 OS=Homo sapiens GN=GDAP1 - [GDAP1_HUMAN]	4
Mitochondrial glutamate carrier 2 OS=Homo sapiens GN=SLC25A18 PE=1 SV=1 - [GHC2_HUMAN]	4
Isoform 2 of Histone H1.0 OS=Homo sapiens GN=H1F0 - [H10_HUMAN]	4
Histone H2A.Z OS=Homo sapiens GN=H2AFZ PE=1 SV=2 - [H2AZ_HUMAN]	4
Histone H2B type 1-K OS=Homo sapiens GN=HIST1H2BK PE=1 SV=3 - [H2B1K_HUMAN]	4
Isoform 3 of Heterogeneous nuclear ribonucleoprotein D-like OS=Homo sapiens GN=HNRNPDL - [HNRDL_HUMAN]	4
Isoform 2 of Inverted formin-2 OS=Homo sapiens GN=INF2 - [INF2_HUMAN]	4
Isoform 3 of IQ motif and SEC7 domain-containing protein 2 OS=Homo sapiens GN=IQSEC2 - [IQEC2_HUMAN]	4
Isoform 6 of Kinesin-like protein KIF21A OS=Homo sapiens GN=KIF21A - [KI21A_HUMAN]	4
Kinesin-1 heavy chain OS=Homo sapiens GN=KIF5B PE=1 SV=1 - [KINH_HUMAN]	4
Isoform 4 of L-lactate dehydrogenase A chain OS=Homo sapiens GN=LDHA - [LDHA_HUMAN]	4
LETM1 and EF-hand domain-containing protein 1, mitochondrial OS=Homo sapiens GN=LETM1 PE=1 SV=1 - [LETM1_HUMAN]	4

Isoform 2 of Lactadherin OS=Homo sapiens GN=MFGE8 - [MFGM_HUMAN]	4
Isoform 2 of Unconventional myosin-Ic OS=Homo sapiens GN=MYO1C - [MYO1C_HUMAN]	4
Nucleoside diphosphate kinase A OS=Homo sapiens GN=NME1 PE=1 SV=1 - [NDKA_HUMAN]	4
NADH dehydrogenase [ubiquinone] 1 alpha subcomplex subunit 8 OS=Homo sapiens GN=NDUFA8 PE=1 SV=3 - [NDUA8_HUMAN]	4
Serine/threonine-protein kinase Nek7 OS=Homo sapiens GN=NEK7 PE=1 SV=1 - [NEK7_HUMAN]	4
Protein NipSnap homolog 2 OS=Homo sapiens GN=GBAS PE=1 SV=1 - [NIPS2_HUMAN]	4
Astrocytic phosphoprotein PEA-15 OS=Homo sapiens GN=PEA15 PE=1 SV=2 - [PEA15_HUMAN]	4
Isoform 2 of Phytanoyl-CoA hydroxylase-interacting protein-like OS=Homo sapiens GN=PHYHIPL - [PHIPL_HUMAN]	4
Protein-L-isoaspartate(D-aspartate) O-methyltransferase OS=Homo sapiens GN=PCMT1 PE=1 SV=4 - [PIMT_HUMAN]	4
Isoform 3 of Serine/threonine-protein phosphatase 2B catalytic subunit alpha isoform OS=Homo sapiens GN=PPP3CA - [PP2BA_HUMAN]	4
Isoform 2 of Major prion protein OS=Homo sapiens GN=PRNP - [PRIO_HUMAN]	4
Isoform 3 of Interferon-inducible double-stranded RNA-dependent protein kinase activator A OS=Homo sapiens GN=PRKRA - [PRKRA_HUMAN]	4
26S protease regulatory subunit 7 OS=Homo sapiens GN=PSMC2 PE=1 SV=3 - [PRS7_HUMAN]	4
Isoform 3 of Serine/threonine-protein phosphatase 2A activator OS=Homo sapiens GN=PPP2R4 - [PTPA_HUMAN]	4
Isoform 2 of Bifunctional purine biosynthesis protein PURH OS=Homo sapiens GN=ATIC - [PUR9_HUMAN]	4
Transcriptional activator protein Pur-alpha OS=Homo sapiens GN=PURA PE=1 SV=2 - [PURA_HUMAN]	4
Ras-related protein Rab-1A OS=Homo sapiens GN=RAB1A PE=1 SV=3 - [RAB1A_HUMAN]	4
Ras-related protein Rab-1B OS=Homo sapiens GN=RAB1B PE=1 SV=1 - [RAB1B_HUMAN]	4
Ras-related protein Rab-31 OS=Homo sapiens GN=RAB31 PE=1 SV=1 - [RAB31_HUMAN]	4
Ras-related protein Rab-6B OS=Homo sapiens GN=RAB6B PE=1 SV=1 - [RAB6B_HUMAN]	4
GTP-binding nuclear protein Ran OS=Homo sapiens GN=RAN PE=1 SV=3 - [RAN_HUMAN]	4
Ras-related protein Rab-11B OS=Homo sapiens GN=RAB11B PE=1 SV=4 - [RB11B_HUMAN]	4
Rho GTPase-activating protein 1 OS=Homo sapiens GN=ARHGAP1 PE=1 SV=1 - [RHG01_HUMAN]	4
Ribonuclease inhibitor OS=Homo sapiens GN=RNH1 PE=1 SV=2 - [RINI_HUMAN]	4
Isoform 2 of 60S ribosomal protein L11 OS=Homo sapiens GN=RPL11 - [RL11_HUMAN]	4
60S ribosomal protein L23 OS=Homo sapiens GN=RPL23 PE=1 SV=1 - [RL23_HUMAN]	4
60S ribosomal protein L6 OS=Homo sapiens GN=RPL6 PE=1 SV=3 - [RL6_HUMAN]	4
Heterogeneous nuclear ribonucleoprotein A0 OS=Homo sapiens GN=HNRNPA0 PE=1 SV=1 - [ROA0_HUMAN]	4
Rap guanine nucleotide exchange factor 2 OS=Homo sapiens GN=RAPGEF2 PE=1 SV=1 - [RPGF2_HUMAN]	4
40S ribosomal protein S4, X isoform OS=Homo sapiens GN=RPS4X PE=1 SV=2 - [RS4X_HUMAN]	4
Isoform 3 of Reticulon-3 OS=Homo sapiens GN=RTN3 - [RTN3_HUMAN]	4
Isoform 3 of Reticulon-4 OS=Homo sapiens GN=RTN4 - [RTN4_HUMAN]	4
Isoform 4 of Reticulon-4 OS=Homo sapiens GN=RTN4 - [RTN4_HUMAN]	4
Protein S100-A8 OS=Homo sapiens GN=S100A8 PE=1 SV=1 - [S10A8_HUMAN]	4
Saccharopine dehydrogenase-like oxidoreductase OS=Homo sapiens GN=SCCPDH PE=1 SV=1 - [SCPDL_HUMAN]	4
Tyrosine-protein phosphatase non-receptor type substrate 1 OS=Homo sapiens GN=SIRPA PE=1 SV=2 - [SHPS1_HUMAN]	4
Small nuclear ribonucleoprotein Sm D2 OS=Homo sapiens GN=SNRPD2 PE=1 SV=1 - [SMD2_HUMAN]	4
Sorting nexin-4 OS=Homo sapiens GN=SNX4 PE=1 SV=1 - [SNX4_HUMAN]	4
Serine/threonine-protein kinase 32C OS=Homo sapiens GN=STK32C PE=1 SV=1 - [ST32C_HUMAN]	4
Bifunctional glutamate/proline--tRNA ligase OS=Homo sapiens GN=EPRS PE=1 SV=5 - [SYEP_HUMAN]	4
Tyrosine--tRNA ligase, cytoplasmic OS=Homo sapiens GN=YARS PE=1 SV=4 - [SYYC_HUMAN]	4
T-complex protein 11-like protein 1 OS=Homo sapiens GN=TCP11L1 PE=1 SV=1 - [T11L1_HUMAN]	4
Isoform 2 of Tubulin alpha chain-like 3 OS=Homo sapiens GN=TUBAL3 - [TBAL3_HUMAN]	4
Isoform 2 of Transcription intermediary factor 1-beta OS=Homo sapiens GN=TRIM28 - [TIF1B_HUMAN]	4
Talin-1 OS=Homo sapiens GN=TLN1 PE=1 SV=3 - [TLN1_HUMAN]	4
V-type proton ATPase subunit E 1 OS=Homo sapiens GN=ATP6V1E1 PE=1 SV=1 - [VATE1_HUMAN]	4
WD repeat-containing protein 13 OS=Homo sapiens GN=WDR13 PE=1 SV=2 - [WDR13_HUMAN]	4
Isoform A3 of Tight junction protein ZO-2 OS=Homo sapiens GN=TJP2 - [ZO2_HUMAN]	4
Transcription elongation factor B (SIII), polypeptide 2 (18kDa, elongin B), isoform CRA_b OS=Homo sapiens GN=TCEB2 PE=1 SV=1 - [B8ZZU8_HUMAN]	4
Isoform 2 of Dihydrolipoyl dehydrogenase, mitochondrial OS=Homo sapiens GN=DLD - [DLDH_HUMAN]	4
Isoform 2 of Coiled-coil domain-containing protein 92 OS=Homo sapiens GN=CCDC92 - [CCD92_HUMAN]	4
High mobility group protein B1 OS=Homo sapiens GN=HMGB1 PE=1 SV=1 - [Q5T7C4_HUMAN]	4
Isoform 2 of Trifunctional enzyme subunit beta, mitochondrial OS=Homo sapiens GN=HADHB - [ECHB_HUMAN]	4

COP9 signalosome complex subunit 1 OS=Homo sapiens GN=GPS1 PE=1 SV=2 - [C9JFE4_HUMAN]	4
Malate dehydrogenase OS=Homo sapiens GN=MDH2 PE=1 SV=1 - [G3XAL0_HUMAN]	4
Ankyrin repeat and MYND domain containing 2, isoform CRA_c OS=Homo sapiens GN=ANKMY2 PE=1 SV=1 - [G3V0G5_HUMAN]	4
Single-stranded DNA-binding protein, mitochondrial (Fragment) OS=Homo sapiens GN=SSBP1 PE=1 SV=1 - [C9K0U8_HUMAN]	4
26S protease regulatory subunit 6A OS=Homo sapiens GN=PSMC3 PE=1 SV=1 - [E9PM69_HUMAN]	4
Rho GTPase-activating protein 44 OS=Homo sapiens GN=ARHGAP44 PE=1 SV=2 - [F5H6L3_HUMAN]	4
Isoform 3 of Excitatory amino acid transporter 2 OS=Homo sapiens GN=SLC1A2 - [EAA2_HUMAN]	4
NADH dehydrogenase [ubiquinone] iron-sulfur protein 8, mitochondrial (Fragment) OS=Homo sapiens GN=NDUFS8 PE=1 SV=1 - [E9PPW7_HUMAN]	4
Isoform 2 of Thioredoxin-dependent peroxide reductase, mitochondrial OS=Homo sapiens GN=PRDX3 - [PRDX3_HUMAN]	4
Sorcin OS=Homo sapiens GN=SRI PE=1 SV=1 - [C9J0K6_HUMAN]	4
Protein transport protein Sec23B (Fragment) OS=Homo sapiens GN=SEC23B PE=1 SV=1 - [Q5QPE2_HUMAN]	4
Isoform 2 of 60S ribosomal protein L18 OS=Homo sapiens GN=RPL18 - [RL18_HUMAN]	4
60S ribosomal protein L30 (Fragment) OS=Homo sapiens GN=RPL30 PE=1 SV=1 - [E5RI99_HUMAN]	4
Histone-binding protein RBBP7 OS=Homo sapiens GN=RBBP7 PE=1 SV=1 - [E9PC52_HUMAN]	4
Polyubiquitin-C (Fragment) OS=Homo sapiens GN=UBC PE=1 SV=1 - [F5H265_HUMAN]	4
Fructose-bisphosphate aldolase OS=Homo sapiens GN=ALDOC PE=1 SV=1 - [A8MVZ9_HUMAN]	4
60S acidic ribosomal protein P0 (Fragment) OS=Homo sapiens GN=RPLP0 PE=1 SV=1 - [F8VPE8_HUMAN]	4
NADH dehydrogenase [ubiquinone] 1 beta subcomplex subunit 10 OS=Homo sapiens GN=NDUFB10 PE=1 SV=1 - [H3BPJ9_HUMAN]	4
Microtubule-associated protein RP/EB family member 2 (Fragment) OS=Homo sapiens GN=MAPRE2 PE=1 SV=1 - [M0QX52_HUMAN]	4
40S ribosomal protein S16 OS=Homo sapiens GN=RPS16 PE=1 SV=1 - [M0R3H0_HUMAN]	4
Dystrobrevin alpha OS=Homo sapiens GN=DTNA PE=1 SV=1 - [M0R021_HUMAN]	4
Ras-related protein Rab-6A (Fragment) OS=Homo sapiens GN=RAB6A PE=1 SV=1 - [H7BYW1_HUMAN]	4
UPF0568 protein C14orf166 OS=Homo sapiens GN=C14orf166 PE=1 SV=1 - [G3V4C6_HUMAN]	4
60S ribosomal protein L9 (Fragment) OS=Homo sapiens GN=RPL9 PE=1 SV=1 - [H0Y9V9_HUMAN]	4
Protein disulfide-isomerase A3 (Fragment) OS=Homo sapiens GN=PDIA3 PE=1 SV=1 - [H7BZJ3_HUMAN]	4
Serum albumin (Fragment) OS=Homo sapiens GN=ALB PE=1 SV=1 - [H0YA55_HUMAN]	4
Heterogeneous nuclear ribonucleoproteins C1/C2 (Fragment) OS=Homo sapiens GN=HNRNPC PE=1 SV=1 - [G3V5X6_HUMAN]	4
SRSF protein kinase 2 (Fragment) OS=Homo sapiens GN=SRPK2 PE=1 SV=1 - [H7C5L6_HUMAN]	4
Ganglioside-induced differentiation-associated protein 1-like 1 (Fragment) OS=Homo sapiens GN=GDAP1L1 PE=1 SV=1 - [H0Y6A7_HUMAN]	4
Aldo-keto reductase family 1 member C1 (Fragment) OS=Homo sapiens GN=AKR1C1 PE=1 SV=1 - [H0Y804_HUMAN]	4
Kinesin light chain 1 OS=Homo sapiens GN=KLC1 PE=1 SV=1 - [G5E9S8_HUMAN]	4
Retinol dehydrogenase 11 (Fragment) OS=Homo sapiens GN=RDH11 PE=1 SV=1 - [G3V2G6_HUMAN]	4
DnaJ homolog subfamily B member 1 (Fragment) OS=Homo sapiens GN=DNAJB1 PE=1 SV=1 - [M0R080_HUMAN]	4
Heterogeneous nuclear ribonucleoprotein D0 (Fragment) OS=Homo sapiens GN=HNRNPD PE=1 SV=1 - [H0YA96_HUMAN]	4
60S ribosomal protein L18a (Fragment) OS=Homo sapiens GN=RPL18A PE=1 SV=1 - [M0R3D6_HUMAN]	4
Regulator of G-protein-signaling 7 (Fragment) OS=Homo sapiens GN=RGS7 PE=1 SV=1 - [Q5T3H5_HUMAN]	4
Tubulin-folding cofactor B OS=Homo sapiens GN=TBCB PE=1 SV=1 - [K7EK42_HUMAN]	4
SH3 and multiple ankyrin repeat domains protein 3 OS=Homo sapiens GN=SHANK3 PE=1 SV=3 - [SHAN3_HUMAN]	4
Isoform 2 of Proliferation-associated protein 2G4 OS=Homo sapiens GN=PA2G4 - [PA2G4_HUMAN]	4
WD repeat-containing protein 7 OS=Homo sapiens GN=WDR7 PE=1 SV=1 - [A2RRE0_HUMAN]	4
Heterogeneous nuclear ribonucleoprotein Q OS=Homo sapiens GN=SYNCRIP PE=1 SV=1 - [B7Z645_HUMAN]	4
Protein-tyrosine-phosphatase OS=Homo sapiens GN=PTPRS PE=1 SV=2 - [G8JL96_HUMAN]	4
Mitochondrial carrier homolog 2 (Fragment) OS=Homo sapiens GN=MTCH2 PE=1 SV=4 - [E9PIE4_HUMAN]	4
Synemin OS=Homo sapiens GN=SYNM PE=1 SV=2 - [C9JIE4_HUMAN]	4
ATPase ASNA1 OS=Homo sapiens GN=ASNA1 PE=1 SV=1 - [A0A087WXS7_HUMAN]	4
Arf-GAP with GTPase, ANK repeat and PH domain-containing protein 1 OS=Homo sapiens GN=AGAP1 PE=1 SV=1 - [A0A087X1U1_HUMAN]	4
Calcium-binding protein 39 OS=Homo sapiens GN=CAB39 PE=1 SV=1 - [A0A087X0K1_HUMAN]	4
Isoform 2 of 4F2 cell-surface antigen heavy chain OS=Homo sapiens GN=SLC3A2 - [4F2_HUMAN]	3
5'-AMP-activated protein kinase catalytic subunit alpha-1 OS=Homo sapiens GN=PRKAA1 PE=1 SV=4 - [AAPK1_HUMAN]	3
Alanyl-tRNA editing protein Aarsd1 OS=Homo sapiens GN=AARSD1 PE=1 SV=2 - [AASD1_HUMAN]	3
Acyl-CoA dehydrogenase family member 9, mitochondrial OS=Homo sapiens GN=ACAD9 PE=1 SV=1 - [ACAD9_HUMAN]	3
Isoform 2 of Very long-chain specific acyl-CoA dehydrogenase, mitochondrial OS=Homo sapiens GN=ACADVL - [ACADV_HUMAN]	3
Isoform 6 of Agrin OS=Homo sapiens GN=AGRN - [AGRIN_HUMAN]	3

Neuroblast differentiation-associated protein AHNAK OS=Homo sapiens GN=AHNAK PE=1 SV=2 - [AHNK_HUMAN]	3
AH receptor-interacting protein OS=Homo sapiens GN=AIP PE=1 SV=2 - [AIP_HUMAN]	3
Aldehyde dehydrogenase, mitochondrial OS=Homo sapiens GN=ALDH2 PE=1 SV=2 - [ALDH2_HUMAN]	3
Annexin A1 OS=Homo sapiens GN=ANXA1 PE=1 SV=2 - [ANXA1_HUMAN]	3
Isoform 3 of AP-2 complex subunit beta OS=Homo sapiens GN=AP2B1 - [AP2B1_HUMAN]	3
Isoform 4 of AP-3 complex subunit delta-1 OS=Homo sapiens GN=AP3D1 - [AP3D1_HUMAN]	3
Isoform 7 of Alpha-tubulin N-acetyltransferase 1 OS=Homo sapiens GN=ATAT1 - [ATAT_HUMAN]	3
Isoform 6 of BAI1-associated protein 3 OS=Homo sapiens GN=BAIAP3 - [BAIP3_HUMAN]	3
Isoform 2 of Brain acid soluble protein 1 OS=Homo sapiens GN=BASP1 - [BASP1_HUMAN]	3
Complement component 1 Q subcomponent-binding protein, mitochondrial OS=Homo sapiens GN=C1QBP PE=1 SV=1 - [C1QBP_HUMAN]	3
Isoform 4 of Voltage-dependent L-type calcium channel subunit beta-4 OS=Homo sapiens GN=CACNB4 - [CACB4_HUMAN]	3
F-actin-capping protein subunit alpha-1 OS=Homo sapiens GN=CAPZA1 PE=1 SV=3 - [CAZA1_HUMAN]	3
Isoform 3 of Cyclin-dependent kinase 14 OS=Homo sapiens GN=CDK14 - [CDK14_HUMAN]	3
Cyclin-dependent kinase 18 OS=Homo sapiens GN=CDK18 PE=1 SV=3 - [CDK18_HUMAN]	3
MICOS complex subunit MIC25 OS=Homo sapiens GN=CHCHD6 PE=1 SV=1 - [MIC25_HUMAN]	3
Claudin-11 OS=Homo sapiens GN=CLDN11 PE=1 SV=2 - [CLD11_HUMAN]	3
Isoform 2 of Clathrin heavy chain 1 OS=Homo sapiens GN=CLTC - [CLH1_HUMAN]	3
Isoform 2 of Collagen alpha-1(IV) chain OS=Homo sapiens GN=COL4A1 - [CO4A1_HUMAN]	3
Coatomer subunit delta OS=Homo sapiens GN=ARCN1 PE=1 SV=1 - [COPD_HUMAN]	3
Coronin-1A OS=Homo sapiens GN=CORO1A PE=1 SV=4 - [COR1A_HUMAN]	3
Isoform 2 of COP9 signalosome complex subunit 8 OS=Homo sapiens GN=COPS8 - [CSN8_HUMAN]	3
Isoform 4 of Catenin delta-1 OS=Homo sapiens GN=CTNND1 - [CTND1_HUMAN]	3
Cytochrome c1, heme protein, mitochondrial OS=Homo sapiens GN=CYC1 PE=1 SV=3 - [CY1_HUMAN]	3
Isoform 5 of Dynactin subunit 1 OS=Homo sapiens GN=DCTN1 - [DCTN1_HUMAN]	3
ATP-dependent RNA helicase A OS=Homo sapiens GN=DHX9 PE=1 SV=4 - [DHX9_HUMAN]	3
Isoform 8 of Dystrophin OS=Homo sapiens GN=DMD - [DMD_HUMAN]	3
Isoform 2 of DnaJ homolog subfamily A member 1 OS=Homo sapiens GN=DNAJA1 - [DNJA1_HUMAN]	3
Dynein light chain 1, cytoplasmic OS=Homo sapiens GN=DYNLL1 PE=1 SV=1 - [DYL1_HUMAN]	3
Isoform 4 of Epidermal growth factor receptor substrate 15-like 1 OS=Homo sapiens GN=EPS15L1 - [EP15R_HUMAN]	3
Isoform 2 of Redox-regulatory protein FAM213A OS=Homo sapiens GN=FAM213A - [F213A_HUMAN]	3
Isoform 2 of Protein-tyrosine kinase 2-beta OS=Homo sapiens GN=PTK2B - [FAK2_HUMAN]	3
Isoform Gamma-A of Fibrinogen gamma chain OS=Homo sapiens GN=FGG - [FIBG_HUMAN]	3
Isoform 12 of Fibronectin OS=Homo sapiens GN=FN1 - [FINC_HUMAN]	3
Fascin OS=Homo sapiens GN=FSCN1 PE=1 SV=3 - [FSCN1_HUMAN]	3
Rab GDP dissociation inhibitor alpha OS=Homo sapiens GN=GDI1 PE=1 SV=2 - [GDIA_HUMAN]	3
78 kDa glucose-regulated protein OS=Homo sapiens GN=HSPA5 PE=1 SV=2 - [GRP78_HUMAN]	3
Glutamate--cysteine ligase catalytic subunit OS=Homo sapiens GN=GCLC PE=1 SV=2 - [GSH1_HUMAN]	3
Histone H1x OS=Homo sapiens GN=H1FX PE=1 SV=1 - [H1X_HUMAN]	3
Histone H2A type 1-H OS=Homo sapiens GN=HIST1H2AH PE=1 SV=3 - [H2A1H_HUMAN]	3
Putative histone H2B type 2-D OS=Homo sapiens GN=HIST2H2BD PE=5 SV=3 - [H2B2D_HUMAN]	3
Hemoglobin subunit alpha OS=Homo sapiens GN=HBA1 PE=1 SV=2 - [HBA_HUMAN]	3
Serine protease HTRA1 OS=Homo sapiens GN=HTRA1 PE=1 SV=1 - [HTRA1_HUMAN]	3
Eukaryotic translation initiation factor 2 subunit 3 OS=Homo sapiens GN=EIF2S3 PE=1 SV=3 - [IF2G_HUMAN]	3
Inositol-trisphosphate 3-kinase A OS=Homo sapiens GN=ITPKA PE=1 SV=1 - [IP3KA_HUMAN]	3
Isoform 3 of Adenylate kinase isoenzyme 5 OS=Homo sapiens GN=AK5 - [KAD5_HUMAN]	3
BTB/POZ domain-containing protein KCTD12 OS=Homo sapiens GN=KCTD12 PE=1 SV=1 - [KCD12_HUMAN]	3
Calcium/calmodulin-dependent protein kinase kinase 1 OS=Homo sapiens GN=CAMKK1 PE=1 SV=2 - [KKCC1_HUMAN]	3
Isoform 6 of Calcium/calmodulin-dependent protein kinase kinase 2 OS=Homo sapiens GN=CAMKK2 - [KKCC2_HUMAN]	3
Ribosomal protein S6 kinase alpha-3 OS=Homo sapiens GN=RPS6KA3 PE=1 SV=1 - [KS6A3_HUMAN]	3
Myeloid leukemia factor 2 OS=Homo sapiens GN=MLF2 PE=1 SV=1 - [MLF2_HUMAN]	3
Isoform 3 of Metaxin-3 OS=Homo sapiens GN=MTX3 - [MTX3_HUMAN]	3
Methylmalonyl-CoA mutase, mitochondrial OS=Homo sapiens GN=MUT PE=1 SV=4 - [MUTA_HUMAN]	3
Isoform 2 of NADH-cytochrome b5 reductase 3 OS=Homo sapiens GN=CYB5R3 - [NB5R3_HUMAN]	3

Cytoplasmic protein NCK2 OS=Homo sapiens GN=NCK2 PE=1 SV=2 - [NCK2_HUMAN]	3
NADH dehydrogenase [ubiquinone] 1 alpha subcomplex subunit 5 OS=Homo sapiens GN=NDUFA5 PE=1 SV=3 - [NDUA5_HUMAN]	3
NADH dehydrogenase [ubiquinone] flavoprotein 2, mitochondrial OS=Homo sapiens GN=NDUFV2 PE=1 SV=2 - [NDUV2_HUMAN]	3
RNA-binding protein Nova-2 OS=Homo sapiens GN=NOVA2 PE=1 SV=1 - [NOVA2_HUMAN]	3
Isoform TrkB-T-TK of BDNF/NT-3 growth factors receptor OS=Homo sapiens GN=NTRK2 - [NTRK2_HUMAN]	3
Obg-like ATPase 1 OS=Homo sapiens GN=OLA1 PE=1 SV=2 - [OLA1_HUMAN]	3
Phosphatidylinositol 4-kinase alpha OS=Homo sapiens GN=PI4KA PE=1 SV=3 - [PI4KA_HUMAN]	3
Plexin-A1 OS=Homo sapiens GN=PLXNA1 PE=1 SV=3 - [PLXA1_HUMAN]	3
Plexin-B2 OS=Homo sapiens GN=PLXNB2 PE=1 SV=3 - [PLXB2_HUMAN]	3
Phosphomevalonate kinase OS=Homo sapiens GN=PMVK PE=1 SV=3 - [PMVK_HUMAN]	3
Peptidyl-prolyl cis-trans isomerase-like 1 OS=Homo sapiens GN=PPIL1 PE=1 SV=1 - [PPIL1_HUMAN]	3
Proline synthase co-transcribed bacterial homolog protein OS=Homo sapiens GN=PROSC PE=1 SV=1 - [PROSC_HUMAN]	3
Isoform 2 of 26S protease regulatory subunit 6B OS=Homo sapiens GN=PSMC4 - [PRS6B_HUMAN]	3
Putative peptidyl-tRNA hydrolase PTRHD1 OS=Homo sapiens GN=PTRHD1 PE=1 SV=1 - [PTRD1_HUMAN]	3
Ras-related protein Rap-2a OS=Homo sapiens GN=RAP2A PE=1 SV=1 - [RAP2A_HUMAN]	3
Ras-related protein Rap-2b OS=Homo sapiens GN=RAP2B PE=1 SV=1 - [RAP2B_HUMAN]	3
Ras-related protein Rab-22A OS=Homo sapiens GN=RAB22A PE=1 SV=2 - [RB22A_HUMAN]	3
Isoform 2 of Regulator of nonsense transcripts 1 OS=Homo sapiens GN=UPF1 - [RENT1_HUMAN]	3
Rab11 family-interacting protein 5 OS=Homo sapiens GN=RAB11FIP5 PE=1 SV=1 - [RFIP5_HUMAN]	3
Transforming protein RhoA OS=Homo sapiens GN=RHOA PE=1 SV=1 - [RHOA_HUMAN]	3
60S ribosomal protein L12 OS=Homo sapiens GN=RPL12 PE=1 SV=1 - [RL12_HUMAN]	3
60S ribosomal protein L26 OS=Homo sapiens GN=RPL26 PE=1 SV=1 - [RL26_HUMAN]	3
60S ribosomal protein L3 OS=Homo sapiens GN=RPL3 PE=1 SV=2 - [RL3_HUMAN]	3
Isoform 2 of Rabphilin-3A OS=Homo sapiens GN=RPH3A - [RP3A_HUMAN]	3
Dolichyl-diphosphooligosaccharide--protein glycosyltransferase subunit 1 OS=Homo sapiens GN=RPN1 PE=1 SV=1 - [RPN1_HUMAN]	3
40S ribosomal protein S18 OS=Homo sapiens GN=RPS18 PE=1 SV=3 - [RS18_HUMAN]	3
Serum amyloid P-component OS=Homo sapiens GN=APCS PE=1 SV=2 - [SAMP_HUMAN]	3
Vesicle-trafficking protein SEC22b OS=Homo sapiens GN=SEC22B PE=1 SV=4 - [SC22B_HUMAN]	3
Isoform 6 of Calcium-binding mitochondrial carrier protein SCaMC-2 OS=Homo sapiens GN=SLC25A25 - [SCMC2_HUMAN]	3
Isoform 2 of Shootin-1 OS=Homo sapiens GN=KIAA1598 - [SHOT1_HUMAN]	3
Isoform 4 of NAD-dependent protein deacylase sirtuin-5, mitochondrial OS=Homo sapiens GN=SIRT5 - [SIR5_HUMAN]	3
Staphylococcal nuclease domain-containing protein 1 OS=Homo sapiens GN=SND1 PE=1 SV=1 - [SND1_HUMAN]	3
Beta-1-syntrophin OS=Homo sapiens GN=SNTB1 PE=1 SV=3 - [SNTB1_HUMAN]	3
Succinyl-CoA ligase [GDP-forming] subunit beta, mitochondrial OS=Homo sapiens GN=SUCLG2 PE=1 SV=2 - [SUCB2_HUMAN]	3
Probable leucine--tRNA ligase, mitochondrial OS=Homo sapiens GN=LARS2 PE=1 SV=2 - [SYLM_HUMAN]	3
Synapsin-3 OS=Homo sapiens GN=SYN3 PE=1 SV=2 - [SYN3_HUMAN]	3
Isoform 3 of Synaptopodin OS=Homo sapiens GN=SYNPO - [SYNPO_HUMAN]	3
Synaptophysin OS=Homo sapiens GN=SYP PE=1 SV=3 - [SYPH_HUMAN]	3
Protein-glutamine gamma-glutamyltransferase 2 OS=Homo sapiens GN=TGM2 PE=1 SV=2 - [TGM2_HUMAN]	3
Isoform 3 of Mitochondrial import inner membrane translocase subunit TIM50 OS=Homo sapiens GN=TIMM50 - [TIM50_HUMAN]	3
Metalloproteinase inhibitor 3 OS=Homo sapiens GN=TIMP3 PE=1 SV=2 - [TIMP3_HUMAN]	3
Isoform 2 of Tumor protein D52 OS=Homo sapiens GN=TPD52 - [TPD52_HUMAN]	3
Isoform 2 of Thioredoxin domain-containing protein 5 OS=Homo sapiens GN=TXNDC5 - [TXND5_HUMAN]	3
Isoform 2 of Mitochondrial uncoupling protein 4 OS=Homo sapiens GN=SLC25A27 - [UCP4_HUMAN]	3
Vesicle-associated membrane protein-associated protein A OS=Homo sapiens GN=VAPA PE=1 SV=3 - [VAPA_HUMAN]	3
Vacuolar protein sorting-associated protein 4A OS=Homo sapiens GN=VPS4A PE=1 SV=1 - [VPS4A_HUMAN]	3
Vitronectin OS=Homo sapiens GN=VTN PE=1 SV=1 - [VTNC_HUMAN]	3
Isoform 6 of Drebrin-like protein OS=Homo sapiens GN=DBNL - [DBNL_HUMAN]	3
Isoform 2 of Methionine--tRNA ligase, cytoplasmic OS=Homo sapiens GN=MARS - [SYMC_HUMAN]	3
Citrate synthase, mitochondrial OS=Homo sapiens GN=CS PE=1 SV=1 - [B4DJV2_HUMAN]	3
Isoform 3 of Glutamate dehydrogenase 1, mitochondrial OS=Homo sapiens GN=GLUD1 - [DHE3_HUMAN]	3
Isoform 2 of Cytoplasmic dynein 1 light intermediate chain 2 OS=Homo sapiens GN=DYNC1LI2 - [DC1L2_HUMAN]	3
Profilin-2 OS=Homo sapiens GN=PFN2 PE=1 SV=1 - [C9J0J7_HUMAN]	3

Isoform 2 of 26S protease regulatory subunit 4 OS=Homo sapiens GN=PSMC1 - [PRS4_HUMAN]	3
Isoform 2 of Annexin A11 OS=Homo sapiens GN=ANXA11 - [ANX11_HUMAN]	3
Flotillin-2 OS=Homo sapiens GN=FLOT2 PE=1 SV=1 - [J3QLD9_HUMAN]	3
Tropomodulin 2 (Neuronal), isoform CRA_a OS=Homo sapiens GN=TMOD2 PE=1 SV=1 - [G5EA42_HUMAN]	3
Cofilin 1 (Non-muscle), isoform CRA_a OS=Homo sapiens GN=CFL1 PE=1 SV=1 - [G3V1A4_HUMAN]	3
Protein transport protein Sec24C OS=Homo sapiens GN=SEC24C PE=1 SV=1 - [G5EA31_HUMAN]	3
Vesicle-associated membrane protein 2 OS=Homo sapiens GN=VAMP2 PE=4 SV=2 - [J3QRU4_HUMAN]	3
X-ray repair cross-complementing protein 6 OS=Homo sapiens GN=XRCC6 PE=1 SV=1 - [B1AHC9_HUMAN]	3
Glutathione S-transferase kappa 1 OS=Homo sapiens GN=GSTK1 PE=1 SV=1 - [E9PFN5_HUMAN]	3
Phosphatidylinositide phosphatase SAC1 OS=Homo sapiens GN=SACM1L PE=1 SV=1 - [E9PGZ4_HUMAN]	3
Caprin-1 (Fragment) OS=Homo sapiens GN=CAPRIN1 PE=1 SV=1 - [E9PLA9_HUMAN]	3
Glutathione S-transferase Mu 2 (Fragment) OS=Homo sapiens GN=GSTM2 PE=1 SV=2 - [E9PLF1_HUMAN]	3
Isoform 3 of Receptor-type tyrosine-protein phosphatase zeta OS=Homo sapiens GN=PTPRZ1 - [PTPRZ_HUMAN]	3
ADP-ribosylation factor 4 (Fragment) OS=Homo sapiens GN=ARF4 PE=1 SV=1 - [C9JPM4_HUMAN]	3
Copine-1 (Fragment) OS=Homo sapiens GN=CPNE1 PE=1 SV=1 - [Q5JX45_HUMAN]	3
COP9 signalosome complex subunit 7a (Fragment) OS=Homo sapiens GN=COPS7A PE=1 SV=1 - [F5H7C6_HUMAN]	3
26S proteasome non-ATPase regulatory subunit 4 (Fragment) OS=Homo sapiens GN=PSMD4 PE=1 SV=1 - [A6PVX3_HUMAN]	3
Hyaluronan and proteoglycan link protein 2 (Fragment) OS=Homo sapiens GN=HAPLN2 PE=1 SV=1 - [Q5T3J1_HUMAN]	3
Ubiquitin-like modifier-activating enzyme 5 OS=Homo sapiens GN=UBA5 PE=1 SV=1 - [E7EWE1_HUMAN]	3
Isoform 3 of Pyruvate dehydrogenase protein X component, mitochondrial OS=Homo sapiens GN=PDHX - [ODPX_HUMAN]	3
60S ribosomal protein L14 OS=Homo sapiens GN=RPL14 PE=1 SV=1 - [E7EPB3_HUMAN]	3
60S ribosomal protein L24 OS=Homo sapiens GN=RPL24 PE=1 SV=1 - [C9JXB8_HUMAN]	3
Ras-related protein Rab-35 (Fragment) OS=Homo sapiens GN=RAB35 PE=1 SV=1 - [F5H157_HUMAN]	3
DnaJ homolog subfamily B member 2 (Fragment) OS=Homo sapiens GN=DNAJB2 PE=1 SV=1 - [C9JRD2_HUMAN]	3
NudC domain-containing protein 2 OS=Homo sapiens GN=NUDCD2 PE=1 SV=1 - [E5RFP0_HUMAN]	3
Pyrroline-5-carboxylate reductase 3 OS=Homo sapiens GN=PYCRL PE=1 SV=2 - [B5MD87_HUMAN]	3
CLIP-associating protein 2 OS=Homo sapiens GN=CLASP2 PE=1 SV=2 - [E3W994_HUMAN]	3
Rho-related GTP-binding protein RhoC (Fragment) OS=Homo sapiens GN=RHOC PE=1 SV=1 - [E9PQH6_HUMAN]	3
ADP-ribosylation factor GTPase-activating protein 1 (Fragment) OS=Homo sapiens GN=ARFGAP1 PE=1 SV=2 - [E5RHT6_HUMAN]	3
Ras-related protein Rab-8B (Fragment) OS=Homo sapiens GN=RAB8B PE=1 SV=1 - [H0YNE9_HUMAN]	3
Ketimine reductase mu-crystallin (Fragment) OS=Homo sapiens GN=CRYM PE=1 SV=1 - [I3L325_HUMAN]	3
Dephospho-CoA kinase domain-containing protein (Fragment) OS=Homo sapiens GN=DCAKD PE=1 SV=1 - [K7ESP4_HUMAN]	3
Guanine nucleotide-binding protein subunit beta-2-like 1 (Fragment) OS=Homo sapiens GN=GNB2L1 PE=1 SV=1 - [H0YAF8_HUMAN]	3
Apolipoprotein O (Fragment) OS=Homo sapiens GN=APOO PE=1 SV=1 - [H7C1U8_HUMAN]	3
Arf-GAP with dual PH domain-containing protein 1 (Fragment) OS=Homo sapiens GN=ADAP1 PE=1 SV=2 - [H7C324_HUMAN]	3
40S ribosomal protein S2 (Fragment) OS=Homo sapiens GN=RPS2 PE=1 SV=1 - [H0YEN5_HUMAN]	3
Serine-threonine kinase receptor-associated protein (Fragment) OS=Homo sapiens GN=STRAP PE=1 SV=1 - [H0YH33_HUMAN]	3
Transcription elongation factor B polypeptide 1 OS=Homo sapiens GN=TCEB1 PE=1 SV=1 - [R4GMY8_HUMAN]	3
Neurofascin (Fragment) OS=Homo sapiens GN=NFASC PE=1 SV=1 - [H7BY57_HUMAN]	3
Receptor-type tyrosine-protein phosphatase F (Fragment) OS=Homo sapiens GN=PTPRF PE=1 SV=1 - [H0Y380_HUMAN]	3
Guanine nucleotide-binding protein subunit beta-5 OS=Homo sapiens GN=GNB5 PE=1 SV=1 - [H0YNW7_HUMAN]	3
cAMP-dependent protein kinase type I-beta regulatory subunit (Fragment) OS=Homo sapiens GN=PRKAR1B PE=1 SV=1 - [H7BYW5_HUMAN]	3
Glutathione peroxidase (Fragment) OS=Homo sapiens GN=GPX4 PE=1 SV=1 - [K7ERP4_HUMAN]	3
40S ribosomal protein S5 OS=Homo sapiens GN=RPS5 PE=1 SV=1 - [M0QZN2_HUMAN]	3
Chromosome 6 open reading frame 55, isoform CRA_b OS=Homo sapiens GN=VTA1 PE=1 SV=1 - [A0A087WY55_HUMAN]	3
MAP/microtubule affinity-regulating kinase 3 OS=Homo sapiens GN=MARK3 PE=1 SV=1 - [A0A0A0MST9_HUMAN]	3
Phosphoinositide phospholipase C OS=Homo sapiens GN=PLCB1 PE=1 SV=1 - [A0A087WW73_HUMAN]	3
RNA-binding protein Raly (Fragment) OS=Homo sapiens GN=RALY PE=1 SV=4 - [Q5QPM0_HUMAN]	3
G-protein-signaling modulator 1 OS=Homo sapiens GN=GPSM1 PE=1 SV=1 - [A0A087WVF5_HUMAN]	3
Heat shock protein 105 kDa OS=Homo sapiens GN=HSPH1 PE=1 SV=1 - [A0A0A0MSM0_HUMAN]	3
Elongation factor Ts (Fragment) OS=Homo sapiens GN=TSFM PE=1 SV=4 - [C9JT21_HUMAN]	3
Thy-1 membrane glycoprotein (Fragment) OS=Homo sapiens GN=THY1 PE=1 SV=4 - [E9PIM6_HUMAN]	3
Non-POU domain-containing octamer-binding protein (Fragment) OS=Homo sapiens GN=NONO PE=1 SV=4 - [H7C367_HUMAN]	3

Tyrosine-protein phosphatase non-receptor type 9 OS=Homo sapiens GN=PTPN9 PE=1 SV=1 - [A0A0A0MR09_HUMAN]	3
C-terminal-binding protein 1 (Fragment) OS=Homo sapiens GN=CTBP1 PE=1 SV=4 - [D6RAX2_HUMAN]	3
Opalin OS=Homo sapiens GN=OPALIN PE=1 SV=1 - [A0A0A0MTN4_HUMAN]	3
MICOS complex subunit MIC27 OS=Homo sapiens GN=APOOL PE=1 SV=1 - [A0A087WYF7_HUMAN]	3
Protein LOC102724023 OS=Homo sapiens GN=LOC102724023 PE=4 SV=1 - [A0A096LNH5_HUMAN]	3
TAR DNA-binding protein 43 (Fragment) OS=Homo sapiens GN=TARDBP PE=1 SV=1 - [A0A087WXQ5_HUMAN]	3
5'-AMP-activated protein kinase catalytic subunit alpha-2 OS=Homo sapiens GN=PRKAA2 PE=1 SV=1 - [A0A087WXX9_HUMAN]	3
SLIT-ROBO Rho GTPase-activating protein 3 OS=Homo sapiens GN=SRGAP3 PE=1 SV=1 - [A0A087WZH4_HUMAN]	3
CUGBP Elav-like family member 2 OS=Homo sapiens GN=CELF2 PE=1 SV=1 - [V9GYD9_HUMAN]	3
FSD1-like protein OS=Homo sapiens GN=FSD1L PE=4 SV=1 - [A0A0C4DG97_HUMAN]	3
Isoform 6 of Serine/threonine-protein phosphatase 2A 55 kDa regulatory subunit B beta isoform OS=Homo sapiens GN=PPP2R2B - [2ABB_HUMAN]	2
Actin-like protein 6B OS=Homo sapiens GN=ACTL6B PE=1 SV=1 - [ACL6B_HUMAN]	2
AFG3-like protein 2 OS=Homo sapiens GN=AFG3L2 PE=1 SV=2 - [AFG32_HUMAN]	2
Isoform 6 of Apoptosis-inducing factor 1, mitochondrial OS=Homo sapiens GN=AIFM1 - [AIFM1_HUMAN]	2
ADP-ribosylation factor 6 OS=Homo sapiens GN=ARF6 PE=1 SV=2 - [ARF6_HUMAN]	2
ADP-ribosylation factor-like protein 3 OS=Homo sapiens GN=ARL3 PE=1 SV=2 - [ARL3_HUMAN]	2
Actin-related protein 2/3 complex subunit 2 OS=Homo sapiens GN=ARPC2 PE=1 SV=1 - [ARPC2_HUMAN]	2
Sodium/potassium-transporting ATPase subunit beta-2 OS=Homo sapiens GN=ATP1B2 PE=1 SV=3 - [AT1B2_HUMAN]	2
Isoform E of Plasma membrane calcium-transporting ATPase 1 OS=Homo sapiens GN=ATP2B1 - [AT2B1_HUMAN]	2
Isoform 3 of Ethanolamine-phosphate phospho-lyase OS=Homo sapiens GN=ETNPPL - [AT2L1_HUMAN]	2
ATP synthase subunit delta, mitochondrial OS=Homo sapiens GN=ATP5D PE=1 SV=2 - [ATPD_HUMAN]	2
Isoform 4 of ATP synthase subunit f, mitochondrial OS=Homo sapiens GN=ATP5J2 - [ATPK_HUMAN]	2
Isoform 2 of Ataxin-10 OS=Homo sapiens GN=ATXN10 - [ATX10_HUMAN]	2
Serine/threonine-protein kinase B-raf OS=Homo sapiens GN=BRAF PE=1 SV=4 - [BRAF_HUMAN]	2
Isoform 2 of Centrosomal protein of 170 kDa protein B OS=Homo sapiens GN=CEP170B - [C170B_HUMAN]	2
C2 domain-containing protein 2-like OS=Homo sapiens GN=C2CD2L PE=1 SV=3 - [C2C2L_HUMAN]	2
Isoform 2 of Coiled-coil and C2 domain-containing protein 1A OS=Homo sapiens GN=CC2D1A - [C2D1A_HUMAN]	2
Calmodulin OS=Homo sapiens GN=CALM1 PE=1 SV=2 - [CALM_HUMAN]	2
Isoform 2 of Calmodulin-regulated spectrin-associated protein 2 OS=Homo sapiens GN=CAMSAP2 - [CAMP2_HUMAN]	2
F-actin-capping protein subunit alpha-2 OS=Homo sapiens GN=CAPZA2 PE=1 SV=3 - [CAZA2_HUMAN]	2
Uncharacterized protein C2orf72 OS=Homo sapiens GN=C2orf72 PE=1 SV=2 - [CB072_HUMAN]	2
Coiled-coil domain-containing protein 6 OS=Homo sapiens GN=CCDC6 PE=1 SV=2 - [CCDC6_HUMAN]	2
Isoform 5 of CUGBP Elav-like family member 1 OS=Homo sapiens GN=CELF1 - [CELF1_HUMAN]	2
Cell cycle exit and neuronal differentiation protein 1 OS=Homo sapiens GN=CEND1 PE=2 SV=1 - [CEND_HUMAN]	2
Isoform 2 of E3 ubiquitin-protein ligase CHIP OS=Homo sapiens GN=STUB1 - [CHIP_HUMAN]	2
CDK5 regulatory subunit-associated protein 3 OS=Homo sapiens GN=CDK5RAP3 PE=1 SV=2 - [CK5P3_HUMAN]	2
Isoform 2 of Clavesin-1 OS=Homo sapiens GN=CLVS1 - [CLVS1_HUMAN]	2
Isoform 2 of Coronin-2B OS=Homo sapiens GN=CORO2B - [COR2B_HUMAN]	2
Cytochrome c oxidase subunit 5A, mitochondrial OS=Homo sapiens GN=COX5A PE=1 SV=2 - [COX5A_HUMAN]	2
Cytochrome c oxidase subunit 5B, mitochondrial OS=Homo sapiens GN=COX5B PE=1 SV=2 - [COX5B_HUMAN]	2
Copine-4 OS=Homo sapiens GN=CPNE4 PE=2 SV=1 - [CPNE4_HUMAN]	2
Crk-like protein OS=Homo sapiens GN=CRKL PE=1 SV=1 - [CRKL_HUMAN]	2
Cytochrome c oxidase subunit 6B1 OS=Homo sapiens GN=COX6B1 PE=1 SV=2 - [CX6B1_HUMAN]	2
Cystatin-C OS=Homo sapiens GN=CST3 PE=1 SV=1 - [CYTC_HUMAN]	2
Glutamate decarboxylase 2 OS=Homo sapiens GN=GAD2 PE=1 SV=1 - [DCE2_HUMAN]	2
Isoform 8 of Dehydrogenase/reductase SDR family member 4 OS=Homo sapiens GN=DHRS4 - [DHRS4_HUMAN]	2
GTP-binding protein Di-Ras1 OS=Homo sapiens GN=DIRAS1 PE=1 SV=1 - [DIRA1_HUMAN]	2
Disks large-associated protein 3 OS=Homo sapiens GN=DLGAP3 PE=1 SV=3 - [DLGP3_HUMAN]	2
DnaJ homolog subfamily A member 2 OS=Homo sapiens GN=DNAJA2 PE=1 SV=1 - [DNJA2_HUMAN]	2
Isoform 2 of DnaJ homolog subfamily A member 3, mitochondrial OS=Homo sapiens GN=DNAJA3 - [DNJA3_HUMAN]	2
DCC-interacting protein 13-alpha OS=Homo sapiens GN=APPL1 PE=1 SV=1 - [DP13A_HUMAN]	2
Dual specificity protein phosphatase 3 OS=Homo sapiens GN=DUSP3 PE=1 SV=1 - [DUS3_HUMAN]	2
Eukaryotic translation initiation factor 3 subunit I OS=Homo sapiens GN=EIF3I PE=1 SV=1 - [EIF3I_HUMAN]	2

Isoform 2 of ELAV-like protein 2 OS=Homo sapiens GN=ELAVL2 - [ELAV2_HUMAN]	2
Isoform 2 of 55 kDa erythrocyte membrane protein OS=Homo sapiens GN=MPP1 - [EM55_HUMAN]	2
Isoform 3 of Epsin-1 OS=Homo sapiens GN=EPN1 - [EPN1_HUMAN]	2
Isoform 2 of Exocyst complex component 1 OS=Homo sapiens GN=EXOC1 - [EXOC1_HUMAN]	2
Exocyst complex component 2 OS=Homo sapiens GN=EXOC2 PE=1 SV=1 - [EXOC2_HUMAN]	2
Protein FAM171B OS=Homo sapiens GN=FAM171B PE=2 SV=3 - [F171B_HUMAN]	2
Protein FAM98B OS=Homo sapiens GN=FAM98B PE=1 SV=1 - [FA98B_HUMAN]	2
Mitochondrial fission 1 protein OS=Homo sapiens GN=FIS1 PE=1 SV=2 - [FIS1_HUMAN]	2
Galactokinase OS=Homo sapiens GN=GALK1 PE=1 SV=1 - [GALK1_HUMAN]	2
Isoform 6 of Rap1 GTPase-GDP dissociation stimulator 1 OS=Homo sapiens GN=RAP1GDS1 - [GDS1_HUMAN]	2
Guanine nucleotide-binding protein subunit alpha-12 OS=Homo sapiens GN=GNA12 PE=1 SV=4 - [GNA12_HUMAN]	2
Neuronal membrane glycoprotein M6-b OS=Homo sapiens GN=GPM6B PE=1 SV=2 - [GPM6B_HUMAN]	2
Glutamate--cysteine ligase regulatory subunit OS=Homo sapiens GN=GCLM PE=1 SV=1 - [GSH0_HUMAN]	2
Glycogen synthase kinase-3 beta OS=Homo sapiens GN=GSK3B PE=1 SV=2 - [GSK3B_HUMAN]	2
Glutathione S-transferase Mu 5 OS=Homo sapiens GN=GSTM5 PE=1 SV=3 - [GSTM5_HUMAN]	2
Histone H2A type 2-B OS=Homo sapiens GN=HIST2H2AB PE=1 SV=3 - [H2A2B_HUMAN]	2
Core histone macro-H2A.2 OS=Homo sapiens GN=H2AFY2 PE=1 SV=3 - [H2AW_HUMAN]	2
Hydroxymethylglutaryl-CoA synthase, cytoplasmic OS=Homo sapiens GN=HMGCS1 PE=1 SV=2 - [HMCS1_HUMAN]	2
Heterogeneous nuclear ribonucleoprotein F OS=Homo sapiens GN=HNRNPF PE=1 SV=3 - [HNRPF_HUMAN]	2
Isoform 2 of Heterogeneous nuclear ribonucleoprotein L OS=Homo sapiens GN=HNRNPL - [HNRPL_HUMAN]	2
Hyaluronan and proteoglycan link protein 4 OS=Homo sapiens GN=HAPLN4 PE=2 SV=1 - [HPLN4_HUMAN]	2
Isoform 2 of Hydroxysteroid dehydrogenase-like protein 2 OS=Homo sapiens GN=HSDL2 - [HSDL2_HUMAN]	2
Intercellular adhesion molecule 5 OS=Homo sapiens GN=ICAM5 PE=1 SV=3 - [ICAM5_HUMAN]	2
Isoform Short of Eukaryotic translation initiation factor 4H OS=Homo sapiens GN=EIF4H - [IF4H_HUMAN]	2
Isoform 5 of Interleukin enhancer-binding factor 3 OS=Homo sapiens GN=ILF3 - [ILF3_HUMAN]	2
Importin subunit alpha-4 OS=Homo sapiens GN=KPNA3 PE=1 SV=2 - [IMA4_HUMAN]	2
Importin subunit alpha-5 OS=Homo sapiens GN=KPNA1 PE=1 SV=3 - [IMA5_HUMAN]	2
Isoform 4 of Intersectin-1 OS=Homo sapiens GN=ITSN1 - [ITSN1_HUMAN]	2
Uncharacterized protein KIAA1211-like OS=Homo sapiens GN=KIAA1211L PE=2 SV=3 - [K121L_HUMAN]	2
Keratin, type II cytoskeletal 74 OS=Homo sapiens GN=KRT74 PE=1 SV=2 - [K2C74_HUMAN]	2
Isoform 4 of Voltage-gated potassium channel subunit beta-2 OS=Homo sapiens GN=KCNAB2 - [KCAB2_HUMAN]	2
Calcium/calmodulin-dependent protein kinase type 1 OS=Homo sapiens GN=CAMK1 PE=1 SV=1 - [KCC1A_HUMAN]	2
BTB/POZ domain-containing protein KCTD16 OS=Homo sapiens GN=KCTD16 PE=2 SV=1 - [KCD16_HUMAN]	2
Kinesin light chain 2 OS=Homo sapiens GN=KLC2 PE=1 SV=1 - [KLC2_HUMAN]	2
Protein kinase C epsilon type OS=Homo sapiens GN=PRKCE PE=1 SV=1 - [KPCE_HUMAN]	2
Isoform 3 of Neural cell adhesion molecule L1 OS=Homo sapiens GN=L1CAM - [L1CAM_HUMAN]	2
Isoform 4 of LIM domain kinase 1 OS=Homo sapiens GN=LIMK1 - [LIMK1_HUMAN]	2
O-acetyl-ADP-ribose deacetylase MACROD1 OS=Homo sapiens GN=MACROD1 PE=1 SV=2 - [MACD1_HUMAN]	2
Isoform 2 of MAP/microtubule affinity-regulating kinase 4 OS=Homo sapiens GN=MARK4 - [MARK4_HUMAN]	2
Isoform 2 of Malignant T-cell-amplified sequence 1 OS=Homo sapiens GN=MCTS1 - [MCTS1_HUMAN]	2
Microfibril-associated glycoprotein 4 OS=Homo sapiens GN=MFAP4 PE=1 SV=2 - [MFAP4_HUMAN]	2
Myosin regulatory light chain 12A OS=Homo sapiens GN=MYL12A PE=1 SV=2 - [ML12A_HUMAN]	2
Isoform 6 of MMS19 nucleotide excision repair protein homolog OS=Homo sapiens GN=MMS19 - [MMS19_HUMAN]	2
Moesin OS=Homo sapiens GN=MSN PE=1 SV=3 - [MOES_HUMAN]	2
Myotubularin-related protein 5 OS=Homo sapiens GN=SBF1 PE=1 SV=3 - [MTMR5_HUMAN]	2
Cytochrome c oxidase subunit NDUFA4 OS=Homo sapiens GN=NDUFA4 PE=1 SV=1 - [NDUA4_HUMAN]	2
NADH dehydrogenase [ubiquinone] 1 alpha subcomplex assembly factor 3 OS=Homo sapiens GN=NDUFAF3 PE=1 SV=1 - [NDUF3_HUMAN]	2
NADH dehydrogenase [ubiquinone] iron-sulfur protein 5 OS=Homo sapiens GN=NDUFS5 PE=1 SV=3 - [NDUS5_HUMAN]	2
Isoform Cytoplasmic of Cysteine desulfurase, mitochondrial OS=Homo sapiens GN=NFS1 - [NFS1_HUMAN]	2
Isoform 7 of Non-specific lipid-transfer protein OS=Homo sapiens GN=SCP2 - [NLTP_HUMAN]	2
Nucleolar protein 56 OS=Homo sapiens GN=NOP56 PE=1 SV=4 - [NOP56_HUMAN]	2
Nuclear protein localization protein 4 homolog OS=Homo sapiens GN=NPLOC4 PE=1 SV=3 - [NPL4_HUMAN]	2
Isoform 5 of N-terminal kinase-like protein OS=Homo sapiens GN=SCYL1 - [NTKL_HUMAN]	2

Cancer-related nucleoside-triphosphatase OS=Homo sapiens GN=NTPCR PE=1 SV=1 - [NTPCR_HUMAN]	2
Diphosphoinositol polyphosphate phosphohydrolase 3-alpha OS=Homo sapiens GN=NUDT10 PE=1 SV=1 - [NUD10_HUMAN]	2
Isoform 3 of Opioid-binding protein/cell adhesion molecule OS=Homo sapiens GN=OPCML - [OPCM_HUMAN]	2
Paralemmin-1 OS=Homo sapiens GN=PALM PE=1 SV=2 - [PALM_HUMAN]	2
[Pyruvate dehydrogenase (acetyl-transferring)] kinase isozyme 1, mitochondrial OS=Homo sapiens GN=PDK1 PE=1 SV=1 - [PDK1_HUMAN]	2
Isoform 2 of 14 kDa phosphohistidine phosphatase OS=Homo sapiens GN=PHPT1 - [PHP14_HUMAN]	2
Isoform 3 of Phosphatidylinositol-binding clathrin assembly protein OS=Homo sapiens GN=PICALM - [PICAL_HUMAN]	2
Peptidyl-prolyl cis-trans isomerase NIMA-interacting 1 OS=Homo sapiens GN=PIN1 PE=1 SV=1 - [PIN1_HUMAN]	2
Isoform 2 of Pleckstrin homology domain-containing family A member 1 OS=Homo sapiens GN=PLEKHA1 - [PKHA1_HUMAN]	2
Isoform 3 of Inactive phospholipase C-like protein 2 OS=Homo sapiens GN=PLCL2 - [PLCL2_HUMAN]	2
Phospholipase D3 OS=Homo sapiens GN=PLD3 PE=1 SV=1 - [PLD3_HUMAN]	2
Plexin-D1 OS=Homo sapiens GN=PLXND1 PE=1 SV=3 - [PLXD1_HUMAN]	2
Isoform 2 of Palmitoyl-protein thioesterase 1 OS=Homo sapiens GN=PPT1 - [PPT1_HUMAN]	2
Peroxiredoxin-6 OS=Homo sapiens GN=PRDX6 PE=1 SV=3 - [PRDX6_HUMAN]	2
Isoform 2 of PH and SEC7 domain-containing protein 1 OS=Homo sapiens GN=PSD - [PSD1_HUMAN]	2
Prostaglandin-H2 D-isomerase OS=Homo sapiens GN=PTGDS PE=1 SV=1 - [PTGDS_HUMAN]	2
Tyrosine-protein phosphatase non-receptor type 23 OS=Homo sapiens GN=PTPN23 PE=1 SV=1 - [PTN23_HUMAN]	2
Cytochrome b-c1 complex subunit 1, mitochondrial OS=Homo sapiens GN=UQCRC1 PE=1 SV=3 - [QCR1_HUMAN]	2
Ras-related protein Rab-12 OS=Homo sapiens GN=RAB12 PE=1 SV=3 - [RAB12_HUMAN]	2
Ras-related protein Rab-18 OS=Homo sapiens GN=RAB18 PE=1 SV=1 - [RAB18_HUMAN]	2
Isoform 2 of Rab GTPase-binding effector protein 1 OS=Homo sapiens GN=RABEP1 - [RABE1_HUMAN]	2
Isoform 2B of GTPase KRas OS=Homo sapiens GN=KRAS - [RASK_HUMAN]	2
Ras-related protein Rab-39B OS=Homo sapiens GN=RAB39B PE=1 SV=1 - [RB39B_HUMAN]	2
RNA-binding protein 14 OS=Homo sapiens GN=RBM14 PE=1 SV=2 - [RBM14_HUMAN]	2
Isoform 4 of RNA-binding protein 4 OS=Homo sapiens GN=RBM4 - [RBM4_HUMAN]	2
Rho-related GTP-binding protein RhoB OS=Homo sapiens GN=RHOB PE=1 SV=1 - [RHOB_HUMAN]	2
Rho-related GTP-binding protein RhoG OS=Homo sapiens GN=RHOG PE=1 SV=1 - [RHOG_HUMAN]	2
60S ribosomal protein L13a OS=Homo sapiens GN=RPL13A PE=1 SV=2 - [RL13A_HUMAN]	2
60S ribosomal protein L27 OS=Homo sapiens GN=RPL27 PE=1 SV=2 - [RL27_HUMAN]	2
Isoform 3 of Rap1 GTPase-activating protein 2 OS=Homo sapiens GN=RAP1GAP2 - [RPGP2_HUMAN]	2
40S ribosomal protein S14 OS=Homo sapiens GN=RPS14 PE=1 SV=3 - [RS14_HUMAN]	2
Isoform 2 of 40S ribosomal protein S24 OS=Homo sapiens GN=RPS24 - [RS24_HUMAN]	2
40S ribosomal protein S25 OS=Homo sapiens GN=RPS25 PE=1 SV=1 - [RS25_HUMAN]	2
Isoform 4 of RUN and FYVE domain-containing protein 2 OS=Homo sapiens GN=RUFY2 - [RUFY2_HUMAN]	2
Protein S100-A13 OS=Homo sapiens GN=S100A13 PE=1 SV=1 - [S10AD_HUMAN]	2
Isoform 4 of Deoxynucleoside triphosphate triphosphohydrolase SAMHD1 OS=Homo sapiens GN=SAMHD1 - [SAMH1_HUMAN]	2
Protein SCAI OS=Homo sapiens GN=SCAI PE=1 SV=2 - [SCAI_HUMAN]	2
Isoform 2 of Sec1 family domain-containing protein 1 OS=Homo sapiens GN=SCFD1 - [SCFD1_HUMAN]	2
Isoform 2 of Calcium-binding mitochondrial carrier protein SCaMC-1 OS=Homo sapiens GN=SLC25A24 - [SCMC1_HUMAN]	2
Sodium channel subunit beta-2 OS=Homo sapiens GN=SCN2B PE=1 SV=1 - [SCN2B_HUMAN]	2
Isoform Short of Splicing factor, proline- and glutamine-rich OS=Homo sapiens GN=SFPQ - [SFPQ_HUMAN]	2
Endophilin-B1 OS=Homo sapiens GN=SH3GLB1 PE=1 SV=1 - [SHLB1_HUMAN]	2
Beta-2-syntrophin OS=Homo sapiens GN=SNTB2 PE=1 SV=1 - [SNTB2_HUMAN]	2
Isoform 4 of Spectrin beta chain, non-erythrocytic 4 OS=Homo sapiens GN=SPTBN4 - [SPTN4_HUMAN]	2
Isoform 3 of Src substrate cortactin OS=Homo sapiens GN=CTTN - [SRC8_HUMAN]	2
Serine racemase OS=Homo sapiens GN=SRR PE=1 SV=1 - [SRR_HUMAN]	2
Serine/threonine-protein kinase 38-like OS=Homo sapiens GN=STK38L PE=1 SV=3 - [ST38L_HUMAN]	2
Stathmin OS=Homo sapiens GN=STMN1 PE=1 SV=3 - [STMN1_HUMAN]	2
Isoform 3 of Syntaxin-1A OS=Homo sapiens GN=STX1A - [STX1A_HUMAN]	2
Syntaxin-binding protein 6 OS=Homo sapiens GN=STXBP6 PE=1 SV=2 - [STXB6_HUMAN]	2
Lysine--tRNA ligase OS=Homo sapiens GN=KARS PE=1 SV=3 - [SYK_HUMAN]	2
Isoform 2-4 of Alpha-synuclein OS=Homo sapiens GN=SNCA - [SYUA_HUMAN]	2
Isoform 2 of TSC22 domain family protein 1 OS=Homo sapiens GN=TSC22D1 - [T22D1_HUMAN]	2

Isoform 2 of TGF-beta-activated kinase 1 and MAP3K7-binding protein 1 OS=Homo sapiens GN=TAB1 - [TAB1_HUMAN]	2
Transgelin-3 OS=Homo sapiens GN=TAGLN3 PE=1 SV=2 - [TAGL3_HUMAN]	2
Isoform 2 of TBC1 domain family member 15 OS=Homo sapiens GN=TBC1D15 - [TBC15_HUMAN]	2
Tubulin-specific chaperone D OS=Homo sapiens GN=TBCD PE=1 SV=2 - [TBCD_HUMAN]	2
Serine/threonine-protein kinase TBK1 OS=Homo sapiens GN=TBK1 PE=1 SV=1 - [TBK1_HUMAN]	2
Mitochondrial import receptor subunit TOM70 OS=Homo sapiens GN=TOMM70A PE=1 SV=1 - [TOM70_HUMAN]	2
Tripartite motif-containing protein 2 OS=Homo sapiens GN=TRIM2 PE=1 SV=1 - [TRIM2_HUMAN]	2
Tetratricopeptide repeat protein 9B OS=Homo sapiens GN=TTC9B PE=2 SV=1 - [TTC9B_HUMAN]	2
Tricarboxylate transport protein, mitochondrial OS=Homo sapiens GN=SLC25A1 PE=1 SV=2 - [TXTP_HUMAN]	2
NEDD8-conjugating enzyme Ubc12 OS=Homo sapiens GN=UBE2M PE=1 SV=1 - [UBC12_HUMAN]	2
Synaptic vesicle membrane protein VAT-1 homolog OS=Homo sapiens GN=VAT1 PE=1 SV=2 - [VAT1_HUMAN]	2
Deubiquitinating protein VCIP135 OS=Homo sapiens GN=VCPIP1 PE=1 SV=2 - [VCIP1_HUMAN]	2
Vacuolar protein sorting-associated protein 35 OS=Homo sapiens GN=VPS35 PE=1 SV=2 - [VPS35_HUMAN]	2
Vacuolar protein sorting-associated protein 4B OS=Homo sapiens GN=VPS4B PE=1 SV=2 - [VPS4B_HUMAN]	2
Exportin-T OS=Homo sapiens GN=XPOT PE=1 SV=2 - [XPOT_HUMAN]	2
X-ray repair cross-complementing protein 5 OS=Homo sapiens GN=XRCC5 PE=1 SV=3 - [XRCC5_HUMAN]	2
GTP-binding protein Rheb OS=Homo sapiens GN=RHEB PE=1 SV=1 - [C9J931_HUMAN]	2
Synaptogyrin-1 OS=Homo sapiens GN=SYNGR1 PE=1 SV=1 - [B5MCD7_HUMAN]	2
Isoform 2 of Ephrin type-A receptor 4 OS=Homo sapiens GN=EPHA4 - [EPHA4_HUMAN]	2
Isoform 3 of AP-3 complex subunit beta-2 OS=Homo sapiens GN=AP3B2 - [AP3B2_HUMAN]	2
Neuroplastin (Fragment) OS=Homo sapiens GN=DKFZp566H1924 PE=1 SV=2 - [Q9UFM8_HUMAN]	2
Isoform 2 of Gamma-soluble NSF attachment protein OS=Homo sapiens GN=NAPG - [SNAG_HUMAN]	2
Muscleblind-like 2 (Drosophila), isoform CRA_b OS=Homo sapiens GN=MBLL PE=1 SV=1 - [O95205_HUMAN]	2
Isoform 2 of Dihydropteridine reductase OS=Homo sapiens GN=QDPR - [DHPR_HUMAN]	2
Isoform 2 of Small nuclear ribonucleoprotein Sm D3 OS=Homo sapiens GN=SNRPD3 - [SMD3_HUMAN]	2
Ribosomal protein S6 kinase alpha-2 OS=Homo sapiens GN=RPS6KA2 PE=1 SV=1 - [B7Z3B5_HUMAN]	2
Superoxide dismutase [Mn], mitochondrial OS=Homo sapiens GN=SOD2 PE=4 SV=1 - [A0A0C4DG56_HUMAN]	2
MAGUK p55 subfamily member 6 OS=Homo sapiens GN=MPP6 PE=1 SV=1 - [B8ZZG1_HUMAN]	2
Isoform 2 of Stomatin-like protein 2, mitochondrial OS=Homo sapiens GN=STOML2 - [STML2_HUMAN]	2
HCG1995540, isoform CRA_b OS=Homo sapiens GN=RAB4B PE=1 SV=1 - [Q6PIK3_HUMAN]	2
NADH dehydrogenase [ubiquinone] 1 alpha subcomplex subunit 13 OS=Homo sapiens GN=NDUFA13 PE=2 SV=1 - [B4DEZ3_HUMAN]	2
Isoform 2 of Prostaglandin E synthase 3 OS=Homo sapiens GN=PTGES3 - [TEBP_HUMAN]	2
Heat shock protein 75 kDa, mitochondrial OS=Homo sapiens GN=TRAP1 PE=1 SV=1 - [I3L0K7_HUMAN]	2
Methylcrotonoyl-CoA carboxylase subunit alpha, mitochondrial OS=Homo sapiens GN=MCCC1 PE=1 SV=1 - [G5E9X5_HUMAN]	2
Profilin 1, isoform CRA_b OS=Homo sapiens GN=PFN1 PE=1 SV=1 - [K7EJ44_HUMAN]	2
Isoleucine--tRNA ligase, cytoplasmic OS=Homo sapiens GN=IARS PE=1 SV=1 - [J3KR24_HUMAN]	2
Metaxin-2 OS=Homo sapiens GN=MTX2 PE=1 SV=1 - [C9JNK6_HUMAN]	2
Dipeptidyl aminopeptidase-like protein 6 OS=Homo sapiens GN=DPP6 PE=1 SV=1 - [E9PDL2_HUMAN]	2
LanC-like protein 1 (Fragment) OS=Homo sapiens GN=LANCL1 PE=1 SV=1 - [E9PHS0_HUMAN]	2
GTPase-activating protein and VPS9 domain-containing protein 1 OS=Homo sapiens GN=GAPVD1 PE=1 SV=1 - [F8W9S7_HUMAN]	2
60S ribosomal protein L8 (Fragment) OS=Homo sapiens GN=RPL8 PE=1 SV=1 - [E9PKZ0_HUMAN]	2
Ras GTPase-activating protein-binding protein 2 (Fragment) OS=Homo sapiens GN=G3BP2 PE=1 SV=1 - [D6RAC7_HUMAN]	2
NSFL1 cofactor p47 OS=Homo sapiens GN=NSFL1C PE=1 SV=1 - [F2Z2K0_HUMAN]	2
Isoform 3 of Calponin-3 OS=Homo sapiens GN=CNN3 - [CNN3_HUMAN]	2
Neutral alpha-glucosidase AB OS=Homo sapiens GN=GANAB PE=1 SV=1 - [F5H6X6_HUMAN]	2
Coenzyme Q-binding protein COQ10 homolog B, mitochondrial OS=Homo sapiens GN=COQ10B PE=1 SV=1 - [B8ZZX2_HUMAN]	2
Succinate-semialdehyde dehydrogenase, mitochondrial OS=Homo sapiens GN=ALDH5A1 PE=1 SV=1 - [C9J8Q5_HUMAN]	2
Acid ceramidase OS=Homo sapiens GN=ASAH1 PE=1 SV=1 - [E7EMM4_HUMAN]	2
NADH dehydrogenase [ubiquinone] iron-sulfur protein 7, mitochondrial OS=Homo sapiens GN=NDUFS7 PE=1 SV=1 - [F5H5N1_HUMAN]	2
Succinate dehydrogenase [ubiquinone] flavoprotein subunit, mitochondrial OS=Homo sapiens GN=SDHA PE=1 SV=1 - [D6RFM5_HUMAN]	2
Peroxisomal multifunctional enzyme type 2 OS=Homo sapiens GN=HSD17B4 PE=1 SV=3 - [E7ER27_HUMAN]	2
Ras-related protein Rab-7a (Fragment) OS=Homo sapiens GN=RAB7A PE=1 SV=1 - [C9J592_HUMAN]	2
mRNA export factor OS=Homo sapiens GN=RAE1 PE=1 SV=1 - [E9PPG9_HUMAN]	2

ATP synthase mitochondrial F1 complex assembly factor 1 OS=Homo sapiens GN=ATPAF1 PE=1 SV=2 - [A8MRA7_HUMAN]	2
Rho GTPase-activating protein 21 (Fragment) OS=Homo sapiens GN=ARHGAP21 PE=1 SV=1 - [E7ESW5_HUMAN]	2
Eukaryotic translation initiation factor 3 subunit E OS=Homo sapiens GN=EIF3E PE=1 SV=1 - [E5RGA2_HUMAN]	2
Exocyst complex component 3 OS=Homo sapiens GN=EXOC3 PE=1 SV=1 - [D6RB59_HUMAN]	2
Glutamate receptor 3 OS=Homo sapiens GN=GRIA3 PE=1 SV=1 - [A0A087WYJ6_HUMAN]	2
AP2-associated protein kinase 1 OS=Homo sapiens GN=AAK1 PE=1 SV=1 - [E9PG46_HUMAN]	2
Biglycan (Fragment) OS=Homo sapiens GN=BGN PE=1 SV=1 - [C9JKG1_HUMAN]	2
Ran-specific GTPase-activating protein (Fragment) OS=Homo sapiens GN=RANBP1 PE=1 SV=1 - [C9JJ34_HUMAN]	2
Sickle tail protein homolog (Fragment) OS=Homo sapiens GN=KIAA1217 PE=1 SV=1 - [Q5T5P0_HUMAN]	2
Isoform 3 of Ral GTPase-activating protein subunit beta OS=Homo sapiens GN=RALGAPB - [RLGPB_HUMAN]	2
Armadillo repeat-containing protein 10 (Fragment) OS=Homo sapiens GN=ARMC10 PE=1 SV=1 - [H7C2M7_HUMAN]	2
Transcription factor A, mitochondrial (Fragment) OS=Homo sapiens GN=TFAM PE=1 SV=1 - [H7BYN3_HUMAN]	2
Prostamide/prostaglandin F synthase (Fragment) OS=Homo sapiens GN=FAM213B PE=1 SV=1 - [J3QKK8_HUMAN]	2
5'-AMP-activated protein kinase subunit gamma-1 (Fragment) OS=Homo sapiens GN=PRKAG1 PE=1 SV=1 - [H0YHF8_HUMAN]	2
Signal transducer and activator of transcription OS=Homo sapiens GN=STAT3 PE=1 SV=1 - [G8JLH9_HUMAN]	2
Signal recognition particle 14 kDa protein OS=Homo sapiens GN=SRP14 PE=1 SV=1 - [H0YLA2_HUMAN]	2
Translin (Fragment) OS=Homo sapiens GN=TSN PE=1 SV=1 - [H7C1D4_HUMAN]	2
Latrophilin-3 (Fragment) OS=Homo sapiens GN=LPHN3 PE=1 SV=1 - [H0Y9K5_HUMAN]	2
Activator of 90 kDa heat shock protein ATPase homolog 1 (Fragment) OS=Homo sapiens GN=AHSA1 PE=1 SV=1 - [H0YJG7_HUMAN]	2
60S ribosomal protein L31 (Fragment) OS=Homo sapiens GN=RPL31 PE=1 SV=1 - [H7C2W9_HUMAN]	2
Histone H3 (Fragment) OS=Homo sapiens GN=H3F3B PE=1 SV=1 - [K7EK07_HUMAN]	2
SURP and G-patch domain-containing protein 2 OS=Homo sapiens GN=SUGP2 PE=1 SV=1 - [M0R3F6_HUMAN]	2
COP9 signalosome complex subunit 3 OS=Homo sapiens GN=COPS3 PE=1 SV=2 - [H7C3P9_HUMAN]	2
Vigilin (Fragment) OS=Homo sapiens GN=HDLBP PE=1 SV=1 - [H0Y394_HUMAN]	2
60S ribosomal protein L13 (Fragment) OS=Homo sapiens GN=RPL13 PE=1 SV=1 - [J3QSB4_HUMAN]	2
Sideroflexin-5 (Fragment) OS=Homo sapiens GN=SFXN5 PE=1 SV=1 - [H7C1S9_HUMAN]	2
rRNA 2'-O-methyltransferase fibrillarin (Fragment) OS=Homo sapiens GN=FBL PE=1 SV=1 - [M0R0P1_HUMAN]	2
cAMP-dependent protein kinase type I-alpha regulatory subunit (Fragment) OS=Homo sapiens GN=PRKAR1A PE=1 SV=1 - [K7EPB2_HUMAN]	2
Myelin-associated glycoprotein (Fragment) OS=Homo sapiens GN=MAG PE=1 SV=1 - [M0QZU4_HUMAN]	2
V-type proton ATPase subunit d 1 (Fragment) OS=Homo sapiens GN=ATP6V0D1 PE=1 SV=1 - [J3QL14_HUMAN]	2
Rho GDP-dissociation inhibitor 1 OS=Homo sapiens GN=ARHGDIA PE=1 SV=1 - [J3KRE2_HUMAN]	2
Microtubule-associated protein (Fragment) OS=Homo sapiens GN=MAP4 PE=1 SV=1 - [H0Y2V1_HUMAN]	2
40S ribosomal protein S11 OS=Homo sapiens GN=RPS11 PE=1 SV=1 - [M0QZC5_HUMAN]	2
Inosine-5'-monophosphate dehydrogenase 2 (Fragment) OS=Homo sapiens GN=IMPDH2 PE=1 SV=1 - [H0Y4R1_HUMAN]	2
Uncharacterized protein (Fragment) OS=Homo sapiens PE=3 SV=1 - [M0QYV0_HUMAN]	2
Fibronectin type III and SPRY domain-containing protein 1 OS=Homo sapiens GN=FSD1 PE=1 SV=1 - [M0R366_HUMAN]	2
Leucine zipper transcription factor-like protein 1 (Fragment) OS=Homo sapiens GN=LZTFL1 PE=1 SV=1 - [H7C488_HUMAN]	2
Cleavage and polyadenylation-specificity factor subunit 5 (Fragment) OS=Homo sapiens GN=NUDT21 PE=1 SV=4 - [H3BND3_HUMAN]	2
Isoform 3 of Programmed cell death 6-interacting protein OS=Homo sapiens GN=PDCD6IP - [PDC6I_HUMAN]	2
Isoform 6 of cGMP-dependent 3',5'-cyclic phosphodiesterase OS=Homo sapiens GN=PDE2A - [PDE2A_HUMAN]	2
Isoform 2 of STE20/SPS1-related proline-alanine-rich protein kinase OS=Homo sapiens GN=STK39 - [STK39_HUMAN]	2
Isoform 3 of TBC1 domain family member 17 OS=Homo sapiens GN=TBC1D17 - [TBC17_HUMAN]	2
HCG1985580, isoform CRA_c OS=Homo sapiens GN=PDCD6 PE=1 SV=1 - [A0A024QZ42_HUMAN]	2
PSAP protein OS=Homo sapiens GN=PSAP PE=1 SV=1 - [Q5BJH1_HUMAN]	2
ARP2 actin-related protein 2 homolog (Yeast), isoform CRA_d OS=Homo sapiens GN=ACTR2 PE=1 SV=2 - [F5H6T1_HUMAN]	2
Keratin, type II cuticular Hb1 OS=Homo sapiens GN=KRT81 PE=1 SV=1 - [A0A087X106_HUMAN]	2
Serine/threonine-protein phosphatase 2A 56 kDa regulatory subunit delta isoform (Fragment) OS=Homo sapiens GN=PPP2R5D PE=1 SV=4 - [H7C5Q9_HUMAN]	2
60S ribosomal protein L17 (Fragment) OS=Homo sapiens GN=RPL17 PE=1 SV=2 - [A0A087WY81_HUMAN]	2
Amino acid transporter OS=Homo sapiens GN=SLC1A3 PE=1 SV=1 - [A0A087X0U3_HUMAN]	2
Neuronal growth regulator 1 OS=Homo sapiens GN=NEGR1 PE=1 SV=1 - [F6X2W2_HUMAN]	2
Neuronal cell adhesion molecule OS=Homo sapiens GN=NRCAM PE=1 SV=1 - [A0A087X2B3_HUMAN]	2
Thioredoxin reductase 2, mitochondrial OS=Homo sapiens GN=TXNRD2 PE=1 SV=1 - [A0A096LNN4_HUMAN]	2
Translation initiation factor eIF-2B subunit delta OS=Homo sapiens GN=EIF2B4 PE=1 SV=1 - [A0A087WTA5_HUMAN]	2

ATPase, H+ transporting, lysosomal accessory protein 1, isoform CRA_c OS=Homo sapiens GN=ATP6AP1 PE=4 SV=1 - [A0A0C4DGX8_HUMAN]	2
Lysophospholipid acyltransferase LPCAT4 OS=Homo sapiens GN=LPCAT4 PE=4 SV=1 - [A0A0C4DGT4_HUMAN]	2
Cullin-associated NEDD8-dissociated protein 1 (Fragment) OS=Homo sapiens GN=CAND1 PE=4 SV=1 - [A0A0C4DGH5_HUMAN]	2

Supplementary Table II. Human peptides identified in P2 and P7 fractions in AD2.

Description AD2 P2	Peptides
Spectrin alpha chain, non-erythrocytic 1 OS=Homo sapiens GN=SPTAN1 PE=1 SV=3 - [SPTN1_HUMAN]	87
Isoform 7 of Plectin OS=Homo sapiens GN=PLEC - [PLEC_HUMAN]	52
Spectrin beta chain, non-erythrocytic 1 OS=Homo sapiens GN=SPTBN1 PE=1 SV=2 - [SPTB2_HUMAN]	52
Glial fibrillary acidic protein OS=Homo sapiens GN=GFAP PE=1 SV=1 - [GFAP_HUMAN]	38
Isoform 3 of Dynamin-1 OS=Homo sapiens GN=DNM1 - [DYN1_HUMAN]	37
Isoform 4 of Dynamin-1 OS=Homo sapiens GN=DNM1 - [DYN1_HUMAN]	37
Vesicle-fusing ATPase OS=Homo sapiens GN=NSF PE=1 SV=1 - [I3L0N3_HUMAN]	35
2',3'-cyclic-nucleotide 3'-phosphodiesterase OS=Homo sapiens GN=CNP PE=1 SV=2 - [CN37_HUMAN]	33
Hexokinase-1 OS=Homo sapiens GN=HK1 PE=1 SV=3 - [HXK1_HUMAN]	32
Neurofilament medium polypeptide OS=Homo sapiens GN=NEFM PE=1 SV=1 - [E7ESP9_HUMAN]	32
Isoform 2 of Annexin A6 OS=Homo sapiens GN=ANXA6 - [ANXA6_HUMAN]	30
Isoform 2 of Contactin-1 OS=Homo sapiens GN=CNTN1 - [CNTN1_HUMAN]	30
Heat shock protein HSP 90-alpha OS=Homo sapiens GN=HSP90AA1 PE=1 SV=5 - [HS90A_HUMAN]	29
Neurofilament light polypeptide OS=Homo sapiens GN=NEFL PE=1 SV=3 - [NFL_HUMAN]	29
Vimentin OS=Homo sapiens GN=VIM PE=1 SV=1 - [B0YJC4_HUMAN]	29
Ankyrin-2 OS=Homo sapiens GN=ANK2 PE=1 SV=4 - [ANK2_HUMAN]	27
Alpha-internexin OS=Homo sapiens GN=INA PE=1 SV=2 - [AINX_HUMAN]	26
Isoform 2 of Clathrin heavy chain 1 OS=Homo sapiens GN=CLTC - [CLH1_HUMAN]	26
Heat shock protein HSP 90-beta OS=Homo sapiens GN=HSP90AB1 PE=1 SV=4 - [HS90B_HUMAN]	25
Isoform C of Prelamin-A/C OS=Homo sapiens GN=LMNA - [LMNA_HUMAN]	25
ATP synthase subunit alpha, mitochondrial OS=Homo sapiens GN=ATP5A1 PE=1 SV=1 - [ATPA_HUMAN]	24
Heat shock 70 kDa protein 12A OS=Homo sapiens GN=HSPA12A PE=1 SV=2 - [HS12A_HUMAN]	24
Isoform 2 of Syntaxin-binding protein 1 OS=Homo sapiens GN=STXBP1 - [STXB1_HUMAN]	24
Glutamate dehydrogenase 1, mitochondrial OS=Homo sapiens GN=GLUD1 PE=1 SV=2 - [DHE3_HUMAN]	23
Calcium-binding mitochondrial carrier protein Aralar1 OS=Homo sapiens GN=SLC25A12 PE=1 SV=2 - [CMC1_HUMAN]	22
Heat shock cognate 71 kDa protein OS=Homo sapiens GN=HSPA8 PE=1 SV=1 - [HSP7C_HUMAN]	22
Isoform 2 of Tubulin alpha-1A chain OS=Homo sapiens GN=TUBA1A - [TBA1A_HUMAN]	22
Tubulin beta-2A chain OS=Homo sapiens GN=TUBB2A PE=1 SV=1 - [TBB2A_HUMAN]	22
Tubulin beta-2B chain OS=Homo sapiens GN=TUBB2B PE=1 SV=1 - [TBB2B_HUMAN]	22
Tubulin beta-4B chain OS=Homo sapiens GN=TUBB4B PE=1 SV=1 - [TBB4B_HUMAN]	22
Tubulin beta chain OS=Homo sapiens GN=TUBB PE=1 SV=2 - [TBB5_HUMAN]	22
Aconitate hydratase, mitochondrial OS=Homo sapiens GN=ACO2 PE=1 SV=2 - [ACON_HUMAN]	21
60 kDa heat shock protein, mitochondrial OS=Homo sapiens GN=HSPD1 PE=1 SV=2 - [CH60_HUMAN]	21
Isoform 2 of Dynamin-3 OS=Homo sapiens GN=DNM3 - [DYN3_HUMAN]	21
Tubulin beta-4A chain OS=Homo sapiens GN=TUBB4A PE=1 SV=2 - [TBB4A_HUMAN]	21
Isoform M1 of Pyruvate kinase PKM OS=Homo sapiens GN=PKM - [KPYM_HUMAN]	20
Lamin-B2 OS=Homo sapiens GN=LMNB2 PE=1 SV=3 - [LMNB2_HUMAN]	20
Tubulin beta-3 chain OS=Homo sapiens GN=TUBB3 PE=1 SV=2 - [TBB3_HUMAN]	20
Isoform 4 of Band 4.1-like protein 3 OS=Homo sapiens GN=EPB41L3 - [E41L3_HUMAN]	20
Aspartate aminotransferase, mitochondrial OS=Homo sapiens GN=GOT2 PE=1 SV=3 - [AATM_HUMAN]	19
Amine oxidase [flavin-containing] B OS=Homo sapiens GN=MAOB PE=1 SV=3 - [AOFB_HUMAN]	19
Isoform 3 of Sodium/potassium-transporting ATPase subunit alpha-1 OS=Homo sapiens GN=ATP1A1 - [AT1A1_HUMAN]	19
Cytoplasmic dynein 1 heavy chain 1 OS=Homo sapiens GN=DYNC1H1 PE=1 SV=5 - [DYHC1_HUMAN]	19

Stress-70 protein, mitochondrial OS=Homo sapiens GN=HSPA9 PE=1 SV=2 - [GRP75_HUMAN]	19
78 kDa glucose-regulated protein OS=Homo sapiens GN=HSPA5 PE=1 SV=2 - [GRP78_HUMAN]	19
Heat shock-related 70 kDa protein 2 OS=Homo sapiens GN=HSPA2 PE=1 SV=1 - [HSP72_HUMAN]	19
ATP-dependent 6-phosphofructokinase, platelet type OS=Homo sapiens GN=PFKP PE=1 SV=2 - [PFKAP_HUMAN]	19
Isoform 2 of Tubulin alpha-4A chain OS=Homo sapiens GN=TUBA4A - [TBA4A_HUMAN]	19
Guanine nucleotide-binding protein G(o) subunit alpha OS=Homo sapiens GN=GNAO1 PE=1 SV=4 - [GNAO_HUMAN]	18
Isoform 2 of V-type proton ATPase catalytic subunit A OS=Homo sapiens GN=ATP6V1A - [VATA_HUMAN]	18
Sodium/potassium-transporting ATPase subunit alpha-2 OS=Homo sapiens GN=ATP1A2 PE=1 SV=1 - [B1AKY9_HUMAN]	18
Actin, cytoplasmic 1 OS=Homo sapiens GN=ACTB PE=1 SV=1 - [ACTB_HUMAN]	17
Annexin A5 OS=Homo sapiens GN=ANXA5 PE=1 SV=2 - [ANXA5_HUMAN]	17
Isoform 3 of Dynamin-2 OS=Homo sapiens GN=DNM2 - [DYN2_HUMAN]	17
Alpha-enolase OS=Homo sapiens GN=ENO1 PE=1 SV=2 - [ENOA_HUMAN]	17
Isocitrate dehydrogenase [NADP], mitochondrial OS=Homo sapiens GN=IDH2 PE=1 SV=2 - [IDHP_HUMAN]	17
NADH-ubiquinone oxidoreductase 75 kDa subunit, mitochondrial OS=Homo sapiens GN=NDUFS1 PE=1 SV=3 - [NDUS1_HUMAN]	17
Dynamin-like 120 kDa protein, mitochondrial OS=Homo sapiens GN=OPA1 PE=1 SV=3 - [OPA1_HUMAN]	17
Protein disulfide-isomerase A3 OS=Homo sapiens GN=PDIA3 PE=1 SV=4 - [PDIA3_HUMAN]	17
Synaptotagmin I, isoform CRA_b OS=Homo sapiens GN=SYT1 PE=1 SV=1 - [J3KQA0_HUMAN]	17
AP-2 complex subunit beta OS=Homo sapiens GN=AP2B1 PE=1 SV=1 - [A0A087X253_HUMAN]	17
Sodium/potassium-transporting ATPase subunit alpha-3 OS=Homo sapiens GN=ATP1A3 PE=1 SV=1 - [M0R116_HUMAN]	17
ATP synthase subunit beta, mitochondrial OS=Homo sapiens GN=ATP5B PE=1 SV=3 - [ATPB_HUMAN]	16
Trifunctional enzyme subunit alpha, mitochondrial OS=Homo sapiens GN=HADHA PE=1 SV=2 - [ECHA_HUMAN]	16
Heat shock 70 kDa protein 1A/1B OS=Homo sapiens GN=HSPA1A PE=1 SV=5 - [HSP71_HUMAN]	16
Microtubule-associated protein 1B OS=Homo sapiens GN=MAP1B PE=1 SV=2 - [MAP1B_HUMAN]	16
Isoform Short of Ubiquitin carboxyl-terminal hydrolase 5 OS=Homo sapiens GN=USP5 - [UBP5_HUMAN]	16
MICOS complex subunit MIC60 OS=Homo sapiens GN=IMMT PE=1 SV=2 - [B9A067_HUMAN]	16
Isoform 2 of Ubiquitin-like modifier-activating enzyme 1 OS=Homo sapiens GN=UBA1 - [UBA1_HUMAN]	16
Serum albumin OS=Homo sapiens GN=ALB PE=1 SV=2 - [ALBU_HUMAN]	15
Fructose-bisphosphate aldolase A OS=Homo sapiens GN=ALDOA PE=1 SV=2 - [ALDOA_HUMAN]	15
Isoform 2 of Dihydropyrimidinase-related protein 2 OS=Homo sapiens GN=DPYSL2 - [DPYL2_HUMAN]	15
Calcium/calmodulin-dependent protein kinase type II subunit alpha OS=Homo sapiens GN=CAMK2A PE=1 SV=2 - [KCC2A_HUMAN]	15
Isoform 10 of Neurofascin OS=Homo sapiens GN=NFASC - [NFASC_HUMAN]	15
Isoform 2 of Tubulin alpha-8 chain OS=Homo sapiens GN=TUBA8 - [TBA8_HUMAN]	15
Tubulin beta-6 chain OS=Homo sapiens GN=TUBB6 PE=1 SV=1 - [TBB6_HUMAN]	15
14-3-3 protein epsilon OS=Homo sapiens GN=YWHAE PE=1 SV=1 - [1433E_HUMAN]	14
Actin, gamma-enteric smooth muscle OS=Homo sapiens GN=ACTG2 PE=1 SV=1 - [ACTH_HUMAN]	14
Isoform 2 of Alpha-actinin-1 OS=Homo sapiens GN=ACTN1 - [ACTN1_HUMAN]	14
Alpha-actinin-4 OS=Homo sapiens GN=ACTN4 PE=1 SV=2 - [ACTN4_HUMAN]	14
EH domain-containing protein 3 OS=Homo sapiens GN=EHD3 PE=1 SV=2 - [EHD3_HUMAN]	14
Glucose-6-phosphate isomerase OS=Homo sapiens GN=GPI PE=1 SV=4 - [G6PI_HUMAN]	14
Glycerol-3-phosphate dehydrogenase, mitochondrial OS=Homo sapiens GN=GPD2 PE=1 SV=3 - [GPDM_HUMAN]	14
Neural cell adhesion molecule 1 OS=Homo sapiens GN=NCAM1 PE=1 SV=3 - [NCAM1_HUMAN]	14
Glycogen phosphorylase, brain form OS=Homo sapiens GN=PYGB PE=1 SV=5 - [PYGB_HUMAN]	14
T-complex protein 1 subunit theta OS=Homo sapiens GN=CCT8 PE=1 SV=4 - [TCPQ_HUMAN]	14
Isoform 2 of Triosephosphate isomerase OS=Homo sapiens GN=TPI1 - [TPIS_HUMAN]	14
Band 4.1-like protein 1 OS=Homo sapiens GN=EPB41L1 PE=4 SV=1 - [A0A0C4DH22_HUMAN]	14
Serine/threonine-protein phosphatase 2A 65 kDa regulatory subunit A alpha isoform OS=Homo sapiens GN=PPP2R1A PE=1 SV=4 - [2AAA_HUMAN]	13
Elongation factor Tu, mitochondrial OS=Homo sapiens GN=TUFM PE=1 SV=2 - [EFTU_HUMAN]	13
Rab GDP dissociation inhibitor alpha OS=Homo sapiens GN=GDI1 PE=1 SV=2 - [GDIA_HUMAN]	13
Isoform 3 of Microtubule-associated protein 2 OS=Homo sapiens GN=MAP2 - [MTAP2_HUMAN]	13
NADH dehydrogenase [ubiquinone] 1 alpha subcomplex subunit 9, mitochondrial OS=Homo sapiens GN=NDUFA9 PE=1 SV=2 - [NDUA9_HUMAN]	13
Isoform 2 of Neurofilament heavy polypeptide OS=Homo sapiens GN=NEFH - [NFH_HUMAN]	13
Cytochrome b-c1 complex subunit 2, mitochondrial OS=Homo sapiens GN=UQCRC2 PE=1 SV=3 - [QCR2_HUMAN]	13
Tyrosine-protein phosphatase non-receptor type substrate 1 OS=Homo sapiens GN=SIRPA PE=1 SV=2 - [SHPS1_HUMAN]	13

Alanine--tRNA ligase, cytoplasmic OS=Homo sapiens GN=AARS PE=1 SV=2 - [SYAC_HUMAN]	13
Isoform IB of Synapsin-1 OS=Homo sapiens GN=SYN1 - [SYN1_HUMAN]	13
T-complex protein 1 subunit beta OS=Homo sapiens GN=CCT2 PE=1 SV=4 - [TCPB_HUMAN]	13
V-type proton ATPase subunit B, brain isoform OS=Homo sapiens GN=ATP6V1B2 PE=1 SV=3 - [VATB2_HUMAN]	13
Visinin-like protein 1 OS=Homo sapiens GN=VSNL1 PE=1 SV=2 - [VISL1_HUMAN]	13
14-3-3 protein eta OS=Homo sapiens GN=YWHAH PE=1 SV=4 - [1433F_HUMAN]	12
14-3-3 protein gamma OS=Homo sapiens GN=YWHAG PE=1 SV=2 - [1433G_HUMAN]	12
Isoform B of AP-2 complex subunit alpha-1 OS=Homo sapiens GN=AP2A1 - [AP2A1_HUMAN]	12
ATP synthase F(0) complex subunit B1, mitochondrial OS=Homo sapiens GN=ATP5F1 PE=1 SV=2 - [AT5F1_HUMAN]	12
Calnexin OS=Homo sapiens GN=CANX PE=1 SV=2 - [CALX_HUMAN]	12
Gamma-enolase OS=Homo sapiens GN=ENO2 PE=1 SV=3 - [ENOG_HUMAN]	12
Endoplasmin OS=Homo sapiens GN=HSP90B1 PE=1 SV=1 - [ENPL_HUMAN]	12
Fibrinogen beta chain OS=Homo sapiens GN=FGB PE=1 SV=2 - [FIBB_HUMAN]	12
Glutaminase kidney isoform, mitochondrial OS=Homo sapiens GN=GLS PE=1 SV=1 - [GLSK_HUMAN]	12
Isoform 3 of Calcium/calmodulin-dependent protein kinase type II subunit beta OS=Homo sapiens GN=CAMK2B - [KCC2B_HUMAN]	12
Malate dehydrogenase, mitochondrial OS=Homo sapiens GN=MDH2 PE=1 SV=3 - [MDHM_HUMAN]	12
Isoform 2 of Pyruvate dehydrogenase E1 component subunit beta, mitochondrial OS=Homo sapiens GN=PDHB - [ODPB_HUMAN]	12
Protein-arginine deiminase type-2 OS=Homo sapiens GN=PADI2 PE=2 SV=2 - [PADI2_HUMAN]	12
Isoform A2 of Heterogeneous nuclear ribonucleoproteins A2/B1 OS=Homo sapiens GN=HNRNPA2B1 - [ROA2_HUMAN]	12
Beta-soluble NSF attachment protein OS=Homo sapiens GN=NAPB PE=1 SV=2 - [SNAB_HUMAN]	12
Transitional endoplasmic reticulum ATPase OS=Homo sapiens GN=VCP PE=1 SV=4 - [TERA_HUMAN]	12
V-type proton ATPase subunit C 1 OS=Homo sapiens GN=ATP6V1C1 PE=1 SV=4 - [VATC1_HUMAN]	12
Myelin-oligodendrocyte glycoprotein OS=Homo sapiens GN=MOG PE=1 SV=1 - [C9JTE0_HUMAN]	12
Fructose-bisphosphate aldolase OS=Homo sapiens GN=ALDOC PE=1 SV=1 - [A8MVZ9_HUMAN]	12
14-3-3 protein zeta/delta OS=Homo sapiens GN=YWHAZ PE=1 SV=1 - [1433Z_HUMAN]	11
ADP/ATP translocase 2 OS=Homo sapiens GN=SLC25A5 PE=1 SV=7 - [ADT2_HUMAN]	11
ADP/ATP translocase 3 OS=Homo sapiens GN=SLC25A6 PE=1 SV=4 - [ADT3_HUMAN]	11
Isoform 2 of Alpha-aminoadipic semialdehyde dehydrogenase OS=Homo sapiens GN=ALDH7A1 - [AL7A1_HUMAN]	11
AP-2 complex subunit alpha-2 OS=Homo sapiens GN=AP2A2 PE=1 SV=2 - [AP2A2_HUMAN]	11
ATP synthase subunit gamma, mitochondrial OS=Homo sapiens GN=ATP5C1 PE=1 SV=1 - [ATPG_HUMAN]	11
Isoform 2 of Gelsolin OS=Homo sapiens GN=GSN - [GELS_HUMAN]	11
Guanine nucleotide-binding protein G(i) subunit alpha-2 OS=Homo sapiens GN=GNAI2 PE=1 SV=3 - [GNAI2_HUMAN]	11
Hemoglobin subunit beta OS=Homo sapiens GN=HBB PE=1 SV=2 - [HBB_HUMAN]	11
Heat shock protein beta-1 OS=Homo sapiens GN=HSPB1 PE=1 SV=2 - [HSPB1_HUMAN]	11
Creatine kinase B-type OS=Homo sapiens GN=CKB PE=1 SV=1 - [KCRB_HUMAN]	11
L-lactate dehydrogenase A chain OS=Homo sapiens GN=LDHA PE=1 SV=2 - [LDHA_HUMAN]	11
Microtubule-associated protein 1A OS=Homo sapiens GN=MAP1A PE=1 SV=6 - [MAP1A_HUMAN]	11
Protein disulfide-isomerase OS=Homo sapiens GN=P4HB PE=1 SV=3 - [PDIA1_HUMAN]	11
Phosphoglycerate kinase 1 OS=Homo sapiens GN=PGK1 PE=1 SV=3 - [PGK1_HUMAN]	11
Protein RUFY3 OS=Homo sapiens GN=RUFY3 PE=1 SV=1 - [RUFY3_HUMAN]	11
Septin-7 OS=Homo sapiens GN=SEPT7 PE=1 SV=2 - [SEPT7_HUMAN]	11
Isoform 2 of Syntaxin-1B OS=Homo sapiens GN=STX1B - [STX1B_HUMAN]	11
Tenascin-R OS=Homo sapiens GN=TNR PE=1 SV=3 - [TENR_HUMAN]	11
V-type proton ATPase subunit E 1 OS=Homo sapiens GN=ATP6V1E1 PE=1 SV=1 - [VATE1_HUMAN]	11
Citrate synthase, mitochondrial OS=Homo sapiens GN=CS PE=1 SV=1 - [B4DJV2_HUMAN]	11
Alpha-crystallin B chain (Fragment) OS=Homo sapiens GN=CRYAB PE=1 SV=1 - [E9PR44_HUMAN]	11
Prohibitin-2 OS=Homo sapiens GN=PHB2 PE=1 SV=1 - [F5GY37_HUMAN]	11
Myelin basic protein OS=Homo sapiens GN=MBP PE=1 SV=1 - [J3QL64_HUMAN]	11
Isoform Short of 14-3-3 protein beta/alpha OS=Homo sapiens GN=YWHAB - [1433B_HUMAN]	10
14-3-3 protein theta OS=Homo sapiens GN=YWHAQ PE=1 SV=1 - [1433T_HUMAN]	10
Isoform 2 of 4F2 cell-surface antigen heavy chain OS=Homo sapiens GN=SLC3A2 - [4F2_HUMAN]	10
Aspartate aminotransferase, cytoplasmic OS=Homo sapiens GN=GOT1 PE=1 SV=3 - [AATC_HUMAN]	10
ADP/ATP translocase 1 OS=Homo sapiens GN=SLC25A4 PE=1 SV=4 - [ADT1_HUMAN]	10

Isoform 2 of Aldehyde dehydrogenase, mitochondrial OS=Homo sapiens GN=ALDH2 - [ALDH2_HUMAN]	10
Isoform 2 of Annexin A7 OS=Homo sapiens GN=ANXA7 - [ANXA7_HUMAN]	10
Isoform C of AP-1 complex subunit beta-1 OS=Homo sapiens GN=AP1B1 - [AP1B1_HUMAN]	10
Isoform 2 of Atlastin-1 OS=Homo sapiens GN=ATL1 - [ATLA1_HUMAN]	10
Rap1 GTPase-GDP dissociation stimulator 1 OS=Homo sapiens GN=RAP1GDS1 PE=1 SV=3 - [GDS1_HUMAN]	10
Guanine nucleotide-binding protein G(i) subunit alpha-1 OS=Homo sapiens GN=GNAI1 PE=1 SV=2 - [GNAI1_HUMAN]	10
Isoform 3 of Guanine nucleotide-binding protein G(s) subunit alpha isoforms short OS=Homo sapiens GN=GNAS - [GNAS2_HUMAN]	10
Keratin, type I cytoskeletal 10 OS=Homo sapiens GN=KRT10 PE=1 SV=6 - [K1C10_HUMAN]	10
Keratin, type II cytoskeletal 1 OS=Homo sapiens GN=KRT1 PE=1 SV=6 - [K2C1_HUMAN]	10
L-lactate dehydrogenase B chain OS=Homo sapiens GN=LDHB PE=1 SV=2 - [LDHB_HUMAN]	10
Isoform 6 of Myelin basic protein OS=Homo sapiens GN=MBP - [MBP_HUMAN]	10
Isoform 2 of 2-oxoglutarate dehydrogenase, mitochondrial OS=Homo sapiens GN=OGDH - [ODO1_HUMAN]	10
Cytochrome b-c1 complex subunit 1, mitochondrial OS=Homo sapiens GN=UQCRC1 PE=1 SV=3 - [QCR1_HUMAN]	10
Synaptosomal-associated protein 25 OS=Homo sapiens GN=SNAP25 PE=1 SV=1 - [SNP25_HUMAN]	10
Syntaxin-1A OS=Homo sapiens GN=STX1A PE=1 SV=1 - [STX1A_HUMAN]	10
Isoform 2 of Succinyl-CoA ligase [ADP-forming] subunit beta, mitochondrial OS=Homo sapiens GN=SUCLA2 - [SUCB1_HUMAN]	10
T-complex protein 1 subunit epsilon OS=Homo sapiens GN=CCT5 PE=1 SV=1 - [TCPE_HUMAN]	10
Voltage-dependent anion-selective channel protein 1 OS=Homo sapiens GN=VDAC1 PE=1 SV=2 - [VDAC1_HUMAN]	10
Isoform 2 of Protein kinase C gamma type OS=Homo sapiens GN=PRKCG - [KPCG_HUMAN]	10
Isoform 2 of Trifunctional enzyme subunit beta, mitochondrial OS=Homo sapiens GN=HADHB - [ECHB_HUMAN]	10
Isoform 2 of Methylmalonate-semialdehyde dehydrogenase [acylating], mitochondrial OS=Homo sapiens GN=ALDH6A1 - [MMSA_HUMAN]	10
Neutral alpha-glucosidase AB OS=Homo sapiens GN=GANAB PE=1 SV=1 - [F5H6X6_HUMAN]	10
Peroxiredoxin-1 (Fragment) OS=Homo sapiens GN=PRDX1 PE=1 SV=1 - [A0A0A0MSI0_HUMAN]	10
Annexin A2 OS=Homo sapiens GN=ANXA2 PE=1 SV=2 - [ANXA2_HUMAN]	9
Isoform E of Plasma membrane calcium-transporting ATPase 1 OS=Homo sapiens GN=ATP2B1 - [AT2B1_HUMAN]	9
Isoform ZK of Plasma membrane calcium-transporting ATPase 4 OS=Homo sapiens GN=ATP2B4 - [AT2B4_HUMAN]	9
ATP synthase subunit d, mitochondrial OS=Homo sapiens GN=ATP5H PE=1 SV=3 - [ATP5H_HUMAN]	9
Brain acid soluble protein 1 OS=Homo sapiens GN=BASP1 PE=1 SV=2 - [BASP1_HUMAN]	9
C-1-tetrahydrofolate synthase, cytoplasmic OS=Homo sapiens GN=MTHFD1 PE=1 SV=3 - [C1TC_HUMAN]	9
Carbonyl reductase [NADPH] 1 OS=Homo sapiens GN=CBR1 PE=1 SV=3 - [CBR1_HUMAN]	9
Cytosolic non-specific dipeptidase OS=Homo sapiens GN=CNDP2 PE=1 SV=2 - [CNDP2_HUMAN]	9
Contactin-associated protein 1 OS=Homo sapiens GN=CNTNAP1 PE=1 SV=1 - [CNTP1_HUMAN]	9
Dynactin subunit 2 OS=Homo sapiens GN=DCTN2 PE=1 SV=4 - [DCTN2_HUMAN]	9
Elongation factor 1-alpha 1 OS=Homo sapiens GN=EEF1A1 PE=1 SV=1 - [EF1A1_HUMAN]	9
Elongation factor 1-alpha 2 OS=Homo sapiens GN=EEF1A2 PE=1 SV=1 - [EF1A2_HUMAN]	9
Rab GDP dissociation inhibitor beta OS=Homo sapiens GN=GDI2 PE=1 SV=2 - [GDIB_HUMAN]	9
Guanine nucleotide-binding protein subunit alpha-13 OS=Homo sapiens GN=GNA13 PE=1 SV=2 - [GNA13_HUMAN]	9
Histone H4 OS=Homo sapiens GN=HIST1H4A PE=1 SV=2 - [H4_HUMAN]	9
Isoform 2 of Heterogeneous nuclear ribonucleoprotein M OS=Homo sapiens GN=HNRNPM - [HNRPM_HUMAN]	9
Importin subunit beta-1 OS=Homo sapiens GN=KPNB1 PE=1 SV=2 - [IMB1_HUMAN]	9
Isoform 3 of Adenylate kinase isoenzyme 5 OS=Homo sapiens GN=AK5 - [KAD5_HUMAN]	9
Isoform Delta 12 of Calcium/calmodulin-dependent protein kinase type II subunit delta OS=Homo sapiens GN=CAMK2D - [KCC2D_HUMAN]	9
Malate dehydrogenase, cytoplasmic OS=Homo sapiens GN=MDH1 PE=1 SV=4 - [MDHC_HUMAN]	9
Isoform 2 of NADH-cytochrome b5 reductase 3 OS=Homo sapiens GN=CYB5R3 - [NB5R3_HUMAN]	9
NADH dehydrogenase [ubiquinone] iron-sulfur protein 3, mitochondrial OS=Homo sapiens GN=NDUFS3 PE=1 SV=1 - [NDUS3_HUMAN]	9
Isoform 2 of NADH dehydrogenase [ubiquinone] flavoprotein 1, mitochondrial OS=Homo sapiens GN=NDUFV1 - [NDUV1_HUMAN]	9
Brevican core protein OS=Homo sapiens GN=BCAN PE=1 SV=2 - [PGCB_HUMAN]	9
Prohibitin OS=Homo sapiens GN=PHB PE=1 SV=1 - [PHB_HUMAN]	9
Rho GTPase-activating protein 1 OS=Homo sapiens GN=ARHGAP1 PE=1 SV=1 - [RHG01_HUMAN]	9
Signal-regulatory protein beta-1 isoform 3 OS=Homo sapiens GN=SIRPB1 PE=1 SV=1 - [SIRBL_HUMAN]	9
Isoform 3 of T-complex protein 1 subunit eta OS=Homo sapiens GN=CCT7 - [TCPH_HUMAN]	9
T-complex protein 1 subunit zeta OS=Homo sapiens GN=CCT6A PE=1 SV=3 - [TCPZ_HUMAN]	9
Exportin-1 OS=Homo sapiens GN=XPO1 PE=1 SV=1 - [XPO1_HUMAN]	9

Septin 11, isoform CRA_b OS=Homo sapiens GN=SEPT11 PE=1 SV=1 - [D6RGI3_HUMAN]	9
Dipeptidyl aminopeptidase-like protein 6 OS=Homo sapiens GN=DPP6 PE=1 SV=1 - [E9PDL2_HUMAN]	9
Dihydrolipoyl dehydrogenase, mitochondrial OS=Homo sapiens GN=DLD PE=1 SV=1 - [E9PEX6_HUMAN]	9
Lon protease homolog, mitochondrial OS=Homo sapiens GN=LONP1 PE=1 SV=1 - [K7EJE8_HUMAN]	9
V-type proton ATPase subunit d 1 OS=Homo sapiens GN=ATP6V0D1 PE=1 SV=1 - [R4GN72_HUMAN]	9
Mitochondrial 2-oxoglutarate/malate carrier protein (Fragment) OS=Homo sapiens GN=SLC25A11 PE=1 SV=1 - [I3L1P8_HUMAN]	9
Sideroflexin-3 OS=Homo sapiens GN=SFXN3 PE=1 SV=1 - [A0A0A0MS41_HUMAN]	9
Synapsin-2 OS=Homo sapiens GN=SYN2 PE=1 SV=1 - [A0A087X2E3_HUMAN]	9
NAD-dependent protein deacetylase sirtuin-2 OS=Homo sapiens GN=SIRT2 PE=1 SV=1 - [A0A0A0MRF5_HUMAN]	9
Septin-8 OS=Homo sapiens GN=SEPT8 PE=1 SV=1 - [A0A087X142_HUMAN]	9
Isoform 2 of Alpha-adducin OS=Homo sapiens GN=ADD1 - [ADDA_HUMAN]	8
ATP synthase subunit O, mitochondrial OS=Homo sapiens GN=ATP5O PE=1 SV=1 - [ATPO_HUMAN]	8
Cytochrome c oxidase subunit 4 isoform 1, mitochondrial OS=Homo sapiens GN=COX4I1 PE=1 SV=1 - [COX41_HUMAN]	8
Copine-5 OS=Homo sapiens GN=CPNE5 PE=1 SV=2 - [CPNE5_HUMAN]	8
Isoform V3 of Versican core protein OS=Homo sapiens GN=VCAN - [CSPG2_HUMAN]	8
Succinate dehydrogenase [ubiquinone] flavoprotein subunit, mitochondrial OS=Homo sapiens GN=SDHA PE=1 SV=2 - [SDHA_HUMAN]	8
Succinate dehydrogenase [ubiquinone] iron-sulfur subunit, mitochondrial OS=Homo sapiens GN=SDHB PE=1 SV=3 - [SDHB_HUMAN]	8
ATP-dependent RNA helicase A OS=Homo sapiens GN=DHX9 PE=1 SV=4 - [DHX9_HUMAN]	8
Isoform 3 of Disks large homolog 4 OS=Homo sapiens GN=DLG4 - [DLG4_HUMAN]	8
Elongation factor 2 OS=Homo sapiens GN=EEF2 PE=1 SV=4 - [EF2_HUMAN]	8
Isoform 2 of Fibrinogen alpha chain OS=Homo sapiens GN=FGA - [FIBA_HUMAN]	8
4-aminobutyrate aminotransferase, mitochondrial OS=Homo sapiens GN=ABAT PE=1 SV=3 - [GABT_HUMAN]	8
Ganglioside-induced differentiation-associated protein 1 OS=Homo sapiens GN=GDAP1 PE=1 SV=3 - [GDAP1_HUMAN]	8
Guanine nucleotide-binding protein G(z) subunit alpha OS=Homo sapiens GN=GNAZ PE=2 SV=3 - [GNAZ_HUMAN]	8
Isoform 3 of Heterogeneous nuclear ribonucleoprotein D0 OS=Homo sapiens GN=HNRNPD - [HNRPD_HUMAN]	8
Isocitrate dehydrogenase [NAD] subunit alpha, mitochondrial OS=Homo sapiens GN=IDH3A PE=1 SV=1 - [IDH3A_HUMAN]	8
Eukaryotic initiation factor 4A-II OS=Homo sapiens GN=EIF4A2 PE=1 SV=2 - [IF4A2_HUMAN]	8
Immunoglobulin superfamily member 8 OS=Homo sapiens GN=IGSF8 PE=1 SV=1 - [IGSF8_HUMAN]	8
Isoform 5 of Interleukin enhancer-binding factor 3 OS=Homo sapiens GN=ILF3 - [ILF3_HUMAN]	8
Keratin, type I cytoskeletal 9 OS=Homo sapiens GN=KRT9 PE=1 SV=3 - [K1C9_HUMAN]	8
ATP-dependent 6-phosphofructokinase, liver type OS=Homo sapiens GN=PFKL PE=1 SV=6 - [PFKAL_HUMAN]	8
Methyl-CpG-binding protein 2 OS=Homo sapiens GN=MECP2 PE=1 SV=1 - [MECP2_HUMAN]	8
Isoform 2 of Dual specificity mitogen-activated protein kinase kinase 1 OS=Homo sapiens GN=MAP2K1 - [MP2K1_HUMAN]	8
Isoform 3 of Nucleoside diphosphate kinase B OS=Homo sapiens GN=NME2 - [NDKB_HUMAN]	8
2-oxoglutarate dehydrogenase-like, mitochondrial OS=Homo sapiens GN=OGDHL PE=1 SV=3 - [OGDHL_HUMAN]	8
Phosphatidylethanolamine-binding protein 1 OS=Homo sapiens GN=PEBP1 PE=1 SV=3 - [PEBP1_HUMAN]	8
Phosphatidylinositol 5-phosphate 4-kinase type-2 beta OS=Homo sapiens GN=PIP4K2B PE=1 SV=1 - [PI42B_HUMAN]	8
Isoform 2 of Serine/threonine-protein phosphatase 2B catalytic subunit alpha isoform OS=Homo sapiens GN=PPP3CA - [PP2BA_HUMAN]	8
Peptidyl-prolyl cis-trans isomerase A OS=Homo sapiens GN=PPIA PE=1 SV=2 - [PPIA_HUMAN]	8
Isoform Cytoplasmic+peroxisomal of Peroxiredoxin-5, mitochondrial OS=Homo sapiens GN=PRDX5 - [PRDX5_HUMAN]	8
Isoform 2 of Bifunctional purine biosynthesis protein PURH OS=Homo sapiens GN=ATIC - [PUR9_HUMAN]	8
Pyruvate carboxylase, mitochondrial OS=Homo sapiens GN=PC PE=1 SV=2 - [PYC_HUMAN]	8
Ras-related protein Rab-2A OS=Homo sapiens GN=RAB2A PE=1 SV=1 - [RAB2A_HUMAN]	8
Ras-related protein Rab-3A OS=Homo sapiens GN=RAB3A PE=1 SV=1 - [RAB3A_HUMAN]	8
Ras-related C3 botulinum toxin substrate 1 OS=Homo sapiens GN=RAC1 PE=1 SV=1 - [RAC1_HUMAN]	8
Isoform 2 of Neuronal-specific septin-3 OS=Homo sapiens GN=SEPT3 - [SEPT3_HUMAN]	8
D-3-phosphoglycerate dehydrogenase OS=Homo sapiens GN=PHGDH PE=1 SV=4 - [SERA_HUMAN]	8
Sideroflexin-1 OS=Homo sapiens GN=SFXN1 PE=1 SV=4 - [SFXN1_HUMAN]	8
Succinate-semialdehyde dehydrogenase, mitochondrial OS=Homo sapiens GN=ALDH5A1 PE=1 SV=2 - [SSDH_HUMAN]	8
Acetyl-CoA acetyltransferase, mitochondrial OS=Homo sapiens GN=ACAT1 PE=1 SV=1 - [THIL_HUMAN]	8
Transketolase OS=Homo sapiens GN=TKT PE=1 SV=3 - [TKT_HUMAN]	8
Mitochondrial import receptor subunit TOM70 OS=Homo sapiens GN=TOMM70A PE=1 SV=1 - [TOM70_HUMAN]	8
Vacuolar protein sorting-associated protein 35 OS=Homo sapiens GN=VPS35 PE=1 SV=2 - [VPS35_HUMAN]	8

T-complex protein 1 subunit gamma OS=Homo sapiens GN=CCT3 PE=1 SV=1 - [B4DUR8_HUMAN]	8
Calcium/calmodulin-dependent protein kinase (CaM kinase) II gamma, isoform CRA_n OS=Homo sapiens GN=CAMK2G PE=1 SV=1 - [Q5SWX3_HUMAN]	8
Isoform 2 of Flotillin-1 OS=Homo sapiens GN=FLOT1 - [FLOT1_HUMAN]	8
ATPase, H+ transporting, lysosomal 50/57kDa, V1 subunit H, isoform CRA_c OS=Homo sapiens GN=ATP6V1H PE=1 SV=1 - [G3V126_HUMAN]	8
Puromycin-sensitive aminopeptidase OS=Homo sapiens GN=NPEPPS PE=1 SV=1 - [E9PLK3_HUMAN]	8
Heterogeneous nuclear ribonucleoprotein A1 (Fragment) OS=Homo sapiens GN=HNRNPA1 PE=1 SV=1 - [F8VZ49_HUMAN]	8
cGMP-dependent 3',5'-cyclic phosphodiesterase OS=Homo sapiens GN=PDE2A PE=1 SV=2 - [E9PEF1_HUMAN]	8
Isoform 3 of Receptor-type tyrosine-protein phosphatase zeta OS=Homo sapiens GN=PTPRZ1 - [PTPRZ_HUMAN]	8
Endophilin-B2 OS=Homo sapiens GN=SH3GLB2 PE=1 SV=1 - [B7ZC39_HUMAN]	8
Ubiquitin thioesterase OTUB1 OS=Homo sapiens GN=OTUB1 PE=1 SV=1 - [F5GYN4_HUMAN]	8
Alpha-soluble NSF attachment protein OS=Homo sapiens GN=NAPA PE=1 SV=1 - [M0R0Y2_HUMAN]	8
Alpha-actinin-2 OS=Homo sapiens GN=ACTN2 PE=1 SV=1 - [ACTN2_HUMAN]	7
Annexin A1 OS=Homo sapiens GN=ANXA1 PE=1 SV=2 - [ANXA1_HUMAN]	7
Apolipoprotein E OS=Homo sapiens GN=APOE PE=1 SV=1 - [APOE_HUMAN]	7
Isoform ZA of Plasma membrane calcium-transporting ATPase 2 OS=Homo sapiens GN=ATP2B2 - [AT2B2_HUMAN]	7
Isoform IIC1 of Myc box-dependent-interacting protein 1 OS=Homo sapiens GN=BIN1 - [BIN1_HUMAN]	7
Isoform BIN1-10-13 of Myc box-dependent-interacting protein 1 OS=Homo sapiens GN=BIN1 - [BIN1_HUMAN]	7
Cyclin-dependent-like kinase 5 OS=Homo sapiens GN=CDK5 PE=1 SV=3 - [CDK5_HUMAN]	7
Isoform 4 of Clusterin OS=Homo sapiens GN=CLU - [CLUS_HUMAN]	7
Copine-6 OS=Homo sapiens GN=CPNE6 PE=1 SV=3 - [CPNE6_HUMAN]	7
Cytochrome c1, heme protein, mitochondrial OS=Homo sapiens GN=CYC1 PE=1 SV=3 - [CY1_HUMAN]	7
Cytoplasmic FMR1-interacting protein 1 OS=Homo sapiens GN=CYFIP1 PE=1 SV=1 - [CYFP1_HUMAN]	7
Dihydropyrimidinase-related protein 3 OS=Homo sapiens GN=DPYSL3 PE=1 SV=1 - [DPYL3_HUMAN]	7
Isoform Gamma-A of Fibrinogen gamma chain OS=Homo sapiens GN=FGG - [FIBG_HUMAN]	7
Ferritin heavy chain OS=Homo sapiens GN=FTH1 PE=1 SV=2 - [FRIH_HUMAN]	7
Guanine nucleotide-binding protein G(q) subunit alpha OS=Homo sapiens GN=GNAQ PE=1 SV=4 - [GNAQ_HUMAN]	7
Histone H1.2 OS=Homo sapiens GN=HIST1H1C PE=1 SV=2 - [H12_HUMAN]	7
Heterogeneous nuclear ribonucleoprotein R OS=Homo sapiens GN=HNRNPR PE=1 SV=1 - [HNRPR_HUMAN]	7
Hyaluronan and proteoglycan link protein 2 OS=Homo sapiens GN=HAPLN2 PE=1 SV=1 - [HPLN2_HUMAN]	7
Isocitrate dehydrogenase [NAD] subunit beta, mitochondrial OS=Homo sapiens GN=IDH3B PE=1 SV=2 - [IDH3B_HUMAN]	7
Eukaryotic initiation factor 4A-I OS=Homo sapiens GN=EIF4A1 PE=1 SV=1 - [IF4A1_HUMAN]	7
UMP-CMP kinase OS=Homo sapiens GN=CMPK1 PE=1 SV=3 - [KCY_HUMAN]	7
Isoform 2 of Kinesin-like protein KIF2A OS=Homo sapiens GN=KIF2A - [KIF2A_HUMAN]	7
Limbic system-associated membrane protein OS=Homo sapiens GN=LSAMP PE=1 SV=2 - [LSAMP_HUMAN]	7
Isoform 2 of Neurochondrin OS=Homo sapiens GN=NCDN - [NCDN_HUMAN]	7
Nucleoside diphosphate kinase A OS=Homo sapiens GN=NME1 PE=1 SV=1 - [NDKA_HUMAN]	7
Dihydrolipoyllysine-residue succinyltransferase component of 2-oxoglutarate dehydrogenase complex, mitochondrial OS=Homo sapiens GN=DLST PE=1 SV=4 - [ODO2_HUMAN]	7
Isoform 3 of Pyruvate dehydrogenase E1 component subunit alpha, somatic form, mitochondrial OS=Homo sapiens GN=PDHA1 - [ODPA_HUMAN]	7
Pyridoxal kinase OS=Homo sapiens GN=PDXK PE=1 SV=1 - [PDXK_HUMAN]	7
Phosphoglycerate mutase 1 OS=Homo sapiens GN=PGAM1 PE=1 SV=2 - [PGAM1_HUMAN]	7
POTE ankyrin domain family member E OS=Homo sapiens GN=POTEE PE=1 SV=3 - [POTEE_HUMAN]	7
Peroxiredoxin-2 OS=Homo sapiens GN=PRDX2 PE=1 SV=5 - [PRDX2_HUMAN]	7
Ras-related protein Rab-5C OS=Homo sapiens GN=RAB5C PE=1 SV=2 - [RAB5C_HUMAN]	7
Succinyl-CoA:3-ketoacid coenzyme A transferase 1, mitochondrial OS=Homo sapiens GN=OXCT1 PE=1 SV=1 - [SCOT1_HUMAN]	7
Gamma-soluble NSF attachment protein OS=Homo sapiens GN=NAPG PE=1 SV=1 - [SNAG_HUMAN]	7
Isoform 2 of Spectrin beta chain, non-erythrocytic 2 OS=Homo sapiens GN=SPTBN2 - [SPTN2_HUMAN]	7
Tubulin beta-1 chain OS=Homo sapiens GN=TUBB1 PE=1 SV=1 - [TBB1_HUMAN]	7
T-complex protein 1 subunit alpha OS=Homo sapiens GN=TCP1 PE=1 SV=1 - [TCPA_HUMAN]	7
T-complex protein 1 subunit delta OS=Homo sapiens GN=CCT4 PE=1 SV=4 - [TCPD_HUMAN]	7
WD repeat-containing protein 1 OS=Homo sapiens GN=WDR1 PE=1 SV=4 - [WDR1_HUMAN]	7
Isoform 2 of Phosphatidylinositol 5-phosphate 4-kinase type-2 alpha OS=Homo sapiens GN=PIP4K2A - [PI42A_HUMAN]	7
Isoform 2 of Annexin A11 OS=Homo sapiens GN=ANXA11 - [ANX11_HUMAN]	7

Heterogeneous nuclear ribonucleoproteins C1/C2 OS=Homo sapiens GN=HNRNPC PE=1 SV=1 - [G3V576_HUMAN]	7
Heterogeneous nuclear ribonucleoprotein K (Fragment) OS=Homo sapiens GN=HNRNPK PE=1 SV=1 - [Q5T6W2_HUMAN]	7
Protein kinase C and casein kinase substrate in neurons protein 1 OS=Homo sapiens GN=PACSIN1 PE=1 SV=1 - [F6U236_HUMAN]	7
Cytoplasmic FMR1-interacting protein 2 OS=Homo sapiens GN=CYFIP2 PE=1 SV=1 - [E7EVJ5_HUMAN]	7
Dihydropyrimidinase-related protein 1 OS=Homo sapiens GN=CRMP1 PE=1 SV=1 - [E9PD68_HUMAN]	7
Ubiquitin carboxyl-terminal hydrolase OS=Homo sapiens GN=UCHL1 PE=1 SV=1 - [D6RE83_HUMAN]	7
Septin-5 OS=Homo sapiens GN=SEPT5 PE=1 SV=1 - [E7EX32_HUMAN]	7
Isoform 2 of Heat shock protein 75 kDa, mitochondrial OS=Homo sapiens GN=TRAP1 - [TRAP1_HUMAN]	7
Isoform 2 of cAMP-dependent protein kinase type I-alpha regulatory subunit OS=Homo sapiens GN=PRKAR1A - [KAP0_HUMAN]	7
Acetyltransferase component of pyruvate dehydrogenase complex (Fragment) OS=Homo sapiens GN=DLAT PE=1 SV=1 - [H0YDD4_HUMAN]	7
Interleukin enhancer-binding factor 2 OS=Homo sapiens GN=ILF2 PE=1 SV=1 - [B4DY09_HUMAN]	7
Band 4.1-like protein 2 OS=Homo sapiens GN=EPB41L2 PE=1 SV=1 - [I6L9B1_HUMAN]	7
Voltage-dependent anion-selective channel protein 2 (Fragment) OS=Homo sapiens GN=VDAC2 PE=1 SV=1 - [A0A0A0MR02_HUMAN]	7
Calcium/calmodulin-dependent protein kinase type 1D OS=Homo sapiens GN=CAMK1D PE=1 SV=1 - [A0A087WUR6_HUMAN]	7
NADH dehydrogenase [ubiquinone] 1 alpha subcomplex subunit 10, mitochondrial OS=Homo sapiens GN=NDUFA10 PE=1 SV=1 - [A0A087WXC5_HUMAN]	7
Isoform 2 of Very long-chain specific acyl-CoA dehydrogenase, mitochondrial OS=Homo sapiens GN=ACADVL - [ACADV_HUMAN]	6
Isoform 2 of ATP-citrate synthase OS=Homo sapiens GN=ACLY - [ACLY_HUMAN]	6
AFG3-like protein 2 OS=Homo sapiens GN=AFG3L2 PE=1 SV=2 - [AFG32_HUMAN]	6
Alcohol dehydrogenase [NADP(+)] OS=Homo sapiens GN=AKR1A1 PE=1 SV=3 - [AK1A1_HUMAN]	6
Isoform 3 of Delta-1-pyrroline-5-carboxylate dehydrogenase, mitochondrial OS=Homo sapiens GN=ALDH4A1 - [AL4A1_HUMAN]	6
Isoform 2 of AP-2 complex subunit mu OS=Homo sapiens GN=AP2M1 - [AP2M1_HUMAN]	6
Isoform 2 of Sodium/potassium-transporting ATPase subunit beta-1 OS=Homo sapiens GN=ATP1B1 - [AT1B1_HUMAN]	6
Sodium/potassium-transporting ATPase subunit beta-2 OS=Homo sapiens GN=ATP1B2 PE=1 SV=3 - [AT1B2_HUMAN]	6
Isoform ZG of Plasma membrane calcium-transporting ATPase 3 OS=Homo sapiens GN=ATP2B3 - [AT2B3_HUMAN]	6
Isoform 4 of Voltage-dependent calcium channel subunit alpha-2/delta-1 OS=Homo sapiens GN=CACNA2D1 - [CA2D1_HUMAN]	6
Cell adhesion molecule 3 OS=Homo sapiens GN=CADM3 PE=1 SV=1 - [CADM3_HUMAN]	6
Cathepsin D OS=Homo sapiens GN=CTSD PE=1 SV=1 - [CATD_HUMAN]	6
MICOS complex subunit MIC19 OS=Homo sapiens GN=CHCHD3 PE=1 SV=1 - [MIC19_HUMAN]	6
Cytochrome c oxidase subunit 2 OS=Homo sapiens GN=MT-CO2 PE=1 SV=1 - [COX2_HUMAN]	6
Isoform 1 of Serine/threonine-protein kinase DCLK1 OS=Homo sapiens GN=DCLK1 - [DCLK1_HUMAN]	6
N(G),N(G)-dimethylarginine dimethylaminohydrolase 1 OS=Homo sapiens GN=DDAH1 PE=1 SV=3 - [DDAH1_HUMAN]	6
Isoform 2 of ATP-dependent RNA helicase DDX3X OS=Homo sapiens GN=DDX3X - [DDX3X_HUMAN]	6
Desmin OS=Homo sapiens GN=DES PE=1 SV=3 - [DESM_HUMAN]	6
Very-long-chain 3-oxoacyl-CoA reductase OS=Homo sapiens GN=HSD17B12 PE=1 SV=2 - [DHB12_HUMAN]	6
Dihydropteridine reductase OS=Homo sapiens GN=QDPR PE=1 SV=2 - [DHPR_HUMAN]	6
Ectonucleotide pyrophosphatase/phosphodiesterase family member 6 OS=Homo sapiens GN=ENPP6 PE=1 SV=2 - [ENPP6_HUMAN]	6
Ferritin light chain OS=Homo sapiens GN=FTL PE=1 SV=2 - [FRIL_HUMAN]	6
Fascin OS=Homo sapiens GN=FSCN1 PE=1 SV=3 - [FSCN1_HUMAN]	6
Isoform 2 of Glyceraldehyde-3-phosphate dehydrogenase OS=Homo sapiens GN=GAPDH - [G3P_HUMAN]	6
Guanine nucleotide-binding protein G(I)/G(S)/G(T) subunit beta-2 OS=Homo sapiens GN=GNB2 PE=1 SV=3 - [GBB2_HUMAN]	6
Guanine nucleotide-binding protein G(k) subunit alpha OS=Homo sapiens GN=GNAI3 PE=1 SV=3 - [GNAI3_HUMAN]	6
Isoform 3 of Neuronal membrane glycoprotein M6-a OS=Homo sapiens GN=GPM6A - [GPM6A_HUMAN]	6
Isoform 1 of Core histone macro-H2A.1 OS=Homo sapiens GN=H2AFY - [H2AY_HUMAN]	6
Isoform 2 of Heterogeneous nuclear ribonucleoprotein L OS=Homo sapiens GN=HNRNPL - [HNRPL_HUMAN]	6
Isoform Short of Heterogeneous nuclear ribonucleoprotein U OS=Homo sapiens GN=HNRNPU - [HNRPU_HUMAN]	6
Hippocalcin-like protein 1 OS=Homo sapiens GN=HPCAL1 PE=1 SV=3 - [HPCL1_HUMAN]	6
Hypoxanthine-guanine phosphoribosyltransferase OS=Homo sapiens GN=HPRT1 PE=1 SV=2 - [HPRT_HUMAN]	6
Kelch repeat and BTB domain-containing protein 11 OS=Homo sapiens GN=KBTBD11 PE=1 SV=1 - [KBTBB_HUMAN]	6
Protein kinase C alpha type OS=Homo sapiens GN=PRKCA PE=1 SV=4 - [KPCA_HUMAN]	6
Isoform 3 of Neural cell adhesion molecule L1 OS=Homo sapiens GN=L1CAM - [L1CAM_HUMAN]	6
Galectin-1 OS=Homo sapiens GN=LGALS1 PE=1 SV=2 - [LEG1_HUMAN]	6
Protein NDRG1 OS=Homo sapiens GN=NDRG1 PE=1 SV=1 - [NDRG1_HUMAN]	6

Isoform 2 of NADH dehydrogenase [ubiquinone] iron-sulfur protein 2, mitochondrial OS=Homo sapiens GN=NDUFS2 - [NDUS2_HUMAN]	6
Prenylcysteine oxidase 1 OS=Homo sapiens GN=PCYOX1 PE=1 SV=3 - [PCYOX_HUMAN]	6
Peripherin OS=Homo sapiens GN=PRPH PE=1 SV=2 - [PERI_HUMAN]	6
Phytanoyl-CoA hydroxylase-interacting protein OS=Homo sapiens GN=PHYHIP PE=1 SV=1 - [PHYIP_HUMAN]	6
Isoform 3 of Phosphatidylinositol 5-phosphate 4-kinase type-2 gamma OS=Homo sapiens GN=PIP4K2C - [PI42C_HUMAN]	6
Protein-L-isoaspartate(D-aspartate) O-methyltransferase OS=Homo sapiens GN=PCMT1 PE=1 SV=4 - [PIMT_HUMAN]	6
Peptidyl-prolyl cis-trans isomerase B OS=Homo sapiens GN=PPIB PE=1 SV=2 - [PPIB_HUMAN]	6
Peroxiredoxin-6 OS=Homo sapiens GN=PRDX6 PE=1 SV=3 - [PRDX6_HUMAN]	6
26S proteasome non-ATPase regulatory subunit 2 OS=Homo sapiens GN=PSMD2 PE=1 SV=3 - [PSMD2_HUMAN]	6
Quinone oxidoreductase OS=Homo sapiens GN=CRYZ PE=1 SV=1 - [QOR_HUMAN]	6
Ras-related protein Rab-5B OS=Homo sapiens GN=RAB5B PE=1 SV=1 - [RAB5B_HUMAN]	6
Ras-related protein Ral-A OS=Homo sapiens GN=RALA PE=1 SV=1 - [RALA_HUMAN]	6
Synaptic vesicle membrane protein VAT-1 homolog OS=Homo sapiens GN=VAT1 PE=1 SV=2 - [VAT1_HUMAN]	6
Voltage-dependent anion-selective channel protein 3 OS=Homo sapiens GN=VDAC3 PE=1 SV=1 - [VDAC3_HUMAN]	6
Catenin beta-1 OS=Homo sapiens GN=CTNNB1 PE=1 SV=1 - [B4DGU4_HUMAN]	6
CaM kinase-like vesicle-associated protein OS=Homo sapiens GN=CAMKV PE=1 SV=1 - [B4DM24_HUMAN]	6
PH and SEC7 domain-containing protein 3 OS=Homo sapiens GN=PSD3 PE=1 SV=1 - [B4DKF8_HUMAN]	6
Septin-2 OS=Homo sapiens GN=SEPT2 PE=1 SV=1 - [B5MCX3_HUMAN]	6
Isoform 2 of Thioredoxin-dependent peroxide reductase, mitochondrial OS=Homo sapiens GN=PRDX3 - [PRDX3_HUMAN]	6
Sorcin OS=Homo sapiens GN=SRI PE=1 SV=1 - [C9J0K6_HUMAN]	6
Heat shock 70 kDa protein 4L (Fragment) OS=Homo sapiens GN=HSPA4L PE=1 SV=1 - [D6RJ96_HUMAN]	6
Ras-related protein Rab-11A (Fragment) OS=Homo sapiens GN=RAB11A PE=3 SV=1 - [H3BMH2_HUMAN]	6
Protein disulfide-isomerase A3 (Fragment) OS=Homo sapiens GN=PDIA3 PE=1 SV=1 - [H7BZJ3_HUMAN]	6
Malic enzyme (Fragment) OS=Homo sapiens GN=ME3 PE=1 SV=4 - [E9PMB9_HUMAN]	6
cAMP-dependent protein kinase catalytic subunit beta OS=Homo sapiens GN=PRKACB PE=1 SV=1 - [A0A087WVC4_HUMAN]	6
Glutathione S-transferase Mu 3 OS=Homo sapiens GN=GSTM3 PE=1 SV=1 - [A0A0A0MTN3_HUMAN]	6
Beta-centractin OS=Homo sapiens GN=ACTR1B PE=1 SV=1 - [ACTY_HUMAN]	5
Alpha-centractin OS=Homo sapiens GN=ACTR1A PE=1 SV=1 - [ACTZ_HUMAN]	5
Isoform 2 of Beta-adducin OS=Homo sapiens GN=ADD2 - [ADDB_HUMAN]	5
Isoform 3 of Apoptosis-inducing factor 1, mitochondrial OS=Homo sapiens GN=AIFM1 - [AIFM1_HUMAN]	5
Cytosolic 10-formyltetrahydrofolate dehydrogenase OS=Homo sapiens GN=ALDH1L1 PE=1 SV=2 - [AL1L1_HUMAN]	5
Amine oxidase [flavin-containing] A OS=Homo sapiens GN=MAOA PE=1 SV=1 - [AOFA_HUMAN]	5
Apolipoprotein D OS=Homo sapiens GN=APOD PE=1 SV=1 - [APOD_HUMAN]	5
Calmodulin OS=Homo sapiens GN=CALM1 PE=1 SV=2 - [CALM_HUMAN]	5
Calreticulin OS=Homo sapiens GN=CALR PE=1 SV=1 - [CALR_HUMAN]	5
Isoform 2 of Adenylyl cyclase-associated protein 1 OS=Homo sapiens GN=CAP1 - [CAP1_HUMAN]	5
Isoform 2 of CD166 antigen OS=Homo sapiens GN=ALCAM - [CD166_HUMAN]	5
Cell division control protein 42 homolog OS=Homo sapiens GN=CDC42 PE=1 SV=2 - [CDC42_HUMAN]	5
Isoform 3 of Catenin alpha-2 OS=Homo sapiens GN=CTNNA2 - [CTNA2_HUMAN]	5
Gap junction alpha-1 protein OS=Homo sapiens GN=GJA1 PE=1 SV=2 - [CXA1_HUMAN]	5
Isoform 2 of Probable ATP-dependent RNA helicase DDX17 OS=Homo sapiens GN=DDX17 - [DDX17_HUMAN]	5
Isoform 3 of Dynamin-1-like protein OS=Homo sapiens GN=DNM1L - [DNM1L_HUMAN]	5
Enoyl-CoA hydratase, mitochondrial OS=Homo sapiens GN=ECHS1 PE=1 SV=4 - [ECHM_HUMAN]	5
Emerin OS=Homo sapiens GN=EMD PE=1 SV=1 - [EMD_HUMAN]	5
Endonuclease domain-containing 1 protein OS=Homo sapiens GN=ENDOD1 PE=1 SV=2 - [ENDD1_HUMAN]	5
Electron transfer flavoprotein subunit beta OS=Homo sapiens GN=ETFB PE=1 SV=3 - [ETFB_HUMAN]	5
Protein FAM49A OS=Homo sapiens GN=FAM49A PE=1 SV=1 - [FA49A_HUMAN]	5
Isoform Cytoplasmic of Fumarate hydratase, mitochondrial OS=Homo sapiens GN=FH - [FUMH_HUMAN]	5
Isoform 3 of Tyrosine-protein kinase Fyn OS=Homo sapiens GN=FYN - [FYN_HUMAN]	5
Guanine nucleotide-binding protein subunit beta-4 OS=Homo sapiens GN=GNB4 PE=1 SV=3 - [GBB4_HUMAN]	5
Guanine nucleotide-binding protein subunit alpha-11 OS=Homo sapiens GN=GNA11 PE=1 SV=2 - [GNA11_HUMAN]	5
Guanine deaminase OS=Homo sapiens GN=GDA PE=1 SV=1 - [GUAD_HUMAN]	5
Histone H2A type 1-B/E OS=Homo sapiens GN=HIST1H2AB PE=1 SV=2 - [H2A1B_HUMAN]	5

Histone H2A type 1-H OS=Homo sapiens GN=HIST1H2AH PE=1 SV=3 - [H2A1H_HUMAN]	5
Histone H2B type 1-K OS=Homo sapiens GN=HIST1H2BK PE=1 SV=3 - [H2B1K_HUMAN]	5
Hemoglobin subunit alpha OS=Homo sapiens GN=HBA1 PE=1 SV=2 - [HBA_HUMAN]	5
Heterogeneous nuclear ribonucleoprotein H2 OS=Homo sapiens GN=HNRNPH2 PE=1 SV=1 - [HNRH2_HUMAN]	5
Epoxide hydrolase 1 OS=Homo sapiens GN=EPHX1 PE=1 SV=1 - [HYEP_HUMAN]	5
Keratin, type II cytoskeletal 2 epidermal OS=Homo sapiens GN=KRT2 PE=1 SV=2 - [K22E_HUMAN]	5
ATP-dependent 6-phosphofructokinase, muscle type OS=Homo sapiens GN=PFKM PE=1 SV=2 - [PFKAM_HUMAN]	5
Adenylate kinase isoenzyme 1 OS=Homo sapiens GN=AK1 PE=1 SV=3 - [KAD1_HUMAN]	5
Adenylate kinase 4, mitochondrial OS=Homo sapiens GN=AK4 PE=1 SV=1 - [KAD4_HUMAN]	5
Isoform 2 of cAMP-dependent protein kinase catalytic subunit alpha OS=Homo sapiens GN=PRKACA - [KAPCA_HUMAN]	5
Creatine kinase U-type, mitochondrial OS=Homo sapiens GN=CKMT1A PE=1 SV=1 - [KCRU_HUMAN]	5
Lamin-B1 OS=Homo sapiens GN=LMNB1 PE=1 SV=2 - [LMNB1_HUMAN]	5
Metallo-beta-lactamase domain-containing protein 2 OS=Homo sapiens GN=MBLAC2 PE=1 SV=3 - [MBLC2_HUMAN]	5
Isoform 2 of Mitogen-activated protein kinase 1 OS=Homo sapiens GN=MAPK1 - [MK01_HUMAN]	5
Moesin OS=Homo sapiens GN=MSN PE=1 SV=3 - [MOES_HUMAN]	5
Myelin proteolipid protein OS=Homo sapiens GN=PLP1 PE=1 SV=2 - [MYPR_HUMAN]	5
Neurocan core protein OS=Homo sapiens GN=NCAN PE=1 SV=3 - [NCAN_HUMAN]	5
Nck-associated protein 1 OS=Homo sapiens GN=NCKAP1 PE=1 SV=1 - [NCKP1_HUMAN]	5
Isoform 2 of Protein NDRG4 OS=Homo sapiens GN=NDRG4 - [NDRG4_HUMAN]	5
NADH dehydrogenase [ubiquinone] 1 alpha subcomplex subunit 5 OS=Homo sapiens GN=NDUFA5 PE=1 SV=3 - [NDUA5_HUMAN]	5
NADH dehydrogenase [ubiquinone] 1 alpha subcomplex subunit 12 OS=Homo sapiens GN=NDUFA12 PE=1 SV=1 - [NDUAC_HUMAN]	5
Protein NipSnap homolog 1 OS=Homo sapiens GN=NIPSNAP1 PE=1 SV=1 - [NIPS1_HUMAN]	5
Isoform 3 of Neuroplastin OS=Homo sapiens GN=NPTN - [NPTN_HUMAN]	5
Isoform 3 of Neuronal cell adhesion molecule OS=Homo sapiens GN=NRCAM - [NRCAM_HUMAN]	5
Poly [ADP-ribose] polymerase 1 OS=Homo sapiens GN=PARP1 PE=1 SV=4 - [PARP1_HUMAN]	5
ProSAAS OS=Homo sapiens GN=PCSK1N PE=1 SV=1 - [PCSK1_HUMAN]	5
Programmed cell death protein 6 OS=Homo sapiens GN=PDCD6 PE=1 SV=1 - [PDCD6_HUMAN]	5
Isoform 2 of 26S proteasome non-ATPase regulatory subunit 1 OS=Homo sapiens GN=PSMD1 - [PSMD1_HUMAN]	5
Glycogen phosphorylase, muscle form OS=Homo sapiens GN=PYGM PE=1 SV=6 - [PYGM_HUMAN]	5
Ras-related protein Rab-10 OS=Homo sapiens GN=RAB10 PE=1 SV=1 - [RAB10_HUMAN]	5
Ras-related protein Rab-3B OS=Homo sapiens GN=RAB3B PE=1 SV=2 - [RAB3B_HUMAN]	5
Ras-related protein Rab-3C OS=Homo sapiens GN=RAB3C PE=2 SV=1 - [RAB3C_HUMAN]	5
Ras-related protein Rab-6B OS=Homo sapiens GN=RAB6B PE=1 SV=1 - [RAB6B_HUMAN]	5
Ras-related C3 botulinum toxin substrate 3 OS=Homo sapiens GN=RAC3 PE=1 SV=1 - [RAC3_HUMAN]	5
Radixin OS=Homo sapiens GN=RDX PE=1 SV=1 - [RADI_HUMAN]	5
Isoform 3 of Ras-related protein Rap-1b OS=Homo sapiens GN=RAP1B - [RAP1B_HUMAN]	5
Transforming protein RhoA OS=Homo sapiens GN=RHOA PE=1 SV=1 - [RHOA_HUMAN]	5
Isoform 2 of Heterogeneous nuclear ribonucleoprotein A3 OS=Homo sapiens GN=HNRNPA3 - [ROA3_HUMAN]	5
Dolichyl-diphosphooligosaccharide--protein glycosyltransferase subunit 1 OS=Homo sapiens GN=RPN1 PE=1 SV=1 - [RPN1_HUMAN]	5
40S ribosomal protein S4, X isoform OS=Homo sapiens GN=RPS4X PE=1 SV=2 - [RS4X_HUMAN]	5
Isoform RTN1-B of Reticulon-1 OS=Homo sapiens GN=RTN1 - [RTN1_HUMAN]	5
Isoform 4 of Reticulon-4 OS=Homo sapiens GN=RTN4 - [RTN4_HUMAN]	5
Sorting and assembly machinery component 50 homolog OS=Homo sapiens GN=SAMM50 PE=1 SV=3 - [SAM50_HUMAN]	5
Isoform 5 of Septin-4 OS=Homo sapiens GN=SEPT4 - [SEPT4_HUMAN]	5
Phosphoserine aminotransferase OS=Homo sapiens GN=PSAT1 PE=1 SV=2 - [SERC_HUMAN]	5
Isoform Short of Splicing factor, proline- and glutamine-rich OS=Homo sapiens GN=SFPQ - [SFPQ_HUMAN]	5
Erythrocyte band 7 integral membrane protein OS=Homo sapiens GN=STOM PE=1 SV=3 - [STOM_HUMAN]	5
Phenylalanine--tRNA ligase alpha subunit OS=Homo sapiens GN=FARSA PE=1 SV=3 - [SYFA_HUMAN]	5
Phenylalanine--tRNA ligase beta subunit OS=Homo sapiens GN=FARSB PE=1 SV=3 - [SYFB_HUMAN]	5
Isoleucine--tRNA ligase, mitochondrial OS=Homo sapiens GN=IARS2 PE=1 SV=2 - [SYIM_HUMAN]	5
Isoform 2-4 of Alpha-synuclein OS=Homo sapiens GN=SNCA - [SYUA_HUMAN]	5
Transaldolase OS=Homo sapiens GN=TALDO1 PE=1 SV=2 - [TALDO_HUMAN]	5
Isoform 2 of Tubulin alpha chain-like 3 OS=Homo sapiens GN=TUBAL3 - [TBAL3_HUMAN]	5

Isoform 4 of Tumor protein D52 OS=Homo sapiens GN=TPD52 - [TPD52_HUMAN]	5
Isoform 4 of Tropomyosin alpha-3 chain OS=Homo sapiens GN=TPM3 - [TPM3_HUMAN]	5
Tubulin polymerization-promoting protein OS=Homo sapiens GN=TPPP PE=1 SV=1 - [TPPP_HUMAN]	5
Tricarboxylate transport protein, mitochondrial OS=Homo sapiens GN=SLC25A1 PE=1 SV=2 - [TXTP_HUMAN]	5
Isoform 2 of V-type proton ATPase 116 kDa subunit a isoform 1 OS=Homo sapiens GN=ATP6V0A1 - [VPP1_HUMAN]	5
Isoform 3 of AP-3 complex subunit beta-2 OS=Homo sapiens GN=AP3B2 - [AP3B2_HUMAN]	5
Hypoxia up-regulated protein 1 OS=Homo sapiens GN=HYOU1 PE=1 SV=1 - [A0A087X054_HUMAN]	5
Protein phosphatase 3 (Formerly 2B), catalytic subunit, beta isoform (Calcineurin A beta), isoform CRA_a OS=Homo sapiens GN=PPP3CB PE=1 SV=1 - [Q5F2F8_HUMAN]	5
Capping protein (Actin filament) muscle Z-line, beta, isoform CRA_a OS=Homo sapiens GN=CAPZB PE=1 SV=1 - [B1AK87_HUMAN]	5
Flotillin-2 OS=Homo sapiens GN=FLOT2 PE=1 SV=1 - [J3QLD9_HUMAN]	5
Cofilin 1 (Non-muscle), isoform CRA_a OS=Homo sapiens GN=CFL1 PE=1 SV=1 - [G3V1A4_HUMAN]	5
Erlin-2 (Fragment) OS=Homo sapiens GN=ERLIN2 PE=1 SV=1 - [E5RHW4_HUMAN]	5
Copine-8 OS=Homo sapiens GN=CPNE8 PE=1 SV=2 - [E7ENV7_HUMAN]	5
Mitogen-activated protein kinase kinase kinase kinase 4 OS=Homo sapiens GN=MAP4K4 PE=1 SV=1 - [E7ENQ1_HUMAN]	5
Adenylyl cyclase-associated protein OS=Homo sapiens GN=CAP2 PE=1 SV=1 - [E9PDI2_HUMAN]	5
ATP synthase subunit g, mitochondrial OS=Homo sapiens GN=ATP5L PE=1 SV=1 - [E9PN17_HUMAN]	5
V-type proton ATPase subunit B, kidney isoform OS=Homo sapiens GN=ATP6V1B1 PE=1 SV=1 - [C9JL73_HUMAN]	5
Acid ceramidase OS=Homo sapiens GN=ASAH1 PE=1 SV=1 - [E7EMM4_HUMAN]	5
Isoform 3 of Excitatory amino acid transporter 2 OS=Homo sapiens GN=SLC1A2 - [EAA2_HUMAN]	5
Myosin light polypeptide 6 OS=Homo sapiens GN=MYL6 PE=1 SV=1 - [F8W1R7_HUMAN]	5
40S ribosomal protein S3 OS=Homo sapiens GN=RPS3 PE=1 SV=1 - [F2Z2S8_HUMAN]	5
Ras/Rap GTPase-activating protein SynGAP (Fragment) OS=Homo sapiens GN=SYNGAP1 PE=1 SV=2 - [B7ZCA0_HUMAN]	5
Isoform 3 of Pyruvate dehydrogenase protein X component, mitochondrial OS=Homo sapiens GN=PDHX - [ODPX_HUMAN]	5
Ras-related protein Rab-35 (Fragment) OS=Homo sapiens GN=RAB35 PE=1 SV=1 - [F5H157_HUMAN]	5
60S acidic ribosomal protein P0 (Fragment) OS=Homo sapiens GN=RPLP0 PE=1 SV=1 - [F8VW21_HUMAN]	5
Cytochrome c (Fragment) OS=Homo sapiens GN=CYCS PE=1 SV=1 - [C9JFR7_HUMAN]	5
NADH dehydrogenase [ubiquinone] 1 beta subcomplex subunit 10 OS=Homo sapiens GN=NDUFB10 PE=1 SV=1 - [H3BPJ9_HUMAN]	5
Ras-related protein Rab-6A (Fragment) OS=Homo sapiens GN=RAB6A PE=1 SV=1 - [H7BYW1_HUMAN]	5
Mitogen-activated protein kinase 10 (Fragment) OS=Homo sapiens GN=MAPK10 PE=1 SV=1 - [H0Y9H3_HUMAN]	5
Septin-9 (Fragment) OS=Homo sapiens GN=SEPT9 PE=1 SV=1 - [K7EK18_HUMAN]	5
Isoform 5 of Cell adhesion molecule 2 OS=Homo sapiens GN=CADM2 - [CADM2_HUMAN]	5
Isoform 2 of Guanine nucleotide-binding protein G(I)/G(S)/G(T) subunit beta-1 OS=Homo sapiens GN=GNB1 - [GBB1_HUMAN]	5
Heterogeneous nuclear ribonucleoprotein Q OS=Homo sapiens GN=SYNCRIP PE=1 SV=1 - [B7Z645_HUMAN]	5
ARP2 actin-related protein 2 homolog (Yeast), isoform CRA_d OS=Homo sapiens GN=ACTR2 PE=1 SV=2 - [F5H6T1_HUMAN]	5
Mitochondrial glutamate carrier 1 (Fragment) OS=Homo sapiens GN=SLC25A22 PE=1 SV=4 - [E9PJH7_HUMAN]	5
Regulator of G-protein-signaling 7 OS=Homo sapiens GN=RGS7 PE=1 SV=1 - [A0A0A0MSE0_HUMAN]	5
Mitochondrial carrier homolog 2 (Fragment) OS=Homo sapiens GN=MTCH2 PE=1 SV=4 - [E9PIE4_HUMAN]	5
Neural cell adhesion molecule 2 OS=Homo sapiens GN=NCAM2 PE=1 SV=2 - [H9KV31_HUMAN]	5
TAR DNA-binding protein 43 (Fragment) OS=Homo sapiens GN=TARDBP PE=1 SV=1 - [A0A087WX29_HUMAN]	5
Protein LOC102724023 OS=Homo sapiens GN=LOC102724023 PE=4 SV=1 - [A0A096LNH5_HUMAN]	5
Isoform 7 of Long-chain-fatty-acid--CoA ligase 6 OS=Homo sapiens GN=ACSL6 - [ACSL6_HUMAN]	4
Isoform 3 of Arf-GAP with GTPase, ANK repeat and PH domain-containing protein 3 OS=Homo sapiens GN=AGAP3 - [AGAP3_HUMAN]	4
4-trimethylaminobutyraldehyde dehydrogenase OS=Homo sapiens GN=ALDH9A1 PE=1 SV=3 - [AL9A1_HUMAN]	4
Isoform 2 of Amphiphysin OS=Homo sapiens GN=AMPH - [AMPH_HUMAN]	4
Isoform 3 of ATPase family AAA domain-containing protein 3A OS=Homo sapiens GN=ATAD3A - [ATD3A_HUMAN]	4
Isoform 4 of Putative tyrosine-protein phosphatase auxilin OS=Homo sapiens GN=DNAJC6 - [AUXI_HUMAN]	4
Cell adhesion molecule 4 OS=Homo sapiens GN=CADM4 PE=1 SV=1 - [CADM4_HUMAN]	4
Carbonic anhydrase 2 OS=Homo sapiens GN=CA2 PE=1 SV=2 - [CAH2_HUMAN]	4
Isoform 2 of Calcium-dependent secretion activator 1 OS=Homo sapiens GN=CADPS - [CAPS1_HUMAN]	4
10 kDa heat shock protein, mitochondrial OS=Homo sapiens GN=HSPE1 PE=1 SV=2 - [CH10_HUMAN]	4
Cytochrome c oxidase subunit 5B, mitochondrial OS=Homo sapiens GN=COX5B PE=1 SV=2 - [COX5B_HUMAN]	4
Isoform 5 of Dynactin subunit 1 OS=Homo sapiens GN=DCTN1 - [DCTN1_HUMAN]	4

Peroxisomal multifunctional enzyme type 2 OS=Homo sapiens GN=HSD17B4 PE=1 SV=3 - [DHB4_HUMAN]	4
Isoform 2 of DmX-like protein 2 OS=Homo sapiens GN=DMXL2 - [DMXL2_HUMAN]	4
Dihydropyrimidinase-related protein 4 OS=Homo sapiens GN=DPYSL4 PE=1 SV=2 - [DPYL4_HUMAN]	4
Dihydropyrimidinase-related protein 5 OS=Homo sapiens GN=DPYSL5 PE=1 SV=1 - [DPYL5_HUMAN]	4
F-box only protein 2 OS=Homo sapiens GN=FBXO2 PE=1 SV=2 - [FBX2_HUMAN]	4
Isoform 2 of Glutathione S-transferase Mu 2 OS=Homo sapiens GN=GSTM2 - [GSTM2_HUMAN]	4
Isoform 2 of Histone H1.0 OS=Homo sapiens GN=H1F0 - [H10_HUMAN]	4
Isoform 3 of Heterogeneous nuclear ribonucleoprotein D-like OS=Homo sapiens GN=HNRNPDL - [HNRDL_HUMAN]	4
Isoform 2 of Heterogeneous nuclear ribonucleoprotein H3 OS=Homo sapiens GN=HNRNPH3 - [HNRH3_HUMAN]	4
Hippocalcin-like protein 4 OS=Homo sapiens GN=HPCAL4 PE=1 SV=3 - [HPCL4_HUMAN]	4
Intercellular adhesion molecule 5 OS=Homo sapiens GN=ICAM5 PE=1 SV=3 - [ICAM5_HUMAN]	4
Protein KIAA1045 OS=Homo sapiens GN=KIAA1045 PE=1 SV=2 - [K1045_HUMAN]	4
cAMP-dependent protein kinase type II-beta regulatory subunit OS=Homo sapiens GN=PRKAR2B PE=1 SV=3 - [KAP3_HUMAN]	4
Guanylate kinase OS=Homo sapiens GN=GUK1 PE=1 SV=2 - [KGUA_HUMAN]	4
Protein kinase C beta type OS=Homo sapiens GN=PRKCB PE=1 SV=4 - [KPCB_HUMAN]	4
LETM1 and EF-hand domain-containing protein 1, mitochondrial OS=Homo sapiens GN=LETM1 PE=1 SV=1 - [LETM1_HUMAN]	4
Leucine-rich PPR motif-containing protein, mitochondrial OS=Homo sapiens GN=LRPPRC PE=1 SV=3 - [LPPRC_HUMAN]	4
Dual specificity mitogen-activated protein kinase kinase 2 OS=Homo sapiens GN=MAP2K2 PE=1 SV=1 - [MP2K2_HUMAN]	4
Neurocalcin-delta OS=Homo sapiens GN=NCALD PE=1 SV=2 - [NCALD_HUMAN]	4
NADH dehydrogenase [ubiquinone] 1 alpha subcomplex subunit 8 OS=Homo sapiens GN=NDUFA8 PE=1 SV=3 - [NDUA8_HUMAN]	4
NADH dehydrogenase [ubiquinone] flavoprotein 2, mitochondrial OS=Homo sapiens GN=NDUFV2 PE=1 SV=2 - [NDUV2_HUMAN]	4
Na(+)/H(+) exchange regulatory cofactor NHE-RF1 OS=Homo sapiens GN=SLC9A3R1 PE=1 SV=4 - [NHRF1_HUMAN]	4
Isoform 3 of Neurotrimin OS=Homo sapiens GN=NTM - [NTRI_HUMAN]	4
Lipoamide acyltransferase component of branched-chain alpha-keto acid dehydrogenase complex, mitochondrial OS=Homo sapiens GN=DBT PE=1 SV=3 - [ODB2_HUMAN]	4
Oligodendrocyte-myelin glycoprotein OS=Homo sapiens GN=OMG PE=1 SV=2 - [OMGP_HUMAN]	4
Isoform 2 of Opioid-binding protein/cell adhesion molecule OS=Homo sapiens GN=OPCML - [OPCM_HUMAN]	4
Isoform 3 of Optineurin OS=Homo sapiens GN=OPTN - [OPTN_HUMAN]	4
Membrane-associated progesterone receptor component 2 OS=Homo sapiens GN=PGRMC2 PE=1 SV=1 - [PGRC2_HUMAN]	4
Isoform 4 of Phosphatidylinositol 4-phosphate 5-kinase type-1 gamma OS=Homo sapiens GN=PIP5K1C - [PI51C_HUMAN]	4
Serine/threonine-protein phosphatase PP1-beta catalytic subunit OS=Homo sapiens GN=PPP1CB PE=1 SV=3 - [PP1B_HUMAN]	4
Palmitoyl-protein thioesterase 1 OS=Homo sapiens GN=PPT1 PE=1 SV=1 - [PPT1_HUMAN]	4
Isoform 2 of Major prion protein OS=Homo sapiens GN=PRNP - [PRIO_HUMAN]	4
Isoform 2 of 26S protease regulatory subunit 8 OS=Homo sapiens GN=PSMC5 - [PRS8_HUMAN]	4
Transcriptional activator protein Pur-alpha OS=Homo sapiens GN=PURA PE=1 SV=2 - [PURA_HUMAN]	4
Cytochrome b-c1 complex subunit 7 OS=Homo sapiens GN=UQCRB PE=1 SV=2 - [QCR7_HUMAN]	4
Ras-related protein Rab-1A OS=Homo sapiens GN=RAB1A PE=1 SV=3 - [RAB1A_HUMAN]	4
Ras-related protein Rab-1B OS=Homo sapiens GN=RAB1B PE=1 SV=1 - [RAB1B_HUMAN]	4
Ras-related protein Ral-B OS=Homo sapiens GN=RALB PE=1 SV=1 - [RALB_HUMAN]	4
GTP-binding nuclear protein Ran OS=Homo sapiens GN=RAN PE=1 SV=3 - [RAN_HUMAN]	4
Isoform 2B of GTPase KRas OS=Homo sapiens GN=KRAS - [RASK_HUMAN]	4
60S ribosomal protein L3 OS=Homo sapiens GN=RPL3 PE=1 SV=2 - [RL3_HUMAN]	4
60S ribosomal protein L4 OS=Homo sapiens GN=RPL4 PE=1 SV=5 - [RL4_HUMAN]	4
40S ribosomal protein S18 OS=Homo sapiens GN=RPS18 PE=1 SV=3 - [RS18_HUMAN]	4
Isoform RTN1-C of Reticulon-1 OS=Homo sapiens GN=RTN1 - [RTN1_HUMAN]	4
Isoform 3 of Reticulon-3 OS=Homo sapiens GN=RTN3 - [RTN3_HUMAN]	4
Isoform 3 of Reticulon-4 OS=Homo sapiens GN=RTN4 - [RTN4_HUMAN]	4
RuvB-like 2 OS=Homo sapiens GN=RUVBL2 PE=1 SV=3 - [RUVB2_HUMAN]	4
Endophilin-A1 OS=Homo sapiens GN=SH3GL2 PE=1 SV=1 - [SH3G2_HUMAN]	4
Isoform 3 of NCK-interacting protein with SH3 domain OS=Homo sapiens GN=NCKIPSD - [SPN90_HUMAN]	4
Isoform 2 of SLIT-ROBO Rho GTPase-activating protein 3 OS=Homo sapiens GN=SRGAP3 - [SRGP3_HUMAN]	4
Stathmin OS=Homo sapiens GN=STMN1 PE=1 SV=3 - [STMN1_HUMAN]	4
Succinyl-CoA ligase [ADP/GDP-forming] subunit alpha, mitochondrial OS=Homo sapiens GN=SUCLG1 PE=1 SV=4 - [SUCA_HUMAN]	4

Isoform Monomeric of Arginine--tRNA ligase, cytoplasmic OS=Homo sapiens GN=RARS - [SYRC_HUMAN]	4
Isoform Tau-A of Microtubule-associated protein tau OS=Homo sapiens GN=MAPT - [TAU_HUMAN]	4
Isoform Tau-D of Microtubule-associated protein tau OS=Homo sapiens GN=MAPT - [TAU_HUMAN]	4
Isoform 2 of Tripeptidyl-peptidase 1 OS=Homo sapiens GN=TPP1 - [TPP1_HUMAN]	4
Tripartite motif-containing protein 2 OS=Homo sapiens GN=TRIM2 PE=1 SV=1 - [TRIM2_HUMAN]	4
Isoform 4 of Tripartite motif-containing protein 3 OS=Homo sapiens GN=TRIM3 - [TRIM3_HUMAN]	4
Isoform 1 of Vinculin OS=Homo sapiens GN=VCL - [VINC_HUMAN]	4
Isoform 2 of 26S protease regulatory subunit 7 OS=Homo sapiens GN=PSMC2 - [PRS7_HUMAN]	4
Proline dehydrogenase 1, mitochondrial OS=Homo sapiens GN=PRODH PE=1 SV=1 - [E7EQL6_HUMAN]	4
Isoform 2 of X-ray repair cross-complementing protein 6 OS=Homo sapiens GN=XRCC6 - [XRCC6_HUMAN]	4
60S ribosomal protein L7 OS=Homo sapiens GN=RPL7 PE=1 SV=1 - [A8MUD9_HUMAN]	4
Active breakpoint cluster region-related protein OS=Homo sapiens GN=ABR PE=1 SV=1 - [B7Z683_HUMAN]	4
ANXA4 protein OS=Homo sapiens GN=ANXA4 PE=1 SV=1 - [Q6P452_HUMAN]	4
Isoform 2 of 26S proteasome non-ATPase regulatory subunit 3 OS=Homo sapiens GN=PSMD3 - [PSMD3_HUMAN]	4
Catenin delta-2 OS=Homo sapiens GN=CTNND2 PE=1 SV=1 - [B4DRK2_HUMAN]	4
Casein kinase II subunit alpha OS=Homo sapiens GN=CSNK2A1 PE=1 SV=1 - [E7EU96_HUMAN]	4
NADH dehydrogenase [ubiquinone] iron-sulfur protein 8, mitochondrial (Fragment) OS=Homo sapiens GN=NDUFS8 PE=1 SV=1 - [E9PN51_HUMAN]	4
40S ribosomal protein S8 OS=Homo sapiens GN=RPS8 PE=1 SV=1 - [Q5JR95_HUMAN]	4
Unconventional myosin-Va (Fragment) OS=Homo sapiens GN=MYO5A PE=1 SV=2 - [E7ERV5_HUMAN]	4
ELAV-like protein (Fragment) OS=Homo sapiens GN=ELAVL2 PE=1 SV=1 - [B1AM48_HUMAN]	4
Transgelin-3 OS=Homo sapiens GN=TAGLN3 PE=1 SV=1 - [C9J5W6_HUMAN]	4
Tropomyosin alpha-1 chain OS=Homo sapiens GN=TPM1 PE=1 SV=2 - [F5H7S3_HUMAN]	4
Ras-related protein Rab-7a (Fragment) OS=Homo sapiens GN=RAB7A PE=1 SV=1 - [C9J592_HUMAN]	4
Polyubiquitin-C (Fragment) OS=Homo sapiens GN=UBC PE=1 SV=1 - [F5H265_HUMAN]	4
DnaJ homolog subfamily B member 2 (Fragment) OS=Homo sapiens GN=DNAJB2 PE=1 SV=1 - [C9JXB9_HUMAN]	4
Serine/threonine-protein phosphatase OS=Homo sapiens GN=PPP1CC PE=1 SV=1 - [F8VYE8_HUMAN]	4
Phosphate carrier protein, mitochondrial OS=Homo sapiens GN=SLC25A3 PE=1 SV=1 - [F8VVM2_HUMAN]	4
Cysteine and glycine-rich protein 1 OS=Homo sapiens GN=CSRP1 PE=1 SV=1 - [E9PP21_HUMAN]	4
Fatty acid-binding protein, heart (Fragment) OS=Homo sapiens GN=FABP3 PE=1 SV=1 - [S4R371_HUMAN]	4
Apolipoprotein O (Fragment) OS=Homo sapiens GN=APOO PE=1 SV=1 - [H7C1U8_HUMAN]	4
Protein deglycase DJ-1 OS=Homo sapiens GN=PARK7 PE=1 SV=1 - [K7ELW0_HUMAN]	4
Histone H3 (Fragment) OS=Homo sapiens GN=H3F3B PE=1 SV=1 - [K7EK07_HUMAN]	4
Unconventional myosin-Id OS=Homo sapiens GN=MYO1D PE=1 SV=1 - [K7EIG7_HUMAN]	4
ARF GTPase-activating protein GIT1 OS=Homo sapiens GN=GIT1 PE=1 SV=1 - [J3QRU8_HUMAN]	4
NADH dehydrogenase [ubiquinone] 1 alpha subcomplex subunit 6 OS=Homo sapiens GN=NDUFA6 PE=4 SV=1 - [A0A0C4DGS0_HUMAN]	4
ATPase ASNA1 OS=Homo sapiens GN=ASNA1 PE=1 SV=1 - [A0A087WXS7_HUMAN]	4
Thy-1 membrane glycoprotein (Fragment) OS=Homo sapiens GN=THY1 PE=1 SV=4 - [E9PIM6_HUMAN]	4
Cytoplasmic dynein 1 intermediate chain 1 OS=Homo sapiens GN=DYNC1I1 PE=1 SV=1 - [A0A0A0MTG2_HUMAN]	4
Electron transfer flavoprotein subunit alpha, mitochondrial (Fragment) OS=Homo sapiens GN=ETFA PE=1 SV=4 - [H0YL12_HUMAN]	4
Basigin (Fragment) OS=Homo sapiens GN=BSG PE=1 SV=1 - [A0A087X2B5_HUMAN]	4
Myristoylated alanine-rich C-kinase substrate OS=Homo sapiens GN=MARCKS PE=1 SV=1 - [A0A087WZH7_HUMAN]	4
Gamma-adducin OS=Homo sapiens GN=ADD3 PE=1 SV=1 - [A0A087WX08_HUMAN]	4
WD repeat-containing protein 13 (Fragment) OS=Homo sapiens GN=WDR13 PE=1 SV=1 - [A0A087X091_HUMAN]	4
Cullin-associated NEDD8-dissociated protein 1 (Fragment) OS=Homo sapiens GN=CAND1 PE=4 SV=1 - [A0A0C4DGH5_HUMAN]	4
Isoform Delta-3 of Serine/threonine-protein phosphatase 2A 56 kDa regulatory subunit delta isoform OS=Homo sapiens GN=PPP2R5D - [2A5D_HUMAN]	3
Serine/threonine-protein phosphatase 2A 55 kDa regulatory subunit B alpha isoform OS=Homo sapiens GN=PPP2R2A PE=1 SV=1 - [2ABA_HUMAN]	3
Isoform 2 of Acyl-coenzyme A thioesterase 13 OS=Homo sapiens GN=ACOT13 - [ACO13_HUMAN]	3
Isoform 3 of Acyl-coenzyme A thioesterase 9, mitochondrial OS=Homo sapiens GN=ACOT9 - [ACOT9_HUMAN]	3
Adipocyte plasma membrane-associated protein OS=Homo sapiens GN=APMAP PE=1 SV=2 - [APMAP_HUMAN]	3
Isoform 3 of Rho guanine nucleotide exchange factor 2 OS=Homo sapiens GN=ARHGEF2 - [ARHG2_HUMAN]	3
Actin-related protein 2/3 complex subunit 2 OS=Homo sapiens GN=ARPC2 PE=1 SV=1 - [ARPC2_HUMAN]	3
Isoform 5 of Alpha-tubulin N-acetyltransferase 1 OS=Homo sapiens GN=ATAT1 - [ATAT_HUMAN]	3
Isoform 2 of Cytosolic acyl coenzyme A thioester hydrolase OS=Homo sapiens GN=ACOT7 - [BACH_HUMAN]	3

Isoform 3 of Brain-specific angiogenesis inhibitor 1-associated protein 2 OS=Homo sapiens GN=BAIAP2 - [BAIP2_HUMAN]	3
B-cell receptor-associated protein 31 OS=Homo sapiens GN=BCAP31 PE=1 SV=3 - [BAP31_HUMAN]	3
Flavin reductase (NADPH) OS=Homo sapiens GN=BLVRB PE=1 SV=3 - [BLVRB_HUMAN]	3
Complement component 1 Q subcomponent-binding protein, mitochondrial OS=Homo sapiens GN=C1QBP PE=1 SV=1 - [C1QBP_HUMAN]	3
Isoform 2 of Carnitine O-acetyltransferase OS=Homo sapiens GN=CRAT - [CACP_HUMAN]	3
Isoform 2 of Macrophage-capping protein OS=Homo sapiens GN=CAPG - [CAPG_HUMAN]	3
F-actin-capping protein subunit alpha-1 OS=Homo sapiens GN=CAPZA1 PE=1 SV=3 - [CAZA1_HUMAN]	3
CDGSH iron-sulfur domain-containing protein 1 OS=Homo sapiens GN=CISD1 PE=1 SV=1 - [CISD1_HUMAN]	3
Isoform Non-brain of Clathrin light chain B OS=Homo sapiens GN=CLTB - [CLCB_HUMAN]	3
Chloride intracellular channel protein 4 OS=Homo sapiens GN=CLIC4 PE=1 SV=4 - [CLIC4_HUMAN]	3
Cytochrome c oxidase subunit 5A, mitochondrial OS=Homo sapiens GN=COX5A PE=1 SV=2 - [COX5A_HUMAN]	3
Isoform 2 of DnaJ homolog subfamily A member 1 OS=Homo sapiens GN=DNAJA1 - [DNJA1_HUMAN]	3
Isoform 2 of DnaJ homolog subfamily C member 5 OS=Homo sapiens GN=DNAJC5 - [DNJC5_HUMAN]	3
Isoform 3 of Drebrin OS=Homo sapiens GN=DBN1 - [DREB_HUMAN]	3
Dynein light chain 1, cytoplasmic OS=Homo sapiens GN=DYNLL1 PE=1 SV=1 - [DYL1_HUMAN]	3
Elongation factor 1-gamma OS=Homo sapiens GN=EEF1G PE=1 SV=3 - [EF1G_HUMAN]	3
ELAV-like protein 1 OS=Homo sapiens GN=ELAVL1 PE=1 SV=2 - [ELAV1_HUMAN]	3
ELAV-like protein 3 OS=Homo sapiens GN=ELAVL3 PE=1 SV=3 - [ELAV3_HUMAN]	3
Isoform 2 of Redox-regulatory protein FAM213A OS=Homo sapiens GN=FAM213A - [F213A_HUMAN]	3
Formin-like protein 2 OS=Homo sapiens GN=FMNL2 PE=1 SV=3 - [FMNL2_HUMAN]	3
Golgi-associated plant pathogenesis-related protein 1 OS=Homo sapiens GN=GLIPR2 PE=1 SV=3 - [GAPR1_HUMAN]	3
Glutamine synthetase OS=Homo sapiens GN=GLUL PE=1 SV=4 - [GLNA_HUMAN]	3
Guanine nucleotide-binding protein-like 1 OS=Homo sapiens GN=GNL1 PE=1 SV=2 - [GNL1_HUMAN]	3
Histone H2A.Z OS=Homo sapiens GN=H2AFZ PE=1 SV=2 - [H2AZ_HUMAN]	3
Putative histone H2B type 2-D OS=Homo sapiens GN=HIST2H2BD PE=5 SV=3 - [H2B2D_HUMAN]	3
Hydroxyacyl-coenzyme A dehydrogenase, mitochondrial OS=Homo sapiens GN=HADH PE=1 SV=3 - [HCDH_HUMAN]	3
Hyaluronan and proteoglycan link protein 1 OS=Homo sapiens GN=HAPLN1 PE=2 SV=2 - [HPLN1_HUMAN]	3
Isoform 2 of Inactive hydroxysteroid dehydrogenase-like protein 1 OS=Homo sapiens GN=HSDL1 - [HSDL1_HUMAN]	3
Isoform 2 of Hydroxysteroid dehydrogenase-like protein 2 OS=Homo sapiens GN=HSDL2 - [HSDL2_HUMAN]	3
Ig gamma-1 chain C region OS=Homo sapiens GN=IGHG1 PE=1 SV=1 - [IGHG1_HUMAN]	3
Isoform 6 of Calcium/calmodulin-dependent protein kinase kinase 2 OS=Homo sapiens GN=CAMKK2 - [KKCC2_HUMAN]	3
Isoform 2 of Lactoylglutathione lyase OS=Homo sapiens GN=GLO1 - [LGUL_HUMAN]	3
Platelet-activating factor acetylhydrolase IB subunit alpha OS=Homo sapiens GN=PAFAH1B1 PE=1 SV=2 - [LIS1_HUMAN]	3
Isoform 2 of Tyrosine-protein kinase Lyn OS=Homo sapiens GN=LYN - [LYN_HUMAN]	3
Isoform 2 of Myelin-associated glycoprotein OS=Homo sapiens GN=MAG - [MAG_HUMAN]	3
NAD-dependent malic enzyme, mitochondrial OS=Homo sapiens GN=ME2 PE=1 SV=1 - [MAOM_HUMAN]	3
Microtubule-associated protein RP/EB family member 3 OS=Homo sapiens GN=MAPRE3 PE=1 SV=1 - [MARE3_HUMAN]	3
NADH-cytochrome b5 reductase 1 OS=Homo sapiens GN=CYB5R1 PE=1 SV=1 - [NB5R1_HUMAN]	3
Neutral cholesterol ester hydrolase 1 OS=Homo sapiens GN=NCEH1 PE=1 SV=3 - [NCEH1_HUMAN]	3
Isoform 2 of Neuronal calcium sensor 1 OS=Homo sapiens GN=NCS1 - [NCS1_HUMAN]	3
Isoform 4 of Protein NDRG2 OS=Homo sapiens GN=NDRG2 - [NDRG2_HUMAN]	3
NADH dehydrogenase [ubiquinone] 1 alpha subcomplex subunit 7 OS=Homo sapiens GN=NDUFA7 PE=1 SV=3 - [NDUA7_HUMAN]	3
NADH dehydrogenase [ubiquinone] iron-sulfur protein 6, mitochondrial OS=Homo sapiens GN=NDUFS6 PE=1 SV=1 - [NDUS6_HUMAN]	3
Isoform 2 of Non-POU domain-containing octamer-binding protein OS=Homo sapiens GN=NONO - [NONO_HUMAN]	3
Protein NipSnap homolog 3A OS=Homo sapiens GN=NIPSNAP3A PE=1 SV=2 - [NPS3A_HUMAN]	3
Isoform 4 of UDP-N-acetylglucosamine--peptide N-acetylglucosaminyltransferase 110 kDa subunit OS=Homo sapiens GN=OGT - [OGT1_HUMAN]	3
Isoform 3 of Pre-B-cell leukemia transcription factor-interacting protein 1 OS=Homo sapiens GN=PBXIP1 - [PBIP1_HUMAN]	3
Poly(rC)-binding protein 1 OS=Homo sapiens GN=PCBP1 PE=1 SV=2 - [PCBP1_HUMAN]	3
Isoform 2 of Propionyl-CoA carboxylase alpha chain, mitochondrial OS=Homo sapiens GN=PCCA - [PCCA_HUMAN]	3
Astrocytic phosphoprotein PEA-15 OS=Homo sapiens GN=PEA15 PE=1 SV=2 - [PEA15_HUMAN]	3
Peflin OS=Homo sapiens GN=PEF1 PE=1 SV=1 - [PEF1_HUMAN]	3
Membrane-associated progesterone receptor component 1 OS=Homo sapiens GN=PGRMC1 PE=1 SV=3 - [PGRC1_HUMAN]	3
Isoform 2 of Phytanoyl-CoA hydroxylase-interacting protein-like OS=Homo sapiens GN=PHYHIPL - [PHIPL_HUMAN]	3

Phosphomevalonate kinase OS=Homo sapiens GN=PMVK PE=1 SV=3 - [PMVK_HUMAN]	3
Isoform 2 of Serine/threonine-protein phosphatase 2A catalytic subunit alpha isoform OS=Homo sapiens GN=PPP2CA - [PP2AA_HUMAN]	3
26S protease regulatory subunit 10B OS=Homo sapiens GN=PSMC6 PE=1 SV=1 - [PRS10_HUMAN]	3
Isoform 2 of 26S protease regulatory subunit 6B OS=Homo sapiens GN=PSMC4 - [PRS6B_HUMAN]	3
Proteasome subunit beta type-1 OS=Homo sapiens GN=PSMB1 PE=1 SV=2 - [PSB1_HUMAN]	3
Prostaglandin-H2 D-isomerase OS=Homo sapiens GN=PTGDS PE=1 SV=1 - [PTGDS_HUMAN]	3
Ras-related protein Rap-2a OS=Homo sapiens GN=RAP2A PE=1 SV=1 - [RAP2A_HUMAN]	3
Ras-related protein Rap-2b OS=Homo sapiens GN=RAP2B PE=1 SV=1 - [RAP2B_HUMAN]	3
Retinol dehydrogenase 11 OS=Homo sapiens GN=RDH11 PE=1 SV=2 - [RDH11_HUMAN]	3
Rho-related GTP-binding protein RhoB OS=Homo sapiens GN=RHOB PE=1 SV=1 - [RHOB_HUMAN]	3
60S ribosomal protein L10a OS=Homo sapiens GN=RPL10A PE=1 SV=2 - [RL10A_HUMAN]	3
60S ribosomal protein L12 OS=Homo sapiens GN=RPL12 PE=1 SV=1 - [RL12_HUMAN]	3
60S ribosomal protein L13 OS=Homo sapiens GN=RPL13 PE=1 SV=4 - [RL13_HUMAN]	3
60S ribosomal protein L23a OS=Homo sapiens GN=RPL23A PE=1 SV=1 - [RL23A_HUMAN]	3
60S ribosomal protein L6 OS=Homo sapiens GN=RPL6 PE=1 SV=3 - [RL6_HUMAN]	3
tRNA-splicing ligase RtcB homolog OS=Homo sapiens GN=RTCB PE=1 SV=1 - [RTCB_HUMAN]	3
Isoform 3 of Electrogenic sodium bicarbonate cotransporter 1 OS=Homo sapiens GN=SLC4A4 - [S4A4_HUMAN]	3
Neutral amino acid transporter A OS=Homo sapiens GN=SLC1A4 PE=1 SV=1 - [SATT_HUMAN]	3
Vesicle-trafficking protein SEC22b OS=Homo sapiens GN=SEC22B PE=1 SV=4 - [SC22B_HUMAN]	3
Protein SCAI OS=Homo sapiens GN=SCAI PE=1 SV=2 - [SCAI_HUMAN]	3
Saccharopine dehydrogenase-like oxidoreductase OS=Homo sapiens GN=SCCPDH PE=1 SV=1 - [SCPDL_HUMAN]	3
Isoform 3 of Secernin-1 OS=Homo sapiens GN=SCRN1 - [SCRN1_HUMAN]	3
Isoform 2 of Shootin-1 OS=Homo sapiens GN=KIAA1598 - [SHOT1_HUMAN]	3
Sorting nexin-1 OS=Homo sapiens GN=SNX1 PE=1 SV=3 - [SNX1_HUMAN]	3
Serine/threonine-protein kinase 32C OS=Homo sapiens GN=STK32C PE=1 SV=1 - [ST32C_HUMAN]	3
Isoform 2 of 116 kDa U5 small nuclear ribonucleoprotein component OS=Homo sapiens GN=EFTUD2 - [U5S1_HUMAN]	3
General vesicular transport factor p115 OS=Homo sapiens GN=USO1 PE=1 SV=2 - [USO1_HUMAN]	3
Vesicle-associated membrane protein-associated protein A OS=Homo sapiens GN=VAPA PE=1 SV=3 - [VAPA_HUMAN]	3
WD repeat-containing protein 37 OS=Homo sapiens GN=WDR37 PE=1 SV=2 - [WDR37_HUMAN]	3
Isoform 4 of Exportin-2 OS=Homo sapiens GN=CSE1L - [XPO2_HUMAN]	3
X-ray repair cross-complementing protein 5 OS=Homo sapiens GN=XRCC5 PE=1 SV=3 - [XRCC5_HUMAN]	3
Isoform 2 of 2,4-dienoyl-CoA reductase, mitochondrial OS=Homo sapiens GN=DECR1 - [DECR_HUMAN]	3
40S ribosomal protein S3a OS=Homo sapiens GN=RPS3A PE=1 SV=1 - [D6R9B6_HUMAN]	3
Isoform 3 of Uncharacterized protein KIAA0513 OS=Homo sapiens GN=KIAA0513 - [K0513_HUMAN]	3
Isoform 2 of Mycophenolic acid acyl-glucuronide esterase, mitochondrial OS=Homo sapiens GN=ABHD10 - [ABHDA_HUMAN]	3
40S ribosomal protein S9 OS=Homo sapiens GN=RPS9 PE=1 SV=1 - [B5MCT8_HUMAN]	3
Isoform 2 of ADP-ribosylation factor-like protein 8B OS=Homo sapiens GN=ARL8B - [ARL8B_HUMAN]	3
Isoform 2 of Cytoplasmic dynein 1 light intermediate chain 2 OS=Homo sapiens GN=DYNC1LI2 - [DC1L2_HUMAN]	3
Acylglycerol kinase, mitochondrial OS=Homo sapiens GN=AGK PE=1 SV=1 - [E9PG39_HUMAN]	3
Profilin-2 OS=Homo sapiens GN=PFN2 PE=1 SV=1 - [C9J0J7_HUMAN]	3
Isoform 2 of 26S protease regulatory subunit 4 OS=Homo sapiens GN=PSMC1 - [PRS4_HUMAN]	3
Isoform 3 of Protein disulfide-isomerase A6 OS=Homo sapiens GN=PDIA6 - [PDIA6_HUMAN]	3
Isocitrate dehydrogenase [NAD] subunit, mitochondrial OS=Homo sapiens GN=IDH3G PE=3 SV=1 - [G5E9Q7_HUMAN]	3
Tropomodulin 2 (Neuronal), isoform CRA_a OS=Homo sapiens GN=TMOD2 PE=1 SV=1 - [G5EA42_HUMAN]	3
60S ribosomal protein L18 OS=Homo sapiens GN=RPL18 PE=1 SV=1 - [G3V203_HUMAN]	3
LanC-like protein 1 (Fragment) OS=Homo sapiens GN=LANCL1 PE=1 SV=1 - [E9PHS0_HUMAN]	3
Rap1 GTPase-activating protein 1 OS=Homo sapiens GN=RAP1GAP PE=1 SV=1 - [F2Z357_HUMAN]	3
5'-AMP-activated protein kinase subunit gamma-1 OS=Homo sapiens GN=PRKAG1 PE=1 SV=1 - [F8VYY9_HUMAN]	3
Pleckstrin homology domain-containing family B member 1 OS=Homo sapiens GN=PLEKHB1 PE=1 SV=1 - [F5GYU9_HUMAN]	3
Isoform 3 of 26S proteasome non-ATPase regulatory subunit 6 OS=Homo sapiens GN=PSMD6 - [PSMD6_HUMAN]	3
Single-stranded DNA-binding protein, mitochondrial (Fragment) OS=Homo sapiens GN=SSBP1 PE=1 SV=1 - [C9K0U8_HUMAN]	3
26S protease regulatory subunit 6A (Fragment) OS=Homo sapiens GN=PSMC3 PE=1 SV=2 - [E9PKD5_HUMAN]	3
Proteasome subunit alpha type OS=Homo sapiens GN=PSMA6 PE=1 SV=1 - [G3V3U4_HUMAN]	3

Endoplasmic reticulum resident protein 29 OS=Homo sapiens GN=ERP29 PE=1 SV=1 - [F8VY02_HUMAN]	3
ADP-ribosylation factor 4 (Fragment) OS=Homo sapiens GN=ARF4 PE=1 SV=1 - [C9JPM4_HUMAN]	3
Malectin (Fragment) OS=Homo sapiens GN=MLEC PE=1 SV=1 - [F5H1S8_HUMAN]	3
Isoform 3 of Stress-induced-phosphoprotein 1 OS=Homo sapiens GN=STIP1 - [STIP1_HUMAN]	3
Mitogen-activated protein kinase OS=Homo sapiens GN=MAPK3 PE=1 SV=1 - [E9PJF0_HUMAN]	3
Ribosomal protein L15 (Fragment) OS=Homo sapiens GN=RPL15 PE=1 SV=1 - [E7EQV9_HUMAN]	3
Proline synthase co-transcribed bacterial homolog protein (Fragment) OS=Homo sapiens GN=PROSC PE=1 SV=1 - [E5RFX7_HUMAN]	3
Nucleosome assembly protein 1-like 4 (Fragment) OS=Homo sapiens GN=NAP1L4 PE=1 SV=2 - [A8MXH2_HUMAN]	3
60S ribosomal protein L24 OS=Homo sapiens GN=RPL24 PE=1 SV=1 - [C9JXB8_HUMAN]	3
Clathrin coat assembly protein AP180 (Fragment) OS=Homo sapiens GN=SNAP91 PE=1 SV=1 - [E5RFC6_HUMAN]	3
Superoxide dismutase [Mn], mitochondrial (Fragment) OS=Homo sapiens GN=SOD2 PE=1 SV=1 - [F5H3C5_HUMAN]	3
Nucleosome assembly protein 1-like 1 (Fragment) OS=Homo sapiens GN=NAP1L1 PE=1 SV=1 - [H0YHC3_HUMAN]	3
Microtubule-associated protein RP/EB family member 2 (Fragment) OS=Homo sapiens GN=MAPRE2 PE=1 SV=1 - [M0QX52_HUMAN]	3
Ethanolamine-phosphate cytidylyltransferase (Fragment) OS=Homo sapiens GN=PCYT2 PE=1 SV=1 - [I3L1L9_HUMAN]	3
Protein lin-7 homolog A (Fragment) OS=Homo sapiens GN=LIN7A PE=1 SV=1 - [H0YI92_HUMAN]	3
Kinesin-like protein (Fragment) OS=Homo sapiens GN=KIF21A PE=1 SV=1 - [H0YIM7_HUMAN]	3
V-type proton ATPase subunit D OS=Homo sapiens GN=ATP6V1D PE=1 SV=1 - [G3V2S6_HUMAN]	3
Arf-GAP with GTPase, ANK repeat and PH domain-containing protein 3 (Fragment) OS=Homo sapiens GN=AGAP3 PE=1 SV=1 - [H7C4F1_HUMAN]	3
26S proteasome non-ATPase regulatory subunit 13 OS=Homo sapiens GN=PSMD13 PE=1 SV=1 - [J3KNQ3_HUMAN]	3
Aflatoxin B1 aldehyde reductase member 2 (Fragment) OS=Homo sapiens GN=AKR7A2 PE=1 SV=1 - [H3BLU7_HUMAN]	3
D-beta-hydroxybutyrate dehydrogenase, mitochondrial (Fragment) OS=Homo sapiens GN=BDH1 PE=1 SV=1 - [H7C2W1_HUMAN]	3
6-phosphogluconate dehydrogenase, decarboxylating (Fragment) OS=Homo sapiens GN=PGD PE=1 SV=1 - [K7EMN2_HUMAN]	3
Poly(rC)-binding protein 2 (Fragment) OS=Homo sapiens GN=PCBP2 PE=1 SV=1 - [H3BRU6_HUMAN]	3
Hydroxyacylglutathione hydrolase, mitochondrial (Fragment) OS=Homo sapiens GN=HAGH PE=1 SV=1 - [H3BPK3_HUMAN]	3
Heme oxygenase 2 (Fragment) OS=Homo sapiens GN=HMOX2 PE=1 SV=1 - [I3L159_HUMAN]	3
Isoform 3 of ATP-dependent RNA helicase DDX1 OS=Homo sapiens GN=DDX1 - [DDX1_HUMAN]	3
Isoform 2 of Ran-specific GTPase-activating protein OS=Homo sapiens GN=RANBP1 - [RANG_HUMAN]	3
Isoform 2 of STE20/SPS1-related proline-alanine-rich protein kinase OS=Homo sapiens GN=STK39 - [STK39_HUMAN]	3
Isoform 3 of TBC1 domain family member 17 OS=Homo sapiens GN=TBC1D17 - [TBC17_HUMAN]	3
Tubulointerstitial nephritis antigen-like OS=Homo sapiens GN=TINAGL1 PE=1 SV=1 - [F6SDV2_HUMAN]	3
Rho GDP-dissociation inhibitor 1 (Fragment) OS=Homo sapiens GN=ARHGDIA PE=1 SV=4 - [J3KTF8_HUMAN]	3
26S proteasome non-ATPase regulatory subunit 8 (Fragment) OS=Homo sapiens GN=PSMD8 PE=1 SV=4 - [K7EJR3_HUMAN]	3
Carbonic anhydrase 1 (Fragment) OS=Homo sapiens GN=CA1 PE=1 SV=4 - [E5RH81_HUMAN]	3
Opalin OS=Homo sapiens GN=OPALIN PE=1 SV=1 - [A0A0A0MTN4_HUMAN]	3
Ras-related protein Rab-14 (Fragment) OS=Homo sapiens GN=RAB14 PE=1 SV=1 - [X6RFL8_HUMAN]	3
GRIP1-associated protein 1 OS=Homo sapiens GN=GRIPAP1 PE=1 SV=1 - [A0A087WT45_HUMAN]	3
Isoform 2 of HLA class I histocompatibility antigen, Cw-16 alpha chain OS=Homo sapiens GN=HLA-C - [1C16_HUMAN]	2
Isoform 3 of Serine/threonine-protein phosphatase 2A 55 kDa regulatory subunit B gamma isoform OS=Homo sapiens GN=PPP2R2C - [2ABG_HUMAN]	2
3-hydroxyisobutyrate dehydrogenase, mitochondrial OS=Homo sapiens GN=HIBADH PE=1 SV=2 - [3HIDH_HUMAN]	2
Isoform 2 of Alpha-1-antitrypsin OS=Homo sapiens GN=SERPINA1 - [A1AT_HUMAN]	2
Isoform APP639 of Amyloid beta A4 protein OS=Homo sapiens GN=APP - [A4_HUMAN]	2
Acyl-CoA-binding protein OS=Homo sapiens GN=DBI PE=1 SV=2 - [ACBP_HUMAN]	2
Long-chain-fatty-acid--CoA ligase 3 OS=Homo sapiens GN=ACSL3 PE=1 SV=3 - [ACSL3_HUMAN]	2
L-aminoadipate-semialdehyde dehydrogenase-phosphopantetheinyl transferase OS=Homo sapiens GN=AASDHPPT PE=1 SV=2 - [ADPPT_HUMAN]	2
Aldose reductase OS=Homo sapiens GN=AKR1B1 PE=1 SV=3 - [ALDR_HUMAN]	2
Isoform 2 of Cytosol aminopeptidase OS=Homo sapiens GN=LAP3 - [AMPL_HUMAN]	2
AP-1 complex subunit gamma-1 OS=Homo sapiens GN=AP1G1 PE=1 SV=5 - [AP1G1_HUMAN]	2
Isoform 3 of AP-3 complex subunit delta-1 OS=Homo sapiens GN=AP3D1 - [AP3D1_HUMAN]	2
Actin-related protein 2/3 complex subunit 1A OS=Homo sapiens GN=ARPC1A PE=1 SV=2 - [ARC1A_HUMAN]	2
ATP synthase subunit e, mitochondrial OS=Homo sapiens GN=ATP5I PE=1 SV=2 - [ATP5I_HUMAN]	2
Isoform 4 of ATP synthase subunit f, mitochondrial OS=Homo sapiens GN=ATP5J2 - [ATPK_HUMAN]	2
Methylglutaconyl-CoA hydratase, mitochondrial OS=Homo sapiens GN=AUH PE=1 SV=1 - [AUHM_HUMAN]	2
Carbonic anhydrase 4 OS=Homo sapiens GN=CA4 PE=1 SV=2 - [CAH4_HUMAN]	2

Calpain-1 catalytic subunit OS=Homo sapiens GN=CAPN1 PE=1 SV=1 - [CAN1_HUMAN]	2
Cell cycle exit and neuronal differentiation protein 1 OS=Homo sapiens GN=CEND1 PE=2 SV=1 - [CEND_HUMAN]	2
Chitinase-3-like protein 1 OS=Homo sapiens GN=CHI3L1 PE=1 SV=2 - [CH3L1_HUMAN]	2
MICOS complex subunit MIC25 OS=Homo sapiens GN=CHCHD6 PE=1 SV=1 - [MIC25_HUMAN]	2
Cytoskeleton-associated protein 4 OS=Homo sapiens GN=CKAP4 PE=1 SV=2 - [CKAP4_HUMAN]	2
Claudin-11 OS=Homo sapiens GN=CLDN11 PE=1 SV=2 - [CLD11_HUMAN]	2
Clavesin-2 OS=Homo sapiens GN=CLVS2 PE=2 SV=1 - [CLVS2_HUMAN]	2
CB1 cannabinoid receptor-interacting protein 1 OS=Homo sapiens GN=CNRIP1 PE=1 SV=1 - [CNRP1_HUMAN]	2
Coatomer subunit gamma-1 OS=Homo sapiens GN=COPG1 PE=1 SV=1 - [COPG1_HUMAN]	2
Isoform 3 of Ubiquinone biosynthesis monooxygenase COQ6 OS=Homo sapiens GN=COQ6 - [COQ6_HUMAN]	2
Copine-3 OS=Homo sapiens GN=CPNE3 PE=1 SV=1 - [CPNE3_HUMAN]	2
Copine-4 OS=Homo sapiens GN=CPNE4 PE=2 SV=1 - [CPNE4_HUMAN]	2
Carnitine O-palmitoyltransferase 2, mitochondrial OS=Homo sapiens GN=CPT2 PE=1 SV=2 - [CPT2_HUMAN]	2
Casein kinase II subunit beta OS=Homo sapiens GN=CSNK2B PE=1 SV=1 - [CSK2B_HUMAN]	2
Caskin-1 OS=Homo sapiens GN=CASKIN1 PE=1 SV=1 - [CSKI1_HUMAN]	2
Isoform 3 of Choline transporter-like protein 1 OS=Homo sapiens GN=SLC44A1 - [CTL1_HUMAN]	2
Isoform 3 of Choline transporter-like protein 2 OS=Homo sapiens GN=SLC44A2 - [CTL2_HUMAN]	2
Cytochrome c oxidase subunit 6B1 OS=Homo sapiens GN=COX6B1 PE=1 SV=2 - [CX6B1_HUMAN]	2
Isoform 2 of Epimerase family protein SDR39U1 OS=Homo sapiens GN=SDR39U1 - [D39U1_HUMAN]	2
Isoform 3 of Dematin OS=Homo sapiens GN=DMTN - [DEMA_HUMAN]	2
Isoform 3 of Putative ATP-dependent RNA helicase DHX30 OS=Homo sapiens GN=DHX30 - [DHX30_HUMAN]	2
Dynein light chain roadblock-type 1 OS=Homo sapiens GN=DYNLRB1 PE=1 SV=3 - [DLRB1_HUMAN]	2
DnaJ homolog subfamily A member 2 OS=Homo sapiens GN=DNAJA2 PE=1 SV=1 - [DNJA2_HUMAN]	2
Isoform 4 of Inactive dipeptidyl peptidase 10 OS=Homo sapiens GN=DPP10 - [DPP10_HUMAN]	2
Dihydropyrimidinase OS=Homo sapiens GN=DPYS PE=1 SV=1 - [DPYS_HUMAN]	2
Eukaryotic translation initiation factor 3 subunit F OS=Homo sapiens GN=EIF3F PE=1 SV=1 - [EIF3F_HUMAN]	2
Isoform 3 of Epsin-1 OS=Homo sapiens GN=EPN1 - [EPN1_HUMAN]	2
Fatty acid-binding protein, brain OS=Homo sapiens GN=FABP7 PE=1 SV=3 - [FABP7_HUMAN]	2
FAS-associated factor 2 OS=Homo sapiens GN=FAF2 PE=1 SV=2 - [FAF2_HUMAN]	2
Fatty acid synthase OS=Homo sapiens GN=FASN PE=1 SV=3 - [FAS_HUMAN]	2
Mitochondrial fission 1 protein OS=Homo sapiens GN=FIS1 PE=1 SV=2 - [FIS1_HUMAN]	2
Peptidyl-prolyl cis-trans isomerase FKBP3 OS=Homo sapiens GN=FKBP3 PE=1 SV=1 - [FKBP3_HUMAN]	2
Peptidyl-prolyl cis-trans isomerase FKBP4 OS=Homo sapiens GN=FKBP4 PE=1 SV=3 - [FKBP4_HUMAN]	2
Peptidyl-prolyl cis-trans isomerase FKBP8 OS=Homo sapiens GN=FKBP8 PE=1 SV=2 - [FKBP8_HUMAN]	2
Isoform 2 of Growth arrest-specific protein 7 OS=Homo sapiens GN=GAS7 - [GAS7_HUMAN]	2
Isoform 2 of Ganglioside-induced differentiation-associated protein 1-like 1 OS=Homo sapiens GN=GDAP1L1 - [GD1L1_HUMAN]	2
Isoform 2 of Glucosidase 2 subunit beta OS=Homo sapiens GN=PRKCSH - [GLU2B_HUMAN]	2
Glyoxylate reductase/hydroxypyruvate reductase OS=Homo sapiens GN=GRHPR PE=1 SV=1 - [GRHPR_HUMAN]	2
Isoform 4 of Glutathione reductase, mitochondrial OS=Homo sapiens GN=GSR - [GSHR_HUMAN]	2
Isoform 4 of Glutathione S-transferase kappa 1 OS=Homo sapiens GN=GSTK1 - [GSTK1_HUMAN]	2
Histone H1x OS=Homo sapiens GN=H1FX PE=1 SV=1 - [H1X_HUMAN]	2
Histone H2A type 2-B OS=Homo sapiens GN=HIST2H2AB PE=1 SV=3 - [H2A2B_HUMAN]	2
Isoform 2 of 3-hydroxyacyl-CoA dehydrogenase type-2 OS=Homo sapiens GN=HSD17B10 - [HCD2_HUMAN]	2
Isoform 2 of Haloacid dehalogenase-like hydrolase domain-containing protein 2 OS=Homo sapiens GN=HDHD2 - [HDHD2_HUMAN]	2
Heme-binding protein 1 OS=Homo sapiens GN=HEBP1 PE=1 SV=1 - [HEBP1_HUMAN]	2
Hepatocyte cell adhesion molecule OS=Homo sapiens GN=HEPACAM PE=1 SV=1 - [HECAM_HUMAN]	2
Isoform Beta of Heat shock protein 105 kDa OS=Homo sapiens GN=HSPH1 - [HS105_HUMAN]	2
Hexokinase-3 OS=Homo sapiens GN=HK3 PE=1 SV=2 - [HXK3_HUMAN]	2
Eukaryotic translation initiation factor 5 OS=Homo sapiens GN=EIF5 PE=1 SV=2 - [IF5_HUMAN]	2
Ig gamma-2 chain C region OS=Homo sapiens GN=IGHG2 PE=1 SV=2 - [IGHG2_HUMAN]	2
Immunoglobulin superfamily member 21 OS=Homo sapiens GN=IGSF21 PE=2 SV=1 - [IGS21_HUMAN]	2
Isoform 2 of Inverted formin-2 OS=Homo sapiens GN=INF2 - [INF2_HUMAN]	2
Isoform 2 of Isovaleryl-CoA dehydrogenase, mitochondrial OS=Homo sapiens GN=IVD - [IVD_HUMAN]	2

BTB/POZ domain-containing protein KCTD16 OS=Homo sapiens GN=KCTD16 PE=2 SV=1 - [KCD16_HUMAN]	2
Creatine kinase M-type OS=Homo sapiens GN=CKM PE=1 SV=2 - [KCRM_HUMAN]	2
Calcium/calmodulin-dependent protein kinase kinase 1 OS=Homo sapiens GN=CAMKK1 PE=1 SV=2 - [KKCC1_HUMAN]	2
Lysosome-associated membrane glycoprotein 2 OS=Homo sapiens GN=LAMP2 PE=1 SV=2 - [LAMP2_HUMAN]	2
LanC-like protein 2 OS=Homo sapiens GN=LANCL2 PE=1 SV=1 - [LANC2_HUMAN]	2
Lupus La protein OS=Homo sapiens GN=SSB PE=1 SV=2 - [LA_HUMAN]	2
Isoform 2 of Liprin-alpha-2 OS=Homo sapiens GN=PPFIA2 - [LIPA2_HUMAN]	2
Isoform 3 of Methionine adenosyltransferase 2 subunit beta OS=Homo sapiens GN=MAT2B - [MAT2B_HUMAN]	2
Isoform 2 of Methylcrotonoyl-CoA carboxylase beta chain, mitochondrial OS=Homo sapiens GN=MCCC2 - [MCCB_HUMAN]	2
Trans-2-enoyl-CoA reductase, mitochondrial OS=Homo sapiens GN=MECR PE=1 SV=2 - [MECR_HUMAN]	2
Microsomal glutathione S-transferase 3 OS=Homo sapiens GN=MGST3 PE=1 SV=1 - [MGST3_HUMAN]	2
Isoform 6 of Mitochondrial Rho GTPase 1 OS=Homo sapiens GN=RHOT1 - [MIRO1_HUMAN]	2
Myeloid leukemia factor 2 OS=Homo sapiens GN=MLF2 PE=1 SV=1 - [MLF2_HUMAN]	2
Dual specificity mitogen-activated protein kinase kinase 4 OS=Homo sapiens GN=MAP2K4 PE=1 SV=1 - [MP2K4_HUMAN]	2
Myotubularin-related protein 5 OS=Homo sapiens GN=SBF1 PE=1 SV=3 - [MTMR5_HUMAN]	2
Isoform 3 of Metaxin-1 OS=Homo sapiens GN=MTX1 - [MTX1_HUMAN]	2
Myosin-10 OS=Homo sapiens GN=MYH10 PE=1 SV=3 - [MYH10_HUMAN]	2
NADPH--cytochrome P450 reductase OS=Homo sapiens GN=POR PE=1 SV=2 - [NCPR_HUMAN]	2
NADH dehydrogenase [ubiquinone] 1 alpha subcomplex subunit 13 OS=Homo sapiens GN=NDUFA13 PE=1 SV=3 - [NDUAD_HUMAN]	2
NADH dehydrogenase [ubiquinone] iron-sulfur protein 5 OS=Homo sapiens GN=NDUFS5 PE=1 SV=3 - [NDUS5_HUMAN]	2
Isoform 2 of Nebulette OS=Homo sapiens GN=NEBL - [NEBL_HUMAN]	2
Protein NipSnap homolog 2 OS=Homo sapiens GN=GBAS PE=1 SV=1 - [NIPS2_HUMAN]	2
Nucleolar protein 58 OS=Homo sapiens GN=NOP58 PE=1 SV=1 - [NOP58_HUMAN]	2
Protein NipSnap homolog 3B OS=Homo sapiens GN=NIPSNAP3B PE=2 SV=1 - [NPS3B_HUMAN]	2
Neuronal pentraxin-1 OS=Homo sapiens GN=NPTX1 PE=2 SV=2 - [NPTX1_HUMAN]	2
Platelet-activating factor acetylhydrolase IB subunit gamma OS=Homo sapiens GN=PAFAH1B3 PE=1 SV=1 - [PA1B3_HUMAN]	2
Protocadherin-1 OS=Homo sapiens GN=PCDH1 PE=1 SV=2 - [PCDH1_HUMAN]	2
Protein disulfide-isomerase A4 OS=Homo sapiens GN=PDIA4 PE=1 SV=2 - [PDIA4_HUMAN]	2
[Pyruvate dehydrogenase (acetyl-transferring)] kinase isozyme 3, mitochondrial OS=Homo sapiens GN=PDK3 PE=1 SV=1 - [PDK3_HUMAN]	2
Isoform 2 of Serine/threonine-protein phosphatase PGAM5, mitochondrial OS=Homo sapiens GN=PGAM5 - [PGAM5_HUMAN]	2
Prostaglandin E synthase 2 OS=Homo sapiens GN=PTGES2 PE=1 SV=1 - [PGES2_HUMAN]	2
Glucose 1,6-bisphosphate synthase OS=Homo sapiens GN=PGM2L1 PE=1 SV=3 - [PGM2L_HUMAN]	2
Pyridoxal phosphate phosphatase OS=Homo sapiens GN=PDXP PE=1 SV=2 - [PLPP_HUMAN]	2
Isoform 2 of Protein phosphatase methylesterase 1 OS=Homo sapiens GN=PPME1 - [PPME1_HUMAN]	2
PRA1 family protein 2 OS=Homo sapiens GN=PRAF2 PE=1 SV=1 - [PRAF2_HUMAN]	2
Profilin-1 OS=Homo sapiens GN=PFN1 PE=1 SV=2 - [PROF1_HUMAN]	2
26S proteasome non-ATPase regulatory subunit 11 OS=Homo sapiens GN=PSMD11 PE=1 SV=3 - [PSD11_HUMAN]	2
26S proteasome non-ATPase regulatory subunit 12 OS=Homo sapiens GN=PSMD12 PE=1 SV=3 - [PSD12_HUMAN]	2
Isoform 3 of Receptor-type tyrosine-protein phosphatase delta OS=Homo sapiens GN=PTPRD - [PTPRD_HUMAN]	2
Isoform 2 of Receptor-type tyrosine-protein phosphatase-like N OS=Homo sapiens GN=PTPRN - [PTPRN_HUMAN]	2
Ras-related protein Rab-18 OS=Homo sapiens GN=RAB18 PE=1 SV=1 - [RAB18_HUMAN]	2
GTPase NRas OS=Homo sapiens GN=NRAS PE=1 SV=1 - [RASN_HUMAN]	2
Isoform 2 of 60S ribosomal protein L11 OS=Homo sapiens GN=RPL11 - [RL11_HUMAN]	2
60S ribosomal protein L27 OS=Homo sapiens GN=RPL27 PE=1 SV=2 - [RL27_HUMAN]	2
Heterogeneous nuclear ribonucleoprotein A0 OS=Homo sapiens GN=HNRNPA0 PE=1 SV=1 - [ROA0_HUMAN]	2
Isoform 3 of Rap1 GTPase-activating protein 2 OS=Homo sapiens GN=RAP1GAP2 - [RPGP2_HUMAN]	2
Ras-related GTP-binding protein A OS=Homo sapiens GN=RRAGA PE=1 SV=1 - [RRAGA_HUMAN]	2
40S ribosomal protein S10 OS=Homo sapiens GN=RPS10 PE=1 SV=1 - [RS10_HUMAN]	2
40S ribosomal protein S14 OS=Homo sapiens GN=RPS14 PE=1 SV=3 - [RS14_HUMAN]	2
40S ribosomal protein S19 OS=Homo sapiens GN=RPS19 PE=1 SV=2 - [RS19_HUMAN]	2
40S ribosomal protein S20 OS=Homo sapiens GN=RPS20 PE=1 SV=1 - [RS20_HUMAN]	2
Isoform 2 of 40S ribosomal protein S24 OS=Homo sapiens GN=RPS24 - [RS24_HUMAN]	2
Isoform 2 of Solute carrier family 12 member 5 OS=Homo sapiens GN=SLC12A5 - [S12A5_HUMAN]	2

Prosaposin OS=Homo sapiens GN=PSAP PE=1 SV=2 - [SAP_HUMAN]	2
Sodium channel subunit beta-2 OS=Homo sapiens GN=SCN2B PE=1 SV=1 - [SCN2B_HUMAN]	2
Small glutamine-rich tetratricopeptide repeat-containing protein alpha OS=Homo sapiens GN=SGTA PE=1 SV=1 - [SGTA_HUMAN]	2
SH3 domain-binding glutamic acid-rich-like protein OS=Homo sapiens GN=SH3BGRL PE=1 SV=1 - [SH3L1_HUMAN]	2
Isoform 3 of Stromal membrane-associated protein 1 OS=Homo sapiens GN=SMAP1 - [SMAP1_HUMAN]	2
Small nuclear ribonucleoprotein Sm D2 OS=Homo sapiens GN=SNRPD2 PE=1 SV=1 - [SMD2_HUMAN]	2
Isoform 2 of Sorting nexin-27 OS=Homo sapiens GN=SNX27 - [SNX27_HUMAN]	2
Isoform 2 of Sorting nexin-3 OS=Homo sapiens GN=SNX3 - [SNX3_HUMAN]	2
Superoxide dismutase [Cu-Zn] OS=Homo sapiens GN=SOD1 PE=1 SV=2 - [SODC_HUMAN]	2
Isoform 2 of Sortilin OS=Homo sapiens GN=SORT1 - [SORT_HUMAN]	2
Isoform 4 of SRC kinase signaling inhibitor 1 OS=Homo sapiens GN=SRCIN1 - [SRCN1_HUMAN]	2
Serine-threonine kinase receptor-associated protein OS=Homo sapiens GN=STRAP PE=1 SV=1 - [STRAP_HUMAN]	2
Isoform 2 of Syntaxin-7 OS=Homo sapiens GN=STX7 - [STX7_HUMAN]	2
Syntaxin-binding protein 3 OS=Homo sapiens GN=STXBP3 PE=1 SV=2 - [STXB3_HUMAN]	2
Synapsin-3 OS=Homo sapiens GN=SYN3 PE=1 SV=2 - [SYN3_HUMAN]	2
Synaptophysin OS=Homo sapiens GN=SYP PE=1 SV=3 - [SYPH_HUMAN]	2
Synaptotagmin-7 OS=Homo sapiens GN=SYT7 PE=1 SV=3 - [SYT7_HUMAN]	2
Gamma-synuclein OS=Homo sapiens GN=SNCG PE=1 SV=2 - [SYUG_HUMAN]	2
Isoform 2 of Transcription factor A, mitochondrial OS=Homo sapiens GN=TFAM - [TFAM_HUMAN]	2
Acetyl-CoA acetyltransferase, cytosolic OS=Homo sapiens GN=ACAT2 PE=1 SV=2 - [THIC_HUMAN]	2
Transmembrane emp24 domain-containing protein 9 OS=Homo sapiens GN=TMED9 PE=1 SV=2 - [TMED9_HUMAN]	2
Transmembrane protein 35 OS=Homo sapiens GN=TMEM35 PE=2 SV=2 - [TMM35_HUMAN]	2
Transmembrane protein 65 OS=Homo sapiens GN=TMEM65 PE=1 SV=2 - [TMM65_HUMAN]	2
Protein disulfide-isomerase TMX3 OS=Homo sapiens GN=TMX3 PE=1 SV=2 - [TMX3_HUMAN]	2
Mitochondrial import receptor subunit TOM22 homolog OS=Homo sapiens GN=TOMM22 PE=1 SV=3 - [TOM22_HUMAN]	2
Isoform 6 of Tumor protein D54 OS=Homo sapiens GN=TPD52L2 - [TPD54_HUMAN]	2
Serotransferrin OS=Homo sapiens GN=TF PE=1 SV=3 - [TRFE_HUMAN]	2
Protein TSSC1 OS=Homo sapiens GN=TSSC1 PE=1 SV=2 - [TSSC1_HUMAN]	2
Isoform 4 of Protein tweety homolog 1 OS=Homo sapiens GN=TTYH1 - [TTYH1_HUMAN]	2
Cytochrome b-c1 complex subunit Rieske, mitochondrial OS=Homo sapiens GN=UQCRFS1 PE=1 SV=2 - [UCRI_HUMAN]	2
V-type proton ATPase subunit G 2 OS=Homo sapiens GN=ATP6V1G2 PE=1 SV=1 - [VATG2_HUMAN]	2
Vacuolar protein sorting-associated protein 29 OS=Homo sapiens GN=VPS29 PE=1 SV=1 - [VPS29_HUMAN]	2
Aldo-keto reductase family 1 member C2 OS=Homo sapiens GN=AKR1C2 PE=1 SV=1 - [B4DK69_HUMAN]	2
Synaptogyrin-1 OS=Homo sapiens GN=SYNGR1 PE=1 SV=1 - [B5MCD7_HUMAN]	2
Profilin-2 OS=Homo sapiens GN=PFN2 PE=1 SV=1 - [C9J712_HUMAN]	2
Transcription elongation factor B (SIII), polypeptide 2 (18kDa, elongin B), isoform CRA_b OS=Homo sapiens GN=TCEB2 PE=1 SV=1 - [B8ZZU8_HUMAN]	2
Isoform 2 of Translationally-controlled tumor protein OS=Homo sapiens GN=TPT1 - [TCTP_HUMAN]	2
Matrin-3 OS=Homo sapiens GN=MATR3 PE=1 SV=1 - [B3KM87_HUMAN]	2
Isoform 2 of ADP-ribosylation factor 3 OS=Homo sapiens GN=ARF3 - [ARF3_HUMAN]	2
COP9 signalosome complex subunit 1 OS=Homo sapiens GN=GPS1 PE=1 SV=2 - [C9JFE4_HUMAN]	2
Calcium/calmodulin-dependent 3',5'-cyclic nucleotide phosphodiesterase 1B OS=Homo sapiens GN=PDE1B PE=1 SV=1 - [B4DK72_HUMAN]	2
MAGUK p55 subfamily member 6 OS=Homo sapiens GN=MPP6 PE=1 SV=1 - [B8ZZG1_HUMAN]	2
Isoform 2 of Leucine-rich repeat LGI family member 3 OS=Homo sapiens GN=LGI3 - [LGI3_HUMAN]	2
Isoform 2 of Stomatin-like protein 2, mitochondrial OS=Homo sapiens GN=STOML2 - [STML2_HUMAN]	2
Isoform 3 of NADH dehydrogenase [ubiquinone] 1 beta subcomplex subunit 8, mitochondrial OS=Homo sapiens GN=NDUFB8 - [NDUB8_HUMAN]	2
Alpha-1-antichymotrypsin OS=Homo sapiens GN=SERPINA3 PE=1 SV=1 - [G3V3A0_HUMAN]	2
Doublecortin and CaM kinase-like 2, isoform CRA_c OS=Homo sapiens GN=DCLK2 PE=1 SV=1 - [G5E9L9_HUMAN]	2
40S ribosomal protein S17 (Fragment) OS=Homo sapiens GN=RPS17 PE=1 SV=1 - [H0YN73_HUMAN]	2
60S ribosomal protein L21 OS=Homo sapiens GN=RPL21 PE=1 SV=1 - [G3V1B3_HUMAN]	2
Vesicle-associated membrane protein 2 OS=Homo sapiens GN=VAMP2 PE=4 SV=2 - [J3QRU4_HUMAN]	2
Mitochondrial pyruvate carrier 2 (Fragment) OS=Homo sapiens GN=MPC2 PE=1 SV=1 - [Q5R3B4_HUMAN]	2
Nascent polypeptide-associated complex subunit alpha (Fragment) OS=Homo sapiens GN=NACA PE=1 SV=1 - [F8W1N5_HUMAN]	2
Ribonuclease inhibitor (Fragment) OS=Homo sapiens GN=RNH1 PE=1 SV=1 - [E9PMJ3_HUMAN]	2

60S ribosomal protein L8 (Fragment) OS=Homo sapiens GN=RPL8 PE=1 SV=1 - [E9PKZ0_HUMAN]	2
Apolipoprotein A-I OS=Homo sapiens GN=APOA1 PE=1 SV=1 - [F8W696_HUMAN]	2
Monoacylglycerol lipase ABHD12 (Fragment) OS=Homo sapiens GN=ABHD12 PE=1 SV=1 - [Q5T712_HUMAN]	2
Isoform 5 of MAGUK p55 subfamily member 2 OS=Homo sapiens GN=MPP2 - [MPP2_HUMAN]	2
Isoform 2 of Dolichyl-diphosphooligosaccharide--protein glycosyltransferase 48 kDa subunit OS=Homo sapiens GN=DDOST - [OST48_HUMAN]	2
Serine/threonine-protein phosphatase 2A activator (Fragment) OS=Homo sapiens GN=PPP2R4 PE=1 SV=1 - [A6PVN7_HUMAN]	2
Ubiquitin-conjugating enzyme E2 N OS=Homo sapiens GN=UBE2N PE=1 SV=1 - [F8VZ29_HUMAN]	2
Glutathione S-transferase Mu 1 OS=Homo sapiens GN=GSTM1 PE=1 SV=1 - [B9ZVX7_HUMAN]	2
COP9 signalosome complex subunit 7a (Fragment) OS=Homo sapiens GN=COPS7A PE=1 SV=1 - [F5H7C6_HUMAN]	2
Cytoplasmic dynein 1 light intermediate chain 1 OS=Homo sapiens GN=DYNC1LI1 PE=1 SV=1 - [E9PHI6_HUMAN]	2
N-acetyl-D-glucosamine kinase OS=Homo sapiens GN=NAGK PE=1 SV=3 - [C9JEV6_HUMAN]	2
ADP-ribosylation factor 5 (Fragment) OS=Homo sapiens GN=ARF5 PE=1 SV=1 - [C9J1Z8_HUMAN]	2
F-actin-capping protein subunit alpha-2 OS=Homo sapiens GN=CAPZA2 PE=1 SV=1 - [C9JUG7_HUMAN]	2
Tetraspanin OS=Homo sapiens GN=CD9 PE=1 SV=1 - [A6NNI4_HUMAN]	2
NADH dehydrogenase [ubiquinone] iron-sulfur protein 7, mitochondrial OS=Homo sapiens GN=NDUFS7 PE=1 SV=1 - [F5H5N1_HUMAN]	2
Voltage-dependent L-type calcium channel subunit beta-3 (Fragment) OS=Homo sapiens GN=CACNB3 PE=1 SV=1 - [F8VUW8_HUMAN]	2
Uncharacterized protein (Fragment) OS=Homo sapiens PE=1 SV=1 - [F8W031_HUMAN]	2
Wiskott-Aldrich syndrome protein family member 1 (Fragment) OS=Homo sapiens GN=WASF1 PE=1 SV=1 - [Q5SZK5_HUMAN]	2
AP-3 complex subunit mu-2 (Fragment) OS=Homo sapiens GN=AP3M2 PE=1 SV=1 - [E5RJ52_HUMAN]	2
Casein kinase I isoform epsilon OS=Homo sapiens GN=CSNK1E PE=1 SV=1 - [B0QY35_HUMAN]	2
Glutathione S-transferase omega-1 (Fragment) OS=Homo sapiens GN=GSTO1 PE=1 SV=1 - [Q5TA02_HUMAN]	2
OCIA domain-containing protein 1 (Fragment) OS=Homo sapiens GN=OCIAD1 PE=1 SV=1 - [D6R918_HUMAN]	2
Actin-related protein 2/3 complex subunit 3 (Fragment) OS=Homo sapiens GN=ARPC3 PE=1 SV=1 - [F8VR50_HUMAN]	2
PRKCA-binding protein (Fragment) OS=Homo sapiens GN=PICK1 PE=1 SV=1 - [F6V107_HUMAN]	2
60S ribosomal protein L14 OS=Homo sapiens GN=RPL14 PE=1 SV=1 - [E7EPB3_HUMAN]	2
C-Jun-amino-terminal kinase-interacting protein 3 OS=Homo sapiens GN=MAPK8IP3 PE=1 SV=1 - [E9PFH7_HUMAN]	2
Ubiquitin carboxyl-terminal hydrolase OS=Homo sapiens GN=USP14 PE=1 SV=2 - [A6NJA2_HUMAN]	2
Rho guanine nucleotide exchange factor 7 OS=Homo sapiens GN=ARHGEF7 PE=1 SV=1 - [E9PDQ5_HUMAN]	2
Transmembrane emp24 domain-containing protein 2 OS=Homo sapiens GN=TMED2 PE=1 SV=1 - [F5GX39_HUMAN]	2
Spliceosome RNA helicase DDX39B OS=Homo sapiens GN=DDX39B PE=1 SV=2 - [Q5STU3_HUMAN]	2
Obg-like ATPase 1 (Fragment) OS=Homo sapiens GN=OLA1 PE=1 SV=1 - [C9JTK6_HUMAN]	2
ADP-ribosylation factor GTPase-activating protein 1 (Fragment) OS=Homo sapiens GN=ARFGAP1 PE=1 SV=2 - [E5RHT6_HUMAN]	2
Fatty aldehyde dehydrogenase OS=Homo sapiens GN=ALDH3A2 PE=1 SV=1 - [J3QRD1_HUMAN]	2
60S ribosomal protein L22 (Fragment) OS=Homo sapiens GN=RPL22 PE=1 SV=1 - [K7EJT5_HUMAN]	2
40S ribosomal protein S16 OS=Homo sapiens GN=RPS16 PE=1 SV=1 - [M0R3H0_HUMAN]	2
Protein prune homolog 2 (Fragment) OS=Homo sapiens GN=PRUNE2 PE=1 SV=1 - [Q5JUB9_HUMAN]	2
cAMP-dependent protein kinase type II-alpha regulatory subunit (Fragment) OS=Homo sapiens GN=PRKAR2A PE=1 SV=1 - [H7C330_HUMAN]	2
26S proteasome non-ATPase regulatory subunit 7 OS=Homo sapiens GN=PSMD7 PE=1 SV=1 - [H3BNT7_HUMAN]	2
Peptidyl-prolyl cis-trans isomerase NIMA-interacting 1 (Fragment) OS=Homo sapiens GN=PIN1 PE=1 SV=1 - [K7EN45_HUMAN]	2
Kinesin-like protein (Fragment) OS=Homo sapiens GN=KIF1A PE=1 SV=1 - [H7C0K6_HUMAN]	2
60S ribosomal protein L31 (Fragment) OS=Homo sapiens GN=RPL31 PE=1 SV=1 - [H7C2W9_HUMAN]	2
SRSF protein kinase 2 (Fragment) OS=Homo sapiens GN=SRPK2 PE=1 SV=1 - [H7C5L6_HUMAN]	2
Thioredoxin-like protein 1 (Fragment) OS=Homo sapiens GN=TXNL1 PE=1 SV=1 - [K7ER96_HUMAN]	2
ERC protein 2 (Fragment) OS=Homo sapiens GN=ERC2 PE=1 SV=1 - [H7C4G9_HUMAN]	2
Serum paraoxonase/arylesterase 2 OS=Homo sapiens GN=PON2 PE=1 SV=1 - [J3QT77_HUMAN]	2
WW domain-binding protein 2 (Fragment) OS=Homo sapiens GN=WBP2 PE=1 SV=1 - [K7EIJ0_HUMAN]	2
Haptoglobin (Fragment) OS=Homo sapiens GN=HP PE=1 SV=1 - [H3BS21_HUMAN]	2
Gamma-aminobutyric acid receptor-associated protein-like 2 OS=Homo sapiens GN=GABARAPL2 PE=1 SV=1 - [H3BSM5_HUMAN]	2
Dehydrogenase/reductase SDR family member 7 (Fragment) OS=Homo sapiens GN=DHRS7 PE=1 SV=1 - [H0YJE4_HUMAN]	2
5'-nucleotidase domain-containing protein 3 (Fragment) OS=Homo sapiens GN=NT5DC3 PE=1 SV=1 - [H7C3S8_HUMAN]	2
Ras-related protein Rab-31 OS=Homo sapiens GN=RAB31 PE=1 SV=2 - [J3QR51_HUMAN]	2
40S ribosomal protein S11 OS=Homo sapiens GN=RPS11 PE=1 SV=1 - [M0QZC5_HUMAN]	2
Neuronal membrane glycoprotein M6-b (Fragment) OS=Homo sapiens GN=GPM6B PE=1 SV=1 - [H7C5H4_HUMAN]	2

IST1 homolog (Fragment) OS=Homo sapiens GN=IST1 PE=1 SV=1 - [H3BMU1_HUMAN]	2
Inosine-5'-monophosphate dehydrogenase 2 (Fragment) OS=Homo sapiens GN=IMPDH2 PE=1 SV=1 - [H0Y4R1_HUMAN]	2
Kinesin-like protein OS=Homo sapiens GN=KIF5A PE=1 SV=1 - [J3KNA1_HUMAN]	2
Fibronectin type III and SPRY domain-containing protein 1 OS=Homo sapiens GN=FSD1 PE=1 SV=1 - [M0R366_HUMAN]	2
Ubiquitin-conjugating enzyme E2 variant 1 OS=Homo sapiens GN=UBE2V1 PE=4 SV=2 - [G3V2F7_HUMAN]	2
WD repeat-containing protein 7 OS=Homo sapiens GN=WDR7 PE=1 SV=1 - [A2RRE0_HUMAN]	2
SYNGR3 protein OS=Homo sapiens GN=SYNGR3 PE=1 SV=1 - [Q96L30_HUMAN]	2
Ribose-phosphate pyrophosphokinase 1 OS=Homo sapiens GN=PRPS1 PE=1 SV=1 - [B7ZB02_HUMAN]	2
Bifunctional coenzyme A synthase (Fragment) OS=Homo sapiens GN=COASY PE=1 SV=4 - [K7EP09_HUMAN]	2
RNA-binding protein Raly (Fragment) OS=Homo sapiens GN=RALY PE=1 SV=4 - [Q5QPM0_HUMAN]	2
60S ribosomal protein L17 (Fragment) OS=Homo sapiens GN=RPL17 PE=1 SV=2 - [A0A087WY81_HUMAN]	2
ADP-ribosylation factor-like protein 8A OS=Homo sapiens GN=ARL8A PE=1 SV=1 - [A0A087X2J2_HUMAN]	2
Actin-binding protein anillin (Fragment) OS=Homo sapiens GN=ANLN PE=1 SV=4 - [H7C1K5_HUMAN]	2
Uncharacterized protein (Fragment) OS=Homo sapiens PE=1 SV=3 - [I3L4J1_HUMAN]	2
Amino acid transporter OS=Homo sapiens GN=SLC1A3 PE=1 SV=1 - [A0A087X0U3_HUMAN]	2
60S ribosomal protein L9 (Fragment) OS=Homo sapiens GN=RPL9 PE=1 SV=4 - [D6RAN4_HUMAN]	2
Kynurenine--oxoglutarate transaminase 3 (Fragment) OS=Homo sapiens GN=CCBL2 PE=1 SV=1 - [A0A0A0MRN7_HUMAN]	2
Ig lambda-2 chain C regions (Fragment) OS=Homo sapiens GN=IGLC2 PE=4 SV=1 - [A0A075B6K9_HUMAN]	2
Neuronal growth regulator 1 OS=Homo sapiens GN=NEGR1 PE=1 SV=1 - [F6X2W2_HUMAN]	2
Polyadenylate-binding protein OS=Homo sapiens GN=PABPC1 PE=1 SV=1 - [A0A087WTT1_HUMAN]	2
Lethal(2) giant larvae protein homolog 1 OS=Homo sapiens GN=LLGL1 PE=1 SV=1 - [A0A087WW77_HUMAN]	2
Mitochondrial amidoxime reducing component 2 (Fragment) OS=Homo sapiens GN=MARC2 PE=1 SV=1 - [X1WI34_HUMAN]	2
Protein FAM49B OS=Homo sapiens GN=FAM49B PE=1 SV=1 - [A0A087X178_HUMAN]	2
ATPase, H+ transporting, lysosomal accessory protein 1, isoform CRA_c OS=Homo sapiens GN=ATP6AP1 PE=4 SV=1 - [A0A0C4DGX8_HUMAN]	2
IQ motif and SEC7 domain-containing protein 1 OS=Homo sapiens GN=IQSEC1 PE=4 SV=1 - [A0A0C4DGT3_HUMAN]	2
3-ketoacyl-CoA thiolase, mitochondrial OS=Homo sapiens GN=ACAA2 PE=1 SV=1 - [A0A0B4J2A4_HUMAN]	2

Description AD2 P7	Peptides
Isoform 7 of Plectin OS=Homo sapiens GN=PLEC - [PLEC_HUMAN]	70
Cytoplasmic dynein 1 heavy chain 1 OS=Homo sapiens GN=DYNC1H1 PE=1 SV=5 - [DYHC1_HUMAN]	65
Spectrin alpha chain, non-erythrocytic 1 OS=Homo sapiens GN=SPTAN1 PE=1 SV=3 - [SPTN1_HUMAN]	42
Vesicle-fusing ATPase OS=Homo sapiens GN=NSF PE=1 SV=1 - [I3L0N3_HUMAN]	36
Isoform 3 of Dynamin-1 OS=Homo sapiens GN=DNM1 - [DYN1_HUMAN]	35
Isoform 4 of Dynamin-1 OS=Homo sapiens GN=DNM1 - [DYN1_HUMAN]	35
Spectrin beta chain, non-erythrocytic 1 OS=Homo sapiens GN=SPTBN1 PE=1 SV=2 - [SPTB2_HUMAN]	32
Glial fibrillary acidic protein OS=Homo sapiens GN=GFAP PE=1 SV=1 - [GFAP_HUMAN]	30
Vimentin OS=Homo sapiens GN=VIM PE=1 SV=4 - [VIME_HUMAN]	26
Neurofilament medium polypeptide OS=Homo sapiens GN=NEFM PE=1 SV=1 - [E7EMV2_HUMAN]	26
Alpha-internexin OS=Homo sapiens GN=INA PE=1 SV=2 - [AINX_HUMAN]	25
Keratin, type II cytoskeletal 2 epidermal OS=Homo sapiens GN=KRT2 PE=1 SV=2 - [K22E_HUMAN]	25
Heat shock 70 kDa protein 12A OS=Homo sapiens GN=HSPA12A PE=1 SV=2 - [HS12A_HUMAN]	23
Isoform 4 of Hexokinase-1 OS=Homo sapiens GN=HK1 - [HXK1_HUMAN]	23
Neurofilament light polypeptide OS=Homo sapiens GN=NEFL PE=1 SV=3 - [NFL_HUMAN]	23
Syntaxin-binding protein 1 OS=Homo sapiens GN=STXBP1 PE=1 SV=1 - [STXB1_HUMAN]	22
Isoform 2 of Syntaxin-binding protein 1 OS=Homo sapiens GN=STXBP1 - [STXB1_HUMAN]	22
Isoform CNPI of 2',3'-cyclic-nucleotide 3'-phosphodiesterase OS=Homo sapiens GN=CNP - [CN37_HUMAN]	21
Keratin, type II cytoskeletal 1 OS=Homo sapiens GN=KRT1 PE=1 SV=6 - [K2C1_HUMAN]	21
Isoform B of AP-2 complex subunit alpha-1 OS=Homo sapiens GN=AP2A1 - [AP2A1_HUMAN]	20
ATP synthase subunit alpha, mitochondrial OS=Homo sapiens GN=ATP5A1 PE=1 SV=1 - [ATPA_HUMAN]	20
Calcium-binding mitochondrial carrier protein Aralar1 OS=Homo sapiens GN=SLC25A12 PE=1 SV=2 - [CMC1_HUMAN]	20
Isoform 2 of Dynamin-3 OS=Homo sapiens GN=DNM3 - [DYN3_HUMAN]	20
ATP-dependent 6-phosphofructokinase, platelet type OS=Homo sapiens GN=PFKP PE=1 SV=2 - [PFKAP_HUMAN]	20
MICOS complex subunit MIC60 OS=Homo sapiens GN=IMMT PE=1 SV=2 - [B9A067_HUMAN]	20
C-1-tetrahydrofolate synthase, cytoplasmic OS=Homo sapiens GN=MTHFD1 PE=1 SV=3 - [C1TC_HUMAN]	19

Isoform 2 of Contactin-1 OS=Homo sapiens GN=CNTN1 - [CNTN1_HUMAN]	19
Isoform C of Prelamin-A/C OS=Homo sapiens GN=LMNA - [LMNA_HUMAN]	19
Tubulin beta-2A chain OS=Homo sapiens GN=TUBB2A PE=1 SV=1 - [TBB2A_HUMAN]	19
Tubulin beta-2B chain OS=Homo sapiens GN=TUBB2B PE=1 SV=1 - [TBB2B_HUMAN]	19
Tubulin beta-3 chain OS=Homo sapiens GN=TUBB3 PE=1 SV=2 - [TBB3_HUMAN]	19
Tubulin beta-4A chain OS=Homo sapiens GN=TUBB4A PE=1 SV=2 - [TBB4A_HUMAN]	19
Tubulin beta chain OS=Homo sapiens GN=TUBB PE=1 SV=2 - [TBB5_HUMAN]	19
Ankyrin-2 OS=Homo sapiens GN=ANK2 PE=1 SV=4 - [ANK2_HUMAN]	18
Isocitrate dehydrogenase [NADP], mitochondrial OS=Homo sapiens GN=IDH2 PE=1 SV=2 - [IDHP_HUMAN]	18
Band 4.1-like protein 3 OS=Homo sapiens GN=EPB41L3 PE=1 SV=1 - [A0A0A0MRA8_HUMAN]	18
AP-2 complex subunit alpha-2 OS=Homo sapiens GN=AP2A2 PE=1 SV=2 - [AP2A2_HUMAN]	17
Isoform 2 of Heterogeneous nuclear ribonucleoprotein M OS=Homo sapiens GN=HNRNPM - [HNRPM_HUMAN]	17
Isoform 4 of NADH-ubiquinone oxidoreductase 75 kDa subunit, mitochondrial OS=Homo sapiens GN=NDUFS1 - [NDUS1_HUMAN]	17
Sodium/potassium-transporting ATPase subunit alpha-3 OS=Homo sapiens GN=ATP1A3 PE=1 SV=1 - [M0R116_HUMAN]	17
Amine oxidase [flavin-containing] B OS=Homo sapiens GN=MAOB PE=1 SV=3 - [AOFB_HUMAN]	16
Elongation factor Tu, mitochondrial OS=Homo sapiens GN=TUFM PE=1 SV=2 - [EFTU_HUMAN]	16
Keratin, type I cytoskeletal 10 OS=Homo sapiens GN=KRT10 PE=1 SV=6 - [K1C10_HUMAN]	16
Lamin-B2 OS=Homo sapiens GN=LMNB2 PE=1 SV=3 - [LMNB2_HUMAN]	16
Microtubule-associated protein 1B OS=Homo sapiens GN=MAP1B PE=1 SV=2 - [MAP1B_HUMAN]	16
Isoform 2 of Neurofilament heavy polypeptide OS=Homo sapiens GN=NEFH - [NFH_HUMAN]	16
Alanine--tRNA ligase, cytoplasmic OS=Homo sapiens GN=AARS PE=1 SV=2 - [SYAC_HUMAN]	16
Isoform 2 of Tubulin alpha-1A chain OS=Homo sapiens GN=TUBA1A - [TBA1A_HUMAN]	16
Isoform 2 of Tubulin alpha-4A chain OS=Homo sapiens GN=TUBA4A - [TBA4A_HUMAN]	16
Isoform 2 of Ubiquitin-like modifier-activating enzyme 1 OS=Homo sapiens GN=UBA1 - [UBA1_HUMAN]	16
Isoform 3 of Sodium/potassium-transporting ATPase subunit alpha-1 OS=Homo sapiens GN=ATP1A1 - [AT1A1_HUMAN]	15
Guanine nucleotide-binding protein G(o) subunit alpha OS=Homo sapiens GN=GNAO1 PE=1 SV=4 - [GNAO_HUMAN]	15
Glycogen phosphorylase, brain form OS=Homo sapiens GN=PYGB PE=1 SV=5 - [PYGB_HUMAN]	15
Isoform 2 of Methylmalonate-semialdehyde dehydrogenase [acylating], mitochondrial OS=Homo sapiens GN=ALDH6A1 - [MMSA_HUMAN]	15
Glutaminase kidney isoform, mitochondrial OS=Homo sapiens GN=GLS PE=1 SV=1 - [GLSK_HUMAN]	14
Microtubule-associated protein 1A OS=Homo sapiens GN=MAP1A PE=1 SV=6 - [MAP1A_HUMAN]	14
Heterogeneous nuclear ribonucleoproteins A2/B1 OS=Homo sapiens GN=HNRNPA2B1 PE=1 SV=2 - [ROA2_HUMAN]	14
Catenin beta-1 OS=Homo sapiens GN=CTNNB1 PE=1 SV=1 - [B4DGU4_HUMAN]	14
Synaptotagmin I, isoform CRA_b OS=Homo sapiens GN=SYT1 PE=1 SV=1 - [J3KQA0_HUMAN]	14
Heat shock cognate 71 kDa protein OS=Homo sapiens GN=HSPA8 PE=1 SV=1 - [E9PKE3_HUMAN]	14
Unconventional myosin-Va OS=Homo sapiens GN=MYO5A PE=1 SV=1 - [A0A087WY00_HUMAN]	14
Isoform 2 of Annexin A6 OS=Homo sapiens GN=ANXA6 - [ANXA6_HUMAN]	13
ATP synthase subunit beta, mitochondrial OS=Homo sapiens GN=ATP5B PE=1 SV=3 - [ATPB_HUMAN]	13
Fibrinogen beta chain OS=Homo sapiens GN=FGB PE=1 SV=2 - [FIBB_HUMAN]	13
Keratin, type I cytoskeletal 9 OS=Homo sapiens GN=KRT9 PE=1 SV=3 - [K1C9_HUMAN]	13
Calcium/calmodulin-dependent protein kinase type II subunit alpha OS=Homo sapiens GN=CAMK2A PE=1 SV=2 - [KCC2A_HUMAN]	13
2-oxoglutarate dehydrogenase-like, mitochondrial OS=Homo sapiens GN=OGDHL PE=1 SV=3 - [OGDHL_HUMAN]	13
Protein RUFY3 OS=Homo sapiens GN=RUFY3 PE=1 SV=1 - [RUFY3_HUMAN]	13
Tubulin alpha-8 chain OS=Homo sapiens GN=TUBA8 PE=1 SV=1 - [TBA8_HUMAN]	13
Tubulin beta-6 chain OS=Homo sapiens GN=TUBB6 PE=1 SV=1 - [TBB6_HUMAN]	13
T-complex protein 1 subunit beta OS=Homo sapiens GN=CCT2 PE=1 SV=4 - [TCPB_HUMAN]	13
Isoform Short of Ubiquitin carboxyl-terminal hydrolase 5 OS=Homo sapiens GN=USP5 - [UBP5_HUMAN]	13
Exportin-1 OS=Homo sapiens GN=XPO1 PE=1 SV=1 - [XPO1_HUMAN]	13
T-complex protein 1 subunit gamma OS=Homo sapiens GN=CCT3 PE=1 SV=1 - [B4DUR8_HUMAN]	13
Serine/threonine-protein phosphatase 2A 65 kDa regulatory subunit A alpha isoform OS=Homo sapiens GN=PPP2R1A PE=1 SV=4 - [2AAA_HUMAN]	12
Isoform 2 of Catenin alpha-2 OS=Homo sapiens GN=CTNNA2 - [CTNA2_HUMAN]	12
Isoform 3 of Dynamin-1-like protein OS=Homo sapiens GN=DNM1L - [DNM1L_HUMAN]	12
Elongation factor 2 OS=Homo sapiens GN=EEF2 PE=1 SV=4 - [EF2_HUMAN]	12
Keratin, type I cytoskeletal 14 OS=Homo sapiens GN=KRT14 PE=1 SV=4 - [K1C14_HUMAN]	12

Keratin, type II cytoskeletal 5 OS=Homo sapiens GN=KRT5 PE=1 SV=3 - [K2C5_HUMAN]	12
Leucine-rich PPR motif-containing protein, mitochondrial OS=Homo sapiens GN=LRPPRC PE=1 SV=3 - [LPPRC_HUMAN]	12
Cytochrome b-c1 complex subunit 2, mitochondrial OS=Homo sapiens GN=UQCRC2 PE=1 SV=3 - [QCR2_HUMAN]	12
Isoform 2 of Protein kinase C gamma type OS=Homo sapiens GN=PRKCG - [KPCG_HUMAN]	12
Isoform 2 of V-type proton ATPase catalytic subunit A OS=Homo sapiens GN=ATP6V1A - [VATA_HUMAN]	12
Sodium/potassium-transporting ATPase subunit alpha-2 OS=Homo sapiens GN=ATP1A2 PE=1 SV=1 - [B1AKY9_HUMAN]	12
14-3-3 protein eta OS=Homo sapiens GN=YWHAH PE=1 SV=4 - [1433F_HUMAN]	11
Actin, cytoplasmic 1 OS=Homo sapiens GN=ACTB PE=1 SV=1 - [ACTB_HUMAN]	11
Actin, alpha cardiac muscle 1 OS=Homo sapiens GN=ACTC1 PE=1 SV=1 - [ACTC_HUMAN]	11
Isoform 2 of Atlastin-1 OS=Homo sapiens GN=ATL1 - [ATLA1_HUMAN]	11
Fatty acid synthase OS=Homo sapiens GN=FASN PE=1 SV=3 - [FAS_HUMAN]	11
Isoform 2 of Gelsolin OS=Homo sapiens GN=GSN - [GELS_HUMAN]	11
Guanine nucleotide-binding protein G(i) subunit alpha-2 OS=Homo sapiens GN=GNAI2 PE=1 SV=3 - [GNAI2_HUMAN]	11
Guanine nucleotide-binding protein G(z) subunit alpha OS=Homo sapiens GN=GNAZ PE=2 SV=3 - [GNAZ_HUMAN]	11
Heat shock-related 70 kDa protein 2 OS=Homo sapiens GN=HSPA2 PE=1 SV=1 - [HSP72_HUMAN]	11
Keratin, type II cytoskeletal 6A OS=Homo sapiens GN=KRT6A PE=1 SV=3 - [K2C6A_HUMAN]	11
Creatine kinase B-type OS=Homo sapiens GN=CKB PE=1 SV=1 - [KCRB_HUMAN]	11
Poly [ADP-ribose] polymerase 1 OS=Homo sapiens GN=PARP1 PE=1 SV=4 - [PARP1_HUMAN]	11
Isoform 2 of Heterogeneous nuclear ribonucleoprotein A1 OS=Homo sapiens GN=HNRNPA1 - [ROA1_HUMAN]	11
T-complex protein 1 subunit delta OS=Homo sapiens GN=CCT4 PE=1 SV=4 - [TCPD_HUMAN]	11
Prohibitin-2 OS=Homo sapiens GN=PHB2 PE=1 SV=1 - [F5GY37_HUMAN]	11
ADP/ATP translocase 1 OS=Homo sapiens GN=SLC25A4 PE=1 SV=4 - [ADT1_HUMAN]	10
Isoform 3 of Ankyrin-3 OS=Homo sapiens GN=ANK3 - [ANK3_HUMAN]	10
Isoform 3 of Brain-specific angiogenesis inhibitor 1-associated protein 2 OS=Homo sapiens GN=BAIAP2 - [BAIP2_HUMAN]	10
Carbonyl reductase [NADPH] 1 OS=Homo sapiens GN=CBR1 PE=1 SV=3 - [CBR1_HUMAN]	10
60 kDa heat shock protein, mitochondrial OS=Homo sapiens GN=HSPD1 PE=1 SV=2 - [CH60_HUMAN]	10
Contactin-associated protein 1 OS=Homo sapiens GN=CNTNAP1 PE=1 SV=1 - [CNTP1_HUMAN]	10
Copine-6 OS=Homo sapiens GN=CPNE6 PE=1 SV=3 - [CPNE6_HUMAN]	10
Isoform 2 of ATP-dependent RNA helicase DDX3X OS=Homo sapiens GN=DDX3X - [DDX3X_HUMAN]	10
Isoform 2 of Dihydropyrimidinase-related protein 2 OS=Homo sapiens GN=DPYSL2 - [DPYL2_HUMAN]	10
Elongation factor 1-alpha 1 OS=Homo sapiens GN=EEF1A1 PE=1 SV=1 - [EF1A1_HUMAN]	10
Glucose-6-phosphate isomerase OS=Homo sapiens GN=GPI PE=1 SV=4 - [G6PI_HUMAN]	10
Heat shock protein beta-1 OS=Homo sapiens GN=HSPB1 PE=1 SV=2 - [HSPB1_HUMAN]	10
Isoform 3 of Calcium/calmodulin-dependent protein kinase type II subunit beta OS=Homo sapiens GN=CAMK2B - [KCC2B_HUMAN]	10
NADH dehydrogenase [ubiquinone] 1 alpha subcomplex subunit 9, mitochondrial OS=Homo sapiens GN=NDUFA9 PE=1 SV=2 - [NDUA9_HUMAN]	10
NADH dehydrogenase [ubiquinone] iron-sulfur protein 3, mitochondrial OS=Homo sapiens GN=NDUFS3 PE=1 SV=1 - [NDUS3_HUMAN]	10
Isoform 2 of 2-oxoglutarate dehydrogenase, mitochondrial OS=Homo sapiens GN=OGDH - [ODO1_HUMAN]	10
Protein disulfide-isomerase A3 OS=Homo sapiens GN=PDIA3 PE=1 SV=4 - [PDIA3_HUMAN]	10
Isoform 2 of 26S protease regulatory subunit 8 OS=Homo sapiens GN=PSMC5 - [PRS8_HUMAN]	10
Pyruvate carboxylase, mitochondrial OS=Homo sapiens GN=PC PE=1 SV=2 - [PYC_HUMAN]	10
Beta-soluble NSF attachment protein OS=Homo sapiens GN=NAPB PE=1 SV=2 - [SNAB_HUMAN]	10
Isoform 2 of Succinyl-CoA ligase [ADP-forming] subunit beta, mitochondrial OS=Homo sapiens GN=SUCLA2 - [SUCB1_HUMAN]	10
Phenylalanine--tRNA ligase beta subunit OS=Homo sapiens GN=FARSB PE=1 SV=3 - [SYFB_HUMAN]	10
Isoform IB of Synapsin-1 OS=Homo sapiens GN=SYN1 - [SYN1_HUMAN]	10
T-complex protein 1 subunit theta OS=Homo sapiens GN=CCT8 PE=1 SV=4 - [TCPQ_HUMAN]	10
Talin-2 OS=Homo sapiens GN=TLN2 PE=1 SV=4 - [TLN2_HUMAN]	10
V-type proton ATPase subunit B, brain isoform OS=Homo sapiens GN=ATP6V1B2 PE=1 SV=3 - [VATB2_HUMAN]	10
Visinin-like protein 1 OS=Homo sapiens GN=VSNL1 PE=1 SV=2 - [VISL1_HUMAN]	10
Ras/Rap GTPase-activating protein SynGAP (Fragment) OS=Homo sapiens GN=SYNGAP1 PE=1 SV=2 - [B7ZCA0_HUMAN]	10
NADH dehydrogenase [ubiquinone] 1 alpha subcomplex subunit 10, mitochondrial OS=Homo sapiens GN=NDUFA10 PE=1 SV=1 - [A0A087WXC5_HUMAN]	10
14-3-3 protein gamma OS=Homo sapiens GN=YWHAG PE=1 SV=2 - [1433G_HUMAN]	9
14-3-3 protein zeta/delta OS=Homo sapiens GN=YWHAZ PE=1 SV=1 - [1433Z_HUMAN]	9

Isoform 2 of Alpha-adducin OS=Homo sapiens GN=ADD1 - [ADDA_HUMAN]	9
ADP/ATP translocase 2 OS=Homo sapiens GN=SLC25A5 PE=1 SV=7 - [ADT2_HUMAN]	9
ADP/ATP translocase 3 OS=Homo sapiens GN=SLC25A6 PE=1 SV=4 - [ADT3_HUMAN]	9
Isoform 2 of Clathrin heavy chain 1 OS=Homo sapiens GN=CLTC - [CLH1_HUMAN]	9
Isoform 1 of Serine/threonine-protein kinase DCLK1 OS=Homo sapiens GN=DCLK1 - [DCLK1_HUMAN]	9
Very-long-chain 3-oxoacyl-CoA reductase OS=Homo sapiens GN=HSD17B12 PE=1 SV=2 - [DHB12_HUMAN]	9
EH domain-containing protein 3 OS=Homo sapiens GN=EHD3 PE=1 SV=2 - [EHD3_HUMAN]	9
Isoform 3 of Guanine nucleotide-binding protein G(s) subunit alpha isoforms short OS=Homo sapiens GN=GNAS - [GNAS2_HUMAN]	9
Glutathione S-transferase Mu 3 OS=Homo sapiens GN=GSTM3 PE=1 SV=3 - [GSTM3_HUMAN]	9
Isoform 1 of Core histone macro-H2A.1 OS=Homo sapiens GN=H2AFY - [H2AY_HUMAN]	9
Heat shock protein HSP 90-alpha OS=Homo sapiens GN=HSP90AA1 PE=1 SV=5 - [HS90A_HUMAN]	9
ATP-dependent 6-phosphofructokinase, liver type OS=Homo sapiens GN=PFKL PE=1 SV=6 - [PFKAL_HUMAN]	9
Adenylate kinase 4, mitochondrial OS=Homo sapiens GN=AK4 PE=1 SV=1 - [KAD4_HUMAN]	9
Isoform 10 of Calcium/calmodulin-dependent protein kinase type II subunit gamma OS=Homo sapiens GN=CAMK2G - [KCC2G_HUMAN]	9
Isoform 3 of Microtubule-associated protein 2 OS=Homo sapiens GN=MAP2 - [MTAP2_HUMAN]	9
Nicotinamide phosphoribosyltransferase OS=Homo sapiens GN=NAMPT PE=1 SV=1 - [NAMPT_HUMAN]	9
Isoform 4 of Neural cell adhesion molecule 1 OS=Homo sapiens GN=NCAM1 - [NCAM1_HUMAN]	9
Isoform 2 of NADH dehydrogenase [ubiquinone] flavoprotein 1, mitochondrial OS=Homo sapiens GN=NDUFV1 - [NDUV1_HUMAN]	9
Isoform 2 of Nidogen-1 OS=Homo sapiens GN=NID1 - [NID1_HUMAN]	9
Brevican core protein OS=Homo sapiens GN=BCAN PE=1 SV=2 - [PGCB_HUMAN]	9
Isoform 4 of Phosphatidylinositol 4-phosphate 5-kinase type-1 gamma OS=Homo sapiens GN=PIP5K1C - [PI51C_HUMAN]	9
D-3-phosphoglycerate dehydrogenase OS=Homo sapiens GN=PHGDH PE=1 SV=4 - [SERA_HUMAN]	9
Alpha-soluble NSF attachment protein OS=Homo sapiens GN=NAPA PE=1 SV=3 - [SNAA_HUMAN]	9
Isoform 3 of Tubulointerstitial nephritis antigen-like OS=Homo sapiens GN=TINAGL1 - [TINAL_HUMAN]	9
Isoform 3 of Exportin-2 OS=Homo sapiens GN=CSE1L - [XPO2_HUMAN]	9
Acylglycerol kinase, mitochondrial OS=Homo sapiens GN=AGK PE=1 SV=1 - [E9PG39_HUMAN]	9
Rap1 GTPase-activating protein 1 OS=Homo sapiens GN=RAP1GAP PE=1 SV=1 - [F2Z357_HUMAN]	9
Adenylyl cyclase-associated protein OS=Homo sapiens GN=CAP2 PE=1 SV=1 - [E9PDI2_HUMAN]	9
ATP synthase F(0) complex subunit B1, mitochondrial OS=Homo sapiens GN=ATP5F1 PE=1 SV=1 - [Q5QNZ2_HUMAN]	9
Ubiquitin thioesterase OTUB1 OS=Homo sapiens GN=OTUB1 PE=1 SV=1 - [F5GYN4_HUMAN]	9
Voltage-dependent anion-selective channel protein 2 (Fragment) OS=Homo sapiens GN=VDAC2 PE=1 SV=1 - [A0A0A0MR02_HUMAN]	9
ATP-dependent RNA helicase DDX1 OS=Homo sapiens GN=DDX1 PE=1 SV=1 - [A0A087X2G1_HUMAN]	9
Isoform 2 of Long-chain-fatty-acid--CoA ligase 6 OS=Homo sapiens GN=ACSL6 - [ACSL6_HUMAN]	8
Isoform 4 of Putative tyrosine-protein phosphatase auxilin OS=Homo sapiens GN=DNAJC6 - [AUXI_HUMAN]	8
Calcium-binding mitochondrial carrier protein Aralar2 OS=Homo sapiens GN=SLC25A13 PE=1 SV=2 - [CMC2_HUMAN]	8
Isoform 3 of Disks large homolog 4 OS=Homo sapiens GN=DLG4 - [DLG4_HUMAN]	8
Guanine nucleotide-binding protein G(I)/G(S)/G(T) subunit beta-2 OS=Homo sapiens GN=GNB2 PE=1 SV=3 - [GBB2_HUMAN]	8
Stress-70 protein, mitochondrial OS=Homo sapiens GN=HSPA9 PE=1 SV=2 - [GRP75_HUMAN]	8
Histone H4 OS=Homo sapiens GN=HIST1H4A PE=1 SV=2 - [H4_HUMAN]	8
Isoform 3 of Heterogeneous nuclear ribonucleoprotein K OS=Homo sapiens GN=HNRNPK - [HNRPK_HUMAN]	8
Heat shock protein HSP 90-beta OS=Homo sapiens GN=HSP90AB1 PE=1 SV=4 - [HS90B_HUMAN]	8
Keratin, type I cytoskeletal 16 OS=Homo sapiens GN=KRT16 PE=1 SV=4 - [K1C16_HUMAN]	8
Isoform Delta 12 of Calcium/calmodulin-dependent protein kinase type II subunit delta OS=Homo sapiens GN=CAMK2D - [KCC2D_HUMAN]	8
Isoform 2 of Kinesin-like protein KIF2A OS=Homo sapiens GN=KIF2A - [KIF2A_HUMAN]	8
Kinesin heavy chain isoform 5C OS=Homo sapiens GN=KIF5C PE=1 SV=1 - [KIF5C_HUMAN]	8
NADH-cytochrome b5 reductase 1 OS=Homo sapiens GN=CYB5R1 PE=1 SV=1 - [NB5R1_HUMAN]	8
Isoform 2 of NADH dehydrogenase [ubiquinone] iron-sulfur protein 2, mitochondrial OS=Homo sapiens GN=NDUFS2 - [NDUS2_HUMAN]	8
Isoform 6 of Poly(rC)-binding protein 2 OS=Homo sapiens GN=PCBP2 - [PCBP2_HUMAN]	8
Pyridoxal kinase OS=Homo sapiens GN=PDXK PE=1 SV=1 - [PDXK_HUMAN]	8
Phosphoglycerate mutase 1 OS=Homo sapiens GN=PGAM1 PE=1 SV=2 - [PGAM1_HUMAN]	8
Prohibitin OS=Homo sapiens GN=PHB PE=1 SV=1 - [PHB_HUMAN]	8
Phytanoyl-CoA hydroxylase-interacting protein OS=Homo sapiens GN=PHYHIP PE=1 SV=1 - [PHYIP_HUMAN]	8
Serine/threonine-protein phosphatase PP1-beta catalytic subunit OS=Homo sapiens GN=PPP1CB PE=1 SV=3 - [PP1B_HUMAN]	8

26S protease regulatory subunit 10B OS=Homo sapiens GN=PSMC6 PE=1 SV=1 - [PRS10_HUMAN]	8
26S protease regulatory subunit 7 OS=Homo sapiens GN=PSMC2 PE=1 SV=3 - [PRS7_HUMAN]	8
26S proteasome non-ATPase regulatory subunit 13 OS=Homo sapiens GN=PSMD13 PE=1 SV=2 - [PSD13_HUMAN]	8
Sorting and assembly machinery component 50 homolog OS=Homo sapiens GN=SAMM50 PE=1 SV=3 - [SAM50_HUMAN]	8
Isoform 2 of Tenascin-R OS=Homo sapiens GN=TNR - [TENR_HUMAN]	8
Voltage-dependent anion-selective channel protein 1 OS=Homo sapiens GN=VDAC1 PE=1 SV=2 - [VDAC1_HUMAN]	8
Versican core protein OS=Homo sapiens GN=VCAN PE=1 SV=2 - [E9PF17_HUMAN]	8
Endophilin-B2 OS=Homo sapiens GN=SH3GLB2 PE=1 SV=1 - [B7ZC39_HUMAN]	8
Heat shock 70 kDa protein 4L (Fragment) OS=Homo sapiens GN=HSPA4L PE=1 SV=1 - [D6RJ96_HUMAN]	8
Myelin basic protein OS=Homo sapiens GN=MBP PE=1 SV=1 - [J3QL64_HUMAN]	8
Mitochondrial 2-oxoglutarate/malate carrier protein (Fragment) OS=Homo sapiens GN=SLC25A11 PE=1 SV=1 - [I3L1P8_HUMAN]	8
Synapsin-2 OS=Homo sapiens GN=SYN2 PE=1 SV=1 - [A0A087X2E3_HUMAN]	8
Isoform Short of 14-3-3 protein beta/alpha OS=Homo sapiens GN=YWHAB - [1433B_HUMAN]	7
14-3-3 protein theta OS=Homo sapiens GN=YWHAQ PE=1 SV=1 - [1433T_HUMAN]	7
Aconitate hydratase, mitochondrial OS=Homo sapiens GN=ACO2 PE=1 SV=2 - [ACON_HUMAN]	7
AP-3 complex subunit beta-2 OS=Homo sapiens GN=AP3B2 PE=1 SV=2 - [AP3B2_HUMAN]	7
ATP synthase subunit gamma, mitochondrial OS=Homo sapiens GN=ATP5C1 PE=1 SV=1 - [ATPG_HUMAN]	7
Isoform 2 of Adenylyl cyclase-associated protein 1 OS=Homo sapiens GN=CAP1 - [CAP1_HUMAN]	7
Cathepsin D OS=Homo sapiens GN=CTSD PE=1 SV=1 - [CATD_HUMAN]	7
Elongation factor 1-alpha 2 OS=Homo sapiens GN=EEF1A2 PE=1 SV=1 - [EF1A2_HUMAN]	7
Isoform Gamma-A of Fibrinogen gamma chain OS=Homo sapiens GN=FGG - [FIBG_HUMAN]	7
Flotillin-1 OS=Homo sapiens GN=FLOT1 PE=1 SV=3 - [FLOT1_HUMAN]	7
Guanine nucleotide-binding protein subunit alpha-13 OS=Homo sapiens GN=GNA13 PE=1 SV=2 - [GNA13_HUMAN]	7
Guanine nucleotide-binding protein G(i) subunit alpha-1 OS=Homo sapiens GN=GNAI1 PE=1 SV=2 - [GNAI1_HUMAN]	7
Heterogeneous nuclear ribonucleoprotein H2 OS=Homo sapiens GN=HNRNPH2 PE=1 SV=1 - [HNRH2_HUMAN]	7
Immunoglobulin superfamily member 8 OS=Homo sapiens GN=IGSF8 PE=1 SV=1 - [IGSF8_HUMAN]	7
Kinesin-1 heavy chain OS=Homo sapiens GN=KIF5B PE=1 SV=1 - [KINH_HUMAN]	7
Isoform 2 of Liprin-alpha-3 OS=Homo sapiens GN=PPFIA3 - [LIPA3_HUMAN]	7
Malate dehydrogenase, mitochondrial OS=Homo sapiens GN=MDH2 PE=1 SV=3 - [MDHM_HUMAN]	7
Isoform 2 of Mitogen-activated protein kinase 1 OS=Homo sapiens GN=MAPK1 - [MK01_HUMAN]	7
Isoform 2 of Dual specificity mitogen-activated protein kinase kinase 1 OS=Homo sapiens GN=MAP2K1 - [MP2K1_HUMAN]	7
Isoform 2 of Neurochondrin OS=Homo sapiens GN=NCDN - [NCDN_HUMAN]	7
Dihydrolipoyllysine-residue acetyltransferase component of pyruvate dehydrogenase complex, mitochondrial OS=Homo sapiens GN=DLAT PE=1 SV=3 - [ODP2_HUMAN]	7
Isoform 2 of Pyruvate dehydrogenase E1 component subunit beta, mitochondrial OS=Homo sapiens GN=PDHB - [ODPB_HUMAN]	7
Protein-arginine deiminase type-2 OS=Homo sapiens GN=PADI2 PE=2 SV=2 - [PADI2_HUMAN]	7
Poly(rC)-binding protein 1 OS=Homo sapiens GN=PCBP1 PE=1 SV=2 - [PCBP1_HUMAN]	7
Putative 3-phosphoinositide-dependent protein kinase 2 OS=Homo sapiens GN=PDPK2P PE=5 SV=1 - [PDPK2_HUMAN]	7
POTE ankyrin domain family member E OS=Homo sapiens GN=POTEE PE=1 SV=3 - [POTEE_HUMAN]	7
Cytochrome b-c1 complex subunit 1, mitochondrial OS=Homo sapiens GN=UQCRC1 PE=1 SV=3 - [QCR1_HUMAN]	7
Isoform 2 of Heterogeneous nuclear ribonucleoprotein A3 OS=Homo sapiens GN=HNRNPA3 - [ROA3_HUMAN]	7
tRNA-splicing ligase RtcB homolog OS=Homo sapiens GN=RTCB PE=1 SV=1 - [RTCB_HUMAN]	7
Succinyl-CoA:3-ketoacid coenzyme A transferase 1, mitochondrial OS=Homo sapiens GN=OXCT1 PE=1 SV=1 - [SCOT1_HUMAN]	7
Isoform 4 of Tyrosine-protein phosphatase non-receptor type substrate 1 OS=Homo sapiens GN=SIRPA - [SHPS1_HUMAN]	7
Isoform 3 of NCK-interacting protein with SH3 domain OS=Homo sapiens GN=NCKIPSD - [SPN90_HUMAN]	7
Isoform 2 of Spectrin beta chain, non-erythrocytic 2 OS=Homo sapiens GN=SPTBN2 - [SPTN2_HUMAN]	7
Signal transducer and activator of transcription 1-alpha/beta OS=Homo sapiens GN=STAT1 PE=1 SV=2 - [STAT1_HUMAN]	7
Isoform 2-4 of Alpha-synuclein OS=Homo sapiens GN=SNCA - [SYUA_HUMAN]	7
Isoform 3 of T-complex protein 1 subunit eta OS=Homo sapiens GN=CCT7 - [TCPH_HUMAN]	7
T-complex protein 1 subunit zeta OS=Homo sapiens GN=CCT6A PE=1 SV=3 - [TCPZ_HUMAN]	7
Ubiquitin carboxyl-terminal hydrolase isozyme L1 OS=Homo sapiens GN=UCHL1 PE=1 SV=2 - [UCHL1_HUMAN]	7
Voltage-dependent anion-selective channel protein 3 OS=Homo sapiens GN=VDAC3 PE=1 SV=1 - [VDAC3_HUMAN]	7
Vacuolar protein sorting-associated protein 51 homolog OS=Homo sapiens GN=VPS51 PE=1 SV=2 - [VPS51_HUMAN]	7

Isoform 2 of Probable ATP-dependent RNA helicase DDX5 OS=Homo sapiens GN=DDX5 - [DDX5_HUMAN]	7
Catenin delta-2 OS=Homo sapiens GN=CTNND2 PE=1 SV=1 - [E7EPC8_HUMAN]	7
Myosin light polypeptide 6 OS=Homo sapiens GN=MYL6 PE=1 SV=1 - [F8W1R7_HUMAN]	7
Mitogen-activated protein kinase OS=Homo sapiens GN=MAPK3 PE=1 SV=1 - [E9PJF0_HUMAN]	7
Succinate dehydrogenase [ubiquinone] flavoprotein subunit, mitochondrial OS=Homo sapiens GN=SDHA PE=1 SV=1 - [D6RFM5_HUMAN]	7
Myelin-oligodendrocyte glycoprotein OS=Homo sapiens GN=MOG PE=1 SV=1 - [C9JTE0_HUMAN]	7
Mitogen-activated protein kinase 10 (Fragment) OS=Homo sapiens GN=MAPK10 PE=1 SV=1 - [H0Y9H3_HUMAN]	7
Sideroflexin-3 OS=Homo sapiens GN=SFXN3 PE=1 SV=1 - [A0A0A0MS41_HUMAN]	7
Isoform SV of 14-3-3 protein epsilon OS=Homo sapiens GN=YWHAE - [1433E_HUMAN]	6
Isoform 2 of 4F2 cell-surface antigen heavy chain OS=Homo sapiens GN=SLC3A2 - [4F2_HUMAN]	6
Isoform 2 of ATPase family AAA domain-containing protein 3A OS=Homo sapiens GN=ATAD3A - [ATD3A_HUMAN]	6
ATP synthase subunit d, mitochondrial OS=Homo sapiens GN=ATP5H PE=1 SV=3 - [ATP5H_HUMAN]	6
ATP synthase subunit O, mitochondrial OS=Homo sapiens GN=ATP5O PE=1 SV=1 - [ATPO_HUMAN]	6
Protein bassoon OS=Homo sapiens GN=BSN PE=2 SV=4 - [BSN_HUMAN]	6
Cell division control protein 42 homolog OS=Homo sapiens GN=CDC42 PE=1 SV=2 - [CDC42_HUMAN]	6
Cyclin-dependent-like kinase 5 OS=Homo sapiens GN=CDK5 PE=1 SV=3 - [CDK5_HUMAN]	6
Isoform 4 of Clusterin OS=Homo sapiens GN=CLU - [CLUS_HUMAN]	6
Bifunctional coenzyme A synthase OS=Homo sapiens GN=COASY PE=1 SV=4 - [COASY_HUMAN]	6
Isoform 2 of Destrin OS=Homo sapiens GN=DSTN - [DEST_HUMAN]	6
Isoform 2 of Fibrinogen alpha chain OS=Homo sapiens GN=FGA - [FIBA_HUMAN]	6
Tyrosine-protein kinase Fyn OS=Homo sapiens GN=FYN PE=1 SV=3 - [FYN_HUMAN]	6
Mitochondrial glutamate carrier 1 OS=Homo sapiens GN=SLC25A22 PE=1 SV=1 - [GHC1_HUMAN]	6
Guanine nucleotide-binding protein subunit alpha-11 OS=Homo sapiens GN=GNA11 PE=1 SV=2 - [GNA11_HUMAN]	6
Isoform 2 of Glycerol-3-phosphate dehydrogenase, mitochondrial OS=Homo sapiens GN=GPD2 - [GPDM_HUMAN]	6
Isoform Short of Heterogeneous nuclear ribonucleoprotein U OS=Homo sapiens GN=HNRNPU - [HNRPU_HUMAN]	6
Keratin, type I cytoskeletal 15 OS=Homo sapiens GN=KRT15 PE=1 SV=3 - [K1C15_HUMAN]	6
Keratin, type II cytoskeletal 3 OS=Homo sapiens GN=KRT3 PE=1 SV=3 - [K2C3_HUMAN]	6
ATP-dependent 6-phosphofructokinase, muscle type OS=Homo sapiens GN=PFKM PE=1 SV=2 - [PFKAM_HUMAN]	6
Adenylate kinase isoenzyme 1 OS=Homo sapiens GN=AK1 PE=1 SV=3 - [KAD1_HUMAN]	6
Isoform 2 of Calcium/calmodulin-dependent protein kinase type 1D OS=Homo sapiens GN=CAMK1D - [KCC1D_HUMAN]	6
Protein kinase C beta type OS=Homo sapiens GN=PRKCB PE=1 SV=4 - [KPCB_HUMAN]	6
L-lactate dehydrogenase B chain OS=Homo sapiens GN=LDHB PE=1 SV=2 - [LDHB_HUMAN]	6
Mitochondrial carrier homolog 2 OS=Homo sapiens GN=MTCH2 PE=1 SV=1 - [MTCH2_HUMAN]	6
Neurocan core protein OS=Homo sapiens GN=NCAN PE=1 SV=3 - [NCAN_HUMAN]	6
Isoform 10 of Neurofascin OS=Homo sapiens GN=NFASC - [NFASC_HUMAN]	6
Isoform 2 of Non-POU domain-containing octamer-binding protein OS=Homo sapiens GN=NONO - [NONO_HUMAN]	6
Isoform 1 of UDP-N-acetylglucosamine--peptide N-acetylglucosaminyltransferase 110 kDa subunit OS=Homo sapiens GN=OGT - [OGT1_HUMAN]	6
Peptidyl-prolyl cis-trans isomerase A OS=Homo sapiens GN=PPIA PE=1 SV=2 - [PPIA_HUMAN]	6
26S proteasome non-ATPase regulatory subunit 11 OS=Homo sapiens GN=PSMD11 PE=1 SV=3 - [PSD11_HUMAN]	6
26S proteasome non-ATPase regulatory subunit 12 OS=Homo sapiens GN=PSMD12 PE=1 SV=3 - [PSD12_HUMAN]	6
Ras-related protein Rab-3A OS=Homo sapiens GN=RAB3A PE=1 SV=1 - [RAB3A_HUMAN]	6
Ras-related protein Rab-6A OS=Homo sapiens GN=RAB6A PE=1 SV=3 - [RAB6A_HUMAN]	6
Ras-related C3 botulinum toxin substrate 1 OS=Homo sapiens GN=RAC1 PE=1 SV=1 - [RAC1_HUMAN]	6
Retinol dehydrogenase 11 OS=Homo sapiens GN=RDH11 PE=1 SV=2 - [RDH11_HUMAN]	6
Isoform 2 of Regulator of nonsense transcripts 1 OS=Homo sapiens GN=UPF1 - [RENT1_HUMAN]	6
Sideroflexin-1 OS=Homo sapiens GN=SFXN1 PE=1 SV=4 - [SFXN1_HUMAN]	6
Isoleucine--tRNA ligase, mitochondrial OS=Homo sapiens GN=IARS2 PE=1 SV=2 - [SYIM_HUMAN]	6
Tubulin-folding cofactor B OS=Homo sapiens GN=TBCB PE=1 SV=2 - [TBCB_HUMAN]	6
Tubulin polymerization-promoting protein OS=Homo sapiens GN=TPPP PE=1 SV=1 - [TPPP_HUMAN]	6
Isoform 2 of Phosphatidylinositol 5-phosphate 4-kinase type-2 alpha OS=Homo sapiens GN=PIP4K2A - [PI42A_HUMAN]	6
Hypoxia up-regulated protein 1 OS=Homo sapiens GN=HYOU1 PE=1 SV=1 - [A0A087X054_HUMAN]	6
Isoform 2 of Trifunctional enzyme subunit beta, mitochondrial OS=Homo sapiens GN=HADHB - [ECHB_HUMAN]	6
Isoform 2 of Phenylalanine--tRNA ligase alpha subunit OS=Homo sapiens GN=FARSA - [SYFA_HUMAN]	6

Erlin-2 (Fragment) OS=Homo sapiens GN=ERLIN2 PE=1 SV=1 - [E5RHW4_HUMAN]	6
Rho guanine nucleotide exchange factor 7 (Fragment) OS=Homo sapiens GN=ARHGEF7 PE=1 SV=1 - [E7EUY6_HUMAN]	6
Serine/threonine-protein phosphatase (Fragment) OS=Homo sapiens GN=PPP1CA PE=1 SV=1 - [E9PMD7_HUMAN]	6
Dihydropyrimidinase-related protein 1 OS=Homo sapiens GN=CRMP1 PE=1 SV=1 - [E9PD68_HUMAN]	6
Heterogeneous nuclear ribonucleoprotein H OS=Homo sapiens GN=HNRNPH1 PE=1 SV=1 - [E9PCY7_HUMAN]	6
Alpha-crystallin B chain (Fragment) OS=Homo sapiens GN=CRYAB PE=1 SV=1 - [E9PR44_HUMAN]	6
Ras-related protein Rab-7a (Fragment) OS=Homo sapiens GN=RAB7A PE=1 SV=1 - [C9J592_HUMAN]	6
EH domain-containing protein 1 (Fragment) OS=Homo sapiens GN=EHD1 PE=1 SV=1 - [C9JC03_HUMAN]	6
Serine/threonine-protein phosphatase OS=Homo sapiens GN=PPP1CC PE=1 SV=1 - [F8VYE8_HUMAN]	6
ARF GTPase-activating protein GIT1 OS=Homo sapiens GN=GIT1 PE=1 SV=1 - [J3QRU8_HUMAN]	6
Isoform 2 of Guanine nucleotide-binding protein G(I)/G(S)/G(T) subunit beta-1 OS=Homo sapiens GN=GNB1 - [GBB1_HUMAN]	6
Band 4.1-like protein 2 OS=Homo sapiens GN=EPB41L2 PE=1 SV=1 - [I6L9B1_HUMAN]	6
Septin-7 OS=Homo sapiens GN=SEPT7 PE=1 SV=3 - [E7ES33_HUMAN]	6
GRIP1-associated protein 1 OS=Homo sapiens GN=GRIPAP1 PE=1 SV=1 - [A0A087WT45_HUMAN]	6
Isoform 2 of Beta-adducin OS=Homo sapiens GN=ADD2 - [ADDB_HUMAN]	5
AFG3-like protein 2 OS=Homo sapiens GN=AFG3L2 PE=1 SV=2 - [AFG32_HUMAN]	5
Fructose-bisphosphate aldolase A OS=Homo sapiens GN=ALDOA PE=1 SV=2 - [ALDOA_HUMAN]	5
Amine oxidase [flavin-containing] A OS=Homo sapiens GN=MAOA PE=1 SV=1 - [AOFA_HUMAN]	5
ADP-ribosylation factor 3 OS=Homo sapiens GN=ARF3 PE=1 SV=2 - [ARF3_HUMAN]	5
ADP-ribosylation factor-like protein 8B OS=Homo sapiens GN=ARL8B PE=1 SV=1 - [ARL8B_HUMAN]	5
Actin-related protein 2 OS=Homo sapiens GN=ACTR2 PE=1 SV=1 - [ARP2_HUMAN]	5
Isoform 2 of Sodium/potassium-transporting ATPase subunit beta-1 OS=Homo sapiens GN=ATP1B1 - [AT1B1_HUMAN]	5
Isoform 2 of Calcium-dependent secretion activator 1 OS=Homo sapiens GN=CADPS - [CAPS1_HUMAN]	5
Isoform 1 of Cell division control protein 42 homolog OS=Homo sapiens GN=CDC42 - [CDC42_HUMAN]	5
Cyclin-dependent kinase 18 OS=Homo sapiens GN=CDK18 PE=1 SV=3 - [CDK18_HUMAN]	5
MICOS complex subunit MIC19 OS=Homo sapiens GN=CHCHD3 PE=1 SV=1 - [MIC19_HUMAN]	5
MICOS complex subunit MIC25 OS=Homo sapiens GN=CHCHD6 PE=1 SV=1 - [MIC25_HUMAN]	5
Claudin-11 OS=Homo sapiens GN=CLDN11 PE=1 SV=2 - [CLD11_HUMAN]	5
CAP-Gly domain-containing linker protein 2 OS=Homo sapiens GN=CLIP2 PE=1 SV=1 - [CLIP2_HUMAN]	5
Cytochrome c oxidase subunit 4 isoform 1, mitochondrial OS=Homo sapiens GN=COX4I1 PE=1 SV=1 - [COX41_HUMAN]	5
Caskin-1 OS=Homo sapiens GN=CASKIN1 PE=1 SV=1 - [CSKI1_HUMAN]	5
COP9 signalosome complex subunit 4 OS=Homo sapiens GN=COPS4 PE=1 SV=1 - [CSN4_HUMAN]	5
Gap junction alpha-1 protein OS=Homo sapiens GN=GJA1 PE=1 SV=2 - [CXA1_HUMAN]	5
Cytochrome c1, heme protein, mitochondrial OS=Homo sapiens GN=CYC1 PE=1 SV=3 - [CY1_HUMAN]	5
Dynactin subunit 2 OS=Homo sapiens GN=DCTN2 PE=1 SV=4 - [DCTN2_HUMAN]	5
Isoform 2 of N(G),N(G)-dimethylarginine dimethylaminohydrolase 1 OS=Homo sapiens GN=DDAH1 - [DDAH1_HUMAN]	5
Isoform 2 of Probable ATP-dependent RNA helicase DDX17 OS=Homo sapiens GN=DDX17 - [DDX17_HUMAN]	5
Desmin OS=Homo sapiens GN=DES PE=1 SV=3 - [DESM_HUMAN]	5
ATP-dependent RNA helicase A OS=Homo sapiens GN=DHX9 PE=1 SV=4 - [DHX9_HUMAN]	5
GTP-binding protein Di-Ras2 OS=Homo sapiens GN=DIRAS2 PE=1 SV=1 - [DIRA2_HUMAN]	5
Dihydropyrimidinase-related protein 3 OS=Homo sapiens GN=DPYSL3 PE=1 SV=1 - [DPYL3_HUMAN]	5
Isoform 5 of Double-stranded RNA-specific adenosine deaminase OS=Homo sapiens GN=ADAR - [DSRAD_HUMAN]	5
Isoform 2 of Glyceraldehyde-3-phosphate dehydrogenase OS=Homo sapiens GN=GAPDH - [G3P_HUMAN]	5
Rab GDP dissociation inhibitor alpha OS=Homo sapiens GN=GDI1 PE=1 SV=2 - [GDIA_HUMAN]	5
Isoform 3 of Neuronal membrane glycoprotein M6-a OS=Homo sapiens GN=GPM6A - [GPM6A_HUMAN]	5
Glutamate receptor 2 OS=Homo sapiens GN=GRIA2 PE=1 SV=3 - [GRIA2_HUMAN]	5
Histone H1.2 OS=Homo sapiens GN=HIST1H1C PE=1 SV=2 - [H12_HUMAN]	5
Isoform 3 of Heterogeneous nuclear ribonucleoprotein H3 OS=Homo sapiens GN=HNRNPH3 - [HNRH3_HUMAN]	5
Isoform 2 of Heterogeneous nuclear ribonucleoprotein L OS=Homo sapiens GN=HNRNPL - [HNRPL_HUMAN]	5
Hippocalcin-like protein 1 OS=Homo sapiens GN=HPCAL1 PE=1 SV=3 - [HPCL1_HUMAN]	5
Hyaluronan and proteoglycan link protein 2 OS=Homo sapiens GN=HAPLN2 PE=1 SV=1 - [HPLN2_HUMAN]	5
Isoform 2 of Heat shock 70 kDa protein 1A/1B OS=Homo sapiens GN=HSPA1A - [HSP71_HUMAN]	5
Isocitrate dehydrogenase [NAD] subunit alpha, mitochondrial OS=Homo sapiens GN=IDH3A PE=1 SV=1 - [IDH3A_HUMAN]	5

Importin subunit beta-1 OS=Homo sapiens GN=KPNB1 PE=1 SV=2 - [IMB1_HUMAN]	5
IQ motif and SEC7 domain-containing protein 1 OS=Homo sapiens GN=IQSEC1 PE=1 SV=1 - [IQEC1_HUMAN]	5
cAMP-dependent protein kinase type II-beta regulatory subunit OS=Homo sapiens GN=PRKAR2B PE=1 SV=3 - [KAP3_HUMAN]	5
Kelch repeat and BTB domain-containing protein 11 OS=Homo sapiens GN=KBTBD11 PE=1 SV=1 - [KBTBB_HUMAN]	5
Laminin subunit beta-2 OS=Homo sapiens GN=LAMB2 PE=1 SV=2 - [LAMB2_HUMAN]	5
Laminin subunit gamma-1 OS=Homo sapiens GN=LAMC1 PE=1 SV=3 - [LAMC1_HUMAN]	5
Matrin-3 OS=Homo sapiens GN=MATR3 PE=1 SV=2 - [MATR3_HUMAN]	5
Methyl-CpG-binding protein 2 OS=Homo sapiens GN=MECP2 PE=1 SV=1 - [MECP2_HUMAN]	5
Dual specificity mitogen-activated protein kinase kinase 2 OS=Homo sapiens GN=MAP2K2 PE=1 SV=1 - [MP2K2_HUMAN]	5
Myelin proteolipid protein OS=Homo sapiens GN=PLP1 PE=1 SV=2 - [MYPR_HUMAN]	5
Neurocalcin-delta OS=Homo sapiens GN=NCALD PE=1 SV=2 - [NCALD_HUMAN]	5
Protein NipSnap homolog 1 OS=Homo sapiens GN=NIPSNAP1 PE=1 SV=1 - [NIPS1_HUMAN]	5
Dihydrolipoyllysine-residue succinyltransferase component of 2-oxoglutarate dehydrogenase complex, mitochondrial OS=Homo sapiens GN=DLST PE=1 SV=4 - [ODO2_HUMAN]	5
Oligodendrocyte-myelin glycoprotein OS=Homo sapiens GN=OMG PE=1 SV=2 - [OMGP_HUMAN]	5
Pantothenate kinase 4 OS=Homo sapiens GN=PANK4 PE=1 SV=1 - [PANK4_HUMAN]	5
Phosphatidylinositol 5-phosphate 4-kinase type-2 beta OS=Homo sapiens GN=PIP4K2B PE=1 SV=1 - [PI42B_HUMAN]	5
Isoform 3 of Phosphatidylinositol 5-phosphate 4-kinase type-2 gamma OS=Homo sapiens GN=PIP4K2C - [PI42C_HUMAN]	5
Isoform 2 of Serine/threonine-protein phosphatase 2A catalytic subunit alpha isoform OS=Homo sapiens GN=PPP2CA - [PP2AA_HUMAN]	5
Palmitoyl-protein thioesterase 1 OS=Homo sapiens GN=PPT1 PE=1 SV=1 - [PPT1_HUMAN]	5
Isoform Cytoplasmic+peroxisomal of Peroxiredoxin-5, mitochondrial OS=Homo sapiens GN=PRDX5 - [PRDX5_HUMAN]	5
Isoform 2 of Bifunctional purine biosynthesis protein PURH OS=Homo sapiens GN=ATIC - [PUR9_HUMAN]	5
Isoform 2 of Glycogen phosphorylase, muscle form OS=Homo sapiens GN=PYGM - [PYGM_HUMAN]	5
Ras-related protein Rab-10 OS=Homo sapiens GN=RAB10 PE=1 SV=1 - [RAB10_HUMAN]	5
Ras-related protein Rab-5B OS=Homo sapiens GN=RAB5B PE=1 SV=1 - [RAB5B_HUMAN]	5
Ras-related protein Rab-5C OS=Homo sapiens GN=RAB5C PE=1 SV=2 - [RAB5C_HUMAN]	5
Ras-related protein Rap-1A OS=Homo sapiens GN=RAP1A PE=1 SV=1 - [RAP1A_HUMAN]	5
Isoform 3 of Ras-related protein Rap-1b OS=Homo sapiens GN=RAP1B - [RAP1B_HUMAN]	5
Isoform RTN1-B of Reticulon-1 OS=Homo sapiens GN=RTN1 - [RTN1_HUMAN]	5
Isoform RTN1-C of Reticulon-1 OS=Homo sapiens GN=RTN1 - [RTN1_HUMAN]	5
Vesicle-trafficking protein SEC22b OS=Homo sapiens GN=SEC22B PE=1 SV=4 - [SC22B_HUMAN]	5
Isoform Short of Splicing factor, proline- and glutamine-rich OS=Homo sapiens GN=SFPQ - [SFPQ_HUMAN]	5
Serine-threonine kinase receptor-associated protein OS=Homo sapiens GN=STRAP PE=1 SV=1 - [STRAP_HUMAN]	5
Succinyl-CoA ligase [ADP/GDP-forming] subunit alpha, mitochondrial OS=Homo sapiens GN=SUCLG1 PE=1 SV=4 - [SUCA_HUMAN]	5
Isoform 2 of Tubulin alpha chain-like 3 OS=Homo sapiens GN=TUBAL3 - [TBAL3_HUMAN]	5
Isoform 2 of Triosephosphate isomerase OS=Homo sapiens GN=TPI1 - [TPIS_HUMAN]	5
Isoform 2 of Tripeptidyl-peptidase 1 OS=Homo sapiens GN=TPP1 - [TPP1_HUMAN]	5
Synaptic vesicle membrane protein VAT-1 homolog OS=Homo sapiens GN=VAT1 PE=1 SV=2 - [VAT1_HUMAN]	5
Vacuolar protein sorting-associated protein 35 OS=Homo sapiens GN=VPS35 PE=1 SV=2 - [VPS35_HUMAN]	5
WD repeat-containing protein 37 OS=Homo sapiens GN=WDR37 PE=1 SV=2 - [WDR37_HUMAN]	5
Septin-5 (Fragment) OS=Homo sapiens GN=SEPT5 PE=1 SV=1 - [C9JM82_HUMAN]	5
Isoform 2 of Leucine--tRNA ligase, cytoplasmic OS=Homo sapiens GN=LARS - [SYLC_HUMAN]	5
Cofilin 1 (Non-muscle), isoform CRA_a OS=Homo sapiens GN=CFL1 PE=1 SV=1 - [G3V1A4_HUMAN]	5
Casein kinase II subunit alpha OS=Homo sapiens GN=CSNK2A1 PE=1 SV=1 - [E7EU96_HUMAN]	5
Pleckstrin homology domain-containing family B member 1 (Fragment) OS=Homo sapiens GN=PLEKHB1 PE=1 SV=1 - [F5H3M1_HUMAN]	5
Isoform 4 of Heterogeneous nuclear ribonucleoprotein R OS=Homo sapiens GN=HNRNPR - [HNRPR_HUMAN]	5
Protein kinase C and casein kinase substrate in neurons protein 1 OS=Homo sapiens GN=PACSIN1 PE=1 SV=1 - [F6U236_HUMAN]	5
Fibrinogen gamma chain (Fragment) OS=Homo sapiens GN=FGG PE=1 SV=1 - [C9JPQ9_HUMAN]	5
CaM kinase-like vesicle-associated protein OS=Homo sapiens GN=CAMKV PE=1 SV=1 - [C9J9E2_HUMAN]	5
ATP synthase subunit g, mitochondrial OS=Homo sapiens GN=ATP5L PE=1 SV=1 - [E9PN17_HUMAN]	5
Isoform 3 of Receptor-type tyrosine-protein phosphatase zeta OS=Homo sapiens GN=PTPRZ1 - [PTPRZ_HUMAN]	5
ADP-ribosylation factor 4 (Fragment) OS=Homo sapiens GN=ARF4 PE=1 SV=1 - [C9JPM4_HUMAN]	5
T-complex protein 1 subunit epsilon OS=Homo sapiens GN=CCT5 PE=1 SV=1 - [E7ENZ3_HUMAN]	5

Ubiquitin carboxyl-terminal hydrolase OS=Homo sapiens GN=USP14 PE=1 SV=2 - [A6NJA2_HUMAN]	5
Phosphate carrier protein, mitochondrial OS=Homo sapiens GN=SLC25A3 PE=1 SV=1 - [F8VVM2_HUMAN]	5
Lamin-B1 OS=Homo sapiens GN=LMNB1 PE=1 SV=1 - [E9PBF6_HUMAN]	5
NADH dehydrogenase [ubiquinone] 1 beta subcomplex subunit 10 OS=Homo sapiens GN=NDUFB10 PE=1 SV=1 - [H3BPJ9_HUMAN]	5
Nidogen-2 (Fragment) OS=Homo sapiens GN=NID2 PE=1 SV=1 - [H0YJV3_HUMAN]	5
Ras-related protein Rab-11A (Fragment) OS=Homo sapiens GN=RAB11A PE=3 SV=1 - [H3BMH2_HUMAN]	5
Unconventional myosin-Id OS=Homo sapiens GN=MYO1D PE=1 SV=1 - [K7EIG7_HUMAN]	5
Interleukin enhancer-binding factor 2 OS=Homo sapiens GN=ILF2 PE=1 SV=1 - [B4DY09_HUMAN]	5
Peroxiredoxin-1 (Fragment) OS=Homo sapiens GN=PRDX1 PE=1 SV=1 - [A0A0A0MSI0_HUMAN]	5
NAD-dependent protein deacetylase sirtuin-2 OS=Homo sapiens GN=SIRT2 PE=1 SV=1 - [A0A0A0MRF5_HUMAN]	5
40S ribosomal protein S3a (Fragment) OS=Homo sapiens GN=RPS3A PE=1 SV=4 - [D6RB09_HUMAN]	5
Gamma-adducin OS=Homo sapiens GN=ADD3 PE=1 SV=1 - [A0A087WX08_HUMAN]	5
Serine/threonine-protein phosphatase 2A 55 kDa regulatory subunit B alpha isoform OS=Homo sapiens GN=PPP2R2A PE=1 SV=1 - [2ABA_HUMAN]	4
Isoform 2 of Very long-chain specific acyl-CoA dehydrogenase, mitochondrial OS=Homo sapiens GN=ACADVL - [ACADV_HUMAN]	4
Isoform 2 of Annexin A7 OS=Homo sapiens GN=ANXA7 - [ANXA7_HUMAN]	4
ADP-ribosylation factor-like protein 8A OS=Homo sapiens GN=ARL8A PE=1 SV=1 - [ARL8A_HUMAN]	4
Isoform ZA of Plasma membrane calcium-transporting ATPase 2 OS=Homo sapiens GN=ATP2B2 - [AT2B2_HUMAN]	4
Isoform ZK of Plasma membrane calcium-transporting ATPase 4 OS=Homo sapiens GN=ATP2B4 - [AT2B4_HUMAN]	4
Isoform 5 of Alpha-tubulin N-acetyltransferase 1 OS=Homo sapiens GN=ATAT1 - [ATAT_HUMAN]	4
Isoform 2 of Late secretory pathway protein AVL9 homolog OS=Homo sapiens GN=AVL9 - [AVL9_HUMAN]	4
Putative annexin A2-like protein OS=Homo sapiens GN=ANXA2P2 PE=5 SV=2 - [AXA2L_HUMAN]	4
Isoform 2 of Brain acid soluble protein 1 OS=Homo sapiens GN=BASP1 - [BASP1_HUMAN]	4
D-beta-hydroxybutyrate dehydrogenase, mitochondrial OS=Homo sapiens GN=BDH1 PE=1 SV=3 - [BDH_HUMAN]	4
Flavin reductase (NADPH) OS=Homo sapiens GN=BLVRB PE=1 SV=3 - [BLVRB_HUMAN]	4
Cell adhesion molecule 3 OS=Homo sapiens GN=CADM3 PE=1 SV=1 - [CADM3_HUMAN]	4
Cytochrome c oxidase subunit 2 OS=Homo sapiens GN=MT-CO2 PE=1 SV=1 - [COX2_HUMAN]	4
Isoform 5 of Dynactin subunit 1 OS=Homo sapiens GN=DCTN1 - [DCTN1_HUMAN]	4
Trifunctional enzyme subunit alpha, mitochondrial OS=Homo sapiens GN=HADHA PE=1 SV=2 - [ECHA_HUMAN]	4
Elongation factor 1-gamma OS=Homo sapiens GN=EEF1G PE=1 SV=3 - [EF1G_HUMAN]	4
Isoform 2 of ELAV-like protein 2 OS=Homo sapiens GN=ELAVL2 - [ELAV2_HUMAN]	4
Alpha-enolase OS=Homo sapiens GN=ENO1 PE=1 SV=2 - [ENOA_HUMAN]	4
Exocyst complex component 5 OS=Homo sapiens GN=EXOC5 PE=1 SV=1 - [EXOC5_HUMAN]	4
Protein FAM49A OS=Homo sapiens GN=FAM49A PE=1 SV=1 - [FA49A_HUMAN]	4
Isoform 2 of Protein-tyrosine kinase 2-beta OS=Homo sapiens GN=PTK2B - [FAK2_HUMAN]	4
F-box only protein 2 OS=Homo sapiens GN=FBXO2 PE=1 SV=2 - [FBX2_HUMAN]	4
Fascin OS=Homo sapiens GN=FSCN1 PE=1 SV=3 - [FSCN1_HUMAN]	4
Isoform 2 of Growth arrest-specific protein 7 OS=Homo sapiens GN=GAS7 - [GAS7_HUMAN]	4
Guanine nucleotide-binding protein G(q) subunit alpha OS=Homo sapiens GN=GNAQ PE=1 SV=4 - [GNAQ_HUMAN]	4
Glutathione S-transferase kappa 1 OS=Homo sapiens GN=GSTK1 PE=1 SV=3 - [GSTK1_HUMAN]	4
Core histone macro-H2A.2 OS=Homo sapiens GN=H2AFY2 PE=1 SV=3 - [H2AW_HUMAN]	4
Histone H2A.Z OS=Homo sapiens GN=H2AFZ PE=1 SV=2 - [H2AZ_HUMAN]	4
Histone H2B type 1-K OS=Homo sapiens GN=HIST1H2BK PE=1 SV=3 - [H2B1K_HUMAN]	4
Isoform 3 of Heterogeneous nuclear ribonucleoprotein D-like OS=Homo sapiens GN=HNRNPDL - [HNRDL_HUMAN]	4
Heterogeneous nuclear ribonucleoprotein F OS=Homo sapiens GN=HNRNPF PE=1 SV=3 - [HNRPF_HUMAN]	4
Isoform 2 of Heterogeneous nuclear ribonucleoprotein Q OS=Homo sapiens GN=SYNCRIP - [HNRPQ_HUMAN]	4
Hyaluronan and proteoglycan link protein 1 OS=Homo sapiens GN=HAPLN1 PE=2 SV=2 - [HPLN1_HUMAN]	4
Isoform Beta of Heat shock protein 105 kDa OS=Homo sapiens GN=HSPH1 - [HS105_HUMAN]	4
Eukaryotic translation initiation factor 2 subunit 3 OS=Homo sapiens GN=EIF2S3 PE=1 SV=3 - [IF2G_HUMAN]	4
Eukaryotic initiation factor 4A-I OS=Homo sapiens GN=EIF4A1 PE=1 SV=1 - [IF4A1_HUMAN]	4
Eukaryotic initiation factor 4A-II OS=Homo sapiens GN=EIF4A2 PE=1 SV=2 - [IF4A2_HUMAN]	4
Isoform 5 of Interleukin enhancer-binding factor 3 OS=Homo sapiens GN=ILF3 - [ILF3_HUMAN]	4
Protein KIAA1045 OS=Homo sapiens GN=KIAA1045 PE=1 SV=2 - [K1045_HUMAN]	4
Isoform 3 of Adenylate kinase isoenzyme 5 OS=Homo sapiens GN=AK5 - [KAD5_HUMAN]	4

Guanylate kinase OS=Homo sapiens GN=GUK1 PE=1 SV=2 - [KGUA_HUMAN]	4
Kinesin-like protein KIF1A OS=Homo sapiens GN=KIF1A PE=1 SV=2 - [KIF1A_HUMAN]	4
LanC-like protein 1 OS=Homo sapiens GN=LANCL1 PE=1 SV=1 - [LANC1_HUMAN]	4
Microtubule-associated protein RP/EB family member 3 OS=Homo sapiens GN=MAPRE3 PE=1 SV=1 - [MARE3_HUMAN]	4
Malate dehydrogenase, cytoplasmic OS=Homo sapiens GN=MDH1 PE=1 SV=4 - [MDHC_HUMAN]	4
Nck-associated protein 1 OS=Homo sapiens GN=NCKAP1 PE=1 SV=1 - [NCKP1_HUMAN]	4
Nucleoside diphosphate kinase A OS=Homo sapiens GN=NME1 PE=1 SV=1 - [NDKA_HUMAN]	4
NADH dehydrogenase [ubiquinone] 1 alpha subcomplex subunit 8 OS=Homo sapiens GN=NDUFA8 PE=1 SV=3 - [NDUA8_HUMAN]	4
NADH dehydrogenase [ubiquinone] flavoprotein 2, mitochondrial OS=Homo sapiens GN=NDUFV2 PE=1 SV=2 - [NDUV2_HUMAN]	4
Isoform 2 of Nebulette OS=Homo sapiens GN=NEBL - [NEBL_HUMAN]	4
Protein NipSnap homolog 2 OS=Homo sapiens GN=GBAS PE=1 SV=1 - [NIPS2_HUMAN]	4
Lipoamide acyltransferase component of branched-chain alpha-keto acid dehydrogenase complex, mitochondrial OS=Homo sapiens GN=DBT PE=1 SV=3 - [ODB2_HUMAN]	4
Isoform 3 of Pyruvate dehydrogenase E1 component subunit alpha, somatic form, mitochondrial OS=Homo sapiens GN=PDHA1 - [ODPA_HUMAN]	4
Isoform 3 of Optineurin OS=Homo sapiens GN=OPTN - [OPTN_HUMAN]	4
Isoform 5 of Poly(rC)-binding protein 3 OS=Homo sapiens GN=PCBP3 - [PCBP3_HUMAN]	4
Astrocytic phosphoprotein PEA-15 OS=Homo sapiens GN=PEA15 PE=1 SV=2 - [PEA15_HUMAN]	4
Isoform 2 of Major prion protein OS=Homo sapiens GN=PRNP - [PRIO_HUMAN]	4
Proline synthase co-transcribed bacterial homolog protein OS=Homo sapiens GN=PROSC PE=1 SV=1 - [PROSC_HUMAN]	4
Putative Ras-related protein Rab-1C OS=Homo sapiens GN=RAB1C PE=5 SV=2 - [RAB1C_HUMAN]	4
Ras-related protein Rab-6B OS=Homo sapiens GN=RAB6B PE=1 SV=1 - [RAB6B_HUMAN]	4
Heterogeneous nuclear ribonucleoprotein A0 OS=Homo sapiens GN=HNRNPA0 PE=1 SV=1 - [ROA0_HUMAN]	4
40S ribosomal protein S4, X isoform OS=Homo sapiens GN=RPS4X PE=1 SV=2 - [RS4X_HUMAN]	4
Isoform 3 of Reticulon-3 OS=Homo sapiens GN=RTN3 - [RTN3_HUMAN]	4
Isoform 3 of Deoxynucleoside triphosphate triphosphohydrolase SAMHD1 OS=Homo sapiens GN=SAMHD1 - [SAMH1_HUMAN]	4
Isoform 2 of Shootin-1 OS=Homo sapiens GN=KIAA1598 - [SHOT1_HUMAN]	4
Synaptosomal-associated protein 25 OS=Homo sapiens GN=SNAP25 PE=1 SV=1 - [SNP25_HUMAN]	4
Sorting nexin-4 OS=Homo sapiens GN=SNX4 PE=1 SV=1 - [SNX4_HUMAN]	4
Isoform 4 of Spectrin beta chain, non-erythrocytic 4 OS=Homo sapiens GN=SPTBN4 - [SPTN4_HUMAN]	4
Isoform 4 of SRC kinase signaling inhibitor 1 OS=Homo sapiens GN=SRCIN1 - [SRCN1_HUMAN]	4
Proto-oncogene tyrosine-protein kinase Src OS=Homo sapiens GN=SRC PE=1 SV=3 - [SRC_HUMAN]	4
Isoform 2 of SLIT-ROBO Rho GTPase-activating protein 3 OS=Homo sapiens GN=SRGAP3 - [SRGP3_HUMAN]	4
Isoform 2 of Suppressor of G2 allele of SKP1 homolog OS=Homo sapiens GN=SUGT1 - [SUGT1_HUMAN]	4
Bifunctional glutamate/proline--tRNA ligase OS=Homo sapiens GN=EPRS PE=1 SV=5 - [SYEP_HUMAN]	4
Lysine--tRNA ligase OS=Homo sapiens GN=KARS PE=1 SV=3 - [SYK_HUMAN]	4
Isoform Monomeric of Arginine--tRNA ligase, cytoplasmic OS=Homo sapiens GN=RARS - [SYRC_HUMAN]	4
Tubulin beta-1 chain OS=Homo sapiens GN=TUBB1 PE=1 SV=1 - [TBB1_HUMAN]	4
T-complex protein 1 subunit alpha OS=Homo sapiens GN=TCP1 PE=1 SV=1 - [TCPA_HUMAN]	4
Isoform 2 of Transcription intermediary factor 1-beta OS=Homo sapiens GN=TRIM28 - [TIF1B_HUMAN]	4
Talin-1 OS=Homo sapiens GN=TLN1 PE=1 SV=3 - [TLN1_HUMAN]	4
Isoform 4 of Tripartite motif-containing protein 3 OS=Homo sapiens GN=TRIM3 - [TRIM3_HUMAN]	4
Tricarboxylate transport protein, mitochondrial OS=Homo sapiens GN=SLC25A1 PE=1 SV=2 - [TXTP_HUMAN]	4
Ubiquitin-like modifier-activating enzyme 6 OS=Homo sapiens GN=UBA6 PE=1 SV=1 - [UBA6_HUMAN]	4
HOMER1F OS=Homo sapiens GN=HOMER1 PE=1 SV=1 - [Q86YM6_HUMAN]	4
60S ribosomal protein L7 OS=Homo sapiens GN=RPL7 PE=1 SV=1 - [A8MUD9_HUMAN]	4
Isoform 2 of Gamma-soluble NSF attachment protein OS=Homo sapiens GN=NAPG - [SNAG_HUMAN]	4
Isoform 2 of Dihydropteridine reductase OS=Homo sapiens GN=QDPR - [DHPR_HUMAN]	4
Ribosomal protein S6 kinase alpha-2 OS=Homo sapiens GN=RPS6KA2 PE=1 SV=1 - [B7Z3B5_HUMAN]	4
PH and SEC7 domain-containing protein 3 OS=Homo sapiens GN=PSD3 PE=1 SV=1 - [B4DKF8_HUMAN]	4
Isoform 2 of Aspartate--tRNA ligase, cytoplasmic OS=Homo sapiens GN=DARS - [SYDC_HUMAN]	4
Isoform 2 of Glutamate dehydrogenase 1, mitochondrial OS=Homo sapiens GN=GLUD1 - [DHE3_HUMAN]	4
Doublecortin and CaM kinase-like 2, isoform CRA_c OS=Homo sapiens GN=DCLK2 PE=1 SV=1 - [G5E9L9_HUMAN]	4
60S ribosomal protein L18 OS=Homo sapiens GN=RPL18 PE=1 SV=1 - [G3V203_HUMAN]	4

Ankyrin repeat and MYND domain containing 2, isoform CRA_c OS=Homo sapiens GN=ANKMY2 PE=1 SV=1 - [G3V0G5_HUMAN]	4
Dipeptidyl aminopeptidase-like protein 6 OS=Homo sapiens GN=DPP6 PE=1 SV=1 - [E9PDL2_HUMAN]	4
X-ray repair cross-complementing protein 6 OS=Homo sapiens GN=XRCC6 PE=1 SV=1 - [B1AHC9_HUMAN]	4
Cytoplasmic FMR1-interacting protein 2 OS=Homo sapiens GN=CYFIP2 PE=1 SV=1 - [E7EVJ5_HUMAN]	4
V-type proton ATPase subunit E 1 (Fragment) OS=Homo sapiens GN=ATP6V1E1 PE=1 SV=1 - [C9J8H1_HUMAN]	4
Transcription elongation factor B polypeptide 1 (Fragment) OS=Homo sapiens GN=TCEB1 PE=1 SV=1 - [E5RHG8_HUMAN]	4
Protein transport protein Sec23A OS=Homo sapiens GN=SEC23A PE=1 SV=1 - [F5H365_HUMAN]	4
Isoform 2 of Aspartate aminotransferase, mitochondrial OS=Homo sapiens GN=GOT2 - [AATM_HUMAN]	4
Polyubiquitin-C (Fragment) OS=Homo sapiens GN=UBC PE=1 SV=1 - [F5H265_HUMAN]	4
NADH-cytochrome b5 reductase 3 (Fragment) OS=Homo sapiens GN=CYB5R3 PE=1 SV=1 - [B1AHF3_HUMAN]	4
Septin-11 (Fragment) OS=Homo sapiens GN=SEPT11 PE=1 SV=1 - [D6RDU5_HUMAN]	4
Isoform 2 of Heme oxygenase 2 OS=Homo sapiens GN=HMOX2 - [HMOX2_HUMAN]	4
Heterogeneous nuclear ribonucleoproteins C1/C2 (Fragment) OS=Homo sapiens GN=HNRNPC PE=1 SV=1 - [G3V5X6_HUMAN]	4
Isoform 2 of cAMP-dependent protein kinase type I-alpha regulatory subunit OS=Homo sapiens GN=PRKAR1A - [KAP0_HUMAN]	4
Septin-9 (Fragment) OS=Homo sapiens GN=SEPT9 PE=1 SV=1 - [K7EK18_HUMAN]	4
Inosine-5'-monophosphate dehydrogenase 2 (Fragment) OS=Homo sapiens GN=IMPDH2 PE=1 SV=1 - [H0Y4R1_HUMAN]	4
Isoform 2 of STE20/SPS1-related proline-alanine-rich protein kinase OS=Homo sapiens GN=STK39 - [STK39_HUMAN]	4
Myelin expression factor 2 OS=Homo sapiens GN=MYEF2 PE=1 SV=1 - [A0A0A0MQW0_HUMAN]	4
RNA-binding protein Raly (Fragment) OS=Homo sapiens GN=RALY PE=1 SV=4 - [Q5QPM0_HUMAN]	4
Limbic system-associated membrane protein OS=Homo sapiens GN=LSAMP PE=1 SV=2 - [F5H5G1_HUMAN]	4
Heterogeneous nuclear ribonucleoprotein D0 (Fragment) OS=Homo sapiens GN=HNRNPD PE=1 SV=4 - [D6RF44_HUMAN]	4
Synemin OS=Homo sapiens GN=SYNM PE=1 SV=2 - [C9JIE4_HUMAN]	4
Thy-1 membrane glycoprotein (Fragment) OS=Homo sapiens GN=THY1 PE=1 SV=4 - [E9PIM6_HUMAN]	4
Collagen alpha-1(VI) chain OS=Homo sapiens GN=COL6A1 PE=1 SV=1 - [A0A087X0S5_HUMAN]	4
Arf-GAP with dual PH domain-containing protein 1 OS=Homo sapiens GN=ADAP1 PE=1 SV=1 - [A0A087WTN6_HUMAN]	4
WD repeat-containing protein 13 (Fragment) OS=Homo sapiens GN=WDR13 PE=1 SV=1 - [A0A087X091_HUMAN]	4
Isoform 3 of Serine/threonine-protein phosphatase 2A 55 kDa regulatory subunit B gamma isoform OS=Homo sapiens GN=PPP2R2C - [2ABG_HUMAN]	3
Isoform 3 of Acyl-coenzyme A thioesterase 9, mitochondrial OS=Homo sapiens GN=ACOT9 - [ACOT9_HUMAN]	3
Isoform 2 of Arf-GAP with GTPase, ANK repeat and PH domain-containing protein 2 OS=Homo sapiens GN=AGAP2 - [AGAP2_HUMAN]	3
Activator of 90 kDa heat shock protein ATPase homolog 1 OS=Homo sapiens GN=AHSA1 PE=1 SV=1 - [AHSA1_HUMAN]	3
Alcohol dehydrogenase [NADP(+)] OS=Homo sapiens GN=AKR1A1 PE=1 SV=3 - [AK1A1_HUMAN]	3
Isoform 2 of Aldehyde dehydrogenase, mitochondrial OS=Homo sapiens GN=ALDH2 - [ALDH2_HUMAN]	3
Fructose-bisphosphate aldolase C OS=Homo sapiens GN=ALDOC PE=1 SV=2 - [ALDOC_HUMAN]	3
Annexin A5 OS=Homo sapiens GN=ANXA5 PE=1 SV=2 - [ANXA5_HUMAN]	3
Isoform 2 of AP-2 complex subunit mu OS=Homo sapiens GN=AP2M1 - [AP2M1_HUMAN]	3
Apolipoprotein D OS=Homo sapiens GN=APOD PE=1 SV=1 - [APOD_HUMAN]	3
Apolipoprotein L2 OS=Homo sapiens GN=APOL2 PE=1 SV=1 - [APOL2_HUMAN]	3
Isoform 3 of Rho guanine nucleotide exchange factor 2 OS=Homo sapiens GN=ARHGEF2 - [ARHG2_HUMAN]	3
ADP-ribosylation factor-like protein 2 OS=Homo sapiens GN=ARL2 PE=1 SV=4 - [ARL2_HUMAN]	3
Actin-related protein 2/3 complex subunit 3 OS=Homo sapiens GN=ARPC3 PE=1 SV=3 - [ARPC3_HUMAN]	3
Sodium/potassium-transporting ATPase subunit beta-2 OS=Homo sapiens GN=ATP1B2 PE=1 SV=3 - [AT1B2_HUMAN]	3
ATP synthase subunit delta, mitochondrial OS=Homo sapiens GN=ATP5D PE=1 SV=2 - [ATPD_HUMAN]	3
Serine/threonine-protein kinase B-raf OS=Homo sapiens GN=BRAF PE=1 SV=4 - [BRAF_HUMAN]	3
C2 domain-containing protein 2-like OS=Homo sapiens GN=C2CD2L PE=1 SV=3 - [C2C2L_HUMAN]	3
Isoform 5 of CUGBP Elav-like family member 1 OS=Homo sapiens GN=CELF1 - [CELF1_HUMAN]	3
Cell cycle exit and neuronal differentiation protein 1 OS=Homo sapiens GN=CEND1 PE=2 SV=1 - [CEND_HUMAN]	3
CDGSH iron-sulfur domain-containing protein 1 OS=Homo sapiens GN=CISD1 PE=1 SV=1 - [CISD1_HUMAN]	3
CDK5 regulatory subunit-associated protein 3 OS=Homo sapiens GN=CDK5RAP3 PE=1 SV=2 - [CK5P3_HUMAN]	3
UPF0568 protein C14orf166 OS=Homo sapiens GN=C14orf166 PE=1 SV=1 - [CN166_HUMAN]	3
Isoform 2 of Collagen alpha-1(IV) chain OS=Homo sapiens GN=COL4A1 - [CO4A1_HUMAN]	3
Isoform 2C2A' of Collagen alpha-2(VI) chain OS=Homo sapiens GN=COL6A2 - [CO6A2_HUMAN]	3
Coronin-1A OS=Homo sapiens GN=CORO1A PE=1 SV=4 - [COR1A_HUMAN]	3
Glutamate decarboxylase 1 OS=Homo sapiens GN=GAD1 PE=1 SV=1 - [DCE1_HUMAN]	3

Glutamate decarboxylase 2 OS=Homo sapiens GN=GAD2 PE=1 SV=1 - [DCE2_HUMAN]	3
Succinate dehydrogenase [ubiquinone] iron-sulfur subunit, mitochondrial OS=Homo sapiens GN=SDHB PE=1 SV=3 - [SDHB_HUMAN]	3
Isoform 8 of Disks large homolog 1 OS=Homo sapiens GN=DLG1 - [DLG1_HUMAN]	3
DnaJ homolog subfamily A member 1 OS=Homo sapiens GN=DNAJA1 PE=1 SV=2 - [DNJA1_HUMAN]	3
Dual specificity protein phosphatase 3 OS=Homo sapiens GN=DUSP3 PE=1 SV=1 - [DUS3_HUMAN]	3
Isoform 4 of Band 4.1-like protein 1 OS=Homo sapiens GN=EPB41L1 - [E41L1_HUMAN]	3
Isoform 2 of ELAV-like protein 3 OS=Homo sapiens GN=ELAVL3 - [ELAV3_HUMAN]	3
Isoform 2 of 55 kDa erythrocyte membrane protein OS=Homo sapiens GN=MPP1 - [EM55_HUMAN]	3
Isoform 2 of Exocyst complex component 1 OS=Homo sapiens GN=EXOC1 - [EXOC1_HUMAN]	3
Ferritin heavy chain OS=Homo sapiens GN=FTH1 PE=1 SV=2 - [FRIH_HUMAN]	3
Ferritin light chain OS=Homo sapiens GN=FTL PE=1 SV=2 - [FRIL_HUMAN]	3
Isoform 2 of Ganglioside-induced differentiation-associated protein 1 OS=Homo sapiens GN=GDAP1 - [GDAP1_HUMAN]	3
Mitochondrial glutamate carrier 2 OS=Homo sapiens GN=SLC25A18 PE=1 SV=1 - [GHC2_HUMAN]	3
Glycogen synthase kinase-3 beta OS=Homo sapiens GN=GSK3B PE=1 SV=2 - [GSK3B_HUMAN]	3
Isoform 2 of Histone H1.0 OS=Homo sapiens GN=H1F0 - [H10_HUMAN]	3
Histone H2A type 1-H OS=Homo sapiens GN=HIST1H2AH PE=1 SV=3 - [H2A1H_HUMAN]	3
Hemoglobin subunit beta OS=Homo sapiens GN=HBB PE=1 SV=2 - [HBB_HUMAN]	3
Integrin-linked protein kinase OS=Homo sapiens GN=ILK PE=1 SV=2 - [ILK_HUMAN]	3
Importin subunit alpha-5 OS=Homo sapiens GN=KPNA1 PE=1 SV=3 - [IMA5_HUMAN]	3
Isoform 2 of Inverted formin-2 OS=Homo sapiens GN=INF2 - [INF2_HUMAN]	3
Isoform 2 of cAMP-dependent protein kinase catalytic subunit alpha OS=Homo sapiens GN=PRKACA - [KAPCA_HUMAN]	3
Isoform 6 of Kinesin-like protein KIF21A OS=Homo sapiens GN=KIF21A - [KI21A_HUMAN]	3
Calcium/calmodulin-dependent protein kinase kinase 1 OS=Homo sapiens GN=CAMKK1 PE=1 SV=2 - [KKCC1_HUMAN]	3
Isoform 6 of Calcium/calmodulin-dependent protein kinase kinase 2 OS=Homo sapiens GN=CAMKK2 - [KKCC2_HUMAN]	3
LETM1 and EF-hand domain-containing protein 1, mitochondrial OS=Homo sapiens GN=LETM1 PE=1 SV=1 - [LETM1_HUMAN]	3
Leucine-rich repeat-containing protein 47 OS=Homo sapiens GN=LRRC47 PE=1 SV=1 - [LRC47_HUMAN]	3
Isoform 2 of Myelin-associated glycoprotein OS=Homo sapiens GN=MAG - [MAG_HUMAN]	3
Isoform 10 of Serine/threonine-protein kinase MARK2 OS=Homo sapiens GN=MARK2 - [MARK2_HUMAN]	3
Isoform 2 of Mitofusin-2 OS=Homo sapiens GN=MFN2 - [MFN2_HUMAN]	3
Isoform 5 of Misshapen-like kinase 1 OS=Homo sapiens GN=MINK1 - [MINK1_HUMAN]	3
Isoform 2 of Mitochondrial amidoxime reducing component 2 OS=Homo sapiens GN=MARC2 - [MARC2_HUMAN]	3
Dual specificity mitogen-activated protein kinase kinase 4 OS=Homo sapiens GN=MAP2K4 PE=1 SV=1 - [MP2K4_HUMAN]	3
Myotubularin-related protein 5 OS=Homo sapiens GN=SBF1 PE=1 SV=3 - [MTMR5_HUMAN]	3
Isoform 2 of Interferon-induced GTP-binding protein Mx1 OS=Homo sapiens GN=MX1 - [MX1_HUMAN]	3
Neutral cholesterol ester hydrolase 1 OS=Homo sapiens GN=NCEH1 PE=1 SV=3 - [NCEH1_HUMAN]	3
NADPH--cytochrome P450 reductase OS=Homo sapiens GN=POR PE=1 SV=2 - [NCPR_HUMAN]	3
Cytochrome c oxidase subunit NDUFA4 OS=Homo sapiens GN=NDUFA4 PE=1 SV=1 - [NDUA4_HUMAN]	3
NADH dehydrogenase [ubiquinone] 1 alpha subcomplex subunit 12 OS=Homo sapiens GN=NDUFA12 PE=1 SV=1 - [NDUAC_HUMAN]	3
NADH dehydrogenase [ubiquinone] 1 beta subcomplex subunit 7 OS=Homo sapiens GN=NDUFB7 PE=1 SV=4 - [NDUB7_HUMAN]	3
NADH dehydrogenase [ubiquinone] iron-sulfur protein 6, mitochondrial OS=Homo sapiens GN=NDUFS6 PE=1 SV=1 - [NDUS6_HUMAN]	3
Serine/threonine-protein kinase Nek7 OS=Homo sapiens GN=NEK7 PE=1 SV=1 - [NEK7_HUMAN]	3
NHP2-like protein 1 OS=Homo sapiens GN=NHP2L1 PE=1 SV=3 - [NH2L1_HUMAN]	3
NAD(P) transhydrogenase, mitochondrial OS=Homo sapiens GN=NNT PE=1 SV=3 - [NNTM_HUMAN]	3
Nuclear receptor-binding protein OS=Homo sapiens GN=NRBP1 PE=1 SV=1 - [NRBP_HUMAN]	3
Dynamin-like 120 kDa protein, mitochondrial OS=Homo sapiens GN=OPA1 PE=1 SV=3 - [OPA1_HUMAN]	3
Platelet-activating factor acetylhydrolase IB subunit gamma OS=Homo sapiens GN=PAFAH1B3 PE=1 SV=1 - [PA1B3_HUMAN]	3
Proliferation-associated protein 2G4 OS=Homo sapiens GN=PA2G4 PE=1 SV=3 - [PA2G4_HUMAN]	3
Programmed cell death protein 6 OS=Homo sapiens GN=PDCD6 PE=1 SV=1 - [PDCD6_HUMAN]	3
Basement membrane-specific heparan sulfate proteoglycan core protein OS=Homo sapiens GN=HSPG2 PE=1 SV=4 - [PGBM_HUMAN]	3
Isoform 2 of Phytanoyl-CoA hydroxylase-interacting protein-like OS=Homo sapiens GN=PHYHIPL - [PHIPL_HUMAN]	3
Protein-L-isoaspartate(D-aspartate) O-methyltransferase OS=Homo sapiens GN=PCMT1 PE=1 SV=4 - [PIMT_HUMAN]	3
Peptidyl-prolyl cis-trans isomerase NIMA-interacting 1 OS=Homo sapiens GN=PIN1 PE=1 SV=1 - [PIN1_HUMAN]	3
Phosphomevalonate kinase OS=Homo sapiens GN=PMVK PE=1 SV=3 - [PMVK_HUMAN]	3

Peptidyl-prolyl cis-trans isomerase B OS=Homo sapiens GN=PPIB PE=1 SV=2 - [PPIB_HUMAN]	3
Protoporphyrinogen oxidase OS=Homo sapiens GN=PPOX PE=1 SV=1 - [PPOX_HUMAN]	3
Transcriptional activator protein Pur-alpha OS=Homo sapiens GN=PURA PE=1 SV=2 - [PURA_HUMAN]	3
Isoform 2 of Rab GTPase-binding effector protein 1 OS=Homo sapiens GN=RABEP1 - [RABE1_HUMAN]	3
GTP-binding nuclear protein Ran OS=Homo sapiens GN=RAN PE=1 SV=3 - [RAN_HUMAN]	3
Ras-related protein Rap-2b OS=Homo sapiens GN=RAP2B PE=1 SV=1 - [RAP2B_HUMAN]	3
Rab11 family-interacting protein 5 OS=Homo sapiens GN=RAB11FIP5 PE=1 SV=1 - [RFIP5_HUMAN]	3
Rho-related GTP-binding protein RhoB OS=Homo sapiens GN=RHOB PE=1 SV=1 - [RHOB_HUMAN]	3
Ribonuclease inhibitor OS=Homo sapiens GN=RNH1 PE=1 SV=2 - [RINI_HUMAN]	3
Isoform 2 of 60S ribosomal protein L11 OS=Homo sapiens GN=RPL11 - [RL11_HUMAN]	3
60S ribosomal protein L12 OS=Homo sapiens GN=RPL12 PE=1 SV=1 - [RL12_HUMAN]	3
60S ribosomal protein L13 OS=Homo sapiens GN=RPL13 PE=1 SV=4 - [RL13_HUMAN]	3
60S ribosomal protein L23 OS=Homo sapiens GN=RPL23 PE=1 SV=1 - [RL23_HUMAN]	3
Isoform 2 of Rabphilin-3A OS=Homo sapiens GN=RPH3A - [RP3A_HUMAN]	3
Isoform 3 of Rap1 GTPase-activating protein 2 OS=Homo sapiens GN=RAP1GAP2 - [RPGP2_HUMAN]	3
40S ribosomal protein S3 OS=Homo sapiens GN=RPS3 PE=1 SV=2 - [RS3_HUMAN]	3
RNA 3'-terminal phosphate cyclase OS=Homo sapiens GN=RTCA PE=1 SV=1 - [RTCA_HUMAN]	3
GTP-binding protein SAR1a OS=Homo sapiens GN=SAR1A PE=1 SV=1 - [SAR1A_HUMAN]	3
Lysosome membrane protein 2 OS=Homo sapiens GN=SCARB2 PE=1 SV=2 - [SCRB2_HUMAN]	3
Sideroflexin-5 OS=Homo sapiens GN=SFXN5 PE=1 SV=1 - [SFXN5_HUMAN]	3
Synaptogyrin-3 OS=Homo sapiens GN=SYNGR3 PE=1 SV=2 - [SNG3_HUMAN]	3
Beta-1-syntrophin OS=Homo sapiens GN=SNTB1 PE=1 SV=3 - [SNTB1_HUMAN]	3
Sorting nexin-3 OS=Homo sapiens GN=SNX3 PE=1 SV=3 - [SNX3_HUMAN]	3
Erythrocyte band 7 integral membrane protein OS=Homo sapiens GN=STOM PE=1 SV=3 - [STOM_HUMAN]	3
Syntaxin-binding protein 6 OS=Homo sapiens GN=STXBP6 PE=1 SV=2 - [STXB6_HUMAN]	3
Succinyl-CoA ligase [GDP-forming] subunit beta, mitochondrial OS=Homo sapiens GN=SUCLG2 PE=1 SV=2 - [SUCB2_HUMAN]	3
Synapsin-3 OS=Homo sapiens GN=SYN3 PE=1 SV=2 - [SYN3_HUMAN]	3
Synaptophysin OS=Homo sapiens GN=SYP PE=1 SV=3 - [SYPH_HUMAN]	3
Isoform 2 of TSC22 domain family protein 1 OS=Homo sapiens GN=TSC22D1 - [T22D1_HUMAN]	3
Tubulin-specific chaperone D OS=Homo sapiens GN=TBCD PE=1 SV=2 - [TBCD_HUMAN]	3
Transketolase OS=Homo sapiens GN=TKT PE=1 SV=3 - [TKT_HUMAN]	3
Mitochondrial import receptor subunit TOM70 OS=Homo sapiens GN=TOMM70A PE=1 SV=1 - [TOM70_HUMAN]	3
Isoform 2 of Tumor protein D52 OS=Homo sapiens GN=TPD52 - [TPD52_HUMAN]	3
Tripartite motif-containing protein 2 OS=Homo sapiens GN=TRIM2 PE=1 SV=1 - [TRIM2_HUMAN]	3
NEDD8-conjugating enzyme Ubc12 OS=Homo sapiens GN=UBE2M PE=1 SV=1 - [UBC12_HUMAN]	3
Ubiquitin-conjugating enzyme E2 N OS=Homo sapiens GN=UBE2N PE=1 SV=1 - [UBE2N_HUMAN]	3
Isoform 2 of Mitochondrial uncoupling protein 4 OS=Homo sapiens GN=SLC25A27 - [UCP4_HUMAN]	3
Cytochrome b-c1 complex subunit Rieske, mitochondrial OS=Homo sapiens GN=UQCRFS1 PE=1 SV=2 - [UCRI_HUMAN]	3
Vesicle-associated membrane protein-associated protein A OS=Homo sapiens GN=VAPA PE=1 SV=3 - [VAPA_HUMAN]	3
V-type proton ATPase subunit C 1 OS=Homo sapiens GN=ATP6V1C1 PE=1 SV=4 - [VATC1_HUMAN]	3
Vacuolar protein sorting-associated protein 29 OS=Homo sapiens GN=VPS29 PE=1 SV=1 - [VPS29_HUMAN]	3
WD repeat-containing protein 1 OS=Homo sapiens GN=WDR1 PE=1 SV=4 - [WDR1_HUMAN]	3
Isoform 2 of WD repeat-containing protein 47 OS=Homo sapiens GN=WDR47 - [WDR47_HUMAN]	3
Isoform 2 of Ras-related protein Rab-5A OS=Homo sapiens GN=RAB5A - [RAB5A_HUMAN]	3
Profilin-2 OS=Homo sapiens GN=PFN2 PE=1 SV=1 - [C9J712_HUMAN]	3
Citrate synthase, mitochondrial OS=Homo sapiens GN=CS PE=1 SV=1 - [B4DJV2_HUMAN]	3
Emerin OS=Homo sapiens GN=EMD PE=1 SV=1 - [Q5HY57_HUMAN]	3
Transcription elongation factor B (SIII), polypeptide 2 (18kDa, elongin B), isoform CRA_b OS=Homo sapiens GN=TCEB2 PE=1 SV=1 - [B8ZZU8_HUMAN]	3
Dolichyl-diphosphooligosaccharide--protein glycosyltransferase subunit 1 OS=Homo sapiens GN=RPN1 PE=1 SV=1 - [B7Z4L4_HUMAN]	3
Profilin-2 OS=Homo sapiens GN=PFN2 PE=1 SV=1 - [C9J0J7_HUMAN]	3
Isocitrate dehydrogenase [NAD] subunit, mitochondrial OS=Homo sapiens GN=IDH3G PE=3 SV=1 - [G5E9Q7_HUMAN]	3
Disks large homolog 2 OS=Homo sapiens GN=DLG2 PE=1 SV=1 - [B7Z264_HUMAN]	3
Isoform 2 of Stomatin-like protein 2, mitochondrial OS=Homo sapiens GN=STOML2 - [STML2_HUMAN]	3

Protein transport protein Sec24C OS=Homo sapiens GN=SEC24C PE=1 SV=1 - [G5EA31_HUMAN]	3
Copine-8 OS=Homo sapiens GN=CPNE8 PE=1 SV=2 - [E7ENV7_HUMAN]	3
Interferon-inducible double-stranded RNA-dependent protein kinase activator A OS=Homo sapiens GN=PRKRA PE=1 SV=1 - [F8WEG8_HUMAN]	3
NADH dehydrogenase [ubiquinone] iron-sulfur protein 8, mitochondrial (Fragment) OS=Homo sapiens GN=NDUFS8 PE=1 SV=1 - [E9PN51_HUMAN]	3
CLIP-associating protein 1 (Fragment) OS=Homo sapiens GN=CLASP1 PE=1 SV=1 - [H0Y5T1_HUMAN]	3
Neutral alpha-glucosidase AB OS=Homo sapiens GN=GANAB PE=1 SV=1 - [F5H6X6_HUMAN]	3
Isoform 5 of MAGUK p55 subfamily member 2 OS=Homo sapiens GN=MPP2 - [MPP2_HUMAN]	3
Vacuolar protein sorting-associated protein 53 homolog OS=Homo sapiens GN=VPS53 PE=1 SV=1 - [F6VX93_HUMAN]	3
26S protease regulatory subunit 6A OS=Homo sapiens GN=PSMC3 PE=1 SV=1 - [E9PM69_HUMAN]	3
Rho GTPase-activating protein 44 OS=Homo sapiens GN=ARHGAP44 PE=1 SV=2 - [F5H6L3_HUMAN]	3
Isoform 3 of Excitatory amino acid transporter 2 OS=Homo sapiens GN=SLC1A2 - [EAA2_HUMAN]	3
UMP-CMP kinase OS=Homo sapiens GN=CMPK1 PE=1 SV=1 - [Q5T0D2_HUMAN]	3
ADP-ribosylation factor 5 (Fragment) OS=Homo sapiens GN=ARF5 PE=1 SV=1 - [C9J1Z8_HUMAN]	3
NADH dehydrogenase [ubiquinone] 1 alpha subcomplex subunit 5 OS=Homo sapiens GN=NDUFA5 PE=1 SV=1 - [F8WAS3_HUMAN]	3
60S ribosomal protein L14 OS=Homo sapiens GN=RPL14 PE=1 SV=1 - [E7EPB3_HUMAN]	3
60S ribosomal protein L24 OS=Homo sapiens GN=RPL24 PE=1 SV=1 - [C9JXB8_HUMAN]	3
COP9 signalosome complex subunit 7a (Fragment) OS=Homo sapiens GN=COPS7A PE=1 SV=1 - [F5H4U8_HUMAN]	3
CD59 glycoprotein OS=Homo sapiens GN=CD59 PE=1 SV=1 - [E9PNW4_HUMAN]	3
Actin-related protein 2/3 complex subunit 4 OS=Homo sapiens GN=ARPC4 PE=1 SV=1 - [F8WDD7_HUMAN]	3
Glutamate receptor 3 OS=Homo sapiens GN=GRIA3 PE=1 SV=1 - [A0A087WYJ6_HUMAN]	3
AP2-associated protein kinase 1 OS=Homo sapiens GN=AAK1 PE=1 SV=1 - [E9PG46_HUMAN]	3
Kinesin light chain 2 OS=Homo sapiens GN=KLC2 PE=1 SV=1 - [A8MZ87_HUMAN]	3
60S acidic ribosomal protein P0 (Fragment) OS=Homo sapiens GN=RPLP0 PE=1 SV=1 - [F8VPE8_HUMAN]	3
Sickle tail protein homolog (Fragment) OS=Homo sapiens GN=KIAA1217 PE=1 SV=1 - [Q5T5P0_HUMAN]	3
Microtubule-associated protein RP/EB family member 2 (Fragment) OS=Homo sapiens GN=MAPRE2 PE=1 SV=1 - [M0QX52_HUMAN]	3
Protein disulfide-isomerase A3 (Fragment) OS=Homo sapiens GN=PDIA3 PE=1 SV=1 - [H7BZJ3_HUMAN]	3
Dephospho-CoA kinase domain-containing protein (Fragment) OS=Homo sapiens GN=DCAKD PE=1 SV=1 - [K7ESP4_HUMAN]	3
Apolipoprotein O (Fragment) OS=Homo sapiens GN=APOO PE=1 SV=1 - [H7C1U8_HUMAN]	3
Septin-4 (Fragment) OS=Homo sapiens GN=SEPT4 PE=1 SV=1 - [J3QLR2_HUMAN]	3
Pyruvate kinase (Fragment) OS=Homo sapiens GN=PKM PE=1 SV=1 - [H3BTN5_HUMAN]	3
Fibronectin type III and SPRY domain-containing protein 1 OS=Homo sapiens GN=FSD1 PE=1 SV=1 - [M0R366_HUMAN]	3
Regulator of G-protein-signaling 7 (Fragment) OS=Homo sapiens GN=RGS7 PE=1 SV=1 - [Q5T3H5_HUMAN]	3
WD repeat-containing protein 7 OS=Homo sapiens GN=WDR7 PE=1 SV=1 - [A2RRE0_HUMAN]	3
Keratin, type II cuticular Hb1 OS=Homo sapiens GN=KRT81 PE=1 SV=1 - [A0A087X106_HUMAN]	3
Mitochondrial carrier homolog 1 OS=Homo sapiens GN=MTCH1 PE=1 SV=1 - [F6WFU6_HUMAN]	3
Isocitrate dehydrogenase [NAD] subunit, mitochondrial OS=Homo sapiens GN=IDH3B PE=1 SV=1 - [A0A087X2E5_HUMAN]	3
cAMP-dependent protein kinase catalytic subunit beta OS=Homo sapiens GN=PRKACB PE=1 SV=1 - [A0A087WVC4_HUMAN]	3
Serine/threonine-protein phosphatase 2A activator OS=Homo sapiens GN=PPP2R4 PE=1 SV=1 - [F6WIT2_HUMAN]	3
C-terminal-binding protein 1 (Fragment) OS=Homo sapiens GN=CTBP1 PE=1 SV=4 - [D6RAX2_HUMAN]	3
Septin-8 OS=Homo sapiens GN=SEPT8 PE=1 SV=1 - [A0A087X142_HUMAN]	3
Arf-GAP with GTPase, ANK repeat and PH domain-containing protein 1 OS=Homo sapiens GN=AGAP1 PE=1 SV=1 - [A0A087X1U1_HUMAN]	3
CUGBP Elav-like family member 2 OS=Homo sapiens GN=CELF2 PE=1 SV=1 - [V9GYD9_HUMAN]	3
ATPase, H+ transporting, lysosomal accessory protein 1, isoform CRA_c OS=Homo sapiens GN=ATP6AP1 PE=4 SV=1 - [A0A0C4DGX8_HUMAN]	3
Tyrosine--tRNA ligase, cytoplasmic OS=Homo sapiens GN=YARS PE=4 SV=1 - [A0A0C4DGZ5_HUMAN]	3
Isoform 2 of HLA class I histocompatibility antigen, Cw-16 alpha chain OS=Homo sapiens GN=HLA-C - [1C16_HUMAN]	2
Isoform Delta-3 of Serine/threonine-protein phosphatase 2A 56 kDa regulatory subunit delta isoform OS=Homo sapiens GN=PPP2R5D - [2A5D_HUMAN]	2
Isoform APP639 of Amyloid beta A4 protein OS=Homo sapiens GN=APP - [A4_HUMAN]	2
Isoform 4 of Active breakpoint cluster region-related protein OS=Homo sapiens GN=ABR - [ABR_HUMAN]	2
Isoform 3 of Arf-GAP with GTPase, ANK repeat and PH domain-containing protein 3 OS=Homo sapiens GN=AGAP3 - [AGAP3_HUMAN]	2
Isoform 6 of Agrin OS=Homo sapiens GN=AGRN - [AGRIN_HUMAN]	2
Rabankyrin-5 OS=Homo sapiens GN=ANKFY1 PE=1 SV=2 - [ANFY1_HUMAN]	2
Isoform 10 of Ankyrin repeat and sterile alpha motif domain-containing protein 1B OS=Homo sapiens GN=ANKS1B - [ANS1B_HUMAN]	2
Isoform 2 of Adipocyte plasma membrane-associated protein OS=Homo sapiens GN=APMAP - [APMAP_HUMAN]	2

Apolipoprotein E OS=Homo sapiens GN=APOE PE=1 SV=1 - [APOE_HUMAN]	2
Actin-related protein 3 OS=Homo sapiens GN=ACTR3 PE=1 SV=3 - [ARP3_HUMAN]	2
Isoform 2 of ATPase family AAA domain-containing protein 1 OS=Homo sapiens GN=ATAD1 - [ATAD1_HUMAN]	2
Isoform 4 of ATP synthase subunit f, mitochondrial OS=Homo sapiens GN=ATP5J2 - [ATPK_HUMAN]	2
Isoform 3 of Basigin OS=Homo sapiens GN=BSG - [BASI_HUMAN]	2
F-actin-capping protein subunit alpha-2 OS=Homo sapiens GN=CAPZA2 PE=1 SV=3 - [CAZA2_HUMAN]	2
Isoform 2 of Cell cycle control protein 50A OS=Homo sapiens GN=TMEM30A - [CC50A_HUMAN]	2
Cell cycle and apoptosis regulator protein 2 OS=Homo sapiens GN=CCAR2 PE=1 SV=2 - [CCAR2_HUMAN]	2
Isoform 3 of Centromere protein V OS=Homo sapiens GN=CENPV - [CENPV_HUMAN]	2
10 kDa heat shock protein, mitochondrial OS=Homo sapiens GN=HSPE1 PE=1 SV=2 - [CH10_HUMAN]	2
Choline dehydrogenase, mitochondrial OS=Homo sapiens GN=CHDH PE=1 SV=2 - [CHDH_HUMAN]	2
E3 ubiquitin-protein ligase CHIP OS=Homo sapiens GN=STUB1 PE=1 SV=2 - [CHIP_HUMAN]	2
Cytoskeleton-associated protein 4 OS=Homo sapiens GN=CKAP4 PE=1 SV=2 - [CKAP4_HUMAN]	2
Isoform 2 of Cytoskeleton-associated protein 5 OS=Homo sapiens GN=CKAP5 - [CKAP5_HUMAN]	2
Collagen alpha-2(IV) chain OS=Homo sapiens GN=COL4A2 PE=1 SV=4 - [CO4A2_HUMAN]	2
Cytochrome c oxidase subunit 5A, mitochondrial OS=Homo sapiens GN=COX5A PE=1 SV=2 - [COX5A_HUMAN]	2
Cytochrome c oxidase subunit 5B, mitochondrial OS=Homo sapiens GN=COX5B PE=1 SV=2 - [COX5B_HUMAN]	2
Copine-3 OS=Homo sapiens GN=CPNE3 PE=1 SV=1 - [CPNE3_HUMAN]	2
Copine-4 OS=Homo sapiens GN=CPNE4 PE=2 SV=1 - [CPNE4_HUMAN]	2
Isoform 2 of Cold shock domain-containing protein E1 OS=Homo sapiens GN=CSDE1 - [CSDE1_HUMAN]	2
Tyrosine-protein kinase CSK OS=Homo sapiens GN=CSK PE=1 SV=1 - [CSK_HUMAN]	2
Isoform 1 of Catenin delta-1 OS=Homo sapiens GN=CTNND1 - [CTND1_HUMAN]	2
Cytochrome c oxidase subunit 6B1 OS=Homo sapiens GN=COX6B1 PE=1 SV=2 - [CX6B1_HUMAN]	2
Isoform 2 of Epimerase family protein SDR39U1 OS=Homo sapiens GN=SDR39U1 - [D39U1_HUMAN]	2
Cytoplasmic dynein 1 light intermediate chain 2 OS=Homo sapiens GN=DYNC1LI2 PE=1 SV=1 - [DC1L2_HUMAN]	2
Probable ATP-dependent RNA helicase DDX6 OS=Homo sapiens GN=DDX6 PE=1 SV=2 - [DDX6_HUMAN]	2
Isoform 3 of Dematin OS=Homo sapiens GN=DMTN - [DEMA_HUMAN]	2
Peroxisomal multifunctional enzyme type 2 OS=Homo sapiens GN=HSD17B4 PE=1 SV=3 - [DHB4_HUMAN]	2
Isoform 2 of Disks large homolog 3 OS=Homo sapiens GN=DLG3 - [DLG3_HUMAN]	2
DnaJ homolog subfamily A member 2 OS=Homo sapiens GN=DNAJA2 PE=1 SV=1 - [DNJA2_HUMAN]	2
DnaJ homolog subfamily B member 4 OS=Homo sapiens GN=DNAJB4 PE=1 SV=1 - [DNJB4_HUMAN]	2
Isoform 2 of DnaJ homolog subfamily C member 5 OS=Homo sapiens GN=DNAJC5 - [DNJC5_HUMAN]	2
Dihydropyrimidinase-related protein 4 OS=Homo sapiens GN=DPYSL4 PE=1 SV=2 - [DPYL4_HUMAN]	2
Dynein light chain 1, cytoplasmic OS=Homo sapiens GN=DYNLL1 PE=1 SV=1 - [DYL1_HUMAN]	2
EH domain-containing protein 2 OS=Homo sapiens GN=EHD2 PE=1 SV=2 - [EHD2_HUMAN]	2
Eukaryotic translation initiation factor 3 subunit F OS=Homo sapiens GN=EIF3F PE=1 SV=1 - [EIF3F_HUMAN]	2
Eukaryotic translation initiation factor 3 subunit I OS=Homo sapiens GN=EIF3I PE=1 SV=1 - [EIF3I_HUMAN]	2
ELAV-like protein 1 OS=Homo sapiens GN=ELAVL1 PE=1 SV=2 - [ELAV1_HUMAN]	2
Engulfment and cell motility protein 1 OS=Homo sapiens GN=ELMO1 PE=1 SV=2 - [ELMO1_HUMAN]	2
Mammalian ependymin-related protein 1 OS=Homo sapiens GN=EPDR1 PE=1 SV=2 - [EPDR1_HUMAN]	2
Isoform 2 of Exocyst complex component 7 OS=Homo sapiens GN=EXOC7 - [EXOC7_HUMAN]	2
Protein FAM98B OS=Homo sapiens GN=FAM98B PE=1 SV=1 - [FA98B_HUMAN]	2
FERM, RhoGEF and pleckstrin domain-containing protein 1 OS=Homo sapiens GN=FARP1 PE=1 SV=1 - [FARP1_HUMAN]	2
Mitochondrial fission 1 protein OS=Homo sapiens GN=FIS1 PE=1 SV=2 - [FIS1_HUMAN]	2
Isoform 2 of Far upstream element-binding protein 3 OS=Homo sapiens GN=FUBP3 - [FUBP3_HUMAN]	2
Isoform 11 of ARF GTPase-activating protein GIT2 OS=Homo sapiens GN=GIT2 - [GIT2_HUMAN]	2
Glutamine synthetase OS=Homo sapiens GN=GLUL PE=1 SV=4 - [GLNA_HUMAN]	2
Glutaredoxin-3 OS=Homo sapiens GN=GLRX3 PE=1 SV=2 - [GLRX3_HUMAN]	2
Histone H1x OS=Homo sapiens GN=H1FX PE=1 SV=1 - [H1X_HUMAN]	2
Histone H2A type 2-B OS=Homo sapiens GN=HIST2H2AB PE=1 SV=3 - [H2A2B_HUMAN]	2
Putative histone H2B type 2-D OS=Homo sapiens GN=HIST2H2BD PE=5 SV=3 - [H2B2D_HUMAN]	2
HLA class I histocompatibility antigen, alpha chain E OS=Homo sapiens GN=HLA-E PE=1 SV=3 - [HLAE_HUMAN]	2
Hydroxymethylglutaryl-CoA synthase, cytoplasmic OS=Homo sapiens GN=HMGCS1 PE=1 SV=2 - [HMCS1_HUMAN]	2

Isoform 2 of Hydroxysteroid dehydrogenase-like protein 2 OS=Homo sapiens GN=HSDL2 - [HSDL2_HUMAN]	2
Intercellular adhesion molecule 5 OS=Homo sapiens GN=ICAM5 PE=1 SV=3 - [ICAM5_HUMAN]	2
Importin subunit alpha-4 OS=Homo sapiens GN=KPNA3 PE=1 SV=2 - [IMA4_HUMAN]	2
Importin subunit alpha-7 OS=Homo sapiens GN=KPNA6 PE=1 SV=1 - [IMA7_HUMAN]	2
Inositol-trisphosphate 3-kinase A OS=Homo sapiens GN=ITPKA PE=1 SV=1 - [IP3KA_HUMAN]	2
Keratin, type II cytoskeletal 74 OS=Homo sapiens GN=KRT74 PE=1 SV=2 - [K2C74_HUMAN]	2
BTB/POZ domain-containing protein KCTD12 OS=Homo sapiens GN=KCTD12 PE=1 SV=1 - [KCD12_HUMAN]	2
Creatine kinase U-type, mitochondrial OS=Homo sapiens GN=CKMT1A PE=1 SV=1 - [KCRU_HUMAN]	2
Protein kinase C epsilon type OS=Homo sapiens GN=PRKCE PE=1 SV=1 - [KPCE_HUMAN]	2
Isoform 4 of L-lactate dehydrogenase A chain OS=Homo sapiens GN=LDHA - [LDHA_HUMAN]	2
Isoform 2 of Leucine-rich glioma-inactivated protein 1 OS=Homo sapiens GN=LGI1 - [LGI1_HUMAN]	2
Protein lin-7 homolog A OS=Homo sapiens GN=LIN7A PE=1 SV=2 - [LIN7A_HUMAN]	2
Isoform 6 of Liprin-alpha-2 OS=Homo sapiens GN=PPFIA2 - [LIPA2_HUMAN]	2
Isoform 2 of Hormone-sensitive lipase OS=Homo sapiens GN=LIPE - [LIPS_HUMAN]	2
Lymphocyte antigen 6H OS=Homo sapiens GN=LY6H PE=2 SV=1 - [LY6H_HUMAN]	2
Isoform 3 of Methionine adenosyltransferase 2 subunit beta OS=Homo sapiens GN=MAT2B - [MAT2B_HUMAN]	2
Metallo-beta-lactamase domain-containing protein 2 OS=Homo sapiens GN=MBLAC2 PE=1 SV=3 - [MBLC2_HUMAN]	2
Isoform 4 of MMS19 nucleotide excision repair protein homolog OS=Homo sapiens GN=MMS19 - [MMS19_HUMAN]	2
Isoform 2 of MOB kinase activator 2 OS=Homo sapiens GN=MOB2 - [MOB2_HUMAN]	2
Isoform 3 of Serine/threonine-protein kinase MRCK alpha OS=Homo sapiens GN=CDC42BPA - [MRCKA_HUMAN]	2
Isoform 1A of Myotubularin-related protein 1 OS=Homo sapiens GN=MTMR1 - [MTMR1_HUMAN]	2
Isoform 2 of Methylthioribose-1-phosphate isomerase OS=Homo sapiens GN=MRI1 - [MTNA_HUMAN]	2
Isoform 3 of Metaxin-3 OS=Homo sapiens GN=MTX3 - [MTX3_HUMAN]	2
Methylmalonyl-CoA mutase, mitochondrial OS=Homo sapiens GN=MUT PE=1 SV=4 - [MUTA_HUMAN]	2
Myosin-10 OS=Homo sapiens GN=MYH10 PE=1 SV=3 - [MYH10_HUMAN]	2
NADH dehydrogenase [ubiquinone] 1 beta subcomplex subunit 3 OS=Homo sapiens GN=NDUFB3 PE=1 SV=3 - [NDUB3_HUMAN]	2
NADH dehydrogenase [ubiquinone] iron-sulfur protein 4, mitochondrial OS=Homo sapiens GN=NDUFS4 PE=1 SV=1 - [NDUS4_HUMAN]	2
NADH dehydrogenase [ubiquinone] iron-sulfur protein 5 OS=Homo sapiens GN=NDUFS5 PE=1 SV=3 - [NDUS5_HUMAN]	2
NADH dehydrogenase [ubiquinone] iron-sulfur protein 7, mitochondrial OS=Homo sapiens GN=NDUFS7 PE=1 SV=3 - [NDUS7_HUMAN]	2
Cancer-related nucleoside-triphosphatase OS=Homo sapiens GN=NTPCR PE=1 SV=1 - [NTPCR_HUMAN]	2
Isoform 3 of Opioid-binding protein/cell adhesion molecule OS=Homo sapiens GN=OPCML - [OPCM_HUMAN]	2
[Pyruvate dehydrogenase (acetyl-transferring)] kinase isozyme 3, mitochondrial OS=Homo sapiens GN=PDK3 PE=1 SV=1 - [PDK3_HUMAN]	2
Isoform 2 of 14 kDa phosphohistidine phosphatase OS=Homo sapiens GN=PHPT1 - [PHP14_HUMAN]	2
Isoform 5 of Neuropathy target esterase OS=Homo sapiens GN=PNPLA6 - [PLPL6_HUMAN]	2
Protein O-linked-mannose beta-1,4-N-acetylglucosaminyltransferase 2 OS=Homo sapiens GN=POMGNT2 PE=1 SV=1 - [PMGT2_HUMAN]	2
Peptidyl-prolyl cis-trans isomerase-like 1 OS=Homo sapiens GN=PPIL1 PE=1 SV=1 - [PPIL1_HUMAN]	2
Peroxiredoxin-2 OS=Homo sapiens GN=PRDX2 PE=1 SV=5 - [PRDX2_HUMAN]	2
Peroxiredoxin-6 OS=Homo sapiens GN=PRDX6 PE=1 SV=3 - [PRDX6_HUMAN]	2
Proline-rich transmembrane protein 2 OS=Homo sapiens GN=PRRT2 PE=1 SV=1 - [PRRT2_HUMAN]	2
Isoform 5 of Polypyrimidine tract-binding protein 2 OS=Homo sapiens GN=PTBP2 - [PTBP2_HUMAN]	2
Isoform 1 of Polypyrimidine tract-binding protein 3 OS=Homo sapiens GN=PTBP3 - [PTBP3_HUMAN]	2
Prostaglandin-H2 D-isomerase OS=Homo sapiens GN=PTGDS PE=1 SV=1 - [PTGDS_HUMAN]	2
Isoform 3 of Receptor-type tyrosine-protein phosphatase delta OS=Homo sapiens GN=PTPRD - [PTPRD_HUMAN]	2
Putative peptidyl-tRNA hydrolase PTRHD1 OS=Homo sapiens GN=PTRHD1 PE=1 SV=1 - [PTRD1_HUMAN]	2
Isoform 2 of Peroxisomal membrane protein 11B OS=Homo sapiens GN=PEX11B - [PX11B_HUMAN]	2
Ras-related protein Rab-12 OS=Homo sapiens GN=RAB12 PE=1 SV=3 - [RAB12_HUMAN]	2
Ras-related protein Rab-31 OS=Homo sapiens GN=RAB31 PE=1 SV=1 - [RAB31_HUMAN]	2
Ras-related protein Ral-A OS=Homo sapiens GN=RALA PE=1 SV=1 - [RALA_HUMAN]	2
Ras-related protein Rap-2a OS=Homo sapiens GN=RAP2A PE=1 SV=1 - [RAP2A_HUMAN]	2
GTPase NRas OS=Homo sapiens GN=NRAS PE=1 SV=1 - [RASN_HUMAN]	2
Ras-related protein Rab-39B OS=Homo sapiens GN=RAB39B PE=1 SV=1 - [RB39B_HUMAN]	2
Isoform 4 of Histone-binding protein RBBP4 OS=Homo sapiens GN=RBBP4 - [RBBP4_HUMAN]	2
Rab3 GTPase-activating protein non-catalytic subunit OS=Homo sapiens GN=RAB3GAP2 PE=1 SV=1 - [RBGPR_HUMAN]	2

RNA-binding protein 14 OS=Homo sapiens GN=RBM14 PE=1 SV=2 - [RBM14_HUMAN]	2
Rho-related GTP-binding protein RhoG OS=Homo sapiens GN=RHOG PE=1 SV=1 - [RHOG_HUMAN]	2
Rap guanine nucleotide exchange factor 2 OS=Homo sapiens GN=RAPGEF2 PE=1 SV=1 - [RPGF2_HUMAN]	2
40S ribosomal protein S18 OS=Homo sapiens GN=RPS18 PE=1 SV=3 - [RS18_HUMAN]	2
Isoform 3 of Reticulon-4 OS=Homo sapiens GN=RTN4 - [RTN4_HUMAN]	2
SUMO-activating enzyme subunit 2 OS=Homo sapiens GN=UBA2 PE=1 SV=2 - [SAE2_HUMAN]	2
Saccharopine dehydrogenase-like oxidoreductase OS=Homo sapiens GN=SCCPDH PE=1 SV=1 - [SCPDL_HUMAN]	2
Isoform 3 of Syntenin-1 OS=Homo sapiens GN=SDCBP - [SDCB1_HUMAN]	2
Isoform 2 of Phosphoserine aminotransferase OS=Homo sapiens GN=PSAT1 - [SERC_HUMAN]	2
Isoform 4 of NAD-dependent protein deacylase sirtuin-5, mitochondrial OS=Homo sapiens GN=SIRT5 - [SIR5_HUMAN]	2
Structural maintenance of chromosomes protein 3 OS=Homo sapiens GN=SMC3 PE=1 SV=2 - [SMC3_HUMAN]	2
Small nuclear ribonucleoprotein Sm D2 OS=Homo sapiens GN=SNRPD2 PE=1 SV=1 - [SMD2_HUMAN]	2
Syntaphilin OS=Homo sapiens GN=SNPH PE=1 SV=2 - [SNPH_HUMAN]	2
Isoform 2 of Sorting nexin-12 OS=Homo sapiens GN=SNX12 - [SNX12_HUMAN]	2
Isoform 2 of Sortilin OS=Homo sapiens GN=SORT1 - [SORT_HUMAN]	2
Probable leucine--tRNA ligase, mitochondrial OS=Homo sapiens GN=LARS2 PE=1 SV=2 - [SYLM_HUMAN]	2
Synaptotagmin-7 OS=Homo sapiens GN=SYT7 PE=1 SV=3 - [SYT7_HUMAN]	2
T-complex protein 11-like protein 1 OS=Homo sapiens GN=TCP11L1 PE=1 SV=1 - [T11L1_HUMAN]	2
Transmembrane protein 11, mitochondrial OS=Homo sapiens GN=TMEM11 PE=1 SV=1 - [TMM11_HUMAN]	2
Transmembrane protein 65 OS=Homo sapiens GN=TMEM65 PE=1 SV=2 - [TMM65_HUMAN]	2
Isoform 3 of Transportin-1 OS=Homo sapiens GN=TNPO1 - [TNPO1_HUMAN]	2
Tumor protein p63-regulated gene 1-like protein OS=Homo sapiens GN=TPRG1L PE=1 SV=1 - [TPRGL_HUMAN]	2
Ufm1-specific protease 2 OS=Homo sapiens GN=UFSP2 PE=1 SV=3 - [UFSP2_HUMAN]	2
General vesicular transport factor p115 OS=Homo sapiens GN=USO1 PE=1 SV=2 - [USO1_HUMAN]	2
Synaptogyrin-1 OS=Homo sapiens GN=SYNGR1 PE=1 SV=1 - [B5MCD7_HUMAN]	2
Isoform 2 of Ephrin type-A receptor 4 OS=Homo sapiens GN=EPHA4 - [EPHA4_HUMAN]	2
Isoform 2 of Aldo-keto reductase family 1 member C3 OS=Homo sapiens GN=AKR1C3 - [AK1C3_HUMAN]	2
Isoform 2 of Methionine--tRNA ligase, cytoplasmic OS=Homo sapiens GN=MARS - [SYMC_HUMAN]	2
Isoform 3 of ATP-citrate synthase OS=Homo sapiens GN=ACLY - [ACLY_HUMAN]	2
Isoform 2 of Dihydrolipoyl dehydrogenase, mitochondrial OS=Homo sapiens GN=DLD - [DLDH_HUMAN]	2
FSD1-like protein OS=Homo sapiens GN=FSD1L PE=1 SV=1 - [Q8N450_HUMAN]	2
Isoform 2 of Small nuclear ribonucleoprotein Sm D3 OS=Homo sapiens GN=SNRPD3 - [SMD3_HUMAN]	2
F-actin-capping protein subunit beta OS=Homo sapiens GN=CAPZB PE=1 SV=1 - [B1AK85_HUMAN]	2
Isoform 2 of SPARC-like protein 1 OS=Homo sapiens GN=SPARCL1 - [SPRL1_HUMAN]	2
High mobility group protein B1 OS=Homo sapiens GN=HMGB1 PE=1 SV=1 - [Q5T7C4_HUMAN]	2
COP9 signalosome complex subunit 1 OS=Homo sapiens GN=GPS1 PE=1 SV=2 - [C9JFE4_HUMAN]	2
Aggrecan OS=Homo sapiens GN=ACAN PE=1 SV=1 - [Q6PID9_HUMAN]	2
60S ribosomal protein L27a OS=Homo sapiens GN=RPL27A PE=1 SV=1 - [E9PJD9_HUMAN]	2
Flotillin-2 OS=Homo sapiens GN=FLOT2 PE=1 SV=1 - [J3QLD9_HUMAN]	2
Heat shock protein 75 kDa, mitochondrial OS=Homo sapiens GN=TRAP1 PE=1 SV=1 - [I3L0K7_HUMAN]	2
Methylcrotonoyl-CoA carboxylase subunit alpha, mitochondrial OS=Homo sapiens GN=MCCC1 PE=1 SV=1 - [G5E9X5_HUMAN]	2
60S ribosomal protein L4 OS=Homo sapiens GN=RPL4 PE=1 SV=1 - [H3BM89_HUMAN]	2
Profilin 1, isoform CRA_b OS=Homo sapiens GN=PFN1 PE=1 SV=1 - [K7EJ44_HUMAN]	2
Vesicle-associated membrane protein 2 OS=Homo sapiens GN=VAMP2 PE=4 SV=2 - [J3QRU4_HUMAN]	2
Islet cell autoantigen 1 (Fragment) OS=Homo sapiens GN=ICA1 PE=1 SV=1 - [C9J3Y4_HUMAN]	2
Eukaryotic translation elongation factor 1 epsilon-1 (Fragment) OS=Homo sapiens GN=EEF1E1 PE=4 SV=1 - [D6RCQ0_HUMAN]	2
RNA-binding protein 4B OS=Homo sapiens GN=RBM4B PE=1 SV=1 - [E9PLB0_HUMAN]	2
Phosphoinositide phospholipase C (Fragment) OS=Homo sapiens GN=PLCB1 PE=1 SV=1 - [H0YCJ2_HUMAN]	2
Ubiquitin carboxyl-terminal hydrolase (Fragment) OS=Homo sapiens GN=UCHL5 PE=1 SV=1 - [Q5LJB0_HUMAN]	2
Golgi-associated plant pathogenesis-related protein 1 OS=Homo sapiens GN=GLIPR2 PE=1 SV=1 - [Q5VZR0_HUMAN]	2
Rho-related GTP-binding protein RhoC (Fragment) OS=Homo sapiens GN=RHOC PE=1 SV=1 - [Q5JR07_HUMAN]	2
Isoform 2 of Stromal interaction molecule 1 OS=Homo sapiens GN=STIM1 - [STIM1_HUMAN]	2
60S ribosomal protein L9 (Fragment) OS=Homo sapiens GN=RPL9 PE=1 SV=1 - [E7ESE0_HUMAN]	2

Toll-interacting protein OS=Homo sapiens GN=TOLLIP PE=1 SV=1 - [F2Z2Y8_HUMAN]	2
Calpain small subunit 1 (Fragment) OS=Homo sapiens GN=CAPNS1 PE=1 SV=1 - [U3KPR7_HUMAN]	2
Serine/threonine-protein phosphatase (Fragment) OS=Homo sapiens GN=PPP3CA PE=1 SV=1 - [E9PPC8_HUMAN]	2
Caprin-1 (Fragment) OS=Homo sapiens GN=CAPRIN1 PE=1 SV=1 - [E9PLA9_HUMAN]	2
Metaxin-2 (Fragment) OS=Homo sapiens GN=MTX2 PE=1 SV=1 - [C9JAZ1_HUMAN]	2
Isoform 2 of Vacuolar protein sorting-associated protein VTA1 homolog OS=Homo sapiens GN=VTA1 - [VTA1_HUMAN]	2
Acyl-CoA synthetase family member 2, mitochondrial (Fragment) OS=Homo sapiens GN=ACSF2 PE=1 SV=1 - [D6RF87_HUMAN]	2
Single-stranded DNA-binding protein, mitochondrial (Fragment) OS=Homo sapiens GN=SSBP1 PE=1 SV=1 - [C9K0U8_HUMAN]	2
Methyltransferase-like protein 7A (Fragment) OS=Homo sapiens GN=METTL7A PE=1 SV=1 - [F8VQX6_HUMAN]	2
Acid ceramidase OS=Homo sapiens GN=ASAH1 PE=1 SV=1 - [E7EMM4_HUMAN]	2
60S ribosomal protein L8 OS=Homo sapiens GN=RPL8 PE=1 SV=1 - [E9PP36_HUMAN]	2
Ribose-phosphate pyrophosphokinase 1 OS=Homo sapiens GN=PRPS1 PE=1 SV=1 - [B1ALA9_HUMAN]	2
Tetraspanin OS=Homo sapiens GN=CD9 PE=1 SV=1 - [A6NNI4_HUMAN]	2
AP-3 complex subunit mu-2 (Fragment) OS=Homo sapiens GN=AP3M2 PE=1 SV=1 - [E5RJ52_HUMAN]	2
Ubiquitin-like modifier-activating enzyme 5 OS=Homo sapiens GN=UBA5 PE=1 SV=1 - [E7EWE1_HUMAN]	2
OCIA domain-containing protein 1 (Fragment) OS=Homo sapiens GN=OCIAD1 PE=1 SV=1 - [D6R918_HUMAN]	2
Protein quaking (Fragment) OS=Homo sapiens GN=QKI PE=1 SV=1 - [F5H8C8_HUMAN]	2
Serine/threonine-protein kinase OSR1 OS=Homo sapiens GN=OXSR1 PE=1 SV=1 - [C9JIG9_HUMAN]	2
Delta-1-pyrroline-5-carboxylate dehydrogenase, mitochondrial (Fragment) OS=Homo sapiens GN=ALDH4A1 PE=1 SV=1 - [Q5TF55_HUMAN]	2
60S ribosomal protein L30 (Fragment) OS=Homo sapiens GN=RPL30 PE=1 SV=1 - [E5RI99_HUMAN]	2
Phosphatidylinositide phosphatase SAC1 (Fragment) OS=Homo sapiens GN=SACM1L PE=1 SV=1 - [C9JV50_HUMAN]	2
Eukaryotic translation initiation factor 3 subunit E OS=Homo sapiens GN=EIF3E PE=1 SV=1 - [E5RGA2_HUMAN]	2
Exocyst complex component 3 OS=Homo sapiens GN=EXOC3 PE=1 SV=1 - [D6RB59_HUMAN]	2
Ly-6/neurotoxin-like protein 1 (Fragment) OS=Homo sapiens GN=LYNX1 PE=1 SV=1 - [A0A087WZS0_HUMAN]	2
DnaJ homolog subfamily B member 2 (Fragment) OS=Homo sapiens GN=DNAJB2 PE=1 SV=1 - [C9JRD2_HUMAN]	2
NudC domain-containing protein 2 OS=Homo sapiens GN=NUDCD2 PE=1 SV=1 - [E5RFP0_HUMAN]	2
Calmodulin OS=Homo sapiens GN=CALM2 PE=1 SV=1 - [F8WBR5_HUMAN]	2
Mitochondrial import inner membrane translocase subunit TIM50 OS=Homo sapiens GN=TIMM50 PE=1 SV=2 - [M0R2F8_HUMAN]	2
ADP-ribosylation factor GTPase-activating protein 1 (Fragment) OS=Homo sapiens GN=ARFGAP1 PE=1 SV=2 - [E5RHT6_HUMAN]	2
Prostamide/prostaglandin F synthase (Fragment) OS=Homo sapiens GN=FAM213B PE=1 SV=1 - [J3QKK8_HUMAN]	2
ATPase ASNA1 (Fragment) OS=Homo sapiens GN=ASNA1 PE=1 SV=1 - [K7ERW9_HUMAN]	2
40S ribosomal protein S16 OS=Homo sapiens GN=RPS16 PE=1 SV=1 - [M0R3H0_HUMAN]	2
Probable glutamate--tRNA ligase, mitochondrial OS=Homo sapiens GN=EARS2 PE=1 SV=1 - [H3BTB7_HUMAN]	2
UBX domain-containing protein 6 (Fragment) OS=Homo sapiens GN=UBXN6 PE=1 SV=1 - [K7EP32_HUMAN]	2
Signal transducer and activator of transcription OS=Homo sapiens GN=STAT3 PE=1 SV=1 - [G8JLH9_HUMAN]	2
Guanine nucleotide-binding protein subunit beta-2-like 1 (Fragment) OS=Homo sapiens GN=GNB2L1 PE=1 SV=1 - [H0YAF8_HUMAN]	2
GTP-binding protein Di-Ras1 (Fragment) OS=Homo sapiens GN=DIRAS1 PE=1 SV=1 - [K7EN06_HUMAN]	2
40S ribosomal protein S2 (Fragment) OS=Homo sapiens GN=RPS2 PE=1 SV=1 - [H0YEN5_HUMAN]	2
Rho GTPase-activating protein 1 (Fragment) OS=Homo sapiens GN=ARHGAP1 PE=1 SV=1 - [H0YE29_HUMAN]	2
Calcium-binding mitochondrial carrier protein SCaMC-3 (Fragment) OS=Homo sapiens GN=SLC25A23 PE=1 SV=1 - [M0QZJ5_HUMAN]	2
Electron transfer flavoprotein subunit alpha, mitochondrial OS=Homo sapiens GN=ETFA PE=1 SV=1 - [H0YK49_HUMAN]	2
DnaJ homolog subfamily B member 1 (Fragment) OS=Homo sapiens GN=DNAJB1 PE=1 SV=1 - [M0R1D6_HUMAN]	2
Serum paraoxonase/arylesterase 2 OS=Homo sapiens GN=PON2 PE=1 SV=1 - [J3QT77_HUMAN]	2
40S ribosomal protein S5 (Fragment) OS=Homo sapiens GN=RPS5 PE=1 SV=1 - [M0R0F0_HUMAN]	2
Dystrophin (Fragment) OS=Homo sapiens GN=DMD PE=1 SV=1 - [H0Y3E8_HUMAN]	2
V-type proton ATPase subunit d 1 (Fragment) OS=Homo sapiens GN=ATP6V0D1 PE=1 SV=1 - [J3QL14_HUMAN]	2
Acyl-CoA synthetase family member 3, mitochondrial (Fragment) OS=Homo sapiens GN=ACSF3 PE=1 SV=1 - [H0YGC7_HUMAN]	2
26S proteasome non-ATPase regulatory subunit 8 OS=Homo sapiens GN=PSMD8 PE=1 SV=1 - [K7EJC1_HUMAN]	2
Microtubule-associated protein 1S (Fragment) OS=Homo sapiens GN=MAP1S PE=1 SV=1 - [M0R1M7_HUMAN]	2
Serine hydroxymethyltransferase (Fragment) OS=Homo sapiens GN=SHMT2 PE=1 SV=1 - [G3V2Y4_HUMAN]	2
Alpha-centractin OS=Homo sapiens GN=ACTR1A PE=1 SV=1 - [R4GMT0_HUMAN]	2
Casein kinase II subunit alpha' (Fragment) OS=Homo sapiens GN=CSNK2A2 PE=1 SV=2 - [H3BSA1_HUMAN]	2
Receptor-type tyrosine-protein phosphatase F (Fragment) OS=Homo sapiens GN=PTPRF PE=1 SV=1 - [H0Y380_HUMAN]	2

Lon protease homolog, mitochondrial (Fragment) OS=Homo sapiens GN=LONP1 PE=1 SV=1 - [K7ER27_HUMAN]	2
Signal recognition particle receptor subunit beta (Fragment) OS=Homo sapiens GN=SRPRB PE=1 SV=1 - [H7C4H2_HUMAN]	2
60S ribosomal protein L18a (Fragment) OS=Homo sapiens GN=RPL18A PE=1 SV=1 - [M0R3D6_HUMAN]	2
Pyruvate dehydrogenase protein X component, mitochondrial (Fragment) OS=Homo sapiens GN=PDHX PE=1 SV=1 - [H0YD97_HUMAN]	2
DmX-like protein 2 (Fragment) OS=Homo sapiens GN=DMXL2 PE=1 SV=1 - [H0YLM8_HUMAN]	2
Signal recognition particle subunit SRP72 OS=Homo sapiens GN=SRP72 PE=1 SV=1 - [R4GNC1_HUMAN]	2
Glutathione peroxidase (Fragment) OS=Homo sapiens GN=GPX4 PE=1 SV=1 - [K7ERP4_HUMAN]	2
Isoform 5 of Cell adhesion molecule 2 OS=Homo sapiens GN=CADM2 - [CADM2_HUMAN]	2
Isoform 3 of Programmed cell death 6-interacting protein OS=Homo sapiens GN=PDCD6IP - [PDC6I_HUMAN]	2
Isoform 2 of Alpha-1-syntrophin OS=Homo sapiens GN=SNTA1 - [SNTA1_HUMAN]	2
Vigilin (Fragment) OS=Homo sapiens GN=HDLBP PE=1 SV=4 - [H7C0A4_HUMAN]	2
ADP-ribosylation factor-like protein 15 OS=Homo sapiens GN=ARL15 PE=1 SV=1 - [A0A087WUW9_HUMAN]	2
Laminin subunit alpha-2 OS=Homo sapiens GN=LAMA2 PE=1 SV=1 - [A0A087WYF1_HUMAN]	2
Complement C1q subcomponent subunit B (Fragment) OS=Homo sapiens GN=C1QB PE=1 SV=2 - [A0A0A0MSV6_HUMAN]	2
Amino acid transporter OS=Homo sapiens GN=SLC1A3 PE=1 SV=1 - [A0A087X0U3_HUMAN]	2
Cytoplasmic dynein 1 intermediate chain 1 OS=Homo sapiens GN=DYNC1I1 PE=1 SV=1 - [A0A0A0MTG2_HUMAN]	2
Proline dehydrogenase 1, mitochondrial OS=Homo sapiens GN=PRODH PE=1 SV=1 - [A0A087WWM6_HUMAN]	2
Opalin OS=Homo sapiens GN=OPALIN PE=1 SV=1 - [A0A0A0MTN4_HUMAN]	2
Protein LOC102724023 OS=Homo sapiens GN=LOC102724023 PE=4 SV=1 - [A0A096LNH5_HUMAN]	2
Neuronal growth regulator 1 OS=Homo sapiens GN=NEGR1 PE=1 SV=1 - [F6X2W2_HUMAN]	2
AH receptor-interacting protein (Fragment) OS=Homo sapiens GN=AIP PE=1 SV=2 - [E9PMH2_HUMAN]	2
TAR DNA-binding protein 43 (Fragment) OS=Homo sapiens GN=TARDBP PE=1 SV=1 - [A0A087WXQ5_HUMAN]	2
Double-stranded RNA-binding protein Staufen homolog 1 OS=Homo sapiens GN=STAU1 PE=1 SV=1 - [A0A087X1A5_HUMAN]	2
Neuronal cell adhesion molecule OS=Homo sapiens GN=NRCAM PE=1 SV=1 - [A0A087X2B3_HUMAN]	2
Myristoylated alanine-rich C-kinase substrate OS=Homo sapiens GN=MARCKS PE=1 SV=1 - [A0A087WZH7_HUMAN]	2
Uncharacterized protein C6orf136 (Fragment) OS=Homo sapiens GN=C6orf136 PE=1 SV=2 - [H7C5D3_HUMAN]	2
Cullin-associated NEDD8-dissociated protein 1 (Fragment) OS=Homo sapiens GN=CAND1 PE=4 SV=1 - [A0A0C4DGH5_HUMAN]	2

Supplementary Table III. Comparison of fungal peptides in P2 and P7 fractions in AD1.

GENE ID P2	GENE ID P7	GENE NAME GENE SYMBOL ORTHOLOG	PANTHER FAMILY/SUBFAMILY
**calcium-binding protein (PC00060)**			
HUMAN|HGNC=9060|UniProtKB=P51178		1-phosphatidylinositol 4,5-bisphosphate phosphodiesterase delta-1;PLCD1;ortholog	1-PHOSPHATIDYLINOSITOL 4,5-BISPHOSPHATE PHOSPHODIESTERASE DELTA-1 (PTHR10336:SF126)
HUMAN|HGNC=9061|UniProtKB=Q8N3E9		1-phosphatidylinositol 4,5-bisphosphate phosphodiesterase delta-3;PLCD3;ortholog	1-PHOSPHATIDYLINOSITOL 4,5-BISPHOSPHATE PHOSPHODIESTERASE DELTA-3 (PTHR10336:SF141)
HUMAN|HGNC=10990|UniProtKB=P12235	HUMAN|HGNC=10990|UniProtKB=P12235	ADP/ATP translocase 1;SLC25A4;ortholog	ADP/ATP TRANSLOCASE 1 (PTHR24089:SF506)
HUMAN|HGNC=10991|UniProtKB=P05141	HUMAN|HGNC=10991|UniProtKB=P05141	ADP/ATP translocase 2;SLC25A5;ortholog	ADP/ATP TRANSLOCASE 2 (PTHR24089:SF446)
HUMAN|HGNC=10992|UniProtKB=P12236	HUMAN|HGNC=10992|UniProtKB=P12236	ADP/ATP translocase 3;SLC25A6;ortholog	ADP/ATP TRANSLOCASE 3 (PTHR24089:SF411)
HUMAN|HGNC=10982|UniProtKB=O75746	HUMAN|HGNC=10982|UniProtKB=O75746	Calcium-binding mitochondrial carrier protein Aralar1;SLC25A12;ortholog	CALCIUM-BINDING MITOCHONDRIAL CARRIER PROTEIN ARALAR1 (PTHR24089:SF503)
HUMAN|HGNC=1442|UniProtKB=P62158	HUMAN|HGNC=1442|UniProtKB=P62158	Calmodulin;CALM1;ortholog	CALMODULIN (PTHR23050:SF234)
HUMAN|HGNC=1445|UniProtKB=P62158	HUMAN|HGNC=1445|UniProtKB=P62158	Calmodulin;CALM1;ortholog	CALMODULIN (PTHR23050:SF239)
HUMAN|HGNC=1449|UniProtKB=P62158	HUMAN|HGNC=1449|UniProtKB=P62158	Calmodulin;CALM1;ortholog	CALMODULIN (PTHR23050:SF245)

HUMAN|HGNC=1473|UniProtKB=P27824		Calnexin;CANX;ortholog	CALNEXIN (PTHR11073:SF29)
HUMAN|HGNC=1476|UniProtKB=P07384		Calpain-1 catalytic subunit;CAPN1;ortholog	CALPAIN-1 CATALYTIC SUBUNIT (PTHR10183:SF343)
HUMAN|HGNC=1455|UniProtKB=P27797		Calreticulin;CALR;ortholog	CALRETICULIN (PTHR11073:SF21)
HUMAN|HGNC=3244|UniProtKB=Q9NZN3	HUMAN|HGNC=3244|UniProtKB=Q9NZN3	EH domain-containing protein 3;EHD3;ortholog	EH DOMAIN-CONTAINING PROTEIN 3 (PTHR11216:SF88)
HUMAN|HGNC=6556|UniProtKB=O95202	HUMAN|HGNC=6556|UniProtKB=O95202	LETM1 and EF-hand domain-containing protein 1, mitochondrial;LETM1;ortholog	LETM1 AND EF-HAND DOMAIN-CONTAINING PROTEIN 1, MITOCHONDRIAL (PTHR14009:SF18)
HUMAN|HGNC=19954|UniProtKB=Q9H936	HUMAN|HGNC=19954|UniProtKB=Q9H936	Mitochondrial glutamate carrier 1;SLC25A22;ortholog	MITOCHONDRIAL GLUTAMATE CARRIER 1 (PTHR24089:SF598)
	HUMAN|HGNC=10988|UniProtKB=Q9H1K4	Mitochondrial glutamate carrier 2;SLC25A18;ortholog	MITOCHONDRIAL GLUTAMATE CARRIER 2 (PTHR24089:SF482)
	HUMAN|HGNC=16701|UniProtKB=P19105	Myosin regulatory light chain 12A;MYL12A;ortholog	MYOSIN REGULATORY LIGHT CHAIN 12A (PTHR23049:SF55)
HUMAN|HGNC=30009|UniProtKB=Q9UBV8		Peflin;PEF1;ortholog	PEFLIN (PTHR10183:SF361)
HUMAN|HGNC=3718|UniProtKB=P26885		Peptidyl-prolyl cis-trans isomerase FKBP2;FKBP2;ortholog	PEPTIDYL-PROLYL CIS-TRANS ISOMERASE FKBP2 (PTHR10516:SF348)
HUMAN|HGNC=3719|UniProtKB=Q00688		Peptidyl-prolyl cis-trans isomerase FKBP3;FKBP3;ortholog	PEPTIDYL-PROLYL CIS-TRANS ISOMERASE FKBP3 (PTHR10516:SF356)
HUMAN|HGNC=3720|UniProtKB=Q02790		Peptidyl-prolyl cis-trans isomerase FKBP4;FKBP4;ortholog	PEPTIDYL-PROLYL CIS-TRANS ISOMERASE FKBP4 (PTHR10516:SF332)
HUMAN|HGNC=3724|UniProtKB=Q14318		Peptidyl-prolyl cis-trans isomerase FKBP8;FKBP8;ortholog	PEPTIDYL-PROLYL CIS-TRANS ISOMERASE FKBP8 (PTHR10516:SF363)
HUMAN|HGNC=8765|UniProtKB=O75340		Programmed cell death protein 6;PDCD6;ortholog	PROGRAMMED CELL DEATH PROTEIN 6 (PTHR10183:SF367)
HUMAN|HGNC=29180|UniProtKB=Q9UPV7	HUMAN|HGNC=29180|UniProtKB=Q9UPV7	Protein KIAA1045;KIAA1045;ortholog	PROTEIN KIAA1045 (PTHR10891:SF706)
HUMAN|HGNC=9393|UniProtKB=P17252	HUMAN|HGNC=9393|UniProtKB=P17252	Protein kinase C alpha type;PRKCA;ortholog	PROTEIN KINASE C ALPHA TYPE (PTHR24356:SF270)
	HUMAN|HGNC=9395|UniProtKB=P05771	Protein kinase C beta type;PRKCB;ortholog	PROTEIN KINASE C BETA TYPE (PTHR24356:SF244)
	HUMAN|HGNC=9401|UniProtKB=Q02156	Protein kinase C epsilon type;PRKCE;ortholog	PROTEIN KINASE C EPSILON TYPE (PTHR24356:SF275)
	HUMAN|HGNC=9402|UniProtKB=P05129	Protein kinase C gamma type;PRKCG;ortholog	PROTEIN KINASE C GAMMA TYPE (PTHR24356:SF293)
	HUMAN|HGNC=10490|UniProtKB=Q99584	Protein S100-A13;S100A13;ortholog	PROTEIN S100-A13 (PTHR11639:SF98)
	HUMAN|HGNC=10498|UniProtKB=P05109	Protein S100-A8;S100A8;ortholog	PROTEIN S100-A8 (PTHR11639:SF111)
	HUMAN|HGNC=10499|UniProtKB=P06702	Protein S100-A9;S100A9;ortholog	PROTEIN S100-A9 (PTHR11639:SF110)
	HUMAN|HGNC=49897|UniProtKB=Q6A1A2	Putative 3-phosphoinositide-dependent protein kinase 2;PDPK2P;ortholog	3-PHOSPHOINOSITIDE-DEPENDENT PROTEIN KINASE 1-RELATED (PTHR24356:SF290)
	HUMAN|HGNC=10432|UniProtKB=P51812	Ribosomal protein S6 kinase alpha-3;RPS6KA3;ortholog	RIBOSOMAL PROTEIN S6 KINASE ALPHA-3 (PTHR24351:SF133)
	HUMAN|HGNC=21332|UniProtKB=Q86UX6	Serine/threonine-protein kinase 32C;STK32C;ortholog	SERINE/THREONINE-PROTEIN KINASE 32C (PTHR24356:SF232)
	HUMAN|HGNC=17848|UniProtKB=Q9Y2H1	Serine/threonine-protein kinase 38-like;STK38L;ortholog	SERINE/THREONINE-PROTEIN KINASE 38-LIKE (PTHR24356:SF259)
HUMAN|HGNC=9300|UniProtKB=P62714	HUMAN|HGNC=9300|UniProtKB=P62714	Serine/threonine-protein phosphatase 2A catalytic subunit beta isoform;PPP2CB;ortholog	SERINE/THREONINE-PROTEIN PHOSPHATASE 2A CATALYTIC SUBUNIT BETA ISOFORM (PTHR11668:SF343)
	HUMAN|HGNC=9281|UniProtKB=P62136	Serine/threonine-protein phosphatase PP1-alpha catalytic subunit;PPP1CA;ortholog	SERINE/THREONINE-PROTEIN PHOSPHATASE PP1-ALPHA CATALYTIC SUBUNIT (PTHR11668:SF365)

HUMAN|HGNC=9282|UniProtKB=P62140	HUMAN|HGNC=9282|UniProtKB=P62140	Serine/threonine-protein phosphatase PP1-beta catalytic subunit;PPP1CB;ortholog	SERINE/THREONINE-PROTEIN PHOSPHATASE PP1-BETA CATALYTIC SUBUNIT (PTHR11668:SF346)
	HUMAN|HGNC=26395|UniProtKB=Q8N6N2	Tetratricopeptide repeat protein 9B;TTC9B;ortholog	TETRATRICOPEPTIDE REPEAT PROTEIN 9B (PTHR43811:SF4)
HUMAN|HGNC=10979|UniProtKB=P53007	HUMAN|HGNC=10979|UniProtKB=P53007	Tricarboxylate transport protein, mitochondrial;SLC25A1;ortholog	TRICARBOXYLATE TRANSPORT PROTEIN, MITOCHONDRIAL (PTHR24089:SF459)
**cell adhesion molecule (PC00069)**			
HUMAN|HGNC=6934|UniProtKB=P43121		Cell surface glycoprotein MUC18;MCAM;ortholog	CELL SURFACE GLYCOPROTEIN MUC18 (PTHR11973:SF21)
HUMAN|HGNC=2172|UniProtKB=Q02246		Contactin-2;CNTN2;ortholog	CONTACTIN-2 (PTHR10489:SF882)
HUMAN|HGNC=8011|UniProtKB=P78357	HUMAN|HGNC=8011|UniProtKB=P78357	Contactin-associated protein 1;CNTNAP1;ortholog	CONTACTIN-ASSOCIATED PROTEIN 1 (PTHR10127:SF740)
HUMAN|HGNC=6561|UniProtKB=P09382		Galectin-1;LGALS1;ortholog	GALECTIN-1 (PTHR11346:SF131)
HUMAN|HGNC=26361|UniProtKB=Q14CZ8		Hepatocyte cell adhesion molecule;HEPACAM;ortholog	HEPATOCYTE CELL ADHESION MOLECULE (PTHR12080:SF66)
HUMAN|HGNC=28246|UniProtKB=Q96ID5		Immunoglobulin superfamily member 21;IGSF21;ortholog	IMMUNOGLOBULIN SUPERFAMILY MEMBER 21 (PTHR10489:SF771)
HUMAN|HGNC=5348|UniProtKB=Q9UMF0	HUMAN|HGNC=5348|UniProtKB=Q9UMF0	Intercellular adhesion molecule 5;ICAM5;ortholog	INTERCELLULAR ADHESION MOLECULE 5 (PTHR13771:SF15)
HUMAN|HGNC=7656|UniProtKB=P13591		Neural cell adhesion molecule 1;NCAM1;ortholog	NEURAL CELL ADHESION MOLECULE 1 (PTHR10489:SF783)
	HUMAN|HGNC=17787|UniProtKB=O14910	Protein lin-7 homolog A;LIN7A;ortholog	PROTEIN LIN-7 HOMOLOG A (PTHR14063:SF10)
	HUMAN|HGNC=10589|UniProtKB=O60939	Sodium channel subunit beta-2;SCN2B;ortholog	SODIUM CHANNEL SUBUNIT BETA-2 (PTHR13869:SF34)
HUMAN|HGNC=11845|UniProtKB=Q9Y490	HUMAN|HGNC=11845|UniProtKB=Q9Y490	Talin-1;TLN1;ortholog	TALIN-1 (PTHR19981:SF24)
HUMAN|HGNC=15447|UniProtKB=Q9Y4G6	HUMAN|HGNC=15447|UniProtKB=Q9Y4G6	Talin-2;TLN2;ortholog	TALIN-2 (PTHR19981:SF28)
	HUMAN|HGNC=12724|UniProtKB=P04004	Vitronectin;VTN;ortholog	VITRONECTIN (PTHR22917:SF7)
**cell junction protein (PC00070)**			
HUMAN|HGNC=8514|UniProtKB=O75508	HUMAN|HGNC=8514|UniProtKB=O75508	Claudin-11;CLDN11;ortholog	CLAUDIN-11 (PTHR12002:SF153)
HUMAN|HGNC=4274|UniProtKB=P17302	HUMAN|HGNC=4274|UniProtKB=P17302	Gap junction alpha-1 protein;GJA1;ortholog	GAP JUNCTION ALPHA-1 PROTEIN (PTHR11984:SF70)
	HUMAN|HGNC=7568|UniProtKB=P35580	Myosin-10;MYH10;ortholog	MYOSIN-10 (PTHR13140:SF549)
	HUMAN|HGNC=17787|UniProtKB=O14910	Protein lin-7 homolog A;LIN7A;ortholog	PROTEIN LIN-7 HOMOLOG A (PTHR14063:SF10)
**chaperone (PC00072)**			
HUMAN|HGNC=5269|UniProtKB=P61604		10 kDa heat shock protein, mitochondrial;HSPE1;ortholog	10 KDA HEAT SHOCK PROTEIN, MITOCHONDRIAL (PTHR10772:SF21)
HUMAN|HGNC=12851|UniProtKB=P62258	HUMAN|HGNC=12851|UniProtKB=P62258	14-3-3 protein epsilon;YWHAE;ortholog	14-3-3 PROTEIN EPSILON (PTHR18860:SF47)
HUMAN|HGNC=12853|UniProtKB=Q04917	HUMAN|HGNC=12853|UniProtKB=Q04917	14-3-3 protein eta;YWHAH;ortholog	14-3-3 PROTEIN ETA (PTHR18860:SF42)
HUMAN|HGNC=12852|UniProtKB=P61981	HUMAN|HGNC=12852|UniProtKB=P61981	14-3-3 protein gamma;YWHAG;ortholog	14-3-3 PROTEIN GAMMA (PTHR18860:SF41)
HUMAN|HGNC=12854|UniProtKB=P27348	HUMAN|HGNC=12854|UniProtKB=P27348	14-3-3 protein theta;YWHAQ;ortholog	14-3-3 PROTEIN THETA (PTHR18860:SF44)
HUMAN|HGNC=12855|UniProtKB=P63104	HUMAN|HGNC=12855|UniProtKB=P63104	14-3-3 protein zeta/delta;YWHAZ;ortholog	14-3-3 PROTEIN ZETA/DELTA (PTHR18860:SF45)
HUMAN|HGNC=5261|UniProtKB=P10809	HUMAN|HGNC=5261|UniProtKB=P10809	60 kDa heat shock protein, mitochondrial;HSPD1;ortholog	60 KDA HEAT SHOCK PROTEIN, MITOCHONDRIAL (PTHR11353:SF118)

	HUMAN|HGNC=358|UniProtKB=O00170	AH receptor-interacting protein;AIP;ortholog	AH RECEPTOR-INTERACTING PROTEIN (PTHR11242:SF6)
HUMAN|HGNC=11140|UniProtKB=Q16143		Beta-synuclein;SNCB;ortholog	BETA-SYNUCLEIN (PTHR13820:SF13)
HUMAN|HGNC=1473|UniProtKB=P27824		Calnexin;CANX;ortholog	CALNEXIN (PTHR11073:SF29)
	HUMAN|HGNC=2586|UniProtKB=Q9UDT6	CAP-Gly domain-containing linker protein 2;CLIP2;ortholog	CAP-GLY DOMAIN-CONTAINING LINKER PROTEIN 2 (PTHR18916:SF45)
HUMAN|HGNC=12028|UniProtKB=P14625		Endoplasmin;HSP90B1;ortholog	ENDOPLASMIN-RELATED (PTHR11528:SF66)
HUMAN|HGNC=19022|UniProtKB=O43301	HUMAN|HGNC=19022|UniProtKB=O43301	Heat shock 70 kDa protein 12A;HSPA12A;ortholog	HEAT SHOCK 70 KDA PROTEIN 12A (PTHR14187:SF63)
HUMAN|HGNC=5246|UniProtKB=P04792	HUMAN|HGNC=5246|UniProtKB=P04792	Heat shock protein beta-1;HSPB1;ortholog	HEAT SHOCK PROTEIN BETA-1 (PTHR11527:SF235)
HUMAN|HGNC=5253|UniProtKB=P07900	HUMAN|HGNC=5253|UniProtKB=P07900	Heat shock protein HSP 90-alpha;HSP90AA1;ortholog	HEAT SHOCK PROTEIN HSP 90-ALPHA-RELATED (PTHR11528:SF63)
HUMAN|HGNC=5258|UniProtKB=P08238	HUMAN|HGNC=5258|UniProtKB=P08238	Heat shock protein HSP 90-beta;HSP90AB1;ortholog	HEAT SHOCK PROTEIN HSP 90-BETA-RELATED (PTHR11528:SF67)
HUMAN|HGNC=11985|UniProtKB=O94826	HUMAN|HGNC=11985|UniProtKB=O94826	Mitochondrial import receptor subunit TOM70;TOMM70A;ortholog	MITOCHONDRIAL IMPORT RECEPTOR SUBUNIT TOM70 (PTHR22904:SF476)
HUMAN|HGNC=3718|UniProtKB=P26885		Peptidyl-prolyl cis-trans isomerase FKBP2;FKBP2;ortholog	PEPTIDYL-PROLYL CIS-TRANS ISOMERASE FKBP2 (PTHR10516:SF348)
HUMAN|HGNC=3719|UniProtKB=Q00688		Peptidyl-prolyl cis-trans isomerase FKBP3;FKBP3;ortholog	PEPTIDYL-PROLYL CIS-TRANS ISOMERASE FKBP3 (PTHR10516:SF356)
HUMAN|HGNC=3720|UniProtKB=Q02790		Peptidyl-prolyl cis-trans isomerase FKBP4;FKBP4;ortholog	PEPTIDYL-PROLYL CIS-TRANS ISOMERASE FKBP4 (PTHR10516:SF332)
HUMAN|HGNC=3724|UniProtKB=Q14318		Peptidyl-prolyl cis-trans isomerase FKBP8;FKBP8;ortholog	PEPTIDYL-PROLYL CIS-TRANS ISOMERASE FKBP8 (PTHR10516:SF363)
	HUMAN|HGNC=9476|UniProtKB=Q92743	Serine protease HTRA1;HTRA1;ortholog	SERINE PROTEASE HTRA1 (PTHR22939:SF107)
HUMAN|HGNC=10819|UniProtKB=O43765		Small glutamine-rich tetratricopeptide repeat-containing protein alpha;SGTA;ortholog	SMALL GLUTAMINE-RICH TETRATRICOPEPTIDE REPEAT-CONTAINING PROTEIN ALPHA (PTHR22904:SF475)
HUMAN|HGNC=11655|UniProtKB=P17987		T-complex protein 1 subunit alpha;TCP1;ortholog	T-COMPLEX PROTEIN 1 SUBUNIT ALPHA (PTHR11353:SF145)
HUMAN|HGNC=1615|UniProtKB=P78371		T-complex protein 1 subunit beta;CCT2;ortholog	T-COMPLEX PROTEIN 1 SUBUNIT BETA (PTHR11353:SF119)
HUMAN|HGNC=1617|UniProtKB=P50991	HUMAN|HGNC=1617|UniProtKB=P50991	T-complex protein 1 subunit delta;CCT4;ortholog	T-COMPLEX PROTEIN 1 SUBUNIT DELTA (PTHR11353:SF146)
HUMAN|HGNC=1618|UniProtKB=P48643		T-complex protein 1 subunit epsilon;CCT5;ortholog	T-COMPLEX PROTEIN 1 SUBUNIT EPSILON (PTHR11353:SF156)
HUMAN|HGNC=1623|UniProtKB=P50990	HUMAN|HGNC=1623|UniProtKB=P50990	T-complex protein 1 subunit theta;CCT8;ortholog	T-COMPLEX PROTEIN 1 SUBUNIT THETA (PTHR11353:SF133)
HUMAN|HGNC=1620|UniProtKB=P40227	HUMAN|HGNC=1620|UniProtKB=P40227	T-complex protein 1 subunit zeta;CCT6A;ortholog	T-COMPLEX PROTEIN 1 SUBUNIT ZETA (PTHR11353:SF140)
	HUMAN|HGNC=26395|UniProtKB=Q8N6N2	Tetratricopeptide repeat protein 9B;TTC9B;ortholog	TETRATRICOPEPTIDE REPEAT PROTEIN 9B (PTHR43811:SF4)
	HUMAN|HGNC=11581|UniProtKB=Q9BTW9	Tubulin-specific chaperone D;TBCD;ortholog	TUBULIN-SPECIFIC CHAPERONE D (PTHR12658:SF3)
**cytoskeletal protein (PC00085)**			
	HUMAN|HGNC=160|UniProtKB=O94805	Actin-like protein 6B;ACTL6B;ortholog	ACTIN-LIKE PROTEIN 6B (PTHR11937:SF266)
HUMAN|HGNC=706|UniProtKB=O15145	HUMAN|HGNC=706|UniProtKB=O15145	Actin-related protein 2/3 complex subunit 3;ARPC3;ortholog	ACTIN-RELATED PROTEIN 2/3 COMPLEX SUBUNIT 3 (PTHR12391:SF1)

HUMAN|HGNC=130|UniProtKB=P62736	HUMAN|HGNC=130|UniProtKB=P62736	Actin, aortic smooth muscle;ACTA2;ortholog	ACTIN, AORTIC SMOOTH MUSCLE (PTHR11937:SF346)
HUMAN|HGNC=132|UniProtKB=P60709	HUMAN|HGNC=132|UniProtKB=P60709	Actin, cytoplasmic 1;ACTB;ortholog	ACTIN, CYTOPLASMIC 1 (PTHR11937:SF288)
HUMAN|HGNC=164|UniProtKB=P35609	HUMAN|HGNC=164|UniProtKB=P35609	Alpha-actinin-2;ACTN2;ortholog	ALPHA-ACTININ-2 (PTHR11915:SF393)
HUMAN|HGNC=165|UniProtKB=Q08043		Alpha-actinin-3;ACTN3;ortholog	ALPHA-ACTININ-3 (PTHR11915:SF346)
HUMAN|HGNC=166|UniProtKB=O43707		Alpha-actinin-4;ACTN4;ortholog	ALPHA-ACTININ-4 (PTHR11915:SF408)
HUMAN|HGNC=167|UniProtKB=P61163		Alpha-centractin;ACTR1A;ortholog	ALPHA-CENTRACTIN (PTHR11937:SF271)
HUMAN|HGNC=6057|UniProtKB=Q16352	HUMAN|HGNC=6057|UniProtKB=Q16352	Alpha-internexin;INA;ortholog	ALPHA-INTERNEXIN (PTHR23239:SF273)
HUMAN|HGNC=493|UniProtKB=Q01484		Ankyrin-2;ANK2;ortholog	ANKYRIN-2 (PTHR24123:SF39)
HUMAN|HGNC=168|UniProtKB=P42025		Beta-centractin;ACTR1B;ortholog	BETA-CENTRACTIN (PTHR11937:SF273)
	HUMAN|HGNC=2586|UniProtKB=Q9UDT6	CAP-Gly domain-containing linker protein 2;CLIP2;ortholog	CAP-GLY DOMAIN-CONTAINING LINKER PROTEIN 2 (PTHR18916:SF45)
HUMAN|HGNC=13518|UniProtKB=Q9Y696		Chloride intracellular channel protein 4;CLIC4;ortholog	CHLORIDE INTRACELLULAR CHANNEL PROTEIN 4 (PTHR11260:SF443)
	HUMAN|HGNC=2252|UniProtKB=P31146	Coronin-1A;CORO1A;ortholog	CORONIN-1A (PTHR10856:SF31)
HUMAN|HGNC=2961|UniProtKB=Q14204	HUMAN|HGNC=2961|UniProtKB=Q14204	Cytoplasmic dynein 1 heavy chain 1;DYNC1H1;ortholog	CYTOPLASMIC DYNEIN 1 HEAVY CHAIN 1 (PTHR10676:SF302)
HUMAN|HGNC=2770|UniProtKB=P17661	HUMAN|HGNC=2770|UniProtKB=P17661	Desmin;DES;ortholog	DESMIN (PTHR23239:SF307)
HUMAN|HGNC=2696|UniProtKB=Q9UJU6		Drebrin-like protein;DBNL;ortholog	DREBRIN-LIKE PROTEIN (PTHR10829:SF30)
HUMAN|HGNC=2712|UniProtKB=Q13561	HUMAN|HGNC=2712|UniProtKB=Q13561	Dynactin subunit 2;DCTN2;ortholog	DYNACTIN SUBUNIT 2 (PTHR15346:SF1)
HUMAN|HGNC=8140|UniProtKB=O60313	HUMAN|HGNC=8140|UniProtKB=O60313	Dynamin-like 120 kDa protein, mitochondrial;OPA1;ortholog	DYNAMIN-LIKE 120 KDA PROTEIN, MITOCHONDRIAL (PTHR11566:SF100)
HUMAN|HGNC=15476|UniProtKB=P63167	HUMAN|HGNC=15476|UniProtKB=P63167	Dynein light chain 1, cytoplasmic;DYNLL1;ortholog	DYNEIN LIGHT CHAIN 1, CYTOPLASMIC (PTHR11886:SF60)
HUMAN|HGNC=3213|UniProtKB=P26641	HUMAN|HGNC=3213|UniProtKB=P26641	Elongation factor 1-gamma;EEF1G;ortholog	ELONGATION FACTOR 1-GAMMA (PTHR11260:SF420)
HUMAN|HGNC=3383|UniProtKB=P27105		Erythrocyte band 7 integral membrane protein;STOM;ortholog	ERYTHROCYTE BAND 7 INTEGRAL MEMBRANE PROTEIN (PTHR10264:SF109)
HUMAN|HGNC=1488|UniProtKB=P52907	HUMAN|HGNC=1488|UniProtKB=P52907	F-actin-capping protein subunit alpha-1;CAPZA1;ortholog	F-ACTIN-CAPPING PROTEIN SUBUNIT ALPHA-1 (PTHR10653:SF9)
HUMAN|HGNC=1490|UniProtKB=P47755	HUMAN|HGNC=1490|UniProtKB=P47755	F-actin-capping protein subunit alpha-2;CAPZA2;ortholog	F-ACTIN-CAPPING PROTEIN SUBUNIT ALPHA-2 (PTHR10653:SF11)
HUMAN|HGNC=1491|UniProtKB=P47756		F-actin-capping protein subunit beta;CAPZB;ortholog	F-ACTIN-CAPPING PROTEIN SUBUNIT BETA (PTHR10619:SF1)
HUMAN|HGNC=11148|UniProtKB=Q16658	HUMAN|HGNC=11148|UniProtKB=Q16658	Fascin;FSCN1;ortholog	FASCIN (PTHR10551:SF19)
HUMAN|HGNC=15968|UniProtKB=Q8TB36		Ganglioside-induced differentiation-associated protein 1;GDAP1;ortholog	GANGLIOSIDE-INDUCED DIFFERENTIATION-ASSOCIATED PROTEIN 1 (PTHR11260:SF391)
HUMAN|HGNC=4235|UniProtKB=P14136	HUMAN|HGNC=4235|UniProtKB=P14136	Glial fibrillary acidic protein;GFAP;ortholog	GLIAL FIBRILLARY ACIDIC PROTEIN (PTHR23239:SF249)
	HUMAN|HGNC=18415|UniProtKB=O75146	Huntingtin-interacting protein 1-related protein;HIP1R;ortholog	HUNTINGTIN-INTERACTING PROTEIN 1-RELATED PROTEIN (PTHR10407:SF17)
HUMAN|HGNC=6413|UniProtKB=P13645	HUMAN|HGNC=6413|UniProtKB=P13645	Keratin, type I cytoskeletal 10;KRT10;ortholog	KERATIN, TYPE I CYTOSKELETAL 10 (PTHR23239:SF308)
HUMAN|HGNC=6416|UniProtKB=P02533	HUMAN|HGNC=6416|UniProtKB=P02533	Keratin, type I cytoskeletal 14;KRT14;ortholog	KERATIN, TYPE I CYTOSKELETAL 14 (PTHR23239:SF272)

	HUMAN|HGNC=6423|UniProtKB=P08779	Keratin, type I cytoskeletal 16;KRT16;ortholog	KERATIN, TYPE I CYTOSKELETAL 16 (PTHR23239:SF263)
	HUMAN|HGNC=6427|UniProtKB=Q04695	Keratin, type I cytoskeletal 17;KRT17;ortholog	KERATIN, TYPE I CYTOSKELETAL 17 (PTHR23239:SF295)
HUMAN|HGNC=6447|UniProtKB=P35527	HUMAN|HGNC=6447|UniProtKB=P35527	Keratin, type I cytoskeletal 9;KRT9;ortholog	KERATIN, TYPE I CYTOSKELETAL 9 (PTHR23239:SF281)
HUMAN|HGNC=6412|UniProtKB=P04264	HUMAN|HGNC=6412|UniProtKB=P04264	Keratin, type II cytoskeletal 1;KRT1;ortholog	KERATIN, TYPE II CYTOSKELETAL 1 (PTHR23239:SF236)
HUMAN|HGNC=6439|UniProtKB=P35908	HUMAN|HGNC=6439|UniProtKB=P35908	Keratin, type II cytoskeletal 2 epidermal;KRT2;ortholog	KERATIN, TYPE II CYTOSKELETAL 2 EPIDERMAL (PTHR23239:SF228)
HUMAN|HGNC=6442|UniProtKB=P13647	HUMAN|HGNC=6442|UniProtKB=P13647	Keratin, type II cytoskeletal 5;KRT5;ortholog	KERATIN, TYPE II CYTOSKELETAL 5 (PTHR23239:SF279)
	HUMAN|HGNC=6443|UniProtKB=P02538	Keratin, type II cytoskeletal 6A;KRT6A;ortholog	KERATIN, TYPE II CYTOSKELETAL 6A (PTHR23239:SF232)
	HUMAN|HGNC=6444|UniProtKB=P04259	Keratin, type II cytoskeletal 6B;KRT6B;ortholog	KERATIN, TYPE II CYTOSKELETAL 6B-RELATED (PTHR23239:SF293)
	HUMAN|HGNC=28929|UniProtKB=Q7RTS7	Keratin, type II cytoskeletal 74;KRT74;ortholog	KERATIN, TYPE II CYTOSKELETAL 74 (PTHR23239:SF328)
	HUMAN|HGNC=28930|UniProtKB=Q5XKE5	Keratin, type II cytoskeletal 79;KRT79;ortholog	KERATIN, TYPE II CYTOSKELETAL 79 (PTHR23239:SF304)
HUMAN|HGNC=6325|UniProtKB=O60282	HUMAN|HGNC=6325|UniProtKB=O60282	Kinesin heavy chain isoform 5C;KIF5C;ortholog	KINESIN HEAVY CHAIN ISOFORM 5C (PTHR24115:SF633)
	HUMAN|HGNC=6324|UniProtKB=P33176	Kinesin-1 heavy chain;KIF5B;ortholog	KINESIN-1 HEAVY CHAIN (PTHR24115:SF708)
HUMAN|HGNC=6638|UniProtKB=Q03252	HUMAN|HGNC=6638|UniProtKB=Q03252	Lamin-B2;LMNB2;ortholog	LAMIN-B2 (PTHR23239:SF280)
HUMAN|HGNC=6835|UniProtKB=P78559	HUMAN|HGNC=6835|UniProtKB=P78559	Microtubule-associated protein 1A;MAP1A;ortholog	MICROTUBULE-ASSOCIATED PROTEIN 1A (PTHR13843:SF13)
HUMAN|HGNC=6836|UniProtKB=P46821	HUMAN|HGNC=6836|UniProtKB=P46821	Microtubule-associated protein 1B;MAP1B;ortholog	MICROTUBULE-ASSOCIATED PROTEIN 1B (PTHR13843:SF15)
HUMAN|HGNC=6892|UniProtKB=Q9UPY8	HUMAN|HGNC=6892|UniProtKB=Q9UPY8	Microtubule-associated protein RP/EB family member 3;MAPRE3;ortholog	MICROTUBULE-ASSOCIATED PROTEIN RP/EB FAMILY MEMBER 3 (PTHR10623:SF23)
HUMAN|HGNC=7373|UniProtKB=P26038	HUMAN|HGNC=7373|UniProtKB=P26038	Moesin;MSN;ortholog	MOESIN (PTHR23281:SF30)
	HUMAN|HGNC=16701|UniProtKB=P19105	Myosin regulatory light chain 12A;MYL12A;ortholog	MYOSIN REGULATORY LIGHT CHAIN 12A (PTHR23049:SF55)
	HUMAN|HGNC=7568|UniProtKB=P35580	Myosin-10;MYH10;ortholog	MYOSIN-10 (PTHR13140:SF549)
HUMAN|HGNC=7739|UniProtKB=P07196	HUMAN|HGNC=7739|UniProtKB=P07196	Neurofilament light polypeptide;NEFL;ortholog	NEUROFILAMENT LIGHT POLYPEPTIDE (PTHR23239:SF242)
HUMAN|HGNC=9461|UniProtKB=P41219		Peripherin;PRPH;ortholog	PERIPHERIN (PTHR23239:SF288)
HUMAN|HGNC=9944|UniProtKB=P35241		Radixin;RDX;ortholog	RADIXIN (PTHR23281:SF31)
HUMAN|HGNC=9164|UniProtKB=Q99719		Septin-5;SEPT5;ortholog	SEPTIN-5 (PTHR18884:SF107)
HUMAN|HGNC=1717|UniProtKB=Q16181		Septin-7;SEPT7;ortholog	SEPTIN-7 (PTHR18884:SF86)
HUMAN|HGNC=11273|UniProtKB=Q13813		Spectrin alpha chain, non-erythrocytic 1;SPTAN1;ortholog	SPECTRIN ALPHA CHAIN, NON-ERYTHROCYTIC 1 (PTHR11915:SF384)
HUMAN|HGNC=11275|UniProtKB=Q01082	HUMAN|HGNC=11275|UniProtKB=Q01082	Spectrin beta chain, non-erythrocytic 1;SPTBN1;ortholog	SPECTRIN BETA CHAIN, NON-ERYTHROCYTIC 1 (PTHR11915:SF343)
	HUMAN|HGNC=11276|UniProtKB=O15020	Spectrin beta chain, non-erythrocytic 2;SPTBN2;ortholog	SPECTRIN BETA CHAIN, NON-ERYTHROCYTIC 2 (PTHR11915:SF336)
	HUMAN|HGNC=11496|UniProtKB=O14994	Synapsin-3;SYN3;ortholog	SYNAPSIN-3 (PTHR10841:SF25)
HUMAN|HGNC=11845|UniProtKB=Q9Y490	HUMAN|HGNC=11845|UniProtKB=Q9Y490	Talin-1;TLN1;ortholog	TALIN-1 (PTHR19981:SF24)

HUMAN|HGNC=15447|UniProtKB=Q9Y4G6	HUMAN|HGNC=15447|UniProtKB=Q9Y4G6	Talin-2;TLN2;ortholog	TALIN-2 (PTHR19981:SF28)
HUMAN|HGNC=29868|UniProtKB=Q9UI15	HUMAN|HGNC=29868|UniProtKB=Q9UI15	Transgelin-3;TAGLN3;ortholog	TRANSGELIN-3 (PTHR18959:SF64)
HUMAN|HGNC=11553|UniProtKB=Q01995	HUMAN|HGNC=11553|UniProtKB=Q01995	Transgelin;TAGLN;ortholog	TRANSGELIN (PTHR18959:SF65)
HUMAN|HGNC=12022|UniProtKB=P13693		Translationally-controlled tumor protein;TPT1;ortholog	TPT1-LIKE PROTEIN-RELATED (PTHR11991:SF6)
HUMAN|HGNC=12013|UniProtKB=P67936		Tropomyosin alpha-4 chain;TPM4;ortholog	TROPOMYOSIN ALPHA-4 CHAIN (PTHR19269:SF55)
HUMAN|HGNC=12011|UniProtKB=P07951		Tropomyosin beta chain;TPM2;ortholog	TROPOMYOSIN BETA CHAIN (PTHR19269:SF61)
HUMAN|HGNC=12410|UniProtKB=Q9NY65		Tubulin alpha-8 chain;TUBA8;ortholog	TUBULIN ALPHA-8 CHAIN (PTHR11588:SF181)
HUMAN|HGNC=20778|UniProtKB=P07437		Tubulin beta chain;TUBB;ortholog	TUBULIN BETA CHAIN (PTHR11588:SF211)
HUMAN|HGNC=12412|UniProtKB=Q13885	HUMAN|HGNC=12412|UniProtKB=Q13885	Tubulin beta-2A chain;TUBB2A;ortholog	TUBULIN BETA-2A CHAIN (PTHR11588:SF203)
HUMAN|HGNC=30829|UniProtKB=Q9BVA1	HUMAN|HGNC=30829|UniProtKB=Q9BVA1	Tubulin beta-2B chain;TUBB2B;ortholog	TUBULIN BETA-2B CHAIN (PTHR11588:SF153)
HUMAN|HGNC=20772|UniProtKB=Q13509	HUMAN|HGNC=20772|UniProtKB=Q13509	Tubulin beta-3 chain;TUBB3;ortholog	TUBULIN BETA-3 CHAIN (PTHR11588:SF174)
HUMAN|HGNC=20774|UniProtKB=P04350	HUMAN|HGNC=20774|UniProtKB=P04350	Tubulin beta-4A chain;TUBB4A;ortholog	TUBULIN BETA-4A CHAIN (PTHR11588:SF198)
HUMAN|HGNC=20771|UniProtKB=P68371	HUMAN|HGNC=20771|UniProtKB=P68371	Tubulin beta-4B chain;TUBB4B;ortholog	TUBULIN BETA-4B CHAIN (PTHR11588:SF169)
HUMAN|HGNC=20776|UniProtKB=Q9BUF5		Tubulin beta-6 chain;TUBB6;ortholog	TUBULIN BETA-6 CHAIN (PTHR11588:SF212)
HUMAN|HGNC=24164|UniProtKB=O94811	HUMAN|HGNC=24164|UniProtKB=O94811	Tubulin polymerization-promoting protein;TPPP;ortholog	TUBULIN POLYMERIZATION-PROMOTING PROTEIN (PTHR12932:SF20)
	HUMAN|HGNC=11581|UniProtKB=Q9BTW9	Tubulin-specific chaperone D;TBCD;ortholog	TUBULIN-SPECIFIC CHAPERONE D (PTHR12658:SF3)
HUMAN|Ensembl=ENSG00000260914|UniProtKB=I3L4J1		Uncharacterized protein (Fragment);unassigned;ortholog	VACUOLAR PROTEIN SORTING-ASSOCIATED PROTEIN 4A (PTHR23074:SF116)
	HUMAN|HGNC=13488|UniProtKB=Q9UN37	Vacuolar protein sorting-associated protein 4A;VPS4A;ortholog	VACUOLAR PROTEIN SORTING-ASSOCIATED PROTEIN 4A (PTHR23074:SF116)
	HUMAN|HGNC=10895|UniProtKB=O75351	Vacuolar protein sorting-associated protein 4B;VPS4B;ortholog	VACUOLAR PROTEIN SORTING-ASSOCIATED PROTEIN 4B (PTHR23074:SF128)
HUMAN|HGNC=12692|UniProtKB=P08670	HUMAN|HGNC=12692|UniProtKB=P08670	Vimentin;VIM;ortholog	VIMENTIN (PTHR23239:SF258)
	HUMAN|HGNC=12754|UniProtKB=O75083	WD repeat-containing protein 1;WDR1;ortholog	WD REPEAT-CONTAINING PROTEIN 1 (PTHR19856:SF2)

defense/immunity protein (PC00090)

HUMAN|HGNC=2172|UniProtKB=Q02246		Contactin-2;CNTN2;ortholog	CONTACTIN-2 (PTHR10489:SF882)
HUMAN|HGNC=26361|UniProtKB=Q14CZ8		Hepatocyte cell adhesion molecule;HEPACAM;ortholog	HEPATOCYTE CELL ADHESION MOLECULE (PTHR12080:SF66)
HUMAN|HGNC=4931|UniProtKB=P01891		HLA class I histocompatibility antigen, A-68 alpha chain;HLA-A;ortholog	HLA CLASS I HISTOCOMPATIBILITY ANTIGEN, A-68 ALPHA CHAIN (PTHR16675:SF191)
HUMAN|HGNC=28246|UniProtKB=Q96ID5		Immunoglobulin superfamily member 21;IGSF21;ortholog	IMMUNOGLOBULIN SUPERFAMILY MEMBER 21 (PTHR10489:SF771)
HUMAN|HGNC=7656|UniProtKB=P13591		Neural cell adhesion molecule 1;NCAM1;ortholog	NEURAL CELL ADHESION MOLECULE 1 (PTHR10489:SF783)

enzyme modulator (PC00095)

HUMAN|HGNC=9060|UniProtKB=P51178		1-phosphatidylinositol 4,5-bisphosphate phosphodiesterase delta-1;PLCD1;ortholog	1-PHOSPHATIDYLINOSITOL 4,5-BISPHOSPHATE PHOSPHODIESTERASE DELTA-1 (PTHR10336:SF126)

HUMAN|HGNC=9061|UniProtKB=Q8N3E9		1-phosphatidylinositol 4,5-bisphosphate phosphodiesterase delta-3;PLCD3;ortholog	1-PHOSPHATIDYLINOSITOL 4,5-BISPHOSPHATE PHOSPHODIESTERASE DELTA-3 (PTHR10336:SF141)
	HUMAN|HGNC=9557|UniProtKB=O00232	26S proteasome non-ATPase regulatory subunit 12;PSMD12;ortholog	26S PROTEASOME NON-ATPASE REGULATORY SUBUNIT 12 (PTHR10855:SF4)
	HUMAN|HGNC=9558|UniProtKB=Q9UNM6	26S proteasome non-ATPase regulatory subunit 13;PSMD13;ortholog	26S PROTEASOME NON-ATPASE REGULATORY SUBUNIT 13 (PTHR10539:SF1)
HUMAN|HGNC=9559|UniProtKB=Q13200		26S proteasome non-ATPase regulatory subunit 2;PSMD2;ortholog	26S PROTEASOME NON-ATPASE REGULATORY SUBUNIT 2 (PTHR10943:SF7)
HUMAN|HGNC=9560|UniProtKB=O43242		26S proteasome non-ATPase regulatory subunit 3;PSMD3;ortholog	26S PROTEASOME NON-ATPASE REGULATORY SUBUNIT 3 (PTHR10758:SF6)
	HUMAN|HGNC=654|UniProtKB=P61204	ADP-ribosylation factor 3;ARF3;ortholog	ADP-RIBOSYLATION FACTOR 3 (PTHR11711:SF288)
HUMAN|HGNC=655|UniProtKB=P18085		ADP-ribosylation factor 4;ARF4;ortholog	ADP-RIBOSYLATION FACTOR 4 (PTHR11711:SF233)
	HUMAN|HGNC=658|UniProtKB=P84085	ADP-ribosylation factor 5;ARF5;ortholog	ADP-RIBOSYLATION FACTOR 5 (PTHR11711:SF278)
	HUMAN|HGNC=659|UniProtKB=P62330	ADP-ribosylation factor 6;ARF6;ortholog	ADP-RIBOSYLATION FACTOR 6 (PTHR11711:SF231)
HUMAN|HGNC=693|UniProtKB=P36404	HUMAN|HGNC=693|UniProtKB=P36404	ADP-ribosylation factor-like protein 2;ARL2;ortholog	ADP-RIBOSYLATION FACTOR-LIKE PROTEIN 2-RELATED (PTHR11711:SF230)
	HUMAN|HGNC=694|UniProtKB=P36405	ADP-ribosylation factor-like protein 3;ARL3;ortholog	ADP-RIBOSYLATION FACTOR-LIKE PROTEIN 3 (PTHR11711:SF261)
	HUMAN|HGNC=25564|UniProtKB=Q9NVJ2	ADP-ribosylation factor-like protein 8B;ARL8B;ortholog	ADP-RIBOSYLATION FACTOR-LIKE PROTEIN 8B (PTHR11711:SF219)
HUMAN|HGNC=14678|UniProtKB=Q96CX2	HUMAN|HGNC=14678|UniProtKB=Q96CX2	BTB/POZ domain-containing protein KCTD12;KCTD12;ortholog	BTB/POZ DOMAIN-CONTAINING PROTEIN KCTD12 (PTHR14499:SF103)
HUMAN|HGNC=29244|UniProtKB=Q68DU8	HUMAN|HGNC=29244|UniProtKB=Q68DU8	BTB/POZ domain-containing protein KCTD16;KCTD16;ortholog	BTB/POZ DOMAIN-CONTAINING PROTEIN KCTD16 (PTHR14499:SF81)
HUMAN|HGNC=2460|UniProtKB=P67870		Casein kinase II subunit beta;CSNK2B;ortholog	CASEIN KINASE II SUBUNIT BETA (PTHR11740:SF12)
HUMAN|HGNC=1736|UniProtKB=P60953	HUMAN|HGNC=1736|UniProtKB=P60953	Cell division control protein 42 homolog;CDC42;ortholog	CELL DIVISION CONTROL PROTEIN 42 HOMOLOG (PTHR24072:SF239)
HUMAN|HGNC=8011|UniProtKB=P78357	HUMAN|HGNC=8011|UniProtKB=P78357	Contactin-associated protein 1;CNTNAP1;ortholog	CONTACTIN-ASSOCIATED PROTEIN 1 (PTHR10127:SF740)
	HUMAN|HGNC=2475|UniProtKB=P01034	Cystatin-C;CST3;ortholog	CYSTATIN-C (PTHR11413:SF69)
	HUMAN|HGNC=7665|UniProtKB=O43639	Cytoplasmic protein NCK2;NCK2;ortholog	CYTOPLASMIC PROTEIN NCK2 (PTHR10663:SF280)
	HUMAN|HGNC=24035|UniProtKB=Q9UKG1	DCC-interacting protein 13-alpha;APPL1;ortholog	DCC-INTERACTING PROTEIN 13-ALPHA (PTHR23180:SF350)
HUMAN|HGNC=8140|UniProtKB=O60313	HUMAN|HGNC=8140|UniProtKB=O60313	Dynamin-like 120 kDa protein, mitochondrial;OPA1;ortholog	DYNAMIN-LIKE 120 KDA PROTEIN, MITOCHONDRIAL (PTHR11566:SF100)
HUMAN|HGNC=15476|UniProtKB=P63167	HUMAN|HGNC=15476|UniProtKB=P63167	Dynein light chain 1, cytoplasmic;DYNLL1;ortholog	DYNEIN LIGHT CHAIN 1, CYTOPLASMIC (PTHR11886:SF60)
HUMAN|HGNC=3244|UniProtKB=Q9NZN3	HUMAN|HGNC=3244|UniProtKB=Q9NZN3	EH domain-containing protein 3;EHD3;ortholog	EH DOMAIN-CONTAINING PROTEIN 3 (PTHR11216:SF88)
HUMAN|HGNC=3189|UniProtKB=P68104	HUMAN|HGNC=3189|UniProtKB=P68104	Elongation factor 1-alpha 1;EEF1A1;ortholog	ELONGATION FACTOR 1-ALPHA 1 (PTHR23115:SF216)
HUMAN|HGNC=3192|UniProtKB=Q05639	HUMAN|HGNC=3192|UniProtKB=Q05639	Elongation factor 1-alpha 2;EEF1A2;ortholog	ELONGATION FACTOR 1-ALPHA 2 (PTHR23115:SF203)
HUMAN|HGNC=3214|UniProtKB=P13639	HUMAN|HGNC=3214|UniProtKB=P13639	Elongation factor 2;EEF2;ortholog	ELONGATION FACTOR 2 (PTHR42908:SF8)
HUMAN|HGNC=12420|UniProtKB=P49411	HUMAN|HGNC=12420|UniProtKB=P49411	Elongation factor Tu, mitochondrial;TUFM;ortholog	ELONGATION FACTOR TU, MITOCHONDRIAL (PTHR43721:SF7)
HUMAN|HGNC=10831|UniProtKB=Q99962	HUMAN|HGNC=10831|UniProtKB=Q99962	Endophilin-A1;SH3GL2;ortholog	ENDOPHILIN-A1 (PTHR10663:SF240)

	HUMAN|HGNC=10833|UniProtKB=Q9Y371	Endophilin-B1;SH3GLB1;ortholog	ENDOPHILIN-B1 (PTHR10663:SF262)
	HUMAN|HGNC=3267|UniProtKB=P41091	Eukaryotic translation initiation factor 2 subunit 3;EIF2S3;ortholog	EUKARYOTIC TRANSLATION INITIATION FACTOR 2 SUBUNIT 3 (PTHR42854:SF1)
HUMAN|HGNC=3299|UniProtKB=P55010		Eukaryotic translation initiation factor 5;EIF5;ortholog	EUKARYOTIC TRANSLATION INITIATION FACTOR 5 (PTHR23001:SF11)
	HUMAN|HGNC=3591|UniProtKB=Q9Y4F1	FERM, RhoGEF and pleckstrin domain-containing protein 1;FARP1;ortholog	FERM, RHOGEF AND PLECKSTRIN DOMAIN-CONTAINING PROTEIN 1 (PTHR12673:SF153)
HUMAN|HGNC=4373|UniProtKB=P60983		Glia maturation factor beta;GMFB;ortholog	GLIA MATURATION FACTOR BETA (PTHR11249:SF5)
HUMAN|HGNC=9846|UniProtKB=P62826	HUMAN|HGNC=9846|UniProtKB=P62826	GTP-binding nuclear protein Ran;RAN;ortholog	GTP-BINDING NUCLEAR PROTEIN RAN (PTHR24071:SF14)
	HUMAN|HGNC=19127|UniProtKB=O95057	GTP-binding protein Di-Ras1;DIRAS1;ortholog	GTP-BINDING PROTEIN DI-RAS1 (PTHR24070:SF295)
	HUMAN|HGNC=19323|UniProtKB=Q96HU8	GTP-binding protein Di-Ras2;DIRAS2;ortholog	GTP-BINDING PROTEIN DI-RAS2 (PTHR24070:SF281)
HUMAN|HGNC=10534|UniProtKB=Q9NR31	HUMAN|HGNC=10534|UniProtKB=Q9NR31	GTP-binding protein SAR1a;SAR1A;ortholog	GTP-BINDING PROTEIN SAR1A (PTHR11711:SF256)
HUMAN|HGNC=7989|UniProtKB=P01111		GTPase NRas;NRAS;ortholog	GTPASE NRAS (PTHR24070:SF354)
HUMAN|HGNC=4384|UniProtKB=P63096	HUMAN|HGNC=4384|UniProtKB=P63096	Guanine nucleotide-binding protein G(i) subunit alpha-1;GNAI1;ortholog	GUANINE NUCLEOTIDE-BINDING PROTEIN G(I) SUBUNIT ALPHA-1 (PTHR10218:SF283)
HUMAN|HGNC=4385|UniProtKB=P04899	HUMAN|HGNC=4385|UniProtKB=P04899	Guanine nucleotide-binding protein G(i) subunit alpha-2;GNAI2;ortholog	GUANINE NUCLEOTIDE-BINDING PROTEIN G(I) SUBUNIT ALPHA-2 (PTHR10218:SF279)
HUMAN|HGNC=4398|UniProtKB=P62879	HUMAN|HGNC=4398|UniProtKB=P62879	Guanine nucleotide-binding protein G(I)/G(S)/G(T) subunit beta-2;GNB2;ortholog	GUANINE NUCLEOTIDE-BINDING PROTEIN G(I)/G(S)/G(T) SUBUNIT BETA-2 (PTHR19850:SF39)
HUMAN|HGNC=4413|UniProtKB=P36915		Guanine nucleotide-binding protein G(I)/G(S)/G(T) subunit beta-2;GNB2;ortholog	GUANINE NUCLEOTIDE-BINDING PROTEIN G(I)/G(S)/G(T) SUBUNIT BETA-2 (PTHR19850:SF39)
HUMAN|HGNC=4387|UniProtKB=P08754		Guanine nucleotide-binding protein G(k) subunit alpha;GNAI3;ortholog	GUANINE NUCLEOTIDE-BINDING PROTEIN G(K) SUBUNIT ALPHA (PTHR10218:SF289)
HUMAN|HGNC=4389|UniProtKB=P09471	HUMAN|HGNC=4389|UniProtKB=P09471	Guanine nucleotide-binding protein G(o) subunit alpha;GNAO1;ortholog	GUANINE NUCLEOTIDE-BINDING PROTEIN G(O) SUBUNIT ALPHA (PTHR10218:SF248)
HUMAN|HGNC=4390|UniProtKB=P50148	HUMAN|HGNC=4390|UniProtKB=P50148	Guanine nucleotide-binding protein G(q) subunit alpha;GNAQ;ortholog	GUANINE NUCLEOTIDE-BINDING PROTEIN G(Q) SUBUNIT ALPHA (PTHR10218:SF255)
HUMAN|HGNC=4395|UniProtKB=P19086	HUMAN|HGNC=4395|UniProtKB=P19086	Guanine nucleotide-binding protein G(z) subunit alpha;GNAZ;ortholog	GUANINE NUCLEOTIDE-BINDING PROTEIN G(Z) SUBUNIT ALPHA (PTHR10218:SF268)
HUMAN|HGNC=4379|UniProtKB=P29992	HUMAN|HGNC=4379|UniProtKB=P29992	Guanine nucleotide-binding protein subunit alpha-11;GNA11;ortholog	GUANINE NUCLEOTIDE-BINDING PROTEIN SUBUNIT ALPHA-11 (PTHR10218:SF262)
HUMAN|HGNC=4380|UniProtKB=Q03113	HUMAN|HGNC=4380|UniProtKB=Q03113	Guanine nucleotide-binding protein subunit alpha-12;GNA12;ortholog	GUANINE NUCLEOTIDE-BINDING PROTEIN SUBUNIT ALPHA-12 (PTHR10218:SF253)
HUMAN|HGNC=4381|UniProtKB=Q14344	HUMAN|HGNC=4381|UniProtKB=Q14344	Guanine nucleotide-binding protein subunit alpha-13;GNA13;ortholog	GUANINE NUCLEOTIDE-BINDING PROTEIN SUBUNIT ALPHA-13 (PTHR10218:SF271)
HUMAN|HGNC=20731|UniProtKB=Q9HAV0		Guanine nucleotide-binding protein subunit beta-4;GNB4;ortholog	GUANINE NUCLEOTIDE-BINDING PROTEIN SUBUNIT BETA-4 (PTHR19850:SF42)
HUMAN|HGNC=9852|UniProtKB=O95373		Importin-7;IPO7;ortholog	IMPORTIN-7 (PTHR10997:SF39)
HUMAN|HGNC=29112|UniProtKB=Q6DN90	HUMAN|HGNC=29112|UniProtKB=Q6DN90	IQ motif and SEC7 domain-containing protein 1;IQSEC1;ortholog	IQ MOTIF AND SEC7 DOMAIN-CONTAINING PROTEIN 1 (PTHR10663:SF219)
	HUMAN|HGNC=7568|UniProtKB=P35580	Myosin-10;MYH10;ortholog	MYOSIN-10 (PTHR13140:SF549)
HUMAN|HGNC=7640|UniProtKB=Q99733		Nucleosome assembly protein 1-like 4;NAP1L4;ortholog	NUCLEOSOME ASSEMBLY PROTEIN 1-LIKE 4 (PTHR11875:SF88)
HUMAN|HGNC=28833|UniProtKB=Q9NTK5	HUMAN|HGNC=28833|UniProtKB=Q9NTK5	Obg-like ATPase 1;OLA1;ortholog	OBG-LIKE ATPASE 1 (PTHR23305:SF17)

HUMAN|HGNC=8647|UniProtKB=Q15365	HUMAN|HGNC=8647|UniProtKB=Q15365	Poly(rC)-binding protein 1;PCBP1;ortholog	POLY(RC)-BINDING PROTEIN 1 (PTHR10288:SF191)
HUMAN|HGNC=29180|UniProtKB=Q9UPV7	HUMAN|HGNC=29180|UniProtKB=Q9UPV7	Protein KIAA1045;KIAA1045;ortholog	PROTEIN KIAA1045 (PTHR10891:SF706)
HUMAN|HGNC=4226|UniProtKB=P31150	HUMAN|HGNC=4226|UniProtKB=P31150	Rab GDP dissociation inhibitor alpha;GDI1;ortholog	RAB GDP DISSOCIATION INHIBITOR ALPHA (PTHR11787:SF19)
HUMAN|HGNC=4227|UniProtKB=P50395		Rab GDP dissociation inhibitor beta;GDI2;ortholog	RAB GDP DISSOCIATION INHIBITOR BETA (PTHR11787:SF21)
HUMAN|HGNC=17168|UniProtKB=Q9H2M9		Rab3 GTPase-activating protein non-catalytic subunit;RAB3GAP2;ortholog	RAB3 GTPASE-ACTIVATING PROTEIN NON-CATALYTIC SUBUNIT (PTHR12472:SF1)
	HUMAN|HGNC=16854|UniProtKB=Q9Y4G8	Rap guanine nucleotide exchange factor 2;RAPGEF2;ortholog	RAP GUANINE NUCLEOTIDE EXCHANGE FACTOR 2 (PTHR23113:SF242)
HUMAN|HGNC=9801|UniProtKB=P63000	HUMAN|HGNC=9801|UniProtKB=P63000	Ras-related C3 botulinum toxin substrate 1;RAC1;ortholog	RAS-RELATED C3 BOTULINUM TOXIN SUBSTRATE 1 (PTHR24072:SF194)
HUMAN|HGNC=9803|UniProtKB=P60763		Ras-related C3 botulinum toxin substrate 3;RAC3;ortholog	RAS-RELATED C3 BOTULINUM TOXIN SUBSTRATE 3 (PTHR24072:SF220)
HUMAN|HGNC=7227|UniProtKB=O14807		Ras-related protein M-Ras;MRAS;ortholog	RAS-RELATED PROTEIN M-RAS (PTHR24070:SF321)
HUMAN|HGNC=10447|UniProtKB=P10301		Ras-related protein R-Ras;RRAS;ortholog	RAS-RELATED PROTEIN R-RAS (PTHR24070:SF351)
HUMAN|HGNC=9839|UniProtKB=P11233		Ras-related protein Ral-A;RALA;ortholog	RAS-RELATED PROTEIN RAL-A (PTHR24070:SF277)
HUMAN|HGNC=9840|UniProtKB=P11234		Ras-related protein Ral-B;RALB;ortholog	RAS-RELATED PROTEIN RAL-B (PTHR24070:SF348)
	HUMAN|HGNC=9855|UniProtKB=P62834	Ras-related protein Rap-1A;RAP1A;ortholog	RAS-RELATED PROTEIN RAP-1A (PTHR24070:SF363)
HUMAN|HGNC=9861|UniProtKB=P10114	HUMAN|HGNC=9861|UniProtKB=P10114	Ras-related protein Rap-2a;RAP2A;ortholog	RAS-RELATED PROTEIN RAP-2A (PTHR24070:SF364)
HUMAN|HGNC=9862|UniProtKB=P61225	HUMAN|HGNC=9862|UniProtKB=P61225	Ras-related protein Rap-2b;RAP2B;ortholog	RAS-RELATED PROTEIN RAP-2B (PTHR24070:SF346)
HUMAN|HGNC=673|UniProtKB=Q07960	HUMAN|HGNC=673|UniProtKB=Q07960	Rho GTPase-activating protein 1;ARHGAP1;ortholog	RHO GTPASE-ACTIVATING PROTEIN 1 (PTHR23176:SF59)
HUMAN|HGNC=668|UniProtKB=P62745	HUMAN|HGNC=668|UniProtKB=P62745	Rho-related GTP-binding protein RhoB;RHOB;ortholog	RHO-RELATED GTP-BINDING PROTEIN RHOB (PTHR24072:SF182)
HUMAN|HGNC=672|UniProtKB=P84095	HUMAN|HGNC=672|UniProtKB=P84095	Rho-related GTP-binding protein RhoG;RHOG;ortholog	RHO-RELATED GTP-BINDING PROTEIN RHOG (PTHR24072:SF210)
	HUMAN|HGNC=7887|UniProtKB=Q9UNW9	RNA-binding protein Nova-2;NOVA2;ortholog	RNA-BINDING PROTEIN NOVA-2 (PTHR10288:SF200)
HUMAN|HGNC=9164|UniProtKB=Q99719		Septin-5;SEPT5;ortholog	SEPTIN-5 (PTHR18884:SF107)
HUMAN|HGNC=1717|UniProtKB=Q16181		Septin-7;SEPT7;ortholog	SEPTIN-7 (PTHR18884:SF86)
HUMAN|HGNC=667|UniProtKB=P61586	HUMAN|HGNC=667|UniProtKB=P61586	Transforming protein RhoA;RHOA;ortholog	TRANSFORMING PROTEIN RHOA (PTHR24072:SF212)
**extracellular matrix protein (PC00102)**			
	HUMAN|HGNC=5273|UniProtKB=P98160	Basement membrane-specific heparan sulfate proteoglycan core protein;HSPG2;ortholog	BASEMENT MEMBRANE-SPECIFIC HEPARAN SULFATE PROTEOGLYCAN CORE PROTEIN (PTHR10574:SF343)
HUMAN|HGNC=1044|UniProtKB=P21810	HUMAN|HGNC=1044|UniProtKB=P21810	Biglycan;BGN;ortholog	BIGLYCAN (PTHR24369:SF99)
HUMAN|HGNC=23059|UniProtKB=Q96GW7	HUMAN|HGNC=23059|UniProtKB=Q96GW7	Brevican core protein;BCAN;ortholog	BREVICAN CORE PROTEIN (PTHR22804:SF45)
HUMAN|HGNC=8011|UniProtKB=P78357	HUMAN|HGNC=8011|UniProtKB=P78357	Contactin-associated protein 1;CNTNAP1;ortholog	CONTACTIN-ASSOCIATED PROTEIN 1 (PTHR10127:SF740)
	HUMAN|HGNC=2380|UniProtKB=P10915	Hyaluronan and proteoglycan link protein 1;HAPLN1;ortholog	HYALURONAN AND PROTEOGLYCAN LINK PROTEIN 1 (PTHR22804:SF46)

HUMAN|HGNC=17410|UniProtKB=Q9GZV7		Hyaluronan and proteoglycan link protein 2;HAPLN2;ortholog	HYALURONAN AND PROTEOGLYCAN LINK PROTEIN 2 (PTHR22804:SF51)
	HUMAN|HGNC=31357|UniProtKB=Q86UW8	Hyaluronan and proteoglycan link protein 4;HAPLN4;ortholog	HYALURONAN AND PROTEOGLYCAN LINK PROTEIN 4 (PTHR22804:SF52)
	HUMAN|HGNC=6485|UniProtKB=O15230	Laminin subunit alpha-5;LAMA5;ortholog	LAMININ SUBUNIT ALPHA-5 (PTHR10574:SF355)
HUMAN|HGNC=6487|UniProtKB=P55268	HUMAN|HGNC=6487|UniProtKB=P55268	Laminin subunit beta-2;LAMB2;ortholog	LAMININ SUBUNIT BETA-2 (PTHR10574:SF346)
	HUMAN|HGNC=6492|UniProtKB=P11047	Laminin subunit gamma-1;LAMC1;ortholog	LAMININ SUBUNIT GAMMA-1 (PTHR10574:SF309)
HUMAN|HGNC=2465|UniProtKB=O14594	HUMAN|HGNC=2465|UniProtKB=O14594	Neurocan core protein;NCAN;ortholog	NEUROCAN CORE PROTEIN (PTHR22804:SF53)
HUMAN|HGNC=8135|UniProtKB=P23515		Oligodendrocyte-myelin glycoprotein;OMG;ortholog	OLIGODENDROCYTE-MYELIN GLYCOPROTEIN (PTHR24373:SF208)
HUMAN|HGNC=9357|UniProtKB=P51888	HUMAN|HGNC=9357|UniProtKB=P51888	Prolargin;PRELP;ortholog	PROLARGIN (PTHR24373:SF224)
**hydrolase (PC00121)**			
HUMAN|HGNC=9060|UniProtKB=P51178		1-phosphatidylinositol 4,5-bisphosphate phosphodiesterase delta-1;PLCD1;ortholog	1-PHOSPHATIDYLINOSITOL 4,5-BISPHOSPHATE PHOSPHODIESTERASE DELTA-1 (PTHR10336:SF126)
HUMAN|HGNC=9061|UniProtKB=Q8N3E9		1-phosphatidylinositol 4,5-bisphosphate phosphodiesterase delta-3;PLCD3;ortholog	1-PHOSPHATIDYLINOSITOL 4,5-BISPHOSPHATE PHOSPHODIESTERASE DELTA-3 (PTHR10336:SF141)
	HUMAN|HGNC=9553|UniProtKB=P62333	26S protease regulatory subunit 10B;PSMC6;ortholog	26S PROTEASE REGULATORY SUBUNIT 10B (PTHR23073:SF49)
	HUMAN|HGNC=9548|UniProtKB=P35998	26S protease regulatory subunit 7;PSMC2;ortholog	26S PROTEASE REGULATORY SUBUNIT 7 (PTHR23073:SF46)
HUMAN|HGNC=14169|UniProtKB=Q6P587		Acylpyruvase FAHD1, mitochondrial;FAHD1;ortholog	ACYLPYRUVASE FAHD1, MITOCHONDRIAL (PTHR11820:SF95)
HUMAN|HGNC=823|UniProtKB=P25705	HUMAN|HGNC=823|UniProtKB=P25705	ATP synthase subunit alpha, mitochondrial;ATP5A1;ortholog	ATP SYNTHASE SUBUNIT ALPHA, MITOCHONDRIAL (PTHR43089:SF1)
HUMAN|HGNC=830|UniProtKB=P06576	HUMAN|HGNC=830|UniProtKB=P06576	ATP synthase subunit beta, mitochondrial;ATP5B;ortholog	ATP SYNTHASE SUBUNIT BETA, MITOCHONDRIAL (PTHR15184:SF59)
HUMAN|HGNC=850|UniProtKB=P48047	HUMAN|HGNC=850|UniProtKB=P48047	ATP synthase subunit O, mitochondrial;ATP5O;ortholog	ATP SYNTHASE SUBUNIT O, MITOCHONDRIAL (PTHR11910:SF9)
HUMAN|HGNC=1476|UniProtKB=P07384		Calpain-1 catalytic subunit;CAPN1;ortholog	CALPAIN-1 CATALYTIC SUBUNIT (PTHR10183:SF343)
HUMAN|HGNC=2529|UniProtKB=P07339	HUMAN|HGNC=2529|UniProtKB=P07339	Cathepsin D;CTSD;ortholog	CATHEPSIN D (PTHR13683:SF487)
HUMAN|HGNC=2172|UniProtKB=Q02246		Contactin-2;CNTN2;ortholog	CONTACTIN-2 (PTHR10489:SF882)
HUMAN|HGNC=8011|UniProtKB=P78357	HUMAN|HGNC=8011|UniProtKB=P78357	Contactin-associated protein 1;CNTNAP1;ortholog	CONTACTIN-ASSOCIATED PROTEIN 1 (PTHR10127:SF740)
HUMAN|HGNC=12585|UniProtKB=P31930	HUMAN|HGNC=12585|UniProtKB=P31930	Cytochrome b-c1 complex subunit 1, mitochondrial;UQCRC1;ortholog	CYTOCHROME B-C1 COMPLEX SUBUNIT 1, MITOCHONDRIAL (PTHR11851:SF161)
HUMAN|HGNC=12586|UniProtKB=P22695	HUMAN|HGNC=12586|UniProtKB=P22695	Cytochrome b-c1 complex subunit 2, mitochondrial;UQCRC2;ortholog	CYTOCHROME B-C1 COMPLEX SUBUNIT 2, MITOCHONDRIAL (PTHR11851:SF172)
HUMAN|HGNC=2961|UniProtKB=Q14204	HUMAN|HGNC=2961|UniProtKB=Q14204	Cytoplasmic dynein 1 heavy chain 1;DYNC1H1;ortholog	CYTOPLASMIC DYNEIN 1 HEAVY CHAIN 1 (PTHR10676:SF302)
HUMAN|HGNC=16991|UniProtKB=Q07065		Cytoskeleton-associated protein 4;CKAP4;ortholog	CYTOSKELETON-ASSOCIATED PROTEIN 4 (PTHR18937:SF294)
HUMAN|HGNC=24437|UniProtKB=Q96KP4		Cytosolic non-specific dipeptidase;CNDP2;ortholog	CYTOSOLIC NON-SPECIFIC DIPEPTIDASE (PTHR43270:SF6)
	HUMAN|HGNC=2365|UniProtKB=Q14194	Dihydropyrimidinase-related protein 1;CRMP1;ortholog	DIHYDROPYRIMIDINASE-RELATED PROTEIN 1 (PTHR11647:SF80)
HUMAN|HGNC=3015|UniProtKB=Q14195	HUMAN|HGNC=3015|UniProtKB=Q14195	Dihydropyrimidinase-related protein 3;DPYSL3;ortholog	DIHYDROPYRIMIDINASE-RELATED PROTEIN 3 (PTHR11647:SF86)

HUMAN|HGNC=3016|UniProtKB=O14531	HUMAN|HGNC=3016|UniProtKB=O14531	Dihydropyrimidinase-related protein 4;DPYSL4;ortholog	DIHYDROPYRIMIDINASE-RELATED PROTEIN 4 (PTHR11647:SF82)
HUMAN|HGNC=20637|UniProtKB=Q9BPU6		Dihydropyrimidinase-related protein 5;DPYSL5;ortholog	DIHYDROPYRIMIDINASE-RELATED PROTEIN 5 (PTHR11647:SF85)
	HUMAN|HGNC=17621|UniProtKB=Q8NFP7	Diphosphoinositol polyphosphate phosphohydrolase 3-alpha;NUDT10;ortholog	DIPHOSPHOINOSITOL POLYPHOSPHATE PHOSPHOHYDROLASE 3-ALPHA (PTHR12629:SF23)
HUMAN|HGNC=8140|UniProtKB=O60313	HUMAN|HGNC=8140|UniProtKB=O60313	Dynamin-like 120 kDa protein, mitochondrial;OPA1;ortholog	DYNAMIN-LIKE 120 KDA PROTEIN, MITOCHONDRIAL (PTHR11566:SF100)
HUMAN|HGNC=23409|UniProtKB=Q6UWR7		Ectonucleotide pyrophosphatase/phosphodiesterase family member 6;ENPP6;ortholog	ECTONUCLEOTIDE PYROPHOSPHATASE/PHOSPHODIESTERASE FAMILY MEMBER 6 (PTHR10151:SF82)
HUMAN|HGNC=3189|UniProtKB=P68104	HUMAN|HGNC=3189|UniProtKB=P68104	Elongation factor 1-alpha 1;EEF1A1;ortholog	ELONGATION FACTOR 1-ALPHA 1 (PTHR23115:SF216)
HUMAN|HGNC=3192|UniProtKB=Q05639	HUMAN|HGNC=3192|UniProtKB=Q05639	Elongation factor 1-alpha 2;EEF1A2;ortholog	ELONGATION FACTOR 1-ALPHA 2 (PTHR23115:SF203)
HUMAN|HGNC=3214|UniProtKB=P13639	HUMAN|HGNC=3214|UniProtKB=P13639	Elongation factor 2;EEF2;ortholog	ELONGATION FACTOR 2 (PTHR42908:SF8)
HUMAN|HGNC=12420|UniProtKB=P49411	HUMAN|HGNC=12420|UniProtKB=P49411	Elongation factor Tu, mitochondrial;TUFM;ortholog	ELONGATION FACTOR TU, MITOCHONDRIAL (PTHR43721:SF7)
	HUMAN|HGNC=3267|UniProtKB=P41091	Eukaryotic translation initiation factor 2 subunit 3;EIF2S3;ortholog	EUKARYOTIC TRANSLATION INITIATION FACTOR 2 SUBUNIT 3 (PTHR42854:SF1)
HUMAN|HGNC=3594|UniProtKB=P49327	HUMAN|HGNC=3594|UniProtKB=P49327	Fatty acid synthase;FASN;ortholog	FATTY ACID SYNTHASE (PTHR43775:SF2)
HUMAN|HGNC=4331|UniProtKB=O94925	HUMAN|HGNC=4331|UniProtKB=O94925	Glutaminase kidney isoform, mitochondrial;GLS;ortholog	GLUTAMINASE KIDNEY ISOFORM, MITOCHONDRIAL (PTHR12544:SF41)
HUMAN|HGNC=4212|UniProtKB=Q9Y2T3		Guanine deaminase;GDA;ortholog	GUANINE DEAMINASE (PTHR11271:SF47)
HUMAN|HGNC=4398|UniProtKB=P62879	HUMAN|HGNC=4398|UniProtKB=P62879	Guanine nucleotide-binding protein G(I)/G(S)/G(T) subunit beta-2;GNB2;ortholog	GUANINE NUCLEOTIDE-BINDING PROTEIN G(I)/G(S)/G(T) SUBUNIT BETA-2 (PTHR19850:SF39)
HUMAN|HGNC=20731|UniProtKB=Q9HAV0		Guanine nucleotide-binding protein subunit beta-4;GNB4;ortholog	GUANINE NUCLEOTIDE-BINDING PROTEIN SUBUNIT BETA-4 (PTHR19850:SF42)
HUMAN|HGNC=4413|UniProtKB=P36915		Guanine nucleotide-binding protein-like 1;GNL1;ortholog	GUANINE NUCLEOTIDE-BINDING PROTEIN-LIKE 1 (PTHR11089:SF55)
HUMAN|HGNC=18344|UniProtKB=Q9BX68		Histidine triad nucleotide-binding protein 2, mitochondrial;HINT2;ortholog	HISTIDINE TRIAD NUCLEOTIDE-BINDING PROTEIN 2, MITOCHONDRIAL (PTHR23089:SF26)
HUMAN|HGNC=28246|UniProtKB=Q96ID5		Immunoglobulin superfamily member 21;IGSF21;ortholog	IMMUNOGLOBULIN SUPERFAMILY MEMBER 21 (PTHR10489:SF771)
HUMAN|HGNC=122|UniProtKB=P24666		Low molecular weight phosphotyrosine protein phosphatase;ACP1;ortholog	LOW MOLECULAR WEIGHT PHOSPHOTYROSINE PROTEIN PHOSPHATASE (PTHR11717:SF26)
HUMAN|HGNC=33711|UniProtKB=Q68D91		Metallo-beta-lactamase domain-containing protein 2;MBLAC2;ortholog	METALLO-BETA-LACTAMASE DOMAIN-CONTAINING PROTEIN 2 (PTHR42951:SF4)
HUMAN|HGNC=18667|UniProtKB=Q10713		Mitochondrial-processing peptidase subunit alpha;PMPCA;ortholog	MITOCHONDRIAL-PROCESSING PEPTIDASE SUBUNIT ALPHA (PTHR11851:SF164)
	HUMAN|HGNC=10542|UniProtKB=O95248	Myotubularin-related protein 5;SBF1;ortholog	MYOTUBULARIN-RELATED PROTEIN 5 (PTHR10807:SF91)
HUMAN|HGNC=2715|UniProtKB=O94760		N(G),N(G)-dimethylarginine dimethylaminohydrolase 1;DDAH1;ortholog	N(G),N(G)-DIMETHYLARGININE DIMETHYLAMINOHYDROLASE 1 (PTHR12737:SF25)
HUMAN|HGNC=7656|UniProtKB=P13591		Neural cell adhesion molecule 1;NCAM1;ortholog	NEURAL CELL ADHESION MOLECULE 1 (PTHR10489:SF783)
HUMAN|HGNC=29260|UniProtKB=Q6PIU2		Neutral cholesterol ester hydrolase 1;NCEH1;ortholog	NEUTRAL CHOLESTEROL ESTER HYDROLASE 1 (PTHR23024:SF275)

HUMAN|HGNC=9325|UniProtKB=P50897		Palmitoyl-protein thioesterase 1;PPT1;ortholog	PALMITOYL-PROTEIN THIOESTERASE 1 (PTHR11247:SF36)
HUMAN|HGNC=30009|UniProtKB=Q9UBV8		Peflin;PEF1;ortholog	PEFLIN (PTHR10183:SF361)
HUMAN|HGNC=30042|UniProtKB=Q9H008		Phospholysine phosphohistidine inorganic pyrophosphate phosphatase;LHPP;ortholog	PHOSPHOLYSINE PHOSPHOHISTIDINE INORGANIC PYROPHOSPHATE PHOSPHATASE (PTHR19288:SF58)
HUMAN|HGNC=8765|UniProtKB=O75340		Programmed cell death protein 6;PDCD6;ortholog	PROGRAMMED CELL DEATH PROTEIN 6 (PTHR10183:SF367)
HUMAN|HGNC=8550|UniProtKB=Q9UQ80		Proliferation-associated protein 2G4;PA2G4;ortholog	PROLIFERATION-ASSOCIATED PROTEIN 2G4 (PTHR10804:SF123)
HUMAN|HGNC=18341|UniProtKB=Q9Y2J8	HUMAN|HGNC=18341|UniProtKB=Q9Y2J8	Protein-arginine deiminase type-2;PADI2;ortholog	PROTEIN-ARGININE DEIMINASE TYPE-2 (PTHR10837:SF20)
	HUMAN|HGNC=33782|UniProtKB=Q6GMV3	Putative peptidyl-tRNA hydrolase PTRHD1;PTRHD1;ortholog	PEPTIDYL-TRNA HYDROLASE PTRHD1-RELATED (PTHR11717:SF25)
HUMAN|HGNC=30259|UniProtKB=Q96GD0		Pyridoxal phosphate phosphatase;PDXP;ortholog	PYRIDOXAL PHOSPHATE PHOSPHATASE (PTHR19288:SF47)
	HUMAN|HGNC=9476|UniProtKB=Q92743	Serine protease HTRA1;HTRA1;ortholog	SERINE PROTEASE HTRA1 (PTHR22939:SF107)
	HUMAN|HGNC=14398|UniProtKB=Q9GZT4	Serine racemase;SRR;ortholog	SERINE RACEMASE (PTHR43050:SF1)
HUMAN|HGNC=9304|UniProtKB=P63151	HUMAN|HGNC=9304|UniProtKB=P63151	Serine/threonine-protein phosphatase 2A 55 kDa regulatory subunit B alpha isoform;PPP2R2A;ortholog	SERINE/THREONINE-PROTEIN PHOSPHATASE 2A 55 KDA REGULATORY SUBUNIT B ALPHA ISOFORM (PTHR11871:SF22)
HUMAN|HGNC=9302|UniProtKB=P30153	HUMAN|HGNC=9302|UniProtKB=P30153	Serine/threonine-protein phosphatase 2A 65 kDa regulatory subunit A alpha isoform;PPP2R1A;ortholog	SERINE/THREONINE-PROTEIN PHOSPHATASE 2A 65 KDA REGULATORY SUBUNIT A ALPHA ISOFORM (PTHR10648:SF16)
HUMAN|HGNC=9300|UniProtKB=P62714	HUMAN|HGNC=9300|UniProtKB=P62714	Serine/threonine-protein phosphatase 2A catalytic subunit beta isoform;PPP2CB;ortholog	SERINE/THREONINE-PROTEIN PHOSPHATASE 2A CATALYTIC SUBUNIT BETA ISOFORM (PTHR11668:SF343)
	HUMAN|HGNC=9281|UniProtKB=P62136	Serine/threonine-protein phosphatase PP1-alpha catalytic subunit;PPP1CA;ortholog	SERINE/THREONINE-PROTEIN PHOSPHATASE PP1-ALPHA CATALYTIC SUBUNIT (PTHR11668:SF365)
HUMAN|HGNC=9282|UniProtKB=P62140	HUMAN|HGNC=9282|UniProtKB=P62140	Serine/threonine-protein phosphatase PP1-beta catalytic subunit;PPP1CB;ortholog	SERINE/THREONINE-PROTEIN PHOSPHATASE PP1-BETA CATALYTIC SUBUNIT (PTHR11668:SF346)
HUMAN|HGNC=11740|UniProtKB=P02787		Serotransferrin;TF;ortholog	SEROTRANSFERRIN (PTHR11485:SF35)
	HUMAN|HGNC=14406|UniProtKB=Q9H3S7	Tyrosine-protein phosphatase non-receptor type 23;PTPN23;ortholog	TYROSINE-PROTEIN PHOSPHATASE NON-RECEPTOR TYPE 23 (PTHR19134:SF349)
HUMAN|HGNC=12513|UniProtKB=P09936	HUMAN|HGNC=12513|UniProtKB=P09936	Ubiquitin carboxyl-terminal hydrolase isozyme L1;UCHL1;ortholog	UBIQUITIN CARBOXYL-TERMINAL HYDROLASE ISOZYME L1 (PTHR10589:SF33)
HUMAN|HGNC=854|UniProtKB=P21281	HUMAN|HGNC=854|UniProtKB=P21281	V-type proton ATPase subunit B, brain isoform;ATP6V1B2;ortholog	V-TYPE PROTON ATPASE SUBUNIT B, BRAIN ISOFORM (PTHR43389:SF1)
HUMAN|HGNC=856|UniProtKB=P21283	HUMAN|HGNC=856|UniProtKB=P21283	V-type proton ATPase subunit C 1;ATP6V1C1;ortholog	V-TYPE PROTON ATPASE SUBUNIT C 1 (PTHR10137:SF6)
HUMAN|HGNC=862|UniProtKB=O95670		V-type proton ATPase subunit G 2;ATP6V1G2;ortholog	PROTEIN ATP6V1G2-DDX39B-RELATED (PTHR12713:SF17)
**isomerase (PC00135)**			
HUMAN|HGNC=14169|UniProtKB=Q6P587		Acylpyruvase FAHD1, mitochondrial;FAHD1;ortholog	ACYLPYRUVASE FAHD1, MITOCHONDRIAL (PTHR11820:SF95)
HUMAN|HGNC=13518|UniProtKB=Q9Y696		Chloride intracellular channel protein 4;CLIC4;ortholog	CHLORIDE INTRACELLULAR CHANNEL PROTEIN 4 (PTHR11260:SF443)
HUMAN|HGNC=2732|UniProtKB=P30046		D-dopachrome decarboxylase;DDT;ortholog	D-DOPACHROME DECARBOXYLASE (PTHR11954:SF30)
HUMAN|HGNC=3149|UniProtKB=Q13011		Delta(3,5)-Delta(2,4)-dienoyl-CoA isomerase, mitochondrial;ECH1;ortholog	DELTA(3,5)-DELTA(2,4)-DIENOYL-COA ISOMERASE, MITOCHONDRIAL (PTHR43149:SF3)

HUMAN|HGNC=3213|UniProtKB=P26641	HUMAN|HGNC=3213|UniProtKB=P26641	Elongation factor 1-gamma;EEF1G;ortholog	ELONGATION FACTOR 1-GAMMA (PTHR11260:SF420)
HUMAN|HGNC=3151|UniProtKB=P30084		Enoyl-CoA hydratase, mitochondrial;ECHS1;ortholog	ENOYL-COA HYDRATASE, MITOCHONDRIAL (PTHR11941:SF111)
HUMAN|HGNC=15968|UniProtKB=Q8TB36		Ganglioside-induced differentiation-associated protein 1;GDAP1;ortholog	GANGLIOSIDE-INDUCED DIFFERENTIATION-ASSOCIATED PROTEIN 1 (PTHR11260:SF391)
HUMAN|HGNC=4458|UniProtKB=P06744		Glucose-6-phosphate isomerase;GPI;ortholog	GLUCOSE-6-PHOSPHATE ISOMERASE (PTHR11469:SF17)
HUMAN|HGNC=4799|UniProtKB=Q16836		Hydroxyacyl-coenzyme A dehydrogenase, mitochondrial;HADH;ortholog	HYDROXYACYL-COENZYME A DEHYDROGENASE, MITOCHONDRIAL (PTHR43561:SF3)
HUMAN|HGNC=5157|UniProtKB=P00492		Hypoxanthine-guanine phosphoribosyltransferase;HPRT1;ortholog	HYPOXANTHINE-GUANINE PHOSPHORIBOSYLTRANSFERASE (PTHR43340:SF7)
	HUMAN|HGNC=7526|UniProtKB=P22033	Methylmalonyl-CoA mutase, mitochondrial;MUT;ortholog	METHYLMALONYL-COA MUTASE, MITOCHONDRIAL (PTHR42710:SF1)
HUMAN|HGNC=9253|UniProtKB=P62937	HUMAN|HGNC=9253|UniProtKB=P62937	Peptidyl-prolyl cis-trans isomerase A;PPIA;ortholog	PEPTIDYL-PROLYL CIS-TRANS ISOMERASE-RELATED (PTHR11071:SF354)
HUMAN|HGNC=9255|UniProtKB=P23284	HUMAN|HGNC=9255|UniProtKB=P23284	Peptidyl-prolyl cis-trans isomerase B;PPIB;ortholog	PEPTIDYL-PROLYL CIS-TRANS ISOMERASE B (PTHR11071:SF330)
HUMAN|HGNC=3718|UniProtKB=P26885		Peptidyl-prolyl cis-trans isomerase FKBP2;FKBP2;ortholog	PEPTIDYL-PROLYL CIS-TRANS ISOMERASE FKBP2 (PTHR10516:SF348)
HUMAN|HGNC=3719|UniProtKB=Q00688		Peptidyl-prolyl cis-trans isomerase FKBP3;FKBP3;ortholog	PEPTIDYL-PROLYL CIS-TRANS ISOMERASE FKBP3 (PTHR10516:SF356)
HUMAN|HGNC=3720|UniProtKB=Q02790		Peptidyl-prolyl cis-trans isomerase FKBP4;FKBP4;ortholog	PEPTIDYL-PROLYL CIS-TRANS ISOMERASE FKBP4 (PTHR10516:SF332)
HUMAN|HGNC=3724|UniProtKB=Q14318		Peptidyl-prolyl cis-trans isomerase FKBP8;FKBP8;ortholog	PEPTIDYL-PROLYL CIS-TRANS ISOMERASE FKBP8 (PTHR10516:SF363)
HUMAN|HGNC=8988|UniProtKB=Q13526	HUMAN|HGNC=8988|UniProtKB=Q13526	Peptidyl-prolyl cis-trans isomerase NIMA-interacting 1;PIN1;ortholog	PIN1-LIKE PROTEIN-RELATED (PTHR10657:SF22)
	HUMAN|HGNC=9260|UniProtKB=Q9Y3C6	Peptidyl-prolyl cis-trans isomerase-like 1;PPIL1;ortholog	PEPTIDYL-PROLYL CIS-TRANS ISOMERASE-LIKE 1 (PTHR11071:SF343)
HUMAN|HGNC=8905|UniProtKB=P36871		Phosphoglucomutase-1;PGM1;ortholog	PHOSPHOGLUCOMUTASE-1 (PTHR22573:SF59)
HUMAN|HGNC=9592|UniProtKB=P41222	HUMAN|HGNC=9592|UniProtKB=P41222	Prostaglandin-H2 D-isomerase;PTGDS;ortholog	PROSTAGLANDIN-H2 D-ISOMERASE (PTHR11430:SF114)
	HUMAN|HGNC=14398|UniProtKB=Q9GZT4	Serine racemase;SRR;ortholog	SERINE RACEMASE (PTHR43050:SF1)
	HUMAN|HGNC=26395|UniProtKB=Q8N6N2	Tetratricopeptide repeat protein 9B;TTC9B;ortholog	TETRATRICOPEPTIDE REPEAT PROTEIN 9B (PTHR43811:SF4)
HUMAN|HGNC=4801|UniProtKB=P40939	HUMAN|HGNC=4801|UniProtKB=P40939	Trifunctional enzyme subunit alpha, mitochondrial;HADHA;ortholog	TRIFUNCTIONAL ENZYME SUBUNIT ALPHA, MITOCHONDRIAL (PTHR43612:SF3)
**ligase (PC00142)**			
	HUMAN|HGNC=3418|UniProtKB=P07814	Bifunctional glutamate/proline--tRNA ligase;EPRS;ortholog	BIFUNCTIONAL GLUTAMATE/PROLINE--TRNA LIGASE (PTHR43382:SF3)
HUMAN|HGNC=7432|UniProtKB=P11586	HUMAN|HGNC=7432|UniProtKB=P11586	C-1-tetrahydrofolate synthase, cytoplasmic;MTHFD1;ortholog	C-1-TETRAHYDROFOLATE SYNTHASE, CYTOPLASMIC (PTHR43274:SF1)
HUMAN|HGNC=3149|UniProtKB=Q13011		Delta(3,5)-Delta(2,4)-dienoyl-CoA isomerase, mitochondrial;ECH1;ortholog	DELTA(3,5)-DELTA(2,4)-DIENOYL-COA ISOMERASE, MITOCHONDRIAL (PTHR43149:SF3)
HUMAN|HGNC=3151|UniProtKB=P30084		Enoyl-CoA hydratase, mitochondrial;ECHS1;ortholog	ENOYL-COA HYDRATASE, MITOCHONDRIAL (PTHR11941:SF111)
HUMAN|HGNC=3594|UniProtKB=P49327	HUMAN|HGNC=3594|UniProtKB=P49327	Fatty acid synthase;FASN;ortholog	FATTY ACID SYNTHASE (PTHR43775:SF2)
	HUMAN|HGNC=4311|UniProtKB=P48506	Glutamate--cysteine ligase catalytic subunit;GCLC;ortholog	GLUTAMATE--CYSTEINE LIGASE CATALYTIC SUBUNIT (PTHR11164:SF2)

	HUMAN|HGNC=4312|UniProtKB=P48507	Glutamate--cysteine ligase regulatory subunit;GCLM;ortholog	GLUTAMATE--CYSTEINE LIGASE REGULATORY SUBUNIT (PTHR13295:SF5)
HUMAN|HGNC=4341|UniProtKB=P15104		Glutamine synthetase;GLUL;ortholog	GLUTAMINE SYNTHETASE (PTHR20852:SF67)
HUMAN|HGNC=29685|UniProtKB=Q9NSE4	HUMAN|HGNC=29685|UniProtKB=Q9NSE4	Isoleucine--tRNA ligase, mitochondrial;IARS2;ortholog	ISOLEUCINE--TRNA LIGASE, MITOCHONDRIAL (PTHR42765:SF3)
HUMAN|HGNC=29207|UniProtKB=Q8N1G4	HUMAN|HGNC=29207|UniProtKB=Q8N1G4	Leucine-rich repeat-containing protein 47;LRRC47;ortholog	LEUCINE-RICH REPEAT-CONTAINING PROTEIN 47 (PTHR10947:SF7)
	HUMAN|HGNC=6215|UniProtKB=Q15046	Lysine--tRNA ligase;KARS;ortholog	LYSINE--TRNA LIGASE (PTHR42918:SF2)
HUMAN|HGNC=3592|UniProtKB=Q9Y285	HUMAN|HGNC=3592|UniProtKB=Q9Y285	Phenylalanine--tRNA ligase alpha subunit;FARSA;ortholog	PHENYLALANINE--TRNA LIGASE ALPHA SUBUNIT (PTHR11538:SF49)
HUMAN|HGNC=17800|UniProtKB=Q9NSD9	HUMAN|HGNC=17800|UniProtKB=Q9NSD9	Phenylalanine--tRNA ligase beta subunit;FARSB;ortholog	PHENYLALANINE--TRNA LIGASE BETA SUBUNIT (PTHR10947:SF5)
	HUMAN|HGNC=17095|UniProtKB=Q15031	Probable leucine--tRNA ligase, mitochondrial;LARS2;ortholog	LEUCINE--TRNA LIGASE, MITOCHONDRIAL-RELATED (PTHR43740:SF2)
HUMAN|HGNC=8636|UniProtKB=P11498	HUMAN|HGNC=8636|UniProtKB=P11498	Pyruvate carboxylase, mitochondrial;PC;ortholog	PYRUVATE CARBOXYLASE, MITOCHONDRIAL (PTHR43778:SF4)
HUMAN|HGNC=9462|UniProtKB=P60891		Ribose-phosphate pyrophosphokinase 1;PRPS1;ortholog	RIBOSE-PHOSPHATE PYROPHOSPHOKINASE 1 (PTHR10210:SF68)
	HUMAN|HGNC=11450|UniProtKB=Q96I99	Succinyl-CoA ligase [GDP-forming] subunit beta, mitochondrial;SUCLG2;ortholog	SUCCINYL-COA LIGASE [GDP-FORMING] SUBUNIT BETA, MITOCHONDRIAL (PTHR11815:SF7)
HUMAN|HGNC=15974|UniProtKB=Q9C040	HUMAN|HGNC=15974|UniProtKB=Q9C040	Tripartite motif-containing protein 2;TRIM2;ortholog	TRIPARTITE MOTIF-CONTAINING PROTEIN 2 (PTHR24103:SF446)
	HUMAN|HGNC=25581|UniProtKB=A0AVT1	Ubiquitin-like modifier-activating enzyme 6;UBA6;ortholog	UBIQUITIN-LIKE MODIFIER-ACTIVATING ENZYME 6 (PTHR10953:SF180)
**lyase (PC00144)**			
HUMAN|HGNC=118|UniProtKB=Q99798	HUMAN|HGNC=118|UniProtKB=Q99798	Aconitate hydratase, mitochondrial;ACO2;ortholog	ACONITATE HYDRATASE, MITOCHONDRIAL (PTHR43160:SF2)
HUMAN|HGNC=13238|UniProtKB=Q9HDC9		Adipocyte plasma membrane-associated protein;APMAP;ortholog	ADIPOCYTE PLASMA MEMBRANE-ASSOCIATED PROTEIN (PTHR10426:SF47)
HUMAN|HGNC=3350|UniProtKB=P06733	HUMAN|HGNC=3350|UniProtKB=P06733	Alpha-enolase;ENO1;ortholog	ALPHA-ENOLASE (PTHR11902:SF26)
HUMAN|HGNC=1373|UniProtKB=P00918		Carbonic anhydrase 2;CA2;ortholog	CARBONIC ANHYDRASE 2 (PTHR18952:SF159)
HUMAN|HGNC=1375|UniProtKB=P22748		Carbonic anhydrase 4;CA4;ortholog	CARBONIC ANHYDRASE 4 (PTHR18952:SF153)
HUMAN|HGNC=3149|UniProtKB=Q13011		Delta(3,5)-Delta(2,4)-dienoyl-CoA isomerase, mitochondrial;ECH1;ortholog	DELTA(3,5)-DELTA(2,4)-DIENOYL-COA ISOMERASE, MITOCHONDRIAL (PTHR43149:SF3)
HUMAN|HGNC=3151|UniProtKB=P30084		Enoyl-CoA hydratase, mitochondrial;ECHS1;ortholog	ENOYL-COA HYDRATASE, MITOCHONDRIAL (PTHR11941:SF111)
HUMAN|HGNC=3353|UniProtKB=P09104		Gamma-enolase;ENO2;ortholog	GAMMA-ENOLASE (PTHR11902:SF21)
	HUMAN|HGNC=4093|UniProtKB=Q05329	Glutamate decarboxylase 2;GAD2;ortholog	GLUTAMATE DECARBOXYLASE 2 (PTHR11999:SF115)
HUMAN|HGNC=4799|UniProtKB=Q16836		Hydroxyacyl-coenzyme A dehydrogenase, mitochondrial;HADH;ortholog	HYDROXYACYL-COENZYME A DEHYDROGENASE, MITOCHONDRIAL (PTHR43561:SF3)
	HUMAN|HGNC=5007|UniProtKB=Q01581	Hydroxymethylglutaryl-CoA synthase, cytoplasmic;HMGCS1;ortholog	HYDROXYMETHYLGLUTARYL-COA SYNTHASE, CYTOPLASMIC (PTHR43323:SF6)
HUMAN|HGNC=6984|UniProtKB=P23368		NAD-dependent malic enzyme, mitochondrial;ME2;ortholog	NAD-DEPENDENT MALIC ENZYME, MITOCHONDRIAL (PTHR23406:SF50)
	HUMAN|HGNC=17981|UniProtKB=O00442	RNA 3'-terminal phosphate cyclase;RTCA;ortholog	RNA 3'-TERMINAL PHOSPHATE CYCLASE (PTHR11096:SF2)
	HUMAN|HGNC=14398|UniProtKB=Q9GZT4	Serine racemase;SRR;ortholog	SERINE RACEMASE (PTHR43050:SF1)

HUMAN|HGNC=11834|UniProtKB=P29401		Transketolase;TKT;ortholog	TRANSKETOLASE (PTHR43195:SF3)
HUMAN|HGNC=4801|UniProtKB=P40939	HUMAN|HGNC=4801|UniProtKB=P40939	Trifunctional enzyme subunit alpha, mitochondrial;HADHA;ortholog	TRIFUNCTIONAL ENZYME SUBUNIT ALPHA, MITOCHONDRIAL (PTHR43612:SF3)

membrane traffic protein (PC00150)

	HUMAN|HGNC=7641|UniProtKB=P54920	Alpha-soluble NSF attachment protein;NAPA;ortholog	ALPHA-SOLUBLE NSF ATTACHMENT PROTEIN (PTHR13768:SF20)
	HUMAN|HGNC=15751|UniProtKB=Q9H115	Beta-soluble NSF attachment protein;NAPB;ortholog	BETA-SOLUBLE NSF ATTACHMENT PROTEIN (PTHR13768:SF19)
HUMAN|HGNC=15751|UniProtKB=Q9H115		Beta-soluble NSF attachment protein;NAPB;ortholog	BETA-SOLUBLE NSF ATTACHMENT PROTEIN (PTHR13768:SF19)
HUMAN|HGNC=11140|UniProtKB=Q16143		Beta-synuclein;SNCB;ortholog	BETA-SYNUCLEIN (PTHR13820:SF13)
	HUMAN|HGNC=649|UniProtKB=P48444	Coatomer subunit delta;ARCN1;ortholog	COATOMER SUBUNIT DELTA (PTHR10121:SF2)
HUMAN|HGNC=2316|UniProtKB=O75131		Copine-3;CPNE3;ortholog	COPINE-3 (PTHR10857:SF58)
HUMAN|HGNC=2317|UniProtKB=Q96A23	HUMAN|HGNC=2317|UniProtKB=Q96A23	Copine-4;CPNE4;ortholog	COPINE-4 (PTHR10857:SF78)
HUMAN|HGNC=2318|UniProtKB=Q9HCH3	HUMAN|HGNC=2318|UniProtKB=Q9HCH3	Copine-5;CPNE5;ortholog	COPINE-5 (PTHR10857:SF69)
HUMAN|HGNC=2319|UniProtKB=O95741	HUMAN|HGNC=2319|UniProtKB=O95741	Copine-6;CPNE6;ortholog	COPINE-6 (PTHR10857:SF66)
HUMAN|HGNC=3244|UniProtKB=Q9NZN3	HUMAN|HGNC=3244|UniProtKB=Q9NZN3	EH domain-containing protein 3;EHD3;ortholog	EH DOMAIN-CONTAINING PROTEIN 3 (PTHR11216:SF88)
HUMAN|HGNC=13799|UniProtKB=P30040		Endoplasmic reticulum resident protein 29;ERP29;ortholog	ENDOPLASMIC RETICULUM RESIDENT PROTEIN 29 (PTHR12211:SF1)
HUMAN|HGNC=30389|UniProtKB=Q96A65		Exocyst complex component 4;EXOC4;ortholog	EXOCYST COMPLEX COMPONENT 4 (PTHR14146:SF1)
HUMAN|HGNC=7642|UniProtKB=Q99747		Gamma-soluble NSF attachment protein;NAPG;ortholog	GAMMA-SOLUBLE NSF ATTACHMENT PROTEIN (PTHR13768:SF18)
HUMAN|HGNC=30904|UniProtKB=O60763		General vesicular transport factor p115;USO1;ortholog	GENERAL VESICULAR TRANSPORT FACTOR P115 (PTHR10013:SF1)
HUMAN|HGNC=6501|UniProtKB=P13473		Lysosome-associated membrane glycoprotein 2;LAMP2;ortholog	LYSOSOME-ASSOCIATED MEMBRANE GLYCOPROTEIN 2 (PTHR11506:SF40)
HUMAN|HGNC=18001|UniProtKB=O96008		Mitochondrial import receptor subunit TOM40 homolog;TOMM40;ortholog	MITOCHONDRIAL IMPORT RECEPTOR SUBUNIT TOM40 HOMOLOG (PTHR10802:SF8)
HUMAN|HGNC=30285|UniProtKB=Q7L099	HUMAN|HGNC=30285|UniProtKB=Q7L099	Protein RUFY3;RUFY3;ortholog	PROTEIN RUFY3 (PTHR22835:SF332)
HUMAN|HGNC=14085|UniProtKB=Q9NQC3		Reticulon-4;RTN4;ortholog	RETICULON-4 (PTHR10994:SF108)
HUMAN|HGNC=11172|UniProtKB=Q13596		Sorting nexin-1;SNX1;ortholog	SORTING NEXIN-1 (PTHR10555:SF192)
	HUMAN|HGNC=11174|UniProtKB=O60493	Sorting nexin-3;SNX3;ortholog	SORTING NEXIN-3 (PTHR10555:SF161)
	HUMAN|HGNC=11175|UniProtKB=O95219	Sorting nexin-4;SNX4;ortholog	SORTING NEXIN-4 (PTHR10555:SF199)
	HUMAN|HGNC=11496|UniProtKB=O14994	Synapsin-3;SYN3;ortholog	SYNAPSIN-3 (PTHR10841:SF25)
HUMAN|HGNC=11501|UniProtKB=O43761		Synaptogyrin-3;SYNGR3;ortholog	SYNAPTOGYRIN-3 (PTHR10838:SF28)
HUMAN|HGNC=11506|UniProtKB=P08247	HUMAN|HGNC=11506|UniProtKB=P08247	Synaptophysin;SYP;ortholog	SYNAPTOPHYSIN (PTHR10306:SF25)
HUMAN|HGNC=11132|UniProtKB=P60880	HUMAN|HGNC=11132|UniProtKB=P60880	Synaptosomal-associated protein 25;SNAP25;ortholog	SYNAPTOSOMAL-ASSOCIATED PROTEIN 25 (PTHR19305:SF17)
HUMAN|HGNC=11514|UniProtKB=O43581	HUMAN|HGNC=11514|UniProtKB=O43581	Synaptotagmin-7;SYT7;ortholog	SYNAPTOTAGMIN-7 (PTHR10024:SF342)
HUMAN|HGNC=11433|UniProtKB=Q16623		Syntaxin-1A;STX1A;ortholog	SYNTAXIN-1A (PTHR19957:SF187)

HUMAN|HGNC=11444|UniProtKB=P61764	HUMAN|HGNC=11444|UniProtKB=P61764	Syntaxin-binding protein 1;STXBP1;ortholog	SYNTAXIN-BINDING PROTEIN 1 (PTHR11679:SF54)
HUMAN|HGNC=11446|UniProtKB=O00186		Syntaxin-binding protein 3;STXBP3;ortholog	SYNTAXIN-BINDING PROTEIN 3 (PTHR11679:SF44)
HUMAN|HGNC=28119|UniProtKB=Q4G0F5		Vacuolar protein sorting-associated protein 26B;VPS26B;ortholog	VACUOLAR PROTEIN SORTING-ASSOCIATED PROTEIN 26B (PTHR12233:SF10)
HUMAN|HGNC=13487|UniProtKB=Q96QK1	HUMAN|HGNC=13487|UniProtKB=Q96QK1	Vacuolar protein sorting-associated protein 35;VPS35;ortholog	VACUOLAR PROTEIN SORTING-ASSOCIATED PROTEIN 35 (PTHR11099:SF3)
HUMAN|HGNC=12648|UniProtKB=Q9P0L0	HUMAN|HGNC=12648|UniProtKB=Q9P0L0	Vesicle-associated membrane protein-associated protein A;VAPA;ortholog	VESICLE-ASSOCIATED MEMBRANE PROTEIN-ASSOCIATED PROTEIN A (PTHR10809:SF64)
HUMAN|HGNC=12649|UniProtKB=O95292		Vesicle-associated membrane protein-associated protein B/C;VAPB;ortholog	VESICLE-ASSOCIATED MEMBRANE PROTEIN-ASSOCIATED PROTEIN B/C (PTHR10809:SF86)
HUMAN|HGNC=10700|UniProtKB=O75396	HUMAN|HGNC=10700|UniProtKB=O75396	Vesicle-trafficking protein SEC22b;SEC22B;ortholog	VESICLE-TRAFFICKING PROTEIN SEC22B (PTHR21136:SF160)
**nucleic acid binding (PC00171)**			
	HUMAN|HGNC=10387|UniProtKB=P62263	40S ribosomal protein S14;RPS14;ortholog	40S RIBOSOMAL PROTEIN S14 (PTHR11759:SF13)
HUMAN|HGNC=10401|UniProtKB=P62269	HUMAN|HGNC=10401|UniProtKB=P62269	40S ribosomal protein S18;RPS18;ortholog	40S RIBOSOMAL PROTEIN S18 (PTHR10871:SF15)
HUMAN|HGNC=10402|UniProtKB=P39019		40S ribosomal protein S19;RPS19;ortholog	40S RIBOSOMAL PROTEIN S19 (PTHR11710:SF7)
HUMAN|HGNC=10405|UniProtKB=P60866		40S ribosomal protein S20;RPS20;ortholog	40S RIBOSOMAL PROTEIN S20 (PTHR11700:SF13)
HUMAN|HGNC=10420|UniProtKB=P23396	HUMAN|HGNC=10420|UniProtKB=P23396	40S ribosomal protein S3;RPS3;ortholog	40S RIBOSOMAL PROTEIN S3 (PTHR11760:SF26)
HUMAN|HGNC=10424|UniProtKB=P62701	HUMAN|HGNC=10424|UniProtKB=P62701	40S ribosomal protein S4, X isoform;RPS4X;ortholog	40S RIBOSOMAL PROTEIN S4, X ISOFORM (PTHR11581:SF21)
HUMAN|HGNC=10440|UniProtKB=P62081		40S ribosomal protein S7;RPS7;ortholog	40S RIBOSOMAL PROTEIN S7 (PTHR11278:SF5)
HUMAN|HGNC=10299|UniProtKB=P62906		60S ribosomal protein L10a;RPL10A;ortholog	60S RIBOSOMAL PROTEIN L10A (PTHR23105:SF87)
HUMAN|HGNC=10302|UniProtKB=P30050	HUMAN|HGNC=10302|UniProtKB=P30050	60S ribosomal protein L12;RPL12;ortholog	60S RIBOSOMAL PROTEIN L12 (PTHR11661:SF11)
	HUMAN|HGNC=10304|UniProtKB=P40429	60S ribosomal protein L13a;RPL13A;ortholog	60S RIBOSOMAL PROTEIN L13A-RELATED (PTHR11545:SF8)
	HUMAN|HGNC=10316|UniProtKB=P62829	60S ribosomal protein L23;RPL23;ortholog	60S RIBOSOMAL PROTEIN L23 (PTHR11761:SF20)
	HUMAN|HGNC=10327|UniProtKB=P61254	60S ribosomal protein L26;RPL26;ortholog	60S RIBOSOMAL PROTEIN L26 (PTHR11143:SF16)
	HUMAN|HGNC=10328|UniProtKB=P61353	60S ribosomal protein L27;RPL27;ortholog	60S RIBOSOMAL PROTEIN L27 (PTHR10497:SF3)
	HUMAN|HGNC=10332|UniProtKB=P39023	60S ribosomal protein L3;RPL3;ortholog	60S RIBOSOMAL PROTEIN L3 (PTHR11363:SF11)
HUMAN|HGNC=10362|UniProtKB=Q02878	HUMAN|HGNC=10362|UniProtKB=Q02878	60S ribosomal protein L6;RPL6;ortholog	60S RIBOSOMAL PROTEIN L6 (PTHR10715:SF4)
HUMAN|HGNC=10364|UniProtKB=P62424		60S ribosomal protein L7a;RPL7A;ortholog	60S RIBOSOMAL PROTEIN L7A (PTHR23105:SF92)
HUMAN|HGNC=10990|UniProtKB=P12235	HUMAN|HGNC=10990|UniProtKB=P12235	ADP/ATP translocase 1;SLC25A4;ortholog	ADP/ATP TRANSLOCASE 1 (PTHR24089:SF506)
HUMAN|HGNC=10991|UniProtKB=P05141	HUMAN|HGNC=10991|UniProtKB=P05141	ADP/ATP translocase 2;SLC25A5;ortholog	ADP/ATP TRANSLOCASE 2 (PTHR24089:SF446)
HUMAN|HGNC=10992|UniProtKB=P12236	HUMAN|HGNC=10992|UniProtKB=P12236	ADP/ATP translocase 3;SLC25A6;ortholog	ADP/ATP TRANSLOCASE 3 (PTHR24089:SF411)
HUMAN|HGNC=20|UniProtKB=P49588	HUMAN|HGNC=20|UniProtKB=P49588	Alanine--tRNA ligase, cytoplasmic;AARS;ortholog	ALANINE--TRNA LIGASE, CYTOPLASMIC (PTHR11777:SF24)
	HUMAN|HGNC=28417|UniProtKB=Q9BTE6	Alanyl-tRNA editing protein Aarsd1;AARSD1;ortholog	ALANYL-TRNA EDITING PROTEIN AARSD1 (PTHR43462:SF1)
	HUMAN|HGNC=43946|UniProtKB=Q9BTE6	Alanyl-tRNA editing protein Aarsd1;AARSD1;ortholog	ALANYL-TRNA EDITING PROTEIN AARSD1 (PTHR43462:SF1)

HUMAN|HGNC=6833|UniProtKB=P21397	HUMAN|HGNC=6833|UniProtKB=P21397	Amine oxidase [flavin-containing] A;MAOA;ortholog	AMINE OXIDASE [FLAVIN-CONTAINING] A (PTHR43563:SF4)
HUMAN|HGNC=6834|UniProtKB=P27338	HUMAN|HGNC=6834|UniProtKB=P27338	Amine oxidase [flavin-containing] B;MAOB;ortholog	AMINE OXIDASE [FLAVIN-CONTAINING] B (PTHR43563:SF6)
HUMAN|HGNC=823|UniProtKB=P25705	HUMAN|HGNC=823|UniProtKB=P25705	ATP synthase subunit alpha, mitochondrial;ATP5A1;ortholog	ATP SYNTHASE SUBUNIT ALPHA, MITOCHONDRIAL (PTHR43089:SF1)
HUMAN|HGNC=830|UniProtKB=P06576	HUMAN|HGNC=830|UniProtKB=P06576	ATP synthase subunit beta, mitochondrial;ATP5B;ortholog	ATP SYNTHASE SUBUNIT BETA, MITOCHONDRIAL (PTHR15184:SF59)
HUMAN|HGNC=2750|UniProtKB=Q08211	HUMAN|HGNC=2750|UniProtKB=Q08211	ATP-dependent RNA helicase A;DHX9;ortholog	ATP-DEPENDENT RNA HELICASE A (PTHR18934:SF176)
	HUMAN|HGNC=2734|UniProtKB=Q92499	ATP-dependent RNA helicase DDX1;DDX1;ortholog	ATP-DEPENDENT RNA HELICASE DDX1 (PTHR24031:SF482)
HUMAN|HGNC=10982|UniProtKB=O75746	HUMAN|HGNC=10982|UniProtKB=O75746	Calcium-binding mitochondrial carrier protein Aralar1;SLC25A12;ortholog	CALCIUM-BINDING MITOCHONDRIAL CARRIER PROTEIN ARALAR1 (PTHR24089:SF503)
HUMAN|HGNC=13518|UniProtKB=Q9Y696		Chloride intracellular channel protein 4;CLIC4;ortholog	CHLORIDE INTRACELLULAR CHANNEL PROTEIN 4 (PTHR11260:SF443)
	HUMAN|HGNC=14453|UniProtKB=Q9P0M6	Core histone macro-H2A.2;H2AFY2;ortholog	CORE HISTONE MACRO-H2A.2 (PTHR23430:SF124)
HUMAN|HGNC=16991|UniProtKB=Q07065		Cytoskeleton-associated protein 4;CKAP4;ortholog	CYTOSKELETON-ASSOCIATED PROTEIN 4 (PTHR18937:SF294)
	HUMAN|HGNC=24035|UniProtKB=Q9UKG1	DCC-interacting protein 13-alpha;APPL1;ortholog	DCC-INTERACTING PROTEIN 13-ALPHA (PTHR23180:SF350)
HUMAN|HGNC=3189|UniProtKB=P68104	HUMAN|HGNC=3189|UniProtKB=P68104	Elongation factor 1-alpha 1;EEF1A1;ortholog	ELONGATION FACTOR 1-ALPHA 1 (PTHR23115:SF216)
HUMAN|HGNC=3192|UniProtKB=Q05639	HUMAN|HGNC=3192|UniProtKB=Q05639	Elongation factor 1-alpha 2;EEF1A2;ortholog	ELONGATION FACTOR 1-ALPHA 2 (PTHR23115:SF203)
HUMAN|HGNC=3213|UniProtKB=P26641	HUMAN|HGNC=3213|UniProtKB=P26641	Elongation factor 1-gamma;EEF1G;ortholog	ELONGATION FACTOR 1-GAMMA (PTHR11260:SF420)
HUMAN|HGNC=3214|UniProtKB=P13639	HUMAN|HGNC=3214|UniProtKB=P13639	Elongation factor 2;EEF2;ortholog	ELONGATION FACTOR 2 (PTHR42908:SF8)
HUMAN|HGNC=12420|UniProtKB=P49411	HUMAN|HGNC=12420|UniProtKB=P49411	Elongation factor Tu, mitochondrial;TUFM;ortholog	ELONGATION FACTOR TU, MITOCHONDRIAL (PTHR43721:SF7)
HUMAN|HGNC=28963|UniProtKB=Q15006		ER membrane protein complex subunit 2;EMC2;ortholog	ER MEMBRANE PROTEIN COMPLEX SUBUNIT 2 (PTHR12760:SF1)
HUMAN|HGNC=3282|UniProtKB=P60842		Eukaryotic initiation factor 4A-I;EIF4A1;ortholog	EUKARYOTIC INITIATION FACTOR 4A-I (PTHR24031:SF379)
HUMAN|HGNC=3284|UniProtKB=Q14240	HUMAN|HGNC=3284|UniProtKB=Q14240	Eukaryotic initiation factor 4A-II;EIF4A2;ortholog	EUKARYOTIC INITIATION FACTOR 4A-II (PTHR24031:SF418)
	HUMAN|HGNC=3267|UniProtKB=P41091	Eukaryotic translation initiation factor 2 subunit 3;EIF2S3;ortholog	EUKARYOTIC TRANSLATION INITIATION FACTOR 2 SUBUNIT 3 (PTHR42854:SF1)
HUMAN|HGNC=3275|UniProtKB=O00303		Eukaryotic translation initiation factor 3 subunit F;EIF3F;ortholog	EUKARYOTIC TRANSLATION INITIATION FACTOR 3 SUBUNIT F (PTHR10540:SF14)
	HUMAN|HGNC=3272|UniProtKB=Q13347	Eukaryotic translation initiation factor 3 subunit I;EIF3I;ortholog	EUKARYOTIC TRANSLATION INITIATION FACTOR 3 SUBUNIT I (PTHR19877:SF6)
HUMAN|HGNC=3299|UniProtKB=P55010		Eukaryotic translation initiation factor 5;EIF5;ortholog	EUKARYOTIC TRANSLATION INITIATION FACTOR 5 (PTHR23001:SF11)
	HUMAN|HGNC=12826|UniProtKB=O43592	Exportin-T;XPOT;ortholog	EXPORTIN-T (PTHR15952:SF12)
HUMAN|HGNC=15968|UniProtKB=Q8TB36		Ganglioside-induced differentiation-associated protein 1;GDAP1;ortholog	GANGLIOSIDE-INDUCED DIFFERENTIATION-ASSOCIATED PROTEIN 1 (PTHR11260:SF391)
HUMAN|HGNC=4413|UniProtKB=P36915		Guanine nucleotide-binding protein-like 1;GNL1;ortholog	GUANINE NUCLEOTIDE-BINDING PROTEIN-LIKE 1 (PTHR11089:SF55)
HUMAN|HGNC=5030|UniProtKB=Q13151	HUMAN|HGNC=5030|UniProtKB=Q13151	Heterogeneous nuclear ribonucleoprotein A0;HNRNPA0;ortholog	HETEROGENEOUS NUCLEAR RIBONUCLEOPROTEIN A0-RELATED (PTHR24012:SF515)

	HUMAN|HGNC=5039|UniProtKB=P52597	Heterogeneous nuclear ribonucleoprotein F;HNRNPF;ortholog	HETEROGENEOUS NUCLEAR RIBONUCLEOPROTEIN F (PTHR13976:SF47)
HUMAN|HGNC=5042|UniProtKB=P55795	HUMAN|HGNC=5042|UniProtKB=P55795	Heterogeneous nuclear ribonucleoprotein H2;HNRNPH2;ortholog	HETEROGENEOUS NUCLEAR RIBONUCLEOPROTEIN H2 (PTHR13976:SF55)
HUMAN|HGNC=18468|UniProtKB=Q9NQE9		Histidine triad nucleotide-binding protein 3;HINT3;ortholog	HISTIDINE TRIAD NUCLEOTIDE-BINDING PROTEIN 3 (PTHR12486:SF6)
HUMAN|HGNC=4716|UniProtKB=P16403	HUMAN|HGNC=4716|UniProtKB=P16403	Histone H1.2;HIST1H1C;ortholog	HISTONE H1.2 (PTHR11467:SF76)
HUMAN|HGNC=4722|UniProtKB=Q92522	HUMAN|HGNC=4722|UniProtKB=Q92522	Histone H1x;H1FX;ortholog	HISTONE H1X (PTHR11467:SF77)
HUMAN|HGNC=13671|UniProtKB=Q96KK5	HUMAN|HGNC=13671|UniProtKB=Q96KK5	Histone H2A type 1-H;HIST1H2AH;ortholog	HISTONE H2A TYPE 1-H (PTHR23430:SF160)
HUMAN|HGNC=20508|UniProtKB=Q8IUE6	HUMAN|HGNC=20508|UniProtKB=Q8IUE6	Histone H2A type 2-B;HIST2H2AB;ortholog	HISTONE H2A TYPE 2-B (PTHR23430:SF154)
HUMAN|HGNC=13954|UniProtKB=O60814	HUMAN|HGNC=13954|UniProtKB=O60814	Histone H2B type 1-K;HIST1H2BK;ortholog	HISTONE H2B TYPE 1-K-RELATED (PTHR23428:SF110)
HUMAN|HGNC=20510|UniProtKB=P62805	HUMAN|HGNC=20510|UniProtKB=P62805	Histone H4;HIST1H4A;ortholog	HISTONE H4 (PTHR10484:SF122)
HUMAN|HGNC=29607|UniProtKB=P62805	HUMAN|HGNC=29607|UniProtKB=P62805	Histone H4;HIST1H4A;ortholog	HISTONE H4 (PTHR10484:SF152)
HUMAN|HGNC=4781|UniProtKB=P62805	HUMAN|HGNC=4781|UniProtKB=P62805	Histone H4;HIST1H4A;ortholog	HISTONE H4 (PTHR10484:SF150)
HUMAN|HGNC=4782|UniProtKB=P62805	HUMAN|HGNC=4782|UniProtKB=P62805	Histone H4;HIST1H4A;ortholog	HISTONE H4 (PTHR10484:SF137)
HUMAN|HGNC=4783|UniProtKB=P62805	HUMAN|HGNC=4783|UniProtKB=P62805	Histone H4;HIST1H4A;ortholog	HISTONE H4 (PTHR10484:SF156)
HUMAN|HGNC=4784|UniProtKB=P62805	HUMAN|HGNC=4784|UniProtKB=P62805	Histone H4;HIST1H4A;ortholog	HISTONE H4 (PTHR10484:SF91)
HUMAN|HGNC=4785|UniProtKB=P62805	HUMAN|HGNC=4785|UniProtKB=P62805	Histone H4;HIST1H4A;ortholog	HISTONE H4 (PTHR10484:SF144)
HUMAN|HGNC=4787|UniProtKB=P62805	HUMAN|HGNC=4787|UniProtKB=P62805	Histone H4;HIST1H4A;ortholog	HISTONE H4 (PTHR10484:SF101)
HUMAN|HGNC=4788|UniProtKB=P62805	HUMAN|HGNC=4788|UniProtKB=P62805	Histone H4;HIST1H4A;ortholog	HISTONE H4 (PTHR10484:SF127)
HUMAN|HGNC=4789|UniProtKB=P62805	HUMAN|HGNC=4789|UniProtKB=P62805	Histone H4;HIST1H4A;ortholog	HISTONE H4 (PTHR10484:SF129)
HUMAN|HGNC=4790|UniProtKB=P62805	HUMAN|HGNC=4790|UniProtKB=P62805	Histone H4;HIST1H4A;ortholog	HISTONE H4 (PTHR10484:SF113)
HUMAN|HGNC=4791|UniProtKB=P62805	HUMAN|HGNC=4791|UniProtKB=P62805	Histone H4;HIST1H4A;ortholog	HISTONE H4 (PTHR10484:SF107)
HUMAN|HGNC=4793|UniProtKB=P62805	HUMAN|HGNC=4793|UniProtKB=P62805	Histone H4;HIST1H4A;ortholog	HISTONE H4 (PTHR10484:SF134)
HUMAN|HGNC=4794|UniProtKB=P62805	HUMAN|HGNC=4794|UniProtKB=P62805	Histone H4;HIST1H4A;ortholog	HISTONE H4 (PTHR10484:SF123)
HUMAN|HGNC=15714|UniProtKB=P42704	HUMAN|HGNC=15714|UniProtKB=P42704	Leucine-rich PPR motif-containing protein, mitochondrial;LRPPRC;ortholog	LEUCINE-RICH PPR MOTIF-CONTAINING PROTEIN, MITOCHONDRIAL (PTHR24015:SF1304)
HUMAN|HGNC=29207|UniProtKB=Q8N1G4	HUMAN|HGNC=29207|UniProtKB=Q8N1G4	Leucine-rich repeat-containing protein 47;LRRC47;ortholog	LEUCINE-RICH REPEAT-CONTAINING PROTEIN 47 (PTHR10947:SF7)
	HUMAN|HGNC=6215|UniProtKB=Q15046	Lysine--tRNA ligase;KARS;ortholog	LYSINE--TRNA LIGASE (PTHR42918:SF2)
	HUMAN|HGNC=6912|UniProtKB=P43243	Matrin-3;MATR3;ortholog	MATRIN-3 (PTHR15592:SF12)
	HUMAN|HGNC=6990|UniProtKB=P51608	Methyl-CpG-binding protein 2;MECP2;ortholog	METHYL-CPG-BINDING PROTEIN 2 (PTHR15074:SF7)
HUMAN|HGNC=19954|UniProtKB=Q9H936	HUMAN|HGNC=19954|UniProtKB=Q9H936	Mitochondrial glutamate carrier 1;SLC25A22;ortholog	MITOCHONDRIAL GLUTAMATE CARRIER 1 (PTHR24089:SF598)
	HUMAN|HGNC=10988|UniProtKB=Q9H1K4	Mitochondrial glutamate carrier 2;SLC25A18;ortholog	MITOCHONDRIAL GLUTAMATE CARRIER 2 (PTHR24089:SF482)
	HUMAN|HGNC=15911|UniProtKB=O00567	Nucleolar protein 56;NOP56;ortholog	NUCLEOLAR PROTEIN 56 (PTHR10894:SF8)

HUMAN|HGNC=7667|UniProtKB=P19338		Nucleolin;NCL;ortholog	NUCLEOLIN (PTHR24012:SF568)
HUMAN|HGNC=17800|UniProtKB=Q9NSD9	HUMAN|HGNC=17800|UniProtKB=Q9NSD9	Phenylalanine--tRNA ligase beta subunit;FARSB;ortholog	PHENYLALANINE--TRNA LIGASE BETA SUBUNIT (PTHR10947:SF5)
HUMAN|HGNC=270|UniProtKB=P09874	HUMAN|HGNC=270|UniProtKB=P09874	Poly [ADP-ribose] polymerase 1;PARP1;ortholog	POLY [ADP-RIBOSE] POLYMERASE 1 (PTHR10459:SF83)
HUMAN|HGNC=8647|UniProtKB=Q15365	HUMAN|HGNC=8647|UniProtKB=Q15365	Poly(rC)-binding protein 1;PCBP1;ortholog	POLY(RC)-BINDING PROTEIN 1 (PTHR10288:SF191)
HUMAN|HGNC=8550|UniProtKB=Q9UQ80		Proliferation-associated protein 2G4;PA2G4;ortholog	PROLIFERATION-ASSOCIATED PROTEIN 2G4 (PTHR10804:SF123)
HUMAN|HGNC=49181|UniProtKB=S4R435		Protein RPS10-NUDT3 (Fragment);RPS10-NUDT3;ortholog	SUBFAMILY NOT NAMED (PTHR12146:SF10)
HUMAN|HGNC=20517|UniProtKB=Q6DRA6	HUMAN|HGNC=20517|UniProtKB=Q6DRA6	Putative histone H2B type 2-D;HIST2H2BD;ortholog	HISTONE H2B TYPE 2-C-RELATED (PTHR23428:SF131)
HUMAN|HGNC=10074|UniProtKB=P13489	HUMAN|HGNC=10074|UniProtKB=P13489	Ribonuclease inhibitor;RNH1;ortholog	RIBONUCLEASE INHIBITOR (PTHR24112:SF52)
	HUMAN|HGNC=17981|UniProtKB=O00442	RNA 3'-terminal phosphate cyclase;RTCA;ortholog	RNA 3'-TERMINAL PHOSPHATE CYCLASE (PTHR11096:SF2)
	HUMAN|HGNC=7887|UniProtKB=Q9UNW9	RNA-binding protein Nova-2;NOVA2;ortholog	RNA-BINDING PROTEIN NOVA-2 (PTHR10288:SF200)
	HUMAN|HGNC=11362|UniProtKB=P42224	Signal transducer and activator of transcription 1-alpha/beta;STAT1;ortholog	SIGNAL TRANSDUCER AND ACTIVATOR OF TRANSCRIPTION 1-ALPHA/BETA (PTHR11801:SF58)
	HUMAN|HGNC=11159|UniProtKB=P62316	Small nuclear ribonucleoprotein Sm D2;SNRPD2;ortholog	SMALL NUCLEAR RIBONUCLEOPROTEIN SM D2 (PTHR12777:SF2)
HUMAN|HGNC=13917|UniProtKB=Q13838		Spliceosome RNA helicase DDX39B;DDX39B;ortholog	SPLICEOSOME RNA HELICASE DDX39B (PTHR24031:SF521)
	HUMAN|HGNC=30646|UniProtKB=Q7KZF4	Staphylococcal nuclease domain-containing protein 1;SND1;ortholog	STAPHYLOCOCCAL NUCLEASE DOMAIN-CONTAINING PROTEIN 1 (PTHR12302:SF10)
HUMAN|HGNC=9701|UniProtKB=Q00577	HUMAN|HGNC=9701|UniProtKB=Q00577	Transcriptional activator protein Pur-alpha;PURA;ortholog	TRANSCRIPTIONAL ACTIVATOR PROTEIN PUR-ALPHA (PTHR12611:SF9)
HUMAN|HGNC=10979|UniProtKB=P53007	HUMAN|HGNC=10979|UniProtKB=P53007	Tricarboxylate transport protein, mitochondrial;SLC25A1;ortholog	TRICARBOXYLATE TRANSPORT PROTEIN, MITOCHONDRIAL (PTHR24089:SF459)
HUMAN|HGNC=854|UniProtKB=P21281	HUMAN|HGNC=854|UniProtKB=P21281	V-type proton ATPase subunit B, brain isoform;ATP6V1B2;ortholog	V-TYPE PROTON ATPASE SUBUNIT B, BRAIN ISOFORM (PTHR43389:SF1)
HUMAN|HGNC=12833|UniProtKB=P13010	HUMAN|HGNC=12833|UniProtKB=P13010	X-ray repair cross-complementing protein 5;XRCC5;ortholog	X-RAY REPAIR CROSS-COMPLEMENTING PROTEIN 5 (PTHR12604:SF7)

oxidoreductase (PC00176)

HUMAN|HGNC=4907|UniProtKB=P31937		3-hydroxyisobutyrate dehydrogenase, mitochondrial;HIBADH;ortholog	3-HYDROXYISOBUTYRATE DEHYDROGENASE, MITOCHONDRIAL (PTHR22981:SF81)
HUMAN|HGNC=412|UniProtKB=P49189		4-trimethylaminobutyraldehyde dehydrogenase;ALDH9A1;ortholog	4-TRIMETHYLAMINOBUTYRALDEHYDE DEHYDROGENASE (PTHR11699:SF247)
	HUMAN|HGNC=21497|UniProtKB=Q9H845	Acyl-CoA dehydrogenase family member 9, mitochondrial;ACAD9;ortholog	ACYL-COA DEHYDROGENASE FAMILY MEMBER 9, MITOCHONDRIAL (PTHR43884:SF7)
HUMAN|HGNC=380|UniProtKB=P14550	HUMAN|HGNC=380|UniProtKB=P14550	Alcohol dehydrogenase [NADP(+)];AKR1A1;ortholog	ALCOHOL DEHYDROGENASE [NADP(+)] (PTHR11732:SF347)
HUMAN|HGNC=404|UniProtKB=P05091	HUMAN|HGNC=404|UniProtKB=P05091	Aldehyde dehydrogenase, mitochondrial;ALDH2;ortholog	ALDEHYDE DEHYDROGENASE, MITOCHONDRIAL (PTHR11699:SF232)
HUMAN|HGNC=6833|UniProtKB=P21397	HUMAN|HGNC=6833|UniProtKB=P21397	Amine oxidase [flavin-containing] A;MAOA;ortholog	AMINE OXIDASE [FLAVIN-CONTAINING] A (PTHR43563:SF4)
HUMAN|HGNC=6834|UniProtKB=P27338	HUMAN|HGNC=6834|UniProtKB=P27338	Amine oxidase [flavin-containing] B;MAOB;ortholog	AMINE OXIDASE [FLAVIN-CONTAINING] B (PTHR43563:SF6)
HUMAN|HGNC=1548|UniProtKB=P16152	HUMAN|HGNC=1548|UniProtKB=P16152	Carbonyl reductase [NADPH] 1;CBR1;ortholog	CARBONYL REDUCTASE [NADPH] 1 (PTHR43490:SF35)

HUMAN|HGNC=1516|UniProtKB=P04040		Catalase;CAT;ortholog	CATALASE (PTHR11465:SF29)
HUMAN|HGNC=13518|UniProtKB=Q9Y696		Chloride intracellular channel protein 4;CLIC4;ortholog	CHLORIDE INTRACELLULAR CHANNEL PROTEIN 4 (PTHR11260:SF443)
HUMAN|HGNC=8011|UniProtKB=P78357	HUMAN|HGNC=8011|UniProtKB=P78357	Contactin-associated protein 1;CNTNAP1;ortholog	CONTACTIN-ASSOCIATED PROTEIN 1 (PTHR10127:SF740)
HUMAN|HGNC=12582|UniProtKB=P14927		Cytochrome b-c1 complex subunit 7;UQCRB;ortholog	CYTOCHROME B-C1 COMPLEX SUBUNIT 7 (PTHR12022:SF1)
HUMAN|HGNC=7421|UniProtKB=P00403	HUMAN|HGNC=7421|UniProtKB=P00403	Cytochrome c oxidase subunit 2;MT-CO2;ortholog	CYTOCHROME C OXIDASE SUBUNIT 2 (PTHR22888:SF14)
	HUMAN|HGNC=2267|UniProtKB=P20674	Cytochrome c oxidase subunit 5A, mitochondrial;COX5A;ortholog	CYTOCHROME C OXIDASE SUBUNIT 5A, MITOCHONDRIAL (PTHR14200:SF14)
HUMAN|HGNC=2269|UniProtKB=P10606	HUMAN|HGNC=2269|UniProtKB=P10606	Cytochrome c oxidase subunit 5B, mitochondrial;COX5B;ortholog	CYTOCHROME C OXIDASE SUBUNIT 5B, MITOCHONDRIAL (PTHR10122:SF4)
HUMAN|HGNC=2280|UniProtKB=P14854	HUMAN|HGNC=2280|UniProtKB=P14854	Cytochrome c oxidase subunit 6B1;COX6B1;ortholog	CYTOCHROME C OXIDASE SUBUNIT 6B1 (PTHR11387:SF26)
	HUMAN|HGNC=7687|UniProtKB=O00483	Cytochrome c oxidase subunit NDUFA4;NDUFA4;ortholog	CYTOCHROME C OXIDASE SUBUNIT NDUFA4 (PTHR14256:SF9)
HUMAN|HGNC=3978|UniProtKB=O75891		Cytosolic 10-formyltetrahydrofolate dehydrogenase;ALDH1L1;ortholog	CYTOSOLIC 10-FORMYLTETRAHYDROFOLATE DEHYDROGENASE (PTHR11699:SF220)
HUMAN|HGNC=8923|UniProtKB=O43175	HUMAN|HGNC=8923|UniProtKB=O43175	D-3-phosphoglycerate dehydrogenase;PHGDH;ortholog	D-3-PHOSPHOGLYCERATE DEHYDROGENASE (PTHR42938:SF9)
	HUMAN|HGNC=1027|UniProtKB=Q02338	D-beta-hydroxybutyrate dehydrogenase, mitochondrial;BDH1;ortholog	D-BETA-HYDROXYBUTYRATE DEHYDROGENASE, MITOCHONDRIAL (PTHR43313:SF21)
HUMAN|HGNC=3149|UniProtKB=Q13011		Delta(3,5)-Delta(2,4)-dienoyl-CoA isomerase, mitochondrial;ECH1;ortholog	DELTA(3,5)-DELTA(2,4)-DIENOYL-COA ISOMERASE, MITOCHONDRIAL (PTHR43149:SF3)
HUMAN|HGNC=9752|UniProtKB=P09417		Dihydropteridine reductase;QDPR;ortholog	DIHYDROPTERIDINE REDUCTASE (PTHR15104:SF1)
HUMAN|HGNC=3482|UniProtKB=P38117		Electron transfer flavoprotein subunit beta;ETFB;ortholog	ELECTRON TRANSFER FLAVOPROTEIN SUBUNIT BETA (PTHR21294:SF14)
HUMAN|HGNC=3213|UniProtKB=P26641	HUMAN|HGNC=3213|UniProtKB=P26641	Elongation factor 1-gamma;EEF1G;ortholog	ELONGATION FACTOR 1-GAMMA (PTHR11260:SF420)
HUMAN|HGNC=3151|UniProtKB=P30084		Enoyl-CoA hydratase, mitochondrial;ECHS1;ortholog	ENOYL-COA HYDRATASE, MITOCHONDRIAL (PTHR11941:SF111)
HUMAN|HGNC=13280|UniProtKB=Q96HE7		ERO1-like protein alpha;ERO1L;ortholog	ERO1-LIKE PROTEIN ALPHA (PTHR12613:SF4)
HUMAN|HGNC=3554|UniProtKB=Q92506		Estradiol 17-beta-dehydrogenase 8;HSD17B8;ortholog	ESTRADIOL 17-BETA-DEHYDROGENASE 8 (PTHR42760:SF32)
HUMAN|HGNC=3594|UniProtKB=P49327	HUMAN|HGNC=3594|UniProtKB=P49327	Fatty acid synthase;FASN;ortholog	FATTY ACID SYNTHASE (PTHR43775:SF2)
	HUMAN|HGNC=1063|UniProtKB=P30043	Flavin reductase (NADPH);BLVRB;ortholog	FLAVIN REDUCTASE (NADPH) (PTHR43355:SF2)
HUMAN|HGNC=964|UniProtKB=O75936		Gamma-butyrobetaine dioxygenase;BBOX1;ortholog	GAMMA-BUTYROBETAINE DIOXYGENASE (PTHR10696:SF41)
HUMAN|HGNC=15968|UniProtKB=Q8TB36		Ganglioside-induced differentiation-associated protein 1;GDAP1;ortholog	GANGLIOSIDE-INDUCED DIFFERENTIATION-ASSOCIATED PROTEIN 1 (PTHR11260:SF391)
HUMAN|HGNC=4335|UniProtKB=P00367		Glutamate dehydrogenase 1, mitochondrial;GLUD1;ortholog	GLUTAMATE DEHYDROGENASE 1, MITOCHONDRIAL (PTHR11606:SF17)
HUMAN|HGNC=4141|UniProtKB=P04406	HUMAN|HGNC=4141|UniProtKB=P04406	Glyceraldehyde-3-phosphate dehydrogenase;GAPDH;ortholog	GLYCERALDEHYDE-3-PHOSPHATE DEHYDROGENASE (PTHR10836:SF70)
HUMAN|HGNC=4570|UniProtKB=Q9UBQ7		Glyoxylate reductase/hydroxypyruvate reductase;GRHPR;ortholog	GLYOXYLATE REDUCTASE/HYDROXYPYRUVATE REDUCTASE (PTHR10996:SF172)
HUMAN|HGNC=4799|UniProtKB=Q16836		Hydroxyacyl-coenzyme A dehydrogenase, mitochondrial;HADH;ortholog	HYDROXYACYL-COENZYME A DEHYDROGENASE, MITOCHONDRIAL (PTHR43561:SF3)

HUMAN|HGNC=5384|UniProtKB=P50213	HUMAN|HGNC=5384|UniProtKB=P50213	Isocitrate dehydrogenase [NAD] subunit alpha, mitochondrial;IDH3A;ortholog	ISOCITRATE DEHYDROGENASE [NAD] SUBUNIT ALPHA, MITOCHONDRIAL (PTHR11835:SF58)
HUMAN|HGNC=5385|UniProtKB=O43837	HUMAN|HGNC=5385|UniProtKB=O43837	Isocitrate dehydrogenase [NAD] subunit beta, mitochondrial;IDH3B;ortholog	ISOCITRATE DEHYDROGENASE [NAD] SUBUNIT BETA, MITOCHONDRIAL (PTHR11835:SF60)
HUMAN|HGNC=6535|UniProtKB=P00338		L-lactate dehydrogenase A chain;LDHA;ortholog	L-LACTATE DEHYDROGENASE A CHAIN (PTHR43128:SF4)
HUMAN|HGNC=6541|UniProtKB=P07195	HUMAN|HGNC=6541|UniProtKB=P07195	L-lactate dehydrogenase B chain;LDHB;ortholog	L-LACTATE DEHYDROGENASE B CHAIN (PTHR43128:SF5)
HUMAN|HGNC=122|UniProtKB=P24666		Low molecular weight phosphotyrosine protein phosphatase;ACP1;ortholog	LOW MOLECULAR WEIGHT PHOSPHOTYROSINE PROTEIN PHOSPHATASE (PTHR11717:SF26)
HUMAN|HGNC=6970|UniProtKB=P40925		Malate dehydrogenase, cytoplasmic;MDH1;ortholog	MALATE DEHYDROGENASE, CYTOPLASMIC (PTHR23382:SF10)
HUMAN|HGNC=6971|UniProtKB=P40926		Malate dehydrogenase, mitochondrial;MDH2;ortholog	MALATE DEHYDROGENASE, MITOCHONDRIAL (PTHR11540:SF35)
HUMAN|HGNC=6984|UniProtKB=P23368		NAD-dependent malic enzyme, mitochondrial;ME2;ortholog	NAD-DEPENDENT MALIC ENZYME, MITOCHONDRIAL (PTHR23406:SF50)
HUMAN|HGNC=17194|UniProtKB=Q9P0J0		NADH dehydrogenase [ubiquinone] 1 alpha subcomplex subunit 13;NDUFA13;ortholog	NADH DEHYDROGENASE [UBIQUINONE] 1 ALPHA SUBCOMPLEX SUBUNIT 13 (PTHR12966:SF1)
HUMAN|HGNC=7688|UniProtKB=Q16718	HUMAN|HGNC=7688|UniProtKB=Q16718	NADH dehydrogenase [ubiquinone] 1 alpha subcomplex subunit 5;NDUFA5;ortholog	NADH DEHYDROGENASE [UBIQUINONE] 1 ALPHA SUBCOMPLEX SUBUNIT 5 (PTHR12653:SF1)
HUMAN|HGNC=7692|UniProtKB=P51970	HUMAN|HGNC=7692|UniProtKB=P51970	NADH dehydrogenase [ubiquinone] 1 alpha subcomplex subunit 8;NDUFA8;ortholog	NADH DEHYDROGENASE [UBIQUINONE] 1 ALPHA SUBCOMPLEX SUBUNIT 8 (PTHR13344:SF1)
HUMAN|HGNC=7693|UniProtKB=Q16795	HUMAN|HGNC=7693|UniProtKB=Q16795	NADH dehydrogenase [ubiquinone] 1 alpha subcomplex subunit 9, mitochondrial;NDUFA9;ortholog	NADH DEHYDROGENASE [UBIQUINONE] 1 ALPHA SUBCOMPLEX SUBUNIT 9, MITOCHONDRIAL (PTHR12126:SF9)
HUMAN|HGNC=7698|UniProtKB=O43676		NADH dehydrogenase [ubiquinone] 1 beta subcomplex subunit 3;NDUFB3;ortholog	NADH DEHYDROGENASE [UBIQUINONE] 1 BETA SUBCOMPLEX SUBUNIT 3 (PTHR15082:SF4)
HUMAN|HGNC=7717|UniProtKB=P19404	HUMAN|HGNC=7717|UniProtKB=P19404	NADH dehydrogenase [ubiquinone] flavoprotein 2, mitochondrial;NDUFV2;ortholog	NADH DEHYDROGENASE [UBIQUINONE] FLAVOPROTEIN 2, MITOCHONDRIAL (PTHR10371:SF8)
HUMAN|HGNC=7713|UniProtKB=O75380		NADH dehydrogenase [ubiquinone] iron-sulfur protein 6, mitochondrial;NDUFS6;ortholog	NADH DEHYDROGENASE [UBIQUINONE] IRON-SULFUR PROTEIN 6, MITOCHONDRIAL (PTHR13156:SF1)
HUMAN|HGNC=7714|UniProtKB=O75251		NADH dehydrogenase [ubiquinone] iron-sulfur protein 7, mitochondrial;NDUFS7;ortholog	NADH DEHYDROGENASE [UBIQUINONE] IRON-SULFUR PROTEIN 7, MITOCHONDRIAL (PTHR11995:SF24)
HUMAN|HGNC=13397|UniProtKB=Q9UHQ9	HUMAN|HGNC=13397|UniProtKB=Q9UHQ9	NADH-cytochrome b5 reductase 1;CYB5R1;ortholog	NADH-CYTOCHROME B5 REDUCTASE 1 (PTHR19370:SF138)
HUMAN|HGNC=7707|UniProtKB=P28331	HUMAN|HGNC=7707|UniProtKB=P28331	NADH-ubiquinone oxidoreductase 75 kDa subunit, mitochondrial;NDUFS1;ortholog	NADH-UBIQUINONE OXIDOREDUCTASE 75 KDA SUBUNIT, MITOCHONDRIAL (PTHR11615:SF194)
HUMAN|HGNC=9353|UniProtKB=P32119	HUMAN|HGNC=9353|UniProtKB=P32119	Peroxiredoxin-2;PRDX2;ortholog	PEROXIREDOXIN-2 (PTHR10681:SF136)
HUMAN|HGNC=16753|UniProtKB=P30041	HUMAN|HGNC=16753|UniProtKB=P30041	Peroxiredoxin-6;PRDX6;ortholog	PEROXIREDOXIN-6 (PTHR43503:SF1)
HUMAN|HGNC=17822|UniProtKB=Q9H7Z7		Prostaglandin E synthase 2;PTGES2;ortholog	PROSTAGLANDIN E SYNTHASE 2 (PTHR12782:SF9)
	HUMAN|HGNC=33782|UniProtKB=Q6GMV3	Putative peptidyl-tRNA hydrolase PTRHD1;PTRHD1;ortholog	PEPTIDYL-TRNA HYDROLASE PTRHD1-RELATED (PTHR11717:SF25)
HUMAN|HGNC=402|UniProtKB=P00352		Retinal dehydrogenase 1;ALDH1A1;ortholog	RETINAL DEHYDROGENASE 1 (PTHR11699:SF255)
HUMAN|HGNC=17964|UniProtKB=Q8TC12		Retinol dehydrogenase 11;RDH11;ortholog	RETINOL DEHYDROGENASE 11 (PTHR24320:SF131)
HUMAN|HGNC=10681|UniProtKB=P21912		Succinate dehydrogenase [ubiquinone] iron-sulfur subunit, mitochondrial;SDHB;ortholog	SUCCINATE DEHYDROGENASE [UBIQUINONE] IRON-SULFUR SUBUNIT, MITOCHONDRIAL (PTHR11921:SF31)

HUMAN|HGNC=408|UniProtKB=P51649		Succinate-semialdehyde dehydrogenase, mitochondrial;ALDH5A1;ortholog	SUCCINATE-SEMIALDEHYDE DEHYDROGENASE, MITOCHONDRIAL (PTHR43353:SF7)
HUMAN|HGNC=11179|UniProtKB=P00441		Superoxide dismutase [Cu-Zn];SOD1;ortholog	SUPEROXIDE DISMUTASE [CU-ZN] (PTHR10003:SF50)
HUMAN|HGNC=16919|UniProtKB=Q99536	HUMAN|HGNC=16919|UniProtKB=Q99536	Synaptic vesicle membrane protein VAT-1 homolog;VAT1;ortholog	SYNAPTIC VESICLE MEMBRANE PROTEIN VAT-1 HOMOLOG (PTHR11695:SF616)
HUMAN|HGNC=19691|UniProtKB=Q9BV79		Trans-2-enoyl-CoA reductase, mitochondrial;MECR;ortholog	TRANS-2-ENOYL-COA REDUCTASE, MITOCHONDRIAL (PTHR11695:SF624)
HUMAN|HGNC=11834|UniProtKB=P29401		Transketolase;TKT;ortholog	TRANSKETOLASE (PTHR43195:SF3)
HUMAN|HGNC=4801|UniProtKB=P40939	HUMAN|HGNC=4801|UniProtKB=P40939	Trifunctional enzyme subunit alpha, mitochondrial;HADHA;ortholog	TRIFUNCTIONAL ENZYME SUBUNIT ALPHA, MITOCHONDRIAL (PTHR43612:SF3)
HUMAN|HGNC=18646|UniProtKB=Q53GQ0	HUMAN|HGNC=18646|UniProtKB=Q53GQ0	Very-long-chain 3-oxoacyl-CoA reductase;HSD17B12;ortholog	VERY-LONG-CHAIN 3-OXOACYL-COA REDUCTASE (PTHR24322:SF672)

receptor (PC00197)

	HUMAN|HGNC=5273|UniProtKB=P98160	Basement membrane-specific heparan sulfate proteoglycan core protein;HSPG2;ortholog	BASEMENT MEMBRANE-SPECIFIC HEPARAN SULFATE PROTEOGLYCAN CORE PROTEIN (PTHR10574:SF343)
HUMAN|HGNC=1044|UniProtKB=P21810	HUMAN|HGNC=1044|UniProtKB=P21810	Biglycan;BGN;ortholog	BIGLYCAN (PTHR24369:SF99)
HUMAN|HGNC=6934|UniProtKB=P43121		Cell surface glycoprotein MUC18;MCAM;ortholog	CELL SURFACE GLYCOPROTEIN MUC18 (PTHR11973:SF21)
HUMAN|HGNC=2172|UniProtKB=Q02246		Contactin-2;CNTN2;ortholog	CONTACTIN-2 (PTHR10489:SF882)
HUMAN|HGNC=8011|UniProtKB=P78357	HUMAN|HGNC=8011|UniProtKB=P78357	Contactin-associated protein 1;CNTNAP1;ortholog	CONTACTIN-ASSOCIATED PROTEIN 1 (PTHR10127:SF740)
HUMAN|HGNC=12825|UniProtKB=O14980	HUMAN|HGNC=12825|UniProtKB=O14980	Exportin-1;XPO1;ortholog	EXPORTIN-1 (PTHR11223:SF9)
	HUMAN|HGNC=6485|UniProtKB=O15230	Laminin subunit alpha-5;LAMA5;ortholog	LAMININ SUBUNIT ALPHA-5 (PTHR10574:SF355)
HUMAN|HGNC=6487|UniProtKB=P55268	HUMAN|HGNC=6487|UniProtKB=P55268	Laminin subunit beta-2;LAMB2;ortholog	LAMININ SUBUNIT BETA-2 (PTHR10574:SF346)
	HUMAN|HGNC=6492|UniProtKB=P11047	Laminin subunit gamma-1;LAMC1;ortholog	LAMININ SUBUNIT GAMMA-1 (PTHR10574:SF309)
HUMAN|HGNC=15714|UniProtKB=P42704	HUMAN|HGNC=15714|UniProtKB=P42704	Leucine-rich PPR motif-containing protein, mitochondrial;LRPPRC;ortholog	LEUCINE-RICH PPR MOTIF-CONTAINING PROTEIN, MITOCHONDRIAL (PTHR24015:SF1304)
HUMAN|HGNC=6705|UniProtKB=Q13449		Limbic system-associated membrane protein;LSAMP;ortholog	LIMBIC SYSTEM-ASSOCIATED MEMBRANE PROTEIN (PTHR42757:SF13)
HUMAN|HGNC=16090|UniProtKB=O00264		Membrane-associated progesterone receptor component 1;PGRMC1;ortholog	MEMBRANE-ASSOCIATED PROGESTERONE RECEPTOR COMPONENT 1 (PTHR10281:SF46)
HUMAN|HGNC=16089|UniProtKB=O15173		Membrane-associated progesterone receptor component 2;PGRMC2;ortholog	MEMBRANE-ASSOCIATED PROGESTERONE RECEPTOR COMPONENT 2 (PTHR10281:SF59)
HUMAN|HGNC=8135|UniProtKB=P23515		Oligodendrocyte-myelin glycoprotein;OMG;ortholog	OLIGODENDROCYTE-MYELIN GLYCOPROTEIN (PTHR24373:SF208)
	HUMAN|HGNC=9099|UniProtKB=Q9UIW2	Plexin-A1;PLXNA1;ortholog	PLEXIN-A1 (PTHR22625:SF47)
HUMAN|HGNC=9357|UniProtKB=P51888	HUMAN|HGNC=9357|UniProtKB=P51888	Prolargin;PRELP;ortholog	PROLARGIN (PTHR24373:SF224)
HUMAN|HGNC=30077|UniProtKB=Q00765		Receptor expression-enhancing protein 5;REEP5;ortholog	RECEPTOR EXPRESSION-ENHANCING PROTEIN 5 (PTHR12300:SF91)
	HUMAN|HGNC=25655|UniProtKB=Q9NUJ3	T-complex protein 11-like protein 1;TCP11L1;ortholog	T-COMPLEX PROTEIN 11-LIKE PROTEIN 1 (PTHR12832:SF22)
	HUMAN|HGNC=14406|UniProtKB=Q9H3S7	Tyrosine-protein phosphatase non-receptor type 23;PTPN23;ortholog	TYROSINE-PROTEIN PHOSPHATASE NON-RECEPTOR TYPE 23 (PTHR19134:SF349)

signaling molecule (PC00207)

HUMAN|HGNC=9060|UniProtKB=P51178		1-phosphatidylinositol 4,5-bisphosphate phosphodiesterase delta-1;PLCD1;ortholog	1-PHOSPHATIDYLINOSITOL 4,5-BISPHOSPHATE PHOSPHODIESTERASE DELTA-1 (PTHR10336:SF126)
HUMAN|HGNC=9061|UniProtKB=Q8N3E9		1-phosphatidylinositol 4,5-bisphosphate phosphodiesterase delta-3;PLCD3;ortholog	1-PHOSPHATIDYLINOSITOL 4,5-BISPHOSPHATE PHOSPHODIESTERASE DELTA-3 (PTHR10336:SF141)
HUMAN|HGNC=1044|UniProtKB=P21810	HUMAN|HGNC=1044|UniProtKB=P21810	Biglycan;BGN;ortholog	BIGLYCAN (PTHR24369:SF99)
HUMAN|HGNC=13518|UniProtKB=Q9Y696		Chloride intracellular channel protein 4;CLIC4;ortholog	CHLORIDE INTRACELLULAR CHANNEL PROTEIN 4 (PTHR11260:SF443)
HUMAN|HGNC=8011|UniProtKB=P78357	HUMAN|HGNC=8011|UniProtKB=P78357	Contactin-associated protein 1;CNTNAP1;ortholog	CONTACTIN-ASSOCIATED PROTEIN 1 (PTHR10127:SF740)
HUMAN|HGNC=2732|UniProtKB=P30046		D-dopachrome decarboxylase;DDT;ortholog	D-DOPACHROME DECARBOXYLASE (PTHR11954:SF30)
HUMAN|HGNC=3213|UniProtKB=P26641	HUMAN|HGNC=3213|UniProtKB=P26641	Elongation factor 1-gamma;EEF1G;ortholog	ELONGATION FACTOR 1-GAMMA (PTHR11260:SF420)
HUMAN|HGNC=3662|UniProtKB=P02675	HUMAN|HGNC=3662|UniProtKB=P02675	Fibrinogen beta chain;FGB;ortholog	FIBRINOGEN BETA CHAIN (PTHR19143:SF313)
HUMAN|HGNC=6561|UniProtKB=P09382		Galectin-1;LGALS1;ortholog	GALECTIN-1 (PTHR11346:SF131)
HUMAN|HGNC=15968|UniProtKB=Q8TB36		Ganglioside-induced differentiation-associated protein 1;GDAP1;ortholog	GANGLIOSIDE-INDUCED DIFFERENTIATION-ASSOCIATED PROTEIN 1 (PTHR11260:SF391)
HUMAN|HGNC=4373|UniProtKB=P60983		Glia maturation factor beta;GMFB;ortholog	GLIA MATURATION FACTOR BETA (PTHR11249:SF5)
HUMAN|HGNC=4413|UniProtKB=P36915		Guanine nucleotide-binding protein-like 1;GNL1;ortholog	GUANINE NUCLEOTIDE-BINDING PROTEIN-LIKE 1 (PTHR11089:SF55)
HUMAN|HGNC=26361|UniProtKB=Q14CZ8		Hepatocyte cell adhesion molecule;HEPACAM;ortholog	HEPATOCYTE CELL ADHESION MOLECULE (PTHR12080:SF66)
HUMAN|Gene=CGI-142|UniProtKB=Q9Y3E1		Hepatoma-derived growth factor-related protein 3;HDGFRP3;ortholog	HEPATOMA-DERIVED GROWTH FACTOR-RELATED PROTEIN 3 (PTHR12550:SF55)
	HUMAN|HGNC=17512|UniProtKB=Q86YM7	Homer protein homolog 1;HOMER1;ortholog	HOMER PROTEIN HOMOLOG 1 (PTHR10918:SF5)
HUMAN|HGNC=5348|UniProtKB=Q9UMF0	HUMAN|HGNC=5348|UniProtKB=Q9UMF0	Intercellular adhesion molecule 5;ICAM5;ortholog	INTERCELLULAR ADHESION MOLECULE 5 (PTHR13771:SF15)
HUMAN|HGNC=16090|UniProtKB=O00264		Membrane-associated progesterone receptor component 1;PGRMC1;ortholog	MEMBRANE-ASSOCIATED PROGESTERONE RECEPTOR COMPONENT 1 (PTHR10281:SF46)
HUMAN|HGNC=16089|UniProtKB=O15173		Membrane-associated progesterone receptor component 2;PGRMC2;ortholog	MEMBRANE-ASSOCIATED PROGESTERONE RECEPTOR COMPONENT 2 (PTHR10281:SF59)
	HUMAN|HGNC=7035|UniProtKB=P55083	Microfibril-associated glycoprotein 4;MFAP4;ortholog	MICROFIBRIL-ASSOCIATED GLYCOPROTEIN 4 (PTHR19143:SF298)
	HUMAN|HGNC=7126|UniProtKB=Q15773	Myeloid leukemia factor 2;MLF2;ortholog	MYELOID LEUKEMIA FACTOR 2 (PTHR13105:SF11)
	HUMAN|HGNC=30092|UniProtKB=P43490	Nicotinamide phosphoribosyltransferase;NAMPT;ortholog	NICOTINAMIDE PHOSPHORIBOSYLTRANSFERASE (PTHR43816:SF1)
	HUMAN|HGNC=9099|UniProtKB=Q9UIW2	Plexin-A1;PLXNA1;ortholog	PLEXIN-A1 (PTHR22625:SF47)
HUMAN|HGNC=29180|UniProtKB=Q9UPV7	HUMAN|HGNC=29180|UniProtKB=Q9UPV7	Protein KIAA1045;KIAA1045;ortholog	PROTEIN KIAA1045 (PTHR10891:SF706)
	HUMAN|HGNC=10490|UniProtKB=Q99584	Protein S100-A13;S100A13;ortholog	PROTEIN S100-A13 (PTHR11639:SF98)
	HUMAN|HGNC=10498|UniProtKB=P05109	Protein S100-A8;S100A8;ortholog	PROTEIN S100-A8 (PTHR11639:SF111)
	HUMAN|HGNC=10499|UniProtKB=P06702	Protein S100-A9;S100A9;ortholog	PROTEIN S100-A9 (PTHR11639:SF110)
HUMAN|HGNC=11953|UniProtKB=Q92752	HUMAN|HGNC=11953|UniProtKB=Q92752	Tenascin-R;TNR;ortholog	TENASCIN-R (PTHR19143:SF307)
HUMAN|HGNC=9662|UniProtKB=P78324	HUMAN|HGNC=9662|UniProtKB=P78324	Tyrosine-protein phosphatase non-receptor type substrate 1;SIRPA;ortholog	TYROSINE-PROTEIN PHOSPHATASE NON-RECEPTOR TYPE SUBSTRATE 1 (PTHR19971:SF21)

storage protein (PC00210)

HUMAN|HGNC=3976|UniProtKB=P02794	HUMAN|HGNC=3976|UniProtKB=P02794	Ferritin heavy chain;FTH1;ortholog	FERRITIN HEAVY CHAIN (PTHR11431:SF53)
HUMAN|HGNC=3999|UniProtKB=P02792	HUMAN|HGNC=3999|UniProtKB=P02792	Ferritin light chain;FTL;ortholog	FERRITIN LIGHT CHAIN (PTHR11431:SF66)
**structural protein (PC00211)**			
HUMAN|HGNC=6057|UniProtKB=Q16352	HUMAN|HGNC=6057|UniProtKB=Q16352	Alpha-internexin;INA;ortholog	ALPHA-INTERNEXIN (PTHR23239:SF273)
HUMAN|HGNC=2770|UniProtKB=P17661	HUMAN|HGNC=2770|UniProtKB=P17661	Desmin;DES;ortholog	DESMIN (PTHR23239:SF307)
HUMAN|HGNC=4235|UniProtKB=P14136	HUMAN|HGNC=4235|UniProtKB=P14136	Glial fibrillary acidic protein;GFAP;ortholog	GLIAL FIBRILLARY ACIDIC PROTEIN (PTHR23239:SF249)
HUMAN|HGNC=6413|UniProtKB=P13645	HUMAN|HGNC=6413|UniProtKB=P13645	Keratin, type I cytoskeletal 10;KRT10;ortholog	KERATIN, TYPE I CYTOSKELETAL 10 (PTHR23239:SF308)
HUMAN|HGNC=6416|UniProtKB=P02533	HUMAN|HGNC=6416|UniProtKB=P02533	Keratin, type I cytoskeletal 14;KRT14;ortholog	KERATIN, TYPE I CYTOSKELETAL 14 (PTHR23239:SF272)
	HUMAN|HGNC=6423|UniProtKB=P08779	Keratin, type I cytoskeletal 16;KRT16;ortholog	KERATIN, TYPE I CYTOSKELETAL 16 (PTHR23239:SF263)
	HUMAN|HGNC=6427|UniProtKB=Q04695	Keratin, type I cytoskeletal 17;KRT17;ortholog	KERATIN, TYPE I CYTOSKELETAL 17 (PTHR23239:SF295)
HUMAN|HGNC=6447|UniProtKB=P35527	HUMAN|HGNC=6447|UniProtKB=P35527	Keratin, type I cytoskeletal 9;KRT9;ortholog	KERATIN, TYPE I CYTOSKELETAL 9 (PTHR23239:SF281)
HUMAN|HGNC=6412|UniProtKB=P04264	HUMAN|HGNC=6412|UniProtKB=P04264	Keratin, type II cytoskeletal 1;KRT1;ortholog	KERATIN, TYPE II CYTOSKELETAL 1 (PTHR23239:SF236)
HUMAN|HGNC=6439|UniProtKB=P35908	HUMAN|HGNC=6439|UniProtKB=P35908	Keratin, type II cytoskeletal 2 epidermal;KRT2;ortholog	KERATIN, TYPE II CYTOSKELETAL 2 EPIDERMAL (PTHR23239:SF228)
HUMAN|HGNC=6442|UniProtKB=P13647	HUMAN|HGNC=6442|UniProtKB=P13647	Keratin, type II cytoskeletal 5;KRT5;ortholog	KERATIN, TYPE II CYTOSKELETAL 5 (PTHR23239:SF279)
	HUMAN|HGNC=6443|UniProtKB=P02538	Keratin, type II cytoskeletal 6A;KRT6A;ortholog	KERATIN, TYPE II CYTOSKELETAL 6A (PTHR23239:SF232)
	HUMAN|HGNC=6444|UniProtKB=P04259	Keratin, type II cytoskeletal 6B;KRT6B;ortholog	KERATIN, TYPE II CYTOSKELETAL 6B-RELATED (PTHR23239:SF293)
	HUMAN|HGNC=28929|UniProtKB=Q7RTS7	Keratin, type II cytoskeletal 74;KRT74;ortholog	KERATIN, TYPE II CYTOSKELETAL 74 (PTHR23239:SF328)
	HUMAN|HGNC=28930|UniProtKB=Q5XKE5	Keratin, type II cytoskeletal 79;KRT79;ortholog	KERATIN, TYPE II CYTOSKELETAL 79 (PTHR23239:SF304)
HUMAN|HGNC=6638|UniProtKB=Q03252	HUMAN|HGNC=6638|UniProtKB=Q03252	Lamin-B2;LMNB2;ortholog	LAMIN-B2 (PTHR23239:SF280)
HUMAN|HGNC=9086|UniProtKB=P60201	HUMAN|HGNC=9086|UniProtKB=P60201	Myelin proteolipid protein;PLP1;ortholog	MYELIN PROTEOLIPID PROTEIN (PTHR11683:SF18)
HUMAN|HGNC=7739|UniProtKB=P07196	HUMAN|HGNC=7739|UniProtKB=P07196	Neurofilament light polypeptide;NEFL;ortholog	NEUROFILAMENT LIGHT POLYPEPTIDE (PTHR23239:SF242)
	HUMAN|HGNC=4460|UniProtKB=P51674	Neuronal membrane glycoprotein M6-a;GPM6A;ortholog	NEURONAL MEMBRANE GLYCOPROTEIN M6-A (PTHR11683:SF19)
	HUMAN|HGNC=4461|UniProtKB=Q13491	Neuronal membrane glycoprotein M6-b;GPM6B;ortholog	NEURONAL MEMBRANE GLYCOPROTEIN M6-B (PTHR11683:SF17)
HUMAN|HGNC=9461|UniProtKB=P41219		Peripherin;PRPH;ortholog	PERIPHERIN (PTHR23239:SF288)
HUMAN|HGNC=12692|UniProtKB=P08670	HUMAN|HGNC=12692|UniProtKB=P08670	Vimentin;VIM;ortholog	VIMENTIN (PTHR23239:SF258)
**transcription factor (PC00218)**			
HUMAN|HGNC=30688|UniProtKB=Q86VP6		Cullin-associated NEDD8-dissociated protein 1;CAND1;ortholog	CULLIN-ASSOCIATED NEDD8-DISSOCIATED PROTEIN 1 (PTHR12696:SF3)
HUMAN|Gene=CGI-142|UniProtKB=Q9Y3E1		Hepatoma-derived growth factor-related protein 3;HDGFRP3;ortholog	HEPATOMA-DERIVED GROWTH FACTOR-RELATED PROTEIN 3 (PTHR12550:SF55)
HUMAN|HGNC=8550|UniProtKB=Q9UQ80		Proliferation-associated protein 2G4;PA2G4;ortholog	PROLIFERATION-ASSOCIATED PROTEIN 2G4 (PTHR10804:SF123)

HUMAN|HGNC=10074|UniProtKB=P13489	HUMAN|HGNC=10074|UniProtKB=P13489	Ribonuclease inhibitor;RNH1;ortholog	RIBONUCLEASE INHIBITOR (PTHR24112:SF52)
	HUMAN|HGNC=11362|UniProtKB=P42224	Signal transducer and activator of transcription 1-alpha/beta;STAT1;ortholog	SIGNAL TRANSDUCER AND ACTIVATOR OF TRANSCRIPTION 1-ALPHA/BETA (PTHR11801:SF58)
	HUMAN|HGNC=30646|UniProtKB=Q7KZF4	Staphylococcal nuclease domain-containing protein 1;SND1;ortholog	STAPHYLOCOCCAL NUCLEASE DOMAIN-CONTAINING PROTEIN 1 (PTHR12302:SF10)
HUMAN|HGNC=9701|UniProtKB=Q00577	HUMAN|HGNC=9701|UniProtKB=Q00577	Transcriptional activator protein Pur-alpha;PURA;ortholog	TRANSCRIPTIONAL ACTIVATOR PROTEIN PUR-ALPHA (PTHR12611:SF9)
**transfer/carrier protein (PC00219)**			
HUMAN|HGNC=10990|UniProtKB=P12235	HUMAN|HGNC=10990|UniProtKB=P12235	ADP/ATP translocase 1;SLC25A4;ortholog	ADP/ATP TRANSLOCASE 1 (PTHR24089:SF506)
HUMAN|HGNC=10991|UniProtKB=P05141	HUMAN|HGNC=10991|UniProtKB=P05141	ADP/ATP translocase 2;SLC25A5;ortholog	ADP/ATP TRANSLOCASE 2 (PTHR24089:SF446)
HUMAN|HGNC=10992|UniProtKB=P12236	HUMAN|HGNC=10992|UniProtKB=P12236	ADP/ATP translocase 3;SLC25A6;ortholog	ADP/ATP TRANSLOCASE 3 (PTHR24089:SF411)
	HUMAN|HGNC=619|UniProtKB=Q9BQE5	Apolipoprotein L2;APOL2;ortholog	APOLIPOPROTEIN L1-RELATED (PTHR14096:SF49)
HUMAN|HGNC=10982|UniProtKB=O75746	HUMAN|HGNC=10982|UniProtKB=O75746	Calcium-binding mitochondrial carrier protein Aralar1;SLC25A12;ortholog	CALCIUM-BINDING MITOCHONDRIAL CARRIER PROTEIN ARALAR1 (PTHR24089:SF503)
HUMAN|HGNC=16171|UniProtKB=Q9H444		Charged multivesicular body protein 4b;CHMP4B;ortholog	CHARGED MULTIVESICULAR BODY PROTEIN 4B-RELATED (PTHR22761:SF29)
HUMAN|HGNC=8011|UniProtKB=P78357	HUMAN|HGNC=8011|UniProtKB=P78357	Contactin-associated protein 1;CNTNAP1;ortholog	CONTACTIN-ASSOCIATED PROTEIN 1 (PTHR10127:SF740)
HUMAN|HGNC=4823|UniProtKB=P69905	HUMAN|HGNC=4823|UniProtKB=P69905	Hemoglobin subunit alpha;HBA1;ortholog	HEMOGLOBIN SUBUNIT ALPHA (PTHR11442:SF64)
HUMAN|HGNC=4824|UniProtKB=P69905	HUMAN|HGNC=4824|UniProtKB=P69905	Hemoglobin subunit alpha;HBA1;ortholog	HEMOGLOBIN SUBUNIT ALPHA (PTHR11442:SF61)
HUMAN|HGNC=4827|UniProtKB=P68871	HUMAN|HGNC=4827|UniProtKB=P68871	Hemoglobin subunit beta;HBB;ortholog	HEMOGLOBIN SUBUNIT BETA (PTHR11442:SF72)
HUMAN|HGNC=4829|UniProtKB=P02042		Hemoglobin subunit delta;HBD;ortholog	HEMOGLOBIN SUBUNIT DELTA (PTHR11442:SF65)
	HUMAN|HGNC=6396|UniProtKB=O00505	Importin subunit alpha-4;KPNA3;ortholog	IMPORTIN SUBUNIT ALPHA-4 (PTHR23316:SF41)
	HUMAN|HGNC=6394|UniProtKB=P52294	Importin subunit alpha-5;KPNA1;ortholog	IMPORTIN SUBUNIT ALPHA-5 (PTHR23316:SF40)
HUMAN|HGNC=6400|UniProtKB=Q14974	HUMAN|HGNC=6400|UniProtKB=Q14974	Importin subunit beta-1;KPNB1;ortholog	IMPORTIN SUBUNIT BETA-1 (PTHR10527:SF32)
HUMAN|HGNC=19954|UniProtKB=Q9H936	HUMAN|HGNC=19954|UniProtKB=Q9H936	Mitochondrial glutamate carrier 1;SLC25A22;ortholog	MITOCHONDRIAL GLUTAMATE CARRIER 1 (PTHR24089:SF598)
	HUMAN|HGNC=10988|UniProtKB=Q9H1K4	Mitochondrial glutamate carrier 2;SLC25A18;ortholog	MITOCHONDRIAL GLUTAMATE CARRIER 2 (PTHR24089:SF482)
HUMAN|HGNC=9592|UniProtKB=P41222	HUMAN|HGNC=9592|UniProtKB=P41222	Prostaglandin-H2 D-isomerase;PTGDS;ortholog	PROSTAGLANDIN-H2 D-ISOMERASE (PTHR11430:SF114)
HUMAN|HGNC=9393|UniProtKB=P17252	HUMAN|HGNC=9393|UniProtKB=P17252	Protein kinase C alpha type;PRKCA;ortholog	PROTEIN KINASE C ALPHA TYPE (PTHR24356:SF270)
	HUMAN|HGNC=9395|UniProtKB=P05771	Protein kinase C beta type;PRKCB;ortholog	PROTEIN KINASE C BETA TYPE (PTHR24356:SF244)
	HUMAN|HGNC=9401|UniProtKB=Q02156	Protein kinase C epsilon type;PRKCE;ortholog	PROTEIN KINASE C EPSILON TYPE (PTHR24356:SF275)
	HUMAN|HGNC=9402|UniProtKB=P05129	Protein kinase C gamma type;PRKCG;ortholog	PROTEIN KINASE C GAMMA TYPE (PTHR24356:SF293)
	HUMAN|HGNC=49897|UniProtKB=Q6A1A2	Putative 3-phosphoinositide-dependent protein kinase 2;PDPK2P;ortholog	3-PHOSPHOINOSITIDE-DEPENDENT PROTEIN KINASE 1-RELATED (PTHR24356:SF290)
	HUMAN|HGNC=10432|UniProtKB=P51812	Ribosomal protein S6 kinase alpha-3;RPS6KA3;ortholog	RIBOSOMAL PROTEIN S6 KINASE ALPHA-3 (PTHR24351:SF133)
	HUMAN|HGNC=21332|UniProtKB=Q86UX6	Serine/threonine-protein kinase 32C;STK32C;ortholog	SERINE/THREONINE-PROTEIN KINASE 32C (PTHR24356:SF232)

	HUMAN|HGNC=17848|UniProtKB=Q9Y2H1	Serine/threonine-protein kinase 38-like;STK38L;ortholog	SERINE/THREONINE-PROTEIN KINASE 38-LIKE (PTHR24356:SF259)
HUMAN|HGNC=11740|UniProtKB=P02787		Serotransferrin;TF;ortholog	SEROTRANSFERRIN (PTHR11485:SF35)
HUMAN|HGNC=399|UniProtKB=P02768		Serum albumin;ALB;ortholog	SERUM ALBUMIN (PTHR11385:SF18)
HUMAN|HGNC=16085|UniProtKB=Q9H9B4	HUMAN|HGNC=16085|UniProtKB=Q9H9B4	Sideroflexin-1;SFXN1;ortholog	SIDEROFLEXIN-1 (PTHR11153:SF32)
	HUMAN|HGNC=19666|UniProtKB=Q8NFX7	Syntaxin-binding protein 6;STXBP6;ortholog	SYNTAXIN-BINDING PROTEIN 6 (PTHR16092:SF24)
HUMAN|HGNC=10979|UniProtKB=P53007	HUMAN|HGNC=10979|UniProtKB=P53007	Tricarboxylate transport protein, mitochondrial;SLC25A1;ortholog	TRICARBOXYLATE TRANSPORT PROTEIN, MITOCHONDRIAL (PTHR24089:SF459)
	HUMAN|HGNC=25581|UniProtKB=A0AVT1	Ubiquitin-like modifier-activating enzyme 6;UBA6;ortholog	UBIQUITIN-LIKE MODIFIER-ACTIVATING ENZYME 6 (PTHR10953:SF180)

transferase (PC00220)

	HUMAN|HGNC=8809|UniProtKB=Q15118	[Pyruvate dehydrogenase (acetyl-transferring)] kinase isozyme 1, mitochondrial;PDK1;ortholog	[PYRUVATE DEHYDROGENASE (ACETYL-TRANSFERRING)] KINASE ISOZYME 1, MITOCHONDRIAL (PTHR11947:SF25)
HUMAN|HGNC=8811|UniProtKB=Q15120	HUMAN|HGNC=8811|UniProtKB=Q15120	[Pyruvate dehydrogenase (acetyl-transferring)] kinase isozyme 3, mitochondrial;PDK3;ortholog	[PYRUVATE DEHYDROGENASE (ACETYL-TRANSFERRING)] KINASE ISOZYME 3, MITOCHONDRIAL (PTHR11947:SF31)
HUMAN|HGNC=23|UniProtKB=P80404		4-aminobutyrate aminotransferase, mitochondrial;ABAT;ortholog	4-AMINOBUTYRATE AMINOTRANSFERASE, MITOCHONDRIAL (PTHR43206:SF1)
	HUMAN|HGNC=9376|UniProtKB=Q13131	5'-AMP-activated protein kinase catalytic subunit alpha-1;PRKAA1;ortholog	5'-AMP-ACTIVATED PROTEIN KINASE CATALYTIC SUBUNIT ALPHA-1 (PTHR24343:SF209)
HUMAN|HGNC=93|UniProtKB=P24752		Acetyl-CoA acetyltransferase, mitochondrial;ACAT1;ortholog	ACETYL-COA ACETYLTRANSFERASE, MITOCHONDRIAL (PTHR18919:SF114)
	HUMAN|HGNC=21497|UniProtKB=Q9H845	Acyl-CoA dehydrogenase family member 9, mitochondrial;ACAD9;ortholog	ACYL-COA DEHYDROGENASE FAMILY MEMBER 9, MITOCHONDRIAL (PTHR43884:SF7)
HUMAN|HGNC=363|UniProtKB=P27144	HUMAN|HGNC=363|UniProtKB=P27144	Adenylate kinase 4, mitochondrial;AK4;ortholog	ADENYLATE KINASE 4, MITOCHONDRIAL (PTHR23359:SF119)
HUMAN|HGNC=361|UniProtKB=P00568	HUMAN|HGNC=361|UniProtKB=P00568	Adenylate kinase isoenzyme 1;AK1;ortholog	ADENYLATE KINASE ISOENZYME 1 (PTHR23359:SF135)
HUMAN|HGNC=8876|UniProtKB=P17858	HUMAN|HGNC=8876|UniProtKB=P17858	ATP-dependent 6-phosphofructokinase, liver type;PFKL;ortholog	ATP-DEPENDENT 6-PHOSPHOFRUCTOKINASE, LIVER TYPE (PTHR13697:SF35)
HUMAN|HGNC=8877|UniProtKB=P08237	HUMAN|HGNC=8877|UniProtKB=P08237	ATP-dependent 6-phosphofructokinase, muscle type;PFKM;ortholog	ATP-DEPENDENT 6-PHOSPHOFRUCTOKINASE, MUSCLE TYPE (PTHR13697:SF38)
HUMAN|HGNC=8878|UniProtKB=Q01813	HUMAN|HGNC=8878|UniProtKB=Q01813	ATP-dependent 6-phosphofructokinase, platelet type;PFKP;ortholog	ATP-DEPENDENT 6-PHOSPHOFRUCTOKINASE, PLATELET TYPE (PTHR13697:SF48)
HUMAN|HGNC=29932|UniProtKB=Q13057	HUMAN|HGNC=29932|UniProtKB=Q13057	Bifunctional coenzyme A synthase;COASY;ortholog	BIFUNCTIONAL COENZYME A SYNTHASE (PTHR10695:SF42)
	HUMAN|HGNC=1469|UniProtKB=Q8N5S9	Calcium/calmodulin-dependent protein kinase kinase 1;CAMKK1;ortholog	CALCIUM/CALMODULIN-DEPENDENT PROTEIN KINASE KINASE 1 (PTHR24347:SF330)
	HUMAN|HGNC=1459|UniProtKB=Q14012	Calcium/calmodulin-dependent protein kinase type 1;CAMK1;ortholog	CALCIUM/CALMODULIN-DEPENDENT PROTEIN KINASE TYPE 1 (PTHR24347:SF344)
HUMAN|HGNC=1460|UniProtKB=Q9UQM7	HUMAN|HGNC=1460|UniProtKB=Q9UQM7	Calcium/calmodulin-dependent protein kinase type II subunit alpha;CAMK2A;ortholog	CALCIUM/CALMODULIN-DEPENDENT PROTEIN KINASE TYPE II SUBUNIT ALPHA (PTHR24347:SF318)
	HUMAN|HGNC=28204|UniProtKB=Q9BSD7	Cancer-related nucleoside-triphosphatase;NTPCR;ortholog	CANCER-RELATED NUCLEOSIDE-TRIPHOSPHATASE (PTHR43146:SF1)
HUMAN|HGNC=13518|UniProtKB=Q9Y696		Chloride intracellular channel protein 4;CLIC4;ortholog	CHLORIDE INTRACELLULAR CHANNEL PROTEIN 4 (PTHR11260:SF443)
HUMAN|HGNC=1991|UniProtKB=P12277	HUMAN|HGNC=1991|UniProtKB=P12277	Creatine kinase B-type;CKB;ortholog	CREATINE KINASE B-TYPE (PTHR11547:SF41)

HUMAN|HGNC=1995|UniProtKB=P12532		Creatine kinase U-type, mitochondrial;CKMT1A;ortholog	CREATINE KINASE U-TYPE, MITOCHONDRIAL (PTHR11547:SF46)
HUMAN|HGNC=31736|UniProtKB=P12532		Creatine kinase U-type, mitochondrial;CKMT1A;ortholog	CREATINE KINASE U-TYPE, MITOCHONDRIAL (PTHR11547:SF46)
	HUMAN|HGNC=8751|UniProtKB=Q07002	Cyclin-dependent kinase 18;CDK18;ortholog	CYCLIN-DEPENDENT KINASE 18 (PTHR24056:SF264)
HUMAN|HGNC=1774|UniProtKB=Q00535	HUMAN|HGNC=1774|UniProtKB=Q00535	Cyclin-dependent-like kinase 5;CDK5;ortholog	CYCLIN-DEPENDENT-LIKE KINASE 5 (PTHR24056:SF283)
HUMAN|HGNC=3149|UniProtKB=Q13011		Delta(3,5)-Delta(2,4)-dienoyl-CoA isomerase, mitochondrial;ECH1;ortholog	DELTA(3,5)-DELTA(2,4)-DIENOYL-COA ISOMERASE, MITOCHONDRIAL (PTHR43149:SF3)
	HUMAN|HGNC=2896|UniProtKB=P10515	Dihydrolipoyllysine-residue acetyltransferase component of pyruvate dehydrogenase complex, mitochondrial;DLAT;ortholog	DIHYDROLIPOYLLYSINE-RESIDUE ACETYLTRANSFERASE COMPONENT OF PYRUVATE DEHYDROGENASE COMPLEX, MITOCHONDRIAL (PTHR23151:SF73)
HUMAN|HGNC=2911|UniProtKB=P36957	HUMAN|HGNC=2911|UniProtKB=P36957	Dihydrolipoyllysine-residue succinyltransferase component of 2-oxoglutarate dehydrogenase complex, mitochondrial;DLST;ortholog	DIHYDROLIPOYLLYSINE-RESIDUE SUCCINYLTRANSFERASE COMPONENT OF 2-OXOGLUTARATE DEHYDROGENASE COMPLEX, MITOCHONDRIAL (PTHR43416:SF7)
HUMAN|HGNC=10381|UniProtKB=P04843	HUMAN|HGNC=10381|UniProtKB=P04843	Dolichyl-diphosphooligosaccharide--protein glycosyltransferase subunit 1;RPN1;ortholog	DOLICHYL-DIPHOSPHOOLIGOSACCHARIDE--PROTEIN GLYCOSYLTRANSFERASE SUBUNIT 1 (PTHR21049:SF3)
HUMAN|HGNC=3213|UniProtKB=P26641	HUMAN|HGNC=3213|UniProtKB=P26641	Elongation factor 1-gamma;EEF1G;ortholog	ELONGATION FACTOR 1-GAMMA (PTHR11260:SF420)
HUMAN|HGNC=3151|UniProtKB=P30084		Enoyl-CoA hydratase, mitochondrial;ECHS1;ortholog	ENOYL-COA HYDRATASE, MITOCHONDRIAL (PTHR11941:SF111)
HUMAN|HGNC=3594|UniProtKB=P49327	HUMAN|HGNC=3594|UniProtKB=P49327	Fatty acid synthase;FASN;ortholog	FATTY ACID SYNTHASE (PTHR43775:SF2)
	HUMAN|HGNC=4118|UniProtKB=P51570	Galactokinase;GALK1;ortholog	GALACTOKINASE (PTHR10457:SF17)
HUMAN|HGNC=15968|UniProtKB=Q8TB36		Ganglioside-induced differentiation-associated protein 1;GDAP1;ortholog	GANGLIOSIDE-INDUCED DIFFERENTIATION-ASSOCIATED PROTEIN 1 (PTHR11260:SF391)
HUMAN|HGNC=9723|UniProtKB=P11216	HUMAN|HGNC=9723|UniProtKB=P11216	Glycogen phosphorylase, brain form;PYGB;ortholog	GLYCOGEN PHOSPHORYLASE, BRAIN FORM (PTHR11468:SF19)
	HUMAN|HGNC=4617|UniProtKB=P49841	Glycogen synthase kinase-3 beta;GSK3B;ortholog	GLYCOGEN SYNTHASE KINASE-3 BETA (PTHR24057:SF26)
HUMAN|HGNC=17376|UniProtKB=Q9UIJ7		GTP:AMP phosphotransferase AK3, mitochondrial;AK3;ortholog	GTP:AMP PHOSPHOTRANSFERASE AK3, MITOCHONDRIAL (PTHR23359:SF110)
HUMAN|HGNC=19022|UniProtKB=O43301	HUMAN|HGNC=19022|UniProtKB=O43301	Heat shock 70 kDa protein 12A;HSPA12A;ortholog	HEAT SHOCK 70 KDA PROTEIN 12A (PTHR14187:SF63)
HUMAN|HGNC=26361|UniProtKB=Q14CZ8		Hepatocyte cell adhesion molecule;HEPACAM;ortholog	HEPATOCYTE CELL ADHESION MOLECULE (PTHR12080:SF66)
	HUMAN|HGNC=5007|UniProtKB=Q01581	Hydroxymethylglutaryl-CoA synthase, cytoplasmic;HMGCS1;ortholog	HYDROXYMETHYLGLUTARYL-COA SYNTHASE, CYTOPLASMIC (PTHR43323:SF6)
HUMAN|HGNC=5157|UniProtKB=P00492		Hypoxanthine-guanine phosphoribosyltransferase;HPRT1;ortholog	HYPOXANTHINE-GUANINE PHOSPHORIBOSYLTRANSFERASE (PTHR43340:SF7)
	HUMAN|HGNC=6178|UniProtKB=P23677	Inositol-trisphosphate 3-kinase A;ITPKA;ortholog	INOSITOL-TRISPHOSPHATE 3-KINASE A (PTHR12400:SF62)
HUMAN|HGNC=2698|UniProtKB=P11182	HUMAN|HGNC=2698|UniProtKB=P11182	Lipoamide acyltransferase component of branched-chain alpha-keto acid dehydrogenase complex, mitochondrial;DBT;ortholog	LIPOAMIDE ACYLTRANSFERASE COMPONENT OF BRANCHED-CHAIN ALPHA-KETO ACID DEHYDROGENASE COMPLEX, MITOCHONDRIAL (PTHR43178:SF3)
	HUMAN|HGNC=6990|UniProtKB=P51608	Methyl-CpG-binding protein 2;MECP2;ortholog	METHYL-CPG-BINDING PROTEIN 2 (PTHR15074:SF7)
HUMAN|HGNC=7064|UniProtKB=O14880		Microsomal glutathione S-transferase 3;MGST3;ortholog	MICROSOMAL GLUTATHIONE S-TRANSFERASE 3 (PTHR10250:SF22)

HUMAN|HGNC=6871|UniProtKB=P28482		Mitogen-activated protein kinase 1;MAPK1;ortholog	MITOGEN-ACTIVATED PROTEIN KINASE 1 (PTHR24055:SF249)
	HUMAN|HGNC=6877|UniProtKB=P27361	Mitogen-activated protein kinase 3;MAPK3;ortholog	MITOGEN-ACTIVATED PROTEIN KINASE 3 (PTHR24055:SF269)
HUMAN|HGNC=6984|UniProtKB=P23368		NAD-dependent malic enzyme, mitochondrial;ME2;ortholog	NAD-DEPENDENT MALIC ENZYME, MITOCHONDRIAL (PTHR23406:SF50)
HUMAN|HGNC=8091|UniProtKB=P04181		Ornithine aminotransferase, mitochondrial;OAT;ortholog	ORNITHINE AMINOTRANSFERASE, MITOCHONDRIAL (PTHR11986:SF100)
	HUMAN|HGNC=8983|UniProtKB=P42356	Phosphatidylinositol 4-kinase alpha;PI4KA;ortholog	PHOSPHATIDYLINOSITOL 4-KINASE ALPHA-RELATED (PTHR10048:SF86)
HUMAN|HGNC=8998|UniProtKB=P78356	HUMAN|HGNC=8998|UniProtKB=P78356	Phosphatidylinositol 5-phosphate 4-kinase type-2 beta;PIP4K2B;ortholog	PHOSPHATIDYLINOSITOL 5-PHOSPHATE 4-KINASE TYPE-2 BETA (PTHR23086:SF66)
	HUMAN|HGNC=23786|UniProtKB=Q8TBX8	Phosphatidylinositol 5-phosphate 4-kinase type-2 gamma;PIP4K2C;ortholog	PHOSPHATIDYLINOSITOL 5-PHOSPHATE 4-KINASE TYPE-2 GAMMA (PTHR23086:SF76)
HUMAN|HGNC=8905|UniProtKB=P36871		Phosphoglucomutase-1;PGM1;ortholog	PHOSPHOGLUCOMUTASE-1 (PTHR22573:SF59)
HUMAN|HGNC=19129|UniProtKB=Q9Y617		Phosphoserine aminotransferase;PSAT1;ortholog	PHOSPHOSERINE AMINOTRANSFERASE (PTHR43247:SF2)
HUMAN|HGNC=9393|UniProtKB=P17252	HUMAN|HGNC=9393|UniProtKB=P17252	Protein kinase C alpha type;PRKCA;ortholog	PROTEIN KINASE C ALPHA TYPE (PTHR24356:SF270)
	HUMAN|HGNC=9395|UniProtKB=P05771	Protein kinase C beta type;PRKCB;ortholog	PROTEIN KINASE C BETA TYPE (PTHR24356:SF244)
	HUMAN|HGNC=9401|UniProtKB=Q02156	Protein kinase C epsilon type;PRKCE;ortholog	PROTEIN KINASE C EPSILON TYPE (PTHR24356:SF275)
	HUMAN|HGNC=9402|UniProtKB=P05129	Protein kinase C gamma type;PRKCG;ortholog	PROTEIN KINASE C GAMMA TYPE (PTHR24356:SF293)
	HUMAN|HGNC=11778|UniProtKB=P21980	Protein-glutamine gamma-glutamyltransferase 2;TGM2;ortholog	PROTEIN-GLUTAMINE GAMMA-GLUTAMYLTRANSFERASE 2 (PTHR11590:SF54)
HUMAN|HGNC=8728|UniProtKB=P22061	HUMAN|HGNC=8728|UniProtKB=P22061	Protein-L-isoaspartate(D-aspartate) O-methyltransferase;PCMT1;ortholog	PROTEIN-L-ISOASPARTATE(D-ASPARTATE) O-METHYLTRANSFERASE (PTHR11579:SF14)
	HUMAN|HGNC=49897|UniProtKB=Q6A1A2	Putative 3-phosphoinositide-dependent protein kinase 2;PDPK2P;ortholog	3-PHOSPHOINOSITIDE-DEPENDENT PROTEIN KINASE 1-RELATED (PTHR24356:SF290)
HUMAN|HGNC=8819|UniProtKB=O00764	HUMAN|HGNC=8819|UniProtKB=O00764	Pyridoxal kinase;PDXK;ortholog	PYRIDOXAL KINASE (PTHR10534:SF10)
HUMAN|HGNC=4226|UniProtKB=P31150	HUMAN|HGNC=4226|UniProtKB=P31150	Rab GDP dissociation inhibitor alpha;GDI1;ortholog	RAB GDP DISSOCIATION INHIBITOR ALPHA (PTHR11787:SF19)
HUMAN|HGNC=4227|UniProtKB=P50395		Rab GDP dissociation inhibitor beta;GDI2;ortholog	RAB GDP DISSOCIATION INHIBITOR BETA (PTHR11787:SF21)
HUMAN|HGNC=9462|UniProtKB=P60891		Ribose-phosphate pyrophosphokinase 1;PRPS1;ortholog	RIBOSE-PHOSPHATE PYROPHOSPHOKINASE 1 (PTHR10210:SF68)
	HUMAN|HGNC=10432|UniProtKB=P51812	Ribosomal protein S6 kinase alpha-3;RPS6KA3;ortholog	RIBOSOMAL PROTEIN S6 KINASE ALPHA-3 (PTHR24351:SF133)
	HUMAN|HGNC=21332|UniProtKB=Q86UX6	Serine/threonine-protein kinase 32C;STK32C;ortholog	SERINE/THREONINE-PROTEIN KINASE 32C (PTHR24356:SF232)
	HUMAN|HGNC=17848|UniProtKB=Q9Y2H1	Serine/threonine-protein kinase 38-like;STK38L;ortholog	SERINE/THREONINE-PROTEIN KINASE 38-LIKE (PTHR24356:SF259)
	HUMAN|HGNC=1097|UniProtKB=P15056	Serine/threonine-protein kinase B-raf;BRAF;ortholog	SERINE/THREONINE-PROTEIN KINASE B-RAF (PTHR23257:SF696)
	HUMAN|HGNC=13386|UniProtKB=Q8TDX7	Serine/threonine-protein kinase Nek7;NEK7;ortholog	SERINE/THREONINE-PROTEIN KINASE NEK7 (PTHR43289:SF2)
	HUMAN|HGNC=11584|UniProtKB=Q9UHD2	Serine/threonine-protein kinase TBK1;TBK1;ortholog	SERINE/THREONINE-PROTEIN KINASE TBK1 (PTHR22969:SF23)
HUMAN|HGNC=8527|UniProtKB=P55809	HUMAN|HGNC=8527|UniProtKB=P55809	Succinyl-CoA:3-ketoacid coenzyme A transferase 1, mitochondrial;OXCT1;ortholog	SUCCINYL-COA:3-KETOACID COENZYME A TRANSFERASE 1, MITOCHONDRIAL (PTHR13707:SF45)

HUMAN|HGNC=11834|UniProtKB=P29401		Transketolase;TKT;ortholog	TRANSKETOLASE (PTHR43195:SF3)
	HUMAN|HGNC=4037|UniProtKB=P06241	Tyrosine-protein kinase Fyn;FYN;ortholog	TYROSINE-PROTEIN KINASE FYN (PTHR24418:SF291)
	HUMAN|HGNC=12841|UniProtKB=P07947	Tyrosine-protein kinase Yes;YES1;ortholog	TYROSINE-PROTEIN KINASE YES (PTHR24418:SF315)
HUMAN|HGNC=18170|UniProtKB=P30085	HUMAN|HGNC=18170|UniProtKB=P30085	UMP-CMP kinase;CMPK1;ortholog	UMP-CMP KINASE (PTHR23359:SF123)
	HUMAN|Ensembl=ENSG00000268643|UniProtKB=M0QYV0	Uncharacterized protein (Fragment);unassigned;ortholog	GLYCOGEN SYNTHASE KINASE-3 ALPHA (PTHR24057:SF33)
HUMAN|HGNC=857|UniProtKB=P36543	HUMAN|HGNC=857|UniProtKB=P36543	V-type proton ATPase subunit E 1;ATP6V1E1;ortholog	V-TYPE PROTON ATPASE SUBUNIT E 1 (PTHR12317:SF40)
**transmembrane receptor regulatory/adaptor protein (PC00226)**			
HUMAN|HGNC=555|UniProtKB=O43747		AP-1 complex subunit gamma-1;AP1G1;ortholog	AP-1 COMPLEX SUBUNIT GAMMA-1 (PTHR22780:SF35)
HUMAN|HGNC=562|UniProtKB=O94973	HUMAN|HGNC=562|UniProtKB=O94973	AP-2 complex subunit alpha-2;AP2A2;ortholog	AP-2 COMPLEX SUBUNIT ALPHA-2 (PTHR22780:SF34)
	HUMAN|HGNC=20879|UniProtKB=Q8WXD9	Caskin-1;CASKIN1;ortholog	CASKIN-1 (PTHR24177:SF98)
	HUMAN|HGNC=30368|UniProtKB=O95886	Disks large-associated protein 3;DLGAP3;ortholog	DISKS LARGE-ASSOCIATED PROTEIN 3 (PTHR12353:SF28)
HUMAN|HGNC=17867|UniProtKB=Q9Y639		Neuroplastin;NPTN;ortholog	NEUROPLASTIN (PTHR10075:SF20)
**transporter (PC00227)**			
HUMAN|HGNC=10990|UniProtKB=P12235	HUMAN|HGNC=10990|UniProtKB=P12235	ADP/ATP translocase 1;SLC25A4;ortholog	ADP/ATP TRANSLOCASE 1 (PTHR24089:SF506)
HUMAN|HGNC=10991|UniProtKB=P05141	HUMAN|HGNC=10991|UniProtKB=P05141	ADP/ATP translocase 2;SLC25A5;ortholog	ADP/ATP TRANSLOCASE 2 (PTHR24089:SF446)
HUMAN|HGNC=10992|UniProtKB=P12236	HUMAN|HGNC=10992|UniProtKB=P12236	ADP/ATP translocase 3;SLC25A6;ortholog	ADP/ATP TRANSLOCASE 3 (PTHR24089:SF411)
	HUMAN|HGNC=619|UniProtKB=Q9BQE5	Apolipoprotein L2;APOL2;ortholog	APOLIPOPROTEIN L1-RELATED (PTHR14096:SF49)
HUMAN|HGNC=823|UniProtKB=P25705	HUMAN|HGNC=823|UniProtKB=P25705	ATP synthase subunit alpha, mitochondrial;ATP5A1;ortholog	ATP SYNTHASE SUBUNIT ALPHA, MITOCHONDRIAL (PTHR43089:SF1)
HUMAN|HGNC=830|UniProtKB=P06576	HUMAN|HGNC=830|UniProtKB=P06576	ATP synthase subunit beta, mitochondrial;ATP5B;ortholog	ATP SYNTHASE SUBUNIT BETA, MITOCHONDRIAL (PTHR15184:SF59)
HUMAN|HGNC=850|UniProtKB=P48047	HUMAN|HGNC=850|UniProtKB=P48047	ATP synthase subunit O, mitochondrial;ATP5O;ortholog	ATP SYNTHASE SUBUNIT O, MITOCHONDRIAL (PTHR11910:SF9)
HUMAN|HGNC=10982|UniProtKB=O75746	HUMAN|HGNC=10982|UniProtKB=O75746	Calcium-binding mitochondrial carrier protein Aralar1;SLC25A12;ortholog	CALCIUM-BINDING MITOCHONDRIAL CARRIER PROTEIN ARALAR1 (PTHR24089:SF503)
HUMAN|HGNC=8011|UniProtKB=P78357	HUMAN|HGNC=8011|UniProtKB=P78357	Contactin-associated protein 1;CNTNAP1;ortholog	CONTACTIN-ASSOCIATED PROTEIN 1 (PTHR10127:SF740)
	HUMAN|HGNC=4572|UniProtKB=P42262	Glutamate receptor 2;GRIA2;ortholog	GLUTAMATE RECEPTOR 2 (PTHR18966:SF269)
HUMAN|HGNC=6400|UniProtKB=Q14974	HUMAN|HGNC=6400|UniProtKB=Q14974	Importin subunit beta-1;KPNB1;ortholog	IMPORTIN SUBUNIT BETA-1 (PTHR10527:SF32)
HUMAN|HGNC=9852|UniProtKB=O95373		Importin-7;IPO7;ortholog	IMPORTIN-7 (PTHR10997:SF39)
HUMAN|HGNC=15714|UniProtKB=P42704	HUMAN|HGNC=15714|UniProtKB=P42704	Leucine-rich PPR motif-containing protein, mitochondrial;LRPPRC;ortholog	LEUCINE-RICH PPR MOTIF-CONTAINING PROTEIN, MITOCHONDRIAL (PTHR24015:SF1304)
HUMAN|HGNC=19954|UniProtKB=Q9H936	HUMAN|HGNC=19954|UniProtKB=Q9H936	Mitochondrial glutamate carrier 1;SLC25A22;ortholog	MITOCHONDRIAL GLUTAMATE CARRIER 1 (PTHR24089:SF598)
	HUMAN|HGNC=10988|UniProtKB=Q9H1K4	Mitochondrial glutamate carrier 2;SLC25A18;ortholog	MITOCHONDRIAL GLUTAMATE CARRIER 2 (PTHR24089:SF482)
HUMAN|HGNC=10942|UniProtKB=P43007		Neutral amino acid transporter A;SLC1A4;ortholog	NEUTRAL AMINO ACID TRANSPORTER A (PTHR11958:SF78)

HUMAN|HGNC=28911|UniProtKB=O60831		PRA1 family protein 2;PRAF2;ortholog	PRA1 FAMILY PROTEIN 2 (PTHR12859:SF4)
HUMAN|HGNC=30077|UniProtKB=Q00765		Receptor expression-enhancing protein 5;REEP5;ortholog	RECEPTOR EXPRESSION-ENHANCING PROTEIN 5 (PTHR12300:SF91)
HUMAN|HGNC=16085|UniProtKB=Q9H9B4	HUMAN|HGNC=16085|UniProtKB=Q9H9B4	Sideroflexin-1;SFXN1;ortholog	SIDEROFLEXIN-1 (PTHR11153:SF32)
	HUMAN|HGNC=10589|UniProtKB=O60939	Sodium channel subunit beta-2;SCN2B;ortholog	SODIUM CHANNEL SUBUNIT BETA-2 (PTHR13869:SF34)
HUMAN|HGNC=805|UniProtKB=P14415	HUMAN|HGNC=805|UniProtKB=P14415	Sodium/potassium-transporting ATPase subunit beta-2;ATP1B2;ortholog	SODIUM/POTASSIUM-TRANSPORTING ATPASE SUBUNIT BETA-2 (PTHR11523:SF36)
HUMAN|HGNC=11444|UniProtKB=P61764	HUMAN|HGNC=11444|UniProtKB=P61764	Syntaxin-binding protein 1;STXBP1;ortholog	SYNTAXIN-BINDING PROTEIN 1 (PTHR11679:SF54)
HUMAN|HGNC=11446|UniProtKB=O00186		Syntaxin-binding protein 3;STXBP3;ortholog	SYNTAXIN-BINDING PROTEIN 3 (PTHR11679:SF44)
HUMAN|HGNC=10979|UniProtKB=P53007	HUMAN|HGNC=10979|UniProtKB=P53007	Tricarboxylate transport protein, mitochondrial;SLC25A1;ortholog	TRICARBOXYLATE TRANSPORT PROTEIN, MITOCHONDRIAL (PTHR24089:SF459)
HUMAN|HGNC=854|UniProtKB=P21281	HUMAN|HGNC=854|UniProtKB=P21281	V-type proton ATPase subunit B, brain isoform;ATP6V1B2;ortholog	V-TYPE PROTON ATPASE SUBUNIT B, BRAIN ISOFORM (PTHR43389:SF1)
HUMAN|HGNC=856|UniProtKB=P21283	HUMAN|HGNC=856|UniProtKB=P21283	V-type proton ATPase subunit C 1;ATP6V1C1;ortholog	V-TYPE PROTON ATPASE SUBUNIT C 1 (PTHR10137:SF6)
HUMAN|HGNC=862|UniProtKB=O95670		V-type proton ATPase subunit G 2;ATP6V1G2;ortholog	PROTEIN ATP6V1G2-DDX39B-RELATED (PTHR12713:SF17)
HUMAN|HGNC=12669|UniProtKB=P21796	HUMAN|HGNC=12669|UniProtKB=P21796	Voltage-dependent anion-selective channel protein 1;VDAC1;ortholog	VOLTAGE-DEPENDENT ANION-SELECTIVE CHANNEL PROTEIN 1 (PTHR11743:SF37)
HUMAN|HGNC=12674|UniProtKB=Q9Y277	HUMAN|HGNC=12674|UniProtKB=Q9Y277	Voltage-dependent anion-selective channel protein 3;VDAC3;ortholog	VOLTAGE-DEPENDENT ANION-SELECTIVE CHANNEL PROTEIN 3 (PTHR11743:SF42)

Supplementary Table IV. Comparison of fungal peptides in P2 and P7 fractions in AD2.

GENE ID P2	GENE ID P7	GENE NAME GENE SYMBOL ORTHOLOG	PANTHER FAMILY/SUBFAMILY
**calcium-binding protein (PC00060)**			
HUMAN|HGNC=10990|UniProtKB=P12235	HUMAN|HGNC=10990|UniProtKB=P12235	ADP/ATP translocase 1;SLC25A4;ortholog	ADP/ATP TRANSLOCASE 1 (PTHR24089:SF506)
HUMAN|HGNC=10991|UniProtKB=P05141	HUMAN|HGNC=10991|UniProtKB=P05141	ADP/ATP translocase 2;SLC25A5;ortholog	ADP/ATP TRANSLOCASE 2 (PTHR24089:SF446)
HUMAN|HGNC=10992|UniProtKB=P12236	HUMAN|HGNC=10992|UniProtKB=P12236	ADP/ATP translocase 3;SLC25A6;ortholog	ADP/ATP TRANSLOCASE 3 (PTHR24089:SF411)
HUMAN|HGNC=10982|UniProtKB=O75746	HUMAN|HGNC=10982|UniProtKB=O75746	Calcium-binding mitochondrial carrier protein Aralar1;SLC25A12;ortholog	CALCIUM-BINDING MITOCHONDRIAL CARRIER PROTEIN ARALAR1 (PTHR24089:SF503)
	HUMAN|HGNC=10983|UniProtKB=Q9UJS0	Calcium-binding mitochondrial carrier protein Aralar2;SLC25A13;ortholog	CALCIUM-BINDING MITOCHONDRIAL CARRIER PROTEIN ARALAR2 (PTHR24089:SF428)
HUMAN|HGNC=1442|UniProtKB=P62158		Calmodulin;CALM1;ortholog	CALMODULIN (PTHR23050:SF234)
HUMAN|HGNC=1445|UniProtKB=P62158		Calmodulin;CALM1;ortholog	CALMODULIN (PTHR23050:SF239)
HUMAN|HGNC=1449|UniProtKB=P62158		Calmodulin;CALM1;ortholog	CALMODULIN (PTHR23050:SF245)
HUMAN|HGNC=1473|UniProtKB=P27824		Calnexin;CANX;ortholog	CALNEXIN (PTHR11073:SF29)
HUMAN|HGNC=1476|UniProtKB=P07384		Calpain-1 catalytic subunit;CAPN1;ortholog	CALPAIN-1 CATALYTIC SUBUNIT (PTHR10183:SF343)
HUMAN|HGNC=1455|UniProtKB=P27797		Calreticulin;CALR;ortholog	CALRETICULIN (PTHR11073:SF21)

	HUMAN|HGNC=23360|UniProtKB=Q8N163	Cell cycle and apoptosis regulator protein 2;CCAR2;ortholog	CELL CYCLE AND APOPTOSIS REGULATOR PROTEIN 2 (PTHR14304:SF15)
	HUMAN|HGNC=3243|UniProtKB=Q9NZN4	EH domain-containing protein 2;EHD2;ortholog	EH DOMAIN-CONTAINING PROTEIN 2 (PTHR11216:SF106)
HUMAN|HGNC=3244|UniProtKB=Q9NZN3	HUMAN|HGNC=3244|UniProtKB=Q9NZN3	EH domain-containing protein 3;EHD3;ortholog	EH DOMAIN-CONTAINING PROTEIN 3 (PTHR11216:SF88)
HUMAN|HGNC=6556|UniProtKB=O95202	HUMAN|HGNC=6556|UniProtKB=O95202	LETM1 and EF-hand domain-containing protein 1, mitochondrial;LETM1;ortholog	LETM1 AND EF-HAND DOMAIN-CONTAINING PROTEIN 1, MITOCHONDRIAL (PTHR14009:SF18)
	HUMAN|HGNC=19954|UniProtKB=Q9H936	Mitochondrial glutamate carrier 1;SLC25A22;ortholog	MITOCHONDRIAL GLUTAMATE CARRIER 1 (PTHR24089:SF598)
	HUMAN|HGNC=10988|UniProtKB=Q9H1K4	Mitochondrial glutamate carrier 2;SLC25A18;ortholog	MITOCHONDRIAL GLUTAMATE CARRIER 2 (PTHR24089:SF482)
HUMAN|HGNC=30009|UniProtKB=Q9UBV8		Peflin;PEF1;ortholog	PEFLIN (PTHR10183:SF361)
HUMAN|HGNC=3719|UniProtKB=Q00688		Peptidyl-prolyl cis-trans isomerase FKBP3;FKBP3;ortholog	PEPTIDYL-PROLYL CIS-TRANS ISOMERASE FKBP3 (PTHR10516:SF356)
HUMAN|HGNC=3720|UniProtKB=Q02790		Peptidyl-prolyl cis-trans isomerase FKBP4;FKBP4;ortholog	PEPTIDYL-PROLYL CIS-TRANS ISOMERASE FKBP4 (PTHR10516:SF332)
HUMAN|HGNC=3724|UniProtKB=Q14318		Peptidyl-prolyl cis-trans isomerase FKBP8;FKBP8;ortholog	PEPTIDYL-PROLYL CIS-TRANS ISOMERASE FKBP8 (PTHR10516:SF363)
HUMAN|HGNC=8765|UniProtKB=O75340	HUMAN|HGNC=8765|UniProtKB=O75340	Programmed cell death protein 6;PDCD6;ortholog	PROGRAMMED CELL DEATH PROTEIN 6 (PTHR10183:SF367)
HUMAN|HGNC=29180|UniProtKB=Q9UPV7	HUMAN|HGNC=29180|UniProtKB=Q9UPV7	Protein KIAA1045;KIAA1045;ortholog	PROTEIN KIAA1045 (PTHR10891:SF706)
HUMAN|HGNC=9393|UniProtKB=P17252		Protein kinase C alpha type;PRKCA;ortholog	PROTEIN KINASE C ALPHA TYPE (PTHR24356:SF270)
HUMAN|HGNC=9395|UniProtKB=P05771	HUMAN|HGNC=9395|UniProtKB=P05771	Protein kinase C beta type;PRKCB;ortholog	PROTEIN KINASE C BETA TYPE (PTHR24356:SF244)
	HUMAN|HGNC=9401|UniProtKB=Q02156	Protein kinase C epsilon type;PRKCE;ortholog	PROTEIN KINASE C EPSILON TYPE (PTHR24356:SF275)
	HUMAN|HGNC=49897|UniProtKB=Q6A1A2	Putative 3-phosphoinositide-dependent protein kinase 2;PDPK2P;ortholog	3-PHOSPHOINOSITIDE-DEPENDENT PROTEIN KINASE 1-RELATED (PTHR24356:SF290)
HUMAN|HGNC=21332|UniProtKB=Q86UX6		Serine/threonine-protein kinase 32C;STK32C;ortholog	SERINE/THREONINE-PROTEIN KINASE 32C (PTHR24356:SF232)
HUMAN|HGNC=9282|UniProtKB=P62140	HUMAN|HGNC=9282|UniProtKB=P62140	Serine/threonine-protein phosphatase PP1-beta catalytic subunit;PPP1CB;ortholog	SERINE/THREONINE-PROTEIN PHOSPHATASE PP1-BETA CATALYTIC SUBUNIT (PTHR11668:SF346)
HUMAN|HGNC=10979|UniProtKB=P53007	HUMAN|HGNC=10979|UniProtKB=P53007	Tricarboxylate transport protein, mitochondrial;SLC25A1;ortholog	TRICARBOXYLATE TRANSPORT PROTEIN, MITOCHONDRIAL (PTHR24089:SF459)

cell adhesion molecule (PC00069)

HUMAN|HGNC=8011|UniProtKB=P78357	HUMAN|HGNC=8011|UniProtKB=P78357	Contactin-associated protein 1;CNTNAP1;ortholog	CONTACTIN-ASSOCIATED PROTEIN 1 (PTHR10127:SF740)
HUMAN|HGNC=6561|UniProtKB=P09382		Galectin-1;LGALS1;ortholog	GALECTIN-1 (PTHR11346:SF131)
HUMAN|HGNC=26361|UniProtKB=Q14CZ8		Hepatocyte cell adhesion molecule;HEPACAM;ortholog	HEPATOCYTE CELL ADHESION MOLECULE (PTHR12080:SF66)
HUMAN|HGNC=28246|UniProtKB=Q96ID5		Immunoglobulin superfamily member 21;IGSF21;ortholog	IMMUNOGLOBULIN SUPERFAMILY MEMBER 21 (PTHR10489:SF771)
HUMAN|HGNC=5348|UniProtKB=Q9UMF0	HUMAN|HGNC=5348|UniProtKB=Q9UMF0	Intercellular adhesion molecule 5;ICAM5;ortholog	INTERCELLULAR ADHESION MOLECULE 5 (PTHR13771:SF15)
HUMAN|HGNC=7656|UniProtKB=P13591		Neural cell adhesion molecule 1;NCAM1;ortholog	NEURAL CELL ADHESION MOLECULE 1 (PTHR10489:SF783)
	HUMAN|HGNC=17787|UniProtKB=O14910	Protein lin-7 homolog A;LIN7A;ortholog	PROTEIN LIN-7 HOMOLOG A (PTHR14063:SF10)
HUMAN|HGNC=8655|UniProtKB=Q08174		Protocadherin-1;PCDH1;ortholog	PROTOCADHERIN-1 (PTHR24027:SF383)
HUMAN|HGNC=10589|UniProtKB=O60939		Sodium channel subunit beta-2;SCN2B;ortholog	SODIUM CHANNEL SUBUNIT BETA-2 (PTHR13869:SF34)
	HUMAN|HGNC=11845|UniProtKB=Q9Y490	Talin-1;TLN1;ortholog	TALIN-1 (PTHR19981:SF24)

	HUMAN|HGNC=15447|UniProtKB=Q9Y4G6	Talin-2;TLN2;ortholog	TALIN-2 (PTHR19981:SF28)
**cell junction protein (PC00070)**			
HUMAN|HGNC=8514|UniProtKB=O75508	HUMAN|HGNC=8514|UniProtKB=O75508	Claudin-11;CLDN11;ortholog	CLAUDIN-11 (PTHR12002:SF153)
HUMAN|HGNC=4274|UniProtKB=P17302	HUMAN|HGNC=4274|UniProtKB=P17302	Gap junction alpha-1 protein;GJA1;ortholog	GAP JUNCTION ALPHA-1 PROTEIN (PTHR11984:SF70)
HUMAN|HGNC=7568|UniProtKB=P35580	HUMAN|HGNC=7568|UniProtKB=P35580	Myosin-10;MYH10;ortholog	MYOSIN-10 (PTHR13140:SF549)
	HUMAN|HGNC=17787|UniProtKB=O14910	Protein lin-7 homolog A;LIN7A;ortholog	PROTEIN LIN-7 HOMOLOG A (PTHR14063:SF10)
**chaperone (PC00072)**			
HUMAN|HGNC=5269|UniProtKB=P61604	HUMAN|HGNC=5269|UniProtKB=P61604	10 kDa heat shock protein, mitochondrial;HSPE1;ortholog	10 KDA HEAT SHOCK PROTEIN, MITOCHONDRIAL (PTHR10772:SF21)
HUMAN|HGNC=12851|UniProtKB=P62258		14-3-3 protein epsilon;YWHAE;ortholog	14-3-3 PROTEIN EPSILON (PTHR18860:SF47)
HUMAN|HGNC=12853|UniProtKB=Q04917	HUMAN|HGNC=12853|UniProtKB=Q04917	14-3-3 protein eta;YWHAH;ortholog	14-3-3 PROTEIN ETA (PTHR18860:SF42)
HUMAN|HGNC=12852|UniProtKB=P61981	HUMAN|HGNC=12852|UniProtKB=P61981	14-3-3 protein gamma;YWHAG;ortholog	14-3-3 PROTEIN GAMMA (PTHR18860:SF41)
HUMAN|HGNC=12854|UniProtKB=P27348	HUMAN|HGNC=12854|UniProtKB=P27348	14-3-3 protein theta;YWHAQ;ortholog	14-3-3 PROTEIN THETA (PTHR18860:SF44)
HUMAN|HGNC=12855|UniProtKB=P63104	HUMAN|HGNC=12855|UniProtKB=P63104	14-3-3 protein zeta/delta;YWHAZ;ortholog	14-3-3 PROTEIN ZETA/DELTA (PTHR18860:SF45)
HUMAN|HGNC=5261|UniProtKB=P10809	HUMAN|HGNC=5261|UniProtKB=P10809	60 kDa heat shock protein, mitochondrial;HSPD1;ortholog	60 KDA HEAT SHOCK PROTEIN, MITOCHONDRIAL (PTHR11353:SF118)
	HUMAN|HGNC=1189|UniProtKB=O95433	Activator of 90 kDa heat shock protein ATPase homolog 1;AHSA1;ortholog	ACTIVATOR OF 90 KDA HEAT SHOCK PROTEIN ATPASE HOMOLOG 1 (PTHR13009:SF14)
HUMAN|HGNC=1473|UniProtKB=P27824		Calnexin;CANX;ortholog	CALNEXIN (PTHR11073:SF29)
	HUMAN|HGNC=2586|UniProtKB=Q9UDT6	CAP-Gly domain-containing linker protein 2;CLIP2;ortholog	CAP-GLY DOMAIN-CONTAINING LINKER PROTEIN 2 (PTHR18916:SF45)
	HUMAN|HGNC=14886|UniProtKB=Q9UDY4	DnaJ homolog subfamily B member 4;DNAJB4;ortholog	DNAJ HOMOLOG SUBFAMILY B MEMBER 4 (PTHR24078:SF391)
HUMAN|HGNC=12028|UniProtKB=P14625		Endoplasmin;HSP90B1;ortholog	ENDOPLASMIN-RELATED (PTHR11528:SF66)
HUMAN|HGNC=11141|UniProtKB=O76070		Gamma-synuclein;SNCG;ortholog	GAMMA-SYNUCLEIN (PTHR13820:SF14)
HUMAN|HGNC=19022|UniProtKB=O43301	HUMAN|HGNC=19022|UniProtKB=O43301	Heat shock 70 kDa protein 12A;HSPA12A;ortholog	HEAT SHOCK 70 KDA PROTEIN 12A (PTHR14187:SF63)
HUMAN|HGNC=5246|UniProtKB=P04792	HUMAN|HGNC=5246|UniProtKB=P04792	Heat shock protein beta-1;HSPB1;ortholog	HEAT SHOCK PROTEIN BETA-1 (PTHR11527:SF235)
HUMAN|HGNC=5253|UniProtKB=P07900	HUMAN|HGNC=5253|UniProtKB=P07900	Heat shock protein HSP 90-alpha;HSP90AA1;ortholog	HEAT SHOCK PROTEIN HSP 90-ALPHA-RELATED (PTHR11528:SF63)
HUMAN|HGNC=5258|UniProtKB=P08238	HUMAN|HGNC=5258|UniProtKB=P08238	Heat shock protein HSP 90-beta;HSP90AB1;ortholog	HEAT SHOCK PROTEIN HSP 90-BETA-RELATED (PTHR11528:SF67)
HUMAN|HGNC=11985|UniProtKB=O94826	HUMAN|HGNC=11985|UniProtKB=O94826	Mitochondrial import receptor subunit TOM70;TOMM70A;ortholog	MITOCHONDRIAL IMPORT RECEPTOR SUBUNIT TOM70 (PTHR22904:SF476)
HUMAN|HGNC=3719|UniProtKB=Q00688		Peptidyl-prolyl cis-trans isomerase FKBP3;FKBP3;ortholog	PEPTIDYL-PROLYL CIS-TRANS ISOMERASE FKBP3 (PTHR10516:SF356)
HUMAN|HGNC=3720|UniProtKB=Q02790		Peptidyl-prolyl cis-trans isomerase FKBP4;FKBP4;ortholog	PEPTIDYL-PROLYL CIS-TRANS ISOMERASE FKBP4 (PTHR10516:SF332)
HUMAN|HGNC=3724|UniProtKB=Q14318		Peptidyl-prolyl cis-trans isomerase FKBP8;FKBP8;ortholog	PEPTIDYL-PROLYL CIS-TRANS ISOMERASE FKBP8 (PTHR10516:SF363)
HUMAN|HGNC=10819|UniProtKB=O43765		Small glutamine-rich tetratricopeptide repeat-containing protein alpha;SGTA;ortholog	SMALL GLUTAMINE-RICH TETRATRICOPEPTIDE REPEAT-CONTAINING PROTEIN ALPHA (PTHR22904:SF475)
HUMAN|HGNC=11655|UniProtKB=P17987	HUMAN|HGNC=11655|UniProtKB=P17987	T-complex protein 1 subunit alpha;TCP1;ortholog	T-COMPLEX PROTEIN 1 SUBUNIT ALPHA (PTHR11353:SF145)

HUMAN|HGNC=1615|UniProtKB=P78371	HUMAN|HGNC=1615|UniProtKB=P78371	T-complex protein 1 subunit beta;CCT2;ortholog	T-COMPLEX PROTEIN 1 SUBUNIT BETA (PTHR11353:SF119)
HUMAN|HGNC=1617|UniProtKB=P50991	HUMAN|HGNC=1617|UniProtKB=P50991	T-complex protein 1 subunit delta;CCT4;ortholog	T-COMPLEX PROTEIN 1 SUBUNIT DELTA (PTHR11353:SF146)
HUMAN|HGNC=1618|UniProtKB=P48643		T-complex protein 1 subunit epsilon;CCT5;ortholog	T-COMPLEX PROTEIN 1 SUBUNIT EPSILON (PTHR11353:SF156)
HUMAN|HGNC=1623|UniProtKB=P50990	HUMAN|HGNC=1623|UniProtKB=P50990	T-complex protein 1 subunit theta;CCT8;ortholog	T-COMPLEX PROTEIN 1 SUBUNIT THETA (PTHR11353:SF133)
HUMAN|HGNC=1620|UniProtKB=P40227	HUMAN|HGNC=1620|UniProtKB=P40227	T-complex protein 1 subunit zeta;CCT6A;ortholog	T-COMPLEX PROTEIN 1 SUBUNIT ZETA (PTHR11353:SF140)
	HUMAN|HGNC=1989|UniProtKB=Q99426	Tubulin-folding cofactor B;TBCB;ortholog	TUBULIN-FOLDING COFACTOR B (PTHR18916:SF53)
	HUMAN|HGNC=11581|UniProtKB=Q9BTW9	Tubulin-specific chaperone D;TBCD;ortholog	TUBULIN-SPECIFIC CHAPERONE D (PTHR12658:SF3)
**cytoskeletal protein (PC00085)**			
HUMAN|HGNC=703|UniProtKB=Q92747		Actin-related protein 2/3 complex subunit 1A;ARPC1A;ortholog	ACTIN-RELATED PROTEIN 2/3 COMPLEX SUBUNIT 1A (PTHR10709:SF21)
	HUMAN|HGNC=706|UniProtKB=O15145	Actin-related protein 2/3 complex subunit 3;ARPC3;ortholog	ACTIN-RELATED PROTEIN 2/3 COMPLEX SUBUNIT 3 (PTHR12391:SF1)
	HUMAN|HGNC=170|UniProtKB=P61158	Actin-related protein 3;ACTR3;ortholog	ACTIN-RELATED PROTEIN 3-RELATED (PTHR11937:SF284)
	HUMAN|HGNC=143|UniProtKB=P68032	Actin, alpha cardiac muscle 1;ACTC1;ortholog	ACTIN, ALPHA CARDIAC MUSCLE 1 (PTHR11937:SF335)
HUMAN|HGNC=132|UniProtKB=P60709	HUMAN|HGNC=132|UniProtKB=P60709	Actin, cytoplasmic 1;ACTB;ortholog	ACTIN, CYTOPLASMIC 1 (PTHR11937:SF288)
HUMAN|HGNC=145|UniProtKB=P63267		Actin, gamma-enteric smooth muscle;ACTG2;ortholog	ACTIN, GAMMA-ENTERIC SMOOTH MUSCLE (PTHR11937:SF278)
HUMAN|HGNC=164|UniProtKB=P35609		Alpha-actinin-2;ACTN2;ortholog	ALPHA-ACTININ-2 (PTHR11915:SF393)
HUMAN|HGNC=166|UniProtKB=O43707		Alpha-actinin-4;ACTN4;ortholog	ALPHA-ACTININ-4 (PTHR11915:SF408)
HUMAN|HGNC=167|UniProtKB=P61163		Alpha-centractin;ACTR1A;ortholog	ALPHA-CENTRACTIN (PTHR11937:SF271)
HUMAN|HGNC=6057|UniProtKB=Q16352	HUMAN|HGNC=6057|UniProtKB=Q16352	Alpha-internexin;INA;ortholog	ALPHA-INTERNEXIN (PTHR23239:SF273)
HUMAN|HGNC=493|UniProtKB=Q01484	HUMAN|HGNC=493|UniProtKB=Q01484	Ankyrin-2;ANK2;ortholog	ANKYRIN-2 (PTHR24123:SF39)
HUMAN|HGNC=168|UniProtKB=P42025		Beta-centractin;ACTR1B;ortholog	BETA-CENTRACTIN (PTHR11937:SF273)
	HUMAN|HGNC=2586|UniProtKB=Q9UDT6	CAP-Gly domain-containing linker protein 2;CLIP2;ortholog	CAP-GLY DOMAIN-CONTAINING LINKER PROTEIN 2 (PTHR18916:SF45)
HUMAN|HGNC=13518|UniProtKB=Q9Y696		Chloride intracellular channel protein 4;CLIC4;ortholog	CHLORIDE INTRACELLULAR CHANNEL PROTEIN 4 (PTHR11260:SF443)
	HUMAN|HGNC=2252|UniProtKB=P31146	Coronin-1A;CORO1A;ortholog	CORONIN-1A (PTHR10856:SF31)
HUMAN|HGNC=2961|UniProtKB=Q14204	HUMAN|HGNC=2961|UniProtKB=Q14204	Cytoplasmic dynein 1 heavy chain 1;DYNC1H1;ortholog	CYTOPLASMIC DYNEIN 1 HEAVY CHAIN 1 (PTHR10676:SF302)
	HUMAN|HGNC=2966|UniProtKB=O43237	Cytoplasmic dynein 1 light intermediate chain 2;DYNC1LI2;ortholog	CYTOPLASMIC DYNEIN 1 LIGHT INTERMEDIATE CHAIN 2 (PTHR12688:SF3)
HUMAN|HGNC=2770|UniProtKB=P17661	HUMAN|HGNC=2770|UniProtKB=P17661	Desmin;DES;ortholog	DESMIN (PTHR23239:SF307)
HUMAN|HGNC=2712|UniProtKB=Q13561	HUMAN|HGNC=2712|UniProtKB=Q13561	Dynactin subunit 2;DCTN2;ortholog	DYNACTIN SUBUNIT 2 (PTHR15346:SF1)
HUMAN|HGNC=8140|UniProtKB=O60313	HUMAN|HGNC=8140|UniProtKB=O60313	Dynamin-like 120 kDa protein, mitochondrial;OPA1;ortholog	DYNAMIN-LIKE 120 KDA PROTEIN, MITOCHONDRIAL (PTHR11566:SF100)
HUMAN|HGNC=15476|UniProtKB=P63167	HUMAN|HGNC=15476|UniProtKB=P63167	Dynein light chain 1, cytoplasmic;DYNLL1;ortholog	DYNEIN LIGHT CHAIN 1, CYTOPLASMIC (PTHR11886:SF60)
HUMAN|HGNC=15468|UniProtKB=Q9NP97		Dynein light chain roadblock-type 1;DYNLRB1;ortholog	DYNEIN LIGHT CHAIN ROADBLOCK-TYPE 1 (PTHR10779:SF21)
HUMAN|HGNC=3213|UniProtKB=P26641	HUMAN|HGNC=3213|UniProtKB=P26641	Elongation factor 1-gamma;EEF1G;ortholog	ELONGATION FACTOR 1-GAMMA (PTHR11260:SF420)

HUMAN|HGNC=3383|UniProtKB=P27105	HUMAN|HGNC=3383|UniProtKB=P27105	Erythrocyte band 7 integral membrane protein;STOM;ortholog	ERYTHROCYTE BAND 7 INTEGRAL MEMBRANE PROTEIN (PTHR10264:SF109)
HUMAN|HGNC=1488|UniProtKB=P52907		F-actin-capping protein subunit alpha-1;CAPZA1;ortholog	F-ACTIN-CAPPING PROTEIN SUBUNIT ALPHA-1 (PTHR10653:SF9)
	HUMAN|HGNC=1490|UniProtKB=P47755	F-actin-capping protein subunit alpha-2;CAPZA2;ortholog	F-ACTIN-CAPPING PROTEIN SUBUNIT ALPHA-2 (PTHR10653:SF11)
HUMAN|HGNC=11148|UniProtKB=Q16658	HUMAN|HGNC=11148|UniProtKB=Q16658	Fascin;FSCN1;ortholog	FASCIN (PTHR10551:SF19)
HUMAN|HGNC=18267|UniProtKB=Q96PY5		Formin-like protein 2;FMNL2;ortholog	FORMIN-LIKE PROTEIN 2 (PTHR23213:SF282)
HUMAN|HGNC=11141|UniProtKB=O76070		Gamma-synuclein;SNCG;ortholog	GAMMA-SYNUCLEIN (PTHR13820:SF14)
HUMAN|HGNC=15968|UniProtKB=Q8TB36		Ganglioside-induced differentiation-associated protein 1;GDAP1;ortholog	GANGLIOSIDE-INDUCED DIFFERENTIATION-ASSOCIATED PROTEIN 1 (PTHR11260:SF391)
HUMAN|HGNC=4235|UniProtKB=P14136	HUMAN|HGNC=4235|UniProtKB=P14136	Glial fibrillary acidic protein;GFAP;ortholog	GLIAL FIBRILLARY ACIDIC PROTEIN (PTHR23239:SF249)
HUMAN|HGNC=6413|UniProtKB=P13645	HUMAN|HGNC=6413|UniProtKB=P13645	Keratin, type I cytoskeletal 10;KRT10;ortholog	KERATIN, TYPE I CYTOSKELETAL 10 (PTHR23239:SF308)
	HUMAN|HGNC=6416|UniProtKB=P02533	Keratin, type I cytoskeletal 14;KRT14;ortholog	KERATIN, TYPE I CYTOSKELETAL 14 (PTHR23239:SF272)
	HUMAN|HGNC=6421|UniProtKB=P19012	Keratin, type I cytoskeletal 15;KRT15;ortholog	KERATIN, TYPE I CYTOSKELETAL 15 (PTHR23239:SF239)
	HUMAN|HGNC=6423|UniProtKB=P08779	Keratin, type I cytoskeletal 16;KRT16;ortholog	KERATIN, TYPE I CYTOSKELETAL 16 (PTHR23239:SF263)
HUMAN|HGNC=6447|UniProtKB=P35527	HUMAN|HGNC=6447|UniProtKB=P35527	Keratin, type I cytoskeletal 9;KRT9;ortholog	KERATIN, TYPE I CYTOSKELETAL 9 (PTHR23239:SF281)
HUMAN|HGNC=6412|UniProtKB=P04264	HUMAN|HGNC=6412|UniProtKB=P04264	Keratin, type II cytoskeletal 1;KRT1;ortholog	KERATIN, TYPE II CYTOSKELETAL 1 (PTHR23239:SF236)
HUMAN|HGNC=6439|UniProtKB=P35908	HUMAN|HGNC=6439|UniProtKB=P35908	Keratin, type II cytoskeletal 2 epidermal;KRT2;ortholog	KERATIN, TYPE II CYTOSKELETAL 2 EPIDERMAL (PTHR23239:SF228)
	HUMAN|HGNC=6440|UniProtKB=P12035	Keratin, type II cytoskeletal 3;KRT3;ortholog	KERATIN, TYPE II CYTOSKELETAL 3 (PTHR23239:SF231)
	HUMAN|HGNC=6442|UniProtKB=P13647	Keratin, type II cytoskeletal 5;KRT5;ortholog	KERATIN, TYPE II CYTOSKELETAL 5 (PTHR23239:SF279)
	HUMAN|HGNC=6443|UniProtKB=P02538	Keratin, type II cytoskeletal 6A;KRT6A;ortholog	KERATIN, TYPE II CYTOSKELETAL 6A (PTHR23239:SF232)
	HUMAN|HGNC=28929|UniProtKB=Q7RTS7	Keratin, type II cytoskeletal 74;KRT74;ortholog	KERATIN, TYPE II CYTOSKELETAL 74 (PTHR23239:SF328)
	HUMAN|HGNC=6325|UniProtKB=O60282	Kinesin heavy chain isoform 5C;KIF5C;ortholog	KINESIN HEAVY CHAIN ISOFORM 5C (PTHR24115:SF633)
	HUMAN|HGNC=6324|UniProtKB=P33176	Kinesin-1 heavy chain;KIF5B;ortholog	KINESIN-1 HEAVY CHAIN (PTHR24115:SF708)
	HUMAN|HGNC=888|UniProtKB=Q12756	Kinesin-like protein KIF1A;KIF1A;ortholog	KINESIN-LIKE PROTEIN KIF1A (PTHR24115:SF616)
HUMAN|HGNC=6637|UniProtKB=P20700		Lamin-B1;LMNB1;ortholog	LAMIN-B1 (PTHR23239:SF316)
HUMAN|HGNC=6638|UniProtKB=Q03252	HUMAN|HGNC=6638|UniProtKB=Q03252	Lamin-B2;LMNB2;ortholog	LAMIN-B2 (PTHR23239:SF280)
HUMAN|HGNC=6835|UniProtKB=P78559	HUMAN|HGNC=6835|UniProtKB=P78559	Microtubule-associated protein 1A;MAP1A;ortholog	MICROTUBULE-ASSOCIATED PROTEIN 1A (PTHR13843:SF13)
HUMAN|HGNC=6836|UniProtKB=P46821	HUMAN|HGNC=6836|UniProtKB=P46821	Microtubule-associated protein 1B;MAP1B;ortholog	MICROTUBULE-ASSOCIATED PROTEIN 1B (PTHR13843:SF15)
HUMAN|HGNC=6892|UniProtKB=Q9UPY8	HUMAN|HGNC=6892|UniProtKB=Q9UPY8	Microtubule-associated protein RP/EB family member 3;MAPRE3;ortholog	MICROTUBULE-ASSOCIATED PROTEIN RP/EB FAMILY MEMBER 3 (PTHR10623:SF23)
HUMAN|HGNC=7373|UniProtKB=P26038		Moesin;MSN;ortholog	MOESIN (PTHR23281:SF30)
HUMAN|HGNC=7568|UniProtKB=P35580	HUMAN|HGNC=7568|UniProtKB=P35580	Myosin-10;MYH10;ortholog	MYOSIN-10 (PTHR13140:SF549)
HUMAN|HGNC=7739|UniProtKB=P07196	HUMAN|HGNC=7739|UniProtKB=P07196	Neurofilament light polypeptide;NEFL;ortholog	NEUROFILAMENT LIGHT POLYPEPTIDE (PTHR23239:SF242)
HUMAN|HGNC=9461|UniProtKB=P41219		Peripherin;PRPH;ortholog	PERIPHERIN (PTHR23239:SF288)

HUMAN|HGNC=9944|UniProtKB=P35241		Radixin;RDX;ortholog	RADIXIN (PTHR23281:SF31)
HUMAN|HGNC=1717|UniProtKB=Q16181		Septin-7;SEPT7;ortholog	SEPTIN-7 (PTHR18884:SF86)
HUMAN|HGNC=11273|UniProtKB=Q13813	HUMAN|HGNC=11273|UniProtKB=Q13813	Spectrin alpha chain, non-erythrocytic 1;SPTAN1;ortholog	SPECTRIN ALPHA CHAIN, NON-ERYTHROCYTIC 1 (PTHR11915:SF384)
HUMAN|HGNC=11275|UniProtKB=Q01082	HUMAN|HGNC=11275|UniProtKB=Q01082	Spectrin beta chain, non-erythrocytic 1;SPTBN1;ortholog	SPECTRIN BETA CHAIN, NON-ERYTHROCYTIC 1 (PTHR11915:SF343)
HUMAN|HGNC=11496|UniProtKB=O14994	HUMAN|HGNC=11496|UniProtKB=O14994	Synapsin-3;SYN3;ortholog	SYNAPSIN-3 (PTHR10841:SF25)
	HUMAN|HGNC=11845|UniProtKB=Q9Y490	Talin-1;TLN1;ortholog	TALIN-1 (PTHR19981:SF24)
	HUMAN|HGNC=15447|UniProtKB=Q9Y4G6	Talin-2;TLN2;ortholog	TALIN-2 (PTHR19981:SF28)
	HUMAN|HGNC=12410|UniProtKB=Q9NY65	Tubulin alpha-8 chain;TUBA8;ortholog	TUBULIN ALPHA-8 CHAIN (PTHR11588:SF181)
HUMAN|HGNC=20778|UniProtKB=P07437	HUMAN|HGNC=20778|UniProtKB=P07437	Tubulin beta chain;TUBB;ortholog	TUBULIN BETA CHAIN (PTHR11588:SF211)
HUMAN|HGNC=16257|UniProtKB=Q9H4B7	HUMAN|HGNC=16257|UniProtKB=Q9H4B7	Tubulin beta-1 chain;TUBB1;ortholog	TUBULIN BETA-1 CHAIN (PTHR11588:SF185)
HUMAN|HGNC=12412|UniProtKB=Q13885	HUMAN|HGNC=12412|UniProtKB=Q13885	Tubulin beta-2A chain;TUBB2A;ortholog	TUBULIN BETA-2A CHAIN (PTHR11588:SF203)
HUMAN|HGNC=30829|UniProtKB=Q9BVA1	HUMAN|HGNC=30829|UniProtKB=Q9BVA1	Tubulin beta-2B chain;TUBB2B;ortholog	TUBULIN BETA-2B CHAIN (PTHR11588:SF153)
HUMAN|HGNC=20772|UniProtKB=Q13509	HUMAN|HGNC=20772|UniProtKB=Q13509	Tubulin beta-3 chain;TUBB3;ortholog	TUBULIN BETA-3 CHAIN (PTHR11588:SF174)
HUMAN|HGNC=20774|UniProtKB=P04350	HUMAN|HGNC=20774|UniProtKB=P04350	Tubulin beta-4A chain;TUBB4A;ortholog	TUBULIN BETA-4A CHAIN (PTHR11588:SF198)
HUMAN|HGNC=20771|UniProtKB=P68371		Tubulin beta-4B chain;TUBB4B;ortholog	TUBULIN BETA-4B CHAIN (PTHR11588:SF169)
HUMAN|HGNC=20776|UniProtKB=Q9BUF5	HUMAN|HGNC=20776|UniProtKB=Q9BUF5	Tubulin beta-6 chain;TUBB6;ortholog	TUBULIN BETA-6 CHAIN (PTHR11588:SF212)
HUMAN|HGNC=24164|UniProtKB=O94811	HUMAN|HGNC=24164|UniProtKB=O94811	Tubulin polymerization-promoting protein;TPPP;ortholog	TUBULIN POLYMERIZATION-PROMOTING PROTEIN (PTHR12932:SF20)
	HUMAN|HGNC=1989|UniProtKB=Q99426	Tubulin-folding cofactor B;TBCB;ortholog	TUBULIN-FOLDING COFACTOR B (PTHR18916:SF53)
	HUMAN|HGNC=11581|UniProtKB=Q9BTW9	Tubulin-specific chaperone D;TBCD;ortholog	TUBULIN-SPECIFIC CHAPERONE D (PTHR12658:SF3)
HUMAN|Ensembl=ENSG00000260914|UniProtKB=I3L4J1		Uncharacterized protein (Fragment);unassigned;ortholog	VACUOLAR PROTEIN SORTING-ASSOCIATED PROTEIN 4A (PTHR23074:SF116)
	HUMAN|HGNC=12692|UniProtKB=P08670	Vimentin;VIM;ortholog	VIMENTIN (PTHR23239:SF258)
HUMAN|HGNC=12754|UniProtKB=O75083	HUMAN|HGNC=12754|UniProtKB=O75083	WD repeat-containing protein 1;WDR1;ortholog	WD REPEAT-CONTAINING PROTEIN 1 (PTHR19856:SF2)
**defense/immunity protein (PC00090)**			
HUMAN|HGNC=18007|UniProtKB=Q9H4G4		Golgi-associated plant pathogenesis-related protein 1;GLIPR2;ortholog	GOLGI-ASSOCIATED PLANT PATHOGENESIS-RELATED PROTEIN 1 (PTHR10334:SF257)
HUMAN|HGNC=26361|UniProtKB=Q14CZ8		Hepatocyte cell adhesion molecule;HEPACAM;ortholog	HEPATOCYTE CELL ADHESION MOLECULE (PTHR12080:SF66)
	HUMAN|HGNC=4962|UniProtKB=P13747	HLA class I histocompatibility antigen, alpha chain E;HLA-E;ortholog	HLA CLASS I HISTOCOMPATIBILITY ANTIGEN, ALPHA CHAIN E (PTHR16675:SF211)
HUMAN|HGNC=28246|UniProtKB=Q96ID5		Immunoglobulin superfamily member 21;IGSF21;ortholog	IMMUNOGLOBULIN SUPERFAMILY MEMBER 21 (PTHR10489:SF771)
HUMAN|HGNC=7656|UniProtKB=P13591		Neural cell adhesion molecule 1;NCAM1;ortholog	NEURAL CELL ADHESION MOLECULE 1 (PTHR10489:SF783)
**enzyme modulator (PC00095)**			
HUMAN|HGNC=9557|UniProtKB=O00232	HUMAN|HGNC=9557|UniProtKB=O00232	26S proteasome non-ATPase regulatory subunit 12;PSMD12;ortholog	26S PROTEASOME NON-ATPASE REGULATORY SUBUNIT 12 (PTHR10855:SF4)

	HUMAN|HGNC=9558|UniProtKB=Q9UNM6	26S proteasome non-ATPase regulatory subunit 13;PSMD13;ortholog	26S PROTEASOME NON-ATPASE REGULATORY SUBUNIT 13 (PTHR10539:SF1)
HUMAN|HGNC=9559|UniProtKB=Q13200		26S proteasome non-ATPase regulatory subunit 2;PSMD2;ortholog	26S PROTEASOME NON-ATPASE REGULATORY SUBUNIT 2 (PTHR10943:SF7)
	HUMAN|HGNC=1189|UniProtKB=O95433	Activator of 90 kDa heat shock protein ATPase homolog 1;AHSA1;ortholog	ACTIVATOR OF 90 KDA HEAT SHOCK PROTEIN ATPASE HOMOLOG 1 (PTHR13009:SF14)
HUMAN|HGNC=2690|UniProtKB=P07108		Acyl-CoA-binding protein;DBI;ortholog	ACYL-COA-BINDING PROTEIN (PTHR23310:SF74)
	HUMAN|HGNC=654|UniProtKB=P61204	ADP-ribosylation factor 3;ARF3;ortholog	ADP-RIBOSYLATION FACTOR 3 (PTHR11711:SF288)
	HUMAN|HGNC=693|UniProtKB=P36404	ADP-ribosylation factor-like protein 2;ARL2;ortholog	ADP-RIBOSYLATION FACTOR-LIKE PROTEIN 2-RELATED (PTHR11711:SF230)
	HUMAN|HGNC=25192|UniProtKB=Q96BM9	ADP-ribosylation factor-like protein 8A;ARL8A;ortholog	ADP-RIBOSYLATION FACTOR-LIKE PROTEIN 8A (PTHR11711:SF282)
	HUMAN|HGNC=25564|UniProtKB=Q9NVJ2	ADP-ribosylation factor-like protein 8B;ARL8B;ortholog	ADP-RIBOSYLATION FACTOR-LIKE PROTEIN 8B (PTHR11711:SF219)
	HUMAN|HGNC=14678|UniProtKB=Q96CX2	BTB/POZ domain-containing protein KCTD12;KCTD12;ortholog	BTB/POZ DOMAIN-CONTAINING PROTEIN KCTD12 (PTHR14499:SF103)
HUMAN|HGNC=29244|UniProtKB=Q68DU8		BTB/POZ domain-containing protein KCTD16;KCTD16;ortholog	BTB/POZ DOMAIN-CONTAINING PROTEIN KCTD16 (PTHR14499:SF81)
HUMAN|HGNC=2460|UniProtKB=P67870		Casein kinase II subunit beta;CSNK2B;ortholog	CASEIN KINASE II SUBUNIT BETA (PTHR11740:SF12)
HUMAN|HGNC=1736|UniProtKB=P60953	HUMAN|HGNC=1736|UniProtKB=P60953	Cell division control protein 42 homolog;CDC42;ortholog	CELL DIVISION CONTROL PROTEIN 42 HOMOLOG (PTHR24072:SF239)
HUMAN|HGNC=8011|UniProtKB=P78357	HUMAN|HGNC=8011|UniProtKB=P78357	Contactin-associated protein 1;CNTNAP1;ortholog	CONTACTIN-ASSOCIATED PROTEIN 1 (PTHR10127:SF740)
	HUMAN|HGNC=2966|UniProtKB=O43237	Cytoplasmic dynein 1 light intermediate chain 2;DYNC1LI2;ortholog	CYTOPLASMIC DYNEIN 1 LIGHT INTERMEDIATE CHAIN 2 (PTHR12688:SF3)
HUMAN|HGNC=13759|UniProtKB=Q7L576		Cytoplasmic FMR1-interacting protein 1;CYFIP1;ortholog	CYTOPLASMIC FMR1-INTERACTING PROTEIN 1 (PTHR12195:SF4)
HUMAN|HGNC=8140|UniProtKB=O60313	HUMAN|HGNC=8140|UniProtKB=O60313	Dynamin-like 120 kDa protein, mitochondrial;OPA1;ortholog	DYNAMIN-LIKE 120 KDA PROTEIN, MITOCHONDRIAL (PTHR11566:SF100)
HUMAN|HGNC=15476|UniProtKB=P63167	HUMAN|HGNC=15476|UniProtKB=P63167	Dynein light chain 1, cytoplasmic;DYNLL1;ortholog	DYNEIN LIGHT CHAIN 1, CYTOPLASMIC (PTHR11886:SF60)
	HUMAN|HGNC=3243|UniProtKB=Q9NZN4	EH domain-containing protein 2;EHD2;ortholog	EH DOMAIN-CONTAINING PROTEIN 2 (PTHR11216:SF106)
HUMAN|HGNC=3244|UniProtKB=Q9NZN3	HUMAN|HGNC=3244|UniProtKB=Q9NZN3	EH domain-containing protein 3;EHD3;ortholog	EH DOMAIN-CONTAINING PROTEIN 3 (PTHR11216:SF88)
HUMAN|HGNC=3189|UniProtKB=P68104	HUMAN|HGNC=3189|UniProtKB=P68104	Elongation factor 1-alpha 1;EEF1A1;ortholog	ELONGATION FACTOR 1-ALPHA 1 (PTHR23115:SF216)
HUMAN|HGNC=3192|UniProtKB=Q05639	HUMAN|HGNC=3192|UniProtKB=Q05639	Elongation factor 1-alpha 2;EEF1A2;ortholog	ELONGATION FACTOR 1-ALPHA 2 (PTHR23115:SF203)
HUMAN|HGNC=3214|UniProtKB=P13639	HUMAN|HGNC=3214|UniProtKB=P13639	Elongation factor 2;EEF2;ortholog	ELONGATION FACTOR 2 (PTHR42908:SF8)
HUMAN|HGNC=12420|UniProtKB=P49411	HUMAN|HGNC=12420|UniProtKB=P49411	Elongation factor Tu, mitochondrial;TUFM;ortholog	ELONGATION FACTOR TU, MITOCHONDRIAL (PTHR43721:SF7)
HUMAN|HGNC=10831|UniProtKB=Q99962		Endophilin-A1;SH3GL2;ortholog	ENDOPHILIN-A1 (PTHR10663:SF240)
	HUMAN|HGNC=3267|UniProtKB=P41091	Eukaryotic translation initiation factor 2 subunit 3;EIF2S3;ortholog	EUKARYOTIC TRANSLATION INITIATION FACTOR 2 SUBUNIT 3 (PTHR42854:SF1)
HUMAN|HGNC=3299|UniProtKB=P55010		Eukaryotic translation initiation factor 5;EIF5;ortholog	EUKARYOTIC TRANSLATION INITIATION FACTOR 5 (PTHR23001:SF11)
	HUMAN|HGNC=3591|UniProtKB=Q9Y4F1	FERM, RhoGEF and pleckstrin domain-containing protein 1;FARP1;ortholog	FERM, RHOGEF AND PLECKSTRIN DOMAIN-CONTAINING PROTEIN 1 (PTHR12673:SF153)
HUMAN|HGNC=9846|UniProtKB=P62826	HUMAN|HGNC=9846|UniProtKB=P62826	GTP-binding nuclear protein Ran;RAN;ortholog	GTP-BINDING NUCLEAR PROTEIN RAN (PTHR24071:SF14)
	HUMAN|HGNC=19323|UniProtKB=Q96HU8	GTP-binding protein Di-Ras2;DIRAS2;ortholog	GTP-BINDING PROTEIN DI-RAS2 (PTHR24070:SF281)

	HUMAN|HGNC=10534|UniProtKB=Q9NR31	GTP-binding protein SAR1a;SAR1A;ortholog	GTP-BINDING PROTEIN SAR1A (PTHR11711:SF256)
HUMAN|HGNC=7989|UniProtKB=P01111	HUMAN|HGNC=7989|UniProtKB=P01111	GTPase NRas;NRAS;ortholog	GTPASE NRAS (PTHR24070:SF354)
HUMAN|HGNC=4384|UniProtKB=P63096	HUMAN|HGNC=4384|UniProtKB=P63096	Guanine nucleotide-binding protein G(i) subunit alpha-1;GNAI1;ortholog	GUANINE NUCLEOTIDE-BINDING PROTEIN G(I) SUBUNIT ALPHA-1 (PTHR10218:SF283)
HUMAN|HGNC=4385|UniProtKB=P04899	HUMAN|HGNC=4385|UniProtKB=P04899	Guanine nucleotide-binding protein G(i) subunit alpha-2;GNAI2;ortholog	GUANINE NUCLEOTIDE-BINDING PROTEIN G(I) SUBUNIT ALPHA-2 (PTHR10218:SF279)
HUMAN|HGNC=4398|UniProtKB=P62879	HUMAN|HGNC=4398|UniProtKB=P62879	Guanine nucleotide-binding protein G(I)/G(S)/G(T) subunit beta-2;GNB2;ortholog	GUANINE NUCLEOTIDE-BINDING PROTEIN G(I)/G(S)/G(T) SUBUNIT BETA-2 (PTHR19850:SF39)
HUMAN|HGNC=4387|UniProtKB=P08754		Guanine nucleotide-binding protein G(k) subunit alpha;GNAI3;ortholog	GUANINE NUCLEOTIDE-BINDING PROTEIN G(K) SUBUNIT ALPHA (PTHR10218:SF289)
HUMAN|HGNC=4389|UniProtKB=P09471	HUMAN|HGNC=4389|UniProtKB=P09471	Guanine nucleotide-binding protein G(o) subunit alpha;GNAO1;ortholog	GUANINE NUCLEOTIDE-BINDING PROTEIN G(O) SUBUNIT ALPHA (PTHR10218:SF248)
HUMAN|HGNC=4390|UniProtKB=P50148	HUMAN|HGNC=4390|UniProtKB=P50148	Guanine nucleotide-binding protein G(q) subunit alpha;GNAQ;ortholog	GUANINE NUCLEOTIDE-BINDING PROTEIN G(Q) SUBUNIT ALPHA (PTHR10218:SF255)
HUMAN|HGNC=4395|UniProtKB=P19086	HUMAN|HGNC=4395|UniProtKB=P19086	Guanine nucleotide-binding protein G(z) subunit alpha;GNAZ;ortholog	GUANINE NUCLEOTIDE-BINDING PROTEIN G(Z) SUBUNIT ALPHA (PTHR10218:SF268)
HUMAN|HGNC=4379|UniProtKB=P29992	HUMAN|HGNC=4379|UniProtKB=P29992	Guanine nucleotide-binding protein subunit alpha-11;GNA11;ortholog	GUANINE NUCLEOTIDE-BINDING PROTEIN SUBUNIT ALPHA-11 (PTHR10218:SF262)
HUMAN|HGNC=4381|UniProtKB=Q14344	HUMAN|HGNC=4381|UniProtKB=Q14344	Guanine nucleotide-binding protein subunit alpha-13;GNA13;ortholog	GUANINE NUCLEOTIDE-BINDING PROTEIN SUBUNIT ALPHA-13 (PTHR10218:SF271)
HUMAN|HGNC=20731|UniProtKB=Q9HAV0		Guanine nucleotide-binding protein subunit beta-4;GNB4;ortholog	GUANINE NUCLEOTIDE-BINDING PROTEIN SUBUNIT BETA-4 (PTHR19850:SF42)
HUMAN|HGNC=4413|UniProtKB=P36915		Guanine nucleotide-binding protein-like 1;GNL1;ortholog	GUANINE NUCLEOTIDE-BINDING PROTEIN-LIKE 1 (PTHR11089:SF55)
	HUMAN|HGNC=29112|UniProtKB=Q6DN90	IQ motif and SEC7 domain-containing protein 1;IQSEC1;ortholog	IQ MOTIF AND SEC7 DOMAIN-CONTAINING PROTEIN 1 (PTHR10663:SF219)
HUMAN|HGNC=7568|UniProtKB=P35580	HUMAN|HGNC=7568|UniProtKB=P35580	Myosin-10;MYH10;ortholog	MYOSIN-10 (PTHR13140:SF549)
HUMAN|HGNC=8574|UniProtKB=P43034		Platelet-activating factor acetylhydrolase IB subunit alpha;PAFAH1B1;ortholog	PLATELET-ACTIVATING FACTOR ACETYLHYDROLASE IB SUBUNIT ALPHA (PTHR22847:SF549)
HUMAN|HGNC=8647|UniProtKB=Q15365	HUMAN|HGNC=8647|UniProtKB=Q15365	Poly(rC)-binding protein 1;PCBP1;ortholog	POLY(RC)-BINDING PROTEIN 1 (PTHR10288:SF191)
HUMAN|HGNC=29180|UniProtKB=Q9UPV7	HUMAN|HGNC=29180|UniProtKB=Q9UPV7	Protein KIAA1045;KIAA1045;ortholog	PROTEIN KIAA1045 (PTHR10891:SF706)
HUMAN|HGNC=4226|UniProtKB=P31150	HUMAN|HGNC=4226|UniProtKB=P31150	Rab GDP dissociation inhibitor alpha;GDI1;ortholog	RAB GDP DISSOCIATION INHIBITOR ALPHA (PTHR11787:SF19)
HUMAN|HGNC=4227|UniProtKB=P50395		Rab GDP dissociation inhibitor beta;GDI2;ortholog	RAB GDP DISSOCIATION INHIBITOR BETA (PTHR11787:SF21)
	HUMAN|HGNC=17168|UniProtKB=Q9H2M9	Rab3 GTPase-activating protein non-catalytic subunit;RAB3GAP2;ortholog	RAB3 GTPASE-ACTIVATING PROTEIN NON-CATALYTIC SUBUNIT (PTHR12472:SF1)
	HUMAN|HGNC=16854|UniProtKB=Q9Y4G8	Rap guanine nucleotide exchange factor 2;RAPGEF2;ortholog	RAP GUANINE NUCLEOTIDE EXCHANGE FACTOR 2 (PTHR23113:SF242)
HUMAN|HGNC=9801|UniProtKB=P63000	HUMAN|HGNC=9801|UniProtKB=P63000	Ras-related C3 botulinum toxin substrate 1;RAC1;ortholog	RAS-RELATED C3 BOTULINUM TOXIN SUBSTRATE 1 (PTHR24072:SF194)
HUMAN|HGNC=9803|UniProtKB=P60763		Ras-related C3 botulinum toxin substrate 3;RAC3;ortholog	RAS-RELATED C3 BOTULINUM TOXIN SUBSTRATE 3 (PTHR24072:SF220)
HUMAN|HGNC=16963|UniProtKB=Q7L523		Ras-related GTP-binding protein A;RRAGA;ortholog	RAS-RELATED GTP-BINDING PROTEIN A (PTHR11259:SF12)
HUMAN|HGNC=9839|UniProtKB=P11233	HUMAN|HGNC=9839|UniProtKB=P11233	Ras-related protein Ral-A;RALA;ortholog	RAS-RELATED PROTEIN RAL-A (PTHR24070:SF277)
HUMAN|HGNC=9840|UniProtKB=P11234		Ras-related protein Ral-B;RALB;ortholog	RAS-RELATED PROTEIN RAL-B (PTHR24070:SF348)
	HUMAN|HGNC=9855|UniProtKB=P62834	Ras-related protein Rap-1A;RAP1A;ortholog	RAS-RELATED PROTEIN RAP-1A (PTHR24070:SF363)

HUMAN|HGNC=9861|UniProtKB=P10114	HUMAN|HGNC=9861|UniProtKB=P10114	Ras-related protein Rap-2a;RAP2A;ortholog	RAS-RELATED PROTEIN RAP-2A (PTHR24070:SF364)
HUMAN|HGNC=9862|UniProtKB=P61225	HUMAN|HGNC=9862|UniProtKB=P61225	Ras-related protein Rap-2b;RAP2B;ortholog	RAS-RELATED PROTEIN RAP-2B (PTHR24070:SF346)
HUMAN|HGNC=673|UniProtKB=Q07960		Rho GTPase-activating protein 1;ARHGAP1;ortholog	RHO GTPASE-ACTIVATING PROTEIN 1 (PTHR23176:SF59)
HUMAN|HGNC=668|UniProtKB=P62745	HUMAN|HGNC=668|UniProtKB=P62745	Rho-related GTP-binding protein RhoB;RHOB;ortholog	RHO-RELATED GTP-BINDING PROTEIN RHOB (PTHR24072:SF182)
	HUMAN|HGNC=672|UniProtKB=P84095	Rho-related GTP-binding protein RhoG;RHOG;ortholog	RHO-RELATED GTP-BINDING PROTEIN RHOG (PTHR24072:SF210)
HUMAN|HGNC=1717|UniProtKB=Q16181		Septin-7;SEPT7;ortholog	SEPTIN-7 (PTHR18884:SF86)
	HUMAN|HGNC=15931|UniProtKB=O15079	Syntaphilin;SNPH;ortholog	SYNTAPHILIN (PTHR16208:SF7)
HUMAN|HGNC=667|UniProtKB=P61586		Transforming protein RhoA;RHOA;ortholog	TRANSFORMING PROTEIN RHOA (PTHR24072:SF212)
**extracellular matrix protein (PC00102)**			
	HUMAN|HGNC=5273|UniProtKB=P98160	Basement membrane-specific heparan sulfate proteoglycan core protein;HSPG2;ortholog	BASEMENT MEMBRANE-SPECIFIC HEPARAN SULFATE PROTEOGLYCAN CORE PROTEIN (PTHR10574:SF343)
HUMAN|HGNC=23059|UniProtKB=Q96GW7	HUMAN|HGNC=23059|UniProtKB=Q96GW7	Brevican core protein;BCAN;ortholog	BREVICAN CORE PROTEIN (PTHR22804:SF45)
HUMAN|HGNC=8011|UniProtKB=P78357	HUMAN|HGNC=8011|UniProtKB=P78357	Contactin-associated protein 1;CNTNAP1;ortholog	CONTACTIN-ASSOCIATED PROTEIN 1 (PTHR10127:SF740)
HUMAN|HGNC=2380|UniProtKB=P10915	HUMAN|HGNC=2380|UniProtKB=P10915	Hyaluronan and proteoglycan link protein 1;HAPLN1;ortholog	HYALURONAN AND PROTEOGLYCAN LINK PROTEIN 1 (PTHR22804:SF46)
HUMAN|HGNC=17410|UniProtKB=Q9GZV7	HUMAN|HGNC=17410|UniProtKB=Q9GZV7	Hyaluronan and proteoglycan link protein 2;HAPLN2;ortholog	HYALURONAN AND PROTEOGLYCAN LINK PROTEIN 2 (PTHR22804:SF51)
	HUMAN|HGNC=6487|UniProtKB=P55268	Laminin subunit beta-2;LAMB2;ortholog	LAMININ SUBUNIT BETA-2 (PTHR10574:SF346)
	HUMAN|HGNC=6492|UniProtKB=P11047	Laminin subunit gamma-1;LAMC1;ortholog	LAMININ SUBUNIT GAMMA-1 (PTHR10574:SF309)
HUMAN|HGNC=2465|UniProtKB=O14594	HUMAN|HGNC=2465|UniProtKB=O14594	Neurocan core protein;NCAN;ortholog	NEUROCAN CORE PROTEIN (PTHR22804:SF53)
HUMAN|HGNC=8135|UniProtKB=P23515	HUMAN|HGNC=8135|UniProtKB=P23515	Oligodendrocyte-myelin glycoprotein;OMG;ortholog	OLIGODENDROCYTE-MYELIN GLYCOPROTEIN (PTHR24373:SF208)
**hydrolase (PC00121)**			
HUMAN|HGNC=2158|UniProtKB=P09543		2',3'-cyclic-nucleotide 3'-phosphodiesterase;CNP;ortholog	2',3'-CYCLIC-NUCLEOTIDE 3'-PHOSPHODIESTERASE (PTHR10156:SF2)
HUMAN|HGNC=9553|UniProtKB=P62333	HUMAN|HGNC=9553|UniProtKB=P62333	26S protease regulatory subunit 10B;PSMC6;ortholog	26S PROTEASE REGULATORY SUBUNIT 10B (PTHR23073:SF49)
	HUMAN|HGNC=9548|UniProtKB=P35998	26S protease regulatory subunit 7;PSMC2;ortholog	26S PROTEASE REGULATORY SUBUNIT 7 (PTHR23073:SF46)
HUMAN|HGNC=823|UniProtKB=P25705	HUMAN|HGNC=823|UniProtKB=P25705	ATP synthase subunit alpha, mitochondrial;ATP5A1;ortholog	ATP SYNTHASE SUBUNIT ALPHA, MITOCHONDRIAL (PTHR43089:SF1)
HUMAN|HGNC=830|UniProtKB=P06576	HUMAN|HGNC=830|UniProtKB=P06576	ATP synthase subunit beta, mitochondrial;ATP5B;ortholog	ATP SYNTHASE SUBUNIT BETA, MITOCHONDRIAL (PTHR15184:SF59)
HUMAN|HGNC=850|UniProtKB=P48047	HUMAN|HGNC=850|UniProtKB=P48047	ATP synthase subunit O, mitochondrial;ATP5O;ortholog	ATP SYNTHASE SUBUNIT O, MITOCHONDRIAL (PTHR11910:SF9)
HUMAN|HGNC=1476|UniProtKB=P07384		Calpain-1 catalytic subunit;CAPN1;ortholog	CALPAIN-1 CATALYTIC SUBUNIT (PTHR10183:SF343)
HUMAN|HGNC=2529|UniProtKB=P07339	HUMAN|HGNC=2529|UniProtKB=P07339	Cathepsin D;CTSD;ortholog	CATHEPSIN D (PTHR13683:SF487)
HUMAN|HGNC=1932|UniProtKB=P36222		Chitinase-3-like protein 1;CHI3L1;ortholog	CHITINASE-3-LIKE PROTEIN 1 (PTHR11177:SF249)
HUMAN|HGNC=8011|UniProtKB=P78357	HUMAN|HGNC=8011|UniProtKB=P78357	Contactin-associated protein 1;CNTNAP1;ortholog	CONTACTIN-ASSOCIATED PROTEIN 1 (PTHR10127:SF740)
HUMAN|HGNC=12585|UniProtKB=P31930	HUMAN|HGNC=12585|UniProtKB=P31930	Cytochrome b-c1 complex subunit 1, mitochondrial;UQCRC1;ortholog	CYTOCHROME B-C1 COMPLEX SUBUNIT 1, MITOCHONDRIAL (PTHR11851:SF161)

HUMAN|HGNC=12586|UniProtKB=P22695	HUMAN|HGNC=12586|UniProtKB=P22695	Cytochrome b-c1 complex subunit 2, mitochondrial;UQCRC2;ortholog	CYTOCHROME B-C1 COMPLEX SUBUNIT 2, MITOCHONDRIAL (PTHR11851:SF172)
HUMAN|HGNC=2961|UniProtKB=Q14204	HUMAN|HGNC=2961|UniProtKB=Q14204	Cytoplasmic dynein 1 heavy chain 1;DYNC1H1;ortholog	CYTOPLASMIC DYNEIN 1 HEAVY CHAIN 1 (PTHR10676:SF302)
HUMAN|HGNC=16991|UniProtKB=Q07065	HUMAN|HGNC=16991|UniProtKB=Q07065	Cytoskeleton-associated protein 4;CKAP4;ortholog	CYTOSKELETON-ASSOCIATED PROTEIN 4 (PTHR18937:SF294)
HUMAN|HGNC=24437|UniProtKB=Q96KP4		Cytosolic non-specific dipeptidase;CNDP2;ortholog	CYTOSOLIC NON-SPECIFIC DIPEPTIDASE (PTHR43270:SF6)
HUMAN|HGNC=3015|UniProtKB=Q14195	HUMAN|HGNC=3015|UniProtKB=Q14195	Dihydropyrimidinase-related protein 3;DPYSL3;ortholog	DIHYDROPYRIMIDINASE-RELATED PROTEIN 3 (PTHR11647:SF86)
HUMAN|HGNC=3016|UniProtKB=O14531	HUMAN|HGNC=3016|UniProtKB=O14531	Dihydropyrimidinase-related protein 4;DPYSL4;ortholog	DIHYDROPYRIMIDINASE-RELATED PROTEIN 4 (PTHR11647:SF82)
HUMAN|HGNC=20637|UniProtKB=Q9BPU6		Dihydropyrimidinase-related protein 5;DPYSL5;ortholog	DIHYDROPYRIMIDINASE-RELATED PROTEIN 5 (PTHR11647:SF85)
HUMAN|HGNC=3013|UniProtKB=Q14117		Dihydropyrimidinase;DPYS;ortholog	DIHYDROPYRIMIDINASE (PTHR11647:SF74)
HUMAN|HGNC=8140|UniProtKB=O60313	HUMAN|HGNC=8140|UniProtKB=O60313	Dynamin-like 120 kDa protein, mitochondrial;OPA1;ortholog	DYNAMIN-LIKE 120 KDA PROTEIN, MITOCHONDRIAL (PTHR11566:SF100)
HUMAN|HGNC=23409|UniProtKB=Q6UWR7		Ectonucleotide pyrophosphatase/phosphodiesterase family member 6;ENPP6;ortholog	ECTONUCLEOTIDE PYROPHOSPHATASE/PHOSPHODIESTERASE FAMILY MEMBER 6 (PTHR10151:SF82)
HUMAN|HGNC=3189|UniProtKB=P68104	HUMAN|HGNC=3189|UniProtKB=P68104	Elongation factor 1-alpha 1;EEF1A1;ortholog	ELONGATION FACTOR 1-ALPHA 1 (PTHR23115:SF216)
HUMAN|HGNC=3192|UniProtKB=Q05639	HUMAN|HGNC=3192|UniProtKB=Q05639	Elongation factor 1-alpha 2;EEF1A2;ortholog	ELONGATION FACTOR 1-ALPHA 2 (PTHR23115:SF203)
HUMAN|HGNC=3214|UniProtKB=P13639	HUMAN|HGNC=3214|UniProtKB=P13639	Elongation factor 2;EEF2;ortholog	ELONGATION FACTOR 2 (PTHR42908:SF8)
HUMAN|HGNC=12420|UniProtKB=P49411	HUMAN|HGNC=12420|UniProtKB=P49411	Elongation factor Tu, mitochondrial;TUFM;ortholog	ELONGATION FACTOR TU, MITOCHONDRIAL (PTHR43721:SF7)
	HUMAN|HGNC=3267|UniProtKB=P41091	Eukaryotic translation initiation factor 2 subunit 3;EIF2S3;ortholog	EUKARYOTIC TRANSLATION INITIATION FACTOR 2 SUBUNIT 3 (PTHR42854:SF1)
HUMAN|HGNC=3594|UniProtKB=P49327	HUMAN|HGNC=3594|UniProtKB=P49327	Fatty acid synthase;FASN;ortholog	FATTY ACID SYNTHASE (PTHR43775:SF2)
HUMAN|HGNC=4331|UniProtKB=O94925	HUMAN|HGNC=4331|UniProtKB=O94925	Glutaminase kidney isoform, mitochondrial;GLS;ortholog	GLUTAMINASE KIDNEY ISOFORM, MITOCHONDRIAL (PTHR12544:SF41)
HUMAN|HGNC=4212|UniProtKB=Q9Y2T3		Guanine deaminase;GDA;ortholog	GUANINE DEAMINASE (PTHR11271:SF47)
HUMAN|HGNC=4398|UniProtKB=P62879	HUMAN|HGNC=4398|UniProtKB=P62879	Guanine nucleotide-binding protein G(I)/G(S)/G(T) subunit beta-2;GNB2;ortholog	GUANINE NUCLEOTIDE-BINDING PROTEIN G(I)/G(S)/G(T) SUBUNIT BETA-2 (PTHR19850:SF39)
HUMAN|HGNC=20731|UniProtKB=Q9HAV0		Guanine nucleotide-binding protein subunit beta-4;GNB4;ortholog	GUANINE NUCLEOTIDE-BINDING PROTEIN SUBUNIT BETA-4 (PTHR19850:SF42)
HUMAN|HGNC=4413|UniProtKB=P36915		Guanine nucleotide-binding protein-like 1;GNL1;ortholog	GUANINE NUCLEOTIDE-BINDING PROTEIN-LIKE 1 (PTHR11089:SF55)
HUMAN|HGNC=28246|UniProtKB=Q96ID5		Immunoglobulin superfamily member 21;IGSF21;ortholog	IMMUNOGLOBULIN SUPERFAMILY MEMBER 21 (PTHR10489:SF771)
HUMAN|HGNC=33711|UniProtKB=Q68D91	HUMAN|HGNC=33711|UniProtKB=Q68D91	Metallo-beta-lactamase domain-containing protein 2;MBLAC2;ortholog	METALLO-BETA-LACTAMASE DOMAIN-CONTAINING PROTEIN 2 (PTHR42951:SF4)
HUMAN|HGNC=10542|UniProtKB=O95248	HUMAN|HGNC=10542|UniProtKB=O95248	Myotubularin-related protein 5;SBF1;ortholog	MYOTUBULARIN-RELATED PROTEIN 5 (PTHR10807:SF91)
HUMAN|HGNC=2715|UniProtKB=O94760		N(G),N(G)-dimethylarginine dimethylaminohydrolase 1;DDAH1;ortholog	N(G),N(G)-DIMETHYLARGININE DIMETHYLAMINOHYDROLASE 1 (PTHR12737:SF25)
HUMAN|HGNC=7656|UniProtKB=P13591		Neural cell adhesion molecule 1;NCAM1;ortholog	NEURAL CELL ADHESION MOLECULE 1 (PTHR10489:SF783)
HUMAN|HGNC=29260|UniProtKB=Q6PIU2	HUMAN|HGNC=29260|UniProtKB=Q6PIU2	Neutral cholesterol ester hydrolase 1;NCEH1;ortholog	NEUTRAL CHOLESTEROL ESTER HYDROLASE 1 (PTHR23024:SF275)
HUMAN|HGNC=9325|UniProtKB=P50897	HUMAN|HGNC=9325|UniProtKB=P50897	Palmitoyl-protein thioesterase 1;PPT1;ortholog	PALMITOYL-PROTEIN THIOESTERASE 1 (PTHR11247:SF36)
HUMAN|HGNC=30009|UniProtKB=Q9UBV8		Peflin;PEF1;ortholog	PEFLIN (PTHR10183:SF361)

HUMAN|HGNC=8574|UniProtKB=P43034		Platelet-activating factor acetylhydrolase IB subunit alpha;PAFAH1B1;ortholog	PLATELET-ACTIVATING FACTOR ACETYLHYDROLASE IB SUBUNIT ALPHA (PTHR22847:SF549)
HUMAN|HGNC=8765|UniProtKB=O75340	HUMAN|HGNC=8765|UniProtKB=O75340	Programmed cell death protein 6;PDCD6;ortholog	PROGRAMMED CELL DEATH PROTEIN 6 (PTHR10183:SF367)
	HUMAN|HGNC=8550|UniProtKB=Q9UQ80	Proliferation-associated protein 2G4;PA2G4;ortholog	PROLIFERATION-ASSOCIATED PROTEIN 2G4 (PTHR10804:SF123)
HUMAN|HGNC=18341|UniProtKB=Q9Y2J8	HUMAN|HGNC=18341|UniProtKB=Q9Y2J8	Protein-arginine deiminase type-2;PADI2;ortholog	PROTEIN-ARGININE DEIMINASE TYPE-2 (PTHR10837:SF20)
	HUMAN|HGNC=33782|UniProtKB=Q6GMV3	Putative peptidyl-tRNA hydrolase PTRHD1;PTRHD1;ortholog	PEPTIDYL-TRNA HYDROLASE PTRHD1-RELATED (PTHR11717:SF25)
HUMAN|HGNC=30259|UniProtKB=Q96GD0		Pyridoxal phosphate phosphatase;PDXP;ortholog	PYRIDOXAL PHOSPHATE PHOSPHATASE (PTHR19288:SF47)
HUMAN|HGNC=9304|UniProtKB=P63151	HUMAN|HGNC=9304|UniProtKB=P63151	Serine/threonine-protein phosphatase 2A 55 kDa regulatory subunit B alpha isoform;PPP2R2A;ortholog	SERINE/THREONINE-PROTEIN PHOSPHATASE 2A 55 KDA REGULATORY SUBUNIT B ALPHA ISOFORM (PTHR11871:SF22)
HUMAN|HGNC=9302|UniProtKB=P30153	HUMAN|HGNC=9302|UniProtKB=P30153	Serine/threonine-protein phosphatase 2A 65 kDa regulatory subunit A alpha isoform;PPP2R1A;ortholog	SERINE/THREONINE-PROTEIN PHOSPHATASE 2A 65 KDA REGULATORY SUBUNIT A ALPHA ISOFORM (PTHR10648:SF16)
HUMAN|HGNC=9282|UniProtKB=P62140	HUMAN|HGNC=9282|UniProtKB=P62140	Serine/threonine-protein phosphatase PP1-beta catalytic subunit;PPP1CB;ortholog	SERINE/THREONINE-PROTEIN PHOSPHATASE PP1-BETA CATALYTIC SUBUNIT (PTHR11668:SF346)
HUMAN|HGNC=11740|UniProtKB=P02787		Serotransferrin;TF;ortholog	SEROTRANSFERRIN (PTHR11485:SF35)
	HUMAN|HGNC=2468|UniProtKB=Q9UQE7	Structural maintenance of chromosomes protein 3;SMC3;ortholog	STRUCTURAL MAINTENANCE OF CHROMOSOMES PROTEIN 3 (PTHR18937:SF288)
	HUMAN|HGNC=12513|UniProtKB=P09936	Ubiquitin carboxyl-terminal hydrolase isozyme L1;UCHL1;ortholog	UBIQUITIN CARBOXYL-TERMINAL HYDROLASE ISOZYME L1 (PTHR10589:SF33)
HUMAN|HGNC=854|UniProtKB=P21281	HUMAN|HGNC=854|UniProtKB=P21281	V-type proton ATPase subunit B, brain isoform;ATP6V1B2;ortholog	V-TYPE PROTON ATPASE SUBUNIT B, BRAIN ISOFORM (PTHR43389:SF1)
HUMAN|HGNC=856|UniProtKB=P21283	HUMAN|HGNC=856|UniProtKB=P21283	V-type proton ATPase subunit C 1;ATP6V1C1;ortholog	V-TYPE PROTON ATPASE SUBUNIT C 1 (PTHR10137:SF6)
HUMAN|HGNC=862|UniProtKB=O95670		V-type proton ATPase subunit G 2;ATP6V1G2;ortholog	PROTEIN ATP6V1G2-DDX39B-RELATED (PTHR12713:SF17)
**isomerase (PC00135)**			
HUMAN|HGNC=13518|UniProtKB=Q9Y696		Chloride intracellular channel protein 4;CLIC4;ortholog	CHLORIDE INTRACELLULAR CHANNEL PROTEIN 4 (PTHR11260:SF443)
HUMAN|HGNC=3213|UniProtKB=P26641	HUMAN|HGNC=3213|UniProtKB=P26641	Elongation factor 1-gamma;EEF1G;ortholog	ELONGATION FACTOR 1-GAMMA (PTHR11260:SF420)
HUMAN|HGNC=3151|UniProtKB=P30084		Enoyl-CoA hydratase, mitochondrial;ECHS1;ortholog	ENOYL-COA HYDRATASE, MITOCHONDRIAL (PTHR11941:SF111)
HUMAN|HGNC=15968|UniProtKB=Q8TB36		Ganglioside-induced differentiation-associated protein 1;GDAP1;ortholog	GANGLIOSIDE-INDUCED DIFFERENTIATION-ASSOCIATED PROTEIN 1 (PTHR11260:SF391)
HUMAN|HGNC=20898|UniProtKB=Q6PCE3		Glucose 1,6-bisphosphate synthase;PGM2L1;ortholog	GLUCOSE 1,6-BISPHOSPHATE SYNTHASE (PTHR22573:SF60)
HUMAN|HGNC=4458|UniProtKB=P06744	HUMAN|HGNC=4458|UniProtKB=P06744	Glucose-6-phosphate isomerase;GPI;ortholog	GLUCOSE-6-PHOSPHATE ISOMERASE (PTHR11469:SF17)
HUMAN|HGNC=4799|UniProtKB=Q16836		Hydroxyacyl-coenzyme A dehydrogenase, mitochondrial;HADH;ortholog	HYDROXYACYL-COENZYME A DEHYDROGENASE, MITOCHONDRIAL (PTHR43561:SF3)
HUMAN|HGNC=5157|UniProtKB=P00492		Hypoxanthine-guanine phosphoribosyltransferase;HPRT1;ortholog	HYPOXANTHINE-GUANINE PHOSPHORIBOSYLTRANSFERASE (PTHR43340:SF7)
HUMAN|HGNC=890|UniProtKB=Q13825		Methylglutaconyl-CoA hydratase, mitochondrial;AUH;ortholog	METHYLGLUTACONYL-COA HYDRATASE, MITOCHONDRIAL (PTHR11941:SF104)
	HUMAN|HGNC=7526|UniProtKB=P22033	Methylmalonyl-CoA mutase, mitochondrial;MUT;ortholog	METHYLMALONYL-COA MUTASE, MITOCHONDRIAL (PTHR42710:SF1)
HUMAN|HGNC=9253|UniProtKB=P62937	HUMAN|HGNC=9253|UniProtKB=P62937	Peptidyl-prolyl cis-trans isomerase A;PPIA;ortholog	PEPTIDYL-PROLYL CIS-TRANS ISOMERASE-RELATED (PTHR11071:SF354)
HUMAN|HGNC=9255|UniProtKB=P23284	HUMAN|HGNC=9255|UniProtKB=P23284	Peptidyl-prolyl cis-trans isomerase B;PPIB;ortholog	PEPTIDYL-PROLYL CIS-TRANS ISOMERASE B (PTHR11071:SF330)
HUMAN|HGNC=3719|UniProtKB=Q00688		Peptidyl-prolyl cis-trans isomerase FKBP3;FKBP3;ortholog	PEPTIDYL-PROLYL CIS-TRANS ISOMERASE FKBP3 (PTHR10516:SF356)

HUMAN|HGNC=3720|UniProtKB=Q02790		Peptidyl-prolyl cis-trans isomerase FKBP4;FKBP4;ortholog	PEPTIDYL-PROLYL CIS-TRANS ISOMERASE FKBP4 (PTHR10516:SF332)
HUMAN|HGNC=3724|UniProtKB=Q14318		Peptidyl-prolyl cis-trans isomerase FKBP8;FKBP8;ortholog	PEPTIDYL-PROLYL CIS-TRANS ISOMERASE FKBP8 (PTHR10516:SF363)
	HUMAN|HGNC=8988|UniProtKB=Q13526	Peptidyl-prolyl cis-trans isomerase NIMA-interacting 1;PIN1;ortholog	PIN1-LIKE PROTEIN-RELATED (PTHR10657:SF22)
	HUMAN|HGNC=9260|UniProtKB=Q9Y3C6	Peptidyl-prolyl cis-trans isomerase-like 1;PPIL1;ortholog	PEPTIDYL-PROLYL CIS-TRANS ISOMERASE-LIKE 1 (PTHR11071:SF343)
HUMAN|HGNC=9592|UniProtKB=P41222	HUMAN|HGNC=9592|UniProtKB=P41222	Prostaglandin-H2 D-isomerase;PTGDS;ortholog	PROSTAGLANDIN-H2 D-ISOMERASE (PTHR11430:SF114)
HUMAN|HGNC=4801|UniProtKB=P40939	HUMAN|HGNC=4801|UniProtKB=P40939	Trifunctional enzyme subunit alpha, mitochondrial;HADHA;ortholog	TRIFUNCTIONAL ENZYME SUBUNIT ALPHA, MITOCHONDRIAL (PTHR43612:SF3)
**ligase (PC00142)**			
	HUMAN|HGNC=3418|UniProtKB=P07814	Bifunctional glutamate/proline--tRNA ligase;EPRS;ortholog	BIFUNCTIONAL GLUTAMATE/PROLINE--TRNA LIGASE (PTHR43382:SF3)
HUMAN|HGNC=7432|UniProtKB=P11586	HUMAN|HGNC=7432|UniProtKB=P11586	C-1-tetrahydrofolate synthase, cytoplasmic;MTHFD1;ortholog	C-1-TETRAHYDROFOLATE SYNTHASE, CYTOPLASMIC (PTHR43274:SF1)
HUMAN|HGNC=3151|UniProtKB=P30084		Enoyl-CoA hydratase, mitochondrial;ECHS1;ortholog	ENOYL-COA HYDRATASE, MITOCHONDRIAL (PTHR11941:SF111)
HUMAN|HGNC=3594|UniProtKB=P49327	HUMAN|HGNC=3594|UniProtKB=P49327	Fatty acid synthase;FASN;ortholog	FATTY ACID SYNTHASE (PTHR43775:SF2)
HUMAN|HGNC=4341|UniProtKB=P15104	HUMAN|HGNC=4341|UniProtKB=P15104	Glutamine synthetase;GLUL;ortholog	GLUTAMINE SYNTHETASE (PTHR20852:SF67)
HUMAN|HGNC=29685|UniProtKB=Q9NSE4	HUMAN|HGNC=29685|UniProtKB=Q9NSE4	Isoleucine--tRNA ligase, mitochondrial;IARS2;ortholog	ISOLEUCINE--TRNA LIGASE, MITOCHONDRIAL (PTHR42765:SF3)
	HUMAN|HGNC=29207|UniProtKB=Q8N1G4	Leucine-rich repeat-containing protein 47;LRRC47;ortholog	LEUCINE-RICH REPEAT-CONTAINING PROTEIN 47 (PTHR10947:SF7)
HUMAN|HGNC=3570|UniProtKB=O95573		Long-chain-fatty-acid--CoA ligase 3;ACSL3;ortholog	LONG-CHAIN-FATTY-ACID--COA LIGASE 3 (PTHR43272:SF20)
	HUMAN|HGNC=6215|UniProtKB=Q15046	Lysine--tRNA ligase;KARS;ortholog	LYSINE--TRNA LIGASE (PTHR42918:SF2)
HUMAN|HGNC=890|UniProtKB=Q13825		Methylglutaconyl-CoA hydratase, mitochondrial;AUH;ortholog	METHYLGLUTACONYL-COA HYDRATASE, MITOCHONDRIAL (PTHR11941:SF104)
HUMAN|HGNC=3592|UniProtKB=Q9Y285		Phenylalanine--tRNA ligase alpha subunit;FARSA;ortholog	PHENYLALANINE--TRNA LIGASE ALPHA SUBUNIT (PTHR11538:SF49)
HUMAN|HGNC=17800|UniProtKB=Q9NSD9	HUMAN|HGNC=17800|UniProtKB=Q9NSD9	Phenylalanine--tRNA ligase beta subunit;FARSB;ortholog	PHENYLALANINE--TRNA LIGASE BETA SUBUNIT (PTHR10947:SF5)
	HUMAN|HGNC=17095|UniProtKB=Q15031	Probable leucine--tRNA ligase, mitochondrial;LARS2;ortholog	LEUCINE--TRNA LIGASE, MITOCHONDRIAL-RELATED (PTHR43740:SF2)
HUMAN|HGNC=8636|UniProtKB=P11498	HUMAN|HGNC=8636|UniProtKB=P11498	Pyruvate carboxylase, mitochondrial;PC;ortholog	PYRUVATE CARBOXYLASE, MITOCHONDRIAL (PTHR43778:SF4)
	HUMAN|HGNC=11450|UniProtKB=Q96I99	Succinyl-CoA ligase [GDP-forming] subunit beta, mitochondrial;SUCLG2;ortholog	SUCCINYL-COA LIGASE [GDP-FORMING] SUBUNIT BETA, MITOCHONDRIAL (PTHR11815:SF7)
	HUMAN|HGNC=30661|UniProtKB=Q9UBT2	SUMO-activating enzyme subunit 2;UBA2;ortholog	SUMO-ACTIVATING ENZYME SUBUNIT 2 (PTHR10953:SF166)
HUMAN|HGNC=15974|UniProtKB=Q9C040	HUMAN|HGNC=15974|UniProtKB=Q9C040	Tripartite motif-containing protein 2;TRIM2;ortholog	TRIPARTITE MOTIF-CONTAINING PROTEIN 2 (PTHR24103:SF446)
	HUMAN|HGNC=25581|UniProtKB=A0AVT1	Ubiquitin-like modifier-activating enzyme 6;UBA6;ortholog	UBIQUITIN-LIKE MODIFIER-ACTIVATING ENZYME 6 (PTHR10953:SF180)
**lyase (PC00144)**			
HUMAN|HGNC=118|UniProtKB=Q99798	HUMAN|HGNC=118|UniProtKB=Q99798	Aconitate hydratase, mitochondrial;ACO2;ortholog	ACONITATE HYDRATASE, MITOCHONDRIAL (PTHR43160:SF2)
HUMAN|HGNC=13238|UniProtKB=Q9HDC9		Adipocyte plasma membrane-associated protein;APMAP;ortholog	ADIPOCYTE PLASMA MEMBRANE-ASSOCIATED PROTEIN (PTHR10426:SF47)
HUMAN|HGNC=3350|UniProtKB=P06733	HUMAN|HGNC=3350|UniProtKB=P06733	Alpha-enolase;ENO1;ortholog	ALPHA-ENOLASE (PTHR11902:SF26)

HUMAN|HGNC=1373|UniProtKB=P00918		Carbonic anhydrase 2;CA2;ortholog	CARBONIC ANHYDRASE 2 (PTHR18952:SF159)
HUMAN|HGNC=1375|UniProtKB=P22748		Carbonic anhydrase 4;CA4;ortholog	CARBONIC ANHYDRASE 4 (PTHR18952:SF153)
HUMAN|HGNC=3151|UniProtKB=P30084		Enoyl-CoA hydratase, mitochondrial;ECHS1;ortholog	ENOYL-COA HYDRATASE, MITOCHONDRIAL (PTHR11941:SF111)
HUMAN|HGNC=3353|UniProtKB=P09104		Gamma-enolase;ENO2;ortholog	GAMMA-ENOLASE (PTHR11902:SF21)
	HUMAN|HGNC=4092|UniProtKB=Q99259	Glutamate decarboxylase 1;GAD1;ortholog	GLUTAMATE DECARBOXYLASE 1 (PTHR11999:SF119)
	HUMAN|HGNC=4093|UniProtKB=Q05329	Glutamate decarboxylase 2;GAD2;ortholog	GLUTAMATE DECARBOXYLASE 2 (PTHR11999:SF115)
HUMAN|HGNC=4799|UniProtKB=Q16836		Hydroxyacyl-coenzyme A dehydrogenase, mitochondrial;HADH;ortholog	HYDROXYACYL-COENZYME A DEHYDROGENASE, MITOCHONDRIAL (PTHR43561:SF3)
	HUMAN|HGNC=5007|UniProtKB=Q01581	Hydroxymethylglutaryl-CoA synthase, cytoplasmic;HMGCS1;ortholog	HYDROXYMETHYLGLUTARYL-COA SYNTHASE, CYTOPLASMIC (PTHR43323:SF6)
HUMAN|HGNC=890|UniProtKB=Q13825		Methylglutaconyl-CoA hydratase, mitochondrial;AUH;ortholog	METHYLGLUTACONYL-COA HYDRATASE, MITOCHONDRIAL (PTHR11941:SF104)
HUMAN|HGNC=6984|UniProtKB=P23368		NAD-dependent malic enzyme, mitochondrial;ME2;ortholog	NAD-DEPENDENT MALIC ENZYME, MITOCHONDRIAL (PTHR23406:SF50)
	HUMAN|HGNC=17981|UniProtKB=O00442	RNA 3'-terminal phosphate cyclase;RTCA;ortholog	RNA 3'-TERMINAL PHOSPHATE CYCLASE (PTHR11096:SF2)
HUMAN|HGNC=11834|UniProtKB=P29401	HUMAN|HGNC=11834|UniProtKB=P29401	Transketolase;TKT;ortholog	TRANSKETOLASE (PTHR43195:SF3)
HUMAN|HGNC=4801|UniProtKB=P40939	HUMAN|HGNC=4801|UniProtKB=P40939	Trifunctional enzyme subunit alpha, mitochondrial;HADHA;ortholog	TRIFUNCTIONAL ENZYME SUBUNIT ALPHA, MITOCHONDRIAL (PTHR43612:SF3)
**membrane traffic protein (PC00150)**			
	HUMAN|HGNC=7641|UniProtKB=P54920	Alpha-soluble NSF attachment protein;NAPA;ortholog	ALPHA-SOLUBLE NSF ATTACHMENT PROTEIN (PTHR13768:SF20)
	HUMAN|HGNC=567|UniProtKB=Q13367	AP-3 complex subunit beta-2;AP3B2;ortholog	AP-3 COMPLEX SUBUNIT BETA-2 (PTHR11134:SF21)
HUMAN|HGNC=16695|UniProtKB=P51572		B-cell receptor-associated protein 31;BCAP31;ortholog	B-CELL RECEPTOR-ASSOCIATED PROTEIN 31 (PTHR12701:SF28)
HUMAN|HGNC=15751|UniProtKB=Q9H115	HUMAN|HGNC=15751|UniProtKB=Q9H115	Beta-soluble NSF attachment protein;NAPB;ortholog	BETA-SOLUBLE NSF ATTACHMENT PROTEIN (PTHR13768:SF19)
HUMAN|HGNC=2236|UniProtKB=Q9Y678		Coatomer subunit gamma-1;COPG1;ortholog	COATOMER SUBUNIT GAMMA-1 (PTHR10261:SF7)
HUMAN|HGNC=2316|UniProtKB=O75131	HUMAN|HGNC=2316|UniProtKB=O75131	Copine-3;CPNE3;ortholog	COPINE-3 (PTHR10857:SF58)
HUMAN|HGNC=2317|UniProtKB=Q96A23	HUMAN|HGNC=2317|UniProtKB=Q96A23	Copine-4;CPNE4;ortholog	COPINE-4 (PTHR10857:SF78)
HUMAN|HGNC=2318|UniProtKB=Q9HCH3		Copine-5;CPNE5;ortholog	COPINE-5 (PTHR10857:SF69)
HUMAN|HGNC=2319|UniProtKB=O95741	HUMAN|HGNC=2319|UniProtKB=O95741	Copine-6;CPNE6;ortholog	COPINE-6 (PTHR10857:SF66)
	HUMAN|HGNC=3243|UniProtKB=Q9NZN4	EH domain-containing protein 2;EHD2;ortholog	EH DOMAIN-CONTAINING PROTEIN 2 (PTHR11216:SF106)
HUMAN|HGNC=3244|UniProtKB=Q9NZN3	HUMAN|HGNC=3244|UniProtKB=Q9NZN3	EH domain-containing protein 3;EHD3;ortholog	EH DOMAIN-CONTAINING PROTEIN 3 (PTHR11216:SF88)
	HUMAN|HGNC=10696|UniProtKB=O00471	Exocyst complex component 5;EXOC5;ortholog	EXOCYST COMPLEX COMPONENT 5 (PTHR12100:SF2)
HUMAN|HGNC=7642|UniProtKB=Q99747		Gamma-soluble NSF attachment protein;NAPG;ortholog	GAMMA-SOLUBLE NSF ATTACHMENT PROTEIN (PTHR13768:SF18)
HUMAN|HGNC=11141|UniProtKB=O76070		Gamma-synuclein;SNCG;ortholog	GAMMA-SYNUCLEIN (PTHR13820:SF14)
HUMAN|HGNC=30904|UniProtKB=O60763	HUMAN|HGNC=30904|UniProtKB=O60763	General vesicular transport factor p115;USO1;ortholog	GENERAL VESICULAR TRANSPORT FACTOR P115 (PTHR10013:SF1)
HUMAN|HGNC=6501|UniProtKB=P13473		Lysosome-associated membrane glycoprotein 2;LAMP2;ortholog	LYSOSOME-ASSOCIATED MEMBRANE GLYCOPROTEIN 2 (PTHR11506:SF40)
HUMAN|HGNC=30285|UniProtKB=Q7L099	HUMAN|HGNC=30285|UniProtKB=Q7L099	Protein RUFY3;RUFY3;ortholog	PROTEIN RUFY3 (PTHR22835:SF332)

HUMAN|HGNC=11172|UniProtKB=Q13596		Sorting nexin-1;SNX1;ortholog	SORTING NEXIN-1 (PTHR10555:SF192)
	HUMAN|HGNC=11174|UniProtKB=O60493	Sorting nexin-3;SNX3;ortholog	SORTING NEXIN-3 (PTHR10555:SF161)
	HUMAN|HGNC=11175|UniProtKB=O95219	Sorting nexin-4;SNX4;ortholog	SORTING NEXIN-4 (PTHR10555:SF199)
HUMAN|HGNC=11496|UniProtKB=O14994	HUMAN|HGNC=11496|UniProtKB=O14994	Synapsin-3;SYN3;ortholog	SYNAPSIN-3 (PTHR10841:SF25)
	HUMAN|HGNC=11501|UniProtKB=O43761	Synaptogyrin-3;SYNGR3;ortholog	SYNAPTOGYRIN-3 (PTHR10838:SF28)
HUMAN|HGNC=11506|UniProtKB=P08247	HUMAN|HGNC=11506|UniProtKB=P08247	Synaptophysin;SYP;ortholog	SYNAPTOPHYSIN (PTHR10306:SF25)
HUMAN|HGNC=11132|UniProtKB=P60880	HUMAN|HGNC=11132|UniProtKB=P60880	Synaptosomal-associated protein 25;SNAP25;ortholog	SYNAPTOSOMAL-ASSOCIATED PROTEIN 25 (PTHR19305:SF17)
HUMAN|HGNC=11514|UniProtKB=O43581	HUMAN|HGNC=11514|UniProtKB=O43581	Synaptotagmin-7;SYT7;ortholog	SYNAPTOTAGMIN-7 (PTHR10024:SF342)
	HUMAN|HGNC=15931|UniProtKB=O15079	Syntaphilin;SNPH;ortholog	SYNTAPHILIN (PTHR16208:SF7)
HUMAN|HGNC=11433|UniProtKB=Q16623		Syntaxin-1A;STX1A;ortholog	SYNTAXIN-1A (PTHR19957:SF187)
	HUMAN|HGNC=11444|UniProtKB=P61764	Syntaxin-binding protein 1;STXBP1;ortholog	SYNTAXIN-BINDING PROTEIN 1 (PTHR11679:SF54)
HUMAN|HGNC=11446|UniProtKB=O00186		Syntaxin-binding protein 3;STXBP3;ortholog	SYNTAXIN-BINDING PROTEIN 3 (PTHR11679:SF44)
HUMAN|HGNC=24878|UniProtKB=Q9BVK6		Transmembrane emp24 domain-containing protein 9;TMED9;ortholog	TRANSMEMBRANE EMP24 DOMAIN-CONTAINING PROTEIN 9 (PTHR22811:SF101)
HUMAN|HGNC=14340|UniProtKB=Q9UBQ0	HUMAN|HGNC=14340|UniProtKB=Q9UBQ0	Vacuolar protein sorting-associated protein 29;VPS29;ortholog	VACUOLAR PROTEIN SORTING-ASSOCIATED PROTEIN 29 (PTHR11124:SF17)
HUMAN|HGNC=13487|UniProtKB=Q96QK1	HUMAN|HGNC=13487|UniProtKB=Q96QK1	Vacuolar protein sorting-associated protein 35;VPS35;ortholog	VACUOLAR PROTEIN SORTING-ASSOCIATED PROTEIN 35 (PTHR11099:SF3)
HUMAN|HGNC=12648|UniProtKB=Q9P0L0	HUMAN|HGNC=12648|UniProtKB=Q9P0L0	Vesicle-associated membrane protein-associated protein A;VAPA;ortholog	VESICLE-ASSOCIATED MEMBRANE PROTEIN-ASSOCIATED PROTEIN A (PTHR10809:SF64)
HUMAN|HGNC=10700|UniProtKB=O75396	HUMAN|HGNC=10700|UniProtKB=O75396	Vesicle-trafficking protein SEC22b;SEC22B;ortholog	VESICLE-TRAFFICKING PROTEIN SEC22B (PTHR21136:SF160)
**nucleic acid binding (PC00171)**			
HUMAN|HGNC=10383|UniProtKB=P46783		40S ribosomal protein S10;RPS10;ortholog	40S RIBOSOMAL PROTEIN S10-RELATED (PTHR12146:SF9)
HUMAN|HGNC=10387|UniProtKB=P62263		40S ribosomal protein S14;RPS14;ortholog	40S RIBOSOMAL PROTEIN S14 (PTHR11759:SF13)
HUMAN|HGNC=10401|UniProtKB=P62269	HUMAN|HGNC=10401|UniProtKB=P62269	40S ribosomal protein S18;RPS18;ortholog	40S RIBOSOMAL PROTEIN S18 (PTHR10871:SF15)
HUMAN|HGNC=10402|UniProtKB=P39019		40S ribosomal protein S19;RPS19;ortholog	40S RIBOSOMAL PROTEIN S19 (PTHR11710:SF7)
HUMAN|HGNC=10405|UniProtKB=P60866		40S ribosomal protein S20;RPS20;ortholog	40S RIBOSOMAL PROTEIN S20 (PTHR11700:SF13)
	HUMAN|HGNC=10420|UniProtKB=P23396	40S ribosomal protein S3;RPS3;ortholog	40S RIBOSOMAL PROTEIN S3 (PTHR11760:SF26)
HUMAN|HGNC=10424|UniProtKB=P62701	HUMAN|HGNC=10424|UniProtKB=P62701	40S ribosomal protein S4, X isoform;RPS4X;ortholog	40S RIBOSOMAL PROTEIN S4, X ISOFORM (PTHR11581:SF21)
HUMAN|HGNC=10299|UniProtKB=P62906		60S ribosomal protein L10a;RPL10A;ortholog	60S RIBOSOMAL PROTEIN L10A (PTHR23105:SF87)
HUMAN|HGNC=10302|UniProtKB=P30050	HUMAN|HGNC=10302|UniProtKB=P30050	60S ribosomal protein L12;RPL12;ortholog	60S RIBOSOMAL PROTEIN L12 (PTHR11661:SF11)
HUMAN|HGNC=10303|UniProtKB=P26373	HUMAN|HGNC=10303|UniProtKB=P26373	60S ribosomal protein L13;RPL13;ortholog	60S RIBOSOMAL PROTEIN L13 (PTHR11722:SF2)
	HUMAN|HGNC=10316|UniProtKB=P62829	60S ribosomal protein L23;RPL23;ortholog	60S RIBOSOMAL PROTEIN L23 (PTHR11761:SF20)
HUMAN|HGNC=10317|UniProtKB=P62750		60S ribosomal protein L23a;RPL23A;ortholog	60S RIBOSOMAL PROTEIN L23A (PTHR11620:SF29)
HUMAN|HGNC=10328|UniProtKB=P61353		60S ribosomal protein L27;RPL27;ortholog	60S RIBOSOMAL PROTEIN L27 (PTHR10497:SF3)

HUMAN|HGNC=10332|UniProtKB=P39023		60S ribosomal protein L3;RPL3;ortholog	60S RIBOSOMAL PROTEIN L3 (PTHR11363:SF11)
HUMAN|HGNC=10362|UniProtKB=Q02878		60S ribosomal protein L6;RPL6;ortholog	60S RIBOSOMAL PROTEIN L6 (PTHR10715:SF4)
HUMAN|HGNC=10990|UniProtKB=P12235	HUMAN|HGNC=10990|UniProtKB=P12235	ADP/ATP translocase 1;SLC25A4;ortholog	ADP/ATP TRANSLOCASE 1 (PTHR24089:SF506)
HUMAN|HGNC=10991|UniProtKB=P05141	HUMAN|HGNC=10991|UniProtKB=P05141	ADP/ATP translocase 2;SLC25A5;ortholog	ADP/ATP TRANSLOCASE 2 (PTHR24089:SF446)
HUMAN|HGNC=10992|UniProtKB=P12236	HUMAN|HGNC=10992|UniProtKB=P12236	ADP/ATP translocase 3;SLC25A6;ortholog	ADP/ATP TRANSLOCASE 3 (PTHR24089:SF411)
HUMAN|HGNC=20|UniProtKB=P49588	HUMAN|HGNC=20|UniProtKB=P49588	Alanine--tRNA ligase, cytoplasmic;AARS;ortholog	ALANINE--TRNA LIGASE, CYTOPLASMIC (PTHR11777:SF24)
HUMAN|HGNC=6833|UniProtKB=P21397	HUMAN|HGNC=6833|UniProtKB=P21397	Amine oxidase [flavin-containing] A;MAOA;ortholog	AMINE OXIDASE [FLAVIN-CONTAINING] A (PTHR43563:SF4)
HUMAN|HGNC=6834|UniProtKB=P27338	HUMAN|HGNC=6834|UniProtKB=P27338	Amine oxidase [flavin-containing] B;MAOB;ortholog	AMINE OXIDASE [FLAVIN-CONTAINING] B (PTHR43563:SF6)
HUMAN|HGNC=823|UniProtKB=P25705	HUMAN|HGNC=823|UniProtKB=P25705	ATP synthase subunit alpha, mitochondrial;ATP5A1;ortholog	ATP SYNTHASE SUBUNIT ALPHA, MITOCHONDRIAL (PTHR43089:SF1)
HUMAN|HGNC=830|UniProtKB=P06576	HUMAN|HGNC=830|UniProtKB=P06576	ATP synthase subunit beta, mitochondrial;ATP5B;ortholog	ATP SYNTHASE SUBUNIT BETA, MITOCHONDRIAL (PTHR15184:SF59)
HUMAN|HGNC=2750|UniProtKB=Q08211	HUMAN|HGNC=2750|UniProtKB=Q08211	ATP-dependent RNA helicase A;DHX9;ortholog	ATP-DEPENDENT RNA HELICASE A (PTHR18934:SF176)
HUMAN|HGNC=10982|UniProtKB=O75746	HUMAN|HGNC=10982|UniProtKB=O75746	Calcium-binding mitochondrial carrier protein Aralar1;SLC25A12;ortholog	CALCIUM-BINDING MITOCHONDRIAL CARRIER PROTEIN ARALAR1 (PTHR24089:SF503)
	HUMAN|HGNC=10983|UniProtKB=Q9UJS0	Calcium-binding mitochondrial carrier protein Aralar2;SLC25A13;ortholog	CALCIUM-BINDING MITOCHONDRIAL CARRIER PROTEIN ARALAR2 (PTHR24089:SF428)
HUMAN|HGNC=13518|UniProtKB=Q9Y696		Chloride intracellular channel protein 4;CLIC4;ortholog	CHLORIDE INTRACELLULAR CHANNEL PROTEIN 4 (PTHR11260:SF443)
	HUMAN|HGNC=14453|UniProtKB=Q9P0M6	Core histone macro-H2A.2;H2AFY2;ortholog	CORE HISTONE MACRO-H2A.2 (PTHR23430:SF124)
HUMAN|HGNC=16991|UniProtKB=Q07065	HUMAN|HGNC=16991|UniProtKB=Q07065	Cytoskeleton-associated protein 4;CKAP4;ortholog	CYTOSKELETON-ASSOCIATED PROTEIN 4 (PTHR18937:SF294)
HUMAN|HGNC=3189|UniProtKB=P68104	HUMAN|HGNC=3189|UniProtKB=P68104	Elongation factor 1-alpha 1;EEF1A1;ortholog	ELONGATION FACTOR 1-ALPHA 1 (PTHR23115:SF216)
HUMAN|HGNC=3192|UniProtKB=Q05639	HUMAN|HGNC=3192|UniProtKB=Q05639	Elongation factor 1-alpha 2;EEF1A2;ortholog	ELONGATION FACTOR 1-ALPHA 2 (PTHR23115:SF203)
HUMAN|HGNC=3213|UniProtKB=P26641	HUMAN|HGNC=3213|UniProtKB=P26641	Elongation factor 1-gamma;EEF1G;ortholog	ELONGATION FACTOR 1-GAMMA (PTHR11260:SF420)
HUMAN|HGNC=3214|UniProtKB=P13639	HUMAN|HGNC=3214|UniProtKB=P13639	Elongation factor 2;EEF2;ortholog	ELONGATION FACTOR 2 (PTHR42908:SF8)
HUMAN|HGNC=12420|UniProtKB=P49411	HUMAN|HGNC=12420|UniProtKB=P49411	Elongation factor Tu, mitochondrial;TUFM;ortholog	ELONGATION FACTOR TU, MITOCHONDRIAL (PTHR43721:SF7)
HUMAN|HGNC=3282|UniProtKB=P60842	HUMAN|HGNC=3282|UniProtKB=P60842	Eukaryotic initiation factor 4A-I;EIF4A1;ortholog	EUKARYOTIC INITIATION FACTOR 4A-I (PTHR24031:SF379)
HUMAN|HGNC=3284|UniProtKB=Q14240	HUMAN|HGNC=3284|UniProtKB=Q14240	Eukaryotic initiation factor 4A-II;EIF4A2;ortholog	EUKARYOTIC INITIATION FACTOR 4A-II (PTHR24031:SF418)
	HUMAN|HGNC=3267|UniProtKB=P41091	Eukaryotic translation initiation factor 2 subunit 3;EIF2S3;ortholog	EUKARYOTIC TRANSLATION INITIATION FACTOR 2 SUBUNIT 3 (PTHR42854:SF1)
HUMAN|HGNC=3275|UniProtKB=O00303	HUMAN|HGNC=3275|UniProtKB=O00303	Eukaryotic translation initiation factor 3 subunit F;EIF3F;ortholog	EUKARYOTIC TRANSLATION INITIATION FACTOR 3 SUBUNIT F (PTHR10540:SF14)
	HUMAN|HGNC=3272|UniProtKB=Q13347	Eukaryotic translation initiation factor 3 subunit I;EIF3I;ortholog	EUKARYOTIC TRANSLATION INITIATION FACTOR 3 SUBUNIT I (PTHR19877:SF6)
HUMAN|HGNC=3299|UniProtKB=P55010		Eukaryotic translation initiation factor 5;EIF5;ortholog	EUKARYOTIC TRANSLATION INITIATION FACTOR 5 (PTHR23001:SF11)
HUMAN|HGNC=15968|UniProtKB=Q8TB36		Ganglioside-induced differentiation-associated protein 1;GDAP1;ortholog	GANGLIOSIDE-INDUCED DIFFERENTIATION-ASSOCIATED PROTEIN 1 (PTHR11260:SF391)
HUMAN|HGNC=4413|UniProtKB=P36915		Guanine nucleotide-binding protein-like 1;GNL1;ortholog	GUANINE NUCLEOTIDE-BINDING PROTEIN-LIKE 1 (PTHR11089:SF55)
HUMAN|HGNC=5030|UniProtKB=Q13151	HUMAN|HGNC=5030|UniProtKB=Q13151	Heterogeneous nuclear ribonucleoprotein A0;HNRNPA0;ortholog	HETEROGENEOUS NUCLEAR RIBONUCLEOPROTEIN A0-RELATED (PTHR24012:SF515)

	HUMAN|HGNC=5039|UniProtKB=P52597	Heterogeneous nuclear ribonucleoprotein F;HNRNPF;ortholog	HETEROGENEOUS NUCLEAR RIBONUCLEOPROTEIN F (PTHR13976:SF47)
HUMAN|HGNC=5042|UniProtKB=P55795	HUMAN|HGNC=5042|UniProtKB=P55795	Heterogeneous nuclear ribonucleoprotein H2;HNRNPH2;ortholog	HETEROGENEOUS NUCLEAR RIBONUCLEOPROTEIN H2 (PTHR13976:SF55)
HUMAN|HGNC=5047|UniProtKB=O43390		Heterogeneous nuclear ribonucleoprotein R;HNRNPR;ortholog	HETEROGENEOUS NUCLEAR RIBONUCLEOPROTEIN R (PTHR24012:SF670)
HUMAN|HGNC=4716|UniProtKB=P16403	HUMAN|HGNC=4716|UniProtKB=P16403	Histone H1.2;HIST1H1C;ortholog	HISTONE H1.2 (PTHR11467:SF76)
HUMAN|HGNC=4722|UniProtKB=Q92522	HUMAN|HGNC=4722|UniProtKB=Q92522	Histone H1x;H1FX;ortholog	HISTONE H1X (PTHR11467:SF77)
HUMAN|HGNC=4724|UniProtKB=P04908		Histone H2A type 1-B/E;HIST1H2AB;ortholog	HISTONE H2A TYPE 1-B/E (PTHR23430:SF129)
HUMAN|HGNC=4734|UniProtKB=P04908		Histone H2A type 1-B/E;HIST1H2AB;ortholog	HISTONE H2A TYPE 1-B/E (PTHR23430:SF174)
HUMAN|HGNC=13671|UniProtKB=Q96KK5	HUMAN|HGNC=13671|UniProtKB=Q96KK5	Histone H2A type 1-H;HIST1H2AH;ortholog	HISTONE H2A TYPE 1-H (PTHR23430:SF160)
HUMAN|HGNC=20508|UniProtKB=Q8IUE6	HUMAN|HGNC=20508|UniProtKB=Q8IUE6	Histone H2A type 2-B;HIST2H2AB;ortholog	HISTONE H2A TYPE 2-B (PTHR23430:SF154)
HUMAN|HGNC=13954|UniProtKB=O60814	HUMAN|HGNC=13954|UniProtKB=O60814	Histone H2B type 1-K;HIST1H2BK;ortholog	HISTONE H2B TYPE 1-K-RELATED (PTHR23428:SF110)
HUMAN|HGNC=20510|UniProtKB=P62805	HUMAN|HGNC=20510|UniProtKB=P62805	Histone H4;HIST1H4A;ortholog	HISTONE H4 (PTHR10484:SF122)
HUMAN|HGNC=29607|UniProtKB=P62805	HUMAN|HGNC=29607|UniProtKB=P62805	Histone H4;HIST1H4A;ortholog	HISTONE H4 (PTHR10484:SF152)
HUMAN|HGNC=4781|UniProtKB=P62805	HUMAN|HGNC=4781|UniProtKB=P62805	Histone H4;HIST1H4A;ortholog	HISTONE H4 (PTHR10484:SF150)
HUMAN|HGNC=4782|UniProtKB=P62805	HUMAN|HGNC=4782|UniProtKB=P62805	Histone H4;HIST1H4A;ortholog	HISTONE H4 (PTHR10484:SF137)
HUMAN|HGNC=4783|UniProtKB=P62805	HUMAN|HGNC=4783|UniProtKB=P62805	Histone H4;HIST1H4A;ortholog	HISTONE H4 (PTHR10484:SF156)
HUMAN|HGNC=4784|UniProtKB=P62805	HUMAN|HGNC=4784|UniProtKB=P62805	Histone H4;HIST1H4A;ortholog	HISTONE H4 (PTHR10484:SF91)
HUMAN|HGNC=4785|UniProtKB=P62805	HUMAN|HGNC=4785|UniProtKB=P62805	Histone H4;HIST1H4A;ortholog	HISTONE H4 (PTHR10484:SF144)
HUMAN|HGNC=4787|UniProtKB=P62805	HUMAN|HGNC=4787|UniProtKB=P62805	Histone H4;HIST1H4A;ortholog	HISTONE H4 (PTHR10484:SF101)
HUMAN|HGNC=4788|UniProtKB=P62805	HUMAN|HGNC=4788|UniProtKB=P62805	Histone H4;HIST1H4A;ortholog	HISTONE H4 (PTHR10484:SF127)
HUMAN|HGNC=4789|UniProtKB=P62805	HUMAN|HGNC=4789|UniProtKB=P62805	Histone H4;HIST1H4A;ortholog	HISTONE H4 (PTHR10484:SF129)
HUMAN|HGNC=4790|UniProtKB=P62805	HUMAN|HGNC=4790|UniProtKB=P62805	Histone H4;HIST1H4A;ortholog	HISTONE H4 (PTHR10484:SF113)
HUMAN|HGNC=4791|UniProtKB=P62805	HUMAN|HGNC=4791|UniProtKB=P62805	Histone H4;HIST1H4A;ortholog	HISTONE H4 (PTHR10484:SF107)
HUMAN|HGNC=4793|UniProtKB=P62805	HUMAN|HGNC=4793|UniProtKB=P62805	Histone H4;HIST1H4A;ortholog	HISTONE H4 (PTHR10484:SF134)
HUMAN|HGNC=4794|UniProtKB=P62805	HUMAN|HGNC=4794|UniProtKB=P62805	Histone H4;HIST1H4A;ortholog	HISTONE H4 (PTHR10484:SF123)
HUMAN|HGNC=15714|UniProtKB=P42704	HUMAN|HGNC=15714|UniProtKB=P42704	Leucine-rich PPR motif-containing protein, mitochondrial;LRPPRC;ortholog	LEUCINE-RICH PPR MOTIF-CONTAINING PROTEIN, MITOCHONDRIAL (PTHR24015:SF1304)
	HUMAN|HGNC=29207|UniProtKB=Q8N1G4	Leucine-rich repeat-containing protein 47;LRRC47;ortholog	LEUCINE-RICH REPEAT-CONTAINING PROTEIN 47 (PTHR10947:SF7)
HUMAN|HGNC=11316|UniProtKB=P05455		Lupus La protein;SSB;ortholog	LUPUS LA PROTEIN (PTHR22792:SF78)
	HUMAN|HGNC=6215|UniProtKB=Q15046	Lysine--tRNA ligase;KARS;ortholog	LYSINE--TRNA LIGASE (PTHR42918:SF2)
	HUMAN|HGNC=6912|UniProtKB=P43243	Matrin-3;MATR3;ortholog	MATRIN-3 (PTHR15592:SF12)
HUMAN|HGNC=6990|UniProtKB=P51608	HUMAN|HGNC=6990|UniProtKB=P51608	Methyl-CpG-binding protein 2;MECP2;ortholog	METHYL-CPG-BINDING PROTEIN 2 (PTHR15074:SF7)
	HUMAN|HGNC=19954|UniProtKB=Q9H936	Mitochondrial glutamate carrier 1;SLC25A22;ortholog	MITOCHONDRIAL GLUTAMATE CARRIER 1 (PTHR24089:SF598)

	HUMAN|HGNC=10988|UniProtKB=Q9H1K4	Mitochondrial glutamate carrier 2;SLC25A18;ortholog	MITOCHONDRIAL GLUTAMATE CARRIER 2 (PTHR24089:SF482)
	HUMAN|HGNC=7819|UniProtKB=P55769	NHP2-like protein 1;NHP2L1;ortholog	NHP2-LIKE PROTEIN 1 (PTHR23105:SF93)
HUMAN|HGNC=29926|UniProtKB=Q9Y2X3		Nucleolar protein 58;NOP58;ortholog	NUCLEOLAR PROTEIN 58 (PTHR10894:SF7)
HUMAN|HGNC=17800|UniProtKB=Q9NSD9	HUMAN|HGNC=17800|UniProtKB=Q9NSD9	Phenylalanine--tRNA ligase beta subunit;FARSB;ortholog	PHENYLALANINE--TRNA LIGASE BETA SUBUNIT (PTHR10947:SF5)
HUMAN|HGNC=8574|UniProtKB=P43034		Platelet-activating factor acetylhydrolase IB subunit alpha;PAFAH1B1;ortholog	PLATELET-ACTIVATING FACTOR ACETYLHYDROLASE IB SUBUNIT ALPHA (PTHR22847:SF549)
HUMAN|HGNC=270|UniProtKB=P09874	HUMAN|HGNC=270|UniProtKB=P09874	Poly [ADP-ribose] polymerase 1;PARP1;ortholog	POLY [ADP-RIBOSE] POLYMERASE 1 (PTHR10459:SF83)
HUMAN|HGNC=8647|UniProtKB=Q15365	HUMAN|HGNC=8647|UniProtKB=Q15365	Poly(rC)-binding protein 1;PCBP1;ortholog	POLY(RC)-BINDING PROTEIN 1 (PTHR10288:SF191)
	HUMAN|HGNC=2747|UniProtKB=P26196	Probable ATP-dependent RNA helicase DDX6;DDX6;ortholog	ATP-DEPENDENT RNA HELICASE DDX6-RELATED (PTHR24031:SF408)
	HUMAN|HGNC=8550|UniProtKB=Q9UQ80	Proliferation-associated protein 2G4;PA2G4;ortholog	PROLIFERATION-ASSOCIATED PROTEIN 2G4 (PTHR10804:SF123)
	HUMAN|HGNC=9280|UniProtKB=P50336	Protoporphyrinogen oxidase;PPOX;ortholog	PROTOPORPHYRINOGEN OXIDASE (PTHR42923:SF1)
HUMAN|HGNC=20517|UniProtKB=Q6DRA6	HUMAN|HGNC=20517|UniProtKB=Q6DRA6	Putative histone H2B type 2-D;HIST2H2BD;ortholog	HISTONE H2B TYPE 2-C-RELATED (PTHR23428:SF131)
	HUMAN|HGNC=10074|UniProtKB=P13489	Ribonuclease inhibitor;RNH1;ortholog	RIBONUCLEASE INHIBITOR (PTHR24112:SF52)
	HUMAN|HGNC=17981|UniProtKB=O00442	RNA 3'-terminal phosphate cyclase;RTCA;ortholog	RNA 3'-TERMINAL PHOSPHATE CYCLASE (PTHR11096:SF2)
	HUMAN|HGNC=11362|UniProtKB=P42224	Signal transducer and activator of transcription 1-alpha/beta;STAT1;ortholog	SIGNAL TRANSDUCER AND ACTIVATOR OF TRANSCRIPTION 1-ALPHA/BETA (PTHR11801:SF58)
HUMAN|HGNC=11159|UniProtKB=P62316	HUMAN|HGNC=11159|UniProtKB=P62316	Small nuclear ribonucleoprotein Sm D2;SNRPD2;ortholog	SMALL NUCLEAR RIBONUCLEOPROTEIN SM D2 (PTHR12777:SF2)
	HUMAN|HGNC=2468|UniProtKB=Q9UQE7	Structural maintenance of chromosomes protein 3;SMC3;ortholog	STRUCTURAL MAINTENANCE OF CHROMOSOMES PROTEIN 3 (PTHR18937:SF288)
HUMAN|HGNC=9701|UniProtKB=Q00577	HUMAN|HGNC=9701|UniProtKB=Q00577	Transcriptional activator protein Pur-alpha;PURA;ortholog	TRANSCRIPTIONAL ACTIVATOR PROTEIN PUR-ALPHA (PTHR12611:SF9)
HUMAN|HGNC=10979|UniProtKB=P53007	HUMAN|HGNC=10979|UniProtKB=P53007	Tricarboxylate transport protein, mitochondrial;SLC25A1;ortholog	TRICARBOXYLATE TRANSPORT PROTEIN, MITOCHONDRIAL (PTHR24089:SF459)
HUMAN|HGNC=854|UniProtKB=P21281	HUMAN|HGNC=854|UniProtKB=P21281	V-type proton ATPase subunit B, brain isoform;ATP6V1B2;ortholog	V-TYPE PROTON ATPASE SUBUNIT B, BRAIN ISOFORM (PTHR43389:SF1)
HUMAN|HGNC=12833|UniProtKB=P13010		X-ray repair cross-complementing protein 5;XRCC5;ortholog	X-RAY REPAIR CROSS-COMPLEMENTING PROTEIN 5 (PTHR12604:SF7)

oxidoreductase (PC00176)

HUMAN|HGNC=4907|UniProtKB=P31937		3-hydroxyisobutyrate dehydrogenase, mitochondrial;HIBADH;ortholog	3-HYDROXYISOBUTYRATE DEHYDROGENASE, MITOCHONDRIAL (PTHR22981:SF81)
HUMAN|HGNC=412|UniProtKB=P49189		4-trimethylaminobutyraldehyde dehydrogenase;ALDH9A1;ortholog	4-TRIMETHYLAMINOBUTYRALDEHYDE DEHYDROGENASE (PTHR11699:SF247)
HUMAN|HGNC=380|UniProtKB=P14550	HUMAN|HGNC=380|UniProtKB=P14550	Alcohol dehydrogenase [NADP(+)];AKR1A1;ortholog	ALCOHOL DEHYDROGENASE [NADP(+)] (PTHR11732:SF347)
HUMAN|HGNC=381|UniProtKB=P15121		Aldose reductase;AKR1B1;ortholog	ALDOSE REDUCTASE (PTHR11732:SF332)
HUMAN|HGNC=6833|UniProtKB=P21397	HUMAN|HGNC=6833|UniProtKB=P21397	Amine oxidase [flavin-containing] A;MAOA;ortholog	AMINE OXIDASE [FLAVIN-CONTAINING] A (PTHR43563:SF4)
HUMAN|HGNC=6834|UniProtKB=P27338	HUMAN|HGNC=6834|UniProtKB=P27338	Amine oxidase [flavin-containing] B;MAOB;ortholog	AMINE OXIDASE [FLAVIN-CONTAINING] B (PTHR43563:SF6)
HUMAN|HGNC=1548|UniProtKB=P16152	HUMAN|HGNC=1548|UniProtKB=P16152	Carbonyl reductase [NADPH] 1;CBR1;ortholog	CARBONYL REDUCTASE [NADPH] 1 (PTHR43490:SF35)
HUMAN|HGNC=13518|UniProtKB=Q9Y696		Chloride intracellular channel protein 4;CLIC4;ortholog	CHLORIDE INTRACELLULAR CHANNEL PROTEIN 4 (PTHR11260:SF443)
	HUMAN|HGNC=24288|UniProtKB=Q8NE62	Choline dehydrogenase, mitochondrial;CHDH;ortholog	CHOLINE DEHYDROGENASE, MITOCHONDRIAL (PTHR11552:SF147)

HUMAN|HGNC=23046|UniProtKB=Q5SYC1		Clavesin-2;CLVS2;ortholog	CLAVESIN-2 (PTHR10174:SF176)
HUMAN|HGNC=8011|UniProtKB=P78357	HUMAN|HGNC=8011|UniProtKB=P78357	Contactin-associated protein 1;CNTNAP1;ortholog	CONTACTIN-ASSOCIATED PROTEIN 1 (PTHR10127:SF740)
HUMAN|HGNC=12582|UniProtKB=P14927		Cytochrome b-c1 complex subunit 7;UQCRB;ortholog	CYTOCHROME B-C1 COMPLEX SUBUNIT 7 (PTHR12022:SF1)
HUMAN|HGNC=7421|UniProtKB=P00403	HUMAN|HGNC=7421|UniProtKB=P00403	Cytochrome c oxidase subunit 2;MT-CO2;ortholog	CYTOCHROME C OXIDASE SUBUNIT 2 (PTHR22888:SF14)
HUMAN|HGNC=2267|UniProtKB=P20674	HUMAN|HGNC=2267|UniProtKB=P20674	Cytochrome c oxidase subunit 5A, mitochondrial;COX5A;ortholog	CYTOCHROME C OXIDASE SUBUNIT 5A, MITOCHONDRIAL (PTHR14200:SF14)
HUMAN|HGNC=2269|UniProtKB=P10606	HUMAN|HGNC=2269|UniProtKB=P10606	Cytochrome c oxidase subunit 5B, mitochondrial;COX5B;ortholog	CYTOCHROME C OXIDASE SUBUNIT 5B, MITOCHONDRIAL (PTHR10122:SF4)
HUMAN|HGNC=2280|UniProtKB=P14854	HUMAN|HGNC=2280|UniProtKB=P14854	Cytochrome c oxidase subunit 6B1;COX6B1;ortholog	CYTOCHROME C OXIDASE SUBUNIT 6B1 (PTHR11387:SF26)
	HUMAN|HGNC=7687|UniProtKB=O00483	Cytochrome c oxidase subunit NDUFA4;NDUFA4;ortholog	CYTOCHROME C OXIDASE SUBUNIT NDUFA4 (PTHR14256:SF9)
HUMAN|HGNC=3978|UniProtKB=O75891		Cytosolic 10-formyltetrahydrofolate dehydrogenase;ALDH1L1;ortholog	CYTOSOLIC 10-FORMYLTETRAHYDROFOLATE DEHYDROGENASE (PTHR11699:SF220)
HUMAN|HGNC=8923|UniProtKB=O43175	HUMAN|HGNC=8923|UniProtKB=O43175	D-3-phosphoglycerate dehydrogenase;PHGDH;ortholog	D-3-PHOSPHOGLYCERATE DEHYDROGENASE (PTHR42938:SF9)
	HUMAN|HGNC=1027|UniProtKB=Q02338	D-beta-hydroxybutyrate dehydrogenase, mitochondrial;BDH1;ortholog	D-BETA-HYDROXYBUTYRATE DEHYDROGENASE, MITOCHONDRIAL (PTHR43313:SF21)
HUMAN|HGNC=9752|UniProtKB=P09417		Dihydropteridine reductase;QDPR;ortholog	DIHYDROPTERIDINE REDUCTASE (PTHR15104:SF1)
HUMAN|HGNC=3482|UniProtKB=P38117		Electron transfer flavoprotein subunit beta;ETFB;ortholog	ELECTRON TRANSFER FLAVOPROTEIN SUBUNIT BETA (PTHR21294:SF14)
HUMAN|HGNC=3213|UniProtKB=P26641	HUMAN|HGNC=3213|UniProtKB=P26641	Elongation factor 1-gamma;EEF1G;ortholog	ELONGATION FACTOR 1-GAMMA (PTHR11260:SF420)
HUMAN|HGNC=3151|UniProtKB=P30084		Enoyl-CoA hydratase, mitochondrial;ECHS1;ortholog	ENOYL-COA HYDRATASE, MITOCHONDRIAL (PTHR11941:SF111)
HUMAN|HGNC=3594|UniProtKB=P49327	HUMAN|HGNC=3594|UniProtKB=P49327	Fatty acid synthase;FASN;ortholog	FATTY ACID SYNTHASE (PTHR43775:SF2)
HUMAN|HGNC=1063|UniProtKB=P30043	HUMAN|HGNC=1063|UniProtKB=P30043	Flavin reductase (NADPH);BLVRB;ortholog	FLAVIN REDUCTASE (NADPH) (PTHR43355:SF2)
HUMAN|HGNC=15968|UniProtKB=Q8TB36		Ganglioside-induced differentiation-associated protein 1;GDAP1;ortholog	GANGLIOSIDE-INDUCED DIFFERENTIATION-ASSOCIATED PROTEIN 1 (PTHR11260:SF391)
HUMAN|HGNC=4335|UniProtKB=P00367		Glutamate dehydrogenase 1, mitochondrial;GLUD1;ortholog	GLUTAMATE DEHYDROGENASE 1, MITOCHONDRIAL (PTHR11606:SF17)
	HUMAN|HGNC=15987|UniProtKB=O76003	Glutaredoxin-3;GLRX3;ortholog	GLUTAREDOXIN-3 (PTHR10293:SF46)
HUMAN|HGNC=4570|UniProtKB=Q9UBQ7		Glyoxylate reductase/hydroxypyruvate reductase;GRHPR;ortholog	GLYOXYLATE REDUCTASE/HYDROXYPYRUVATE REDUCTASE (PTHR10996:SF172)
HUMAN|HGNC=4799|UniProtKB=Q16836		Hydroxyacyl-coenzyme A dehydrogenase, mitochondrial;HADH;ortholog	HYDROXYACYL-COENZYME A DEHYDROGENASE, MITOCHONDRIAL (PTHR43561:SF3)
HUMAN|HGNC=5384|UniProtKB=P50213	HUMAN|HGNC=5384|UniProtKB=P50213	Isocitrate dehydrogenase [NAD] subunit alpha, mitochondrial;IDH3A;ortholog	ISOCITRATE DEHYDROGENASE [NAD] SUBUNIT ALPHA, MITOCHONDRIAL (PTHR11835:SF58)
HUMAN|HGNC=5385|UniProtKB=O43837		Isocitrate dehydrogenase [NAD] subunit beta, mitochondrial;IDH3B;ortholog	ISOCITRATE DEHYDROGENASE [NAD] SUBUNIT BETA, MITOCHONDRIAL (PTHR11835:SF60)
HUMAN|HGNC=6535|UniProtKB=P00338		L-lactate dehydrogenase A chain;LDHA;ortholog	L-LACTATE DEHYDROGENASE A CHAIN (PTHR43128:SF4)
HUMAN|HGNC=6541|UniProtKB=P07195	HUMAN|HGNC=6541|UniProtKB=P07195	L-lactate dehydrogenase B chain;LDHB;ortholog	L-LACTATE DEHYDROGENASE B CHAIN (PTHR43128:SF5)
HUMAN|HGNC=6970|UniProtKB=P40925	HUMAN|HGNC=6970|UniProtKB=P40925	Malate dehydrogenase, cytoplasmic;MDH1;ortholog	MALATE DEHYDROGENASE, CYTOPLASMIC (PTHR23382:SF10)
HUMAN|HGNC=6971|UniProtKB=P40926	HUMAN|HGNC=6971|UniProtKB=P40926	Malate dehydrogenase, mitochondrial;MDH2;ortholog	MALATE DEHYDROGENASE, MITOCHONDRIAL (PTHR11540:SF35)
HUMAN|HGNC=890|UniProtKB=Q13825		Methylglutaconyl-CoA hydratase, mitochondrial;AUH;ortholog	METHYLGLUTACONYL-COA HYDRATASE, MITOCHONDRIAL (PTHR11941:SF104)

HUMAN|HGNC=6984|UniProtKB=P23368		NAD-dependent malic enzyme, mitochondrial;ME2;ortholog	NAD-DEPENDENT MALIC ENZYME, MITOCHONDRIAL (PTHR23406:SF50)
	HUMAN|HGNC=7863|UniProtKB=Q13423	NAD(P) transhydrogenase, mitochondrial;NNT;ortholog	NAD(P) TRANSHYDROGENASE, MITOCHONDRIAL (PTHR10160:SF28)
HUMAN|HGNC=17194|UniProtKB=Q9P0J0		NADH dehydrogenase [ubiquinone] 1 alpha subcomplex subunit 13;NDUFA13;ortholog	NADH DEHYDROGENASE [UBIQUINONE] 1 ALPHA SUBCOMPLEX SUBUNIT 13 (PTHR12966:SF1)
HUMAN|HGNC=7688|UniProtKB=Q16718		NADH dehydrogenase [ubiquinone] 1 alpha subcomplex subunit 5;NDUFA5;ortholog	NADH DEHYDROGENASE [UBIQUINONE] 1 ALPHA SUBCOMPLEX SUBUNIT 5 (PTHR12653:SF1)
HUMAN|HGNC=7692|UniProtKB=P51970	HUMAN|HGNC=7692|UniProtKB=P51970	NADH dehydrogenase [ubiquinone] 1 alpha subcomplex subunit 8;NDUFA8;ortholog	NADH DEHYDROGENASE [UBIQUINONE] 1 ALPHA SUBCOMPLEX SUBUNIT 8 (PTHR13344:SF1)
HUMAN|HGNC=7693|UniProtKB=Q16795	HUMAN|HGNC=7693|UniProtKB=Q16795	NADH dehydrogenase [ubiquinone] 1 alpha subcomplex subunit 9, mitochondrial;NDUFA9;ortholog	NADH DEHYDROGENASE [UBIQUINONE] 1 ALPHA SUBCOMPLEX SUBUNIT 9, MITOCHONDRIAL (PTHR12126:SF9)
	HUMAN|HGNC=7698|UniProtKB=O43676	NADH dehydrogenase [ubiquinone] 1 beta subcomplex subunit 3;NDUFB3;ortholog	NADH DEHYDROGENASE [UBIQUINONE] 1 BETA SUBCOMPLEX SUBUNIT 3 (PTHR15082:SF4)
	HUMAN|HGNC=7702|UniProtKB=P17568	NADH dehydrogenase [ubiquinone] 1 beta subcomplex subunit 7;NDUFB7;ortholog	NADH DEHYDROGENASE [UBIQUINONE] 1 BETA SUBCOMPLEX SUBUNIT 7 (PTHR20900:SF1)
HUMAN|HGNC=7717|UniProtKB=P19404	HUMAN|HGNC=7717|UniProtKB=P19404	NADH dehydrogenase [ubiquinone] flavoprotein 2, mitochondrial;NDUFV2;ortholog	NADH DEHYDROGENASE [UBIQUINONE] FLAVOPROTEIN 2, MITOCHONDRIAL (PTHR10371:SF8)
HUMAN|HGNC=7713|UniProtKB=O75380	HUMAN|HGNC=7713|UniProtKB=O75380	NADH dehydrogenase [ubiquinone] iron-sulfur protein 6, mitochondrial;NDUFS6;ortholog	NADH DEHYDROGENASE [UBIQUINONE] IRON-SULFUR PROTEIN 6, MITOCHONDRIAL (PTHR13156:SF1)
	HUMAN|HGNC=7714|UniProtKB=O75251	NADH dehydrogenase [ubiquinone] iron-sulfur protein 7, mitochondrial;NDUFS7;ortholog	NADH DEHYDROGENASE [UBIQUINONE] IRON-SULFUR PROTEIN 7, MITOCHONDRIAL (PTHR11995:SF24)
HUMAN|HGNC=13397|UniProtKB=Q9UHQ9	HUMAN|HGNC=13397|UniProtKB=Q9UHQ9	NADH-cytochrome b5 reductase 1;CYB5R1;ortholog	NADH-CYTOCHROME B5 REDUCTASE 1 (PTHR19370:SF138)
HUMAN|HGNC=7707|UniProtKB=P28331		NADH-ubiquinone oxidoreductase 75 kDa subunit, mitochondrial;NDUFS1;ortholog	NADH-UBIQUINONE OXIDOREDUCTASE 75 KDA SUBUNIT, MITOCHONDRIAL (PTHR11615:SF194)
HUMAN|HGNC=9353|UniProtKB=P32119	HUMAN|HGNC=9353|UniProtKB=P32119	Peroxiredoxin-2;PRDX2;ortholog	PEROXIREDOXIN-2 (PTHR10681:SF136)
HUMAN|HGNC=16753|UniProtKB=P30041	HUMAN|HGNC=16753|UniProtKB=P30041	Peroxiredoxin-6;PRDX6;ortholog	PEROXIREDOXIN-6 (PTHR43503:SF1)
HUMAN|HGNC=17822|UniProtKB=Q9H7Z7		Prostaglandin E synthase 2;PTGES2;ortholog	PROSTAGLANDIN E SYNTHASE 2 (PTHR12782:SF9)
	HUMAN|HGNC=9280|UniProtKB=P50336	Protoporphyrinogen oxidase;PPOX;ortholog	PROTOPORPHYRINOGEN OXIDASE (PTHR42923:SF1)
	HUMAN|HGNC=33782|UniProtKB=Q6GMV3	Putative peptidyl-tRNA hydrolase PTRHD1;PTRHD1;ortholog	PEPTIDYL-TRNA HYDROLASE PTRHD1-RELATED (PTHR11717:SF25)
HUMAN|HGNC=2419|UniProtKB=Q08257		Quinone oxidoreductase;CRYZ;ortholog	QUINONE OXIDOREDUCTASE (PTHR11695:SF632)
HUMAN|HGNC=17964|UniProtKB=Q8TC12	HUMAN|HGNC=17964|UniProtKB=Q8TC12	Retinol dehydrogenase 11;RDH11;ortholog	RETINOL DEHYDROGENASE 11 (PTHR24320:SF131)
HUMAN|HGNC=10680|UniProtKB=P31040		Succinate dehydrogenase [ubiquinone] flavoprotein subunit, mitochondrial;SDHA;ortholog	SUCCINATE DEHYDROGENASE [UBIQUINONE] FLAVOPROTEIN SUBUNIT, MITOCHONDRIAL (PTHR11632:SF63)
HUMAN|HGNC=10681|UniProtKB=P21912	HUMAN|HGNC=10681|UniProtKB=P21912	Succinate dehydrogenase [ubiquinone] iron-sulfur subunit, mitochondrial;SDHB;ortholog	SUCCINATE DEHYDROGENASE [UBIQUINONE] IRON-SULFUR SUBUNIT, MITOCHONDRIAL (PTHR11921:SF31)
HUMAN|HGNC=408|UniProtKB=P51649		Succinate-semialdehyde dehydrogenase, mitochondrial;ALDH5A1;ortholog	SUCCINATE-SEMIALDEHYDE DEHYDROGENASE, MITOCHONDRIAL (PTHR43353:SF7)
HUMAN|HGNC=11179|UniProtKB=P00441		Superoxide dismutase [Cu-Zn];SOD1;ortholog	SUPEROXIDE DISMUTASE [CU-ZN] (PTHR10003:SF50)
HUMAN|HGNC=16919|UniProtKB=Q99536	HUMAN|HGNC=16919|UniProtKB=Q99536	Synaptic vesicle membrane protein VAT-1 homolog;VAT1;ortholog	SYNAPTIC VESICLE MEMBRANE PROTEIN VAT-1 HOMOLOG (PTHR11695:SF616)
HUMAN|HGNC=19691|UniProtKB=Q9BV79		Trans-2-enoyl-CoA reductase, mitochondrial;MECR;ortholog	TRANS-2-ENOYL-COA REDUCTASE, MITOCHONDRIAL (PTHR11695:SF624)
HUMAN|HGNC=11834|UniProtKB=P29401	HUMAN|HGNC=11834|UniProtKB=P29401	Transketolase;TKT;ortholog	TRANSKETOLASE (PTHR43195:SF3)

HUMAN|HGNC=4801|UniProtKB=P40939	HUMAN|HGNC=4801|UniProtKB=P40939	Trifunctional enzyme subunit alpha, mitochondrial;HADHA;ortholog	TRIFUNCTIONAL ENZYME SUBUNIT ALPHA, MITOCHONDRIAL (PTHR43612:SF3)
HUMAN|HGNC=18646|UniProtKB=Q53GQ0	HUMAN|HGNC=18646|UniProtKB=Q53GQ0	Very-long-chain 3-oxoacyl-CoA reductase;HSD17B12;ortholog	VERY-LONG-CHAIN 3-OXOACYL-COA REDUCTASE (PTHR24322:SF672)
**receptor (PC00197)**			
	HUMAN|HGNC=5273|UniProtKB=P98160	Basement membrane-specific heparan sulfate proteoglycan core protein;HSPG2;ortholog	BASEMENT MEMBRANE-SPECIFIC HEPARAN SULFATE PROTEOGLYCAN CORE PROTEIN (PTHR10574:SF343)
HUMAN|HGNC=8011|UniProtKB=P78357	HUMAN|HGNC=8011|UniProtKB=P78357	Contactin-associated protein 1;CNTNAP1;ortholog	CONTACTIN-ASSOCIATED PROTEIN 1 (PTHR10127:SF740)
HUMAN|HGNC=12825|UniProtKB=O14980	HUMAN|HGNC=12825|UniProtKB=O14980	Exportin-1;XPO1;ortholog	EXPORTIN-1 (PTHR11223:SF9)
	HUMAN|HGNC=6487|UniProtKB=P55268	Laminin subunit beta-2;LAMB2;ortholog	LAMININ SUBUNIT BETA-2 (PTHR10574:SF346)
	HUMAN|HGNC=6492|UniProtKB=P11047	Laminin subunit gamma-1;LAMC1;ortholog	LAMININ SUBUNIT GAMMA-1 (PTHR10574:SF309)
HUMAN|HGNC=15714|UniProtKB=P42704	HUMAN|HGNC=15714|UniProtKB=P42704	Leucine-rich PPR motif-containing protein, mitochondrial;LRPPRC;ortholog	LEUCINE-RICH PPR MOTIF-CONTAINING PROTEIN, MITOCHONDRIAL (PTHR24015:SF1304)
HUMAN|HGNC=6705|UniProtKB=Q13449		Limbic system-associated membrane protein;LSAMP;ortholog	LIMBIC SYSTEM-ASSOCIATED MEMBRANE PROTEIN (PTHR42757:SF13)
	HUMAN|HGNC=1665|UniProtKB=Q14108	Lysosome membrane protein 2;SCARB2;ortholog	LYSOSOME MEMBRANE PROTEIN 2 (PTHR11923:SF85)
HUMAN|HGNC=16090|UniProtKB=O00264		Membrane-associated progesterone receptor component 1;PGRMC1;ortholog	MEMBRANE-ASSOCIATED PROGESTERONE RECEPTOR COMPONENT 1 (PTHR10281:SF46)
HUMAN|HGNC=16089|UniProtKB=O15173		Membrane-associated progesterone receptor component 2;PGRMC2;ortholog	MEMBRANE-ASSOCIATED PROGESTERONE RECEPTOR COMPONENT 2 (PTHR10281:SF59)
HUMAN|HGNC=18002|UniProtKB=Q9NS69		Mitochondrial import receptor subunit TOM22 homolog;TOMM22;ortholog	MITOCHONDRIAL IMPORT RECEPTOR SUBUNIT TOM22 HOMOLOG (PTHR12504:SF3)
HUMAN|HGNC=8135|UniProtKB=P23515	HUMAN|HGNC=8135|UniProtKB=P23515	Oligodendrocyte-myelin glycoprotein;OMG;ortholog	OLIGODENDROCYTE-MYELIN GLYCOPROTEIN (PTHR24373:SF208)
	HUMAN|HGNC=25655|UniProtKB=Q9NUJ3	T-complex protein 11-like protein 1;TCP11L1;ortholog	T-COMPLEX PROTEIN 11-LIKE PROTEIN 1 (PTHR12832:SF22)
**signaling molecule (PC00207)**			
HUMAN|HGNC=13518|UniProtKB=Q9Y696		Chloride intracellular channel protein 4;CLIC4;ortholog	CHLORIDE INTRACELLULAR CHANNEL PROTEIN 4 (PTHR11260:SF443)
HUMAN|HGNC=8011|UniProtKB=P78357	HUMAN|HGNC=8011|UniProtKB=P78357	Contactin-associated protein 1;CNTNAP1;ortholog	CONTACTIN-ASSOCIATED PROTEIN 1 (PTHR10127:SF740)
HUMAN|HGNC=3213|UniProtKB=P26641	HUMAN|HGNC=3213|UniProtKB=P26641	Elongation factor 1-gamma;EEF1G;ortholog	ELONGATION FACTOR 1-GAMMA (PTHR11260:SF420)
	HUMAN|HGNC=16286|UniProtKB=Q92556	Engulfment and cell motility protein 1;ELMO1;ortholog	ENGULFMENT AND CELL MOTILITY PROTEIN 1 (PTHR12771:SF26)
HUMAN|HGNC=3662|UniProtKB=P02675	HUMAN|HGNC=3662|UniProtKB=P02675	Fibrinogen beta chain;FGB;ortholog	FIBRINOGEN BETA CHAIN (PTHR19143:SF313)
HUMAN|HGNC=6561|UniProtKB=P09382		Galectin-1;LGALS1;ortholog	GALECTIN-1 (PTHR11346:SF131)
HUMAN|HGNC=11141|UniProtKB=O76070		Gamma-synuclein;SNCG;ortholog	GAMMA-SYNUCLEIN (PTHR13820:SF14)
HUMAN|HGNC=15968|UniProtKB=Q8TB36		Ganglioside-induced differentiation-associated protein 1;GDAP1;ortholog	GANGLIOSIDE-INDUCED DIFFERENTIATION-ASSOCIATED PROTEIN 1 (PTHR11260:SF391)
HUMAN|HGNC=4413|UniProtKB=P36915		Guanine nucleotide-binding protein-like 1;GNL1;ortholog	GUANINE NUCLEOTIDE-BINDING PROTEIN-LIKE 1 (PTHR11089:SF55)
HUMAN|HGNC=26361|UniProtKB=Q14CZ8		Hepatocyte cell adhesion molecule;HEPACAM;ortholog	HEPATOCYTE CELL ADHESION MOLECULE (PTHR12080:SF66)
HUMAN|HGNC=5348|UniProtKB=Q9UMF0	HUMAN|HGNC=5348|UniProtKB=Q9UMF0	Intercellular adhesion molecule 5;ICAM5;ortholog	INTERCELLULAR ADHESION MOLECULE 5 (PTHR13771:SF15)
HUMAN|HGNC=16090|UniProtKB=O00264		Membrane-associated progesterone receptor component 1;PGRMC1;ortholog	MEMBRANE-ASSOCIATED PROGESTERONE RECEPTOR COMPONENT 1 (PTHR10281:SF46)

HUMAN|HGNC=16089|UniProtKB=O15173		Membrane-associated progesterone receptor component 2;PGRMC2;ortholog	MEMBRANE-ASSOCIATED PROGESTERONE RECEPTOR COMPONENT 2 (PTHR10281:SF59)
HUMAN|HGNC=7126|UniProtKB=Q15773		Myeloid leukemia factor 2;MLF2;ortholog	MYELOID LEUKEMIA FACTOR 2 (PTHR13105:SF11)
	HUMAN|HGNC=30092|UniProtKB=P43490	Nicotinamide phosphoribosyltransferase;NAMPT;ortholog	NICOTINAMIDE PHOSPHORIBOSYLTRANSFERASE (PTHR43816:SF1)
HUMAN|HGNC=29180|UniProtKB=Q9UPV7	HUMAN|HGNC=29180|UniProtKB=Q9UPV7	Protein KIAA1045;KIAA1045;ortholog	PROTEIN KIAA1045 (PTHR10891:SF706)
HUMAN|HGNC=11953|UniProtKB=Q92752		Tenascin-R;TNR;ortholog	TENASCIN-R (PTHR19143:SF307)
HUMAN|HGNC=9662|UniProtKB=P78324		Tyrosine-protein phosphatase non-receptor type substrate 1;SIRPA;ortholog	TYROSINE-PROTEIN PHOSPHATASE NON-RECEPTOR TYPE SUBSTRATE 1 (PTHR19971:SF21)
**storage protein (PC00210)**			
HUMAN|HGNC=3976|UniProtKB=P02794	HUMAN|HGNC=3976|UniProtKB=P02794	Ferritin heavy chain;FTH1;ortholog	FERRITIN HEAVY CHAIN (PTHR11431:SF53)
HUMAN|HGNC=3999|UniProtKB=P02792	HUMAN|HGNC=3999|UniProtKB=P02792	Ferritin light chain;FTL;ortholog	FERRITIN LIGHT CHAIN (PTHR11431:SF66)
**structural protein (PC00211)**			
HUMAN|HGNC=6057|UniProtKB=Q16352	HUMAN|HGNC=6057|UniProtKB=Q16352	Alpha-internexin;INA;ortholog	ALPHA-INTERNEXIN (PTHR23239:SF273)
HUMAN|HGNC=2770|UniProtKB=P17661	HUMAN|HGNC=2770|UniProtKB=P17661	Desmin;DES;ortholog	DESMIN (PTHR23239:SF307)
HUMAN|HGNC=4235|UniProtKB=P14136	HUMAN|HGNC=4235|UniProtKB=P14136	Glial fibrillary acidic protein;GFAP;ortholog	GLIAL FIBRILLARY ACIDIC PROTEIN (PTHR23239:SF249)
HUMAN|HGNC=6413|UniProtKB=P13645	HUMAN|HGNC=6413|UniProtKB=P13645	Keratin, type I cytoskeletal 10;KRT10;ortholog	KERATIN, TYPE I CYTOSKELETAL 10 (PTHR23239:SF308)
	HUMAN|HGNC=6416|UniProtKB=P02533	Keratin, type I cytoskeletal 14;KRT14;ortholog	KERATIN, TYPE I CYTOSKELETAL 14 (PTHR23239:SF272)
	HUMAN|HGNC=6421|UniProtKB=P19012	Keratin, type I cytoskeletal 15;KRT15;ortholog	KERATIN, TYPE I CYTOSKELETAL 15 (PTHR23239:SF239)
	HUMAN|HGNC=6423|UniProtKB=P08779	Keratin, type I cytoskeletal 16;KRT16;ortholog	KERATIN, TYPE I CYTOSKELETAL 16 (PTHR23239:SF263)
HUMAN|HGNC=6447|UniProtKB=P35527	HUMAN|HGNC=6447|UniProtKB=P35527	Keratin, type I cytoskeletal 9;KRT9;ortholog	KERATIN, TYPE I CYTOSKELETAL 9 (PTHR23239:SF281)
HUMAN|HGNC=6412|UniProtKB=P04264	HUMAN|HGNC=6412|UniProtKB=P04264	Keratin, type II cytoskeletal 1;KRT1;ortholog	KERATIN, TYPE II CYTOSKELETAL 1 (PTHR23239:SF236)
HUMAN|HGNC=6439|UniProtKB=P35908	HUMAN|HGNC=6439|UniProtKB=P35908	Keratin, type II cytoskeletal 2 epidermal;KRT2;ortholog	KERATIN, TYPE II CYTOSKELETAL 2 EPIDERMAL (PTHR23239:SF228)
	HUMAN|HGNC=6440|UniProtKB=P12035	Keratin, type II cytoskeletal 3;KRT3;ortholog	KERATIN, TYPE II CYTOSKELETAL 3 (PTHR23239:SF231)
	HUMAN|HGNC=6442|UniProtKB=P13647	Keratin, type II cytoskeletal 5;KRT5;ortholog	KERATIN, TYPE II CYTOSKELETAL 5 (PTHR23239:SF279)
	HUMAN|HGNC=6443|UniProtKB=P02538	Keratin, type II cytoskeletal 6A;KRT6A;ortholog	KERATIN, TYPE II CYTOSKELETAL 6A (PTHR23239:SF232)
	HUMAN|HGNC=28929|UniProtKB=Q7RTS7	Keratin, type II cytoskeletal 74;KRT74;ortholog	KERATIN, TYPE II CYTOSKELETAL 74 (PTHR23239:SF328)
HUMAN|HGNC=6637|UniProtKB=P20700		Lamin-B1;LMNB1;ortholog	LAMIN-B1 (PTHR23239:SF316)
HUMAN|HGNC=6638|UniProtKB=Q03252	HUMAN|HGNC=6638|UniProtKB=Q03252	Lamin-B2;LMNB2;ortholog	LAMIN-B2 (PTHR23239:SF280)
HUMAN|HGNC=9086|UniProtKB=P60201	HUMAN|HGNC=9086|UniProtKB=P60201	Myelin proteolipid protein;PLP1;ortholog	MYELIN PROTEOLIPID PROTEIN (PTHR11683:SF18)
HUMAN|HGNC=7739|UniProtKB=P07196	HUMAN|HGNC=7739|UniProtKB=P07196	Neurofilament light polypeptide;NEFL;ortholog	NEUROFILAMENT LIGHT POLYPEPTIDE (PTHR23239:SF242)
HUMAN|HGNC=9461|UniProtKB=P41219		Peripherin;PRPH;ortholog	PERIPHERIN (PTHR23239:SF288)
	HUMAN|HGNC=12692|UniProtKB=P08670	Vimentin;VIM;ortholog	VIMENTIN (PTHR23239:SF258)
**transcription factor (PC00218)**			
	HUMAN|HGNC=8550|UniProtKB=Q9UQ80	Proliferation-associated protein 2G4;PA2G4;ortholog	PROLIFERATION-ASSOCIATED PROTEIN 2G4 (PTHR10804:SF123)

	HUMAN|HGNC=10074|UniProtKB=P13489	Ribonuclease inhibitor;RNH1;ortholog	RIBONUCLEASE INHIBITOR (PTHR24112:SF52)
	HUMAN|HGNC=11362|UniProtKB=P42224	Signal transducer and activator of transcription 1-alpha/beta;STAT1;ortholog	SIGNAL TRANSDUCER AND ACTIVATOR OF TRANSCRIPTION 1-ALPHA/BETA (PTHR11801:SF58)
HUMAN|HGNC=9701|UniProtKB=Q00577	HUMAN|HGNC=9701|UniProtKB=Q00577	Transcriptional activator protein Pur-alpha;PURA;ortholog	TRANSCRIPTIONAL ACTIVATOR PROTEIN PUR-ALPHA (PTHR12611:SF9)
**transfer/carrier protein (PC00219)**			
HUMAN|HGNC=2690|UniProtKB=P07108		Acyl-CoA-binding protein;DBI;ortholog	ACYL-COA-BINDING PROTEIN (PTHR23310:SF74)
HUMAN|HGNC=10990|UniProtKB=P12235	HUMAN|HGNC=10990|UniProtKB=P12235	ADP/ATP translocase 1;SLC25A4;ortholog	ADP/ATP TRANSLOCASE 1 (PTHR24089:SF506)
HUMAN|HGNC=10991|UniProtKB=P05141	HUMAN|HGNC=10991|UniProtKB=P05141	ADP/ATP translocase 2;SLC25A5;ortholog	ADP/ATP TRANSLOCASE 2 (PTHR24089:SF446)
HUMAN|HGNC=10992|UniProtKB=P12236	HUMAN|HGNC=10992|UniProtKB=P12236	ADP/ATP translocase 3;SLC25A6;ortholog	ADP/ATP TRANSLOCASE 3 (PTHR24089:SF411)
	HUMAN|HGNC=619|UniProtKB=Q9BQE5	Apolipoprotein L2;APOL2;ortholog	APOLIPOPROTEIN L1-RELATED (PTHR14096:SF49)
HUMAN|HGNC=10982|UniProtKB=O75746	HUMAN|HGNC=10982|UniProtKB=O75746	Calcium-binding mitochondrial carrier protein Aralar1;SLC25A12;ortholog	CALCIUM-BINDING MITOCHONDRIAL CARRIER PROTEIN ARALAR1 (PTHR24089:SF503)
	HUMAN|HGNC=10983|UniProtKB=Q9UJS0	Calcium-binding mitochondrial carrier protein Aralar2;SLC25A13;ortholog	CALCIUM-BINDING MITOCHONDRIAL CARRIER PROTEIN ARALAR2 (PTHR24089:SF428)
HUMAN|HGNC=23046|UniProtKB=Q5SYC1		Clavesin-2;CLVS2;ortholog	CLAVESIN-2 (PTHR10174:SF176)
HUMAN|HGNC=8011|UniProtKB=P78357	HUMAN|HGNC=8011|UniProtKB=P78357	Contactin-associated protein 1;CNTNAP1;ortholog	CONTACTIN-ASSOCIATED PROTEIN 1 (PTHR10127:SF740)
	HUMAN|HGNC=16906|UniProtKB=Q9Y2Q3	Glutathione S-transferase kappa 1;GSTK1;ortholog	GLUTATHIONE S-TRANSFERASE KAPPA 1 (PTHR42943:SF1)
HUMAN|HGNC=4823|UniProtKB=P69905		Hemoglobin subunit alpha;HBA1;ortholog	HEMOGLOBIN SUBUNIT ALPHA (PTHR11442:SF64)
HUMAN|HGNC=4824|UniProtKB=P69905		Hemoglobin subunit alpha;HBA1;ortholog	HEMOGLOBIN SUBUNIT ALPHA (PTHR11442:SF61)
HUMAN|HGNC=4827|UniProtKB=P68871	HUMAN|HGNC=4827|UniProtKB=P68871	Hemoglobin subunit beta;HBB;ortholog	HEMOGLOBIN SUBUNIT BETA (PTHR11442:SF72)
	HUMAN|HGNC=6396|UniProtKB=O00505	Importin subunit alpha-4;KPNA3;ortholog	IMPORTIN SUBUNIT ALPHA-4 (PTHR23316:SF41)
	HUMAN|HGNC=6394|UniProtKB=P52294	Importin subunit alpha-5;KPNA1;ortholog	IMPORTIN SUBUNIT ALPHA-5 (PTHR23316:SF40)
	HUMAN|HGNC=6399|UniProtKB=O60684	Importin subunit alpha-7;KPNA6;ortholog	IMPORTIN SUBUNIT ALPHA-7 (PTHR23316:SF46)
HUMAN|HGNC=6400|UniProtKB=Q14974	HUMAN|HGNC=6400|UniProtKB=Q14974	Importin subunit beta-1;KPNB1;ortholog	IMPORTIN SUBUNIT BETA-1 (PTHR10527:SF32)
	HUMAN|HGNC=17587|UniProtKB=Q9Y6C9	Mitochondrial carrier homolog 2;MTCH2;ortholog	MITOCHONDRIAL CARRIER HOMOLOG 2 (PTHR10780:SF23)
	HUMAN|HGNC=19954|UniProtKB=Q9H936	Mitochondrial glutamate carrier 1;SLC25A22;ortholog	MITOCHONDRIAL GLUTAMATE CARRIER 1 (PTHR24089:SF598)
	HUMAN|HGNC=10988|UniProtKB=Q9H1K4	Mitochondrial glutamate carrier 2;SLC25A18;ortholog	MITOCHONDRIAL GLUTAMATE CARRIER 2 (PTHR24089:SF482)
HUMAN|HGNC=18002|UniProtKB=Q9NS69		Mitochondrial import receptor subunit TOM22 homolog;TOMM22;ortholog	MITOCHONDRIAL IMPORT RECEPTOR SUBUNIT TOM22 HOMOLOG (PTHR12504:SF3)
HUMAN|HGNC=9592|UniProtKB=P41222	HUMAN|HGNC=9592|UniProtKB=P41222	Prostaglandin-H2 D-isomerase;PTGDS;ortholog	PROSTAGLANDIN-H2 D-ISOMERASE (PTHR11430:SF114)
HUMAN|HGNC=9393|UniProtKB=P17252		Protein kinase C alpha type;PRKCA;ortholog	PROTEIN KINASE C ALPHA TYPE (PTHR24356:SF270)
HUMAN|HGNC=9395|UniProtKB=P05771	HUMAN|HGNC=9395|UniProtKB=P05771	Protein kinase C beta type;PRKCB;ortholog	PROTEIN KINASE C BETA TYPE (PTHR24356:SF244)
	HUMAN|HGNC=9401|UniProtKB=Q02156	Protein kinase C epsilon type;PRKCE;ortholog	PROTEIN KINASE C EPSILON TYPE (PTHR24356:SF275)
	HUMAN|HGNC=49897|UniProtKB=Q6A1A2	Putative 3-phosphoinositide-dependent protein kinase 2;PDPK2P;ortholog	3-PHOSPHOINOSITIDE-DEPENDENT PROTEIN KINASE 1-RELATED (PTHR24356:SF290)
HUMAN|HGNC=21332|UniProtKB=Q86UX6		Serine/threonine-protein kinase 32C;STK32C;ortholog	SERINE/THREONINE-PROTEIN KINASE 32C (PTHR24356:SF232)

HUMAN|HGNC=11740|UniProtKB=P02787		Serotransferrin;TF;ortholog	SEROTRANSFERRIN (PTHR11485:SF35)
HUMAN|HGNC=399|UniProtKB=P02768		Serum albumin;ALB;ortholog	SERUM ALBUMIN (PTHR11385:SF18)
HUMAN|HGNC=16085|UniProtKB=Q9H9B4	HUMAN|HGNC=16085|UniProtKB=Q9H9B4	Sideroflexin-1;SFXN1;ortholog	SIDEROFLEXIN-1 (PTHR11153:SF32)
	HUMAN|HGNC=16073|UniProtKB=Q8TD22	Sideroflexin-5;SFXN5;ortholog	SIDEROFLEXIN-5 (PTHR11153:SF24)
	HUMAN|HGNC=30661|UniProtKB=Q9UBT2	SUMO-activating enzyme subunit 2;UBA2;ortholog	SUMO-ACTIVATING ENZYME SUBUNIT 2 (PTHR10953:SF166)
	HUMAN|HGNC=19666|UniProtKB=Q8NFX7	Syntaxin-binding protein 6;STXBP6;ortholog	SYNTAXIN-BINDING PROTEIN 6 (PTHR16092:SF24)
HUMAN|HGNC=24878|UniProtKB=Q9BVK6		Transmembrane emp24 domain-containing protein 9;TMED9;ortholog	TRANSMEMBRANE EMP24 DOMAIN-CONTAINING PROTEIN 9 (PTHR22811:SF101)
HUMAN|HGNC=10979|UniProtKB=P53007	HUMAN|HGNC=10979|UniProtKB=P53007	Tricarboxylate transport protein, mitochondrial;SLC25A1;ortholog	TRICARBOXYLATE TRANSPORT PROTEIN, MITOCHONDRIAL (PTHR24089:SF459)
	HUMAN|HGNC=25581|UniProtKB=A0AVT1	Ubiquitin-like modifier-activating enzyme 6;UBA6;ortholog	UBIQUITIN-LIKE MODIFIER-ACTIVATING ENZYME 6 (PTHR10953:SF180)

transferase (PC00220)

HUMAN|HGNC=8811|UniProtKB=Q15120	HUMAN|HGNC=8811|UniProtKB=Q15120	[Pyruvate dehydrogenase (acetyl-transferring)] kinase isozyme 3, mitochondrial;PDK3;ortholog	[PYRUVATE DEHYDROGENASE (ACETYL-TRANSFERRING)] KINASE ISOZYME 3, MITOCHONDRIAL (PTHR11947:SF31)
HUMAN|HGNC=23|UniProtKB=P80404		4-aminobutyrate aminotransferase, mitochondrial;ABAT;ortholog	4-AMINOBUTYRATE AMINOTRANSFERASE, MITOCHONDRIAL (PTHR43206:SF1)
HUMAN|HGNC=94|UniProtKB=Q9BWD1		Acetyl-CoA acetyltransferase, cytosolic;ACAT2;ortholog	ACETYL-COA ACETYLTRANSFERASE, CYTOSOLIC (PTHR18919:SF115)
HUMAN|HGNC=93|UniProtKB=P24752		Acetyl-CoA acetyltransferase, mitochondrial;ACAT1;ortholog	ACETYL-COA ACETYLTRANSFERASE, MITOCHONDRIAL (PTHR18919:SF114)
HUMAN|HGNC=363|UniProtKB=P27144	HUMAN|HGNC=363|UniProtKB=P27144	Adenylate kinase 4, mitochondrial;AK4;ortholog	ADENYLATE KINASE 4, MITOCHONDRIAL (PTHR23359:SF119)
HUMAN|HGNC=361|UniProtKB=P00568	HUMAN|HGNC=361|UniProtKB=P00568	Adenylate kinase isoenzyme 1;AK1;ortholog	ADENYLATE KINASE ISOENZYME 1 (PTHR23359:SF135)
HUMAN|HGNC=8876|UniProtKB=P17858	HUMAN|HGNC=8876|UniProtKB=P17858	ATP-dependent 6-phosphofructokinase, liver type;PFKL;ortholog	ATP-DEPENDENT 6-PHOSPHOFRUCTOKINASE, LIVER TYPE (PTHR13697:SF35)
HUMAN|HGNC=8877|UniProtKB=P08237	HUMAN|HGNC=8877|UniProtKB=P08237	ATP-dependent 6-phosphofructokinase, muscle type;PFKM;ortholog	ATP-DEPENDENT 6-PHOSPHOFRUCTOKINASE, MUSCLE TYPE (PTHR13697:SF38)
HUMAN|HGNC=8878|UniProtKB=Q01813	HUMAN|HGNC=8878|UniProtKB=Q01813	ATP-dependent 6-phosphofructokinase, platelet type;PFKP;ortholog	ATP-DEPENDENT 6-PHOSPHOFRUCTOKINASE, PLATELET TYPE (PTHR13697:SF48)
	HUMAN|HGNC=29932|UniProtKB=Q13057	Bifunctional coenzyme A synthase;COASY;ortholog	BIFUNCTIONAL COENZYME A SYNTHASE (PTHR10695:SF42)
HUMAN|HGNC=1469|UniProtKB=Q8N5S9	HUMAN|HGNC=1469|UniProtKB=Q8N5S9	Calcium/calmodulin-dependent protein kinase kinase 1;CAMKK1;ortholog	CALCIUM/CALMODULIN-DEPENDENT PROTEIN KINASE KINASE 1 (PTHR24347:SF330)
HUMAN|HGNC=1460|UniProtKB=Q9UQM7	HUMAN|HGNC=1460|UniProtKB=Q9UQM7	Calcium/calmodulin-dependent protein kinase type II subunit alpha;CAMK2A;ortholog	CALCIUM/CALMODULIN-DEPENDENT PROTEIN KINASE TYPE II SUBUNIT ALPHA (PTHR24347:SF318)
	HUMAN|HGNC=28204|UniProtKB=Q9BSD7	Cancer-related nucleoside-triphosphatase;NTPCR;ortholog	CANCER-RELATED NUCLEOSIDE-TRIPHOSPHATASE (PTHR43146:SF1)
HUMAN|HGNC=2330|UniProtKB=P23786		Carnitine O-palmitoyltransferase 2, mitochondrial;CPT2;ortholog	CARNITINE O-PALMITOYLTRANSFERASE 2, MITOCHONDRIAL (PTHR22589:SF96)
HUMAN|HGNC=13518|UniProtKB=Q9Y696		Chloride intracellular channel protein 4;CLIC4;ortholog	CHLORIDE INTRACELLULAR CHANNEL PROTEIN 4 (PTHR11260:SF443)
HUMAN|HGNC=1991|UniProtKB=P12277	HUMAN|HGNC=1991|UniProtKB=P12277	Creatine kinase B-type;CKB;ortholog	CREATINE KINASE B-TYPE (PTHR11547:SF41)
HUMAN|HGNC=1994|UniProtKB=P06732		Creatine kinase M-type;CKM;ortholog	CREATINE KINASE M-TYPE (PTHR11547:SF45)
HUMAN|HGNC=1995|UniProtKB=P12532	HUMAN|HGNC=1995|UniProtKB=P12532	Creatine kinase U-type, mitochondrial;CKMT1A;ortholog	CREATINE KINASE U-TYPE, MITOCHONDRIAL (PTHR11547:SF46)
HUMAN|HGNC=31736|UniProtKB=P12532	HUMAN|HGNC=31736|UniProtKB=P12532	Creatine kinase U-type, mitochondrial;CKMT1A;ortholog	CREATINE KINASE U-TYPE, MITOCHONDRIAL (PTHR11547:SF46)

	HUMAN|HGNC=8751|UniProtKB=Q07002	Cyclin-dependent kinase 18;CDK18;ortholog	CYCLIN-DEPENDENT KINASE 18 (PTHR24056:SF264)
HUMAN|HGNC=1774|UniProtKB=Q00535	HUMAN|HGNC=1774|UniProtKB=Q00535	Cyclin-dependent-like kinase 5;CDK5;ortholog	CYCLIN-DEPENDENT-LIKE KINASE 5 (PTHR24056:SF283)
	HUMAN|HGNC=2896|UniProtKB=P10515	Dihydrolipoyllysine-residue acetyltransferase component of pyruvate dehydrogenase complex, mitochondrial;DLAT;ortholog	DIHYDROLIPOYLLYSINE-RESIDUE ACETYLTRANSFERASE COMPONENT OF PYRUVATE DEHYDROGENASE COMPLEX, MITOCHONDRIAL (PTHR23151:SF73)
HUMAN|HGNC=2911|UniProtKB=P36957	HUMAN|HGNC=2911|UniProtKB=P36957	Dihydrolipoyllysine-residue succinyltransferase component of 2-oxoglutarate dehydrogenase complex, mitochondrial;DLST;ortholog	DIHYDROLIPOYLLYSINE-RESIDUE SUCCINYLTRANSFERASE COMPONENT OF 2-OXOGLUTARATE DEHYDROGENASE COMPLEX, MITOCHONDRIAL (PTHR43416:SF7)
HUMAN|HGNC=10381|UniProtKB=P04843		Dolichyl-diphosphooligosaccharide--protein glycosyltransferase subunit 1;RPN1;ortholog	DOLICHYL-DIPHOSPHOOLIGOSACCHARIDE--PROTEIN GLYCOSYLTRANSFERASE SUBUNIT 1 (PTHR21049:SF3)
HUMAN|HGNC=3213|UniProtKB=P26641	HUMAN|HGNC=3213|UniProtKB=P26641	Elongation factor 1-gamma;EEF1G;ortholog	ELONGATION FACTOR 1-GAMMA (PTHR11260:SF420)
HUMAN|HGNC=3151|UniProtKB=P30084		Enoyl-CoA hydratase, mitochondrial;ECHS1;ortholog	ENOYL-COA HYDRATASE, MITOCHONDRIAL (PTHR11941:SF111)
HUMAN|HGNC=3594|UniProtKB=P49327	HUMAN|HGNC=3594|UniProtKB=P49327	Fatty acid synthase;FASN;ortholog	FATTY ACID SYNTHASE (PTHR43775:SF2)
HUMAN|HGNC=15968|UniProtKB=Q8TB36		Ganglioside-induced differentiation-associated protein 1;GDAP1;ortholog	GANGLIOSIDE-INDUCED DIFFERENTIATION-ASSOCIATED PROTEIN 1 (PTHR11260:SF391)
HUMAN|HGNC=20898|UniProtKB=Q6PCE3		Glucose 1,6-bisphosphate synthase;PGM2L1;ortholog	GLUCOSE 1,6-BISPHOSPHATE SYNTHASE (PTHR22573:SF60)
HUMAN|HGNC=9723|UniProtKB=P11216	HUMAN|HGNC=9723|UniProtKB=P11216	Glycogen phosphorylase, brain form;PYGB;ortholog	GLYCOGEN PHOSPHORYLASE, BRAIN FORM (PTHR11468:SF19)
HUMAN|HGNC=9726|UniProtKB=P11217		Glycogen phosphorylase, muscle form;PYGM;ortholog	GLYCOGEN PHOSPHORYLASE, MUSCLE FORM (PTHR11468:SF18)
	HUMAN|HGNC=4617|UniProtKB=P49841	Glycogen synthase kinase-3 beta;GSK3B;ortholog	GLYCOGEN SYNTHASE KINASE-3 BETA (PTHR24057:SF26)
HUMAN|HGNC=19022|UniProtKB=O43301	HUMAN|HGNC=19022|UniProtKB=O43301	Heat shock 70 kDa protein 12A;HSPA12A;ortholog	HEAT SHOCK 70 KDA PROTEIN 12A (PTHR14187:SF63)
HUMAN|HGNC=26361|UniProtKB=Q14CZ8		Hepatocyte cell adhesion molecule;HEPACAM;ortholog	HEPATOCYTE CELL ADHESION MOLECULE (PTHR12080:SF66)
	HUMAN|HGNC=5007|UniProtKB=Q01581	Hydroxymethylglutaryl-CoA synthase, cytoplasmic;HMGCS1;ortholog	HYDROXYMETHYLGLUTARYL-COA SYNTHASE, CYTOPLASMIC (PTHR43323:SF6)
HUMAN|HGNC=5157|UniProtKB=P00492		Hypoxanthine-guanine phosphoribosyltransferase;HPRT1;ortholog	HYPOXANTHINE-GUANINE PHOSPHORIBOSYLTRANSFERASE (PTHR43340:SF7)
	HUMAN|HGNC=6178|UniProtKB=P23677	Inositol-trisphosphate 3-kinase A;ITPKA;ortholog	INOSITOL-TRISPHOSPHATE 3-KINASE A (PTHR12400:SF62)
	HUMAN|HGNC=6040|UniProtKB=Q13418	Integrin-linked protein kinase;ILK;ortholog	INTEGRIN-LINKED PROTEIN KINASE (PTHR23257:SF671)
HUMAN|HGNC=14235|UniProtKB=Q9NRN7		L-aminoadipate-semialdehyde dehydrogenase-phosphopantetheinyl transferase;AASDHPPT;ortholog	L-AMINOADIPATE-SEMIALDEHYDE DEHYDROGENASE-PHOSPHOPANTETHEINYL TRANSFERASE (PTHR12215:SF17)
HUMAN|HGNC=2698|UniProtKB=P11182	HUMAN|HGNC=2698|UniProtKB=P11182	Lipoamide acyltransferase component of branched-chain alpha-keto acid dehydrogenase complex, mitochondrial;DBT;ortholog	LIPOAMIDE ACYLTRANSFERASE COMPONENT OF BRANCHED-CHAIN ALPHA-KETO ACID DEHYDROGENASE COMPLEX, MITOCHONDRIAL (PTHR43178:SF3)
HUMAN|HGNC=6990|UniProtKB=P51608	HUMAN|HGNC=6990|UniProtKB=P51608	Methyl-CpG-binding protein 2;MECP2;ortholog	METHYL-CPG-BINDING PROTEIN 2 (PTHR15074:SF7)
HUMAN|HGNC=890|UniProtKB=Q13825		Methylglutaconyl-CoA hydratase, mitochondrial;AUH;ortholog	METHYLGLUTACONYL-COA HYDRATASE, MITOCHONDRIAL (PTHR11941:SF104)
HUMAN|HGNC=7064|UniProtKB=O14880		Microsomal glutathione S-transferase 3;MGST3;ortholog	MICROSOMAL GLUTATHIONE S-TRANSFERASE 3 (PTHR10250:SF22)
HUMAN|HGNC=6984|UniProtKB=P23368		NAD-dependent malic enzyme, mitochondrial;ME2;ortholog	NAD-DEPENDENT MALIC ENZYME, MITOCHONDRIAL (PTHR23406:SF50)
	HUMAN|HGNC=7993|UniProtKB=Q9UHY1	Nuclear receptor-binding protein;NRBP1;ortholog	NUCLEAR RECEPTOR-BINDING PROTEIN (PTHR13902:SF100)
HUMAN|HGNC=8998|UniProtKB=P78356	HUMAN|HGNC=8998|UniProtKB=P78356	Phosphatidylinositol 5-phosphate 4-kinase type-2 beta;PIP4K2B;ortholog	PHOSPHATIDYLINOSITOL 5-PHOSPHATE 4-KINASE TYPE-2 BETA (PTHR23086:SF66)

HUMAN|HGNC=8896|UniProtKB=P00558		Phosphoglycerate kinase 1;PGK1;ortholog	PHOSPHOGLYCERATE KINASE 1 (PTHR11406:SF20)
HUMAN|HGNC=19129|UniProtKB=Q9Y617		Phosphoserine aminotransferase;PSAT1;ortholog	PHOSPHOSERINE AMINOTRANSFERASE (PTHR43247:SF2)
HUMAN|HGNC=9393|UniProtKB=P17252		Protein kinase C alpha type;PRKCA;ortholog	PROTEIN KINASE C ALPHA TYPE (PTHR24356:SF270)
HUMAN|HGNC=9395|UniProtKB=P05771	HUMAN|HGNC=9395|UniProtKB=P05771	Protein kinase C beta type;PRKCB;ortholog	PROTEIN KINASE C BETA TYPE (PTHR24356:SF244)
	HUMAN|HGNC=9401|UniProtKB=Q02156	Protein kinase C epsilon type;PRKCE;ortholog	PROTEIN KINASE C EPSILON TYPE (PTHR24356:SF275)
	HUMAN|HGNC=25902|UniProtKB=Q8NAT1	Protein O-linked-mannose beta-1,4-N-acetylglucosaminyltransferase 2;POMGNT2;ortholog	PROTEIN O-LINKED-MANNOSE BETA-1,4-N-ACETYLGLUCOSAMINYLTRANSFERASE 2 (PTHR20961:SF60)
HUMAN|HGNC=8728|UniProtKB=P22061	HUMAN|HGNC=8728|UniProtKB=P22061	Protein-L-isoaspartate(D-aspartate) O-methyltransferase;PCMT1;ortholog	PROTEIN-L-ISOASPARTATE(D-ASPARTATE) O-METHYLTRANSFERASE (PTHR11579:SF14)
	HUMAN|HGNC=11283|UniProtKB=P12931	Proto-oncogene tyrosine-protein kinase Src;SRC;ortholog	PROTO-ONCOGENE TYROSINE-PROTEIN KINASE SRC (PTHR24418:SF279)
	HUMAN|HGNC=49897|UniProtKB=Q6A1A2	Putative 3-phosphoinositide-dependent protein kinase 2;PDPK2P;ortholog	3-PHOSPHOINOSITIDE-DEPENDENT PROTEIN KINASE 1-RELATED (PTHR24356:SF290)
HUMAN|HGNC=8819|UniProtKB=O00764	HUMAN|HGNC=8819|UniProtKB=O00764	Pyridoxal kinase;PDXK;ortholog	PYRIDOXAL KINASE (PTHR10534:SF10)
HUMAN|HGNC=4226|UniProtKB=P31150	HUMAN|HGNC=4226|UniProtKB=P31150	Rab GDP dissociation inhibitor alpha;GDI1;ortholog	RAB GDP DISSOCIATION INHIBITOR ALPHA (PTHR11787:SF19)
HUMAN|HGNC=4227|UniProtKB=P50395		Rab GDP dissociation inhibitor beta;GDI2;ortholog	RAB GDP DISSOCIATION INHIBITOR BETA (PTHR11787:SF21)
HUMAN|HGNC=21332|UniProtKB=Q86UX6		Serine/threonine-protein kinase 32C;STK32C;ortholog	SERINE/THREONINE-PROTEIN KINASE 32C (PTHR24356:SF232)
	HUMAN|HGNC=1097|UniProtKB=P15056	Serine/threonine-protein kinase B-raf;BRAF;ortholog	SERINE/THREONINE-PROTEIN KINASE B-RAF (PTHR23257:SF696)
	HUMAN|HGNC=13386|UniProtKB=Q8TDX7	Serine/threonine-protein kinase Nek7;NEK7;ortholog	SERINE/THREONINE-PROTEIN KINASE NEK7 (PTHR43289:SF2)
HUMAN|HGNC=8527|UniProtKB=P55809	HUMAN|HGNC=8527|UniProtKB=P55809	Succinyl-CoA:3-ketoacid coenzyme A transferase 1, mitochondrial;OXCT1;ortholog	SUCCINYL-COA:3-KETOACID COENZYME A TRANSFERASE 1, MITOCHONDRIAL (PTHR13707:SF45)
HUMAN|HGNC=11834|UniProtKB=P29401	HUMAN|HGNC=11834|UniProtKB=P29401	Transketolase;TKT;ortholog	TRANSKETOLASE (PTHR43195:SF3)
	HUMAN|HGNC=2444|UniProtKB=P41240	Tyrosine-protein kinase CSK;CSK;ortholog	TYROSINE-PROTEIN KINASE CSK (PTHR24418:SF289)
	HUMAN|HGNC=4037|UniProtKB=P06241	Tyrosine-protein kinase Fyn;FYN;ortholog	TYROSINE-PROTEIN KINASE FYN (PTHR24418:SF291)
HUMAN|HGNC=18170|UniProtKB=P30085		UMP-CMP kinase;CMPK1;ortholog	UMP-CMP KINASE (PTHR23359:SF123)
HUMAN|HGNC=857|UniProtKB=P36543		V-type proton ATPase subunit E 1;ATP6V1E1;ortholog	V-TYPE PROTON ATPASE SUBUNIT E 1 (PTHR12317:SF40)

transmembrane receptor regulatory/adaptor protein (PC00226)

HUMAN|HGNC=555|UniProtKB=O43747		AP-1 complex subunit gamma-1;AP1G1;ortholog	AP-1 COMPLEX SUBUNIT GAMMA-1 (PTHR22780:SF35)
HUMAN|HGNC=562|UniProtKB=O94973	HUMAN|HGNC=562|UniProtKB=O94973	AP-2 complex subunit alpha-2;AP2A2;ortholog	AP-2 COMPLEX SUBUNIT ALPHA-2 (PTHR22780:SF34)
HUMAN|HGNC=20879|UniProtKB=Q8WXD9	HUMAN|HGNC=20879|UniProtKB=Q8WXD9	Caskin-1;CASKIN1;ortholog	CASKIN-1 (PTHR24177:SF98)

transporter (PC00227)

HUMAN|HGNC=10990|UniProtKB=P12235	HUMAN|HGNC=10990|UniProtKB=P12235	ADP/ATP translocase 1;SLC25A4;ortholog	ADP/ATP TRANSLOCASE 1 (PTHR24089:SF506)
HUMAN|HGNC=10991|UniProtKB=P05141	HUMAN|HGNC=10991|UniProtKB=P05141	ADP/ATP translocase 2;SLC25A5;ortholog	ADP/ATP TRANSLOCASE 2 (PTHR24089:SF446)
HUMAN|HGNC=10992|UniProtKB=P12236	HUMAN|HGNC=10992|UniProtKB=P12236	ADP/ATP translocase 3;SLC25A6;ortholog	ADP/ATP TRANSLOCASE 3 (PTHR24089:SF411)
	HUMAN|HGNC=619|UniProtKB=Q9BQE5	Apolipoprotein L2;APOL2;ortholog	APOLIPOPROTEIN L1-RELATED (PTHR14096:SF49)
HUMAN|HGNC=823|UniProtKB=P25705	HUMAN|HGNC=823|UniProtKB=P25705	ATP synthase subunit alpha, mitochondrial;ATP5A1;ortholog	ATP SYNTHASE SUBUNIT ALPHA, MITOCHONDRIAL (PTHR43089:SF1)

HUMAN|HGNC=830|UniProtKB=P06576	HUMAN|HGNC=830|UniProtKB=P06576	ATP synthase subunit beta, mitochondrial;ATP5B;ortholog	ATP SYNTHASE SUBUNIT BETA, MITOCHONDRIAL (PTHR15184:SF59)
HUMAN|HGNC=850|UniProtKB=P48047	HUMAN|HGNC=850|UniProtKB=P48047	ATP synthase subunit O, mitochondrial;ATP5O;ortholog	ATP SYNTHASE SUBUNIT O, MITOCHONDRIAL (PTHR11910:SF9)
HUMAN|HGNC=16695|UniProtKB=P51572		B-cell receptor-associated protein 31;BCAP31;ortholog	B-CELL RECEPTOR-ASSOCIATED PROTEIN 31 (PTHR12701:SF28)
HUMAN|HGNC=10982|UniProtKB=O75746	HUMAN|HGNC=10982|UniProtKB=O75746	Calcium-binding mitochondrial carrier protein Aralar1;SLC25A12;ortholog	CALCIUM-BINDING MITOCHONDRIAL CARRIER PROTEIN ARALAR1 (PTHR24089:SF503)
	HUMAN|HGNC=10983|UniProtKB=Q9UJS0	Calcium-binding mitochondrial carrier protein Aralar2;SLC25A13;ortholog	CALCIUM-BINDING MITOCHONDRIAL CARRIER PROTEIN ARALAR2 (PTHR24089:SF428)
HUMAN|HGNC=8011|UniProtKB=P78357	HUMAN|HGNC=8011|UniProtKB=P78357	Contactin-associated protein 1;CNTNAP1;ortholog	CONTACTIN-ASSOCIATED PROTEIN 1 (PTHR10127:SF740)
	HUMAN|HGNC=4572|UniProtKB=P42262	Glutamate receptor 2;GRIA2;ortholog	GLUTAMATE RECEPTOR 2 (PTHR18966:SF269)
HUMAN|HGNC=6400|UniProtKB=Q14974	HUMAN|HGNC=6400|UniProtKB=Q14974	Importin subunit beta-1;KPNB1;ortholog	IMPORTIN SUBUNIT BETA-1 (PTHR10527:SF32)
HUMAN|HGNC=15714|UniProtKB=P42704	HUMAN|HGNC=15714|UniProtKB=P42704	Leucine-rich PPR motif-containing protein, mitochondrial;LRPPRC;ortholog	LEUCINE-RICH PPR MOTIF-CONTAINING PROTEIN, MITOCHONDRIAL (PTHR24015:SF1304)
	HUMAN|HGNC=17587|UniProtKB=Q9Y6C9	Mitochondrial carrier homolog 2;MTCH2;ortholog	MITOCHONDRIAL CARRIER HOMOLOG 2 (PTHR10780:SF23)
	HUMAN|HGNC=19954|UniProtKB=Q9H936	Mitochondrial glutamate carrier 1;SLC25A22;ortholog	MITOCHONDRIAL GLUTAMATE CARRIER 1 (PTHR24089:SF598)
	HUMAN|HGNC=10988|UniProtKB=Q9H1K4	Mitochondrial glutamate carrier 2;SLC25A18;ortholog	MITOCHONDRIAL GLUTAMATE CARRIER 2 (PTHR24089:SF482)
HUMAN|HGNC=10942|UniProtKB=P43007		Neutral amino acid transporter A;SLC1A4;ortholog	NEUTRAL AMINO ACID TRANSPORTER A (PTHR11958:SF78)
HUMAN|HGNC=28911|UniProtKB=O60831		PRA1 family protein 2;PRAF2;ortholog	PRA1 FAMILY PROTEIN 2 (PTHR12859:SF4)
HUMAN|HGNC=16085|UniProtKB=Q9H9B4	HUMAN|HGNC=16085|UniProtKB=Q9H9B4	Sideroflexin-1;SFXN1;ortholog	SIDEROFLEXIN-1 (PTHR11153:SF32)
	HUMAN|HGNC=16073|UniProtKB=Q8TD22	Sideroflexin-5;SFXN5;ortholog	SIDEROFLEXIN-5 (PTHR11153:SF24)
HUMAN|HGNC=10589|UniProtKB=O60939		Sodium channel subunit beta-2;SCN2B;ortholog	SODIUM CHANNEL SUBUNIT BETA-2 (PTHR13869:SF34)
HUMAN|HGNC=805|UniProtKB=P14415	HUMAN|HGNC=805|UniProtKB=P14415	Sodium/potassium-transporting ATPase subunit beta-2;ATP1B2;ortholog	SODIUM/POTASSIUM-TRANSPORTING ATPASE SUBUNIT BETA-2 (PTHR11523:SF36)
	HUMAN|HGNC=11444|UniProtKB=P61764	Syntaxin-binding protein 1;STXBP1;ortholog	SYNTAXIN-BINDING PROTEIN 1 (PTHR11679:SF54)
HUMAN|HGNC=11446|UniProtKB=O00186		Syntaxin-binding protein 3;STXBP3;ortholog	SYNTAXIN-BINDING PROTEIN 3 (PTHR11679:SF44)
HUMAN|HGNC=10979|UniProtKB=P53007	HUMAN|HGNC=10979|UniProtKB=P53007	Tricarboxylate transport protein, mitochondrial;SLC25A1;ortholog	TRICARBOXYLATE TRANSPORT PROTEIN, MITOCHONDRIAL (PTHR24089:SF459)
HUMAN|HGNC=854|UniProtKB=P21281	HUMAN|HGNC=854|UniProtKB=P21281	V-type proton ATPase subunit B, brain isoform;ATP6V1B2;ortholog	V-TYPE PROTON ATPASE SUBUNIT B, BRAIN ISOFORM (PTHR43389:SF1)
HUMAN|HGNC=856|UniProtKB=P21283	HUMAN|HGNC=856|UniProtKB=P21283	V-type proton ATPase subunit C 1;ATP6V1C1;ortholog	V-TYPE PROTON ATPASE SUBUNIT C 1 (PTHR10137:SF6)
HUMAN|HGNC=862|UniProtKB=O95670		V-type proton ATPase subunit G 2;ATP6V1G2;ortholog	PROTEIN ATP6V1G2-DDX39B-RELATED (PTHR12713:SF17)
HUMAN|HGNC=12669|UniProtKB=P21796	HUMAN|HGNC=12669|UniProtKB=P21796	Voltage-dependent anion-selective channel protein 1;VDAC1;ortholog	VOLTAGE-DEPENDENT ANION-SELECTIVE CHANNEL PROTEIN 1 (PTHR11743:SF37)
HUMAN|HGNC=12674|UniProtKB=Q9Y277	HUMAN|HGNC=12674|UniProtKB=Q9Y277	Voltage-dependent anion-selective channel protein 3;VDAC3;ortholog	VOLTAGE-DEPENDENT ANION-SELECTIVE CHANNEL PROTEIN 3 (PTHR11743:SF42)

Supplementary table V. Fungal species identified in the homogenate and P7 fractions from AD1 and AD2 patients.

Species	Patients	Sequence 5´---3´
Cladosporium sp	AD2P7	**ITS2**:GTGATTGCAGATTCAGTGAATCATCAGAATCTTTAGAACGCACATTGCGCCCCCTGG<br>TATTCCGGGGGGCATGCCTGTTCGAGCGTCATTTCACCACTCAAGCCTCGCTTGGTATTGG<br>GCAACGCGGTCCGCCGCGTGCCTCAAATCGACCGGCTGGGTCTTCTGTCCCCTAAGCGTTG<br>TGGAAACTATTCGCTAAAGGGTGTTCGGGAGGCTACGCCGTAAAACAACCCCATTTCTAA<br>GGTTGACCTCGGATCAGGTAGGGATACCCGCTGAACTTAAGCATATCAATAAGCGGAGGA<br>AAGCACTCAAACAGGCATGCTCTACGGATTGCCATAGAGCGCAAGGTGCGTTCAA
Malassezia globosa	AD2P7	**ITS2**:GTGATTGCAGATTCCGTGAATCATCAGAATCTTTAGAACGCACCTTGCGCTCTATGGT<br>ATTCCGTAGAGCATGCCTGTTTGAGTGCCGTGAATTCTCCCATCCCAAGCGGTTTTTATCAA<br>AGAATTGCTAGGCGAAGGGGTTGAGATGGGCGTTGTTATAACTGCTTCTCTTCTCTAGAA<br>AAGCTCGCCCGAAAAACAGCTAACGCCTCTGGGCCACTTTGCATCCGCTTCTCTGAGGGG<br>AGAAGCGGCCAAGCGCGCTCTGATCATCAGGCATAGCATGATACGTCATTTGCTATGCTG<br>TAGGAGAGCATTTGGTTGTGGTTATACCGCGTGCGTCATTTTTTTTTGCAAACGCAAAAAA<br>AA
Malassezia restricta	AD1H	**ITS2**:GTGATTGCAGATTCCGTGATCATCGAATCTTTGAACGCACCTTGCGCTCTATGGCAAT<br>CCGTAGAGCATGCCTGTTTGAGTGCCATGAAATCTCCCACCCCAAGCGGTTTTTACATGAA<br>ACGGCTTGGCGGATGGGGTCTGGATGGGTGCCTCTGCCTGCGCTACCTAGCACAGGCTCG<br>CCCGAAATGCATGAGCGCCTTGAGACACTTTGCATCCGCCTCTCTGTTTGGGAGGAGGCG<br>GCCAAGCAGTGTTTTTCTCCTGGCATGGCATGATACGTCATTTGCTATGTCGCCTAAAGGA<br>GGAATGTTTGGTTGTGTCTGCGTGTGCTTCGAACTTGCCTCTGTGGCACATCCCAATTTCAC<br>TTCTGGTCTCAAATCAGGTAGGATCACCCGCTGAACTTAAGCATA
	AD1P7	**ITS1**:CGGACGCCACAAAGTGTCCCTGGCCGCCTACACCCACTATACATCCACAAACCCGTG<br>TGCACTGTCCTTGGAAAGGGCATTTGGAGAGAAAAAGGAAGAGAATCGAGAACAGTGCG<br>ATCCGTCCCGTTGGGCGTTGTTCTCTGGCTTGTGCTCTCTGAGGCCCTTTCCTCCCTACAAA<br>CTCAAAGGGTTATAATAAACGTGAAAATTATTTGGACCGTCCCTGGCCAAAAAATTATACA<br>CAATTTTCAAAAACGGATAAAAAAGAGATCCCCCCGACTGTTTC
Rhodotorula mucilaginosa	AD2P7	**ITS1**:TTGGAGTCCGACTCTCACTTTCTAACCCTGTGCACTTGTTTGGGATAGTAACTCTCGC<br>AAGAGAGCGAACTCCTATTCACTTATAAACACAAAGTCTATGAATGTATTAAATTTTATAA<br>CAAAATAAAACTTTCAACAACGGATCAAAATTCTAATGAATGTATTCGTTTTATATAAACAT<br>AATAATAATTTTTACTATTTATTAATTTTGGTTATATT
Uncultured basidiomycota	AD2P7	**ITS1**:TACGGACGCCAAAGTGTCCCTGGCCGCCTACACCCACTATACATCCACAAACCCGTG<br>TGCACTGTCTTGGAGAAAGGCTTCAGAGAAGTTTTTTGTGGCCTCTCTTGGGGTCTTTCTTC<br>GCTACAAACTCGAATGGTTAGTATGAACGTGGAACTTGGTTGGACCGTCACTGGCCAACA<br>AACTATACACAACTTTCAACAACGGATACAAAGGGCGAAGCAGGACCCTCGAGAGGCATC<br>AATTTGCTTTTTTGTAGAGTTTTTTGACTCTGAGACTAGTC

SUPPLEMENTARY FIGURE 2

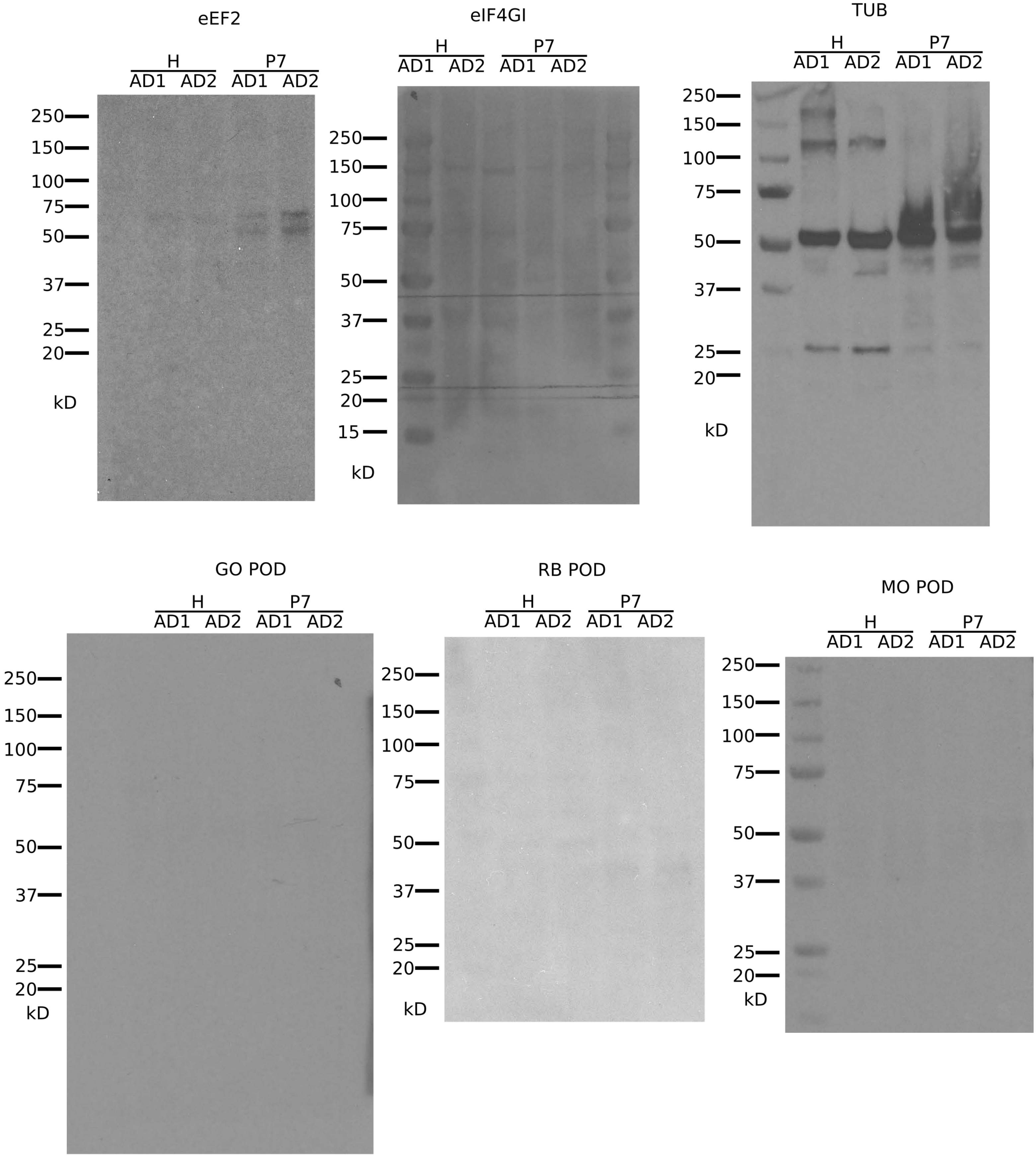

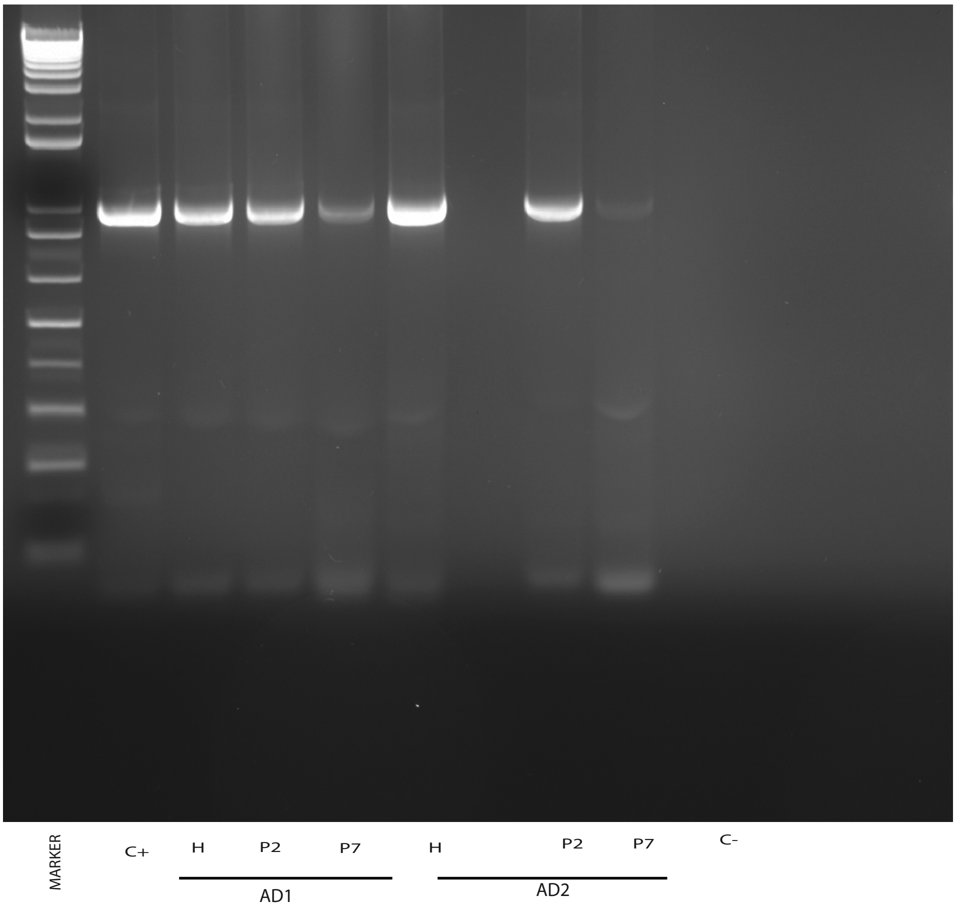
MARKER
C+
H
P2
P7
H
P2
P7
C-
AD1
AD2

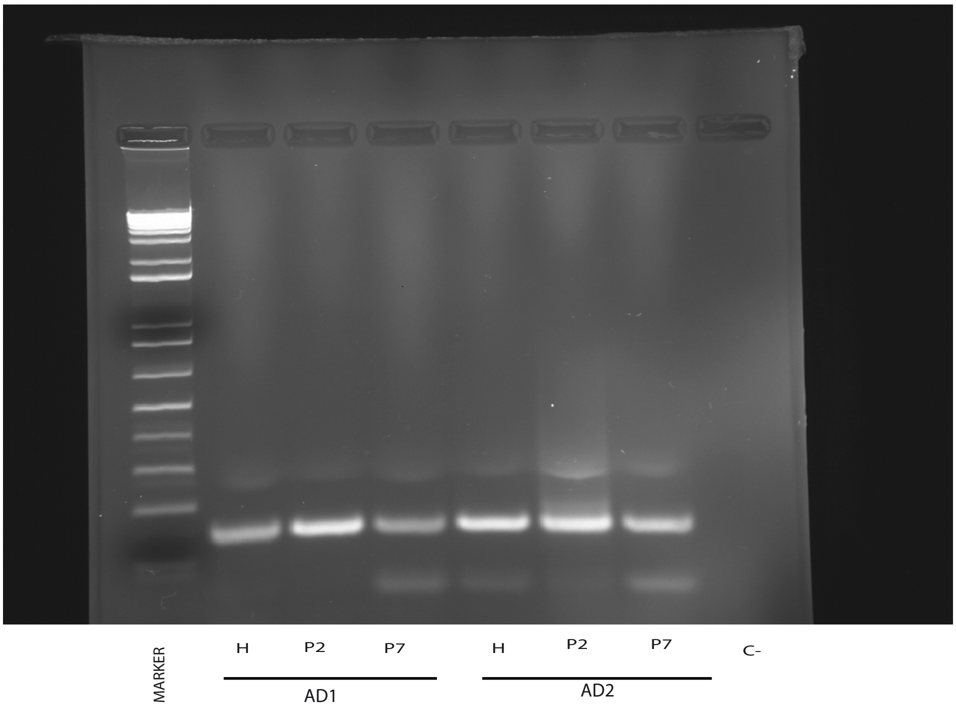
MARKER
H
P2
P7
H
P2
P7
C-
AD1
AD2

FIGURE 3

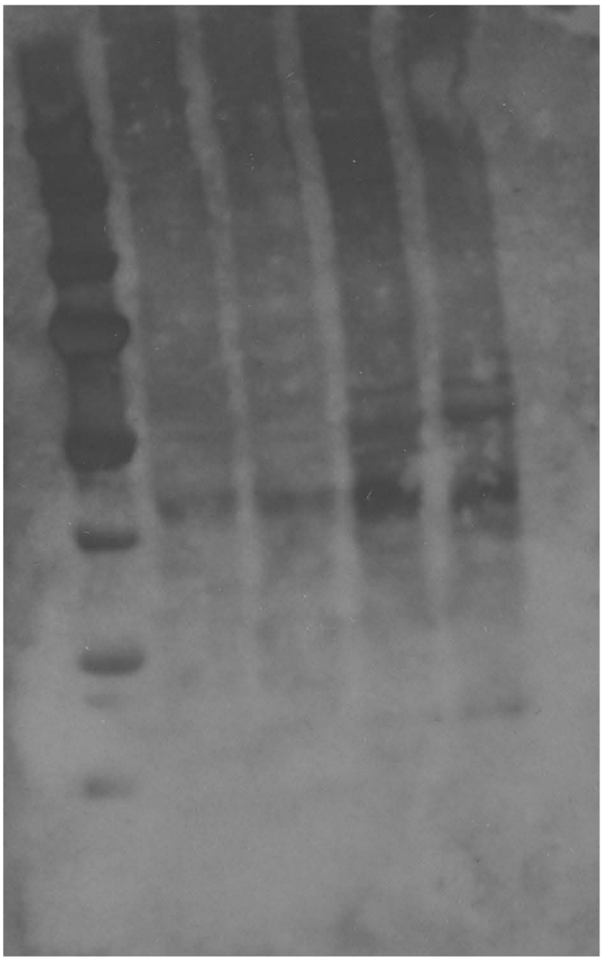

P. betae

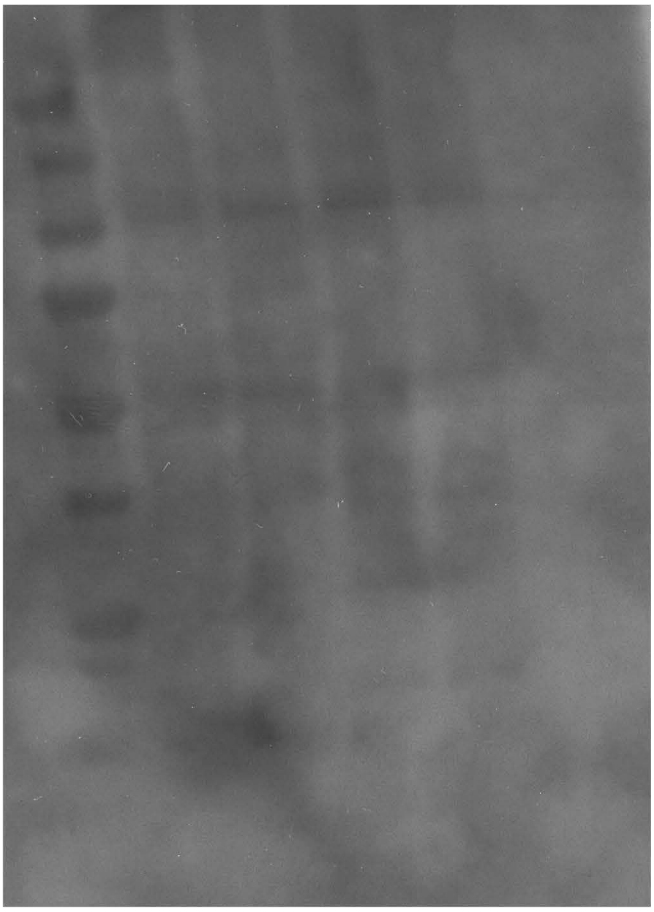

C. albicans

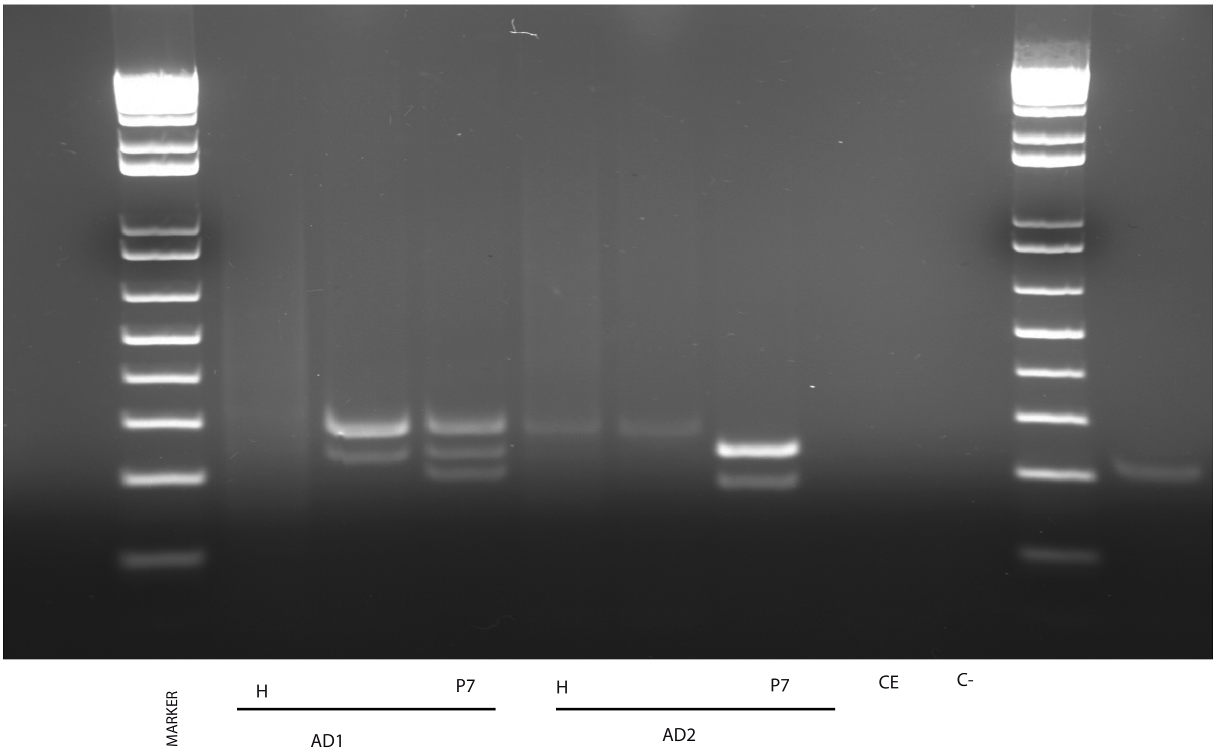
MARKER
H
P7
AD1
H
P7
AD2
CE
C-

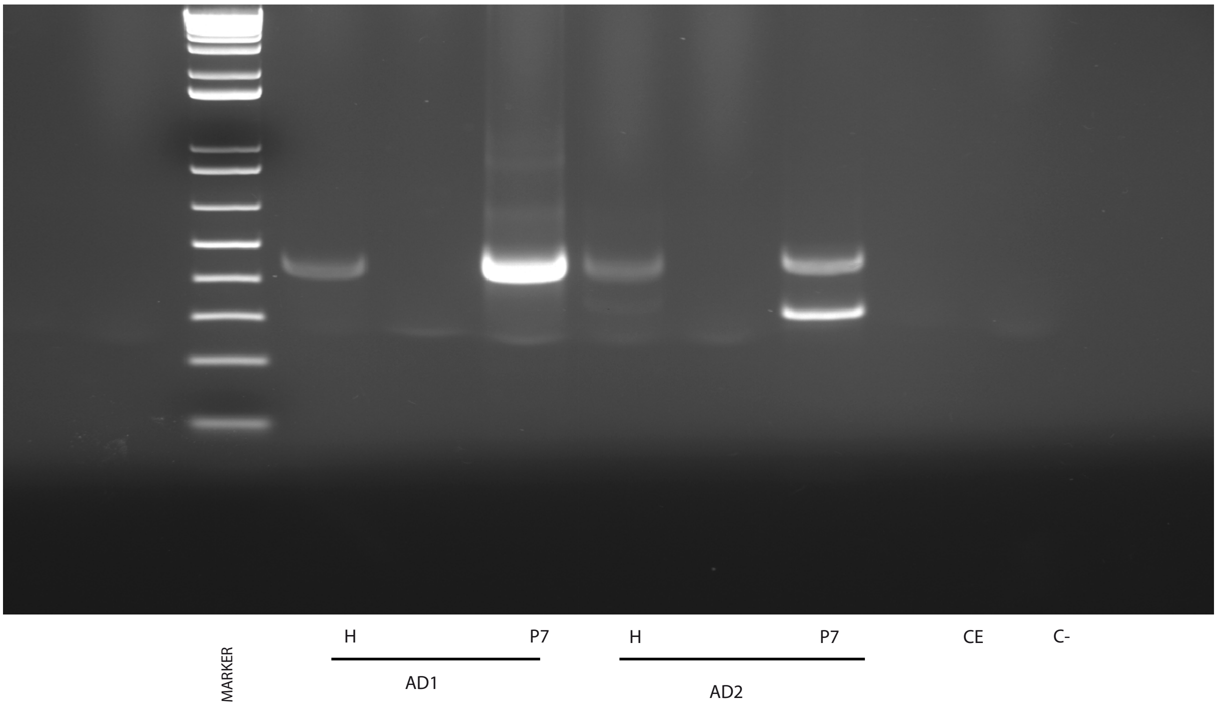
MARKER
H
P7
AD1
H
P7
AD2
CE
C-
